# Stereocontrolled Synthesis of Chiral Helicene‐Indenido *ansa*‐ and Half‐Sandwich Metal Complexes and Their Use in Catalysis

**DOI:** 10.1002/anie.202414698

**Published:** 2024-11-14

**Authors:** Tereza Edlová, Jiří Rybáček, Hélène Cattey, Jaroslav Vacek, Lucie Bednárová, Pierre Le Gendre, Adrien T. Normand, Irena G. Stará, Ivo Starý

**Affiliations:** ^1^ Institut de Chimie Moléculaire de L'Université de Bourgogne (ICMUB) 9 avenue Alain Savary Dijon 21000 France; ^2^ Institute of Organic Chemistry and Biochemistry Czech Academy of Sciences Flemingovo nám. 542/2 160 00 Prague 6 Czech Republic

**Keywords:** helicenes, half-sandwich complexes, *ansa*-metallocene, enantioselective catalysis, C−H activation

## Abstract

Despite recent tremendous progress in the synthesis of nonplanar chiral aromatics, and helicenes in particular, their conversion to half‐sandwich or sandwich transition metal complexes still lags behind, although they represent an attractive family of modular and underexplored chiral architectures with a potential catalytic use. In this work, starting from various chiral helicene‐indene proligands, we prepared the enantio‐ and diastereopure oxa[6]‐ and oxa[7]helicene‐indenido half‐sandwich Rh^I^ and Rh^III^ complexes and oxa[7]helicene‐bisindenido *ansa*‐metallocene Fe^II^ complex. To document their use, oxahelicene‐indenido half‐sandwich Rh^III^ complexes were employed as chiral catalysts in enantioselective C−H arylation of benzo[*h*]quinolines with 1‐diazonaphthoquinones to afford a series of axially chiral biaryls in mostly good to high yields and in up to 96 : 4 *er*. Thus, we developed stereocontrolled synthesis of chiral helicene‐indenido *ansa*‐ and half‐sandwich metal complexes, successfully demonstrated the first use of such helicene Cp‐related metal complexes in enantioselective catalysis, and described an unusual sequence of efficient central‐to‐helical‐to‐planar‐to‐axial chirality transfer.

## Graphical abstract

Stereocontrolled syntheses of chiral (oxa)helicene‐indenido half‐sandwich (Rh) or *ansa*‐metallocene (Fe) complexes were developed. The enantio‐ and diastereopure half‐sandwich Rh(III) complexes were used as catalysts in enantioselective C−H arylation to form axially chiral biaryls in up to 96 : 4 *er*, and an unusual sequence of efficient central‐to‐helical‐to‐planar‐to‐axial chirality transfer was described.

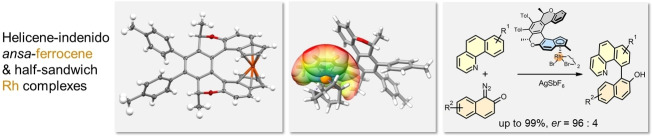

## Introduction

**Figure 1:**
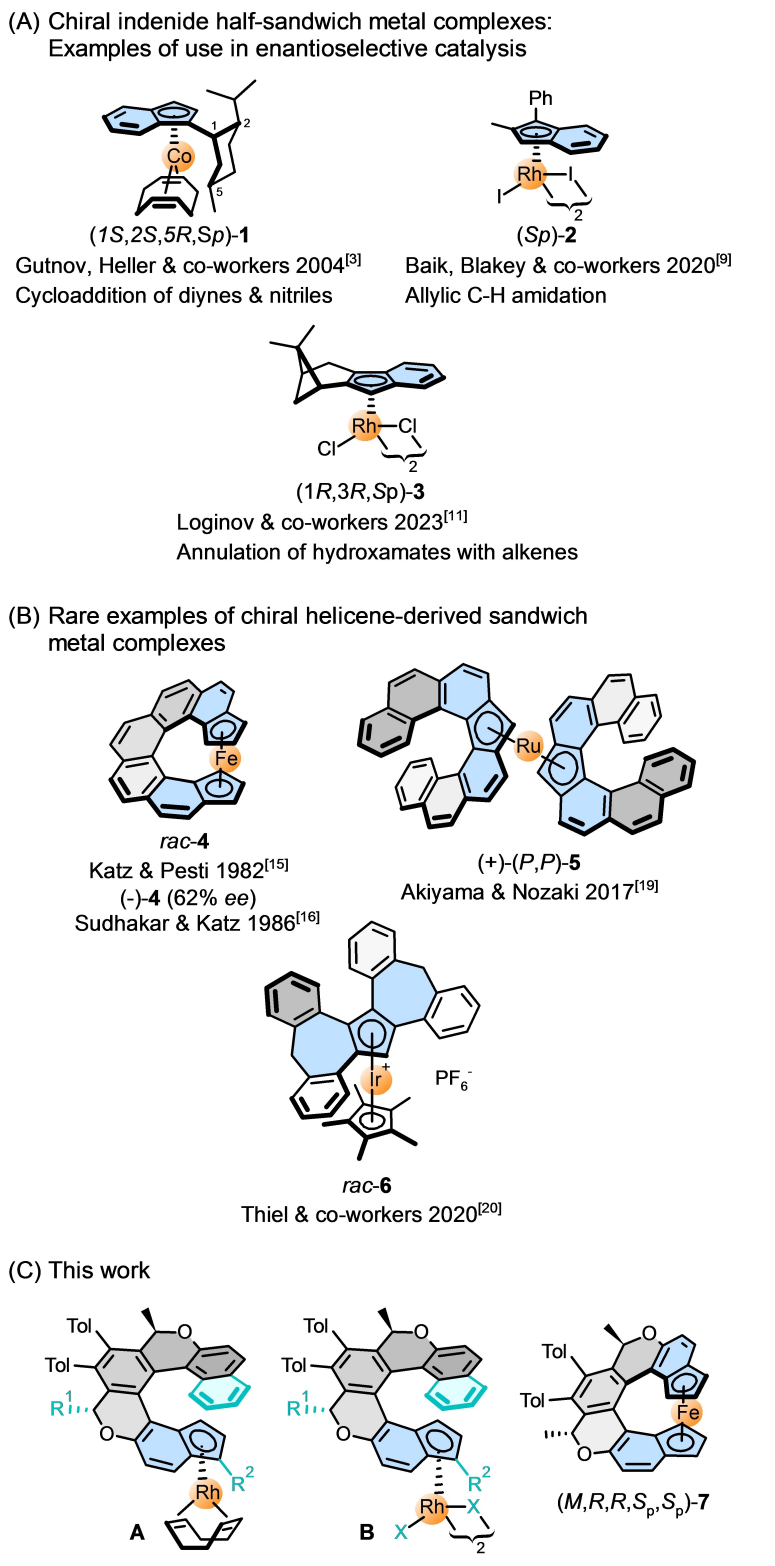
A: Examples of chiral indenido half‐sandwich metal complexes used in enantioselective catalysis. B: Rare chiral helicene‐derived indenido, fluorenido and other sandwich metal complexes, not yet examined in catalysis. C: The chiral (oxa)helicene‐indenido half‐sandwich Rh^I^ (**A**) and Rh^III^ (**B**) complexes (structural variations marked in dark turquoise) and the *ansa*‐metallocene Fe^II^ complex **7** reported in this work were prepared enantio‐ and diastereopure; the Rh^III^ complexes were used in enantioselective C−H arylation with diazonaphthoquinones.

Benzannulated Cp ligands can be classified into indenide or fluorenide types with the Cp unit fused to the end of the polyaromatic system or embedded in its core, respectively. The corresponding half‐sandwich metal complexes are relatively abundant in the chemical literature,^[1]^ but there are only scattered examples of their use in enantioselective catalysis. Regarding chiral indenide ligands, Schumann and co‐workers pioneered the preparation and use of 1‐ and 2‐menthylindenido Rh^I^ complexes in asymmetric hydrogenation,^[2]^ Gutnov, Heller, and co‐workers,[^3^, ^4^] Hapke and co‐workers,[^5^, ^6^] and Heller, Stará, and co‐workers^[7]^ successfully applied 1‐menthylindenido Co^I^ complexes (e.g., (−)‐(1*S*,2*S*,5*R*,*S*_p_)‐**1**)^[8]^ in asymmetric [2+2+2] cycloaddition of alkynes or alkynes/nitriles. Baik, Blakey, and co‐workers employed a planar chiral Rh^III^ indenido catalyst (e.g., (*S*)‐**2**) in enantioselective allylic C−H amidation^[9]^ and alkene aziridination,[^9^, ^10^] and Loginov and co‐workers used an α‐pinene‐derived indenido Rh^III^ complex (e.g., (1*R*,3*R*,*S*_p_)‐**3**) in enantioselective annulation of hydroxamates with alkenes^[11]^ (Figure 1A). Chiral fluorenide ligands were used in enantioselective catalysis only rarely with still unconvincing results as reported by Gutnov, Heller, and co‐workers finding low catalytic activity of a respective Co complex in asymmetric [2+2+2] cycloaddition of alkynes (and optionally alkynes/nitriles).[^3^, ^4^]

Focusing on higher homologs with the Cp unit annulated to or embedded into an inherently chiral polyaromatic helicene scaffold,^[12]^ such systems are still rare, underexplored and, to the best of our knowledge, Cp‐helicene metal complexes have never been applied to enantioselective catalysis. Only cycloiridated helicenes with an external Cp* ligand at iridium were recently used by us as efficient chiral catalysts in the asymmetric transfer hydrogenation of imines.^[13]^ In a broader context, Cp‐helicene metallocenes were pioneered by Katz and Šlusarek in 1979 reporting, *inter alia*, a dimeric bisferrocene derived from Cp‐[5]helicene^[14]^ (counting Cp and benzene units together, all of which are *ortho* fused). Katz and co‐workers described also other monomeric, dimeric, and polymeric metallocenes (Fe, Co) with Cp‐[7]‐ (e.g., **4**),[^15^, ^16^] Cp‐[8]‐,^[17]^ and Cp‐[9]helicene^[18]^ scaffolds, some of them optically pure (Figure 1B). Akiyama and Nozaki prepared metallocenes (Fe, Ru) with coordinated Cp‐[7]helicene ligand(s) (e.g., **5**)^[19]^ also in optically pure form with interesting chiroptical properties. Thiel and co‐workers paid systematic attention to benzannulated homologs of the Cp unit^[1]^ including intrinsically chiral [5]helicene proligands and corresponding sandwich or half‐sandwich metal (Ti, Fe, Rh, Ir, Ru) complexes (e.g., **6**)^[20]^ focusing on their spectroscopic, structural and dynamic characterization.[^21^, ^22^, ^23^, ^24^, ^25^, ^26^]

Herein, we report on the preparation of the enantio‐ and diastereopure (oxa)helicene‐indenido Rh^I^ (**A**) and Rh^III^ (**B**) complexes and *ansa*‐metallocene Fe^II^ complex (*M*,*R*,*R*,*S*_p_,*S*_p_)‐**7** (Figure 1C). The helical Rh^III^ complexes were used as chiral catalysts in enantioselective C−H arylation of benzo[*h*]quinolines with 1‐diazonaphthoquinones to afford a series of axially chiral biaryls in mostly good to high yields and in up to 96 : 4 *er*. All planar stereogenic centers in the article were assigned according to the most recent literature.^[27]^

## Results and Discussion

### Synthesis of Helicene‐Indene Proligands

**Scheme 1:**
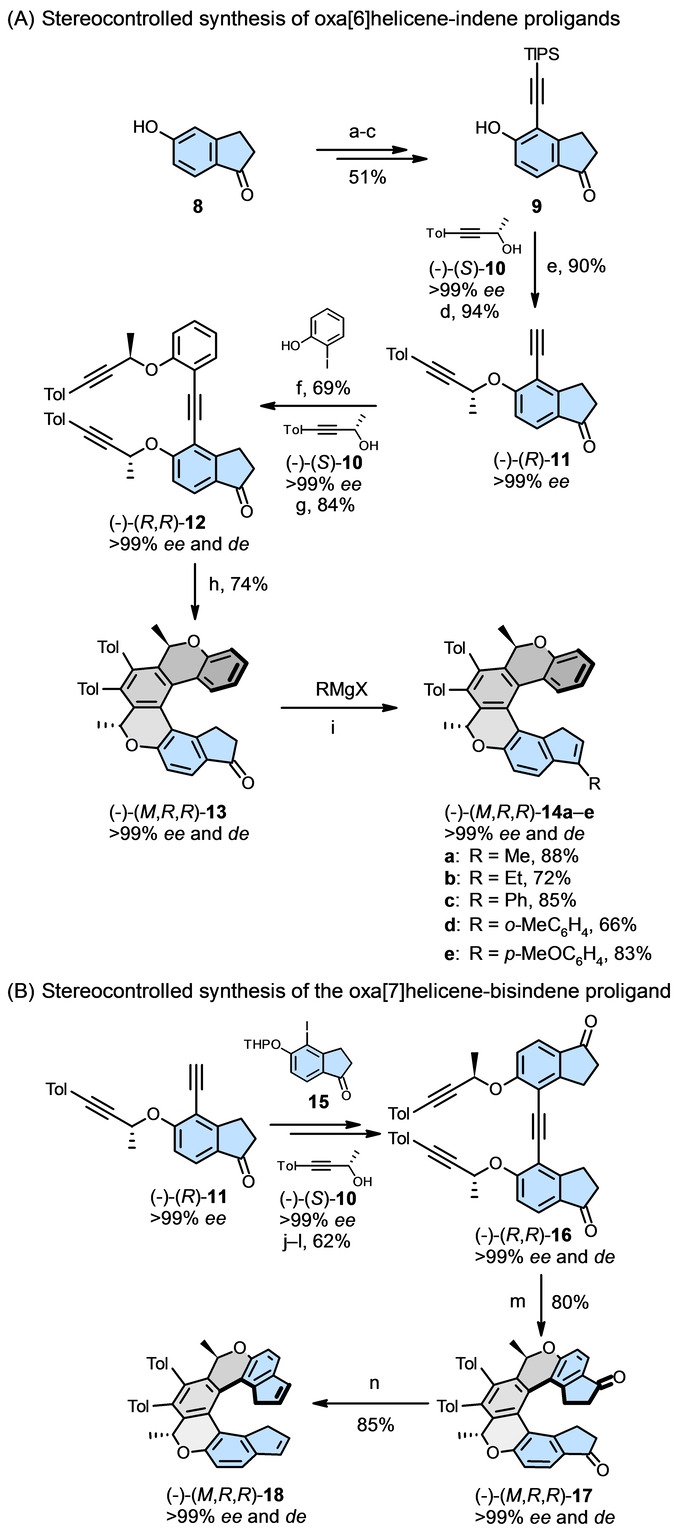
Illustrative synthesis of enantio‐ and diastereopure helicene‐indene proligands (−)‐(*M*,*R*,*R*)‐**14 a**–**e** and (−)‐(*M*,*R*,*R*)‐**18**. Reaction conditions: (a) NIS (1.0 equiv.), DMF, 0 °C, 8.5 h, then AcCl (1.5 equiv.), Et_3_N (2.5 equiv.), DCM, 0 °C to rt, 30 min, 60 %; (b) TIPS‐acetylene (3.0 equiv.), Pd(PPh_3_)_2_Cl_2_ (5 mol %), CuI (10 mol %), Et_3_N‐toluene (1 : 2), 40 °C, 3 h, 88 %; (c) K_2_CO_3_ (2.0 equiv.), MeOH, rt, 20 min, 97 %; (d) (−)‐(*S*)‐4‐(*p*‐tolyl)‐3‐butyl‐2‐ol **10** (1.2 equiv.), PPh_3_ (1.1 equiv.), DIAD (1.2 equiv.), benzene, 0 °C to rt, 2 h, 94 %; (e) TBAF ⋅ 3H_2_O (1.0 equiv.), THF, 0 °C, 5 min, 90 %; (f) 2‐iodophenol (3.0 equiv.), Pd(PPh_3_)_4_ (5 mol %), CuI (5 mol %), Et_3_N (1.3 equiv.), toluene, 50 °C, 48 h, 70 %; (g) (−)‐(*S*)‐4‐(*p*‐tolyl)‐3‐butyl‐2‐ol **10** (1.2 equiv.), PPh_3_ (1.1 equiv.), DIAD (1.2 equiv.), benzene, 0 °C to rt, 2 h, 84 %; (h) Ni(PPh_3_)_2_(CO)_2_ (20 mol %), toluene, 120 °C, 5 min, 74 %; (i) MeMgBr (1.5 equiv.)/ EtMgBr (1.5 equiv.)/PhMgCl (2.0 equiv.)/*o*‐MeC_6_H_4_MgCl (1.5 equiv.)/*p*‐MeOC_6_H_4_MgBr (1.5 equiv.), THF, −80 °C to rt, 1 h, then *p*‐TsOH (20 mol %), toluene, 110 °C, 1 min, 88 % for **14 a**, 72 % for **14 b**, 85 % for **14 c**, 66 % for **14 d**, 83 % for **14 e**; (j) THP‐protected 5‐hydroxy‐4‐iodo‐2,3‐dihydro‐1*H*‐inden‐1‐one **15** (2.0 equiv.), Pd(PPh_3_)_4_ (5 mol %), CuI (10 mol %), toluene‐Et_3_N (3 : 1), 50 °C, 18 h, 79 %; (k) *p*‐TsOH (10 mol %), THF‐MeOH (1 : 1), rt, 18 h, 91 %; (l) (−)‐(*S*)‐4‐(*p*‐tolyl)‐3‐butyl‐2‐ol **10** (1.2 equiv.), PPh_3_ (1.1 equiv.), DIAD (1.2 equiv.), benzene, 0 °C to rt, 4 h, 86 %; (m) CpCo(CO)(fum) (40 mol %), THF, flow reactor, 250 °C, 80 bar, 0.5 mL/min, 16 min residence time, 80 %; (n) NaBH_4_ (8.0 equiv.), MeOH, rt, 30 min, then *p*‐TsOH (20 mol %), toluene, 110 °C, 1 min, 85 %.

In order to place the annulated indene subunit at the terminal position of the helicene scaffold, where the maximal chiral discrimination can be expected, we used a well‐established methodology for the helicene synthesis, which relies on [2+2+2] cycloisomerization of triynes.[^28^, ^29^] Due to its high modularity with respect to aromatic building blocks from which key triynes are assembled, the synthesis of helicene‐indene proligands could start from the commercially available 5‐hydroxy‐1‐indanone **8**, which in the end forms the embedded indene subunit in proligands **14**, **20**, and **21** (Scheme 1, Table 1). The preparation of enantio‐ and diastereopure oxa[6]helicene‐indene proligands (−)‐(*M*,*R*,*R*)‐**14 a**–**e** well illustrates the general synthetic procedure we employed (Schema 1A). The alkyne sidearms with stereogenic centers in triyne (−)‐(*R*,*R*)‐**12** were attached through Mitsunobu reaction using an enantiopure propargyl‐type alcohol (−)‐(*S*)‐**10** (readily accessible from the commercially available (−)‐(*S*)‐3‐butyn‐2‐ol). Triyne (−)‐(*R*,*R*)‐**12** was subjected to fast [2+2+2] cycloisomerization catalyzed by air‐stable Ni(PPh_3_)_2_(CO)_2_ to afford enantio‐ and diastereopure oxa[6]helicene‐like ketone (−)‐(*M*,*R*,*R*)‐**13**. It is worth noting that *M* helicity of its scaffold is controlled through stereogenic centers by 1,3‐allylic‐type strain, as we demonstrated earlier.[^13^, ^30^, ^31^, ^32^, ^33^] Importantly, (*M*,*R*,*R*)‐**13** is about 8.2 kcal/mol lower in energy than its diastereomer (*P*,*R*,*R*)‐**13** with opposite helicity, as calculated at the DFT B3LYP/Def2TZVP/GD3 level in vacuo using the Gaussian 16 package.^[34]^ Then, upon addition of various Grignard reagents and subsequent acid‐assisted elimination of water, the straightforward synthesis of a small series of enantio‐ and diastereopure oxa[6]helicene‐indene proligands (−)‐(*M*,*R*,*R*)‐**14 a**–**e** was accomplished. Importantly, the opposite enantiomers (+)‐(*P*,*S*,*S*)‐**14 a**,**c**–**e** were simply obtained through the aforementioned asymmetric synthesis starting from the commercially available (+)‐(*R*)‐3‐butyn‐2‐ol (for details, see Supporting Information).

**Table 1:** Preparation of half‐sandwich helicene‐indenido complexes **22**–**24** (Rh^I^) and **25**–**27** (Rh^III^) from helicene‐indene proligands **14**, **20** and **21**.^[a]^

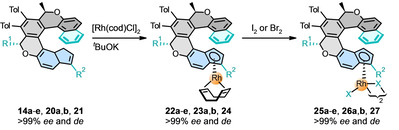					
Entry	Proligand (LH)	Conditions (LH→LRh^I^)	Rh^I^ Complex (LRh^I^)	Conditions (LRh^I^→LRh^III^)	Rh^III^ Complex (LRh^III^)
1	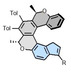		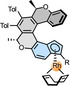		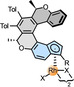
	(−)‐(*M*,*R*,*R*)‐**14 a** R=Me	A	(−)‐(*M*,*R*,*R*,*R*_p_)‐**22 a**, 69 % R=Me	B	(−)‐(*M*,*R*,*R*,*R*_p_)‐**25 a**(I), 97 % R=Me, X=I
2		A		C	(−)‐(*M*,*R*,*R*,*R*_p_)‐**25 a**(Br), 85 % R=Me, X=Br
3	(−)‐(*M*,*R*,*R*)‐**14 b** R=Et	A	(−)‐(*M*,*R*,*R,R_p_*)‐**22 b**, 70 % R=Et	C	(−)‐(*M*,*R*,*R,R_p_*)‐**25 b**, 97 % R=Et, X=Br
4	(−)‐(*M*,*R*,*R*)‐**14 c** R=Ph	A	(−)‐(*M*,*R*,*R*,*R*_p_)‐**22 c**, 58 % R=Ph	C	(−)‐(*M*,*R*,*R*,*R*_p_)‐**25 c**, 78 % R=Ph, X=Br
5	(−)‐(*M*,*R*,*R*)‐**14 d** R=*o*‐MeC_6_H_4_	A	(−)‐(*M*,*R*,*R*,*R*_p_)‐**22 d**, 81 % R=*o*‐MeC_6_H_4_	C	(−)‐(*M*,*R*,*R*,*R*_p_)‐**25 d**, 74 % R=*o*‐MeC_6_H_4_, X=Br
6	(−)‐(*M*,*R*,*R*)‐**14 e** R=*p*‐MeOC_6_H_4_	A	(−)‐(*M*,*R*,*R*,*R*_p_)‐**22 e**, 73 % R=*p*‐MeOC_6_H_4_	B	(−)‐(*M*,*R*,*R*,*R*_p_)‐**25 e**, 79 % R=*p*‐MeOC_6_H_4_, X=I
7	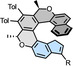		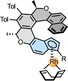		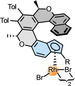
	(−)‐(*M*,*R*,*R*)‐**20 a** R=H	A	(−)‐(*M*,*R*,*R*,*R*_p_)‐**23 a**, 79 % R=H	C	(−)‐(*M*,*R*,*R*,*R*_p_)‐**26 a**, 76 % R=H
8	(−)‐(*M*,*R*,*R*)‐**20 b** R=Me	A	(−)‐(*M*,*R*,*R*,*R*_p_)‐**23 b**, 74 % R=Me	C	(−)‐(*M*,*R*,*R*,*R*_p_)‐**26 b**, 73 % R=Me
9	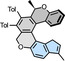		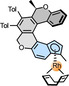		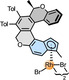
	(−)‐(*M*,*R*)‐**21**	A	(−)‐(*M*,*R*,*R*_p_)‐**24**, 84 %	C	(−)‐(*M*,*R*,*R*_p_)‐**27**, 71 %

[a] Reaction conditions: (A) LH (1.0 equiv.), [Rh(cod)Cl]_2_ (0.55 equiv.), potassium *tert*‐butoxide (1.3 equiv.), THF, rt, 2–4 h; (B) LRh^I^ (1.0 equiv.), I_2_ (2.5 equiv.), diethyl ether, 0 °C, 14–18 h; (C) LRh^I^ (1.0 equiv.), Br_2_ (2.5 equiv.), pentane, 0 °C, 14–18 h. Structural variations of proligands and complexes are marked in dark turquoise.

The *C*_2_‐symmetric oxa[7]helicene‐bisindene proligand (−)‐(*M*,*R*,*R*)‐**18** was synthesized analogously (Scheme 1B). Starting from the aforementioned diyne (−)‐(*R*)‐**11** and THP‐protected 5‐hydroxy‐4‐iodo‐2,3‐dihydro‐1*H*‐inden‐1‐one **15**, they were converted to triyne (−)‐(*R*,*R*)‐**16** in three steps. After stereocontrolled [2+2+2] cycloisomerization, this time catalyzed by CpCo(CO)(fum) (fum=dimethyl fumarate) and carried out in a flow reactor, the helical diketone (−)‐(*M*,*R*,*R*)‐**17** was transformed to the enantio‐ and diastereopure oxa[7]helicene‐bisindene proligand (−)‐(*M*,*R*,*R*)‐**18**.

The synthesis of enantio‐ and diastereopure oxa[7]helicene‐indene proligands (−)‐(*M*,*R*,*R*)‐**20 a**,**b** and oxa[6]helicene‐indene proligand (−)‐(*M*,*R*)‐**21** (with only one stereogenic center to control helicity of the scaffold)^[13]^ is described in Supporting Information.

## Synthesis of Helicene‐Indenido Metal Complexes

**Scheme 2:**
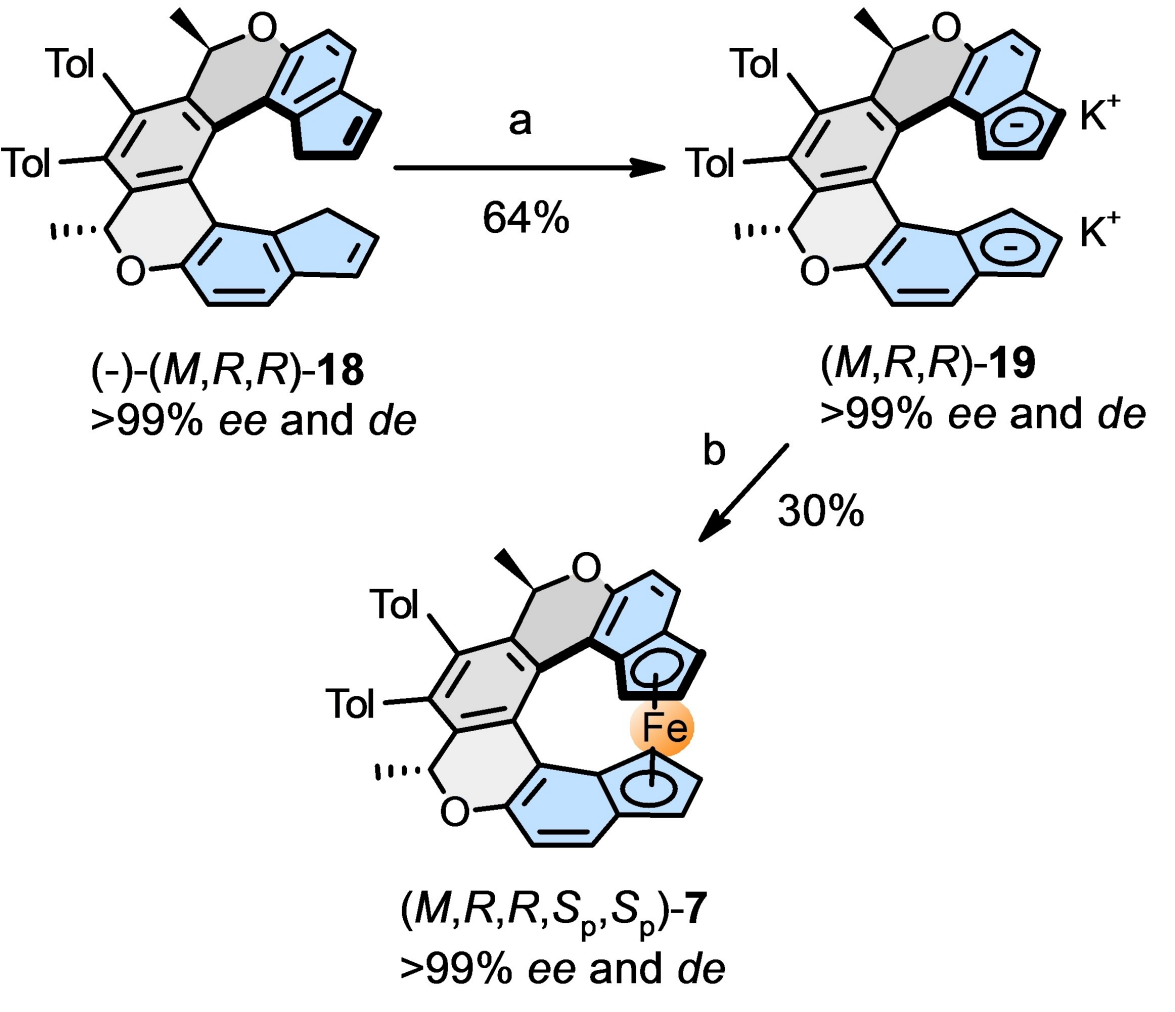
Preparation of the enantio‐ and diastereopure oxa[7]helicene‐bisindenido *ansa*‐ferrocene complex (*M*,*R*,*R*,*S*_p_,*S*_p_)‐**7**. Reaction conditions (in drybox): (a) KHMDS (2.0 equiv.), diethyl ether, rt, 5 min, 64 %; (b) FeCl_2_ (1.0 equiv.), THF, −20 °C to rt, 18 h, 30 %.

**Figure 2:**
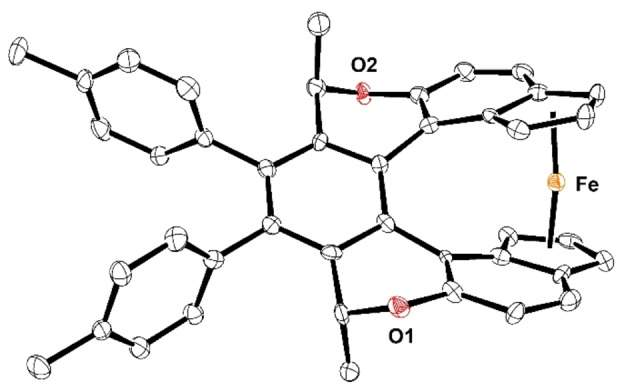
ORTEP depiction of the XRD structure of (*M*,*R*,*R*,*S*_p_,*S*_p_)‐**7** (CCDC 2373549).^[77]^ One pentane solvent molecule and hydrogen atoms are omitted for clarity, thermal ellipsoids are drawn at the 50 % probability level.

With a series of chiral helicene‐indene proligands in hand, we could turn our attention to the preparation of the corresponding metal complexes. First, we embarked on the straightforward synthesis of the enantio‐ and diastereopure oxa[7]helicene‐bisindenido *ansa*‐ferrocene complex (*M*,*R*,*R*,*S*_p_,*S*_p_)‐**7** (Scheme 2). After double deprotonation of the oxa[7]helicene‐bisindene proligand (−)‐(*M*,*R*,*R*)‐**18**, the corresponding potassium bisindenide salt (*M*,*R*,*R*)‐**19** was isolated. It reacted with anhydrous iron dichloride to form the *ansa*‐ferrocene complex (*M*,*R*,*R*,*S*_p_,*S*_p_)‐**7** in moderate isolated yield after recrystallization. Single crystal XRD analysis proved that the bidentate ligand (*M*,*R*,*R*)‐**19** behaves as a tweezer for a single Fe^II^ atom (Figure 2). The “ferrocene” subunit in (*M*,*R*,*R*,*S*_p_,*S*_p_)‐**7** is distorted due to conformational constrains of the oxa[7]helicene‐bisindenyl scaffold, with the angle of tilt between the terminal cyclopentadienyl rings of 16.5(4)°. Actually, such an angle is not uncommon for *ansa*‐ferrocene derivatives (for 16.5±1°, Cambridge Structural Database contains about ninety such cases) and exceptionally can reach up to 33.7° (CCDC 1118743).^[35]^ However, the *ansa*‐ferrocene complex (*M*,*R*,*R*,*S*_p_,*S*_p_)‐**7** decomposes slowly in air. It can be directly compared with the only described helicene‐ferrocene hybrid *rac*‐**4** (Figure 1B), with its tilting angle of 19.8° (CCDC 1117488).[^15^, ^36^] Importantly, the X‐ray structure of (*M*,*R*,*R*,*S*_p_,*S*_p_)‐**7** also confirmed the predicted *M* helicity of the oxa[7]helicene‐bisindenyl scaffold, which is defined by the known absolute *R*,*R* configuration at the stereogenic centers (see the section Synthesis of Helicene‐Indene Proligands for discussion).

**Figure 3:**
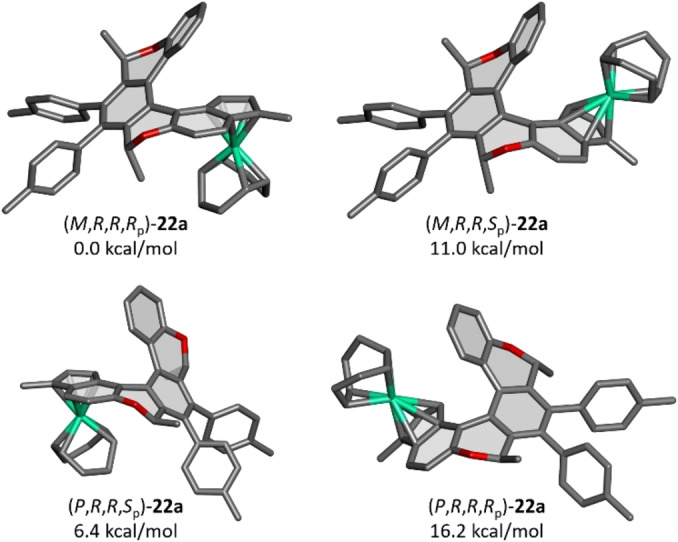
The optimized structure and relative energy of four possible diastereomers of the chiral oxa[6]helicene‐indenido Rh^I^ complex **22 a**, calculated at the DFT B3LYP/Def2TZVP/GD3 level in vacuo using the Gaussian 16 package^[34]^ (hydrogen atoms omitted for clarity; grey: C, red: O, turquoise: Rh).

Bearing in mind the possible catalytic use of chiral helicene‐indenido half‐sandwich metal complexes, we turned our attention to the preparation of the corresponding Rh^I^ and Rh^III^ systems. In general, if the chiral Cp/indene proligand exhibits diastereotopic faces by lacking *C*_2_ symmetry, then η^5^‐coordination of the metal would introduce a new element of planar chirality[^27^, ^37^] (*cf*. the ingeniously employed *C*_2_ symmetry of chiral Cp proligands/half‐sandwich metal complexes by Cramer and co‐workers[^38^, ^39^] and You and co‐workers[^40^, ^41^]). Nevertheless, there are not many examples of fully diastereoselective metal coordination to chiral Cp/indene proligands to form enantio‐ and diastereopure half‐sandwich metal complexes as reported, e.g., by Paquette and Sivik,^[42]^ Baker and Wallace,^[43]^ Sowa Jr. and co‐workers,^[44]^ or recently Loginov and co‐workers (Figure 1, (1*R*,3*R*,*S_p_*)‐**3**).^[11]^ In the case of chiral (oxa)helicene‐indene proligands **14**, **20** and **21**, we assumed that metal coordination to the embedded indene subunit may proceed diastereofacially, since one of its sides is sterically shielded by the other end of the helical scaffold. To our delight, the experiments confirmed this assumption (Table 1). Upon treatment of enantio‐ and diastereopure oxa[6]helicene‐indene proligands (−)‐(*M*,*R*,*R*)‐**14 a**–**e**, their enantiomers (+)‐(*P*,*S*,*S*)‐**14 a**,**d**,**e**, oxa[7]helicene‐indene proligands (−)‐(*M*,*R*,*R*)‐**20 a**,**b**, and oxa[6]helicene‐indene proligand (−)‐(*M*,*R*)‐**21** with a single stereogenic center with [Rh(cod)Cl]_2_ and potassium *tert*‐butoxide, followed by column chromatography on a short pad of silica gel (in air), the enantio‐ and diastereopure Rh^I^ half‐sandwich complexes (−)‐(*M*,*R*,*R*,*R*_p_)‐**22 a**–**e**, (+)‐(*P*,*S*,*S*,*S*_p_)‐**22 a**,**d**,**e**, (−)‐(*M*,*R*,*R*,*R*_p_)‐**23 a**,**b**, and (−)‐(*M*,*R*,*R*_p_)‐**24** were obtained in mostly good yields. Moreover, they could be readily oxidized by iodine or bromine to the corresponding dimeric Rh^III^ half‐sandwich iodido/bromido complexes (−)‐(*M*,*R*,*R*,*R*_p_)‐**25 a**–**e**, (−)‐(*M*,*R*,*R*,*R*_p_)‐**26 a**,**b**, and (−)‐(*M*,*R*,*R*_p_)‐**27** in good to quantitative yields and without stereochemical scrambling, see below. We observed sufficient chemical and thermal stability of the synthesized Rh^I^ and Rh^III^ (oxa)helicene‐indenido half‐sandwich complexes in the solid state, which could be handled in air and stored in the refrigerator under argon for months without noticeable deterioration.

The diastereofacial coordination of Rh^I^ to chiral (oxa)helicene‐indene proligands **14**, **20**, **21** and the configuration of the chiral indenide half‐sandwich subunit (a newly formed element of planar chirality) deserve more detailed discussion. Coordination of Rh^I^ to the representative proligand (−)‐(*M*,*R*,*R*)‐**14 a** can in principle lead to four diastereomeric half‐sandwich complexes with respect to the prochirality of the indene subunit and the possible scaffold flip from *M* to *P* helicity, which would bring the methyl groups at the stereogenic centers from the pseudoaxial to the pseudoequatorial position (Figure 3). The DFT calculations showed that the expected diastereomer (−)‐(*M*,*R*,*R*,*R*_p_)‐**22 a** is indeed preferred because of its by far lowest energy. In accord with this theoretical prediction, the ^1^H NMR spectra of Rh^I^ and Rh^III^ (oxa)helicene‐indenido half‐sandwich complexes **22**–**24** and **25**–**27**, respectively, did not indicate the presence of two (or more) diastereomers, irrespective of analyzing the crude reaction mixture or the purified final product. In the case of complex (−)‐(*M*,*R*,*R*,*R*_p_)‐**22 a**, traces of a side product could be observed by NMR but its low content (approximately 3 mol %) precluded its unambiguous identification.

**Figure 4:**
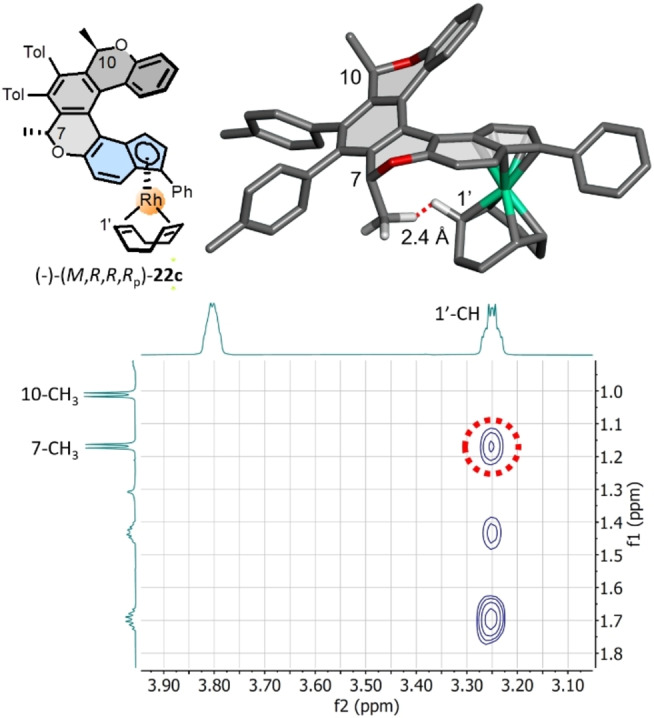
A part of the ^1^H−^1^H NOESY NMR spectrum of the enantio‐ and diastereopure Rh^I^ complex (−)‐(*M*,*R*,*R*,*R*_p_)‐**22 c** showing a through‐space correlation between hydrogen atoms of the *syn* oriented pseudoaxial 7‐CH_3_ group and the 1′‐CH unit of the coordinated COD‐Rh^I^ fragment. The molecular structure was optimized at the DFT B3LYP/Def2TZVP/GD3 level in vacuo using the Gaussian 16 package^[34]^ (only the concerned hydrogens shown; grey: C, red: O, turquoise: Rh, white: H).

Despite all attempts, we did not receive crystals of sufficient quality for single‐crystal XRD analysis as Rh^I^ complexes **22**–**24** and **25**–**27**, were poorly soluble and the crystallization attempts resulted in amorphous precipitates. However, the configuration of the planar‐chiral indenide half‐sandwich subunit was unambiguously determined based on detailed analysis of the ^1^H−^1^H NOESY NMR spectrum of the enantio‐ and diastereopure Rh^I^ complex (−)‐(*M*,*R*,*R*,*R*_p_)‐**22 c** (Figure 4). The *syn* orientation and spatial proximity of the pseudoaxial 7‐CH_3_ group at the stereogenic center and the coordinated COD‐Rh^I^ fragment led to a through‐space correlation between the respective hydrogen atoms of 7‐CH_3_ and 1′‐CH (H−H distance *ca* 2.4 Å), which provided a clearly visible cross‐peak in the NOESY spectrum. This conspicuously indicates the configuration of the complex as the diastereoisomers of (−)‐(*M*,*R*,*R*,*R*_p_)‐**22 c** should display a distance of 5.2–6.7 Å between the corresponding protons of 7‐CH_3_ and 1’‐CH, thus measurable through‐space correlation should not be observed.

**Figure 5:**
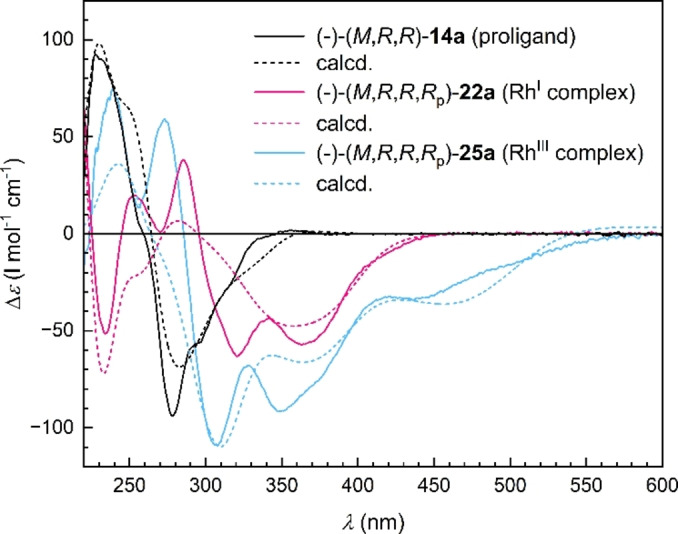
Correlation of the ECD spectra of the enantio‐ and diastereopure oxa[6]helicene‐indene proligand (−)‐(*M*,*R*,*R*)‐**14 a** and the corresponding Rh^I^ and Rh^III^ complexes (−)‐(*M*,*R*,*R*,*R*_p_)‐**22 a** and (−)‐(*M*,*R*,*R*,*R*_p_)‐**25a**(Br), respectively, to demonstrate the conservation of *M* helicity of the scaffold; experimental spectra measured in THF (10^−4^ M); theoretical ECD spectra calculated at the TD‐DFT PBE0/Def2‐TZVP (or Def2‐SVP)/GD3/PCM (in THF) level using the Gaussian 16 package.^[34]^

The stereochemical aspects of the enantio‐ and diastereopure oxa[6]helicene‐indene proligand (−)‐(*M*,*R*,*R*)‐**14 a** and its Rh^I^ and Rh^III^ complexes (−)‐(*M*,*R*,*R*,*R*_p_)‐**22 a** and (−)‐(*M*,*R*,*R*,*R*_p_)‐**25 a**(Br), respectively, were also studied by ECD spectroscopy (Figure 5). The helical scaffold of the proligand (−)‐(*M*,*R*,*R*)‐**14 a** represents the dominating chiral chromophore, which expresses an intense bisignate Cotton effect. Its negative longest‐wavelength band at ~278 nm points to *M* helicity of the molecule, as already established in the family of helicenes and their congeners.[^32^, ^45^, ^46^, ^47^, ^48^, ^49^] In contrast, the ECD spectra of the Rh^I^ and Rh^III^ complexes are more complicated, reflecting the strong electron‐transfer interaction between the oxahelicene‐indenide ligand and the rhodium atom. In such a case, absolute configuration cannot be reliably inferred from the sign of the spectral bands in the long‐wavelength region, as they are most likely the result of a fusion of the helically and planarly chiral chromophores. Stereochemical assignments were thus supported by comparison of experimental data with ECD spectra calculated at the TD‐DFT PBE0/Def2‐TZVP(or Def2‐SVP)/GD3/PCM (in THF) level of theory using the Gaussian 16 software package,^[34]^ showing reasonable agreement (for details, see Supporting Information).

In the context of the discussed chiroptical properties, the specific rotation [α]_D_ as one of the chirality measures practically multiplies upon transition from the helicene‐indene proligands (−)‐(*M*,*R*,*R*)‐**14 a**–**e**, (−)‐(*M*,*R*,*R*)‐**20 a**,**b**, and (−)‐(*M*,*R*)‐**21** to helicene‐indenido Rh^I^ complexes (−)‐(*M*,*R*,*R*,*R*_p_)‐**22 a**–**e**, (−)‐(*M*,*R*,*R*,*R*_p_)‐**23 a**,**b**, and (−)‐(*M*,*R*,*R*_p_)‐**24**, and helicene‐indenido Rh^III^ complexes (−)‐(*M*,*R*,*R*,*R*_p_)‐**25 a**(I)/(Br),**b**–**e**, (−)‐(*M*,*R*,*R*,*R*_p_)‐**26 a**,**b**, and (−)‐(*M*,*R*,*R*_p_)‐**27** (Figure S168).

Calculating the percent buried volume *V*_bur_ of the chiral catalyst and its distribution in space, which can be visualized as a topographic steric map, is one of the useful computational tools for chiral catalyst design. Employing the ChimeraX software[^50^, ^51^, ^52^] and the algorithm implemented in the SEQCROW plugin,[^53^, ^54^] we could compare *V*_bur_ of the chiral binaphthol‐based cyclopentadienido Rh^I^ half‐sandwich complex (−)‐(*R*)‐**28** developed by You and co‐workers^[41]^ and the representative chiral oxa[6]helicene‐indenido Rh^I^ half‐sandwich complex (−)‐(*M*,*R*,*R*,*R*_p_)‐**22 a** studied by us (Table 2). The Cp‐based ligand in the complex (−)‐(*R*)‐**28** exhibits slightly higher steric demands in close proximity to the Rh^I^ atom than the indenide ligand in (−)‐(*M*,*R*,*R*,*R*_p_)‐**22 a** as reflected by both the overall *V*_bur_ (49 % *vs* 43 %) and *V*_bur_ in the most congested SW quadrant (15 % *vs* 13 %). However, inspection of the wider surroundings of the Rh^I^ coordination center (the radius of the integration sphere increased from 3.5 Å to 7.0 Å) revealed the reverse trend, since the indenide ligand in (−)‐(*M*,*R*,*R*,*R*_p_)‐**22 a** is now characterized by a higher overall *V*_bur_ (24 % *vs* 21 %) as well as *V*_bur_ in the most congested SW quadrant (13 % *vs* 11 %) compared to the Cp‐ligand in (−)‐(*R*)‐**28**. Thus, (−)‐(*M*,*R*,*R*,*R*_p_)‐**22 a** expresses favorable overall *V*_bur_ as well as its distribution in individual quadrants to form the desired asymmetric shape of the chiral pocket. Indeed, in line with this computational prediction, both catalysts can be successfully used in enantioselective C−H arylation with diazonaphthoquinones, see below.

**Table 2:** Calculated buried volume of the chiral half‐sandwich Rh^I^ complexes (−)‐(*M*,*R*,*R*,*R*_p_)‐**22 a** and (−)‐(*R*)‐**28**.^[41]^

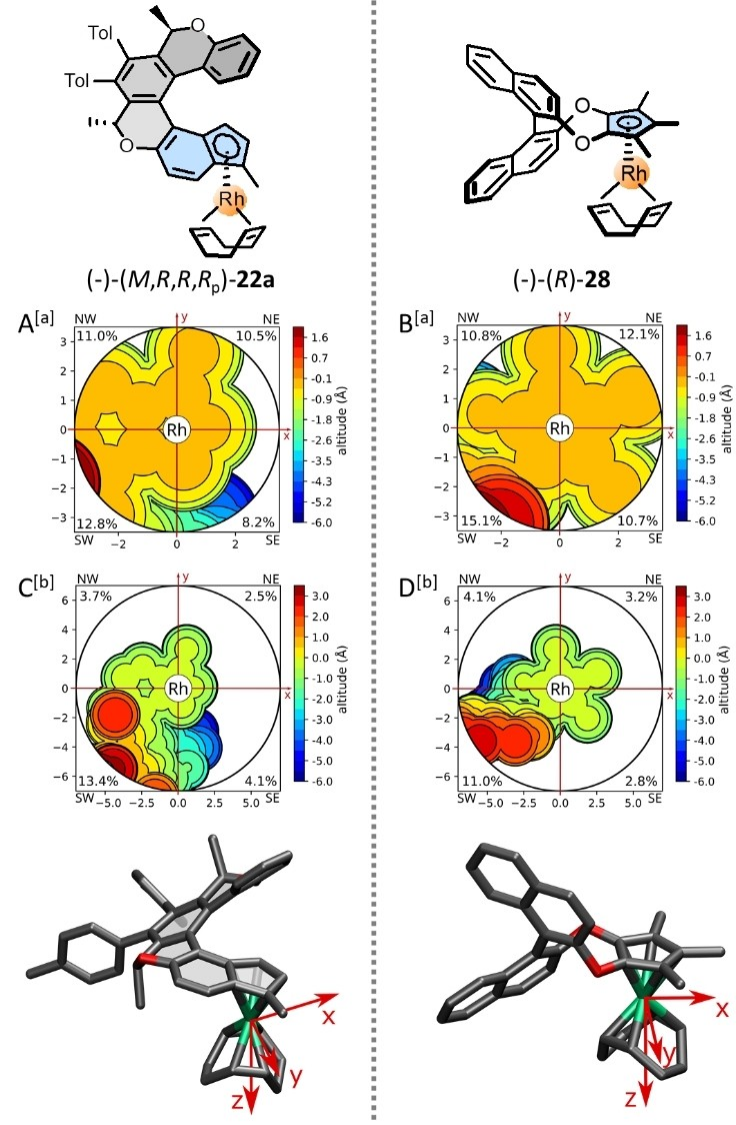				
	(−)‐(*M*,*R*,*R*,*R*_p_)‐**22 a**		(−)‐(*R*)‐**28**	
Integr. radius	3.5 Å	7.0 Å	3.5 Å	7.0 Å
	Buried volume *V*_bur_ (%)^[c]^			
Overall	42.5	23.7	48.7	21.2
NW quadrant	11.0	3.7	10.8	4.1
NE quadrant	10.5	2.5	12.1	3.2
SE quadrant	8.2	4.1	10.7	2.8
SW quadrant	12.8	13.4	15.1	11.0

[a] Figures A, B: integration radius of 3.5 Å. [b] Figures C, D: integration radius of 7.0 Å. [c] quadrant‐resolved percent buried volumes and topographic steric maps were generated by the ChimeraX software[^50^, ^51^, ^52^] using the algorithm implemented in the SEQCROW plugin[^53^, ^54^] (for details, see the Supporting Information).

## Application to Enantioselective Catalysis

Chiral cyclopentadienide (Cp) ligands[^55^, ^56^] and the corresponding half‐sandwich metal complexes are now gaining increasing popularity in enantioselective catalysis.[^57^, ^58^, ^59^] Since the pioneering work in this area by Kagan and co‐workers (1979),^[60]^ Vollhardt and Halterman (1988),^[61]^ and Colletti and Halterman (1989),^[62]^ the renewed interest in their use came after discoveries in 2012, when Ye and Cramer introduced the concept of backwall & sidewalls to the design of *C*_2_ symmetric cyclohexane‐fused Cp ligands^[63]^ and Ward, Rovis, and co‐workers rendered a chiral environment of biotinylated Cp rhodium complexes in the streptavidin cavity.^[64]^ Afterward, several chiral Cp‐ligand families have been developed, e.g., by Cramer and co‐workers,[^38^, ^57^, ^65^, ^66^] You and co‐workers,[^40^, ^41^] Antonchick, Waldmann, and co‐workers,[^67^, ^68^] Wang and co‐workers,[^69^, ^70^, ^71^] or Perekalin and co‐workers.[^72^, ^73^] The respective half‐sandwich metal complexes (Rh, Ir, Ru, Co, Ru, Ln) have been successfully applied as efficient chiral catalysts in various enantioselective processes,[^59^, ^74^] mostly involving C−H activation[^39^, ^40^, ^41^] but also in other reactions such as asymmetric hydrogenation of oximes.^[38]^

**Scheme 3:**
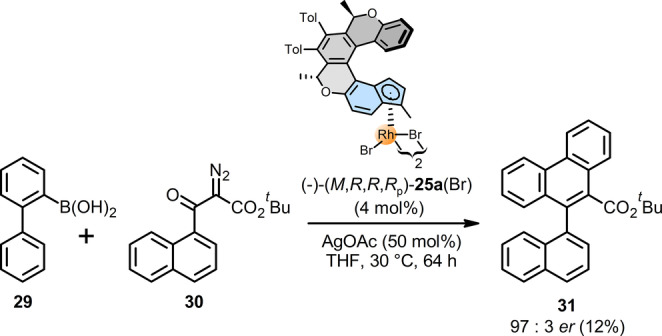
Application of the enantio‐ and diastereopure oxa[6]helicene‐Indenido half‐sandwich Rh^III^ complex (−)‐(*M*,*R*,*R*,*R*_p_)‐**25 a**(Br) in the model Rh^III^‐catalyzed C−H activation associated with atroposelective [4+2] annulative coupling of biphenyl boronic acids with α‐diazo β‐ketoesters.^[75]^

To explore the catalytic activity and chirality transfer efficiency of the Rh^III^ complexes (−)‐(*M*,*R*,*R*,*R*_p_)‐**25 a**(I)/(Br), **b**–**e**, (−)‐(*M*,*R*,*R*,*R*_p_)‐**26 a**,**b**, and (−)‐(*M*,*R*,*R*_p_)‐**27**, we first embarked on Rh^III^‐catalyzed C−H activation associated with atroposelective [4+2] annulative coupling of biphenyl boronic acid **29** with α‐diazo β‐ketoester **30** recently studied by Li, Li, and co‐workers, who obtained a model chiral biaryl **31** in up to *er*=96 : 4 and 68 % yield employing Cramer‐type Cp‐ligands.^[75]^ Intriguingly, the use of the oxa[6]helicene‐indenido half‐sandwich Rh^III^ complex (−)‐(*M*,*R*,*R*,*R*_p_)‐**25 a**(Br) as catalyst in the same model reaction resulted also in high *er*=97 : 3 but low yield of **31** (12 %) due to its insufficient activity (despite the “indenyl effect”^[76]^) or stability^[11]^ (Scheme 3).

To continue screening other enantioselective reactions, where the enantio‐ and diastereopure oxahelicene‐indenido half‐sandwich Rh^III^ complexes (−)‐(*M*,*R*,*R*,*R*_p_)‐**25 a**(I)/(Br), **b**–**e**, (−)‐(*M*,*R*,*R*,*R*_p_)‐**26 a**,**b**, and (−)‐(*M*,*R*,*R*_p_)‐**27** can be applied as chiral catalysts, we turned attention to C−H arylation of benzo[*h*]quinoline with 1‐diazonaphthoquinone to afford axially chiral biaryls recently investigated by You and co‐workers^[41]^ (Table 3). In this case, to our delight, the studied oxahelicene‐indenido half‐sandwich Rh^III^ complexes proved to be effective in terms of catalytic activity as well as enantioselectivity. First, by screening the prepared chiral Rh^III^ catalysts, we found that (−)‐(*M*,*R*,*R*,*R*_p_)‐**25 a**(Br) performed best among its counterparts at 30 °C (87 : 13 *er*, 75 %, entry 4), which showed virtually the same efficiency (91 : 9 *er*, 98 %, entry 5) as the best chiral catalyst (−)‐(*R*)‐**35** by You and co‐workers^[41]^ (92 : 8 *er*, 95 %, entry 2) after lowering the reaction temperature to −20 °C. When the steric bulkiness of the substituent in the outer position 3 on the indenide subunit of (−)‐(*M*,*R*,*R*,*R*_p_)‐**25 a**–**c** was systematically increased by going from Me to Et and Ph, we could observe a gradual and significant decrease in *er* from 87 : 13 to 79 : 21 and 59 : 41 (entries 4, 6, 7). The introduction of the most bulky *o*‐MeC_6_H_4_ group to (−)‐(*M*,*R*,*R*,*R*_p_)‐**25 d** even reversed and slightly increased *er* (entry 8).

**Table 3:** Rh^III^‐Catalyzed enantioselective C−H arylation of benzo[*h*]quinoline **32 a** with 1‐diazonaphthoquinone **33 a**: Screening of chiral catalysts and reaction conditions.

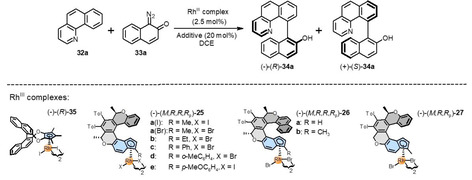						
Entry	Rh^III^ complex	Additive	Temperature (°C)	Time (h)	Yield (%)^[b]^	Er^[c]^ (−)‐(*R*)‐**34 a** : (+)‐(*S*)‐**34 a**
1^[a]^	(−)‐(*R*)‐**35**	AgNO_3_	rt	10	99^[d]^	88 : 12
2^[a]^	(−)‐(*R*)‐**35**	AgNO_3_	−20	48	95^[d]^	92 : 8
3	(−)‐(*M*,*R*,*R*,*R*_p_)‐**25 a**(I)	AgSbF_6_	30	24	50	86 : 14
4	(−)‐(*M*,*R*,*R*,*R*_p_)‐**25 a**(Br)	AgSbF_6_	30	24	75	87 : 13
5	(−)‐(*M*,*R*,*R*,*R*_p_)‐**25 a**(Br)	AgSbF_6_	−20	48	98	91 : 9
6	(−)‐(*M*,*R*,*R*,*R*_p_)‐**25 b**	AgSbF_6_	30	24	79	79 : 21
7	(−)‐(*M*,*R*,*R*,*R*_p_)‐**25 c**	AgSbF_6_	30	24	32	59 : 41
8	(−)‐(*M,R*,*R*,*R*_p_)‐**25 d**	AgSbF_6_	30	24	25	24 : 76
9	(−)‐(*M*,*R*,*R*,*R*_p_)‐**25 e**	AgSbF_6_	30	24	8	71 : 29
10	(−)‐(*M*,*R*,*R*,*R*_p_)‐**26 a**	AgSbF_6_	30	24	96	76 : 24
11	(−)‐(*M*,*R*,*R*,*R*_p_)‐**26 b**	AgSbF_6_	30	24	76	77 : 23
12	(−)‐(*M*,*R*,*R*_p_)‐**27**	AgSbF_6_	30	24	59	72 : 28

[a] You and co‐workers.^[41]^ [b] Determined by ^1^H NMR spectroscopy of the crude reaction mixture using 1,3,5‐trimethoxybenzene as an internal standard. [c] Determined by SFC or HPLC on a chiral column (see the Supporting Information). [d] Using CH_2_Br_2_ as an internal standard.^[41]^

The efficiency of the oxa[6]helicene‐indenido half‐sandwich Rh^III^ complex (−)‐(*M*,*R*,*R*,*R*_p_)‐**25 a**(Br) in chirality transfer is somewhat surprising, since the dominating helical scaffold, which was thought to primarily control chiral discrimination, actually winds away from the catalytic center. To gain a more detailed insight into the role of the various central, planar and helical chirality elements present in the investigated Rh^III^ catalysts, we focused on the oxa[7]helicene‐indenido half‐sandwich Rh^III^ complexes (−)‐(*M*,*R*,*R*,*R*_p_)‐**26 a**,**b** (Table 3). Extending the helical scaffold remotely from the catalytic center resulted in a small but non‐negligible decrease in *er* regardless of the absence/presence of the Me group at position 3 of the indenide subunit (*cf*. entries 10, 11 and 4). Removal of the stereogenic center in the vicinity of the indenide subunit of (−)‐(*M*,*R*,*R*_p_)‐**27**, i.e., deleting the pseudoaxial Me group oriented *syn* to the coordinated Rh^III^ atom, resulted in a similarly modest decrease in *er* (*cf*. entry 12 and 4). Based on these results, it is likely that the element of planar chirality closest to the reaction center governs chirality transfer, but in subtle interplay with the other elements of central and helical chirality present in the oxahelicene‐indenido half‐sandwich Rh^III^ complexes.

Having identified the most efficient oxa[6]helicene‐indenide half‐sandwich Rh^III^ complex (−)‐(*M*,*R*,*R*,*R*_p_)‐**25 a**(Br) and optimized the reaction conditions, we could explore the substrate scope of the Rh^III^‐catalyzed enantioselective C−H arylation of benzo[*h*]quinolines **32 a**–**e** with 1‐diazonaphthoquinones **33 a**–**d** to receive axially chiral biaryls **34 a**–**h** (Table 4). Clearly, using the unfunctionalized benzo[*h*]quinoline component **32 a** and the functionalized 1‐diazonaphthoquinone components **33 a**–**d**, products **34 a**–**d** were obtained in nearly quantitative yields and in high *er* (up to 96 : 4 for (+)‐(*R*)‐**34 d**). Conversely, when placing substituents at the benzo[*h*]quinoline components **32 b**–**e** and using the unfunctionalized 1‐diazonaphthoquinone components **33 a**, the yield and *er* of **34 e**–**h** may depend on a particular substitution pattern. Nevertheless, the oxa[6]helicene‐indenido half‐sandwich Rh^III^ complex (−)‐(*M*,*R*,*R*,*R*_p_)‐**25 a**(Br) successfully competes in all respects with (−)‐(*R*)‐**35** developed by You and co‐workers,^[41]^ and may even the former outperform the latter in some cases.

**Table 4:** Rh^III^‐Catalyzed enantioselective C−H arylation of benzo[*h*]quinolines **32 a**–**e** with 1‐diazonaphthoquinones **33 a**–**d** to deliver axially chiral biaryls **34 a**–**h**: Scope of the reaction.^[a,b,c]^

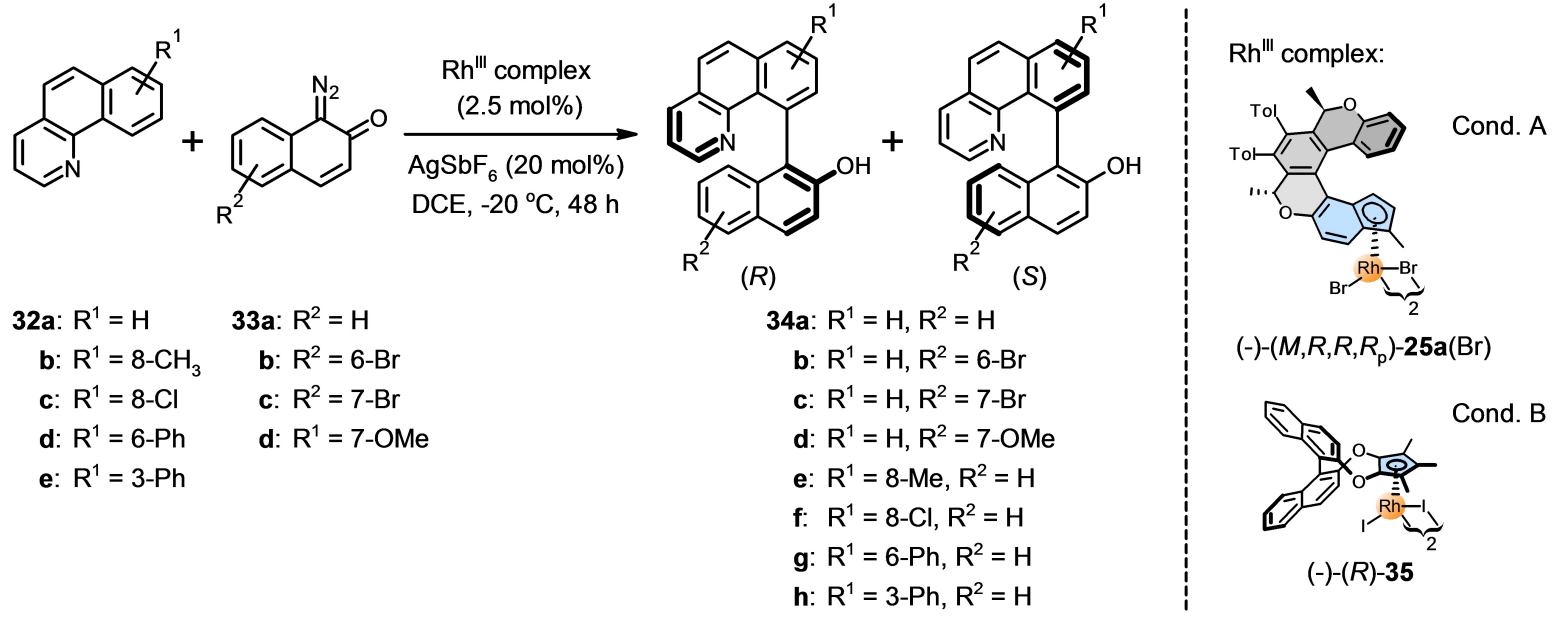				
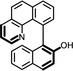		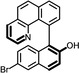	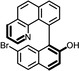	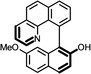
(−)‐(*R*)‐**34 a**		(+)‐(*R*)‐**34 b**	(+)‐(*R*)‐**34 c**	(+)‐(*R*)‐**34 d**
A: 91 : 9 *er*, 98 %^[d]^		A: 93 : 7 *er*, 96 %^[d]^	A: 94 : 6 *er*, 96 %^[d]^	A: 96 : 4 *er*, 99 %^[d]^
B: 92 : 8 *er*, 95 %^[e]^		B: 93 : 7 *er*, 98 %^[e]^	B: 91 : 9 *er*, 90 %^[e]^	B: 92 : 8 *er*, 98 %^[e]^

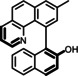		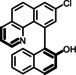	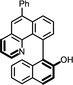	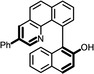
(−)‐(*R*)‐**34 e**		(−)‐(*R*)‐**34 f**	(−)‐(*R*)‐**34 g**	(+)‐(*R*)‐**34 h**
A: 93 : 7 *er*, 72 %^[d]^		A: 83 : 17 *er*, 18 %^[d]^	A: 91 : 9 *er*, 57 %^[d]^	A: 82 : 18 *er*, 24 %^[d]^
N.a.		N.a.	B: 91 : 9 *er*, 97 %^[e]^	B: 95 : 5 *er*, 94 %^[e]^

[a] Yields of isolated products are given. [b] *Er* determined by SFC or HPLC on a chiral column (see the Supporting Information). [c] Absolute configuration of products **34 a**–**d**,**g**,**h** based on the literature data, the stereochemistry of the products **34 a**,**e** was assigned by analogy.^[41]^ [d] Reaction conditions A: Rh^III^ complex (−)‐(*M*,*R*,*R*,*R*_p_)‐**25 a**(Br). [e] Reaction conditions B as published by You and co‐workers:^[41]^ Rh^III^ complex (−)‐(*R*)‐**35** (2.5 mol %), AgNO_3_ (20 mol %), DCE, −20 °C, 48 h.

## Conclusions

In summary, a general synthetic methodology for the preparation of helicenes based on [2+2+2] cycloisomerization of triynes allowed us to efficiently incorporate an indene subunit into the helical scaffold and transform these chiral proligands into the corresponding enantio‐ and diastereopure oxa[6]‐ and oxa[7]helicene‐indenido half‐sandwich Rh^I^ and Rh^III^ complexes, oxa[7]helicene‐bisindenido *ansa*‐metallocene Fe^II^ complex, and racemic dibenzo[6]helicene‐indenido half‐sandwich Rh^I^ and Rh^III^ complexes. We observed a diastereofacial η^5^‐coordinatination of Rh^I^ to the prochiral indenide subunit, leading to the incorporation of central, planar, and helical chirality elements into the same molecule. The respective enantio‐ and diastereopure Rh^III^ complexes were used as chiral catalysts in enantioselective C−H arylation of benzo[*h*]quinolines with 1‐diazonaphthoquinones to afford a series of axially chiral biaryls in mostly good to high yields and in up to 96 : 4 *er*. Based on the experimental results, we assume that the element of planar chirality closest to the reaction center governs chirality transfer, but in subtle interplay with the other elements of central and helical chirality present in oxahelicene‐indenido Rh^III^ complexes. Thus, we developed stereocontrolled synthesis of chiral helicene‐indenido *ansa*‐ and half‐sandwich metal complexes, successfully demonstrated the first use of such helicene Cp‐related metal complexes in enantioselective catalysis, and described an unusual sequence of efficient central‐to‐helical‐to‐planar‐to‐axial chirality transfer.

## Supporting Information

The ESI contains the synthetic procedures, complete experimental details, HPLC traces of the chiral biaryl products, DFT structure optimization, UV/Vis, ECD spectra, and copies of ^1^H and ^13^C NMR spectra of all new compounds (PDF). The authors have cited additional references within the Supporting Information.[^78^, ^79^, ^80^, ^81^, ^82^, ^83^, ^84^, ^85^, ^86^, ^87^, ^88^, ^89^, ^90^, ^91^, ^92^, ^93^, ^94^, ^95^, ^96^, ^97^, ^98^, ^99^]

## Conflict of Interests

The authors declare no conflict of interest.

1

## Supporting information

As a service to our authors and readers, this journal provides supporting information supplied by the authors. Such materials are peer reviewed and may be re‐organized for online delivery, but are not copy‐edited or typeset. Technical support issues arising from supporting information (other than missing files) should be addressed to the authors.

Supporting Information

## Data Availability

The data that support the findings of this study are available from the corresponding author upon reasonable request.
