# Supplementary material for: Stereocontrolled Synthesis of Chiral Helicene‐Indenido ansa‐ and Half‐Sandwich Metal Complexes and Their Use in Catalysis
Source: Angew Chem Int Ed Engl. 2024 Nov 14;64(1):e202414698. doi: 10.1002/anie.202414698 (PMC11701364; doi:10.1002/anie.202414698)
Supplement: Supplementary file 1 — Supporting Information [file ANIE-64-e202414698-s001.pdf]

Angewandte Chemie
Eine Zeitschrift der Gesellschaft Deutscher Chemiker

Supporting Information

Stereocontrolled Synthesis of Chiral Helicene-Indenido *ansa*- and Half-Sandwich Metal Complexes and Their Use in Catalysis

T. Edlová, J. Rybáček, H. Cattey, J. Vacek, L. Bednárová, P. Le Gendre, A. T. Normand, I. G. Stará*, I. Starý**

“Stereocontrolled Synthesis of Chiral Helicene-Indenido *ansa*- and Half-Sandwich Metal Complexes and Their Use in Catalysis”

Supporting Information

Tereza Edlová,* Jiří Rybáček, Hélène Cattey, Jaroslav Vacek, Lucie Bednárová, Pierre Le Gendre, Adrien T. Normand, Irena G. Stará,* and Ivo Starý*

Table of contents

General information....2

Reagents and conditions....2

Analyses....2

Ligand backbone synthesis procedures and characterizations....4

Oxa[6]helicene (+)-(*P*,*S*,*S*)- and (-)-(*M*,*R*,*R*)-**13**....4

Oxa[7]helicene (-)-(*M*,*R*,*R*)-**44**....21

Synthesis of oxa[6]helicene (-)-(*M*,*R*)-**49** with only one chiral center....32

Oxa[7]helicene (-)-(*M*,*R*,*R*)-**17**....42

Synthesis of proligands....50

General procedures....50

Synthesis and characterization of oxa[6]helicene proligands (-)-(*M*,*R*,*R*)- and (+)-(*P*,*S*,*S*)-**14a**-**e**. 51

Synthesis and characterization of oxa[7]helicene proligands (-)-(*M*,*R*,*R*)-**20a**,**b**. 61

Synthesis and characterization of oxa[6]helicene proligand (-)-(*M*,*R*)-**21** with only one chiral center. ..65

Synthesis and characterization of symmetric oxa[7]helicene proligand (-)-(*M,R,R*)-**18** and potassium salt (*M,R,R*)-**19**....67

Synthesis of *ansa*-ferrocene (*M,R,R,*S_p*,*S_p)-**7**....71

Synthesis of Rh(I) and Rh(III) complexes....75

General procedures....75

Oxa[6]helicene complexes of Rh(I) and Rh(III) (-)-(*M*,*R*,*R*,R_p)-**22a**-**e**, (+)-(*P*,*S*,*S*,S_p)-**22a**-**e**, and (-)-(*M*,*R*,*R*,R_p)-**25a**-**e**....76

Oxa[7]helicene complexes of Rh(I) and Rh(III) (-)-(*M*,*R*,*R*,R_p)-**23a**,**b** and (-)-(*M*,*R*,*R*,R_p)-**26a**,**b**....120

Oxa[6]helicene with only one chiral center - complexes of Rh(I) and Rh(III) (-)-(*M*,*R*,R_p)-**24** and (-)-(*M*,*R*,R_p)-**27**....136

UV/VIS spectra of the proligands (-)-(*M*,*R*,*R*)-**14a**-**e**, **18**, **20a**,**b**, (-)-(*M*,*R*)-**21**, potassium salt (*M,R,R*)-**19** and complexes (*M,R,R,*S_p*,*S_p)-**7**, (-)-(*M,R,R,*R_p)-**22a**-**e**, (-)-(*M,R,R,*R_p)-**23a**,**b**, (-)-(*M,R,*R_p)-**24**, (-)-(*M,R,R,*R_p)-**25a**-**e**, (-)-(*M,R,R,*R_p)-**26a**,**b** and (-)-(*M,R,*R_p)-**27**....144

ECD spectra of selected compounds (-)-(*M*,*R*,*R*)- and (+)-(*P*,*S*,*S*)-**14c** and (-)-(*M*,*R*,*R*,R_p)- and (+)-(*P*,*S*,*S*,S_p)-**22e**....146

Specific rotation $[\alpha]_D$ of the helicene-indene proligands (-)-(*M*,*R*,*R*)-**14a–e**, (-)-(*M*,*R*,*R*)-**20a**,**b**, (-)-(*M*,*R*)-**21**, helicene-indenido Rh^{I} complexes (-)-(*M*,*R*,*R*,R_p)-**22a–e**, (-)-(*M*,*R*,*R*,R_p)-**23a**,**b**, (-)-(*M*,*R*,R_p)-**24**, and helicene-indenido Rh^{III} complexes (-)-(*M*,*R*,*R*,R_p)-**25a**(I)/(Br),**b–e**, (-)-(*M*,*R*,*R*,R_p)-**26a**,**b**, and (-)-(*M*,*R*,R_p)-**27**....148

Commented assignment of the NMR spectra of compound (-)-(*M,R,R*,R_p)-**22c**....149

Catalysis....151

Atroposelective [4+2] annulative coupling of biphenyl boronic acid **29**....151

C-H activation of benzo[*h*]quinolines **32** - Catalyst optimization....153

General procedure for catalyst optimization **GP5**....153

Results of catalyst optimization, NMR spectra & HPLC chromatograms of **34a**....154

C-H activation of benzo[*h*]quinolines **32a**-**e** - Scope of the catalysis....166

General procedure **GP6**....166

Results of the catalysis scope, characterization of products **34a-h**, NMR spectra & HPLC chromatograms of **34a-h**....167
DFT calculations and buried volumes 191
Single Crystal X-Ray analysis data for compound (M,R,R,S_p,S_p)-**7** 206
References....215

General information

Reagents and conditions

If not indicated otherwise, the reactions were performed under an inert atmosphere using common Schlenk techniques. Toluene, dichloromethane, Et_2O, pentane, and THF were dried using an MBraun MB SPS-800 solvent purification system or distilled from Na (benzene, toluene, THF) or CaH_2 (Et_3N, diisopropylamine, dichloromethane, dichloroethane). TLC was performed on Silica gel 60 F_{254}-coated aluminum sheets (Merck) and spots were detected by vanillin staining. Column chromatography was performed on Silica gel 60 (0.040-0.063 mm, Fluka) and Silica gel 60 C18 (0.035-0.07 mm, Carl Roth). The starting materials 1-iodo-2-naphthol,[1] (*R*)/(S)-**10**,[2] and CpCo(CO)(fum)[3] were prepared by literature procedures. All other reagents were purchased from commercial suppliers and used as received.

Analyses

The identity and purity of the compounds were established using NMR spectroscopy, high-resolution mass spectrometry (HRMS), optical rotation, IR, and UV/VIS spectroscopies, melting point (Mp), elemental analysis (EA), and X-ray diffraction analysis.

The ^{1}H NMR spectra were measured at 400.13 MHz, 499.88 MHz, and 600.13 MHz, and the ^{13}C NMR spectra at 100.61 MHz, 125.71 MHz, and 150.90 MHz in $CDCl_3$, CD_2Cl_2, DMSO-d_6 or THF-d_8. NMR spectroscopy chemical shifts are quoted in parts per million (δ) relative to TMS (for ^{1}H and ^{13}C), or $BF_3{\cdot}Et_2O$ (for ^{19}F). For ^{1}H and ^{13}C spectra, values were determined by using solvent residual signals (*e.g.* $CDHCl_2$ in CD_2Cl_2) as internal standards.[4] The ^{19}F NMR spectra (in $CDCl_3$) were referenced according to IUPAC recommendations (Absolute referencing). The coupling constants J are given in Hz.

High-resolution ESI mass spectra were recorded using a hybrid FT mass spectrometer combining a linear ion trap MS and the Orbitrap mass analyzer (LTQ Orbitrap XL, Thermo Fisher Scientific) or on a Thermo Scientific Orbitrap Exploris 240. High-resolution EI mass spectra were recorded using Agilent 7250 GC/Q-TOF.

The IR spectra were measured in $CHCl_3$ or KBr on an FT-IR spectrometer Bruker Equinox 55 or by ATR measurement (for powder samples) on an IR FT BRUKER Alpha or Agilent Cary 630 FTIR. UV-VIS spectra were recorded on a SAFAS UVmc2 and the molar absorption coefficients were established by three-point measurements.

IR and UV-Vis spectra of air or moisture-sensitive compounds were measured on Agilent Cary 630 FTIR and an Agilent Cary 60 UV-Vis spectrometer, respectively, in a glovebox under argon.

The optical rotations were measured on an AUTOPOL IV Polarimeter or an Anton Paar MCP 100 in THF or $CHCl_3$. The concentrations are given in g/100mL. The final oxidized complexes were measured in very low concentrations due to their high absorbance at 589 nm. Thus, it has to be taken into account that the precision of these results is limited.

The melting point was determined on a Mikro-Heiztisch Polytherm A (Hund, Wetzlar) apparatus and is uncorrected.

Elemental analyses were measured on CHNS ThermoFisher Scientific Flash EA 1112 analyzer or ThermoFisher Flash*Smart*.

The CD spectra were acquired on a J-1500 CD spectrometer (Jasco Analytical Instruments, Inc.) in THF (10^{-4} M solutions) using a 2 mm quartz sample cell.

Enantiomeric excess of the catalysis products was established on an HPLC Thermo Fisher Vanquish fitted with a Lux 5 µm Cellulose-1 column 250 x 4.6 mm (Phenomenex) using *n*-hexane and isopropyl alcohol as eluents and detected

by a UV detector set at 254 nm or on Waters UPC2 UHPSFC/MS system equipped with PDA and QDa detectors, using 99.995% grade CO_2 and LC-MS grade organic modifiers.

Ligand backbone synthesis procedures and characterizations

Oxa[6]helicene (+)-(*P*,*S*,*S*)- and (-)-(*M*,*R*,*R*)-**13**

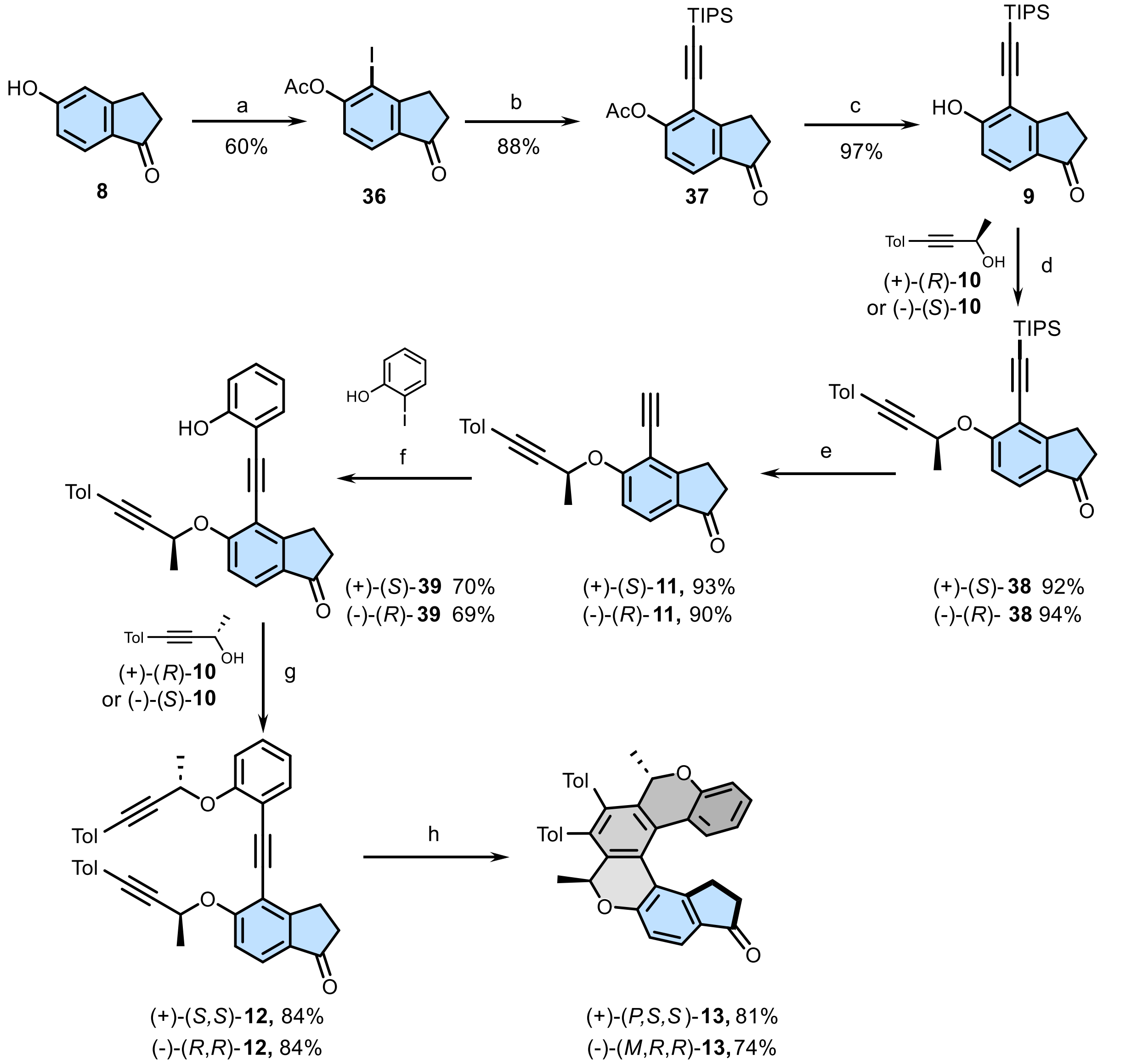

(a) NIS (1.02 equiv.), DMF, 0 °C, 8.5 h, then AcCl (1.5 equiv.), Et_3N (2.5 equiv.), DCM, 0 °C to rt, 30 min, 60%; (b) TIPS-acetylene (3.0 equiv.), $Pd(PPh_3)_2Cl_2$ (5 mol%), CuI (10 mol%), Et_3N-toluene (1:2), 40 °C, 3 h, 88%; (c) K_2CO_3 (2.0 equiv.), MeOH, rt, 20 min, 97%; (d) (+)-(*R*)-**10** (1.2 equiv.), PPh_3 (1.1 equiv.), DIAD (1.2 equiv.), benzene, 0 °C to rt, 2 h, 92% or (-)-(*S*)-**10** (1.1 equiv.), PPh_3 (1.1 equiv.), DIAD (1.2 equiv.), benzene, 0 °C to rt, 2 h, 94%; (e) TBAF·$3H_2O$ (1.0 equiv.), THF, 0 °C, 5 min, 93% for (+)-(*S*)-**11**, 90% for (-)-(*R*)-**11**; (f) 2-iodophenol (3.0 equiv.), $Pd(PPh_3)_4$ (5 mol%), CuI (5 mol%), Et_3N (1.25 equiv.), toluene, 50 °C, 48 h, 70% for (+)-(*S*)-**39**, 69% for (-)-(*R*)-**39**; (g) (+)-(*R*)-**10** or (-)-(*S*)-**10** (1.2 equiv.), PPh_3 (1.1 equiv.), DIAD (1.2 equiv.), benzene, 0 °C to rt, 2 h, 84% for both (+)-(*S*,*S*)-**12** and (-)-(*R*,*R*)-**12**; (h) $Ni(PPh_3)_2(CO)_2$ (20 mol%), toluene, 120 °C, 5 min, 81% for (+)-(*P*,*S*,*S*)-**13**, 74% for (-)-(*M*,*R*,*R*)-**13**.

4-Iodo-1-oxo-2,3-dihydro-1*H*-inden-5-yl acetate 36

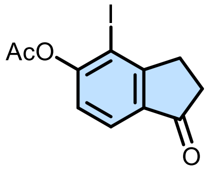

5-Hydroxyindanone **8** (5.00 g, 34 mmol, 1.0 equiv.) was weighed into a dry 250 mL round bottom flask under inert atmosphere and DMF (100 mL, dried on 4Å molecular sieves) was added. The solution was cooled to 0 °C by water/ice bath. Separately, half of the necessary *N*-iodosuccinimide (NIS) solution in dry DMF (1.0 M, prepared from 3.872 g, 17 mmol, in 17 mL of DMF, 0.51 equiv.) was prepared under an inert atmosphere. The solution was added during 3.5 h on the side of the flask (not dropwise). After the addition, a second batch of the NIS solution was prepared (from 3.872 g, 17 mmol, in 17 mL of DMF, 0.51 equiv.) and added immediately after the first half during additional 3.5 h. (This was done to prevent degradation of the solution in the syringe during prolonged standing at room temperature). After the addition was completed, the reaction was allowed to stir for further 90 min. The reaction mixture then was diluted with brine and extracted with ether (3x150 mL). The combined organic layers were washed with brine (100 mL), dried over anhydrous $MgSO_4$, and the solvents were evaporated. The residue was precipitated from MeCN/EtOAc (3:1, 200 mL total) to give the desired iodo derivative (6.450 g, 70%) as a white amorphous solid (^{1}H NMR in DMSO-d_6 shows the presence of about 8 mol% of diiodide, the crude product was used as received).

In a dried 250 mL flask under an inert atmosphere, the isolated mixture after iodination (6.405 g, 23 mmol, 1.0 equiv.) was dissolved in dry dichloromethane (160 mL) and triethylamine (8.13 mL, 58 mmol, 2.5 equiv.) was added. The solution was cooled to 0 °C and acetyl chloride (2.50 mL, 35 mmol, 1.5 equiv.) was added dropwise. The reaction mixture was allowed to warm to room temperature and stirred for an additional 30 min. Then it was quenched by the addition of a saturated solution of NH_4Cl (60 mL) and extracted with DCM (4x75 mL). The combined organic fractions were dried over anhydrous $MgSO_4$ and concentrated under reduced pressure. The residue was purified by column chromatography on silica gel (*n*-hexane:DCM:EtOAc 4:4:1) to give acetate **36** (6.32 g, 60% yield from 5-hydroxyindanone **8**) as a white crystalline solid.

R_f = 0.26 (*n*-hexane:EtOAc 4:1).

Mp: 152-153 °C (dichloromethane).

^{1}H NMR (400 MHz, 298 K, $CDCl_3$): δ = 7.74 (d, *J* = 8.1 Hz, 1H), 7.12 (d, *J* = 8.1 Hz, 1H), 3.02 (m, 2H), 2.75 (m, 2H), 2.41 (s, 3H).

^{13}C{^{1}H} NMR (101 MHz, 298 K, $CDCl_3$): δ = 205.4, 168.3, 161.3, 156.5, 136.5, 124.7, 122.9, 93.1, 36.9, 31.4, 21.4.

HRMS (ESI) *m/z*: ($[M+H]^+$) calcd for $C_{11}H_{10}O_3I$ 316.9669, found 316.9669 (Δ = -0.13 ppm).

IR ($CHCl_3$): 3027 (w), 2926 (w), 1774 (m), 1717 (s), 1592 (w), 1574 (w), 1454 (w), 1436 (w), 1370 (m), 1320 (m), 1280 (w), 1248 (m), 1188 (vs), 1156 (w), 1038 (m), 1015 (m), 992 (vw), 952 (w), 814 (w) cm^{-1}.

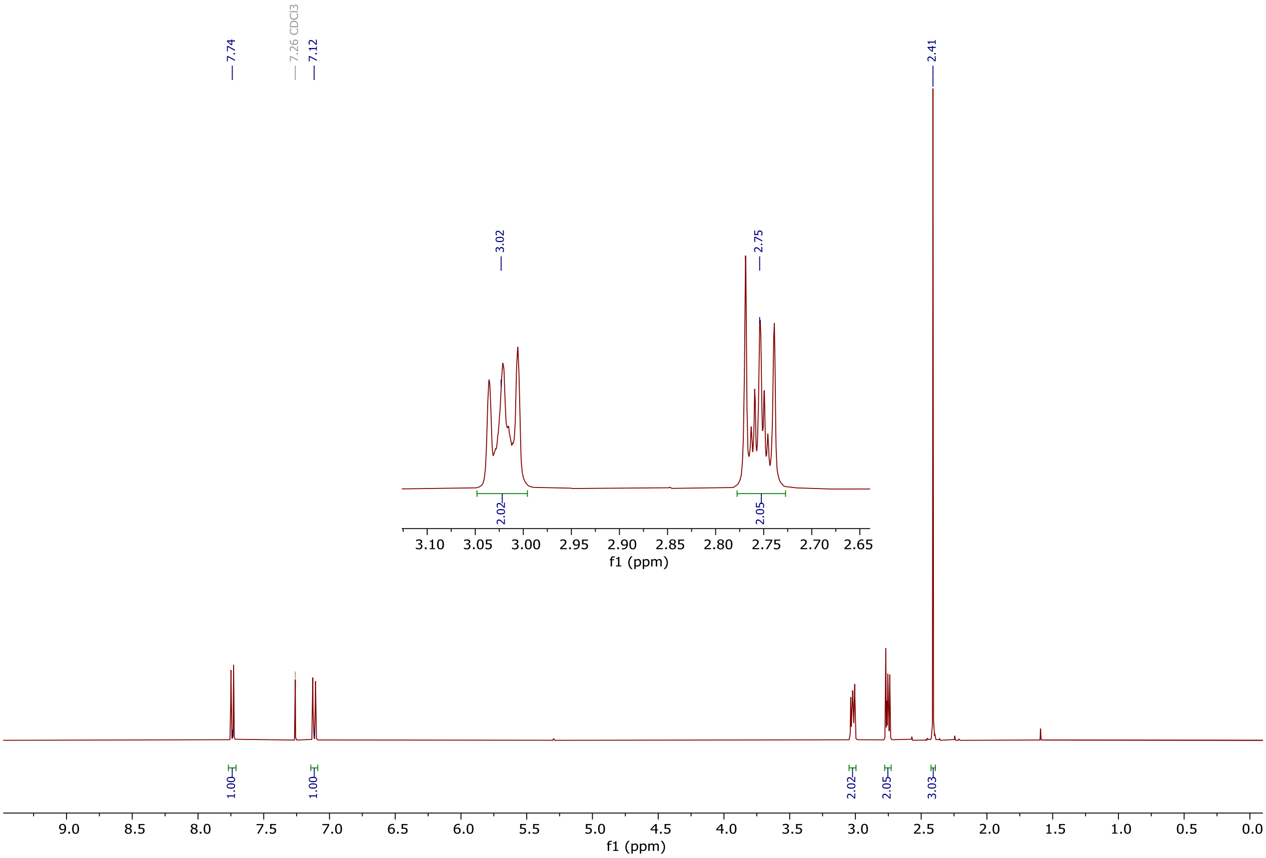

Figure S1: **^{1}H NMR of 36** (400 MHz, 298 K, $CDCl_3$).

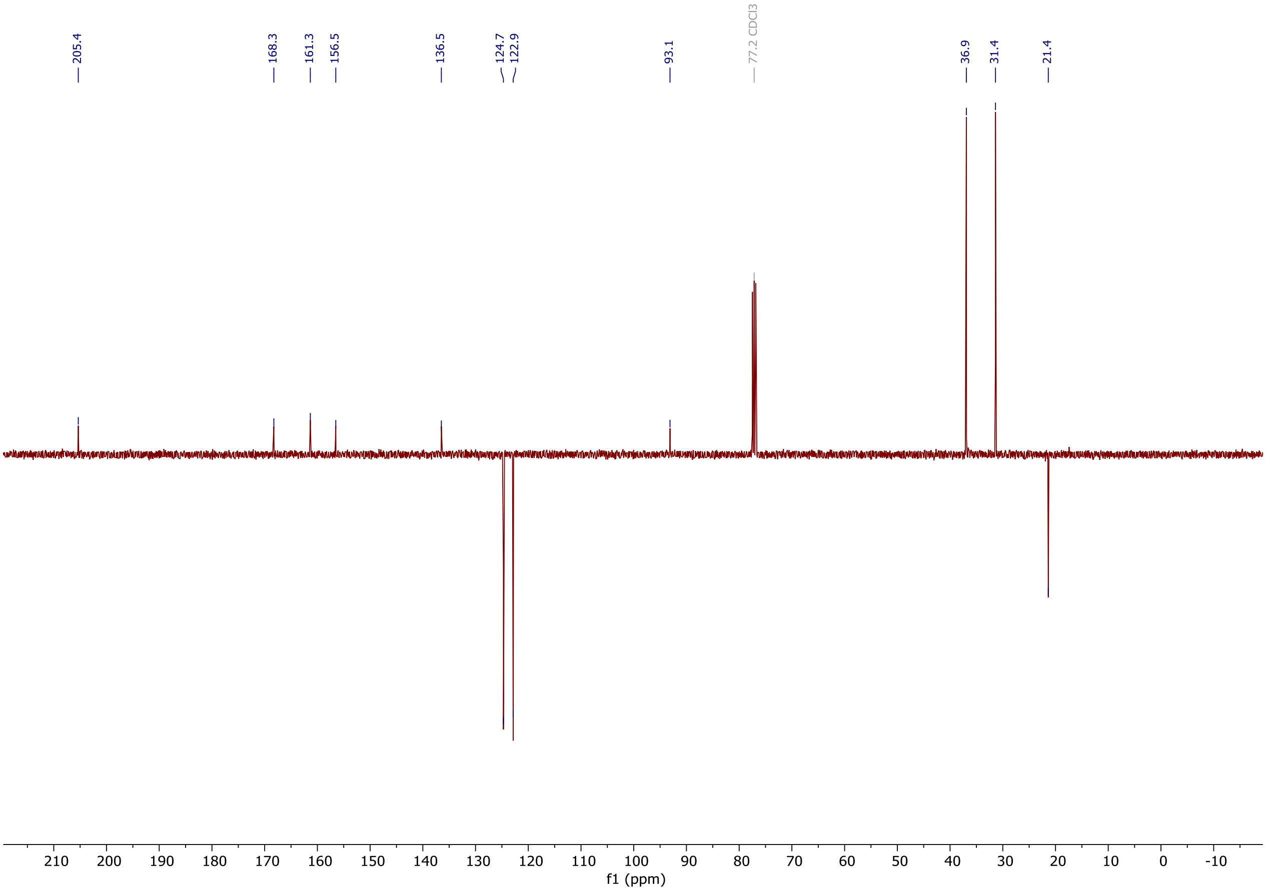

Figure S2: ^{13}C APT NMR of 36 (101 MHz, 298 K, $CDCl_3$).

1-Oxo-4-((triisopropylsilyl)ethynyl)-2,3-dihydro-1*H*-inden-5-yl acetate 37

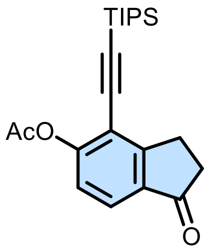

A solution of 4-iodo-1-oxo-2,3-dihydro-1H-inden-5-yl **36** (5.00 g, 16 mmol, 1.0 equiv.), $Pd(PPh_3)_2Cl_2$ (555 mg, 0.8 mmol, 5 mol%), and CuI (301 mg, 1.6 mmol, 10 mol%) in a mixture of dry triethylamine (20 mL) and dry toluene (40 mL) was degassed by three freeze-pump-thaw cycles. The solution of the reactants was inserted into a preheated oil bath set to 40 °C. After 5 min, (triisopropylsilyl)acetylene (10.6 mL, 47 mmol, 3.0 equiv.) was added dropwise and the reaction was allowed to stir at this temperature for 3 h. A color change from red to black was observed. After 3 h, the reaction mixture was diluted with EtOAc (100 mL) and extracted with water (2x50 mL) and brine (50 mL), dried over anhydrous $MgSO_4$, and volatiles were evaporated under reduced pressure. The residue was purified by column chromatography on silica gel (cyclohexane:EtOAc 1:0 to 4:1) to give alkyne **37** (5.179 g, 88%) as an off-white amorphous solid.

$\mathbf{R_f}$ = 0.50 (*n*-hexane:EtOAc 4:1).

^{1}H NMR (400 MHz, 298 K, $CDCl_3$): δ = 7.71 (d, *J* = 8.3 Hz, 1H), 7.12 (dt, *J* = 8.2, 0.8 Hz, 1H), 3.19 (m, 2H), 2.74 (m, 2H), 2.35 (s, 3H), 1.14 (m, 21H).

^{13}C{^{1}H} NMR (101 MHz, 298 K, $CDCl_3$): δ = 205.4, 168.3, 159.9, 156.6, 135.1, 124.5, 122.3, 116.7, 102.3, 98.1, 36.5, 25.9, 21.0, 18.8 (6C), 11.3 (3C).

HRMS (ESI) *m/z*: ([M+H]$^+$) calcd for $C_{22}H_{31}O_3Si$ 371.2037, found 371.2035 (Δ = -0.48 ppm).

IR ($CHCl_3$): 3026 (w), 2959 (m), 2945 (s), 2928 (w), 2893 (s), 2867 (s), 2157 (w), 1771 (s), 1710 (vs), 1603 (w), 1583 (m), 1466 (m), 1444 (w), 1404 (w), 1383 (w), 1370 (m), 1325 (w), 1285 (m), 1242 (m), 1189 (vs), 1149 (w), 1070 (w), 1055 (m), 1018 (m), 997 (w), 954 (vw), 884 (m), 817 (w), 679 (m), 615 (w) cm^{-1}.

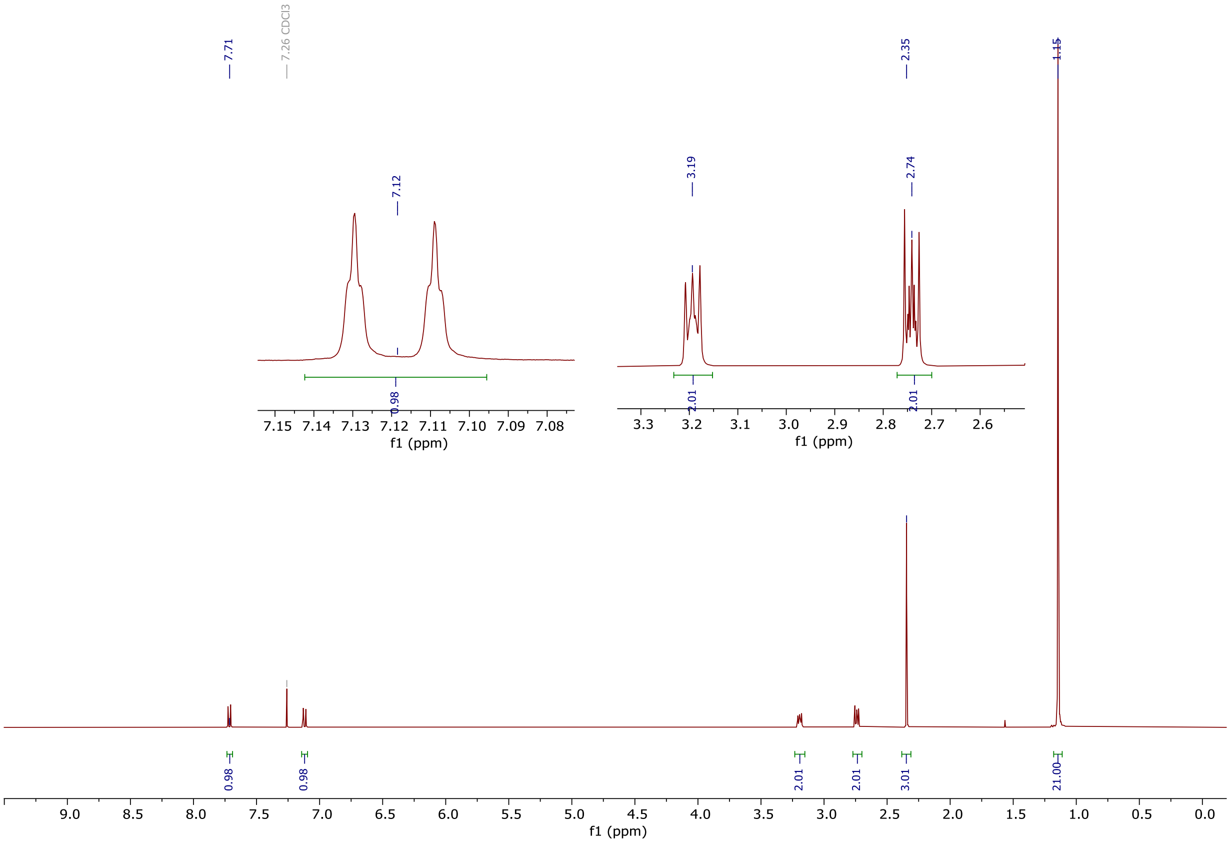

Figure S3: ¹H NMR of 37 (400 MHz, 298 K, $CDCl_3$).

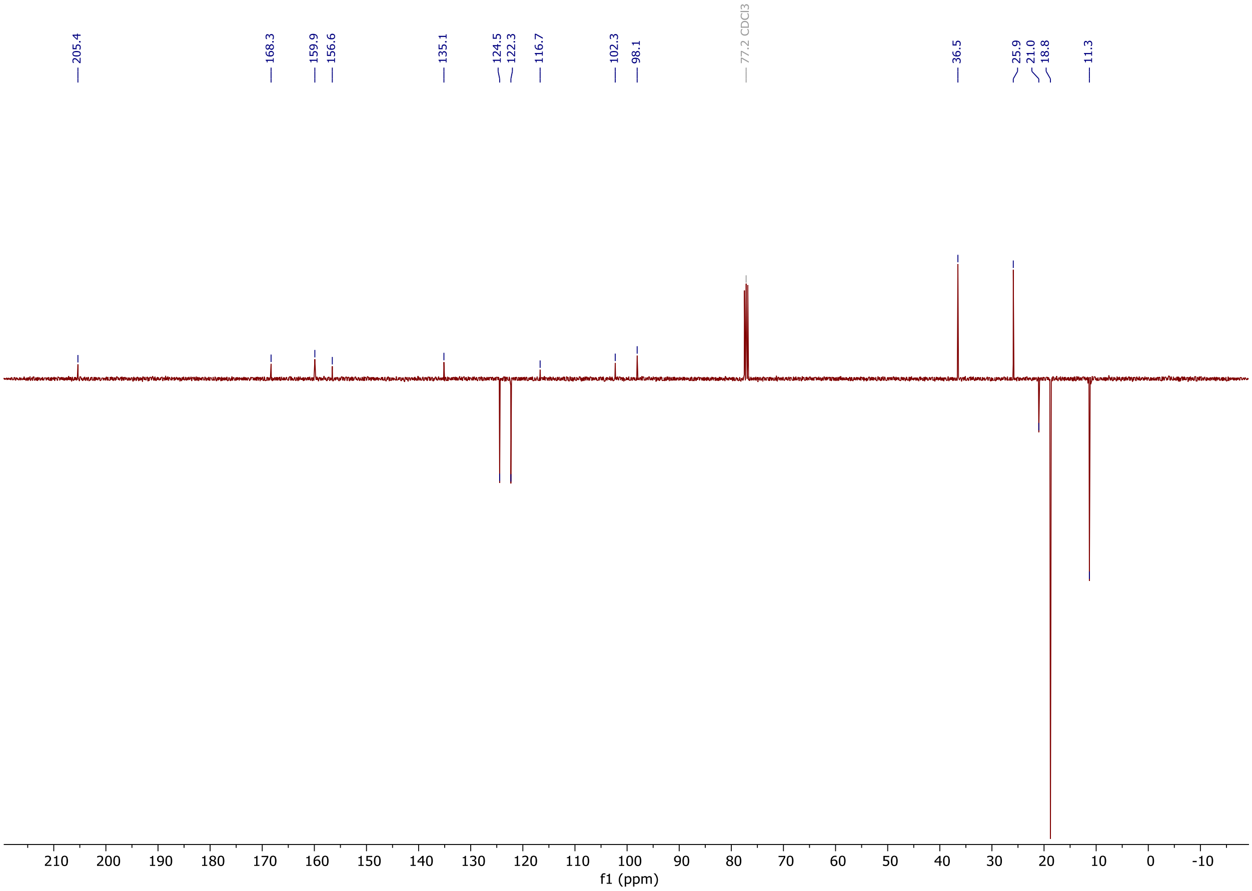

Figure S4: ¹³C APT NMR of 37 (101 MHz, 298 K, $CDCl_3$).

5-Hydroxy-4-((triisopropylsilyl)ethynyl)-2,3-dihydro-1*H*-inden-1-one 9

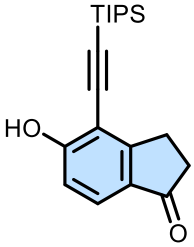

The acetyl-protected phenol **37** (8.48 g, 23 mmol, 1.0 equiv.) was dissolved in methanol (190 mL). K_2CO_3 (6.325 g, 46 mmol, 2.0 equiv.) was added in one portion at room temperature and the resulting solution was allowed to stir for 20 min. The reaction mixture was diluted with water (100 mL), quenched by the addition of a saturated solution of NH_4Cl (50 mL), and extracted with ether (3x100 mL). The combined organic fractions were dried over anhydrous $MgSO_4$, and the volatiles were removed under reduced pressure to give **9** (7.320 g, 97%) as an off-white crystalline solid. The product was used as received without further purification.

$\mathbf{R_f}$ = 0.24 (*n*-hexane:EtOAc 4:1).

Mp: 146-147 °C (*n*-hexane:EtOAc).

^{1}**H NMR** (400 MHz, 298 K, $CDCl_3$): δ = 7.66 (dd, *J* = 8.4, 1.6 Hz, 1H), 6.98 (dd, *J* = 8.4, 1.6 Hz, 1H), 6.32 (bs, 1H), 3.13 (m, 2H), 2.70 (m, 2H), 1.16 (s, 21H).

^{13}**C{^{1}H} NMR** (101 MHz, 298 K, $CDCl_3$): δ = 204.8, 162.6, 159.6, 130.6, 126.0, 115.1, 108.0, 104.2, 97.3, 36.5, 25.7, 18.8 (6C), 11.2 (3C).

HRMS (ESI) *m/z*: ($[M+H]^+$) calcd for $C_{20}H_{29}O_2Si$ 329.1931, found 329.1927 (Δ = -1.18 ppm).

IR ($CHCl_3$): 3499 (m), 2959 (s), 2956 (s), 2929 (s), 2867 (s), 2143 (w), 1701 (vs), 1609 (w), 1586 (vs), 1472 (m), 1463 (m), 1406 (vw), 1384 (w), 1367 (w), 1274 (s), 1243 (s), 1071 (w), 997 (m), 883 (m), 818 (m), 679 (m), 664 (m), 547 (w) cm^{-1}.

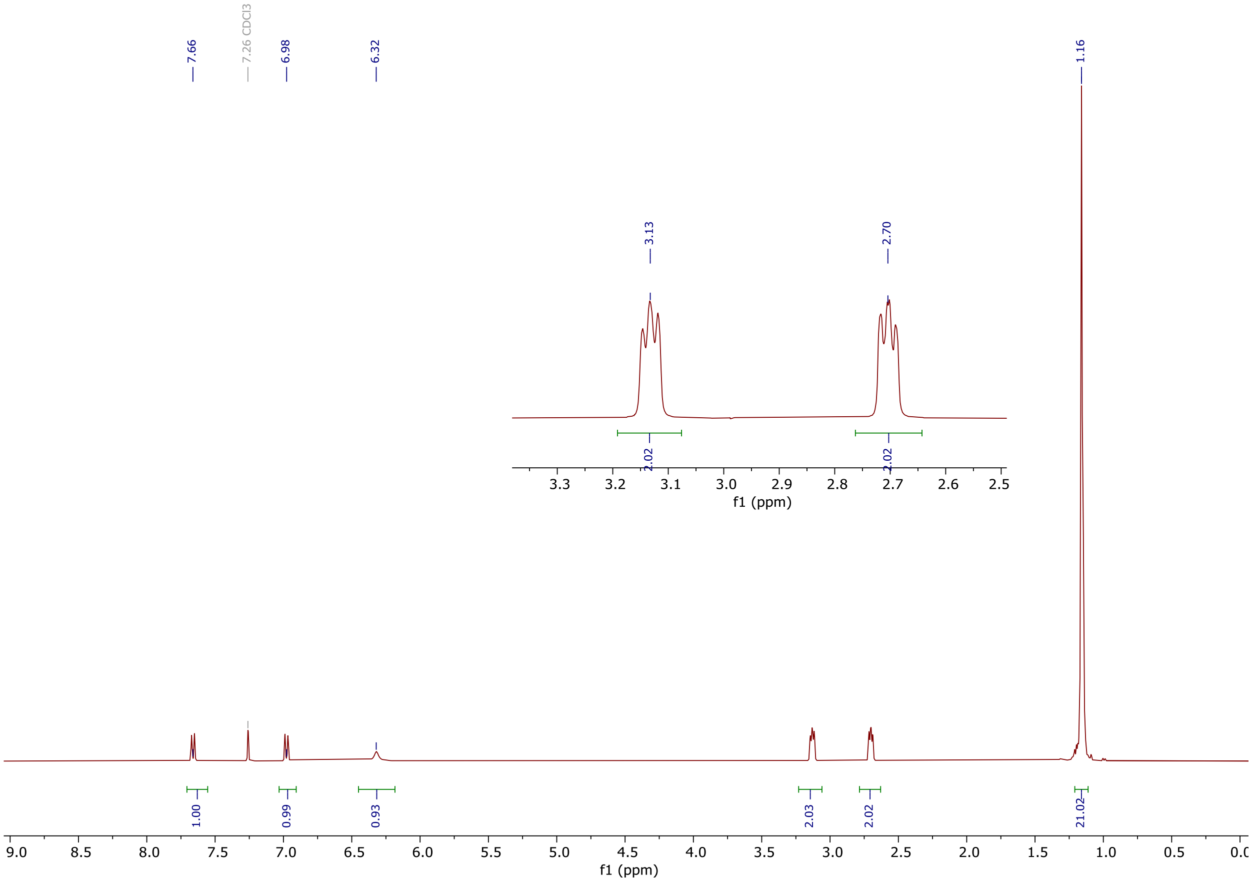

Figure S5: ^{1}H NMR of 9 (400 MHz, 298 K, $CDCl_3$).

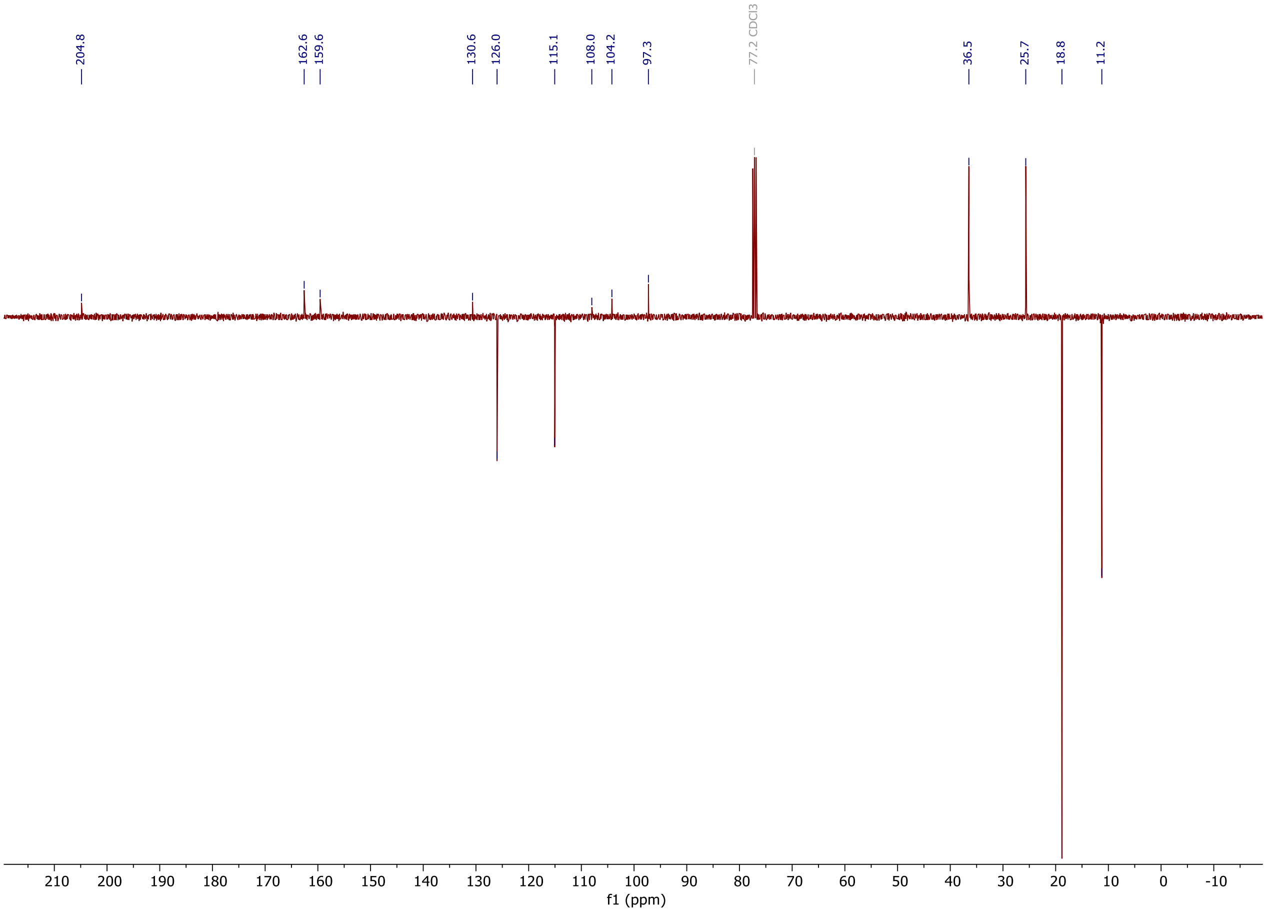

Figure S6: ^{13}C APT NMR of 9 (101 MHz, 298 K, $CDCl_3$).

(+)-(*S*)-5-((4-(*p*-Tolyl)but-3-yn-2-yl)oxy)-4-((triisopropylsilyl)ethynyl)-2,3-dihydro-1*H*-inden-1-one (+)-(*S*)-**38**

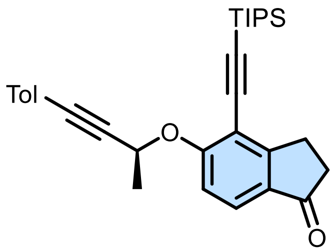

A solution of compound **9** (7.240 g, 22 mmol, 1.0 equiv.), (+)-(*R*)-**10** (4.237 g, 26 mmol, 1.2 equiv.), and PPh_3 (6.358 g, 24 mmol, 1.1 equiv.) in of benzene (150 mL) under inert atmosphere was inserted into an ice/water bath. Diisopropyl azodicarboxylate (5.2 mL, 5.35 g, 26 mmol, 1.2 equiv.) was added dropwise and the resulting solution was stirred for 10 min and then warmed to ambient temperature and stirred for additional 2 h. The solvent was evaporated and the residue was subjected to column chromatography on silica gel (*n*-hexane:EtOAc 8:1) to give (+)-(*S*)-**38** (9.550 g, 92%) as a light yellow amorphous solid.

$\mathbf{R_f}$ = 0.47 (*n*-hexane:EtOAc 4:1).

Mp: 95 °C (dichloromethane).

$[\alpha]^{20}_D$ +104.4 (*c* 0.293, THF).

^{1}H NMR (400 MHz, 298 K, $CDCl_3$): δ = 7.70 (d, *J* = 8.6 Hz, 1H), 7.27 (m, 2H, overlapping the signal of $CDCl_3$), 7.23 (d, *J* = 8.6 Hz, 1H), 7.09 (m, 2H), 5.21 (q, *J* = 6.5 Hz, 1H), 3.15 (m, 2H), 2.69 (m, 2H), 2.33 (s, 3H), 1.79 (d, *J* = 6.5 Hz, 3H), 1.16 (s, 21H).

^{13}C{^{1}H} NMR (101 MHz, 298 K, $CDCl_3$): δ = 205.5, 163.9, 160.3, 139.1, 131.8 (2C), 130.8, 129.2 (2C), 124.8, 119.1, 113.8, 111.8, 100.7, 99.3, 87.0, 86.7, 66.0, 36.5, 25.8, 22.5, 21.6, 18.9 (6C), 11.4 (3C).

HRMS (ESI) *m/z*: ($[M+H]^+$) calcd for $C_{31}H_{39}O_2Si$ 471.2714, found 471.2712 (Δ = -0.36 ppm).

IR ($CHCl_3$): 3031 (w), 2959 (m), 2944 (s), 2926 (m), 2892 (m), 2866 (m), 2240 (w), 2227 (w), 2159 (w), 2144 (w), 1699 (vs), 1583 (s), 1510 (m), 1477 (m), 1464 (m), 1444 (w), 1407 (w), 1383 (w), 1376 (w) 1367 (w), 1336 (m), 1323 (m), 1282 (m), 1263 (s), 1243 (m), 1181 (w), 1163 (w), 1120 (w), 1085 (m), 1040 (w), 997 (w), 943 (w), 883 (m), 819 (m), 678 (w), 661 (w) cm^{-1}.

(-)-(*R*)-5-((4-(*p*-Tolyl)but-3-yn-2-yl)oxy)-4-((triisopropylsilyl)ethynyl)-2,3-dihydro-1*H*-inden-1-one (-)-(*R*)-**38**

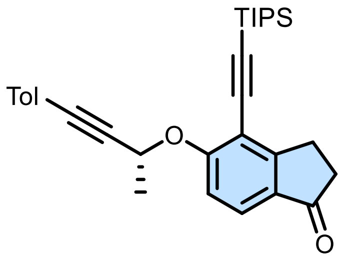

Prepared similarly to (+)-(*S*)-**38** staring from **9** (5.210 g, 16 mmol, 1.0 equiv.), (-)-(*S*)-**10** (2.795 g, 17 mmol, 1.1 equiv.), PPh_3 (4.576 g, 17 mmol, 1.1 equiv.), and diisopropyl azodicarboxylate (3.7 mL, 3.85 g, 19 mmol, 1.2 equiv.). Yielded (-)-(*R*)-**38 (**6.980 g, 94%) as a white amorphous solid.

NMR spectra in accordance with (+)-(*S*)-**38**.

$[\alpha]^{20}_D$ -103.4 (*c* 0.229, THF).

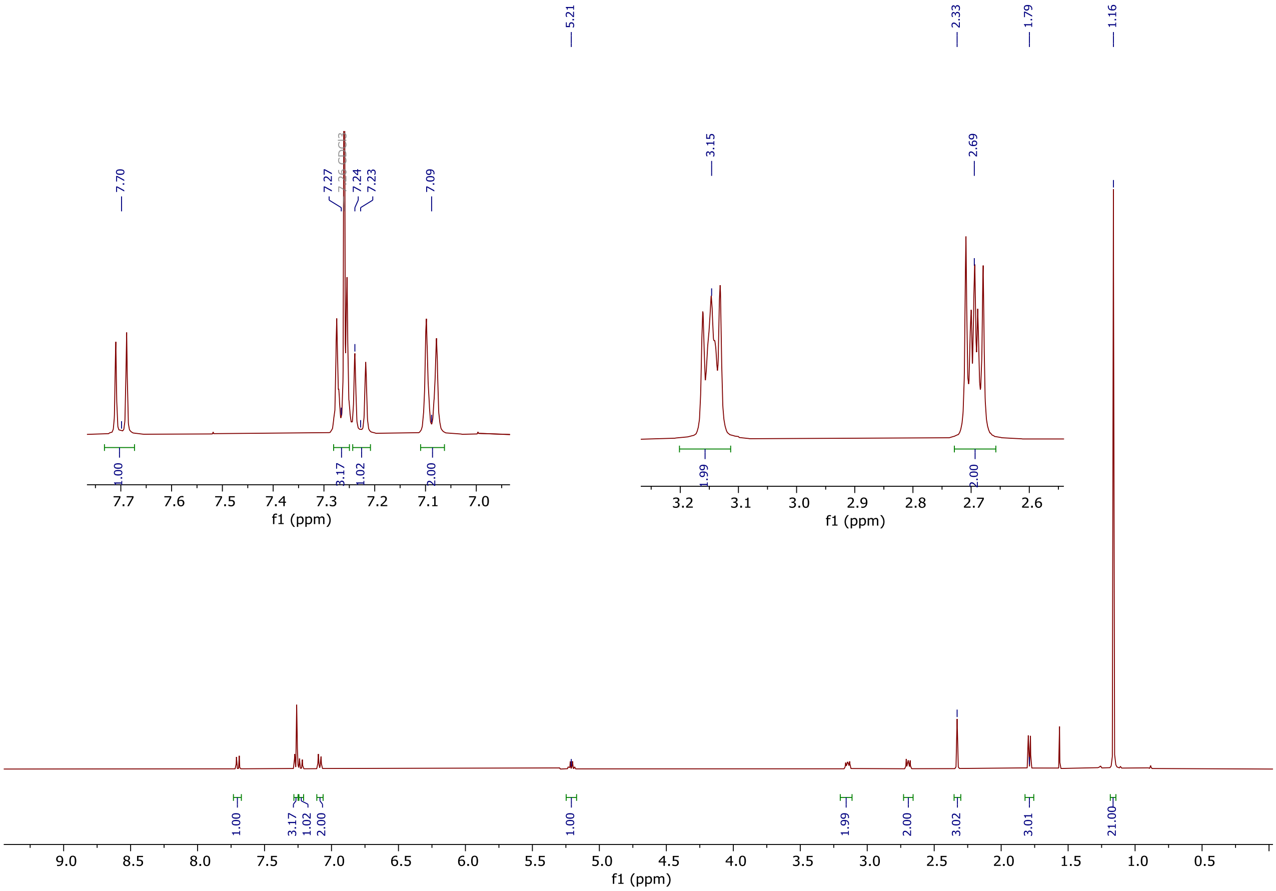

Figure S7: ^{1}H NMR of (+)-(*S*)-**38** (400 MHz, 298 K, $CDCl_3$).

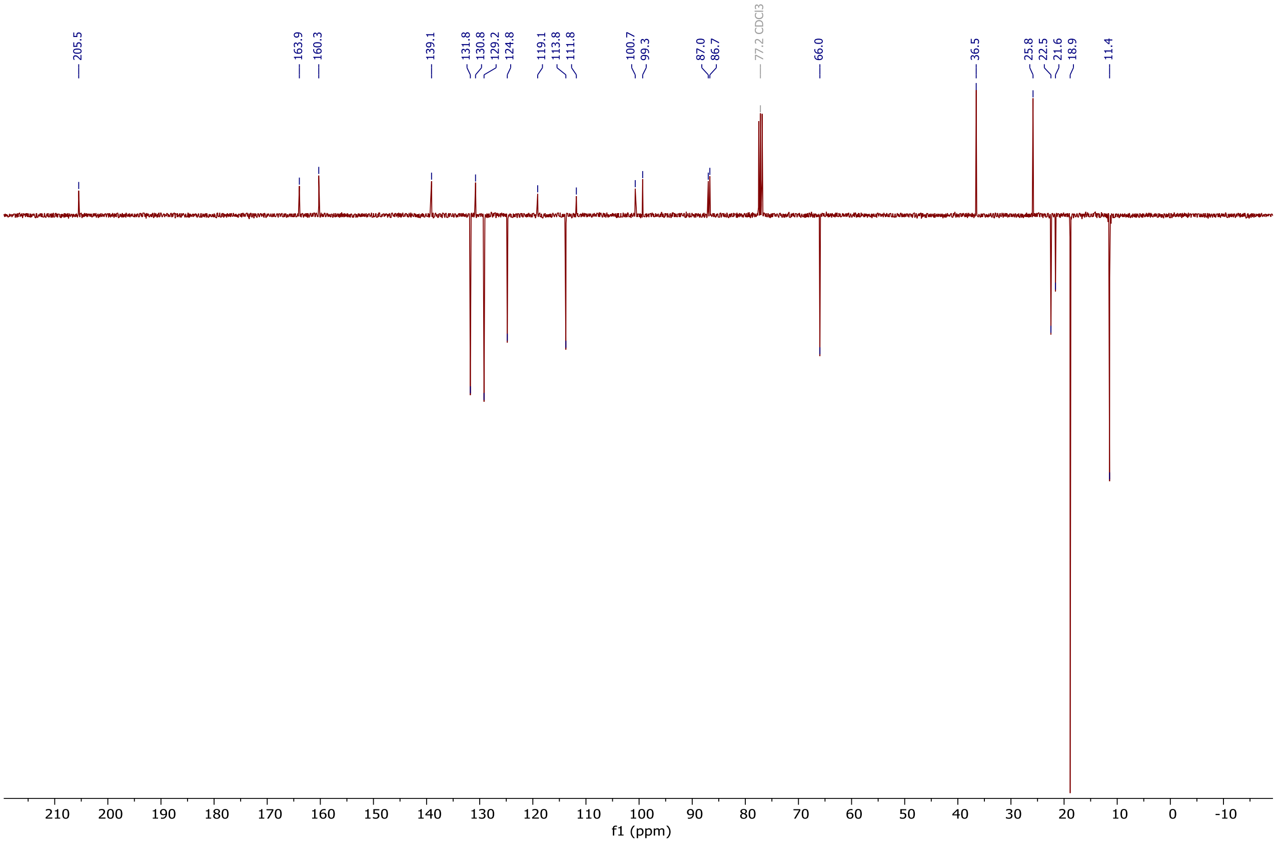

Figure S8: ^{13}C APT NMR of (+)-(*S*)-**38** (101 MHz, 298 K, $CDCl_3$).

(+)-(*S*)-4-Ethynyl-5-((4-(*p*-tolyl)but-3-yn-2-yl)oxy)-2,3-dihydro-1*H*-inden-1-one (+)-(*S*)-**11**

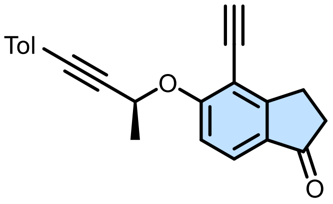

The silylated diyne (+)-(*S*)-**38** (5.830 g, 12 mmol, 1.0 equiv.) was dissolved in dry THF (100 mL) and cooled to 0 °C. Tetra-*n*-butylammonium fluoride trihydrate (3.908 g, 12 mmol, 1.0 equiv.) was weighed separately and dissolved in dry THF (35 mL). The solution of TBAF was then added dropwise to the stirred solution of diyne (+)-(*S*)-**38**. A color change from colorless to dark red was observed. The reaction was quenched 5 min after the addition was completed by the addition of MeOH (50 mL). The volatiles were removed under reduced pressure and the residue (dark red oil) was purified by column chromatography on silica gel (cyclohexane:EtOAc 1:0 to 4:1) to give (+)-(*S*)-**11** (3.607 g, 93%) as a light yellow crystalline solid.

R_f = 0.31 (*n*-hexane:EtOAc 4:1).

Mp: 117-118 °C (dichloromethane).

$[\alpha]^{20}_D$ +5.1 (*c* 0.273, THF).

^{1}H NMR (400 MHz, 298 K, $CDCl_3$): δ = 7.74 (d, *J* = 8.6 Hz, 1H), 7.28 – 7.22 (m, 3H, overlapping with the NMR solvent signal), 7.08 (m, 2H), 5.22 (q, *J* = 6.5 Hz, 1H), 3.53 (s, 1H), 3.15 (m, 2H), 2.69 (m, 2H), 2.33 (s, 3H), 1.83 (d, *J* = 6.5 Hz, 3H).

$^{13}C\{^1H\}$ NMR (101 MHz, 298 K, $CDCl_3$): δ = 205.2, 164.0, 160.6, 139.1, 131.7 (2C), 130.8, 129.2 (2C), 125.4, 119.0, 113.6, 110.2, 87.1, 86.4, 86.2, 76.5, 66.1, 36.4, 25.7, 22.4, 21.6.

HRMS (ESI) *m/z*: ($[M+H]^+$) calcd for $C_{22}H_{19}O_2$ 315.1380, found 315.1377 (Δ = -0.86 ppm).

IR ($CHCl_3$): 3304 (w), 3029 (w), 2927 (w), 2856 (w), 2235 (w), 2228 (w), 2107 (vw), 1701 (s), 1585 (s), 1511 (m), 1478 (m), 1443 (w), 1407 (vw), 1377 (w), 1333 (m-w), 1323 (m), 1281 (m), 1260 (vs), 1243 (m-w), 1181 (vw), 1165 (w), 1106 (vw), 1085 (m), 1040 (w), 1020 (w), 947 (vw), 819 (m), 656 (w), 620 (w) cm^{-1}.

(-)-(*R*)-4-Ethynyl-5-((4-(*p*-tolyl)but-3-yn-2-yl)oxy)-2,3-dihydro-1*H*-inden-1-one (-)-(*R*)-**11**

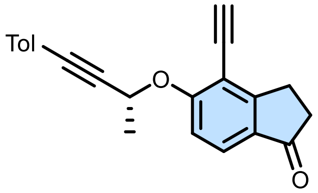

Prepared similarly to (+)-(*S*)-**11** staring from (-)-(*R*)-**38** (7.00 g, 15 mmol, 1.0 equiv.) and TBAF·$3H_2O$ (4.692 g, 15 mmol, 1.0 equiv.). Yielded (-)-(*R*)-**11** (4.195 g, 90%) as a light yellow crystalline solid.

NMR spectra in accordance with (+)-(*S*)-**11**.

$[\alpha]^{20}_D$ -8.6 (*c* 0.243, THF).

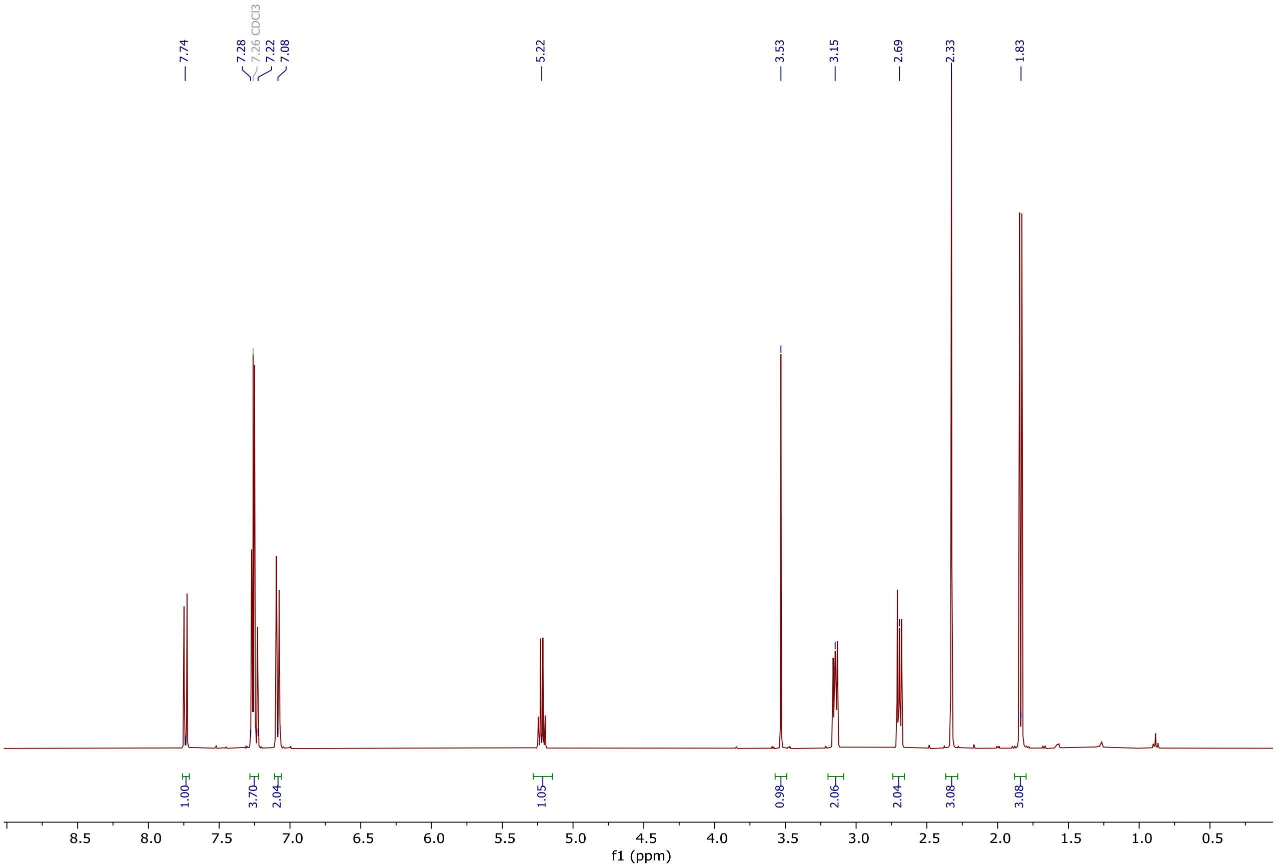

Figure S9: ¹H NMR of (+)-(*S*)-**11** (400 MHz, 298 K, $CDCl_3$).

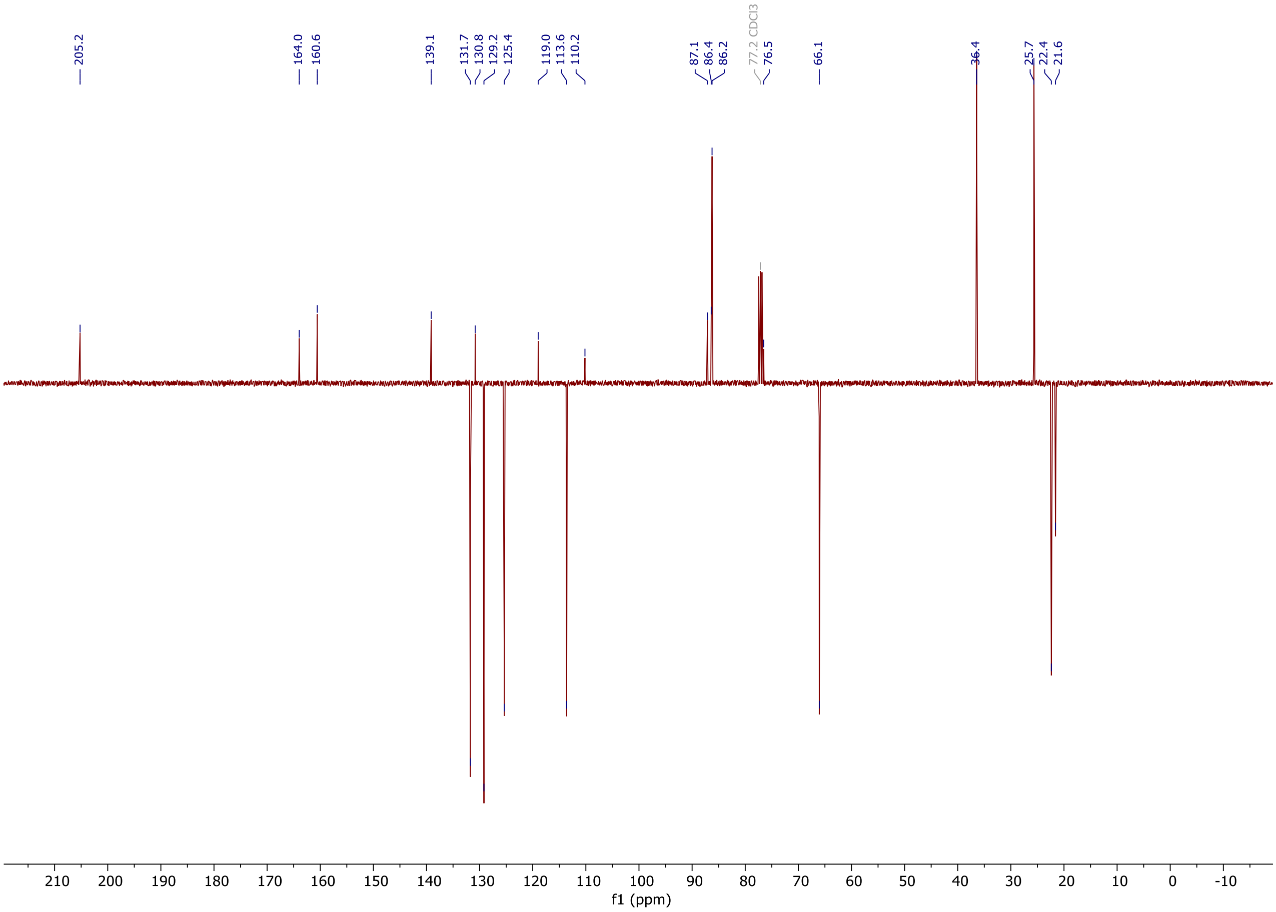

Figure S10: ¹³C APT NMR of (+)-(*S*)-**11** (101 MHz, 298 K, $CDCl_3$).

(+)-(*S*)-4-((2-Hydroxyphenyl)ethynyl)-5-((4-(*p*-tolyl)but-3-yn-2-yl)oxy)-2,3-dihydro-1*H*-inden-1-one (+)-(*S*)-**39**

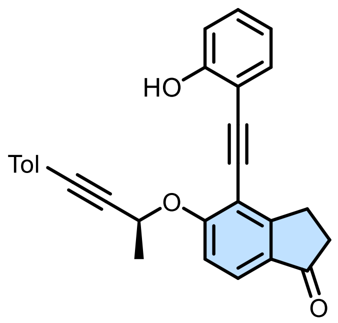

A dried Schlenk was charged with 2-iodophenol (2.099 g, 10 mmol, 3.0 equiv.), $Pd(PPh_3)_4$ (184 mg, 0.16 mmol, 5 mol%) and CuI (30 mg, 0.16 mmol, 5 mol%) were added and dissolved in toluene (30 mL) and triethylamine (0.55 mL, 4 mmol, 1.25 equiv.) was added. In a separate Schlenk flask, a solution of diyne (+)-(*S*)-**11** (1.000 g, 3 mmol, 1.0 equiv.) in toluene (10 mL) was prepared. Both solutions were degassed by three freeze-pump-thaw cycles. The flask with the reaction mixture was then immersed into a preheated oil bath set to 50 °C and the solution of diyne (+)-(*S*)-**11** was added dropwise within 20 min. The reaction mixture was allowed to stir at 50 °C for 48 h after which some unreacted diyne remained in the reaction mixture. The volatiles were evaporated under reduced pressure. The residue was purified by column chromatography on silica gel (cyclohexane:EtOAc 6:1) to give (+)-(*S*)-**39** (900.5 mg, 70%) as an orange crystalline solid.

R_f = 0.13 (*n*-hexane:EtOAc 4:1).

Mp: 155-156 °C (*n*-hexane).

$[\alpha]^{20}_D$ +248.5 (*c* 0.268, THF).

^{1}H NMR (400 MHz, 298 K, $CDCl_3$): δ = 7.75 (d, *J* = 8.6 Hz, 1H), 7.42 (dd, *J* = 7.7, 1.6 Hz, 1H), 7.32 – 7.26 (m, 4H, overlapping NMR solvent signal), 7.09 (m, 2H), 7.02 (dd, *J* = 8.3, 1.1 Hz, 1H), 6.92 (td, *J* = 7.5, 1.1 Hz, 1H), 6.53 (s, 1H), 5.29 (q, *J* = 6.5 Hz, 1H), 3.22 (m, 2H), 2.73 (m, 2H), 2.32 (s, 3H), 1.90 (d, *J* = 6.5 Hz, 3H).

^{13}C{^{1}H} NMR (101 MHz, 298 K, $CDCl_3$): δ = 205.1, 162.7, 158.4, 157.3, 139.2, 131.8 (2C), 131.0, 130.9, 130.6, 129.2 (2C), 125.1, 120.3, 118.8, 114.8, 113.0, 110.6, 109.7, 93.0, 90.1, 87.5, 86.1, 66.2, 36.4, 25.6, 22.4, 21.6.

HRMS (ESI) *m/z*: ($[M+H]^+$) calcd for $C_{28}H_{23}O_3$ 407.1642, found 407.1638 (Δ = -1.02 ppm).

IR ($CHCl_3$): 3523 (w), 3468 (w), 3086 (vw), 3030 (w), 2974 (m-w), 2855 (w), 2239 (w), 2226 (w), 2202 (vw), 1702 (vs-s), 1615 (w), 1584 (s-m), 1510 (m-w) 1490 (w), 1473 (m), 1463 (w), 1445 (w), 1378 (w), 1329 (w), 1293 (w), 1277 (m), 1253 (vs), 1237 (w), 1181 (vw), 1120 (w), 1169 (w), 1097 (w), 1084 (m), 1039 (w), 1021 (w), 938 (w), 831 (w), 819 (m-w), 567 (w) cm^{-1}.

(-)-(*R*)-4-((2-Hydroxyphenyl)ethynyl)-5-((4-(*p*-tolyl)but-3-yn-2-yl)oxy)-2,3-dihydro-1*H*-inden-1-one (-)-(*R*)-**39**

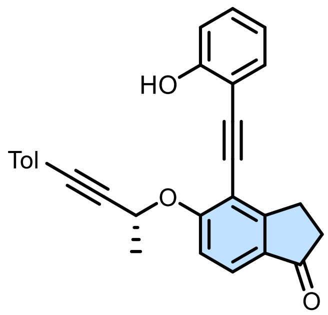

Prepared similarly to (+)-(*S*)-**39** staring from (-)-(*R*)-**11** (1.000 g, 3 mmol, 1.0 equiv.), 2-iodophenol (2.099 g, 10 mmol, 3.0 equiv.), $Pd(PPh_3)_4$ (184 mg, 0.16 mmol, 5 mol%), CuI (30 mg, 0.16 mmol, 5 mol%), and triethylamine (0.55 mL, 4 mmol, 1.25 equiv.). Yielded (-)-(*R*)-**39** (0.892 g, 69%) as a light brown amorphous solid.

NMR spectra in accordance with (+)-(*S*)-**39**.

$[\alpha]^{20}_D$ -239.9 (*c* 0.258, THF).

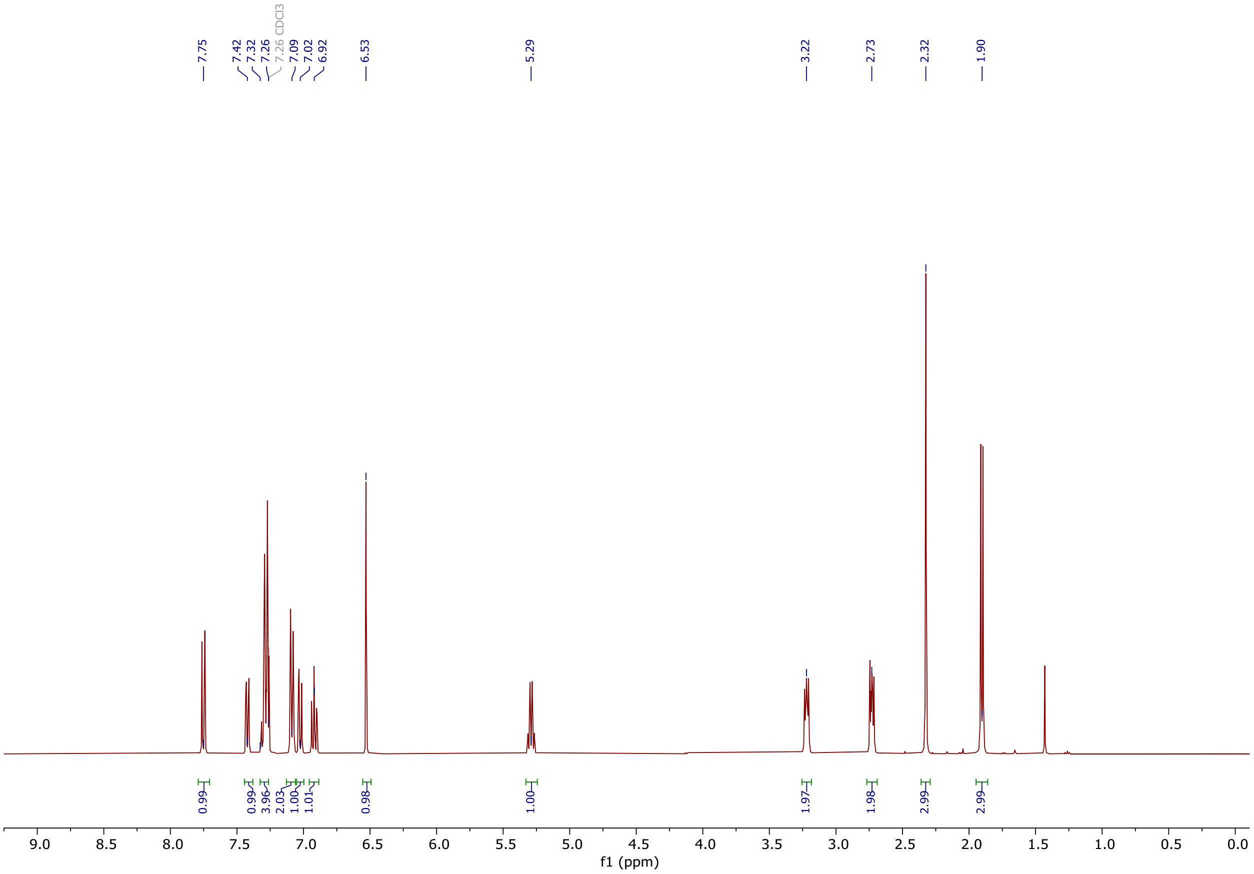

Figure S11: ¹H NMR of (+)-(*S*)-**39** (400 MHz, 298 K, $CDCl_3$).

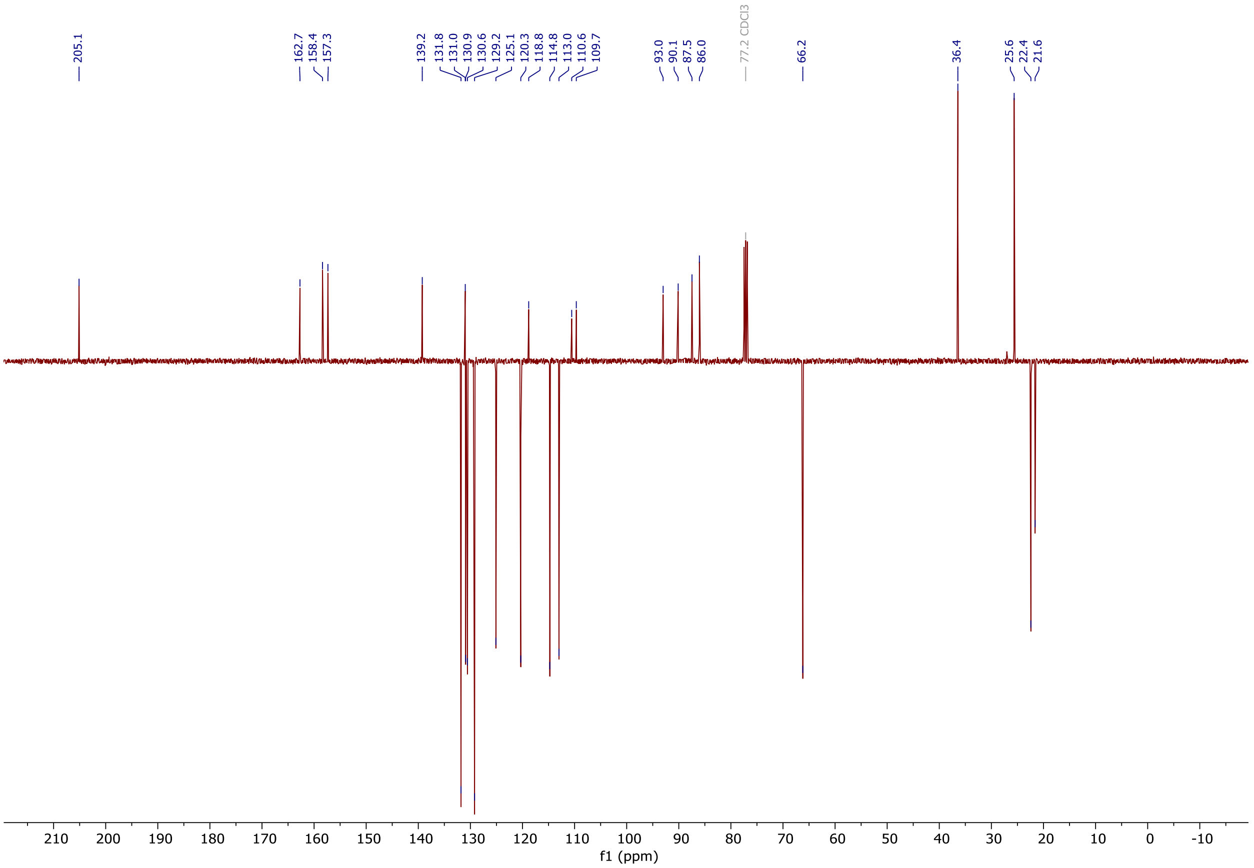

Figure S12: ¹³C APT NMR of (+)-(*S*)-**39** (101 MHz, 298 K, $CDCl_3$).

(+)-5-(((*S*)-4-(*p*-Tolyl)but-3-yn-2-yl)oxy)-4-((2-(((*S*)-4-(*p*-tolyl)but-3-yn-2-yl)oxy)phenyl)ethynyl)-2,3-dihydro-1*H*-inden-1-one (+)-(*S,S*)-**12**

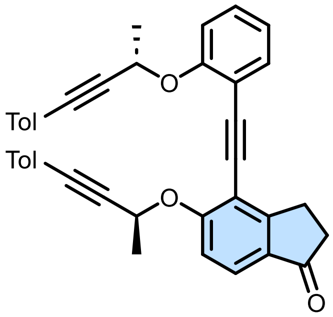

A solution of phenol (+)-(*S*)-**39** (1.653 g, 4.1 mmol), (+)-(*R*)-**10** (782 mg, 4.9 mmol, 1.2 equiv.), and PPh_3 (1.173 g, 4.5 mmol, 1.1 equiv.) in benzene (60 mL) under inert atmosphere was inserted into an ice/water bath. Diisopropyl azodicarboxylate (0.96 mL, 0.987 g, 4.9 mmol, 1.2 equiv.) was added dropwise and the resulting solution was stirred for 10 min and then warmed to ambient temperature and stirred for additional 2 h. The volatiles were removed under reduced pressure and the residues were purified by column chromatography on silica gel (cyclohexane:EtOAc 8:1 to 4:1) and gave the yellow foam (2.008 g) which was recrystallized from heptane/EtOAc to obtain (+)-(*S,S*)-**12** (1.880 g, 84%) as white crystals.

R_f = 0.28 (*n*-hexane:EtOAc 4:1).

Mp: 56-57 °C (hexane/EtOAc 4:1).

$[\alpha]^{20}_D$ +339.1 (*c* 0.388, $CHCl_3$).

^{1}H NMR (400 MHz, 298 K, $CDCl_3$): δ = 7.71 (d, *J* = 8.6 Hz, 1H), 7.55 (m, 1H), 7.33 (ddd, *J* = 8.3, 7.4, 1.7 Hz, 1H), 7.29 – 7.20 (m, overlapping with the solvent signal, 6H), 7.10 – 7.05 (m, 4H), 7.00 (td, *J* = 7.5, 1.1 Hz, 1H), 5.29 (q, *J* = 6.5 Hz, 1H), 5.22 (q, *J* = 6.5 Hz, 1H), 3.35 – 3.20 (m, 2H), 2.68 (m, 2H), 2.32 (s, 6H), 1.87 (d, *J* = 6.5 Hz, 3H), 1.82 (d, *J* = 6.5 Hz, 3H).

^{13}C{^{1}H} NMR (101 MHz, 298 K, $CDCl_3$): δ = 205.7, 163.0, 160.2, 158.2, 139.0, 138.8, 133.7, 131.8 (2C), 131.7 (2C), 131.0, 129.9, 129.18 (2C), 129.16 (2C), 124.6, 121.5, 119.4, 119.1, 115.2, 114.24, 114.19, 112.2, 95.4, 87.6, 87.0, 86.8, 86.5, 86.4, 66.2, 65.8, 36.6, 25.9, 22.7, 22.5, 21.6 (2C)**.**

HRMS (ESI) *m/z*: ($[M+H]^+$) calcd for $C_{39}H_{33}O_3$ 549.2424, found 549.2419 (Δ = -0.99 ppm).

IR ($CHCl_3$): 3082 (w), 3031 (w), 2926 (m), 2855 (w), 2236 (w), 2226 (w), 2206 (w), 1699 (vs), 1582 (s), 1510 (s), 1495 (m), 1476 (w), 1447 (m), 1407 (w), 1375 (w), 1330 (m), 1277 (s-m), 1259 (m), 1249 (s), 1234 (m), 1180 (w), 1164 (w), 1120 (m), 1109 (m), 1086 (s), 1038 (m), 1020 (w), 947 (w), 819 (s) cm^{-1}.

(-)-5-(((*R*)-4-(*p*-Tolyl)but-3-yn-2-yl)oxy)-4-((2-(((*R*)-4-(*p*-tolyl)but-3-yn-2-yl)oxy)phenyl)ethynyl)-2,3-dihydro-1*H*-inden-1-one (-)-(*R,R*)-**12**

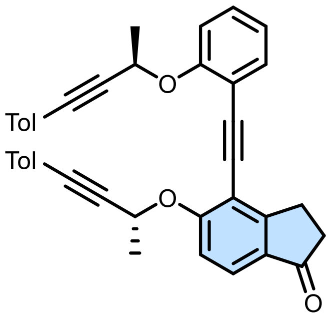

Prepared similarly to (+)-(*S,S*)-**12** staring from (-)-(*R*)-**39** (791 gm, 1.95 mmol), (-)-(*S*)-**10** (374 mg, 2.34 mmol, 1.2 equiv.), PPh_3 (561 mg, 2.14 mmol, 1.1 equiv.), and diisopropyl azodicarboxylate (0.46 mL, 470 mg, 2.34 mmol, 1.2 equiv.) in 30 mL of benzene. Yielded (-)-(*R,R*)-**12** (898 mg, 84%) as a light yellow crystalline solid.

NMR spectra in accordance with (+)-(*S,S*)-**12**.

$[\alpha]^{20}_D$ -335.7 (*c* 0.260, $CHCl_3$).

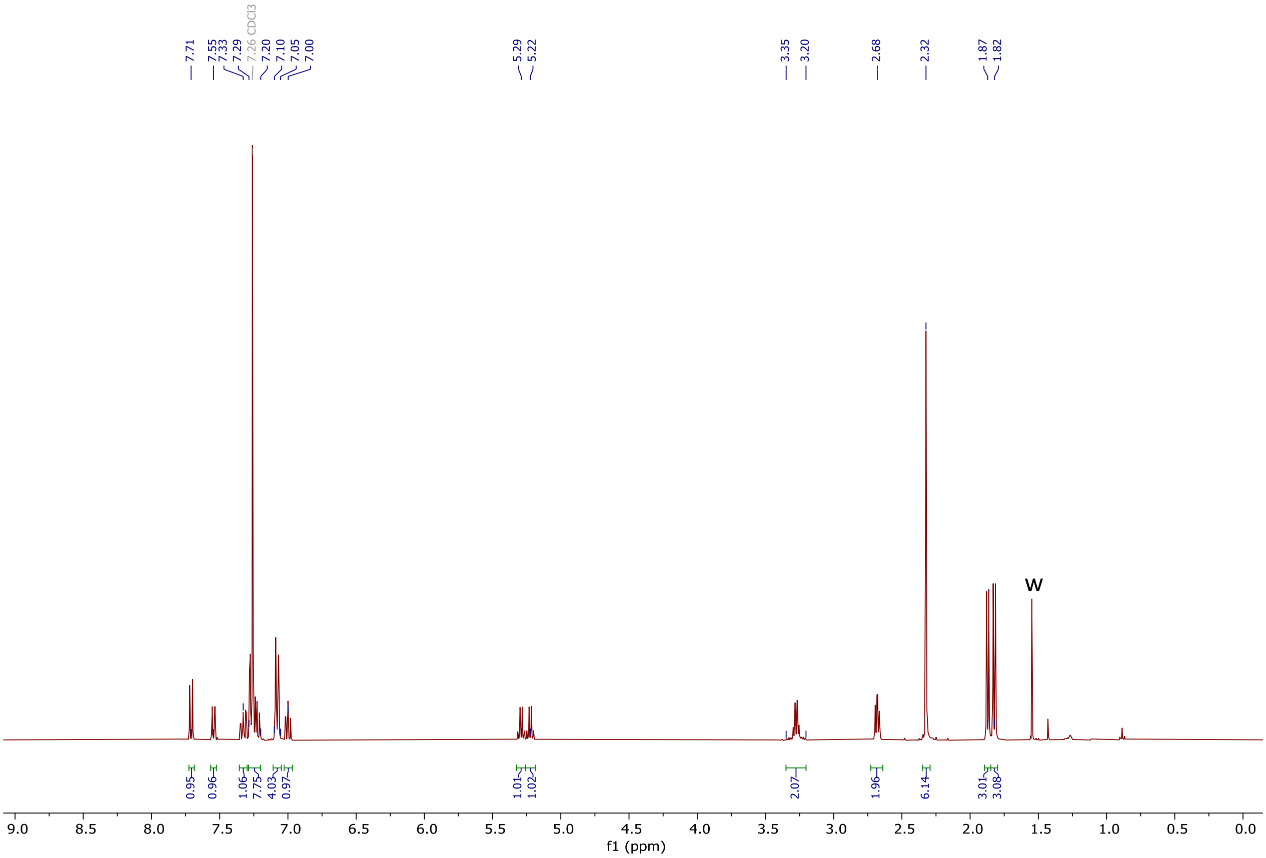

Figure S13: ^{1}H NMR of (+)-(*S*,*S*)-**12** (400 MHz, 298 K, $CDCl_3$) (w = water).

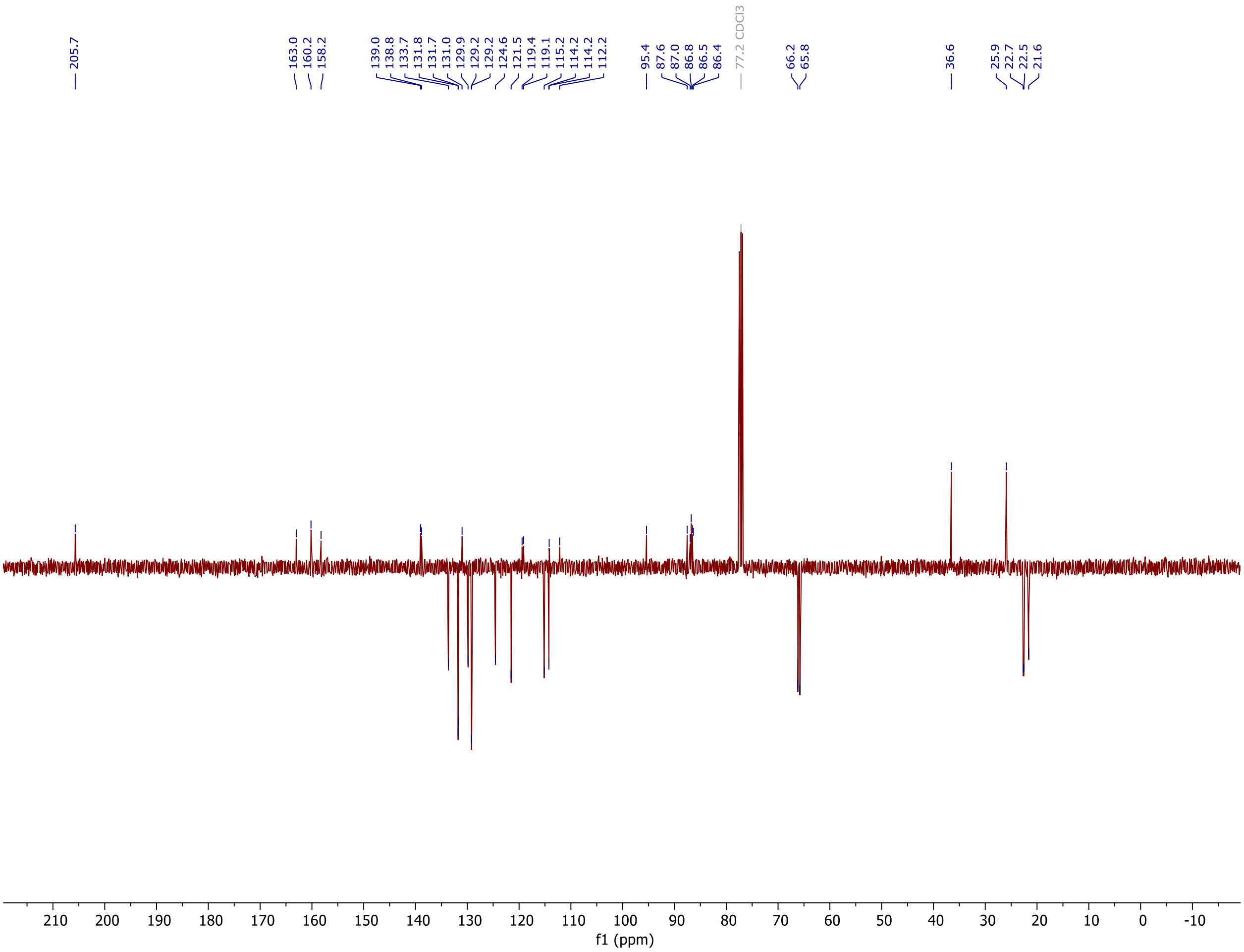

Figure S14: ^{13}C APT NMR of (+)-(*S*,*S*)-**12** (101 MHz, 298 K, $CDCl_3$).

Compound (+)-(*P,S,S*)-**13**

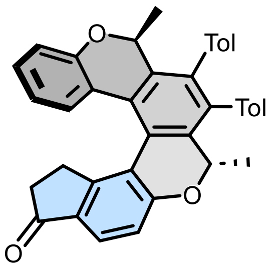

Batch synthesis using $Ni(PPh_3)_2(CO)_2$: Triyne (+)-(*S,S*)-**12** (150 mg, 0.27 mmol, 1.0 equiv.) and $Ni(PPh_3)_2(CO)_2$ (35 mg, 0.05 mmol, 20 mol%) was weighed into a Schlenk vessel under inert atmosphere. Toluene (15 mL) was added and the reaction mixture was inserted into a preheated oil bath set to 120 °C. The reaction was stirred at this temperature for 5 min during which a color change from light yellow to black/brown was observed. The reaction mixture was allowed to cool to ambient temperature. The volatiles were removed under reduced pressure and the residue was purified by column chromatography on silica gel (*n*-hexane:EtOAc 8:1) to give helicene (+)-(*P,S,S*)-**13** (122.8 mg, 81%) as a light yellow amorphous solid.

Synthesis under flow conditions: A Schlenk vessel was charged with triyne (+)-(*S,S*)-**12** (500 mg, 0.9 mmol, 1.0 equiv.) and dry THF (40 mL) under inert atmosphere. The mixture was degassed by three freeze-pump-thaw cycles and after warming to ambient temperature, $CpCo(CO)_2$ (61 µL, 82 mg, 0.5 mmol, 50 mol%) was added by a micro syringe. The reaction mixture was passed through a flow reactor[5] consisting of an HPLC pump, heated stainless steel capillary (volume of 8.0 mL) and a backpressure regulator (250 °C, 80 bar, flow rate 0.7 mL/min, residence time 11.4 min). The stock solution of reactants, waiting to be passed into the reactor, was stored at 0 °C. The reaction mixture was diluted with EtOAc and the volatiles were removed under reduced pressure. The residue was purified by column chromatography on silica gel (*n*-hexane:EtOAc 8:1 to 4:1) to give helicene (+)-(*P,S,S*)-**13** (408.7 mg, 83%) as a light yellow amorphous solid.

$\mathbf{R_f}$ = 0.44 (*n*-hexane:EtOAc 4:1).

Mp: 276-277 °C (crystallized by diffusion of hexane into PhCl).

$[\alpha]^{20}_D$ +559.3 (*c* 0.175, THF).

^{1}**H NMR** (400 MHz, 298 K, $CDCl_3$): δ = 7.69 (d, *J* = 8.2 Hz, 1H), 7.18 – 7.07 (m, 6H), 7.04 (m, 2H), 6.91 – 6.87 (m, 2H), 6.73 – 6.64 (m, 3H), 5.35 (q, *J* = 6.7 Hz, 1H), 5.29 (q, *J* = 6.7 Hz, 1H), 2.51 (ddd, *J* = 17.4, 8.0, 3.4 Hz, 1H), 2.38 (ddd, *J* = 18.7, 8.0, 3.3 Hz, 1H), 2.27 (s, 6H), 2.27 – 2.20 (partially overlapping with tolyl CH_3, m, 1H), 2.03 (ddd, *J* = 17.5, 8.1, 3.3 Hz, 1H), 0.99 (d, *J* = 6.6 Hz, 3H), 0.93 (d, *J* = 6.7 Hz, 3H).

^{13}**C{^{1}H} NMR** (101 MHz, 298 K, $CDCl_3$): δ = 206.1, 160.2, 154.5, 152.9, 139.8, 139.1, 137.6, 136.9, 136.5, 136.4, 134.7, 134.6, 132.7, 130.9, 130.7, 129.5, 129.1, 129.0, 128.9, 128.8, 128.63, 128.61, 126.3, 126.2, 125.0, 124.8, 123.4, 123.0, 122.0, 119.6, 119.5, 74.2, 73.1, 36.9, 26.3, 21.3 (2C), 18.7, 18.3.

HRMS (ESI) *m/z*: ($[M+H]^+$) calcd for $C_{39}H_{33}O_3$ 549.2424, found 549.2419 (Δ = -0.98 ppm).

IR ($CHCl_3$): 3055 (w), 3026 (w), 2956 (w), 2928 (w), 1696 (vs), 1604 (w), 1582 (s), 1548 (w), 1517 (w), 1486 (w), 1445 (m), 1406 (w), 1368 (m), 1331 (m), 1309 (vw), 1294 (w), 1284 (w), 1273 (w), 1261 (m), 1250 (m), 1183 (w), 1150 (m), 1110 (w), 1102 (w), 1068 (m), 1033 (w), 1026 (w), 1022 (w), 967 (w), 944 (w), 936 (w), 823 (w), 819 (w), 618 (w), 548 (vw) cm^{-1}.

Compound (-)-(*M,R,R*)-**13**

Prepared similarly to (+)-(*P,S,S*)-**13** staring from (-)-(*R,R*)-**12** (200 mg, 0.36 mmol, 1.0 equiv.) and $Ni(PPh_3)_2(CO)_2$ (46.6 mg, 0.07 mmol, 20 mol%). Yielded (-)-(*M,R,R*)-**13** (147 mg, 74%) as a light yellow amorphous solid.

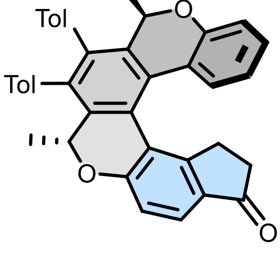

NMR spectra in accordance with (+)-(*P,S,S*)-**13**.

$[\alpha]^{20}_D$ -560.2 (*c* 0.181, THF).

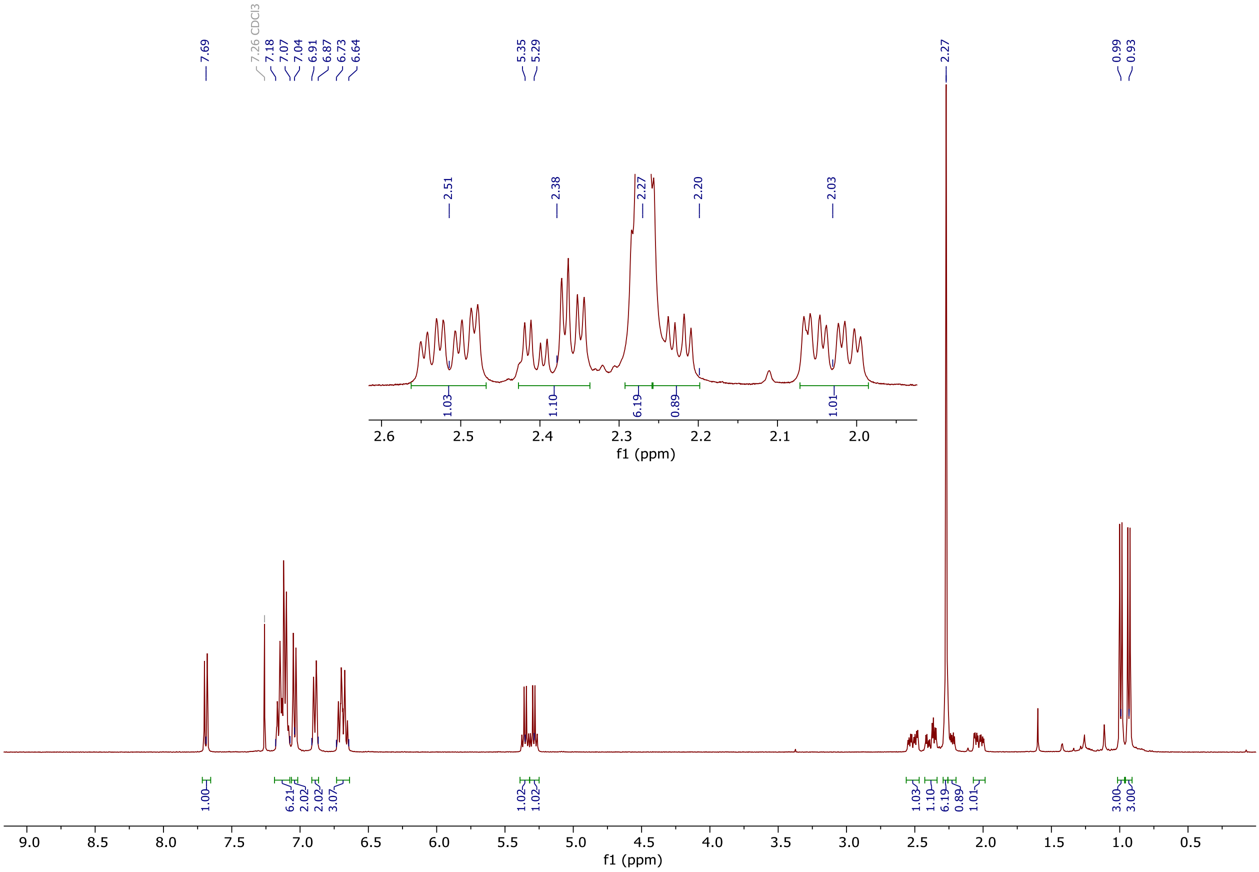

Figure S15: ^{1}H NMR of (+)-(*P,S,S*)-**13** (400 MHz, 298 K, $CDCl_3$).

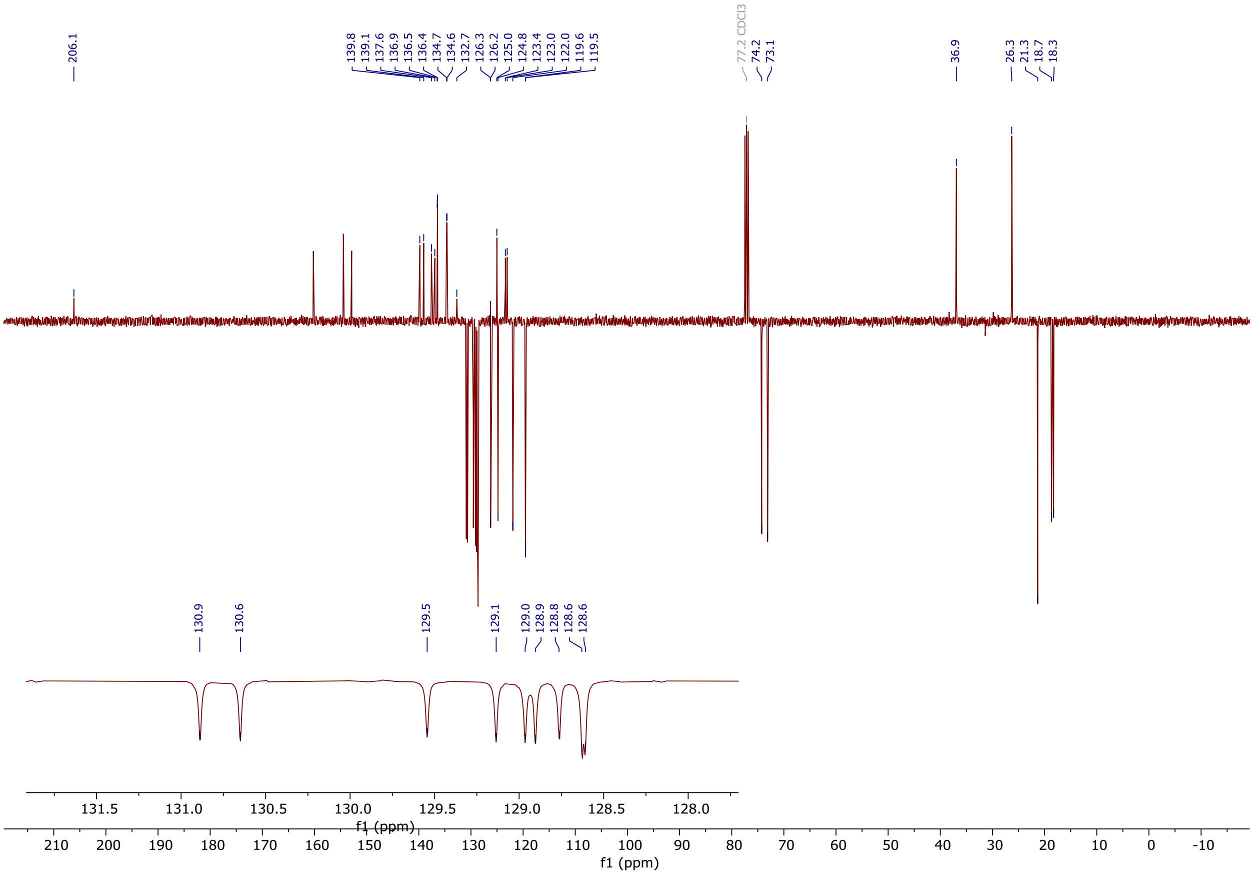

Figure S16: ^{13}C APT NMR of (+)-(*P,S,S*)-**13** (101 MHz, 298 K, $CDCl_3$).

Oxa[7]helicene (-)-(*M*,*R*,*R*)-**44**

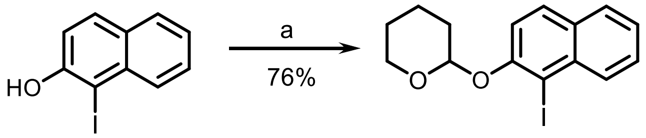

40

(a) DHP (5.0 equiv.), PPTS (10 mol%), DCM, rt, 18 h, 76%.

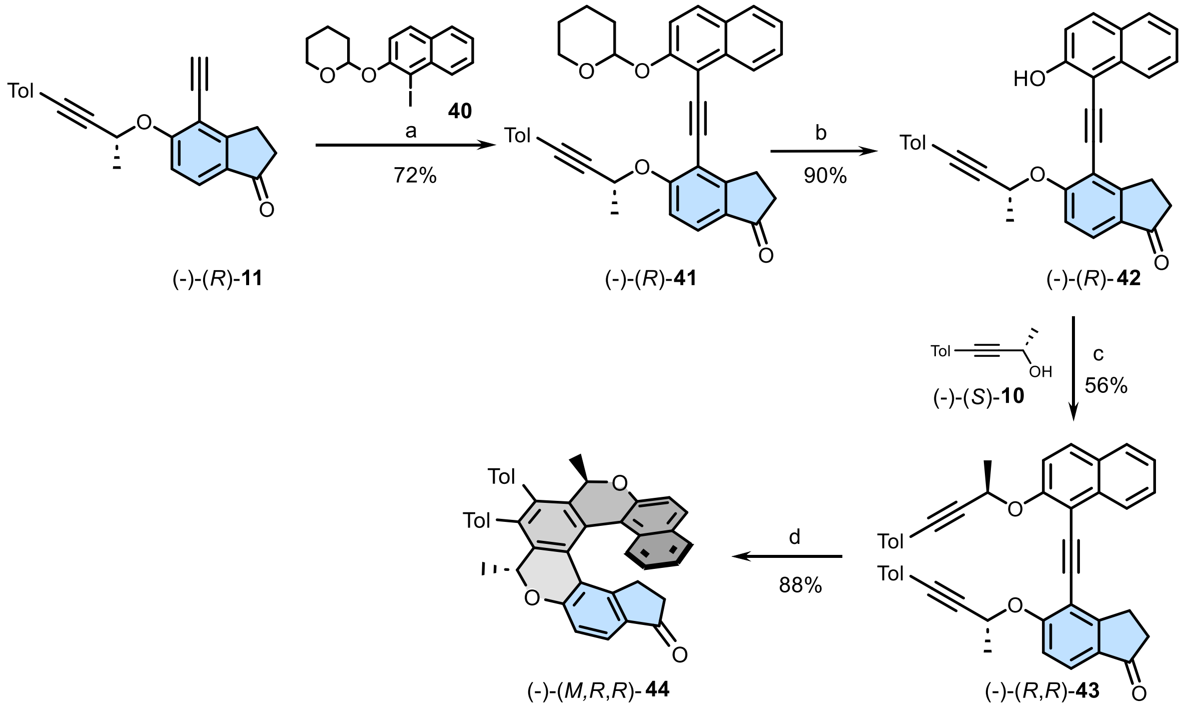

(a) **40** (2.0 equiv.), $Pd(PPh_3)_4$ (5 mol%), CuI (10 mol%), Et_3N-toluene (2:5), 50 °C, 3 h, 72%; (b) *p*-TsOH (10 mol%), THF-MeOH (1:1), rt, 18 h, 90%; (c) (-)-(*S*)-**10** (1.2 equiv.), PPh_3 (1.1 equiv.), DIAD (1.2 equiv.), benzene, 0 °C to rt, 3 h, 56%; (d) $Ni(PPh_3)_2(CO)_2$ (20 mol%), toluene, 120 °C, 10 min, then 200 °C, 15 min, 88%.

2-((1-Iodonaphthalen-2-yl)oxy)tetrahydro-2*H*-pyran 40

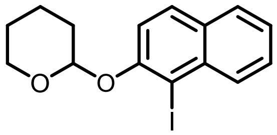

A 250 mL round bottom flask under air was charged with 1-iodo-2-naphthol (3.970 g, 14.7 mmol, 1.0 equiv.) and 3,4-dihydropyran (6.7 mL, 6.18 g, 73.5 mmol, 5.0 equiv.). The starting materials were dissolved in DCM (50 mL), and pyridinium *p*-toluenesulfonate (0.369 g, 1.47 mmol, 10 mol%) was added. The reaction mixture was allowed to stir at room temperature for 18 h. The volatiles were removed under reduced pressure and the residue was purified by column chromatography on silica gel (*n*-hexane:DCM:EtOAc 4:4:1) to give product **40** (3.95 g, 76%) as a viscous yellow liquid.

R_f = 0.47 (*n*-hexane:EtOAc 4:1).

^{1}H NMR (400 MHz, 298 K, $CDCl_3$): δ = 8.16 (ddt, *J* = 8.6, 1.2, 0.7 Hz, 1H), 7.78 (dt, *J* = 9.0, 0.6 Hz, 1H), 7.74 (ddt, *J* = 8.1, 1.2, 0.6 Hz, 1H), 7.54 (ddd, *J* = 8.4, 6.9, 1.3 Hz, 1H), 7.42 – 7.36 (m, 2H), 5.70 (t, *J* = 2.9 Hz, 1H), 3.95 (m, 1H), 3.65 – 3.59 (m, 1H), 2.34 – 2.21 (m, 1H), 2.15 – 2.06 (m, 1H), 1.99 – 1.88 (m, 1H), 1.84 – 1.62 (m, 3H).

^{13}C{^{1}H} NMR (101 MHz, 298 K, $CDCl_3$): δ = 154.4, 135.8, 131.5, 130.5, 130.2, 128.3, 128.0, 124.7, 116.4, 97.2, 89.6, 62.0, 30.4, 25.4, 18.5.

HRMS (EI) *m/z*: ($[M]^+$) calcd for $C_{15}H_{15}O_2I$ 354.0111, found 354.0105 (Δ = -1.76 ppm).

IR ($CHCl_3$): 2949 (m), 2925 (w), 2874 (w), 2854 (w), 1622 (m), 1594 (m), 1556 (w), 1501 (m), 1460 (m), 1455 (m), 1442 (w), 1428 (w), 1390 (w), 1350 (m-w), 1339 (m), 1324 (m-w), 1286 (w), 1267 (m), 1256 (s), 1239 (s), 1203 (m), 1183 (m), 1121 (m), 1114 (m), 1150 (m-w), 1037 (m), 1022 (m), 997 (vs), 957 (m), 873 (s-m), 819 (m), 805 (m) cm^{-1}.

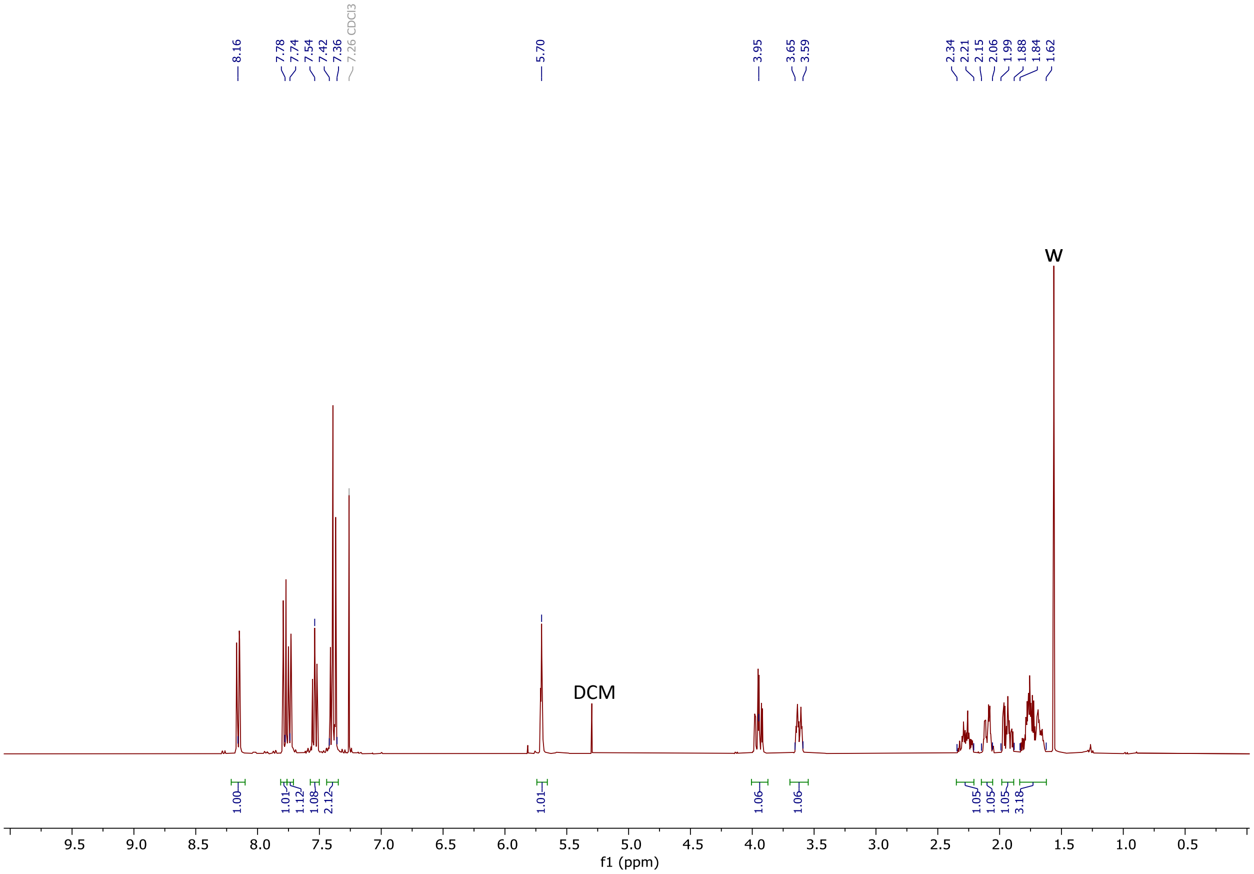

Figure S17: ^{1}H NMR of 40 (400 MHz, 298 K, $CDCl_3$) (w = water, DCM = dichloromethane).

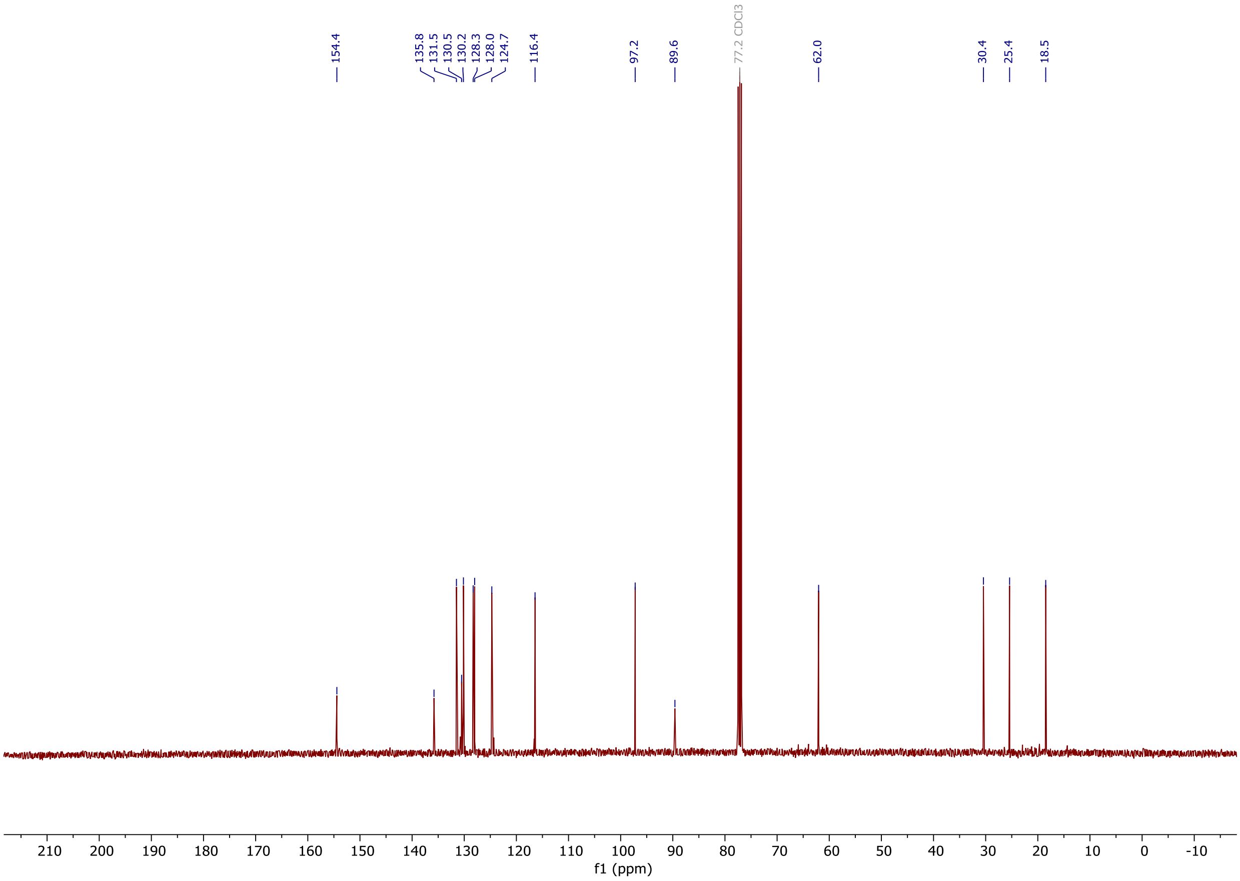

Figure S18: ^{13}C NMR of 40 (101 MHz, 298 K, $CDCl_3$).

(-)-4-((2-((Tetrahydro-2*H*-pyran-2-yl)oxy)naphthalen-1-yl)ethynyl)-5-(((*R*)-4-(*p*-tolyl)but-3-yn-2-yl)oxy)-2,3-dihydro-1*H*-inden-1-one (-)-(*R*)-**41**

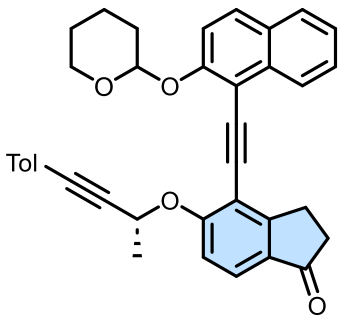

Iodide **40** (1.13 g, 3.2 mmol, 2.0 equiv.), $Pd(PPh_3)_4$ (92 mg, 0.08 mmol, 5 mol%) and CuI (30 mg, 0.16 mmol, 10 mol%) were combined in a dry Schlenk flask under inert atmosphere. Then toluene (15 mL) and triethylamine (8 mL) were added. Diyne (-)-(*R*)-**11** (500 mg, 1.6 mmol, 1.0 equiv.) was weighed separately into a second Schlenk flask and dissolved in toluene (5 mL). Both solutions were degassed by three freeze-pump-thaw cycles. The Schlenk flask with the reaction was inserted into a preheated oil bath set to 50 °C and the solution of diyne (-)-(*R*)-**11** was added dropwise. The mixture was stirred at 50 °C for 3 h. The volatiles were removed under reduced pressure and the residue was purified by column chromatography on silica gel (*n*-hexane:EtOAc 5:1 to 3:1) to give product (-)-(*R*)-**44** (623 mg, 72%) as a light yellow crystalline solid.

R_f = 0.27 (*n*-hexane:EtOAc 2:1).

Mp: 70-71 °C (*n*-hexane).

$[\alpha]^{20}_D$ -269.0 (*c* 0.333, THF), mixture of diastereoisomers.

^{1}H NMR (mixture of diastereoisomers) (400 MHz, 298 K, $CDCl_3$): δ = 8.64 (dd, *J* = 8.4, 1.1 Hz, 1H), 7.84 – 7.79 (m, 2H), 7.76 (d, *J* = 8.6 Hz, 1H), 7.56 (m, 1H), 7.50 (d, *J* = 9.1 Hz, 1H), 7.42 (ddd, *J* = 8.1, 6.8, 1.2 Hz, 1H), 7.32 (d, *J* = 8.6 Hz, 1H), 7.30 – 7.24 (m, 2H, overlapping with the solvent signal), 7.11 – 7.05 (m, 2H), 5.73 (m, 1H), 5.35 (q, *J* = 6.5 Hz, 1H), 4.05 (td, *J* = 11.0, 3.1 Hz, 1H), 3.66 (m, 1H), 3.34 (m, 2H), 2.75 (m, 2H), 2.33 (s, 3H), 2.29 – 2.16 (m, 1H), 2.14 – 2.06 (m, 1H), 2.03 – 1.99 (m, 1H), 1.98 (d, *J* = 6.5, 3H), 1.81 – 1.70 (m, 2H), 1.69 – 1.61 (m, 1H).

^{13}C{^{1}H} NMR (mixture of diastereoisomers) (101 MHz, 298 K, $CDCl_3$): δ = 205.6, 163.2, 159.3, 156.6, 156.5, 139.1, 134.6, 131.8, 130.9, 130.3, 129.2, 128.2, 127.2, 125.9, 124.7, 124.6, 119.0, 116.74, 116.68, 113.43, 113.41, 112.2, 97.3, 97.2, 93.7, 91.8, 87.1, 86.6, 65.9, 62.1, 36.6, 30.7, 30.6, 25.92, 25.91, 25.4, 22.68, 22.67, 21.6, 18.81, 18.78.

HRMS (ESI) *m/z*: ($[M+H]^+$) calcd for $C_{37}H_{33}O_4$ 541.2373, found 541.2372 (Δ = -0.25 ppm).

IR ($CHCl_3$): 3060 (w), 2949 (m-w), 2927 (w), 2884 (w), 2872 (w), 2854 (w), 2243 (w), 2225 (w), 2208 (w), 1698 (vs), 1621 (w), 1592 (s-m), 1584 (s), 1510 (m), 1482 (w-m), 1465 (w), 1454 (w), 1443 (w), 1434 (w), 1428 (w), 1356 (w), 1331 (m), 1324 (m), 1293 (w), 1278 (m), 1266 (vs-s), 1259 (vs), 1230 (w), 1183 (w), 1171 (w), 1147 (w), 1121 (m), 1084 (vs-s), 1047 (m), 1037 (m), 1021 (m), 994 (m), 975 (w), 943 (w), 876 (w), 836 (w), 819 (s-m) cm^{-1}.

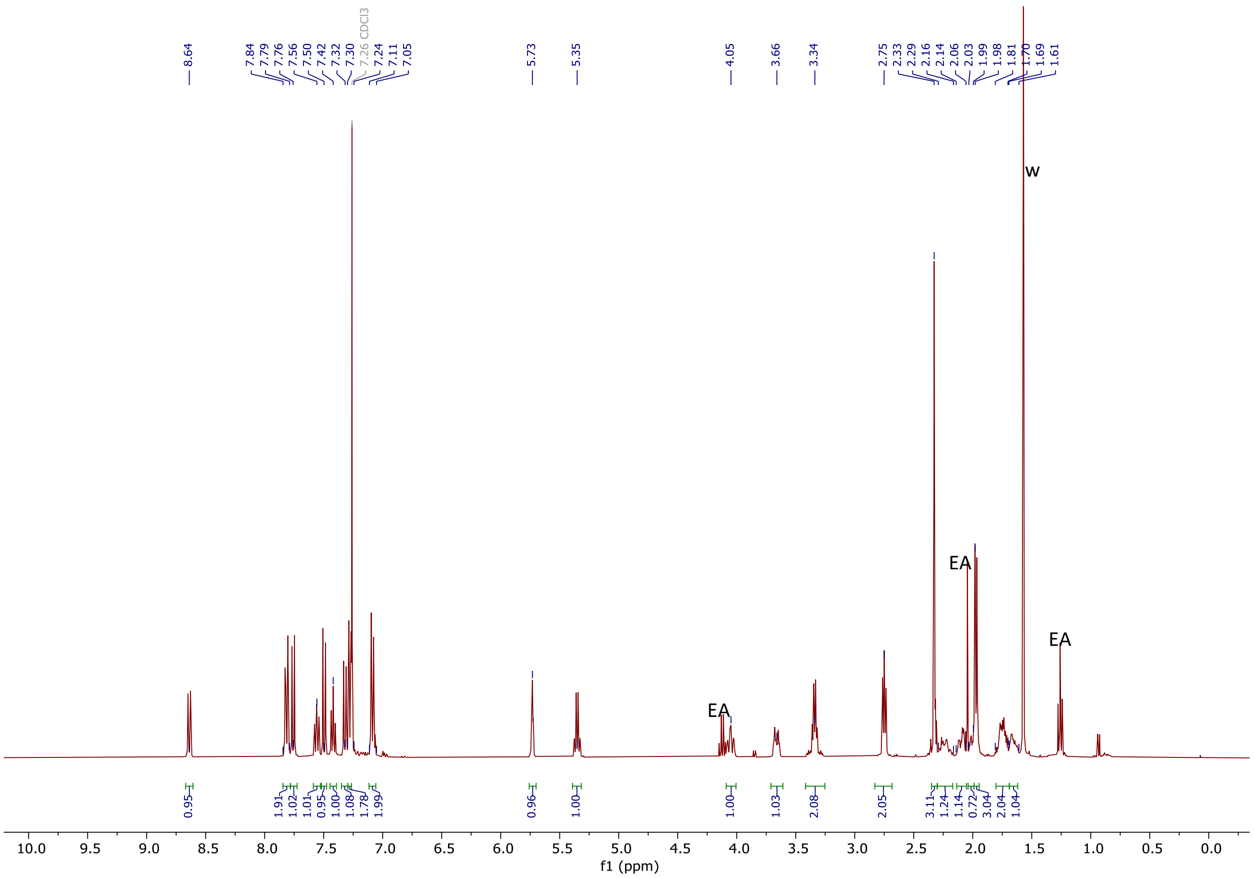

Figure S19: ^{1}H NMR of (-)-(*R*)-**41** (400 MHz, 298 K, $CDCl_3$) (EA = ethyl acetate, w = water).

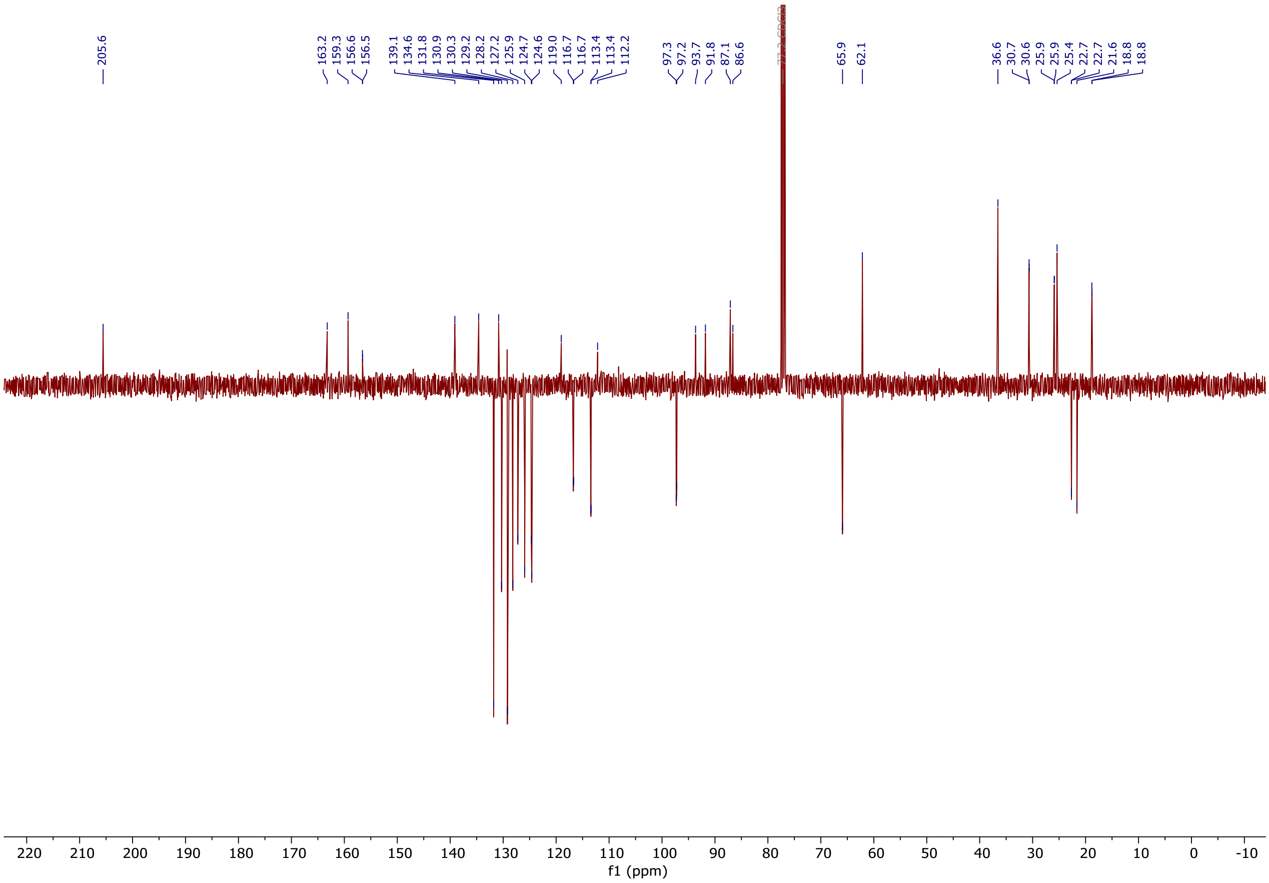

Figure S20: ^{13}C APT NMR of (-)-(*R*)-**41** (101 MHz, 298 K, $CDCl_3$).

(-)-(*R*)-4-((2-Hydroxynaphthalen-1-yl)ethynyl)-5-((4-(*p*-tolyl)but-3-yn-2-yl)oxy)-2,3-dihydro-1*H*-inden-1-one (-)-(*R*)-**42**

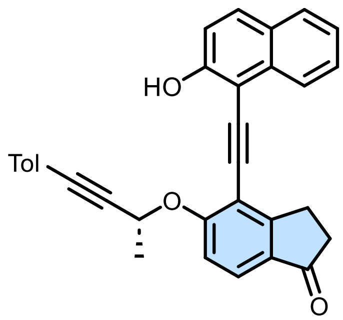

The THP-protected diyne (-)-(*R*)-**41** (620 mg, 1.15 mmol, 1.0 equiv.) was dissolved in a mixture of THF-MeOH (1:1, 30 mL total) and *p*-TsOH·H_2O (22 mg, 0.1 mmol, 10 mol%) was added and the reaction mixture was allowed to stir at room temperature for 18 h. The volatiles were removed under reduced pressure and the residue was dissolved in DCM (50 mL) and extracted with water (20 mL) and brine (20 mL). The organic layer was dried over anhydrous $MgSO_4$ and the solvents were evaporated *in vacuo*. The residue was purified by column chromatography on silica gel (*n*-hexane:EtOAc:DCM 5:1.0 to 2:1:2) to give product (-)-(*R*)-**42** (471 mg, 90%) as an off-white amorphous solid.

R_f = 0.17 (*n*-hexane:EtOAc 2:1).

Mp: 170 – 171 °C (recrystallized from *n*-hexane-EtOAc).

$[\alpha]^{20}_D$ -347.0 (*c* 0.481, THF).

^{1}H NMR (400 MHz, 298 K, $CDCl_3$): δ = 8.18 (d, *J* = 1.1 Hz, 1H), 7.79 (dd, *J* = 9.0, 7.7 Hz, 3H), 7.58 (ddd, *J* = 8.2, 6.9, 1.3 Hz, 1H), 7.40 (ddd, *J* = 8.1, 6.9, 1.2 Hz, 1H), 7.34 – 7.27 (m, 3H, overlapping the solvent signal), 7.11 – 7.07 (m, 2H), 6.80 (brs, 1H), 5.34 (q, *J* = 6.5 Hz, 1H), 3.37 (m, 2H), 2.80 (m, 2H), 2.33 (s, 3H), 1.96 (d, *J* = 6.5 Hz, 3H).

^{13}C{^{1}H} NMR (101 MHz, 298 K, $CDCl_3$): δ = 205.2, 162.4, 158.1, 156.8, 139.3, 133.0, 131.8, 131.1, 131.0, 129.2, 128.5, 127.6, 125.0, 124.9, 124.2, 118.8, 116.5 (2C), 112.9, 110.9, 103.1, 94.8, 91.8, 87.5, 86.1, 66.2, 36.5, 25.8, 22.5, 21.6.

HRMS (ESI) *m/z*: ($[M+H]^+$) calcd for $C_{32}H_{25}O_3$ 457.1798, found 457.1799 (Δ = 0.18 ppm).

IR ($CHCl_3$): 3527 (w), 3456 (w), 3061 (w), 2996 (w), 2925 (w), 2872 (w), 2244 (w), 2227 (w), 2200 (w), 1701 (vs), 1621 (w), 1589 (s), 1520 (m-w), 1511 (m), 1481 (w), 1464 (m), 1445 (w), 1430 (w), 1391 (w), 1378 (w), 1334 (m), 1320 (s-m), 1277 (m), 1255 (vs), 1229 (w), 1202 (w), 1182 (vw), 1165 (w), 1143 (m), 1120 (w), 1106 (w), 1082 (s), 1046 (m), 1021 (w), 946 (w), 833 (w), 819 (s) cm^{-1}.

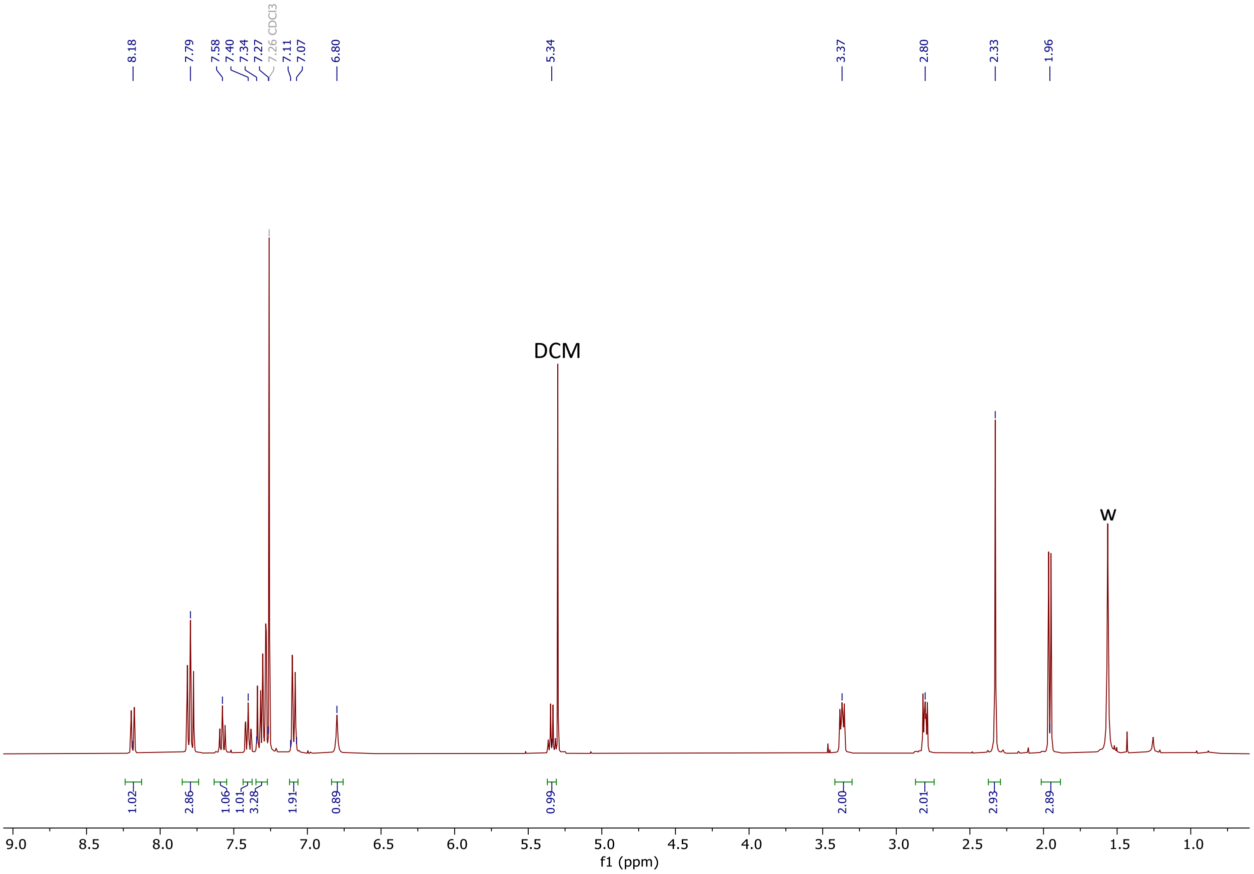

Figure S21: ¹H NMR of (-)-(*R*)-**42** (400 MHz, 298 K, $CDCl_3$) (DCM = dichloromethane, w = water).

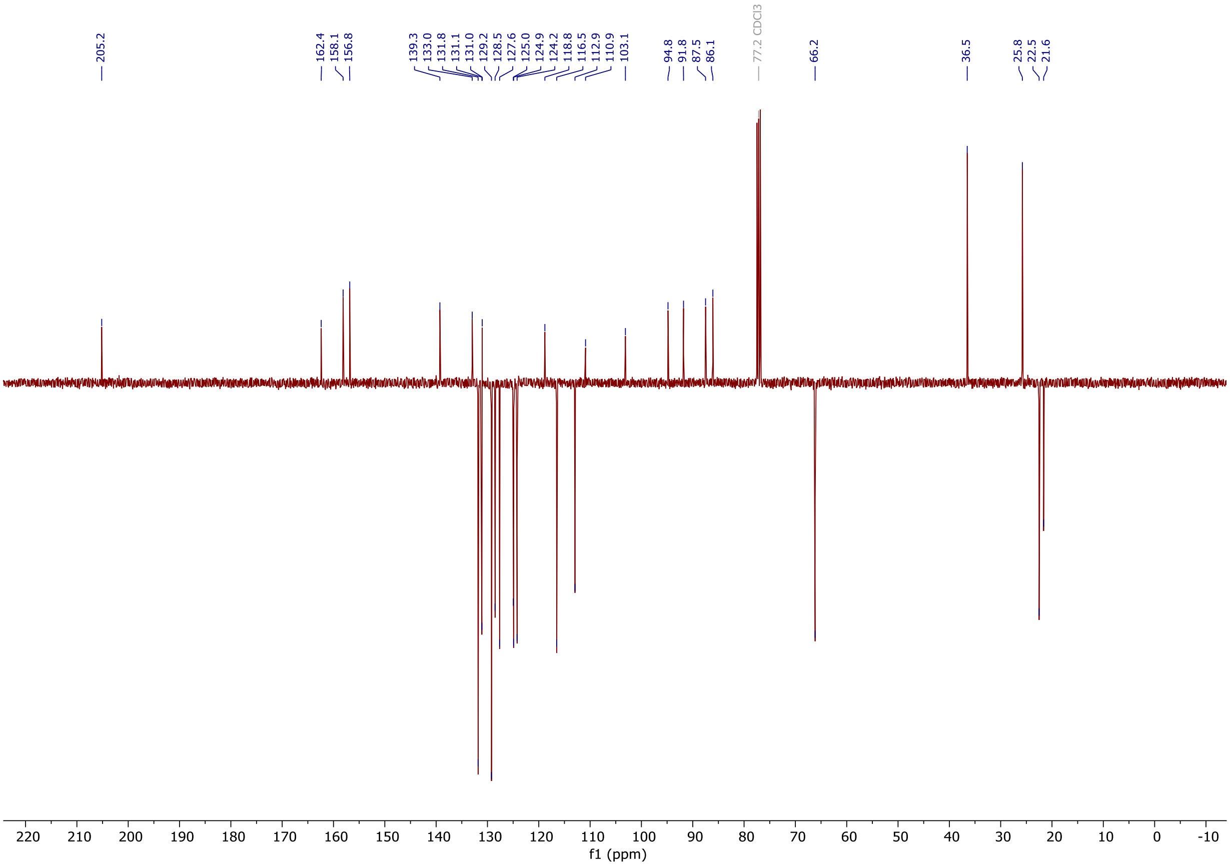

Figure S22: ¹³C NMR of (-)-(*R*)-**42** (101 MHz, 298 K, $CDCl_3$).

(-)-5-(((*R*)-4-(*p*-Tolyl)but-3-yn-2-yl)oxy)-4-((2-(((*R*)-4-(*p*-tolyl)but-3-yn-2-yl)oxy)naphthalen-1-yl)ethynyl)-2,3-dihydro-1*H*-inden-1-one (-)-(*R,R*)-**43**

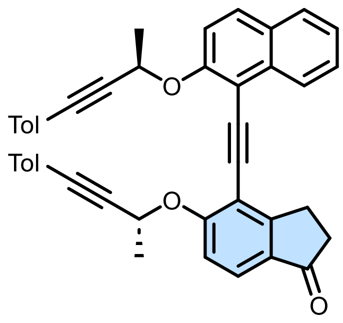

A solution of alkyne (-)-(*R*)-**42** (440 mg, 0.96 mmol, 1.0 equiv.), (-)-(*S*)-**10** (185 mg, 1.16 mmol, 1.2 equiv.), and PPh_3 (278 mg, 1.06 mmol, 1.1 equiv.) in benzene (15 mL) was cooled to 0 °C and diisopropyl azodicarboxylate (0.23 mL, 1.16 mmol, 1.2 equiv.) was added dropwise. The reaction was stirred at 0 °C for additional 5 min and then allowed to warm to room temperature. The resulting yellow solution was allowed to stir at room temperature for 3 h. The volatiles were removed under reduced pressure and the residue was purified by column chromatography on silica gel (*n*-hexane:EtOAc 5:1) and then further purified by recrystallization from heptane, filtered on a grade 4 frit, washed with hexane (10 mL), and dried under vacuum to give product (-)-(*R,R*)-**43** (325 mg (56%).

R_f = 0.33 (*n*-hexane:EtOAc 2:1).

Mp: 74 °C (*n*-hexane).

$[\alpha]^{20}_D$ -495.2 (*c* 0.302, THF).

^{1}H NMR (400 MHz, 298 K, $CDCl_3$): δ = 8.65 (dd, *J* = 8.4, 1.1 Hz, 1H), 7.85 (m, 2H), 7.77 (d, *J* = 8.6 Hz, 1H), 7.59 (ddd, *J* = 8.3, 6.8, 1.3 Hz, 1H), 7.55 (d, *J* = 9.0 Hz, 1H), 7.45 (ddd, *J* = 8.1, 6.8, 1.2 Hz, 1H), 7.35 – 7.27 (m, 5H, overlapping with NMR solvent signal), 7.10 (t, *J* = 7.8 Hz, 4H), 5.44 (q, *J* = 6.5 Hz, 1H), 5.37 (q, *J* = 6.5 Hz, 1H), 3.40 (m, 2H), 2.73 (ddd, *J* = 7.0, 4.9, 1.9 Hz, 2H), 2.35 (s, 3H), 2.33 (s, 3H), 2.02 (d, *J* = 6.5 Hz, 3H), 1.90 (d, *J* = 6.5 Hz, 3H).

^{13}C{^{1}H} NMR (101 MHz, 298 K, $CDCl_3$): δ = 205.7, 163.1, 159.6, 157.2, 139.1, 138.9, 134.6, 131.8, 131.7, 130.9, 130.1, 129.3, 129.2, 129.1, 128.2, 127.3, 126.0, 124.8, 124.5, 119.3, 119.1, 117.0, 113.5, 112.2, 109.1, 93.6, 92.3, 87.8, 87.1, 86.7, 86.5, 66.7, 65.9, 36.6, 26.0, 22.8, 22.7, 21.60, 21.57.

HRMS (ESI) *m/z*: ($[M+H]^+$) calcd for $C_{43}H_{35}O_3$ 599.2581, found 599.2582 (Δ = 0.24 ppm).

IR ($CHCl_3$): 3084 (w), 3059 (w), 2994 (w), 2938 (w), 2925 (w), 2870 (w), 2225 (w), 2208 (w), 1697 (vs), 1621 (w), 1584 (s), 1510 (s), 1483 (w), 1464 (w), 1444 (w), 1434 (w), 1427 (w), 1375 (w), 1327 (s-m), 1293 (w), 1266 (vs-s), 1259 (vs-s), 1180 (w), 1171 (w), 1148 (w), 1119 (m), 1105 (m-w), 1086 (vs), 1040 (s-m), 1020 (m), 947 (w), 911 (w), 836 (w), 819 (s) cm^{-1}.

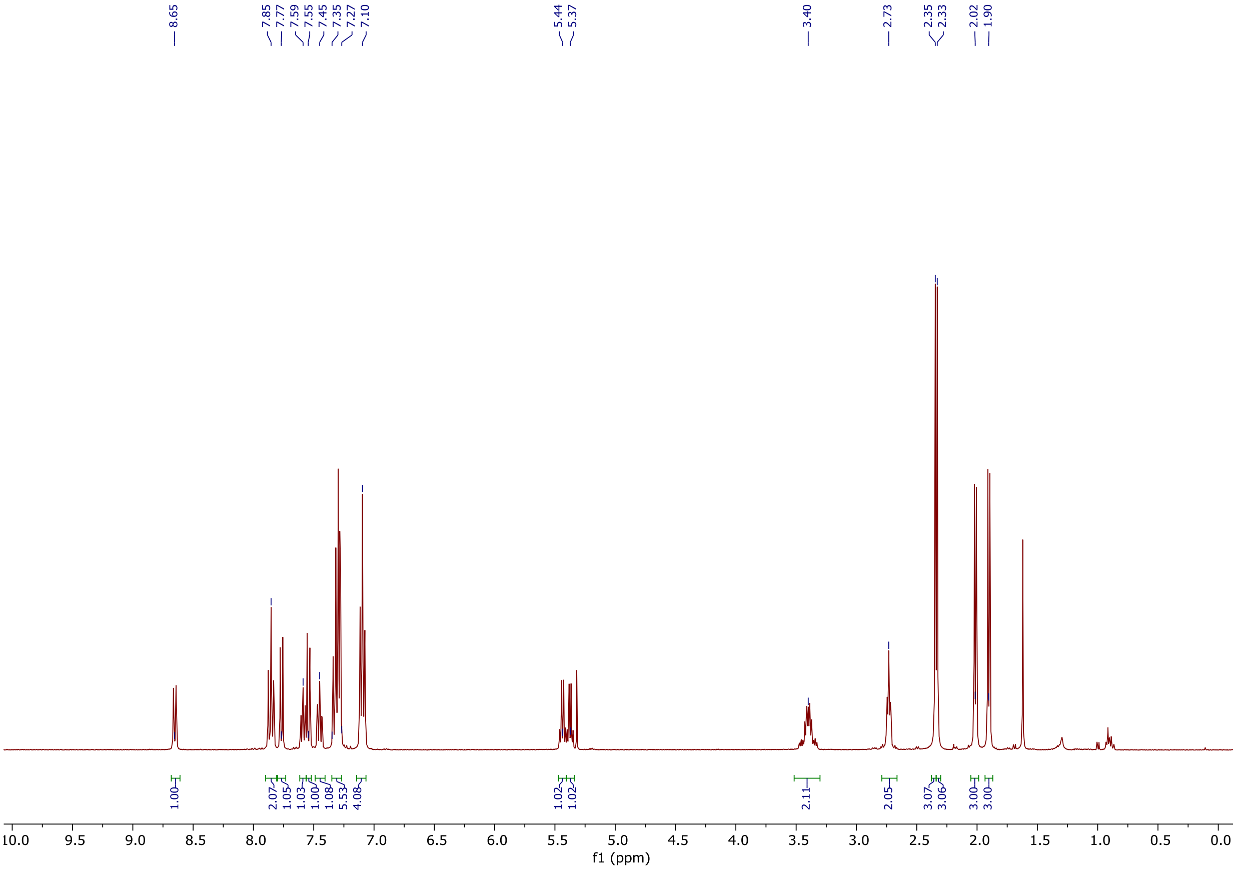

Figure S23: ¹H NMR of (-)-(*R,R*)-**43** (400 MHz, 298 K, $CDCl_3$).

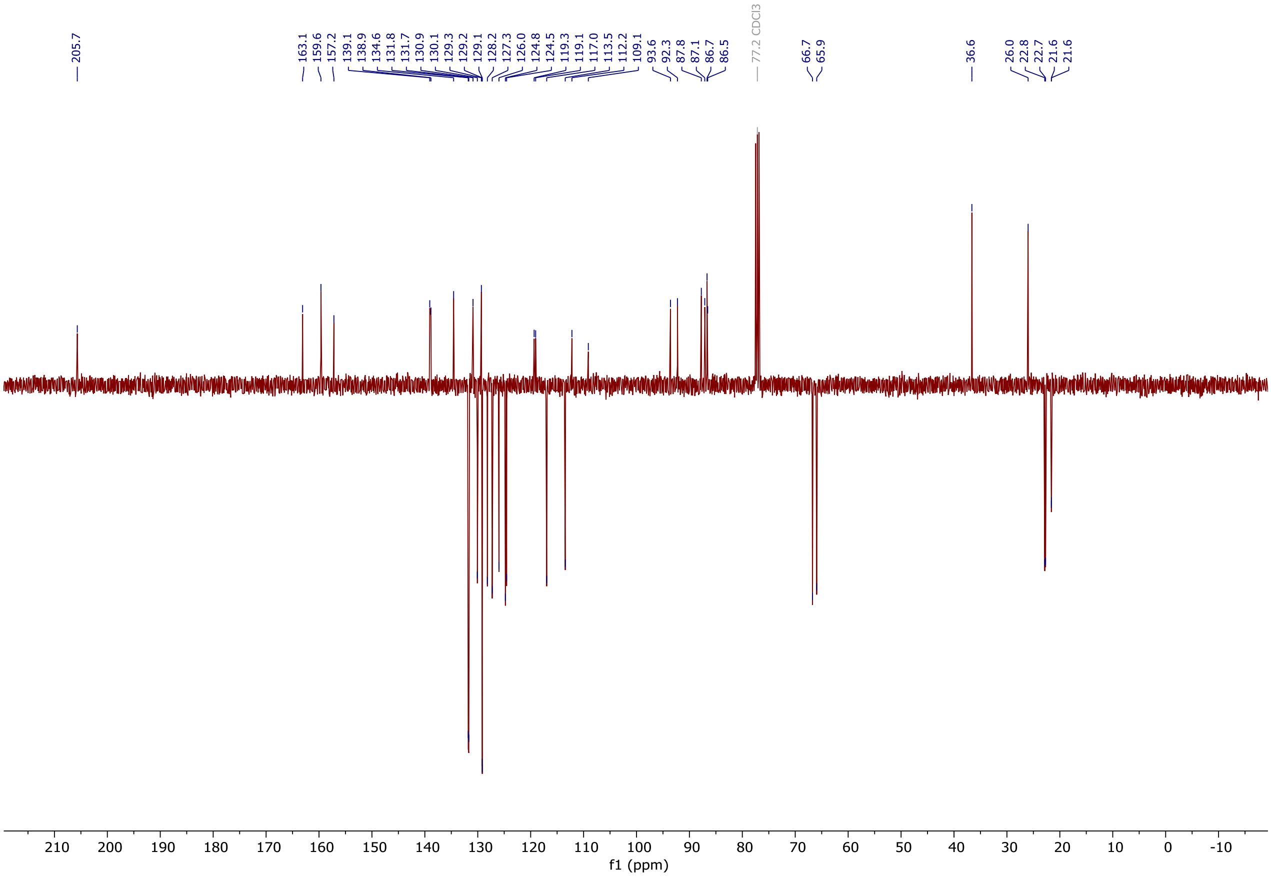

Figure S24: ¹³C APT NMR of (-)-(*R,R*)-**43** (101 MHz, 298 K, $CDCl_3$).

Compound (-)-(*M,R,R*)-**44**

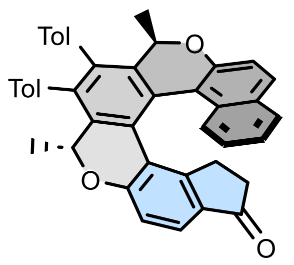

Triyne (-)-(*R,R*)-**43** (100 mg, 0.17 mmol, 1.0 equiv.) and $Ni(CO)_2(PPh_3)_2$ (21.4 mg, 0.03 mmol, 20 mol%) were weighed into a dry Schlenk vessel under inert atmosphere. Dry toluene (10 mL) was added and the reaction mixture was inserted into a preheated oil bath set to 120 °C and stirred at that temperature for 10 min. The reaction mixture was allowed to cool to room temperature before transferring into a round bottom flask. The solvent was removed under reduced pressure and the residue was purified by column chromatography on silica gel (*n*-hexane:EtOAc 6:1) to give the crude product as a mixture of diastereoisomers (*M,R,R*)- and (*P,R,R*)-**44**. The mixture was dissolved in PhCl (3 mL) and heated in a microwave reactor at 200 °C for 15 min under inert atmosphere. The evaporation of the solvent gave the product (-)-(*M,R,R*)-**44** (88 mg, 88%) as a single diastereoisomer, a yellow solid.

$\mathbf{R_f}$ = 0.19 (*n*-hexane:EtOAc 9:1).

Mp: Decomposition >170 °C) (PhCl).

$[\alpha]^{20}_D$ -547.7 (*c* 0.590, THF).

^{1}H NMR (400 MHz, 298 K, $CDCl_3$): δ = 7.72 (d, *J* = 8.7 Hz, 1H), 7.59 (m, 1H), 7.40 (d, *J* = 8.2 Hz, 1H), 7.33 – 7.27 (m, 2H), 7.22 – 7.15 (m, 2H), 7.14 – 7.09 (m, 2H), 7.09 – 7.02 (m, 2H), 6.94 (m, 2H), 6.81 (m, 3H), 5.38 (m, 2H), 2.29 (s, 6H), 2.18 – 2.03 (m, 2H), 1.78 – 1.66 (m, 1H), 1.08 (d, *J* = 6.7 Hz, 3H), 1.05 – 0.98 (m, 4H, doublet of the CH_3 group attached to the chiral center overlapping with one proton of the CH_2 of the cyclopentenone).

^{13}C{^{1}H} NMR (101 MHz, 298 K, $CDCl_3$): δ = 205.5, 159.4, 153.8, 152.2, 139.6, 139.5, 137.4, 136.5, 136.4 (2C), 134.9, 134.8, 131.8, 130.8, 130.7, 130.5, 130.1, 129.11, 129.06, 128.80, 128.77, 128.7, 128.6, 128.3, 127.7, 125.0, 124.81, 124.77, 124.51, 124.49, 124.4, 124.0, 120.0, 119.3, 119.1, 74.2, 73.5, 36.6, 25.4, 21.3 (2C), 18.5, 18.1.

HRMS (ESI) *m/z*: ($[M+H]^+$) calcd for $C_{43}H_{35}O_3$ 599.2581, found 599.2574 (Δ = -1.09 ppm).

IR ($CHCl_3$): 3088 (vw), 3051 (w), 2990 (w), 2929 (w), 2868 (w), 1694 (vs), 1618 (w), 1592 (m), 1582 (s), 1516 (w), 1464 (w), 1445 (w), 1429 (w), 1382 (w), 1367 (m-w), 1331 (w), 1284 (w), 1251 (m-w), 1228 (m-w), 1183 (w), 1150 (m-w), 1136 (w), 1111 (vw), 1067 (w), 1055 (w), 1019 (w), 1005 (w), 965 (w), 904 (w), 865 (w), 848 (w), 532 (w) cm^{-1}.

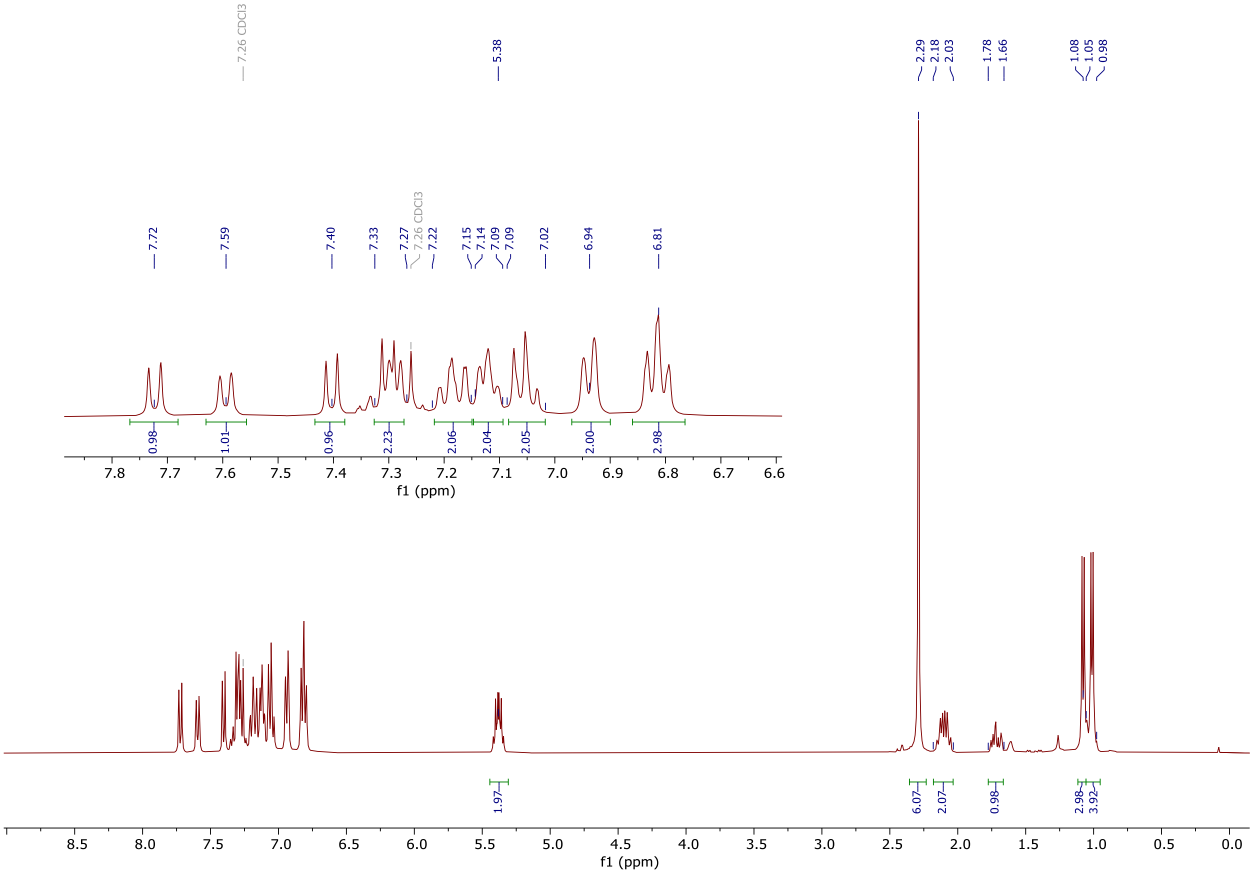

Figure S25: ^{1}H NMR of (-)-(*M,R,R*)-**44** (400 MHz, 298 K, $CDCl_3$).

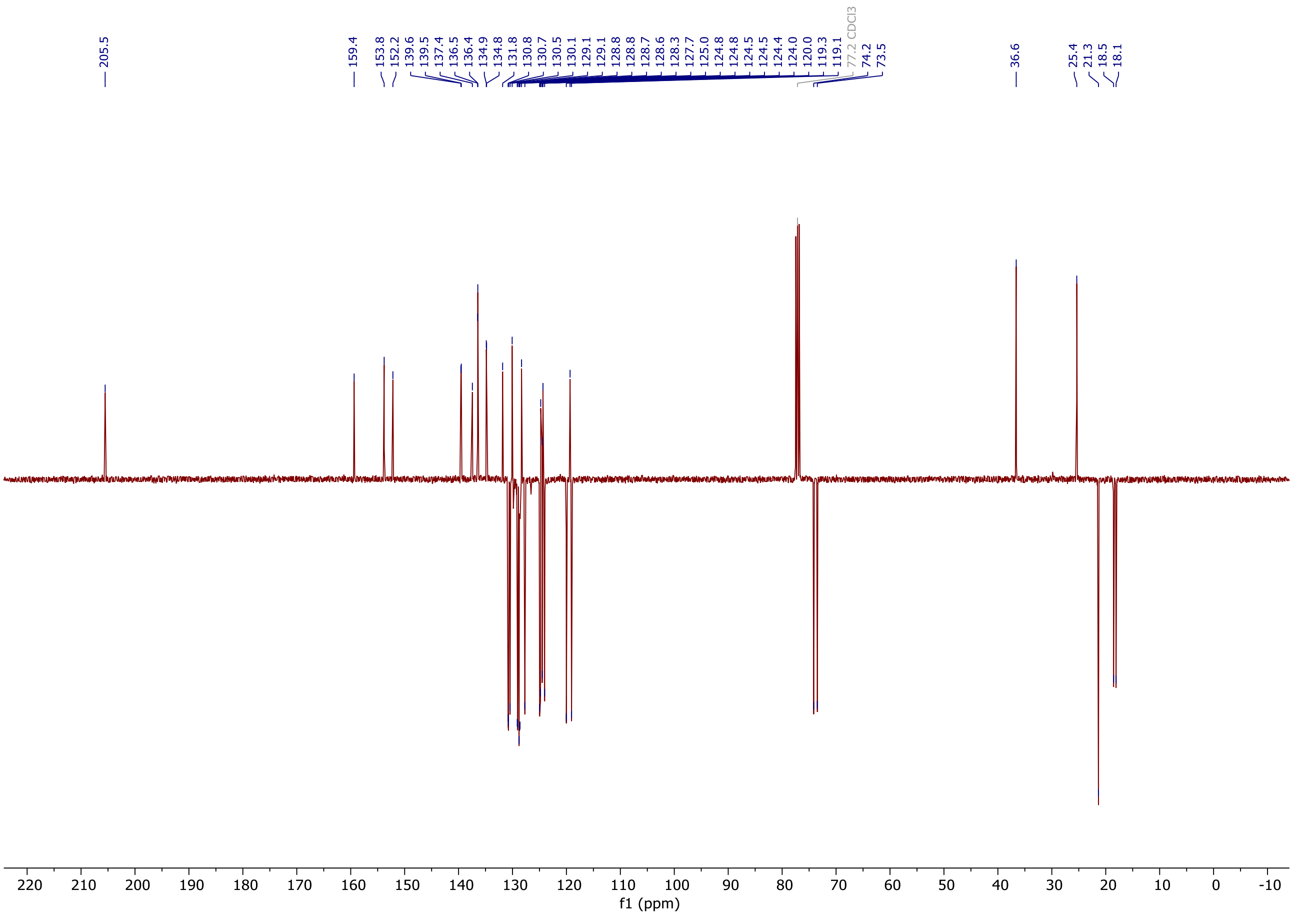

Figure S26: ^{13}C APT NMR of (-)-(*M,R,R*)-**44** (101 MHz, 298 K, $CDCl_3$).

Synthesis of oxa[6]helicene (-)-(*M*,*R*)-**49** with only one chiral center

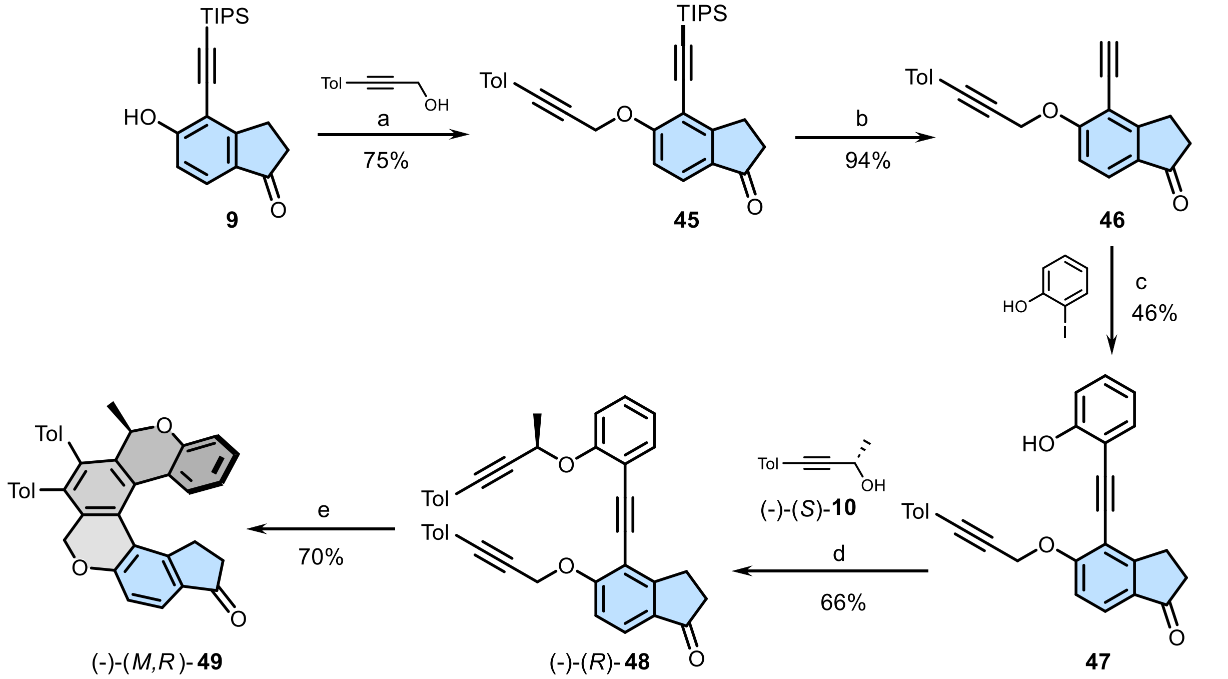

(a) 3-(*p*-Tolyl)-2-propyn-1-ol (1.1 equiv.), PPh_3 (1.1 equiv.), DIAD (1.2 equiv.), benzene, 0 °C to rt, 3 h, 75%; (b) TBAF·$3H_2O$ (1.0 equiv.), THF, 0 °C, 5 min, 94%; (c) 2-iodophenol (3.0 equiv.), $Pd(PPh_3)_4$ (5 mol%), CuI (5 mol%), Et_3N (1.25 equiv.), toluene, 50 °C, 40 h, 46%; (d) (-)-(*S*)-**10** (1.2 equiv.), PPh_3 (1.1 equiv.), DIAD (1.2 equiv.), benzene, 0 °C to rt, 4 h, 66%; (e) $Ni(PPh_3)_2(CO)_2$ (20 mol%), toluene, 120 °C, 5 min, 70%.

((4-(*p*-Tolyl)prop-2-yn-1-yl)oxy)-4-((triisopropylsilyl)ethynyl)-2,3-dihydro-1*H*-inden-1-one 45

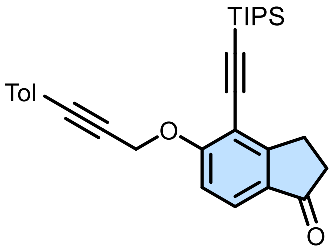

A solution of compound **9** (1.500 g, 4.6 mmol, 1.0 equiv.), 3-(4-methylphenyl)-2-propyn-1-ol (0.734 g, 5.0 mmol, 1.1 equiv.), and PPh_3 (1.317 g, 5.0 mmol, 1.1 equiv.) in benzene (30 mL) under inert atmosphere was inserted into an ice/water bath. Diisopropyl azodicarboxylate (1.1 mL, 1.11 g, 5.5 mmol, 1.2 equiv.) was added dropwise and the resulting solution was stirred for 10 min and then warmed to ambient temperature and stirred for additional 3 h. The solvent was evaporated and the residue was subjected to column chromatography on silica gel (*n*-hexane:EtOAc 8:1) to give product **45** (1.564 g, 75%) as a white amorphous solid.

$\mathbf{R_f}$ = 0.50 (*n*-hexane:EtOAc 4:1).

^{1}H NMR (400 MHz, 298 K, $CDCl_3$): δ = 7.71 (d, *J* = 8.6 Hz, 1H), 7.33 – 7.28 (m, 2H), 7.15 – 7.08 (m, 3H), 5.05 (s, 2H), 3.15 (m, 2H), 2.70 (m, 2H), 2.34 (s, 3H), 1.16 (s, 21H).

^{13}C{^{1}H} NMR (101 MHz, 298 K, $CDCl_3$): δ = 205.5, 163.9, 160.5, 139.2, 131.8, 131.0, 129.2, 124.9, 119.1, 112.6, 111.6, 101.2, 99.1, 88.4, 82.4, 58.1, 36.5, 25.9, 21.7, 18.8, 11.4.

HRMS (ESI) *m/z*: ($[M+H]^+$) calcd for $C_{30}H_{37}O_2Si$ 457.2557, found 457.2554 (Δ = -0.83 ppm).

IR ($CHCl_3$): 3084 (vw), 3056 (vw), 2959 (m), 2944 (m-s), 2925 (m), 2866 (m-s), 2227 (w), 2166 (w), 2148 (w), 1701 (vs), 1593 (m), 1582 (vs), 1510 (m), 1480 (w-m), 1463 (w), 1426 (w), 1373 (w), 1329 (w-m), 1282 (m), 1268 (vs), 1044 (w), 1021 (vw), 997 (w), 985 (w), 952 (vw), 890 (w), 884 (w-m), 819 (w-m), 680 (w-m), 605 (w) cm^{-1}.

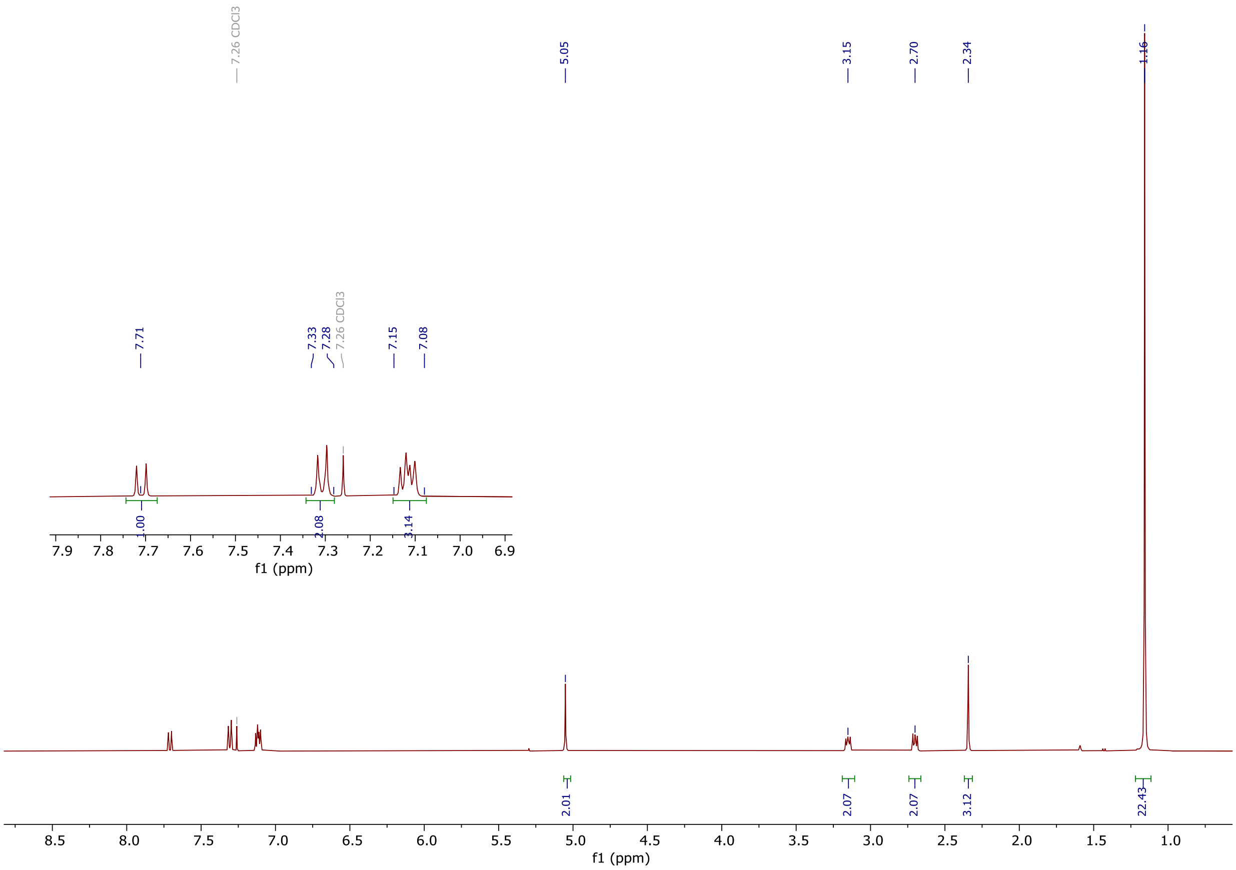

Figure S27: ^{1}H NMR of 45 (400 MHz, 298 K, $CDCl_3$).

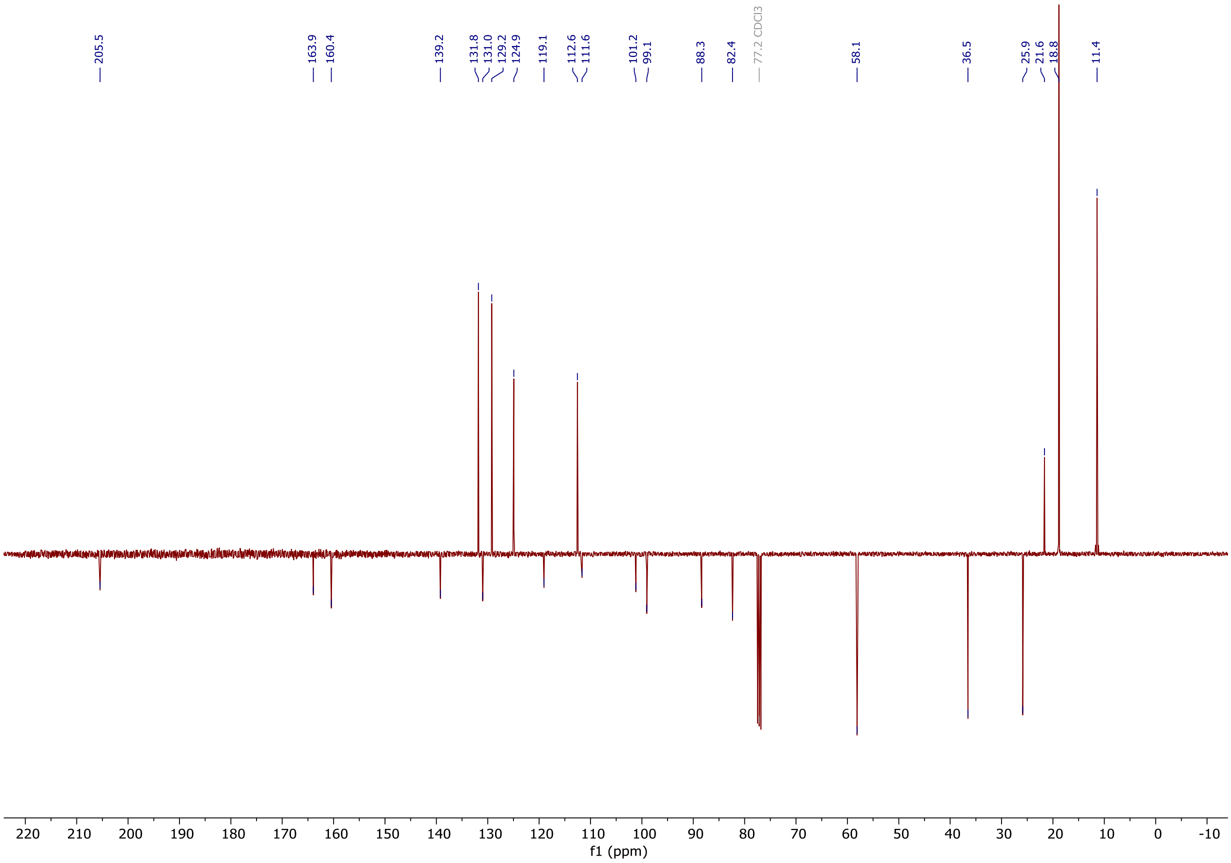

Figure S28: ^{13}C APT NMR of 45 (101 MHz, 298 K, $CDCl_3$).

4-Ethynyl-5-((4-(*p*-tolyl)prop-2-yn-1-yl)oxy)-2,3-dihydro-1*H*-inden-1-one 46

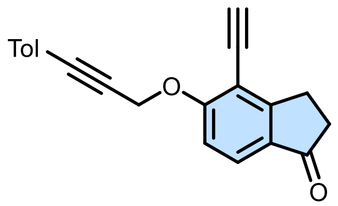

The silylated diyne **45** (1.549 g, 3.4 mmol, 1.0 equiv.) was dissolved in dry THF (20 mL) and cooled to 0 °C. TBAF·$3H_2O$ (1.070 g, 3.4 mmol, 1.0 equiv.) was weighed separately and dissolved in dry THF (10 mL). The solution of TBAF was then added dropwise to the stirred solution of diyne **45**. A color change from colorless to dark red was observed. The reaction was quenched 5 min after the addition was completed by an addition of MeOH (10 mL). The volatiles were removed under reduced pressure and the residue (dark red oil) was purified by column chromatography on silica gel (cyclohexane:EtOAc 1:0 to 4:1) to give **46** (0.958 g, 94%) as a light yellow amorphous solid.

R_f = 0.32 (*n*-hexane:EtOAc 4:1).

^{1}H NMR (400 MHz, 298 K, $CDCl_3$): δ = 7.76 (d, *J* = 8.6 Hz, 1H), 7.33 – 7.27 (m, 2H), 7.19 (d, *J* = 8.6 Hz, 1H), 7.13 – 7.07 (m, 2H), 5.11 (s, 2H), 3.57 (s, 1H), 3.16 (m, 2H), 2.70 (m, 2H), 2.33 (s, 3H).

^{13}C{^{1}H} NMR (101 MHz, 298 K, $CDCl_3$): δ = 205.2, 163.9, 160.7, 139.4, 131.9, 131.1, 129.3, 125.6, 118.9, 112.3, 109.9, 88.7, 86.5, 82.1, 76.5, 58.0, 36.5, 25.7, 21.6.

HRMS (ESI) *m/z*: ($[M+H]^+$) calcd for $C_{21}H_{17}O_2$ 301.1223, found 301.1221 (Δ = -0.55 ppm).

IR ($CHCl_3$): 3304 (m), 3013 (m), 2926 (w), 2870 (w), 2248 (w), 2222 (w), 1704 (vs), 1583 (vs), 1510 (m), 1479 (m), 1453 (w), 1427 (m-w), 1407 (w), 1372 (m-w), 1329 (m-s), 1282 (m), 1267 (vs), 1236 (s), 1065 (s), 1042 (m), 985 (w), 974 (w), 935 (w), 878 (w), 819 (m), 809 (m), 621 (m), 526 (w) cm^{-1}.

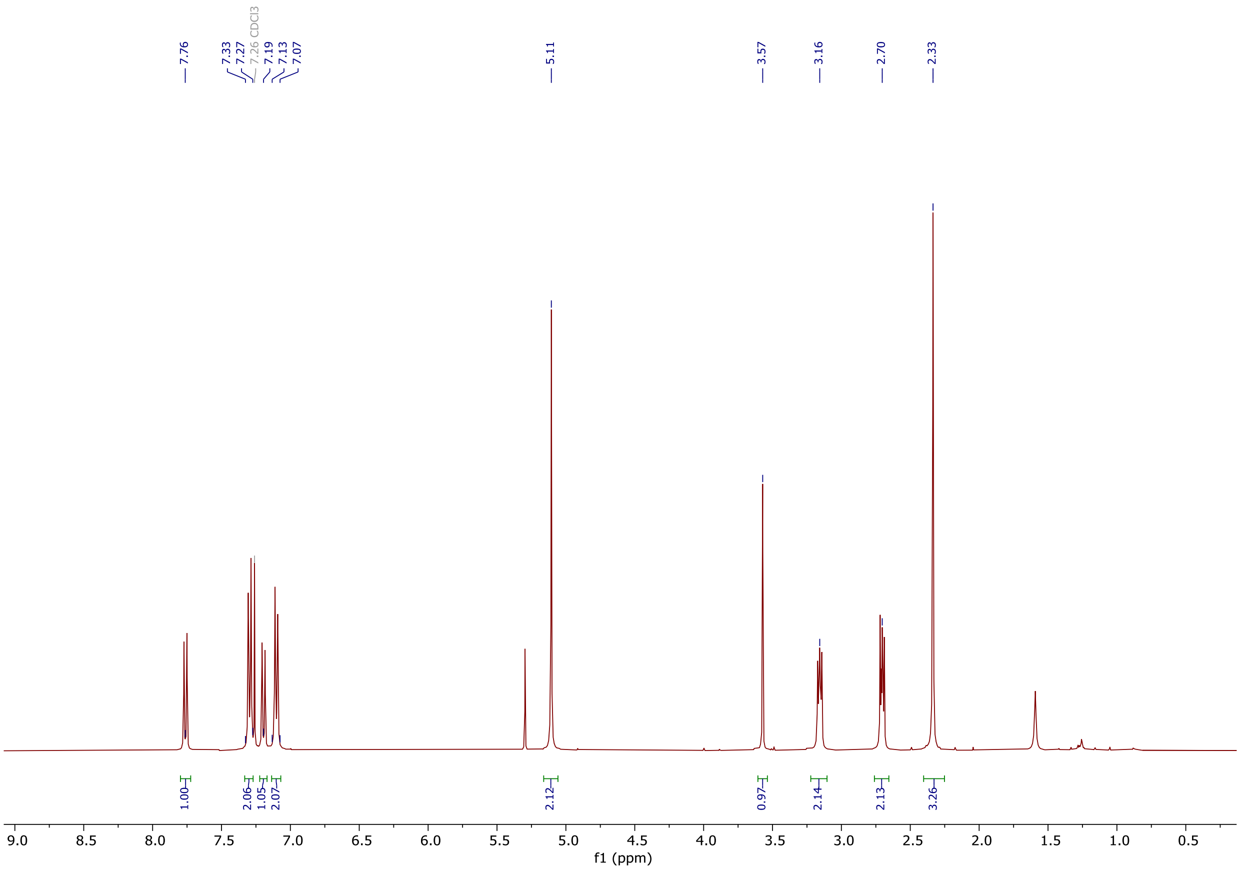

Figure S29: ^{1}H NMR of 46 (400 MHz, 298 K, $CDCl_3$).

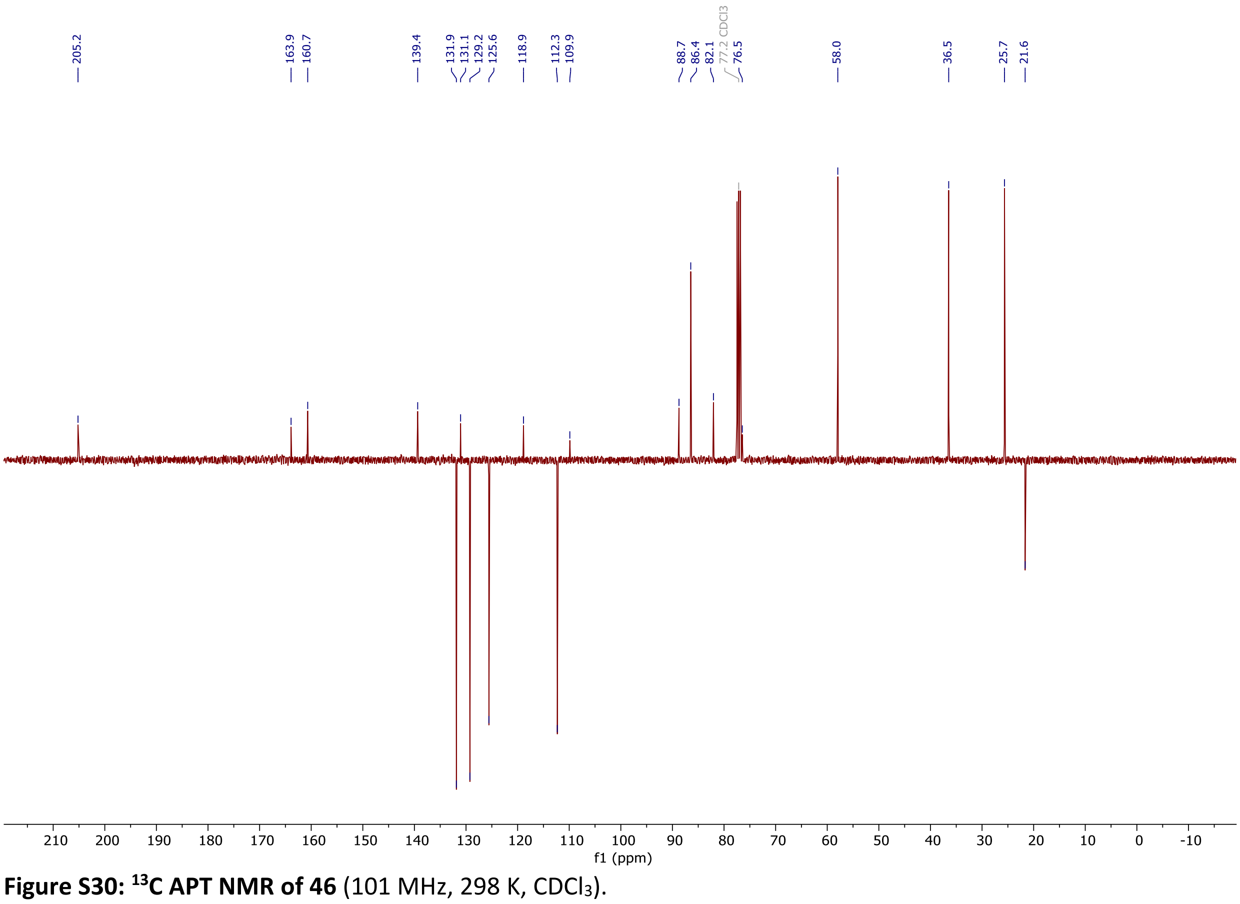

Figure S30: ^{13}C APT NMR of 46 (101 MHz, 298 K, $CDCl_3$).

4-((2-Hydroxyphenyl)ethynyl)-5-((4-(*p*-tolyl)prop-2-yn-1-yl)oxy)-2,3-dihydro-1*H*-inden-1-one 47

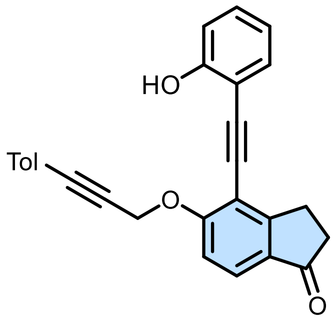

A dried Schlenk flask was charged with 2-iodophenol (1.099 g, 5.0 mmol, 3.0 equiv.), $Pd(PPh_3)_4$ (96 mg, 0.08 mmol, 5 mol%), and CuI (16 mg, 0.08 mmol, 5 mol%), and dissolved in toluene (10 mL) and triethylamine (0.29 mL, 2.1 mmol, 1.25 equiv.) was added. In a separate Schlenk flask, a solution of diyne **46** (0.500 g, 1.7 mmol, 1.0 equiv.) in toluene (10 mL) was prepared. Both solutions were degassed by three freeze-pump-thaw cycles. The Schlenk flask with the reaction was then immersed into a preheated oil bath set to 50 °C and the solution of diyne **46** was added dropwise within 20 min. The reaction mixture was allowed to stir at 50 °C for 40 h. The volatiles were evaporated under reduced pressure. The residue was purified by column chromatography on silica gel (*n*-hexane:DCM:EtOAc 8:0:1 to 8:1:1) to give phenol **47** (297 mg, 46%) as an off white amorphous solid.

$\mathbf{R_f}$ = 0.15 (*n*-hexane:EtOAc 4:1).

^{1}H NMR (400 MHz, 298 K, $CDCl_3$): δ = 7.76 (d, *J* = 8.6 Hz, 1H), 7.42 (dd, *J* = 7.7, 1.6 Hz, 1H), 7.37 – 7.33 (m, 2H), 7.30 (ddd, *J* = 8.3, 7.4, 1.7 Hz, 1H), 7.19 (d, *J* = 8.5 Hz, 1H), 7.14 – 7.09 (m, 2H), 7.01 (dd, *J* = 8.4, 1.1 Hz, 1H), 6.92 (ddd, *J* = 7.6, 7.6, 1.1 Hz, 1H), 6.59 (s, 1H), 5.13 (s, 2H), 3.22 (m, 2H), 2.74 (m, 2H), 2.34 (s, 3H).

^{13}C{^{1}H} NMR (101 MHz, 298 K, $CDCl_3$): δ = 205.1, 162.8, 158.5, 157.4, 139.4, 131.9, 131.2, 131.0, 130.7, 129.2, 125.3, 120.4, 118.8, 114.9, 111.9, 110.6, 109.6, 93.4, 89.8, 89.1, 81.7, 58.2, 36.5, 25.6, 21.7.

HRMS (ESI) *m*/*z*: ($[M+H]^+$) calcd for $C_{27}H_{21}O_3$ 393.1485, found 393.1485 (Δ = -0.15 ppm).

IR (KBr): 3343 (vs), 3082 (vw), 3030 (vw), 2921 (w), 2248 (vw), 2222 (vw), 2209 (vw), 1704 (s), 1691 (vs), 1581 (vs), 1510 (m), 1491 (m), 1475 (w), 1455 (w), 1425 (w), 1376 (w), 1338 (m), 1275 (m), 1254 (m), 1231 (m), 1192 (w), 1180 (w), 1160 (w), 1148 (w), 1098 (w), 1064 (m), 1034 (m), 983 (w), 886 (vw), 816 (m), 754 (m), 683 (w), 531 (w) cm^{-1}.

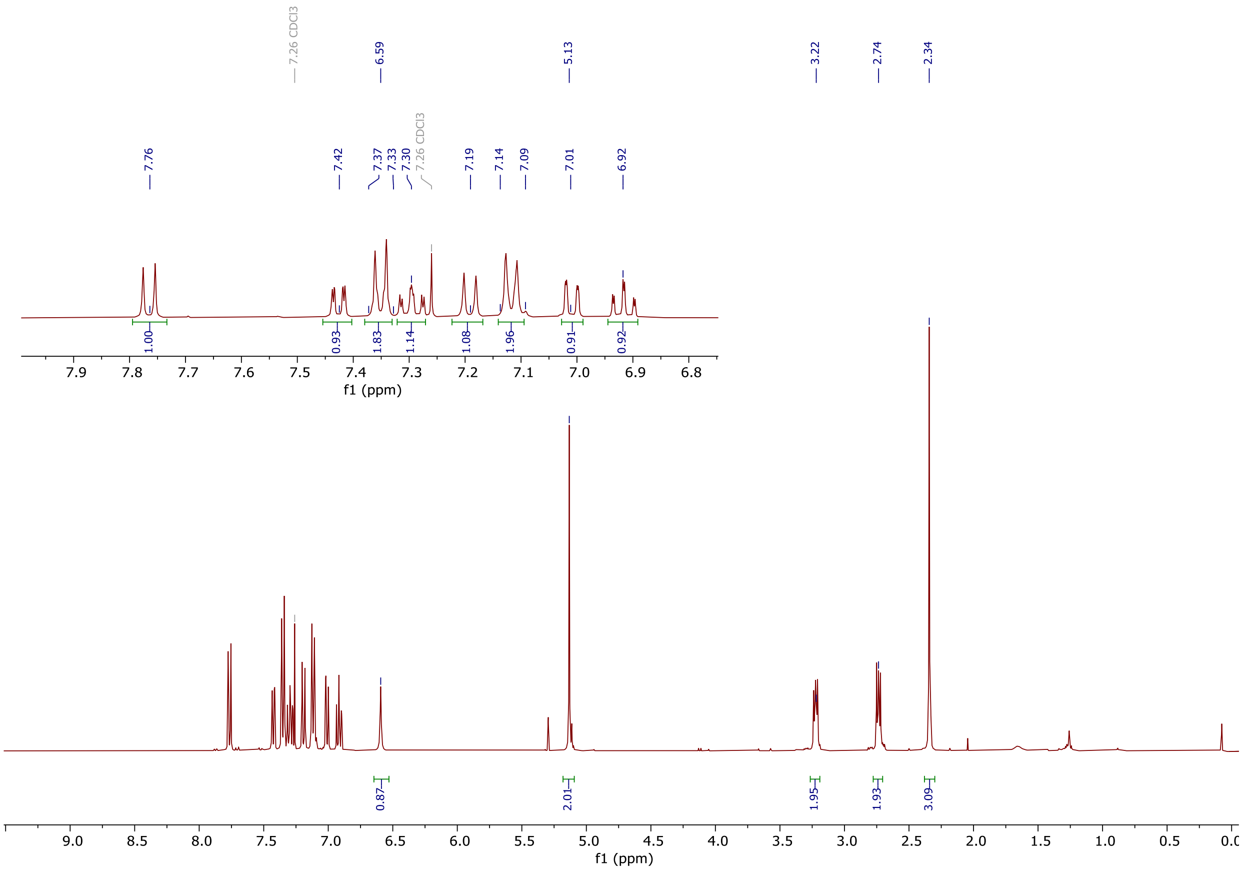

Figure S31: ^{1}H NMR of 47 (400 MHz, 298 K, $CDCl_3$).

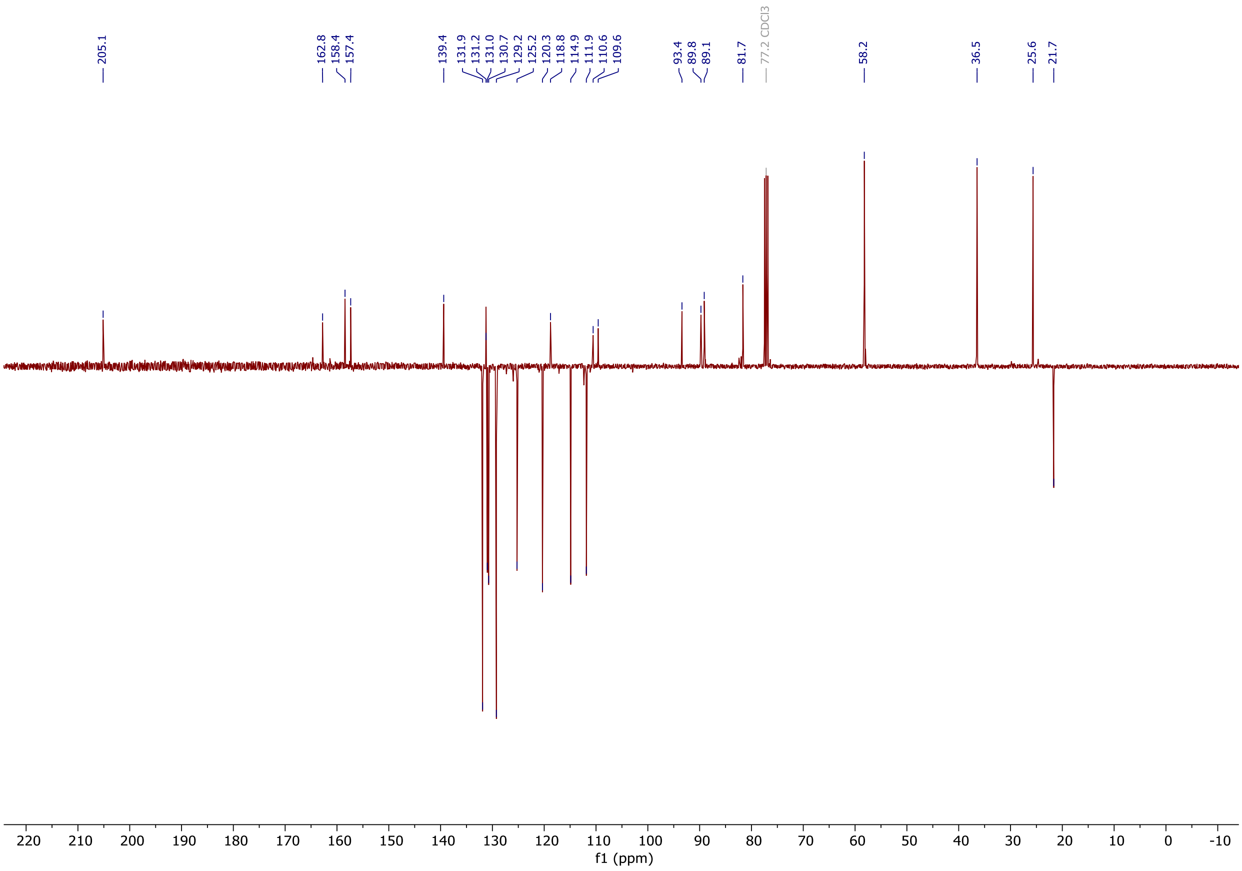

Figure S32: ^{13}C APT NMR of 47 (101 MHz, 298 K, $CDCl_3$).

(-)-5-((4-(*p*-Tolyl)prop-2-yn-1-yl)oxy)-4-((2-(((*R*)-4-(*p*-tolyl)but-3-yn-2-yl)oxy)phenyl)ethynyl)-2,3-dihydro-1*H*-inden-1-one (-)-(*R*)-**48**

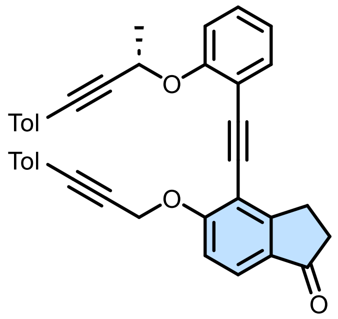

A solution of the phenol **47** (250 mg, 0.64 mmol), (-)-(*S*)-**10** (122.5 mg, 0.77 mmol, 1.2 equiv.), and PPh_3 (183.8 mg, 0.70 mmol, 1.1 equiv.) in benzene (10 mL) under inert atmosphere was inserted into an ice/water bath. Diisopropyl azodicarboxylate (0.15 mL, 155 mg, 0.77 mmol, 1.2 equiv.) was dropwise added and the resulting solution was stirred for 10 min and then warmed to ambient temperature and stirred for additional 4 h. The volatiles were removed under reduced pressure and the residues were purified by column chromatography on silica gel (*n*-hexane:EtOAc 8:1 to 6:1). The product was not completely separated from the reduced diisopropyl azodicarboxylate, thus it was further purified by reversed-phase chromatography (C18, MeOH) to give triyne (-)-(*R*)-**48** (225 mg, 66%) as a yellow foam.

R_f = 0.30 (*n*-hexane:EtOAc 4:1).

$[\alpha]^{20}_D$ -216.4 (*c* 0.325, THF).

^{1}H NMR (400 MHz, 298 K, $CDCl_3$): δ = 7.73 (d, *J* = 8.6 Hz, 1H), 7.57 (dd, *J* = 7.6, 1.7 Hz, 1H), 7.36 – 7.27 (m, 4H), 7.21 (m, 2H), 7.11 – 7.06 (m, 5H), 6.99 (ddd, *J* = 7.5, 7.5, 1.1 Hz, 1H), 5.22 (q, *J* = 6.5 Hz, 1H), 5.14 (s, 2H), 3.28 (m, 2H), 2.69 (m, 2H), 2.33 (s, 3H), 2.32 (s, 3H), 1.82 (d, *J* = 6.5 Hz, 3H).

^{13}C{^{1}H} NMR (101 MHz, 298 K, $CDCl_3$): δ = 205.7, 163.0, 160.3, 158.4, 139.2, 138.8, 133.5, 131.9, 131.7, 131.1, 129.9, 129.23, 129.16, 124.7, 121.6, 119.4, 119.0, 115.2, 114.0, 112.7, 111.8, 95.5, 88.5, 87.6, 86.4, 82.5, 65.9, 58.0, 36.6, 25.9, 22.6, 21.6 (2C).

HRMS (ESI) *m/z*: ($[M+H]^+$) calcd for $C_{38}H_{31}O_3$ 535.2268, found 535.2266 (Δ = -0.36 ppm).

IR ($CHCl_3$): 3055 (w), 3012 (w), 2925 (w), 2869 (w), 2236 (w), 1701 (vs), 1581 (s), 1510 (s), 1495 (m), 1476 (m), 1448 (m), 1427 (w), 1373 (w), 1333 (w-m), 1310 (m), 1278 (m-s), 1234 (s), 1164 (w), 1110 (w), 1086 (w-m), 1063 (m), 1042 (m), 984 (w), 819 (s), 539 (w) cm^{-1}.

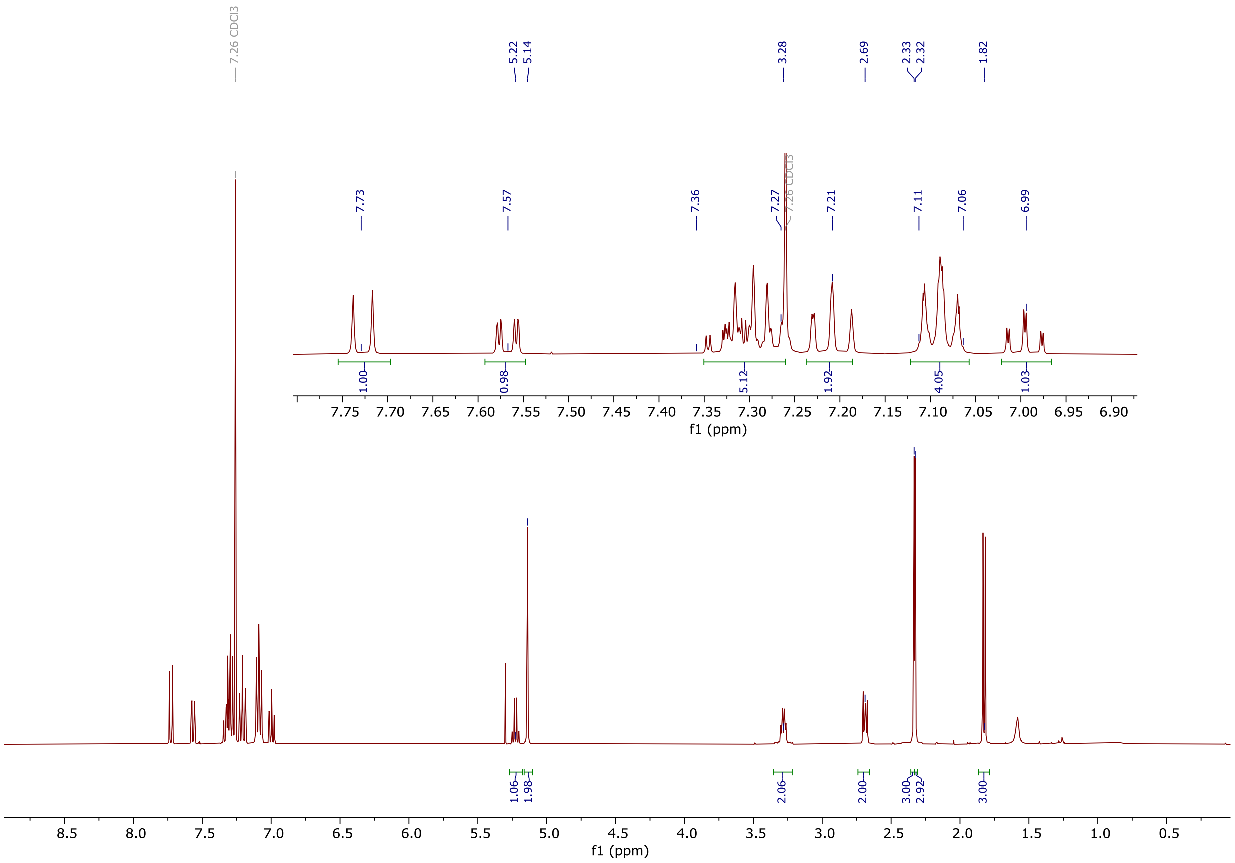

Figure S33: ^{1}H NMR of (-)-(*R*)-**48** (400 MHz, 298 K, $CDCl_3$).

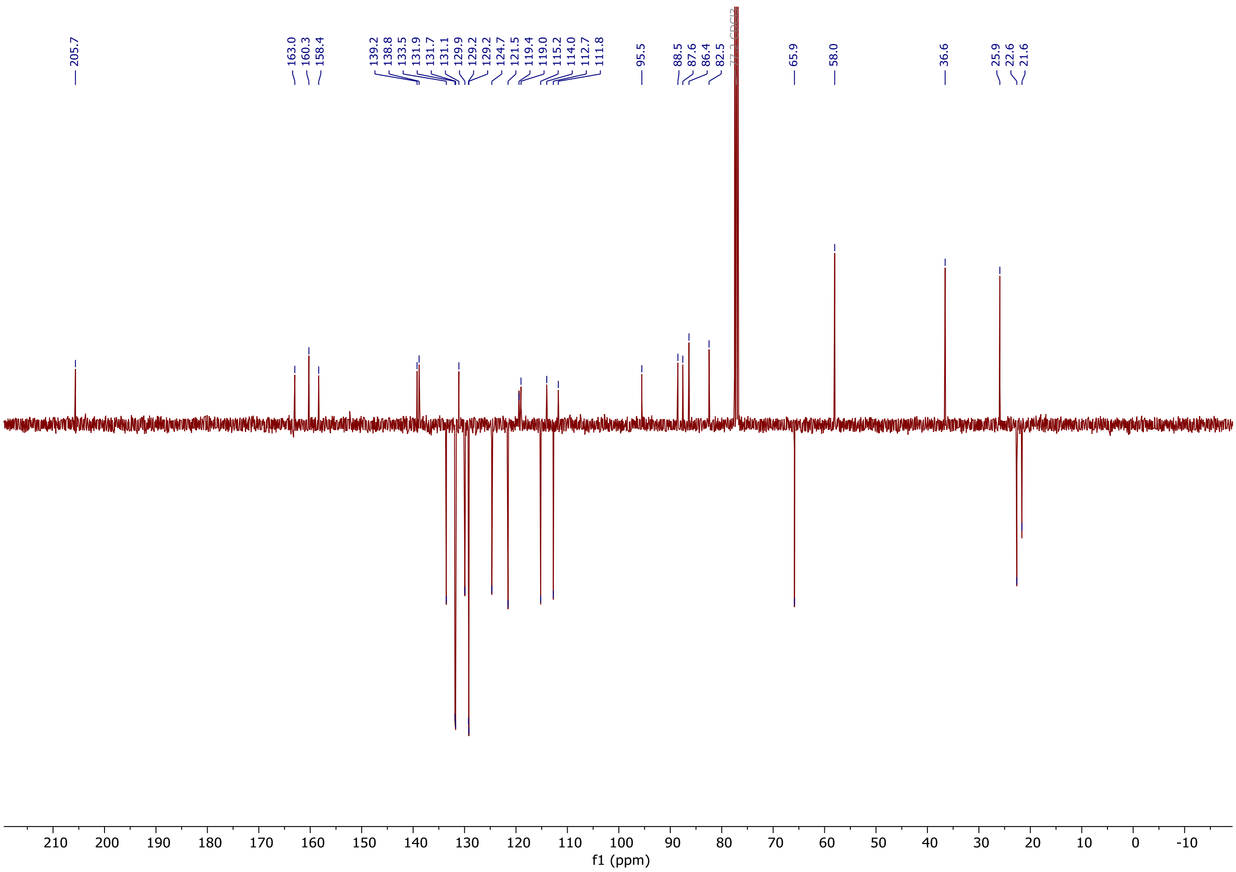

Figure S34: ^{13}C APT NMR of (-)-(*R*)-**48** (101 MHz, 298 K, $CDCl_3$).

Compound (-)-(*M,R*)-**49**

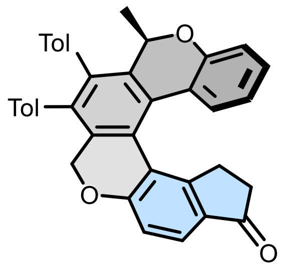

Triyne (-)-(*R*)-**48** (197 mg, 0.37 mmol, 1.0 equiv.) and $Ni(PPh_3)_2(CO)_2$ (47 mg, 0.07 mmol, 20 mol%) were weighed into a Schlenk vessel under inert atmosphere. Toluene (20 mL) was added and the reaction mixture was inserted into a preheated oil bath set to 120 °C. The reaction was stirred at this temperature for 5 min during which a color change from light yellow to black/brown was observed. The reaction mixture was allowed to cool to ambient temperature. The volatiles were removed under reduced pressure and the residue was purified by column chromatography on silica gel (*n*-hexane:EtOAc, 8:1) to give helicene (-)-(*M,R*)-**49** (137 mg, 70%) as a light yellow amorphous solid.

$\mathbf{R_f}$ = 0.37 (*n*-hexane:EtOAc 4:1).

$[\alpha]^{20}_D$ -411 (*c* 0.145, THF).

^{1}H NMR (500 MHz, 298 K, $CDCl_3$): δ = 7.69 (d, *J* = 8.3 Hz, 1H), 7.19 – 7.14 (m, 2H), 7.08 – 6.97 (m, 7H), 6.94 (m, 2H), 6.75 (m, 1H), 6.68 (ddd, *J* = 7.5, 7.5, 1.2 Hz, 1H), 5.19 (q, *J* = 6.7 Hz, 1H), 4.91 (d, *J* = 13.9 Hz, 1H), 4.46 (d, *J* = 13.9 Hz, 1H), 2.48 (ddd, *J* = 17.5, 8.1, 3.4 Hz, 1H), 2.37 (ddd, *J* = 18.5, 8.0, 3.2 Hz, 1H), 2.28 (s, 3H), 2.27 (s, 3H), 2.26 – 2.21 (m, 1H), 1.98 (ddd, *J* = 17.4, 8.1, 3.3 Hz, 1H), 1.13 (d, *J* = 6.7 Hz, 3H).

^{13}C{^{1}H} NMR (126 MHz, 298 K, $CDCl_3$): δ = 206.2, 163.1, 154.7, 153.0, 139.3, 137.5, 137.3, 136.7, 136.64, 136.61, 134.71, 134.67, 132.8, 131.0, 130.0, 129.7, 129.4, 129.1, 128.9, 128.8, 128.7, 128.4, 126.2, 126.0, 125.0, 124.8, 123.7, 122.0, 119.5, 118.1, 73.1, 69.1, 36.9, 26.2, 21.3, 19.3.

HRMS (ESI) *m/z*: ($[M+H]^+$) calcd for $C_{38}H_{31}O_3$ 535.2268, found 535.2266 (Δ = -0.26 ppm).

IR ($CHCl_3$): 3082 (w), 3048 (w), 3010 (w), 2920 (w), 2867 (w), 1696 (vs), 1582 (s), 1517 (w), 1486 (w), 1448 (w), 1426 (w), 1368 (w), 1337 (w), 1250 (s), 1151 (w), 1144 (w), 1139 (w), 1108 (w), 1067 (m), 1053 (w), 1022 (w-m), 956 (w), 840 (w), 821 (w), 530 (w), 512 (w) cm^{-1}.

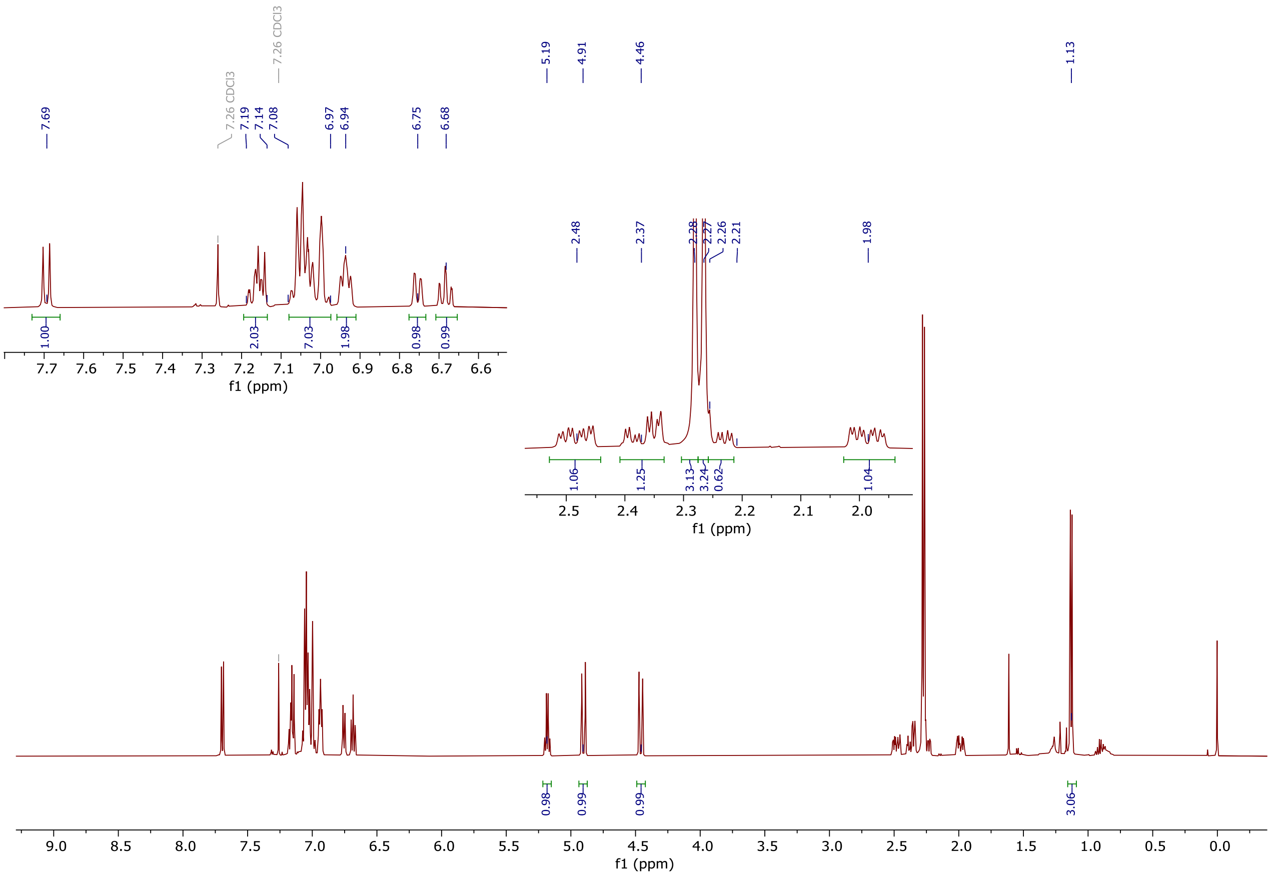

Figure S35: ^{1}H NMR of (-)-(*M,R*)-**49** (500 MHz, 298 K, $CDCl_3$).

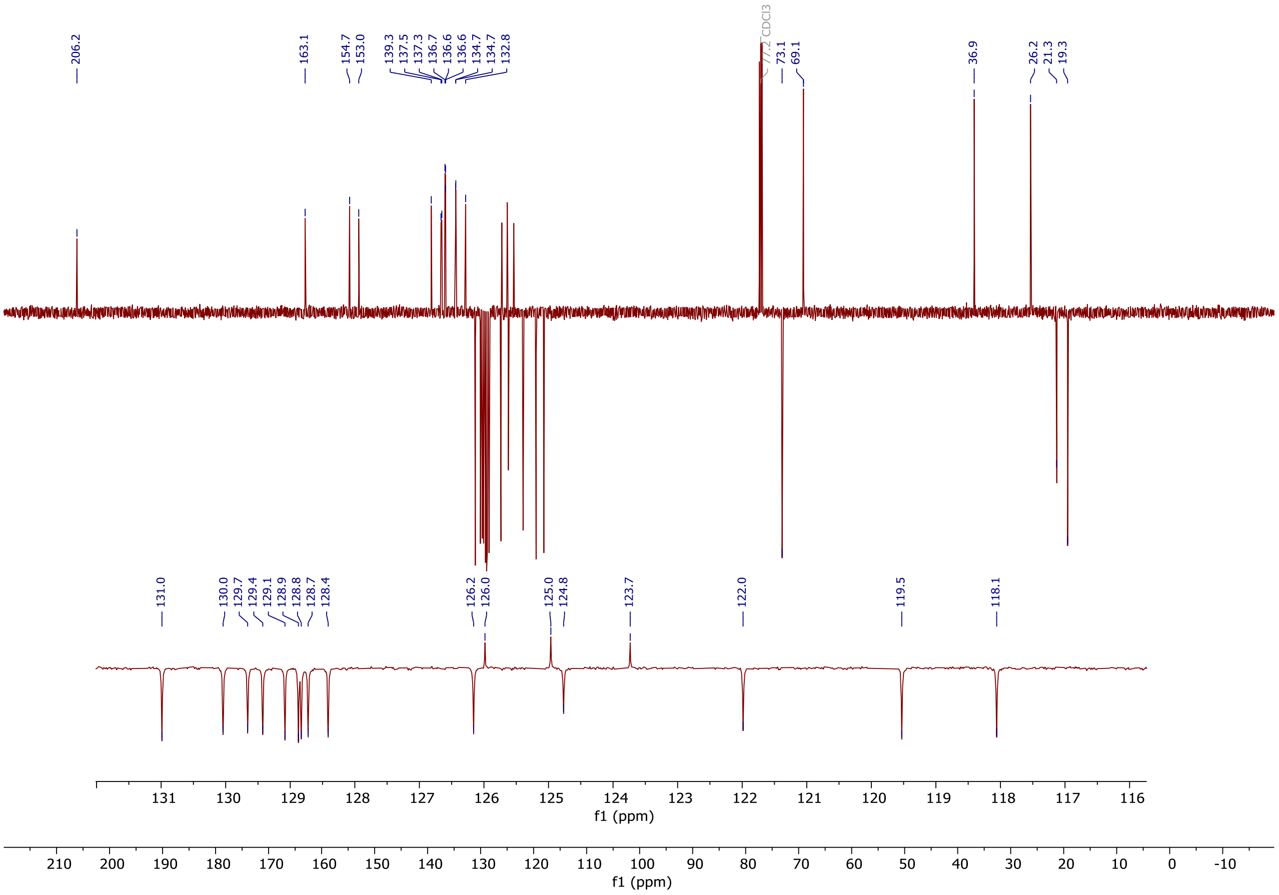

Figure S36: ^{13}C APT NMR of (-)-(*M,R*)-**49** (126 MHz, 298 K, $CDCl_3$).

Oxa[7]helicene (-)-(*M*,*R*,*R*)-**17**

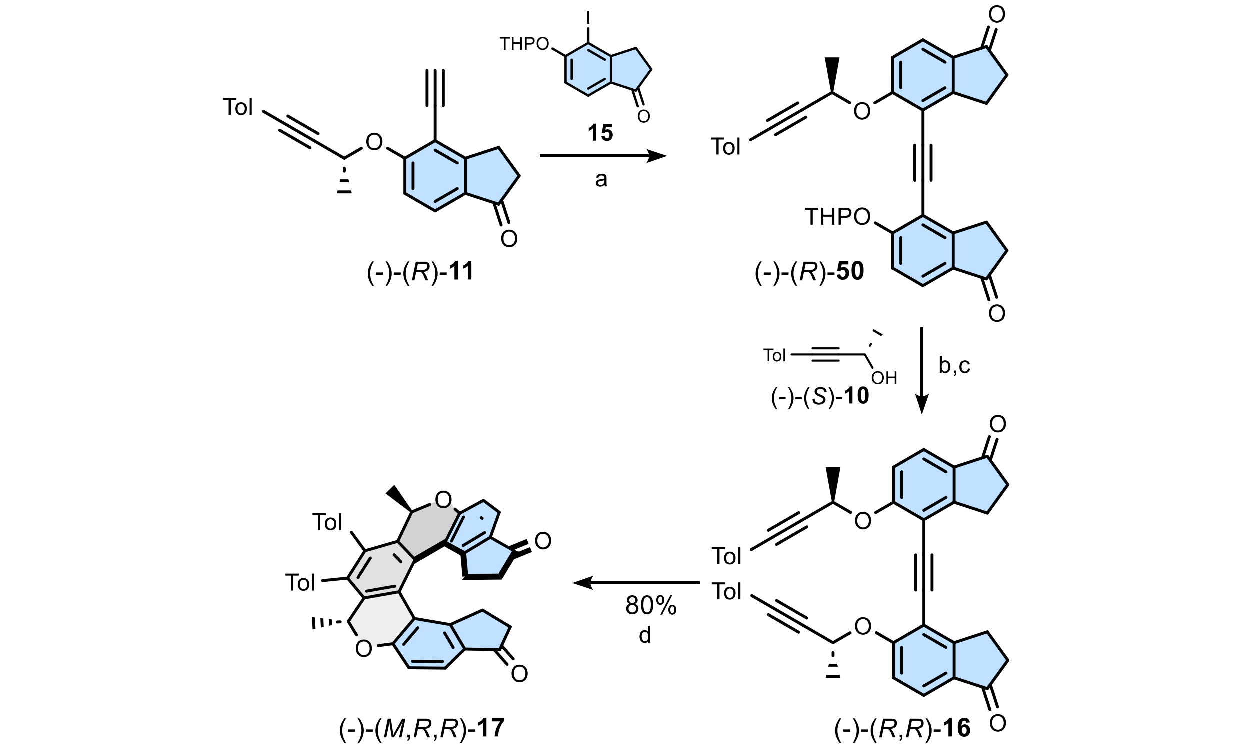

(a) **15** (2.0 equiv.), Pd(PPh$_3$)$_4$ (5 mol%), CuI (10 mol%), toluene-Et$_3$N (3:1), 50 °C, 18 h, 79%; (b) *p*-TsOH (10 mol%), THF-MeOH (1:1), rt, 18 h, 91%; (c) (-)-(*S*)-**10** (1.2 equiv.), PPh$_3$ (1.1 equiv.), DIAD (1.2 equiv.), benzene, 0 °C to rt, 4 h, 86%; (d) CpCo(CO)(fum) (40 mol%), THF, flow reactor, 250 °C, 80 bar, 0.5 mL/min, 16 min residence time, 80%.

4-Iodo-5-((tetrahydro-2*H*-pyran-2-yl)oxy)-2,3-dihydro-1*H*-inden-1-one 15

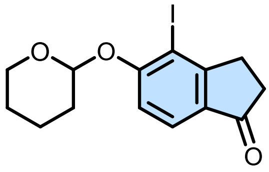

Crude 4-iodo-5-hydroxy-1-indanone (1.00 g, 3.6 mmol, 1.0 equiv.) was prepared following the same procedure as for the preparation of **36**. After the filtration, the crude iodinated product was suspended in a mixture of DCM (20 mL) and 3,4-dihydropyran (0.614 g, 7 mmol, 2.0 equiv.). The pyridinium *p*-toluenesulfonate (92 mg, 0.37 mmol, 10 mol%) was added and the mixture was stirred at room temperature for 21 h. The volatiles were then removed under reduced pressure and the residue was purified by column chromatography on silica gel (*n*-hexane:DCM:EtOAc, 2:2:1) to give product **15** (820 mg, 63%) as a highly viscous colorless oil which crystallized when stored in fridge.

R$_f$ = 0.26 (*n*-hexane:EtOAc 4:1).

Mp: 46-48 °C (*n*-hexane-DCM-EtOAc at 3 °C).

^{1}H NMR (400 MHz, 298 K, CDCl$_3$): δ = 7.60 (d, *J* = 8.4 Hz, 1H), 7.02 (d, *J* = 8.4 Hz, 1H), 5.62 (t, *J* = 2.8 Hz, 1H), 3.75 (td, *J* = 11.2, 3.0 Hz, 1H), 3.61 – 3.55 (m, 1H), 2.94 – 2.88 (m, 2H), 2.67 – 2.60 (m, 2H), 2.13 (dtt, *J* = 12.5, 11.3, 3.4 Hz, 1H), 2.01 – 1.93 (m, 1H), 1.85 (dddd, *J* = 13.6, 12.1, 4.3, 3.1 Hz, 1H), 1.75 – 1.65 (m, 2H), 1.64 – 1.56 (m, 1H).

^{13}C{^{1}H} NMR (101 MHz, 298 K, CDCl$_3$): δ = 205.5, 161.4, 160.9, 132.7, 125.1, 114.1, 96.9, 88.1, 62.0, 36.8, 31.5, 30.1, 25.2, 18.2.

HRMS (ESI) *m/z*: ([M+Na]$^+$) calcd for $C_{14}H_{15}O_3INa$ 380.9958, found 380.9957 (Δ = -0.18 ppm).

IR (ATR): 3062 (w), 3038 (vw), 2953 (m), 2919 (m), 2919 (w), 2875 (w), 1709 (vs), 1584 (s), 1559 (m), 1460 (m), 1452 (m), 1415 (w), 1392 (w), 1354 (m), 1313 (m), 1289 (m), 1276 (m), 1250 (s), 1235 (s), 1202 (m-s), 1177 (m), 1106 (vs), 1050 (w), 1036 (m), 1036 (m-s), 1021 (s), 969 (s), 938 (w), 895 (s), 868 (s), 849 (m), 818 (s), 790 (m), 760 (m), 718 (w), 657 (m), 616 (w), 603 (w), 578 (m), 544 (w) cm^{-1}.

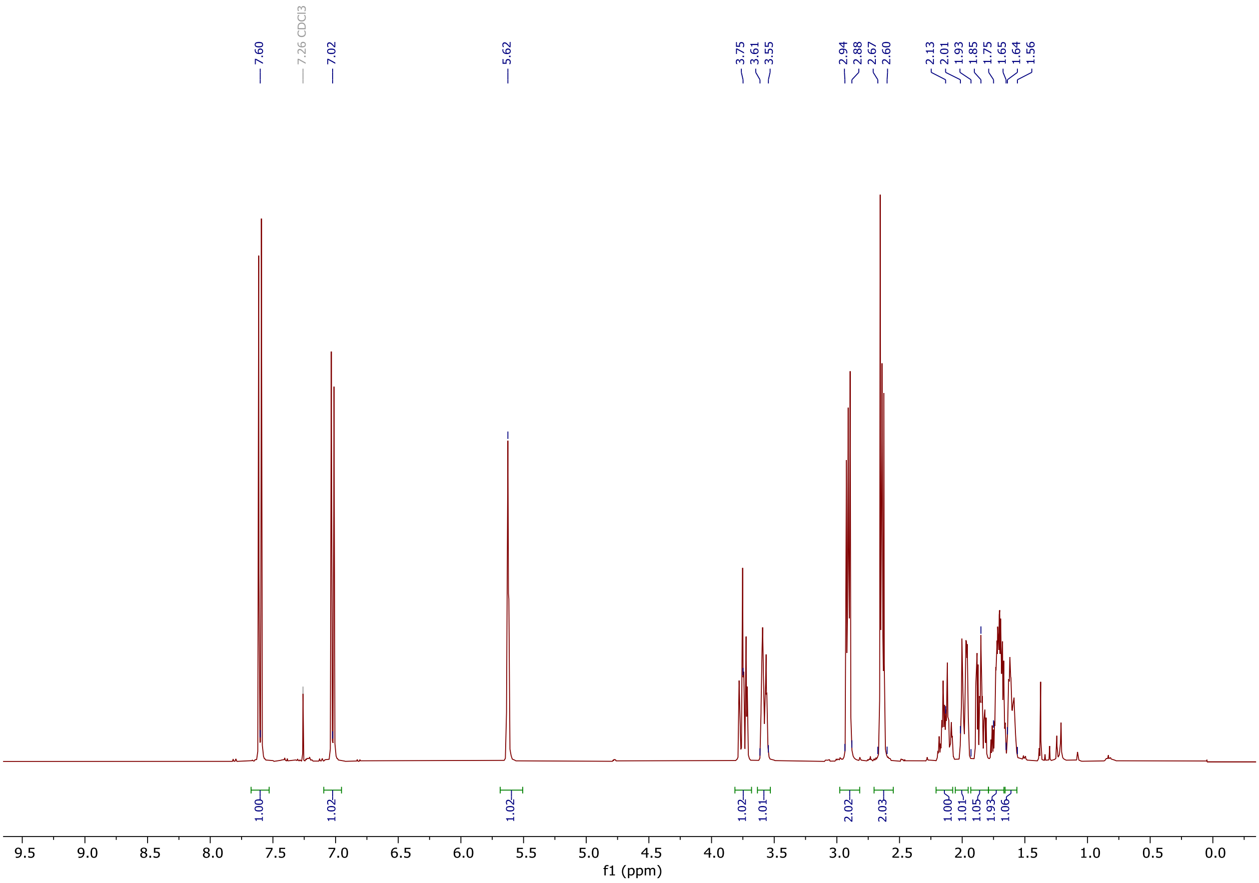

Figure S37: ¹H NMR of 15 (400 MHz, 298 K, $CDCl_3$).

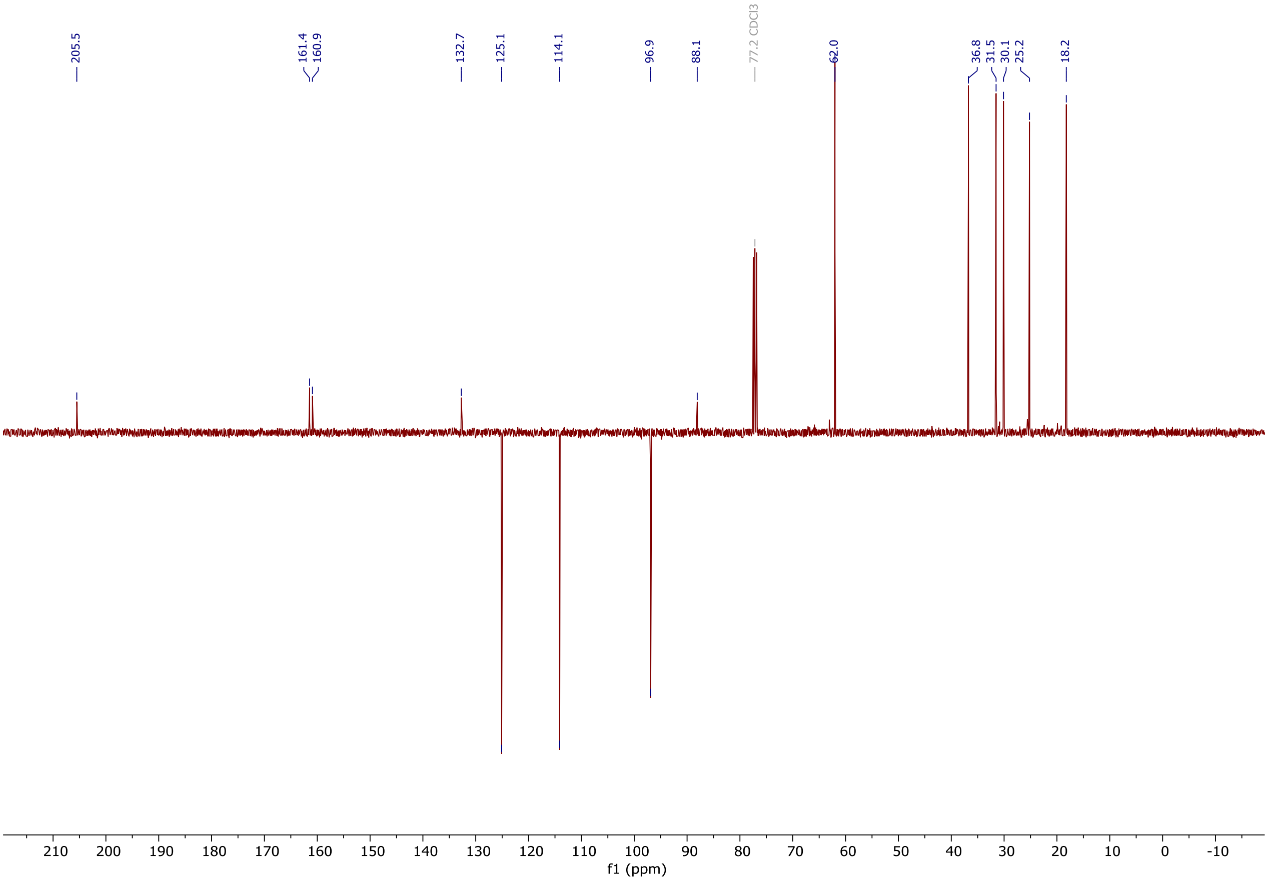

Figure S38: ¹³C APT NMR of 15 (101 MHz, 298 K, $CDCl_3$).

Compound (-)-(R)-**50**

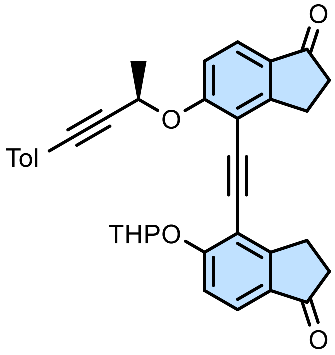

The iodide **15** (2.165 g, 6 mmol, 2 equiv.), $Pd(PPh_3)_4$ (175 mg, 0.15 mmol, 5 mol%) and CuI (58 mg, 0.3 mmol, 10 mol%) were combined in a dried Schlenk and an inert atmosphere was established. Then 10 ml of toluene and 5 mL of triethylamine were added. Diyne (-)-(*R*)-**11** (950 mg, 3 mmol, 1.0 equiv.) was weighed separately into a second Schlenk and dissolved in 5 mL of toluene. Both solutions were degassed by 3 freeze-pump-thaw cycles. The reaction Schlenk was inserted into a preheated oil bath set to 50 °C and the solution of diyne was slowly added by syringe (20 minutes). The reaction was allowed to stir overnight (18h). The solvent was evaporated and the residue was purified by column chromatography (SiO_2, Cyn-hex:EtOAc, 1:1) to give (-)-(R)-**50** as 1.311 g (79%) of an orange amorphous solid.

Rf SiO_2, n-hex:EtOAc, 1:1, Rf = 0.38

OR $[\alpha]^{20}_{D}$ -211.6 (*c* 0.276, THF)

^{1}H NMR (401 MHz, 298 K, $CDCl_3$) δ = 7.75 (d, *J* = 8.6 Hz, 1H), 7.70 (d, *J* = 8.6 Hz, 1H), 7.29 – 7.21 (m, 4H), 7.11 – 7.05 (m, 2H), 5.70 – 5.64 (m, 1H), 5.30 (q, *J* = 6.5 Hz, 1H), 3.91 (td, *J* = 10.9, 2.9 Hz, 2H), 3.72– 3.60 (m, 1H), 3.33– 3.18 (m, 4H), 2.77 – 2.65 (m, 4H), 2.32 (s, 3H), 2.23 – 2.11 (m, 1H), 2.08 – 1.90 (m, 2H), 1.85 (d, *J* = 6.4 Hz, 3H), 1.80 – 1.57 (m, 3H).

^{13}C{^{1}H} NMR (101 MHz, 298 K, $CDCl_3$) δ = 205.4, 205.2, 163.0, 162.4, 160.0, 159.6, 139.2, 131.7 (2C), 131.1, 130.8, 129.2 (2C), 125.2, 125.0, 118.9, 114.6, 113.4, 111.5, 111.4, 96.9, 91.6, 91.1, 87.0, 86.4, 65.9, 62.2, 36.4 (2C), 30.3, 26.0, 25.9, 25.2, 22.5, 21.6, 18.6.

HRMS (ESI) *m/z*: $[M + Na]^+$ calcd for $C_{36}H_{32}O_5Na$ 567.2142, found 567.2134 (Δ = -1.44 ppm).

IR ($CHCl_3$) 2949 (w), 2926 (w), 2881 (w), 2873 (w), 2855 (w), 2242 (vw), 2227 (w), 1699 (vs), 1609 (w), 1584 (s), 1510 (m), 1473 (w), 1442 (w), 1406 (vw), 1375 (w), 1324 (m), 1306 (s), 1271 (s), 1257 (s), 1243 (m), 1184 (w), 1120 (m), 1100 (w), 1085 (m), 1071 (s), 1038 (m), 1021 (m), 873 (w), 819 (m), 543 (vw)

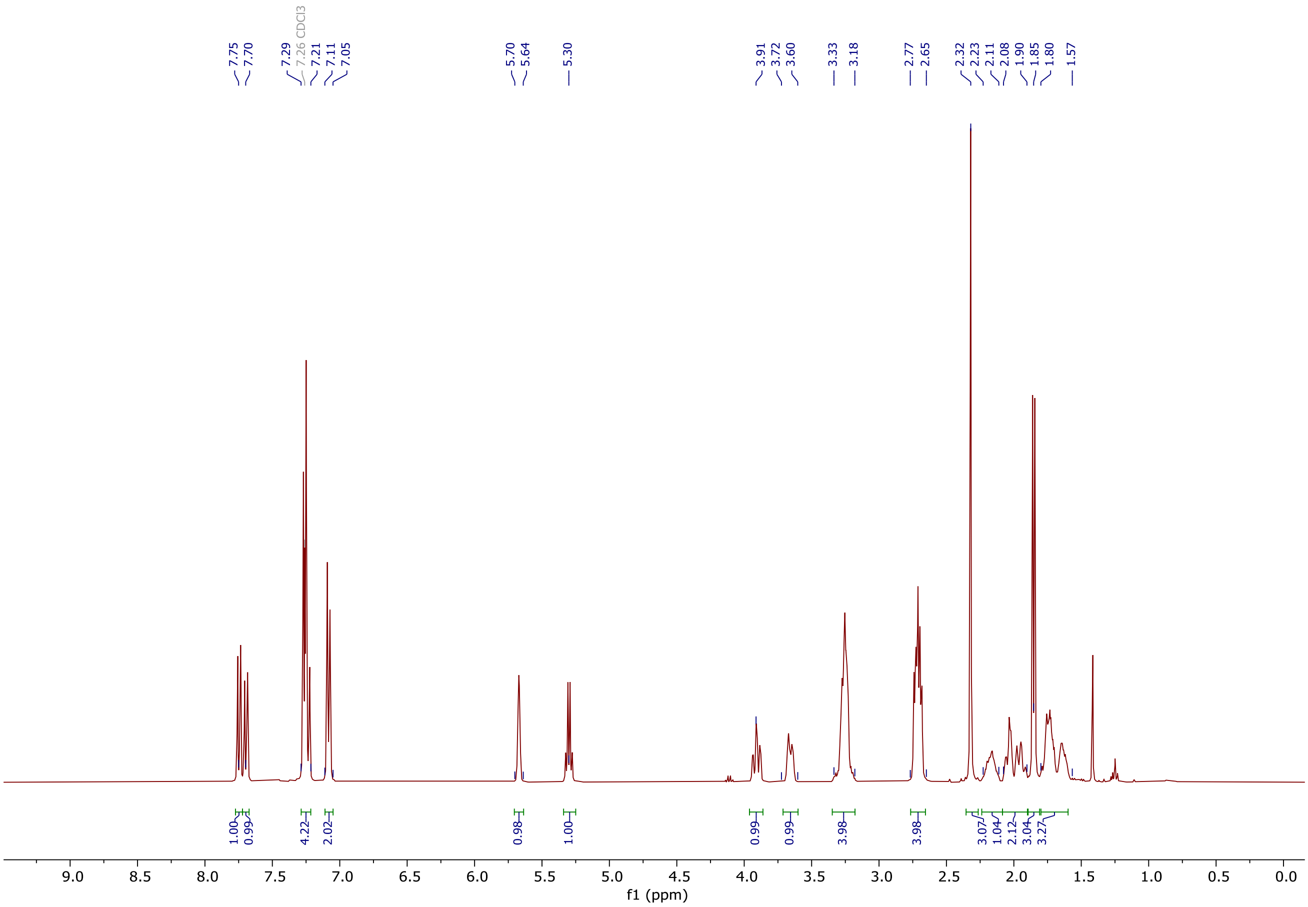

Figure S39: ^{1}H NMR of (-)-(R)-**50** (401 MHz, 298 K, $CDCl_3$)

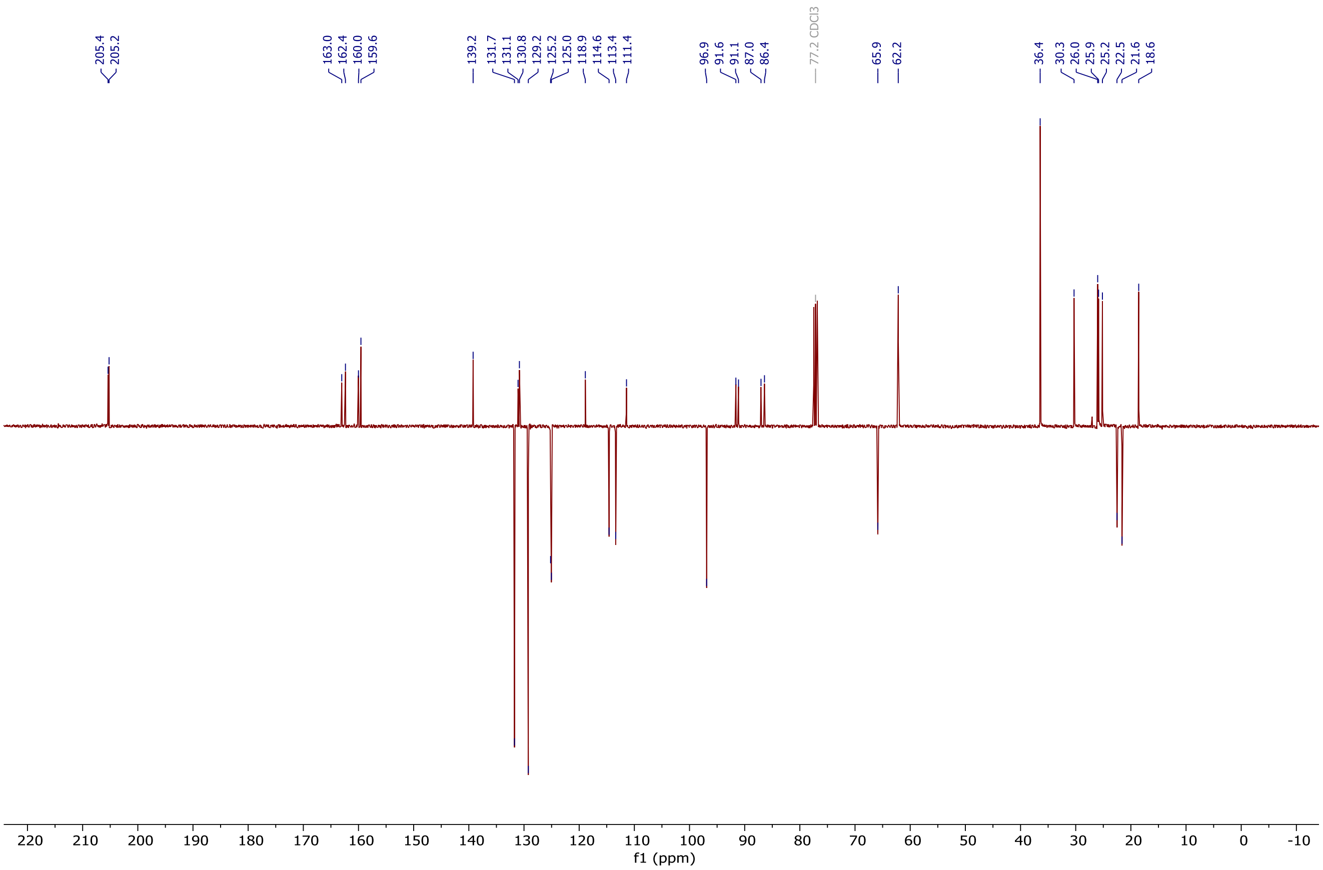

Figure S40: ^{13}C APT NMR of (-)-(R)-**50** (101 MHz, 298 K, $CDCl_3$)

Compound (-)-(*R,R*)-**16**

Prepared in two steps:

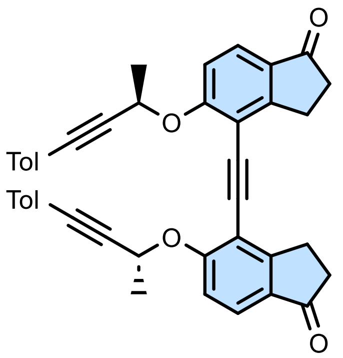

The THP-protected diyne (-)-(*R*)-**50** (2.100 g, 3.9 mmol, 1.0 equiv.) was dissolved in THF/MeOH 1:1 (80 mL total) and pTsOH·H_2O (73 mg, 0.4 mmol, 10 mol%) was added. The mixture was allowed to stir overnight (18 h), during which a white precipitate formed. The reaction mixture was subjected to centrifugation (2300 rpm, 120 s). The supernatant was removed and the solid was washed with diethyl ether and centrifuged again. The supernatant was removed and the solid residue was dried under vacuum to give 1.611 g (91%) of a white insoluble powder, which was assumed to be the pure product of the deprotection and was used without further purification for the next step.

To the suspension of the product of deprotection (1.550 g, 3.4 mmol, 1.0 equiv.), (-)-(S)-**10** (647 mg, 4 mmol, 1.2 equiv.) and PPh_3 (971 mg, 3.7 mmol, 1.1 equiv.) in dry benzene (50 mL) was inserted into an ice bath and DIAD (0.8 mL, 0.820 mg, 4 mmol, 1.2 equiv.) was added dropwise. The resulting yellow solution was allowed to stir at room temperature for 4 h. The volatiles were removed under reduced pressure and the residue was subjected to column chromatography (SiO_2, *n*-hex:EtOAc, 4:1) to give light yellow solid containing residual reduced DIAD. The solid was reprecipitated from Cy/EtOAc (4:1) and filtered on a frit (ultasonication of the suspension resulted in the formation of easy-to-filter material). The precipitation was performed twice, affording 1.744 g (86%) of a white amorphous solid.

Rf SiO_2, n-hex:EtOAc, 4:1, Rf = 0.1

OR $[\alpha]^{20}_{D}$ -404.2 (*c* 0.325, THF)

^{1}H NMR (400 MHz, 298 K, $CDCl_3$) δ = 7.75 (d, *J* = 8.6 Hz, 2H), 7.30 – 7.23 (m, 6H), 7.09 (m, 4H), 5.30 (q, *J* = 6.6 Hz, 2H), 3.28 (m, 4H), 2.71 (m, 4H), 2.32 (s, 6H), 1.89 (d, *J* = 6.5 Hz, 6H).

^{13}C{^{1}H} NMR (101 MHz, 298 K, $CDCl_3$) δ = 205.4 (2C), 163.0 (2C), 159.9 (2C), 139.2 (2C), 131.8 (4C), 130.9 (2C), 129.2 (4C), 125.1 (2C), 119.0 (2C), 113.6 (2C), 111.6 (2C), 91.6 (2C), 87.1 (2C), 86.5 (2C), 66.0 (2C), 36.5 (2C), 25.9 (2C), 22.6 (2C), 21.6 (2C).

HRMS (ESI) *m/z*: $[M + H]^+$ calcd for $C_{42}H_{35}O_4$ 603.2530, found 603.2525 (Δ = -0.87 ppm).

IR ($CHCl_3$) 2242 (vw), 2227 (w), 2938 (w), 2925 (w), 1699 (vs), 1610 (s), 1584 (s), 1510 (m), 1473 (w), 1407 (vw), 1375 (w), 1324 (m), 1306 (s), 1271 (s), 1257 (s), 1243 (m), 1180 (vw), 1120 (w), 1085 (s), 1041 (m), 1021 (w), 819 (m), 545 (vw).

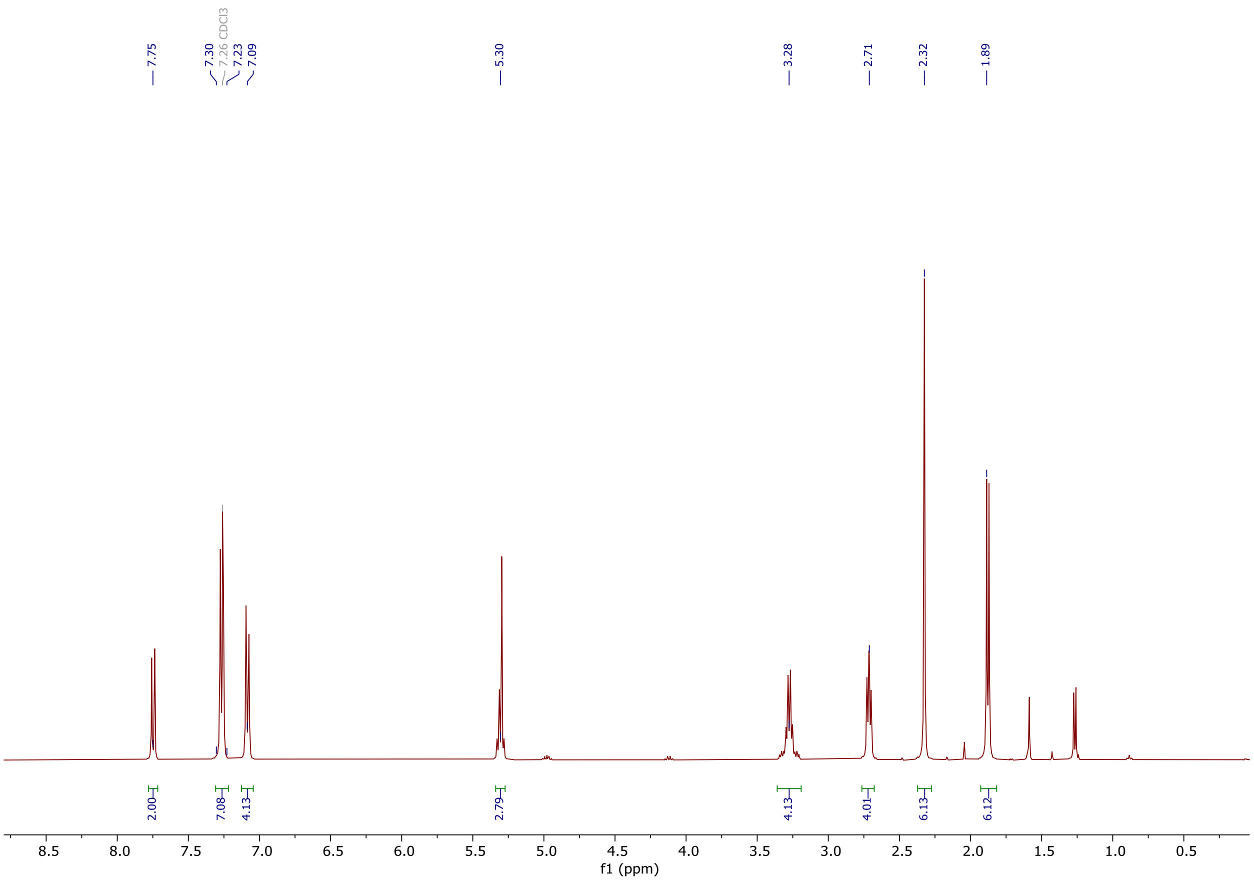

Figure S41: ¹H NMR of (-)-(*R,R*)-**16** (400 MHz, 298 K, $CDCl_3$)

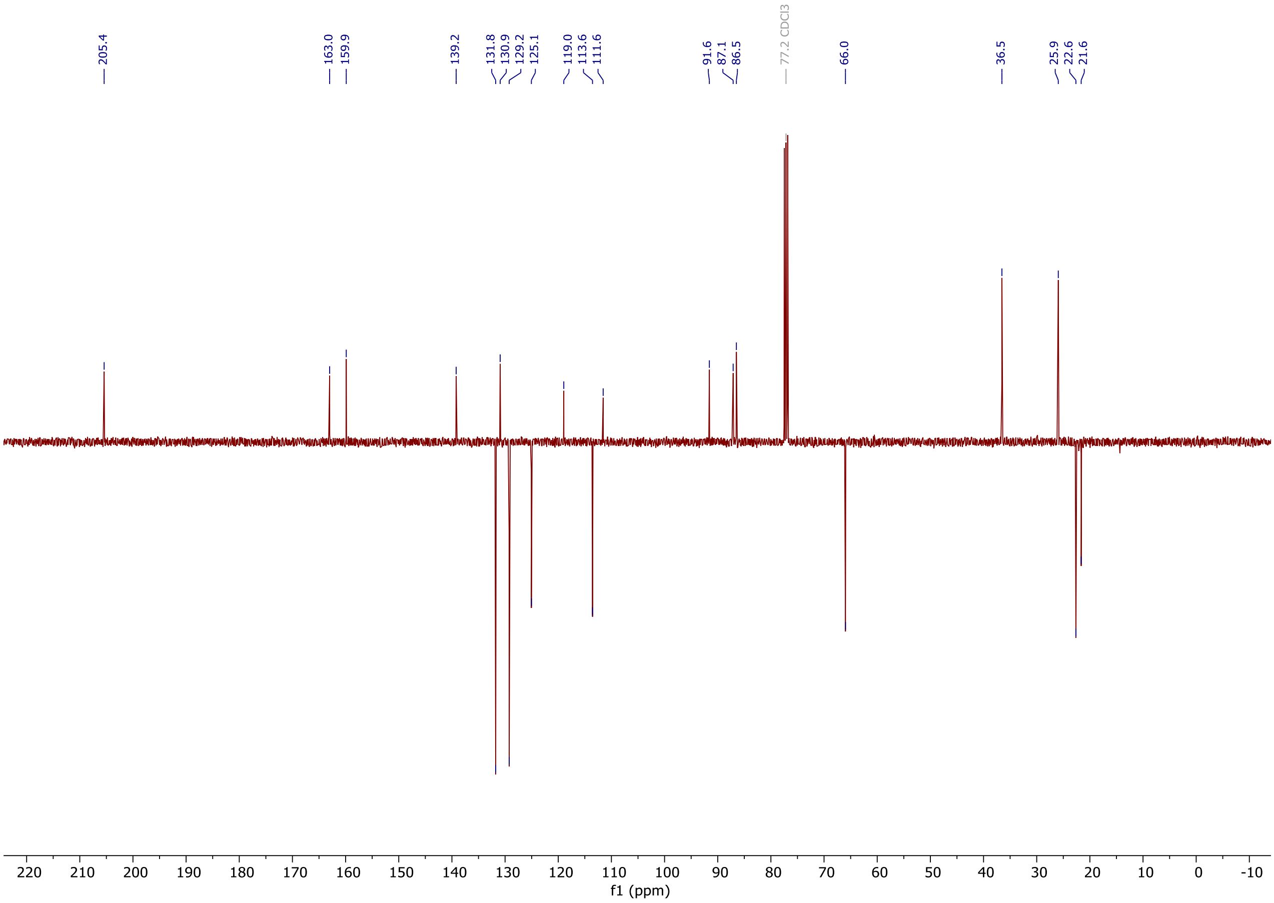

Figure S42: ¹³C APT NMR of (-)-(*R,R*)-**16** (101 MHz, 298 K, $CDCl_3$)

Compound (-)-(*M*,*R*,*R*)-**17**

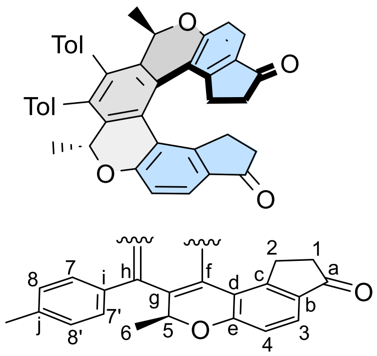

The triyne (-)-(*R*,*R*)-**16** (2.400 g, 4 mmol, 1.0 equiv.) and CpCo(CO)(fum) (473 mg, 1.6 mmol, 40 mol%) were weighed into a Schlenk vessel and inert atmosphere was established. Dry THF (40 mL) was added and the solution was sonicated until all material dissolved. The solution was fed into a flow reactor (setup as in lit.[5], 250 °C, 80 bar, 0.5 mL/min, 16 min residence time). The solvents were removed under reduced pressure and the residue was purified by column chromatography (SiO_2, *n*-hex:EtOAc, 1:6 -> 1:1) to give 1.923 mg (80 %) of (-)-(*M*,*R*,*R*)-**17** as a light yellow solid.

Rf SiO_2, n-hex:EtOAc, 4:1, Rf = 0.12

OR $[\alpha]^{20}_{D}$ -488.9 (*c* 0.317, THF)

^{1}H NMR (400 MHz, 298 K, $CDCl_3$) δ = 7.68 (d, *J* = 8.2 Hz, 2H, CH^3), 7.20 – 7.09 (m, 6H, overlapping of CH^4 and $CH^{8/8'}$), 6.91 (m, 2H, $CH^{7/7'}$), 6.71 (dd, *J* = 7.7, 1.8 Hz, 2H, $CH^{7/7'}$), 5.39 (q, *J* = 6.7 Hz, 2H, CH^5), 2.45 (ddd, *J* = 17.2, 8.0, 2.8 Hz, 2H, $CH_2{}^2$), 2.39 – 2.18 (m, 10H, overlapping of $CH_3{}^9$ and $CH_2{}^1$), 1.81 (ddd, *J* = 17.3, 7.9, 4.0 Hz, 2H, $CH_2{}^2$), 0.97 (d, *J* = 6.7 Hz, 6H, $CH_3{}^6$).

^{13}C{^{1}H} NMR (101 MHz, 298 K, $CDCl_3$) δ = 205.2 (C^a), 159.6 (C^e), 152.9 (C^c), 139.8 (C^g), 137.3 (C^f), 136.7 (C^j), 134.3 (C^i), 133.0 (C^d), 130.7 ($CH^{7/7'}$), 129.0, 128.9, 128.7 ($CH^{7/7'/8/8'}$), 125.3 (CH^3), 124.7 (C^h), 124.6(C^b), 120.0 (CH^4), 74.2 (CH^5), 37.0 ($CH_2{}^1$), 26.0 ($CH_2{}^2$), 21.3 ($CH_3{}^9$), 18.4 ($CH_3{}^6$).

HRMS (APCI) *m/z*: $[M + H]^+$ calcd for $C_{42}H_{35}O_4$ 603.2530, found 603.2528 (Δ = -0.34 ppm).

IR (ATR) 2988 (vw), 2935 (vw), 2917 (vw), 1712 (vs), 1577 (s), 1548 (w), 1442 (w), 1425 (w), 1381 (w), 1363 (w), 1326 (m), 1280 (w), 1251 (s), 1225 (s), 1180 (w), 1151 (m), 1107 (w), 1061 (s), 1019 (m), 988 (w), 968 (w), 957 (m), 905 (w), 879 (w), 845 (w). 819 (m), 804 (m), 862 (w), 738 (m), 686 (w), 676 (w), 662 (w), 616 (w), 602 (w) 534 (m), 513 (m-s).

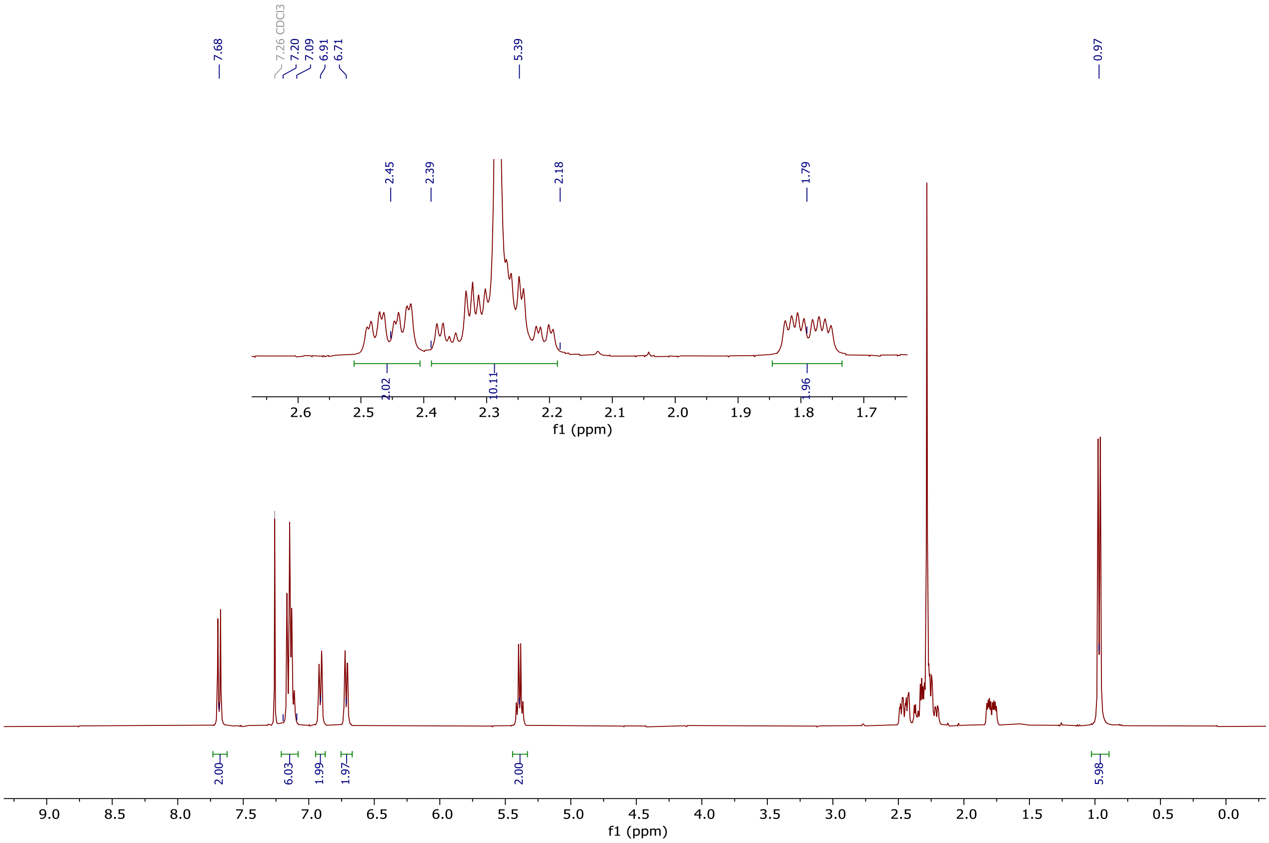

Figure S43: ^{1}H NMR of (-)-(*M*,*R*,*R*)-**17** (400 MHz, 298 K, $CDCl_3$)

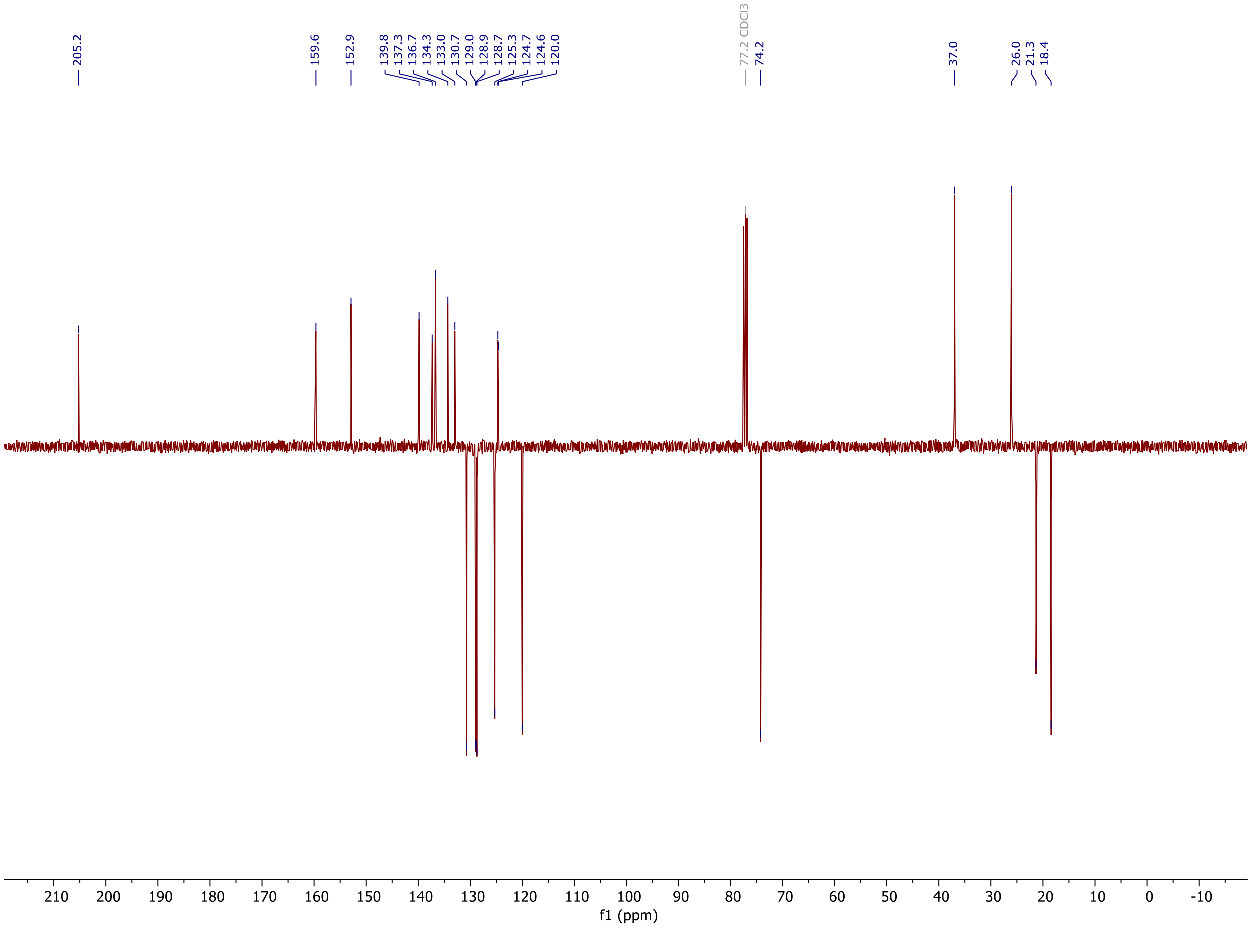

Figure S44: ^{13}C APT NMR of (-)-(*M*,*R*,*R*)-**17** (101 MHz, 298 K, $CDCl_3$)

Synthesis of proligands

General procedures

General procedure for the preparation of alkyl and aryl-substituted helicene proligands (GP1)

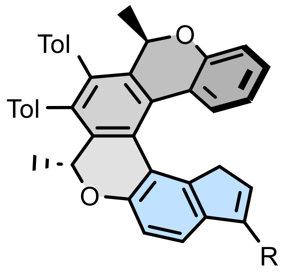

The starting indanone helicene (1.0 equiv.) was weighed into a small dry Schlenk vessel under inert atmosphere. THF (10 mL/mmol) was added and the solution was cooled to -80 °C by isopropanol/liquid N_2 bath. The solution of the appropriate Grignard reagent (1.5 equiv.) was added dropwise and the reaction mixture was allowed to warm to ambient temperature and stirred for 1 h. The reaction was quenched by an addition of a diluted solution of NH_4Cl (approx. 5 mL of saturated solution + 5 mL of water per mmol). The reaction mixture was diluted with EtOAc (40 mL), washed with water (2x20 mL), and brine (20 mL). The organic phase was dried over anhydrous $MgSO_4$ and volatiles were removed under reduced pressure. The residue was purified by passing through a short silica gel column with the indicated solvent. The TLC of fractions collected was visualized using vanillin stain and products staining blue/green were collected. The intermediate alcohol was isolated as a mixture of diastereoisomers. The mixture was immediately used for the next step assuming a 100% yield. The intermediate diastereomeric mixture of alcohols was dissolved in toluene (20 mL/mmol) and transferred into a small Schlenk vessel. *p*-Toluenesulfonic acid monohydrate (20 mol%) was added and the reaction mixture was inserted into a preheated oil bath set to 110 °C. The reaction was stirred for 60 s and then immediately cooled to ambient temperature, diluted with EtOAc (30 mL), washed with water (3x20 mL) and brine (20 mL). The organic phase was dried over anhydrous $MgSO_4$ and the volatiles were removed under reduced pressure. The substituted oxa[6]helicenes were obtained analytically pure and further used without purification if not indicated otherwise.

Alternatively, it is possible to avoid the purification of the intermediate alcohol and subject to purification the product after dehydration. However, the decreased stability of the final product on silica gel was observed, thus purification after the first step is advisable.

Synthesis and characterization of oxa[6]helicene proligands (-)-(*M*,*R*,*R*)- and (+)-(*P*,*S*,*S*)-**14a**-**e**.

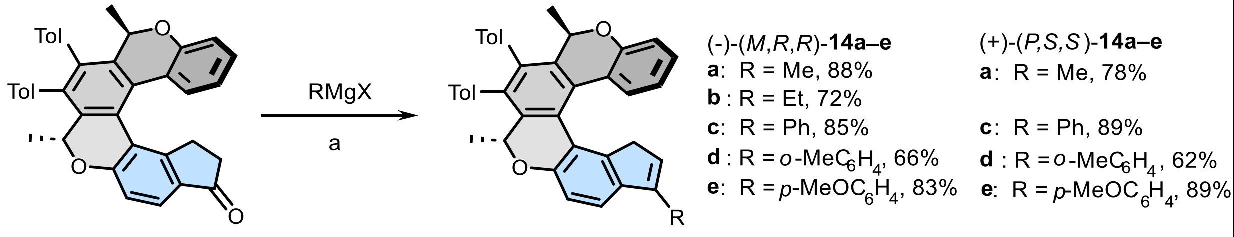

(-)-(*M*,*R*,*R*)-**13**

(a) MeMgBr (1.5 equiv.) or EtMgBr (1.5 equiv.) or PhMgCl (2.0 equiv.) or *o*-MeC_6H_4MgCl (1.5 equiv.) or *p*-$MeOC_6H_4MgBr$ (1.5 equiv.), THF, -80 °C to rt, 1 h, then *p*-TsOH (20 mol%), toluene, 110 °C, 1 min.

Compound (-)-(*M,R,R*)-**14a**

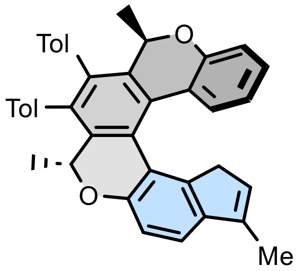

Prepared according to **GP1** starting from (-)-(*M,R,R*)-**13** (500 mg, 0.54 mmol, 1.0 equiv.), methylmagnesium bromide (0.45 mL, 3M in diethyl ether, 0.82 mmol, 1.5 equiv.), and *p*-TsOH·H_2O (31 mg, 0.15 mmol, 20 mol%) providing (-)-(*M,R,R*)-**14a** (438 mg, 88%) as a light yellow amorphous solid.

$\mathbf{R_f}$ = 0.52 (*n*-hexane:EtOAc 9:1).

$\mathbf{[\alpha]^{20}_D}$ -641.2 (*c* 0.302, THF).

^{1}H NMR (500 MHz, 298 K, $CDCl_3$): δ = 7.22 (d, *J* = 8.0 Hz, 1H), 7.19 (dd, *J* = 7.8, 1.9 Hz, 1H), 7.15 (dd, *J* = 7.8, 1.8 Hz, 1H), 7.13 – 7.06 (m, 5H), 7.03 (m, 1H), 6.88 (m, 2H), 6.73 (dd, *J* = 7.8, 1.9 Hz, 1H), 6.69 (dd, *J* = 7.8, 1.9 Hz, 1H), 6.60 (td, *J* = 7.5, 1.4 Hz, 1H), 5.83 (q, *J* = 1.9 Hz, 1H), 5.30 (q, *J* = 6.7 Hz, 1H, partially overlapping), 5.27 (q, *J* = 6.7 Hz, 1H, partially overlapping), 2.78 (dt, *J* = 23.4, 2.2 Hz, 1H), 2.27 (s, 6H), 2.17 (dt, *J* = 23.4, 2.3 Hz, 1H), 2.09 (q, *J* = 2.0 Hz, 3H), 1.00 (d, *J* = 6.7 Hz, 3H), 0.87 (d, *J* = 6.8 Hz, 3H).

^{13}C{^{1}H} NMR (126 MHz, 298 K, $CDCl_3$): δ = 152.7, 152.1, 141.94, 141.89, 140.8, 138.6, 138.2, 136.8, 136.7, 136.1, 135.1, 131.0, 130.7, 129.4, 129.1, 129.0, 128.74, 128.66, 128.53, 128.45, 127.9, 126.9, 126.2, 125.5, 125.4, 122.2, 121.9, 119.3, 119.1, 117.3, 73.3, 73.2, 38.6, 21.3 (2C), 18.7, 18.1, 13.3.

HRMS (ESI) *m*/*z*: ($[M+H]^+$) calcd for $C_{40}H_{35}O_2$ 547.2632, found 547.2627 (Δ = -0.90 ppm).

IR (ATR): 3046 (vw), 3019 (vw), 2991 (w), 2976 (w), 2919 (w), 2862 (w), 1582 (w-m), 1512 (m), 1485 (m), 1444 (m), 1419 (s), 1376 (m), 1363 (m), 1345 (m), 1299 (w), 1270 (w), 1243 (m), 1218 (s), 1181 (w-m), 1150 (s), 1107 (m), 1099 (m), 1085 (w), 1063 (s), 1029 (m), 1019 (m), 1004 (m), 963 (m), 936 (m), 905 (m), 863 (m), 834 (m), 820 (s), 806 (m), 791 (m), 754 (s), 742 (s), 682 (m) cm^{-1}.

UV/VIS (THF): λ_{max} (log ε) = 266 (4.65), 315 (4.09) nm.

Compound (+)-(*P,S,S*)-**14a**

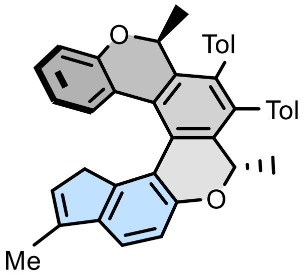

Prepared according to **GP1** starting from (+)-(*P,S,S*)-**13** (150 mg, 0.27 mmol, 1.0 equiv.), methylmagnesium bromide (0.14 mL, 3M in diethyl ether, 0.41 mmol, 1.5 equiv.), and *p*-TsOH·H_2O (9.4 mg, 0.05 mmol, 20 mol%) providing (+)-(*P,S,S*)-**14a (**116 mg, 78%) as a light yellow amorphous solid.

NMR spectra in accordance with (-)-(*M,R,R*)-**14a**.

$\mathbf{[\alpha]^{20}_D}$ +630.1 (*c* 0.166, THF)

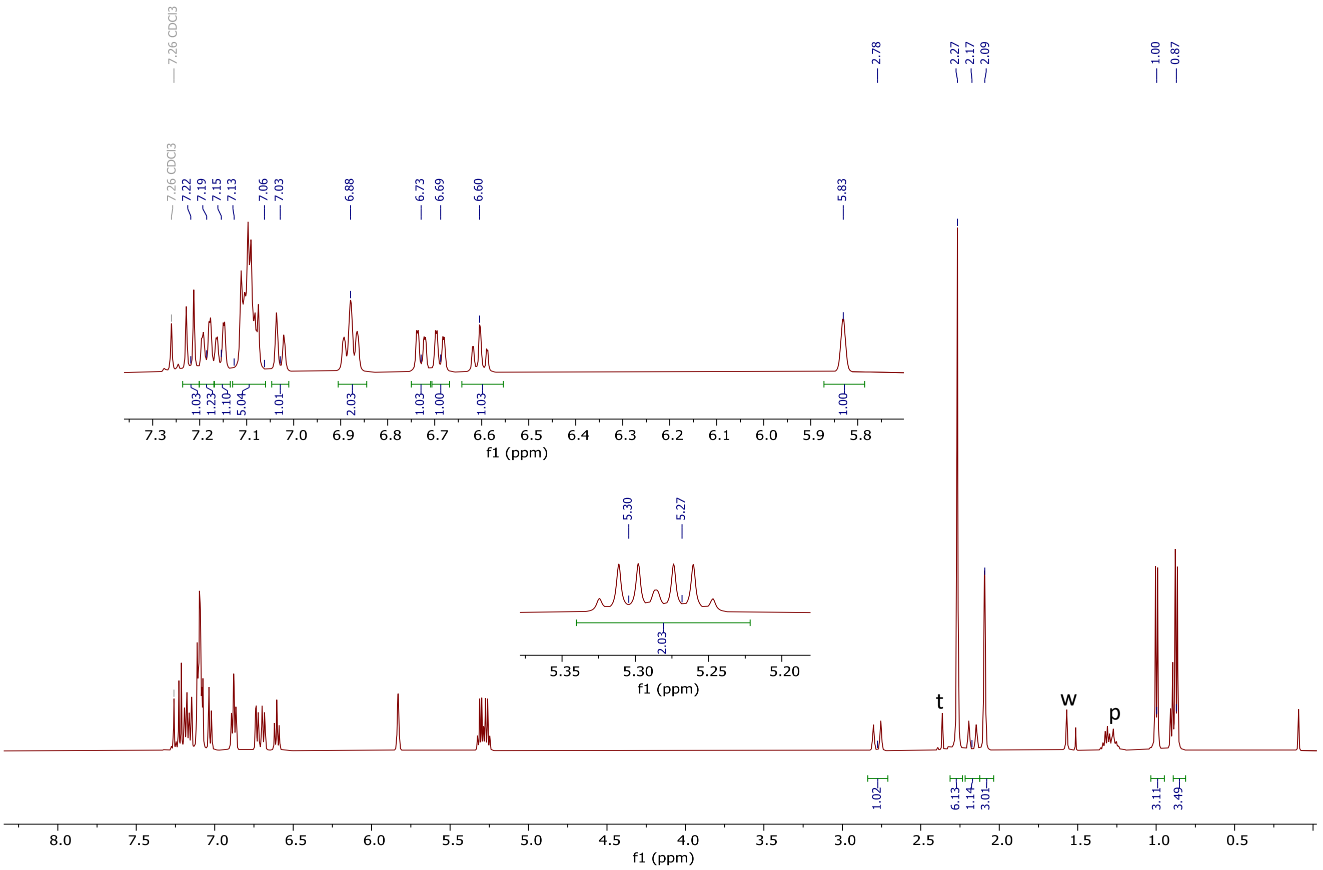

Figure S45: ^{1}H NMR of (-)-(*M,R,R*)-**14a** (500 MHz, 298 K, $CDCl_3$) (t = toluene, w = water, p = pentane).

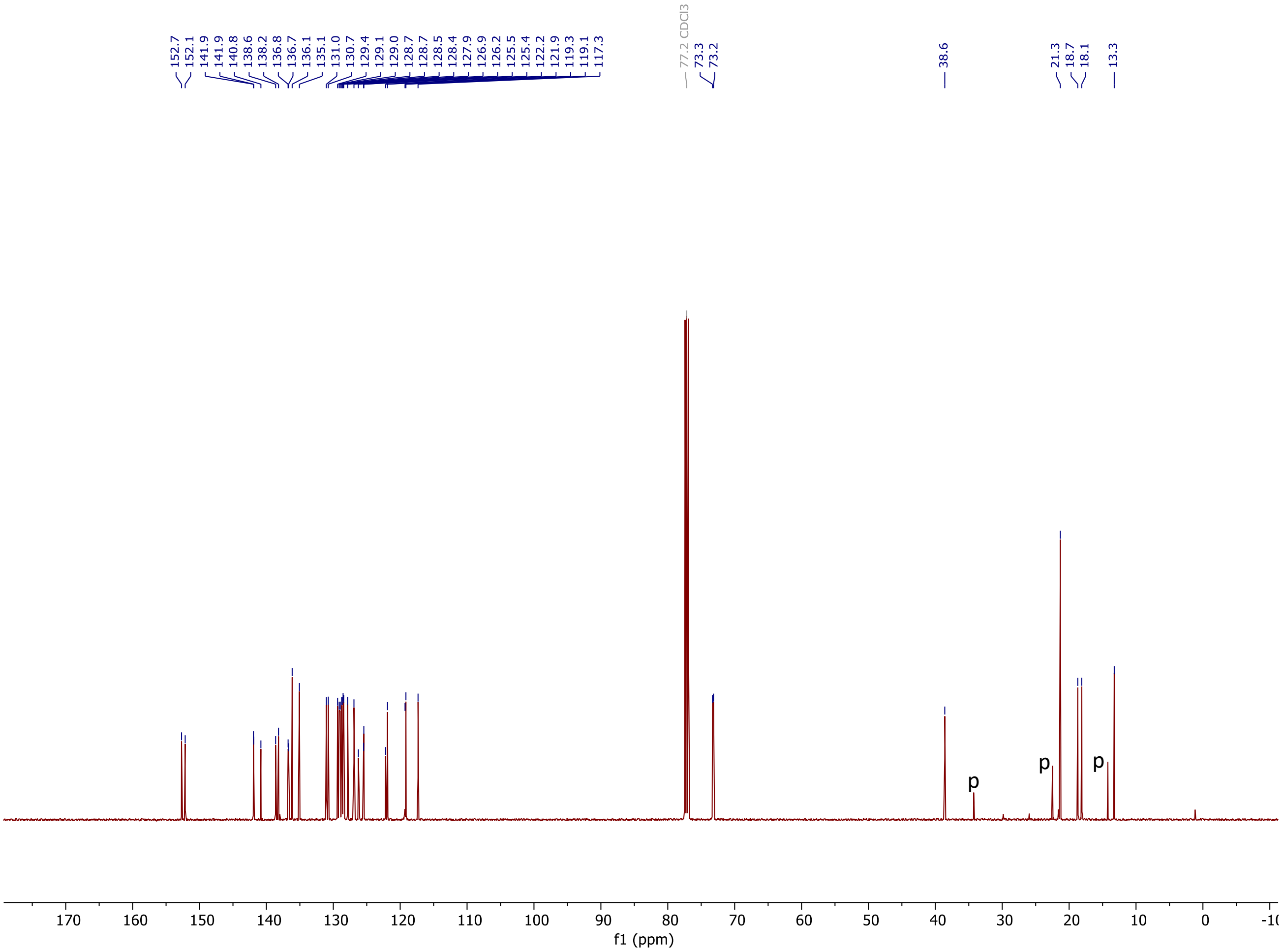

Figure S46: ^{13}C NMR of (-)-(*M,R,R*)-**14a** (126 MHz, 298 K, $CDCl_3$) (p = pentane).

Compound (-)-*(M,R,R)*-**14b**

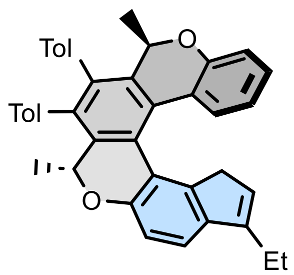

Prepared according to **GP1** starting from (-)-(*M,R,R*)-**13** (100 mg, 0.18 mmol, 1.0 equiv.), ethylmagnesium bromide (0.27 mL, 1M in THF, 0.27 mmol, 1.5 equiv.), and *p*-TsOH·H_2O (7 mg, 0.04 mmol, 20 mol%) providing (-)-(*M,R,R*)-**14b** (72.6 mg, 72%) as a light yellow amorphous solid.

R_f = 0.53 (*n*-hexane:EtOAc 9:1).

$[\alpha]^{20}_D$ -589.6 (*c* 0.137, THF).

^{1}H NMR (500 MHz, 298 K, $CDCl_3$): δ = 7.23 (d, *J* = 8.0 Hz, 1H), 7.17 (dd, *J* = 7.8, 1.9 Hz, 1H), 7.14 (dd, *J* = 7.8, 1.9 Hz, 1H), 7.11 – 7.04 (m, 5H), 7.03 – 7.00 (m, 1H), 6.90 – 6.85 (m, 2H), 6.71 (dd, *J* = 7.7, 1.9 Hz, 1H), 6.67 (dd, *J* = 7.8, 1.9 Hz, 1H), 6.59 (td, *J* = 7.5, 1.3 Hz, 1H), 5.81 (m, 1H), 5.32 – 5.22 (m, 2H), 2.77 (dq, *J* = 23.3, 2.3 Hz, 1H), 2.48 (tdd, *J* = 9.6, 6.9, 3.8 Hz, 2H), 2.26 (s, 6H), 2.17 (dq, *J* = 23.4, 2.4 Hz, 1H), 1.22 (t, *J* = 7.4 Hz, 3H), 0.98 (d, *J* = 6.7 Hz, 3H), 0.86 (d, *J* = 6.7 Hz, 3H).

^{13}C{^{1}H} NMR (126 MHz, 298 K, $CDCl_3$): δ = 152.7, 152.1, 144.4, 142.2, 141.4, 140.8, 138.6, 136.74, 136.69, 136.2 (2C), 135.1 (2C), 131.1, 130.7, 129.4, 129.1, 129.0, 128.74, 128.66, 128.5, 128.4, 126.9, 126.2, 125.7, 125.52, 125.47, 122.3, 121.9, 119.2, 119.1, 117.3, 73.3, 73.2, 38.6, 21.3 (2C), 20.8, 18.7, 18.1, 12.3.

HRMS (ESI) m/z: ($[M+H]^+$) calcd for $C_{41}H_{37}O_2$ 561.2788, found 561.2785 (Δ = -0.56 ppm).

IR ($CHCl_3$): 3081 (w), 3048 (w), 3007 (m), 2970 (s), 2928 (s), 2874 (m), 1615 (w), 1605 (w), 1584 (m), 1517 (s-m), 1487 (m), 1464 (s-m), 1459 (m), 1445 (m), 1422 (s), 1367 (s), 1358 (w), 1334 (w), 1273 (w), 1253 (m), 1235 (w), 1183 (w), 1151 (m-s), 1110 (m), 1101 (m), 1066 (s), 1033 (w), 1023 (m), 1006 (w-m), 973 (w), 965 (vw), 955 (w), 943 (w), 901 (w), 863 (m), 837 (m), 825 (m) cm^{-1}.

UV/VIS (THF): λ_{max} (log ε) = 266 (4.62), 335 (4.11) nm.

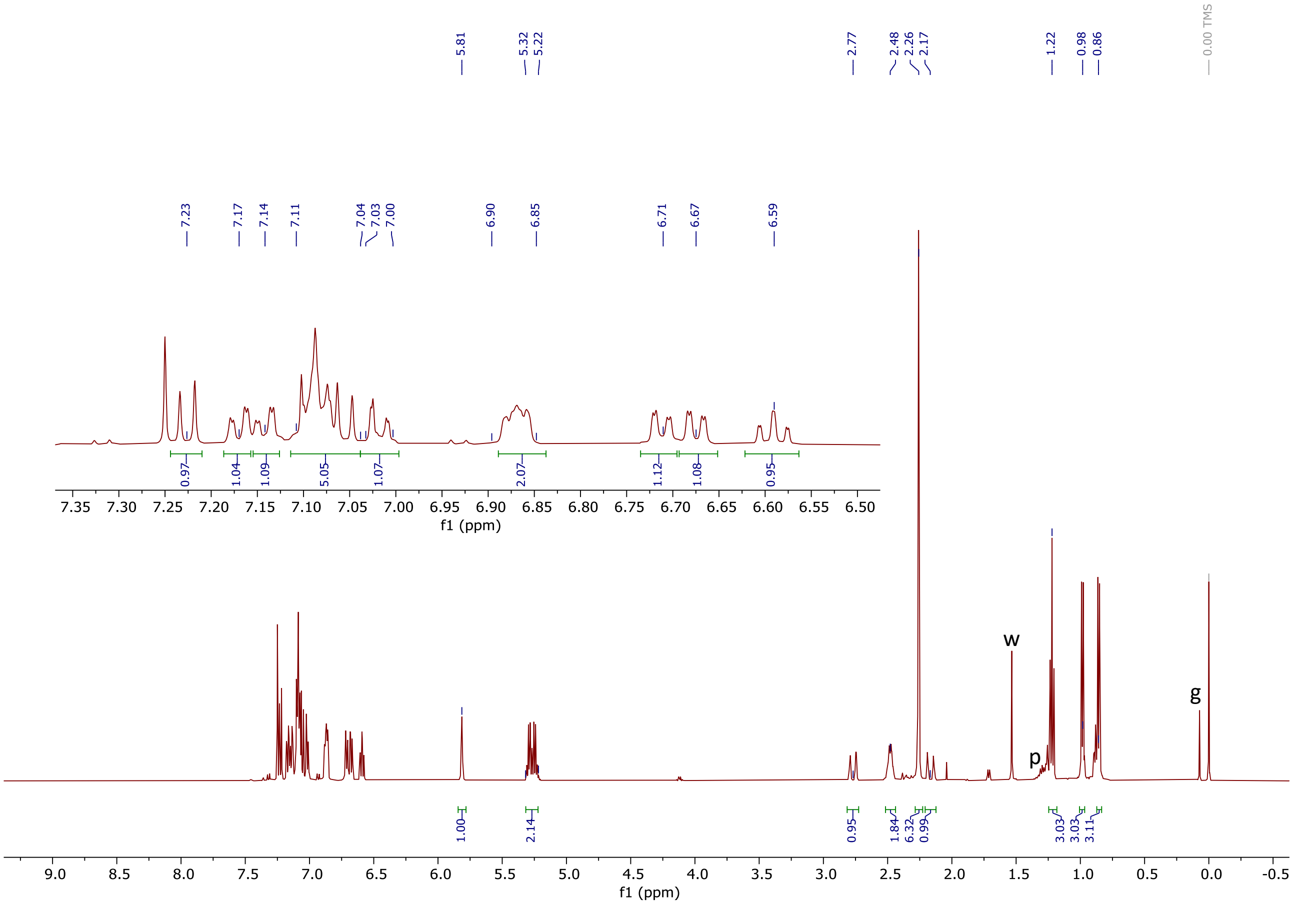

Figure S47: ^{1}H NMR of (-)-*(M,R,R)*-**14b** (500 MHz, 298 K, $CDCl_3$) (w = water, p = pentane, g = silicone grease).

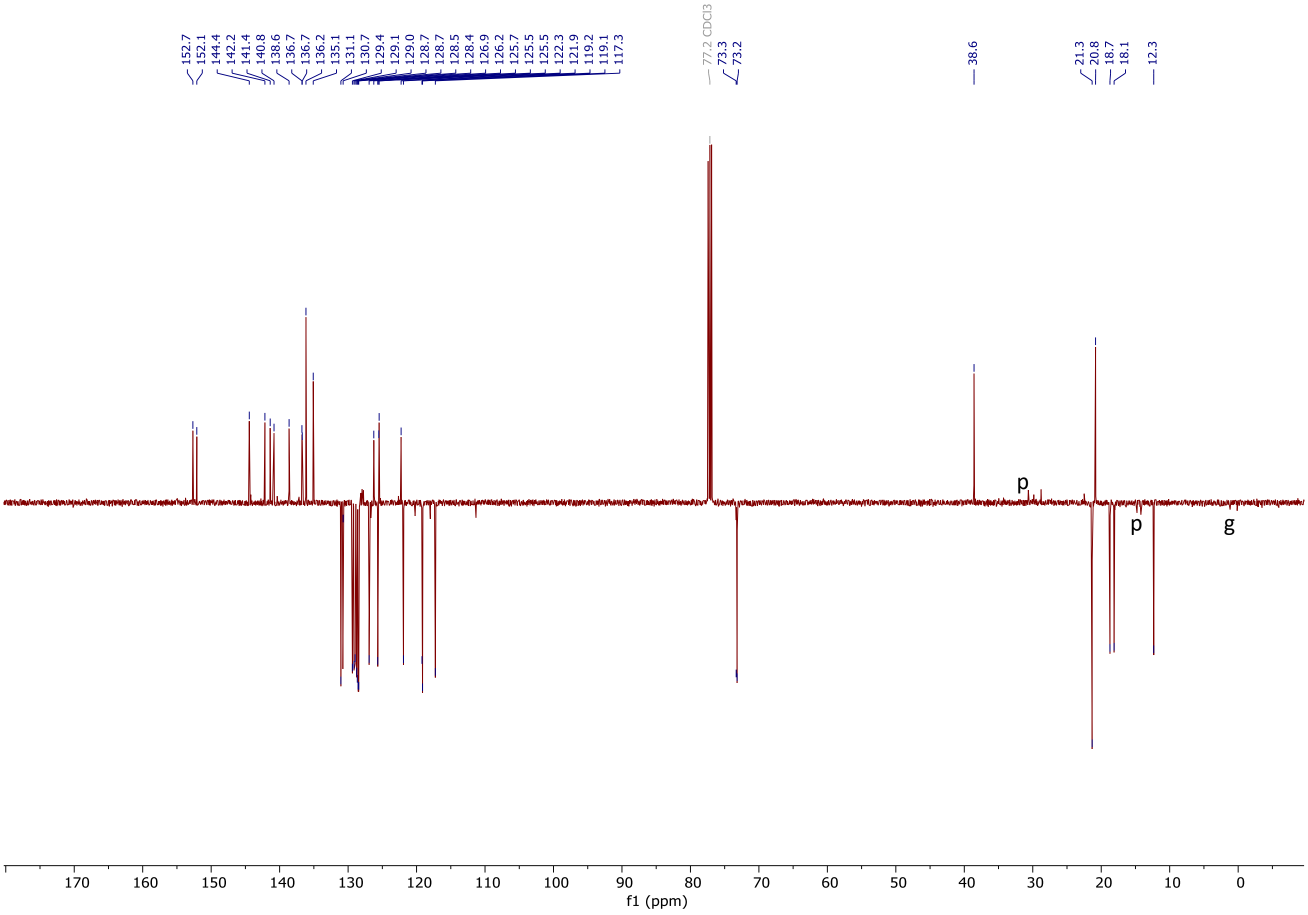

Figure S48: ^{13}C NMR of (-)-*(M,R,R)*-**14b** (126 MHz, 298 K, $CDCl_3$).

Compound (+)-(*P,S,S*)-**14c**

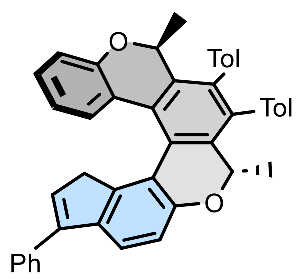

Prepared according to **GP1** starting from (+)-(*P,S,S*)-**13** (100 mg, 0.18 mmol, 1.0 equiv.), phenylmagnesium chloride (0.18 mL, 2M in THF, 0.36 mmol, 2.0 equiv.), and *p*-TsOH·H_2O (8 mg, 0.04 mmol, 20 mol%) providing (+)-(*P,S,S*)-**14c** (99 mg, 89%) as a light yellow amorphous solid.

R$_f$ = 0.45 (*n*-hexane:EtOAc 9:1).

[α]$^{20}_{D}$ +544.3 (*c* 0.286, THF).

^{1}H NMR (400 MHz, 298 K, $CDCl_3$): δ = 7.62 – 7.53 (m, 2H), 7.49 (m, 1H), 7.43 (m, 2H), 7.34 (t, *J* = 7.3 Hz, 1H), 7.23 – 7.14 (m, 3H), 7.14 – 7.02 (m, 5H), 6.94 – 6.85 (m, 2H), 6.75 (dd, *J* = 7.7, 1.9 Hz, 1H), 6.71 (dd, *J* = 7.7, 1.9 Hz, 1H), 6.63 (m, 1H), 6.22 (d, *J* = 2.3 Hz, 1H), 5.33 (q, *J* = 6.7 Hz, 1H, partially overlapping), 5.29 (q, *J* = 6.7 Hz, 1H, partially overlapping), 2.96 (dd, *J* = 24.1, 2.2 Hz, 1H), 2.38 (dd, *J* = 24.3, 2.4 Hz, 1H), 2.27 (m, 6H), 1.02 (d, *J* = 6.7 Hz, 3H), 0.90 (d, *J* = 6.7 Hz, 3H).

^{13}C{^{1}H} NMR (101 MHz, 298 K, $CDCl_3$): δ = 152.7, 152.4, 143.6, 142.4, 140.8, 139.5, 138.7, 136.9, 136.7, 136.4, 136.2 (2C), 135.03, 135.02, 131.0, 130.7, 130.1, 129.3, 129.1, 128.8, 128.7, 128.59 (2C), 128.56, 128.5, 127.9 (2C), 127.5, 126.9, 126.3, 125.4, 125.3, 122.5, 122.0, 120.9, 119.2, 117.5, 73.4, 73.2, 39.1, 21.3 (2C), 18.7, 18.2.

HRMS (ESI) m/z: ([M+H]$^+$) calcd for $C_{45}H_{37}O_2$ 609.2788, found 609.2783 (Δ = -0.83 ppm).

IR (ATR): 3022 (w), 2974 (w-m), 2923 (m), 2864 (w), 1707 (w), 1582 (m), 1515 (m), 1486 (m), 1443 (m), 1421 (s), 1381 (w), 1364 (m), 1348 (m), 1332 (w), 1298 (w), 1271 (w), 1245 (m), 1220 (s), 1182 (w), 1145 (m), 1100 (m), 1064 (s), 1024 (m), 1008 (m), 973 (m), 935 (w), 909 (w), 892 (w), 863 (w), 820 (m), 806 (m), 791 (m), 742 (vs), 697 (s), 649 (m), 518 (m), 484 (w), 452 (m) cm^{-1}.

Compound (-)-*(M,R,R)*-**14c**

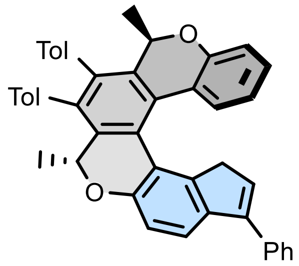

Prepared according to **GP1** starting from (-)-(*M,R,R*)-**13** (100 mg, 0.18 mmol, 1.0 equiv.), phenylmagnesium chloride (0.32 mL, 1.14M in THF, 0.22 mmol, 2 equiv.), and *p*TsOH·H_2O (7 mg, 0.04 mmol, 20 mol%) providing (-)-*(M,R,R)*-**14c** (94 mg, 85%) as a light yellow amorphous solid.

NMR spectra in accordance with (+)-(*P,S,S*)-**14c**.

[α]$^{20}_{D}$ -630.4 (*c* 0.136, THF).

UV/VIS (THF): λ_{max} (log ε) = 266 (4.64), 336 (4.07) nm.

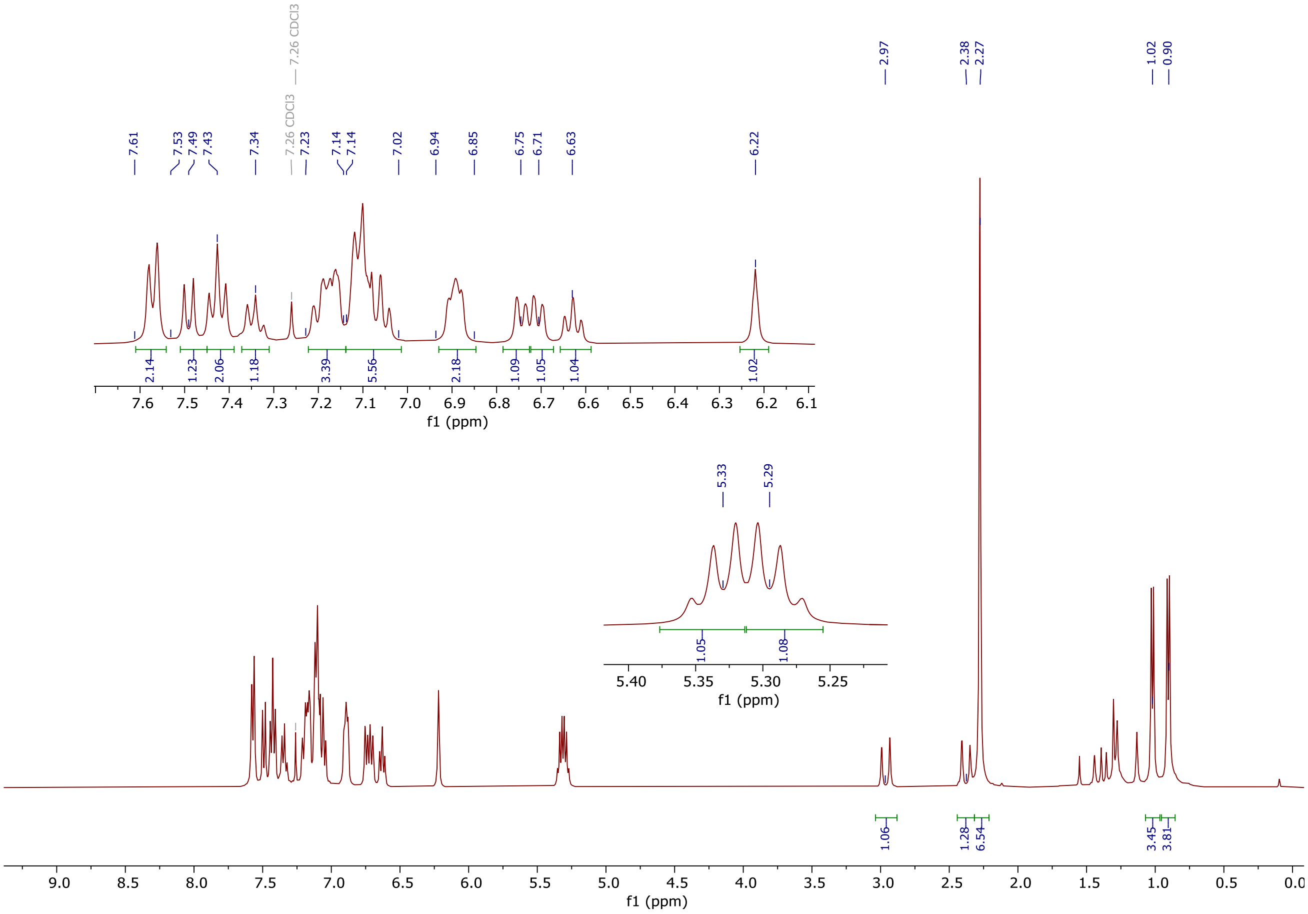

Figure S49: ^{1}H NMR of (+)-(*P,S,S*)-**14c** (400 MHz, 298 K, $CDCl_3$).

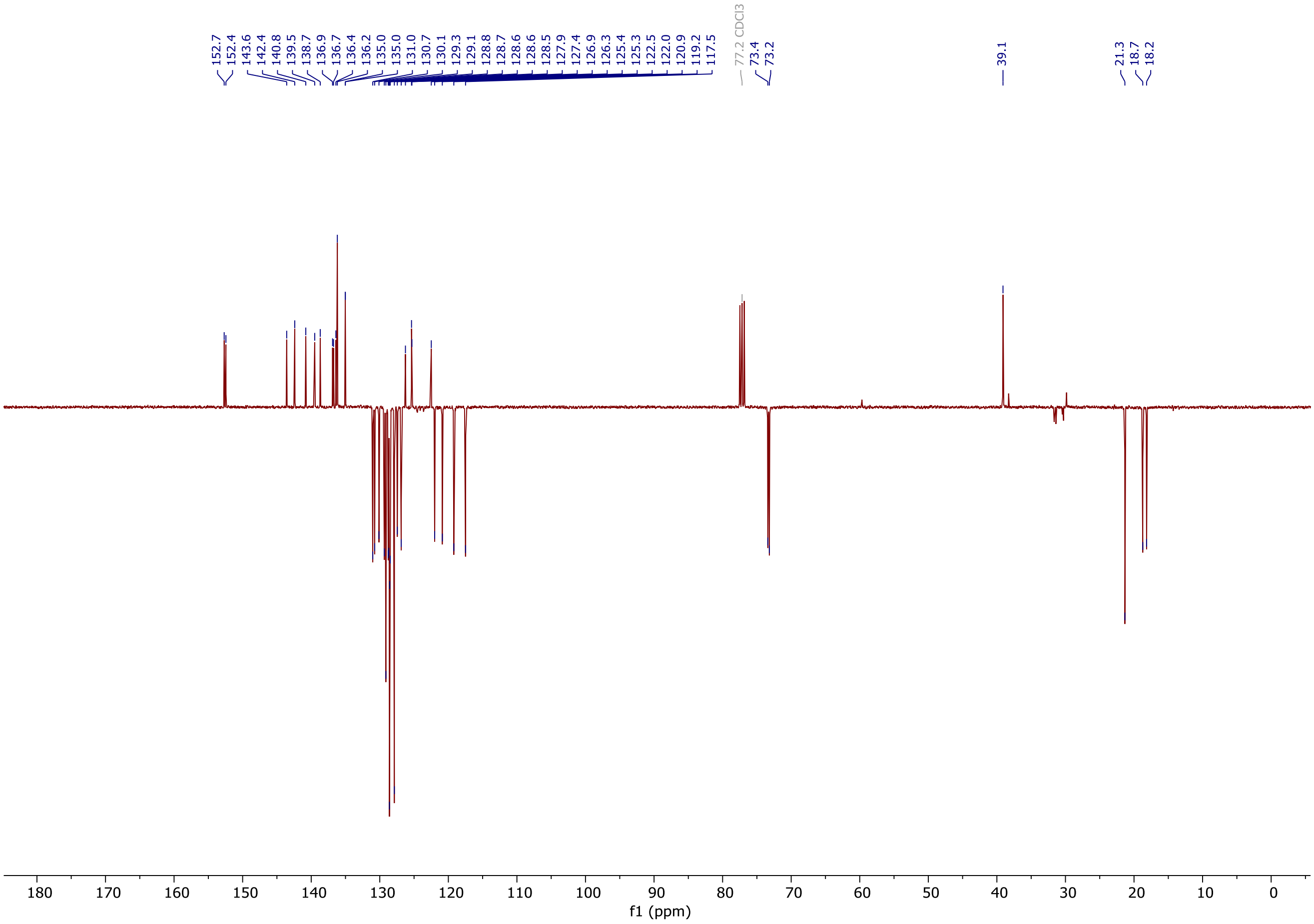

Figure S50: ^{13}C APT NMR of (+)-(*P,S,S*)-**14c** (101 MHz, 298 K, $CDCl_3$).

Compound (+)-(*P,S,S*)-**14d**

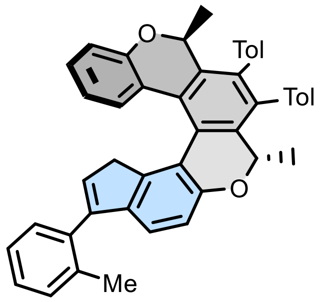

Prepared according to **GP1** starting from (+)-(*P,S,S*)-**13** (200 mg, 0.36 mmol, 1.0 equiv.), *o*-methylphenylmagnesium chloride (0.55 mL, 1M in THF, 0.55 mmol, 1.5 equiv.), and *p*-TsOH·H_2O (12.5 mg, 0.07 mmol, 20 mol%). The crude product was purified after the second step by column chromatography on silica gel (pentane:EtOAc 9:1) providing (+)-(*P,S,S*)-**14d** (139 mg, 62%) as a light yellow amorphous solid.

R_f = 0.45 (*n*-hexane:EtOAc 9:1).

$[\alpha]^{20}_D$ +523.5 (*c* 0.306, THF).

^{1}H NMR (400 MHz, 298 K, $CDCl_3$): δ = 7.29 – 7.13 (m, 7H), 7.13 – 7.09 (m, 3H), 7.07 – 7.04 (m, 1H), 7.03 – 6.95 (m, 2H), 6.89 (dt, *J* = 7.8, 2.3 Hz, 2H), 6.75 (dd, *J* = 7.8, 1.9 Hz, 1H), 6.71 (dd, *J* = 7.8, 1.9 Hz, 1H), 6.62 (td, *J* = 7.5, 1.4 Hz, 1H), 6.02 (m, 1H), 5.32 (q, *J* = 6.6 Hz, 1H, partially overlapping), 5.29 (q, *J* = 6.6 Hz, 1H, partially overlapping), 2.99 (dd, *J* = 23.8, 2.1 Hz, 1H), 2.37 (dd, *J* = 23.8, 2.3 Hz, 1H), 2.27 (d, *J* = 1.6 Hz, 6H), 2.23 (s, 3H), 1.02 (d, *J* = 6.7 Hz, 3H), 0.93 (d, *J* = 6.7 Hz, 3H).

^{13}C{^{1}H} NMR (101 MHz, 298 K, $CDCl_3$): δ = 152.8, 152.3, 143.5, 141.5, 140.9, 140.7, 138.6, 136.83, 136.76, 136.5, 136.19, 136.16, 135.1, 135. 0, 131.0, 130.7, 130.4, 130.2, 129.6, 129.4, 129.1, 128.8, 128.7, 128.6, 128.5, 127.5, 126.9, 126.1, 125.6, 125.4, 125.3, 122.1, 121.7, 121.0, 119.2, 117.3, 73.4, 73.2, 39.3, 21.3 (2C), 20.3, 18.7, 18.2.

HRMS (ESI) *m/z*: ($[M+H]^+$) calcd for $C_{46}H_{39}O_2$ 623.2945, found 623.2940 (Δ = -0.82 ppm).

IR (ATR): 3018 (w), 2972 (w), 2921 (w), 2858 (w), 1582 (w-m), 1513 (m), 1484 (m), 1441 (m), 1420 (s), 1380 (w), 1364 (m), 1338 (w), 1297 (w), 1271 (w), 1243 (m), 1219 (s), 1181 (w), 1143 (m-s), 1108 (m), 1099 (m), 1063 (s), 1020 (m), 1006 (w), 984 (w), 970 (w), 932 (w), 905 (w), 893 (w), 862 (w), 829 (m), 790 (w), 752 (vs), 740 (s), 724 (s), 683 (m), 647 (m), 618 (w), 600 (w), 560 (w), 519 (m), 488 (m), 457 (m) cm^{-1}.

UV/VIS (THF): λ_{max} (log ε) = 267 (4.59), 329 (3.98) nm.

Compound (-)-(*M,R,R*)-**14d**

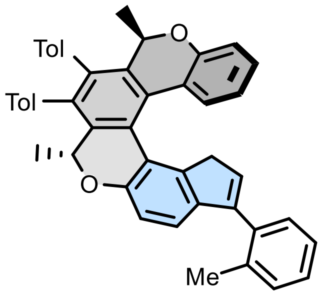

Prepared according to **GP1** starting from (-)-(*M,R,R*)-**13** (166 mg, 0.30 mmol, 1.0 equiv.), *o*-methylphenylmagnesium chloride (0.46 mL, 1M THF, 0.46 mmol, 1.5 equiv.), and *p*-TsOH·H_2O (11 mg, 0.06 mmol, 20 mol%) providing (-)-(*M,R,R*)-**14d** (126 mg, 66%) as a light yellow amorphous solid.

NMR spectra in accordance with (+)-(*P,S,S*)-**14d**.

$[\alpha]^{20}_D$ -527.4 (*c* 0.106, THF).

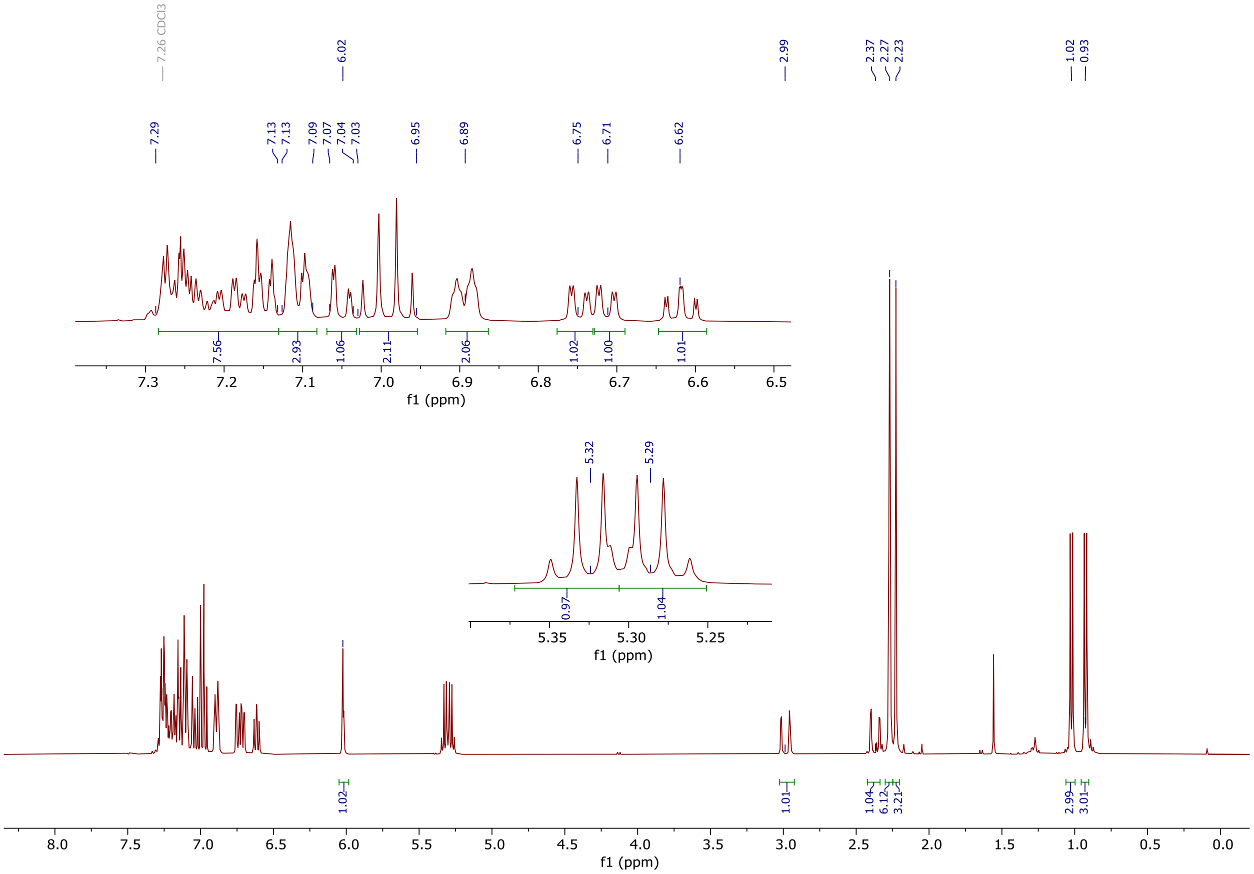

Figure S51: ^{1}H NMR of (+)-(*P,S,S*)-**14d** (400 MHz, 298 K, $CDCl_3$).

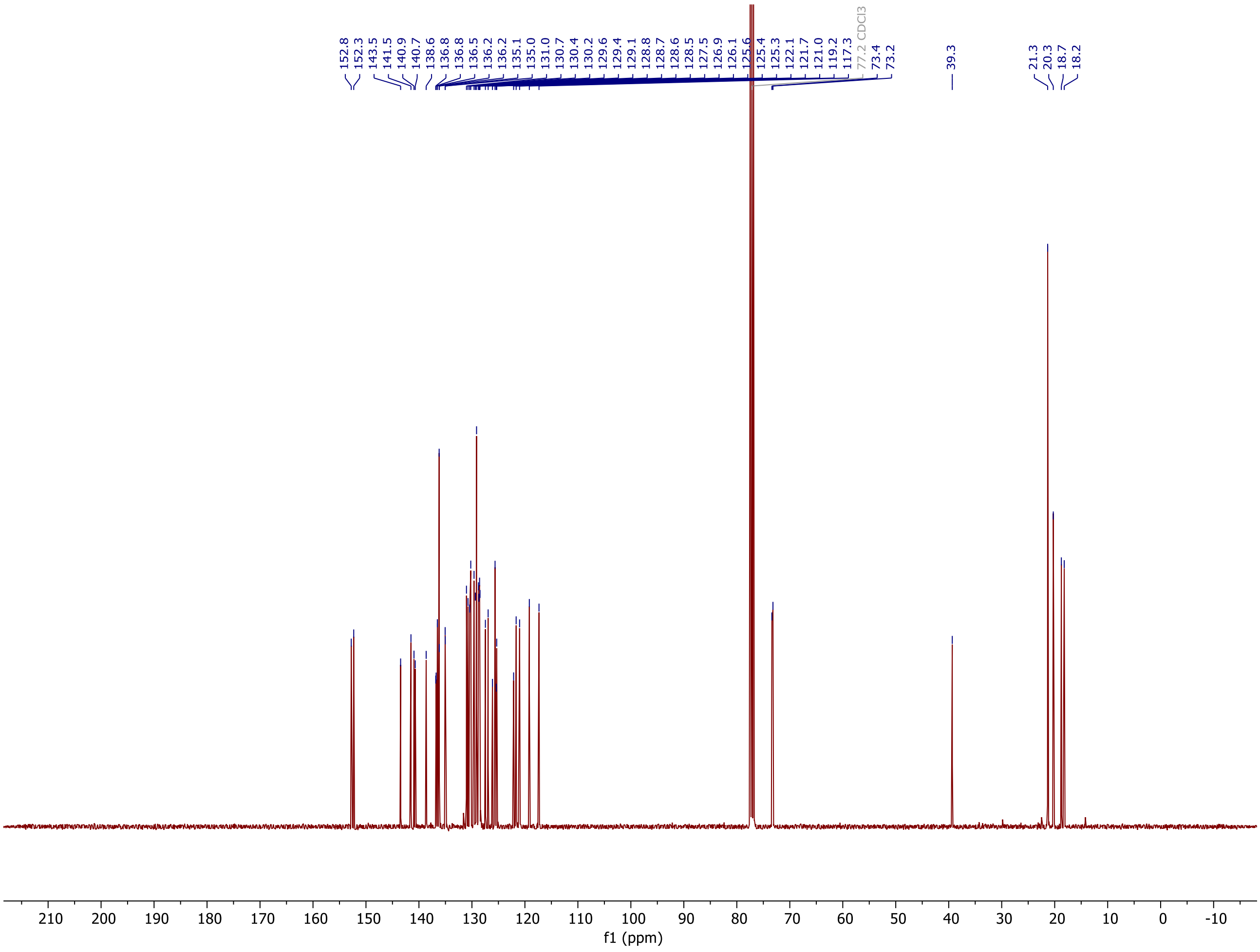

Figure S52: ^{13}C NMR of (+)-(*P,S,S*)-**14d** (101 MHz, 298 K, $CDCl_3$).

Compound (+)-(*P,S,S*)-**14e**

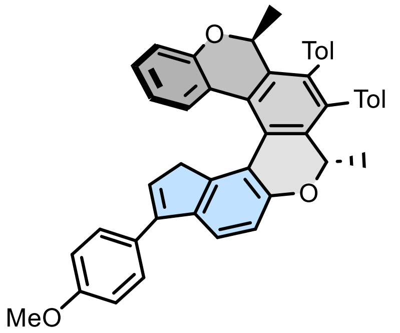

Prepared according to **GP1** starting from (+)-(*P,S,S*)-**13** (350 mg, 0.64 mmol, 1.0 equiv.), *p*-methoxyphenylmagnesium bromide (0.96 mL, 1M in THF, 0.96 mmol, 1.5 equiv.), and *p*-TsOH·H_2O (22 mg, 0.13 mmol, 20 mol%) providing (+)-(*P,S,S*)-**14e** (361 mg, 89%) as a light yellow amorphous solid.

R_f = 0.32 (*n*-hexane:EtOAc 9:1).

$[\alpha]^{20}_D$ +555.8 (*c* 0.149, THF).

^{1}H NMR (400 MHz, 298 K, $CDCl_3$): δ = 7.54 – 7.42 (m, 3H), 7.21 – 7.13 (m, 3H, overlapping with residual toluene signal), 7.12 – 7.02 (m, 5H), 6.99 – 6.93 (m, 2H), 6.92 – 6.86 (m, 2H), 6.76 – 6.67 (m, 2H), 6.62 (td, *J* = 7.5, 1.4 Hz, 1H), 6.14 (t, *J* = 2.2 Hz, 1H), 5.34 – 5.25 (m, 2H), 3.85 (s, 3H), 2.94 (dd, *J* = 24.1, 2.2 Hz, 1H), 2.35 (dd, *J* = 24.1, 2.2 Hz, 1H, overlapping with residual toluene signal), 2.27 (s, 6H), 1.01 (d, *J* = 6.6 Hz, 3H), 0.89 (d, *J* = 6.7 Hz, 3H).

^{13}C{^{1}H} NMR (101 MHz, 298 K, $CDCl_3$): δ = 159.0, 152.6, 152.2, 142.9, 142.3, 140.7, 139.6, 138.6, 136.7, 136.6, 136.1, 134.9, 134.9, 130.9, 130.6, 129.2, 129.1, 129.02, 128.97, 128.93 (2C), 128.87, 128.6, 128.5, 128.4, 128.3, 128.2, 126.8, 126.1, 125.28, 125.26, 125.2, 122.3, 121.9, 120.7, 119.1, 117.3, 113.9 (2C), 73.3, 73.1, 55.3, 38.9, 21.2 (2C), 18.6, 18.0.

HRMS (ESI) *m/z*: ($[M+H]^+$) calcd for $C_{46}H_{39}O_3$ 639.2894, found 639.2885 (Δ = -1.39 ppm).

IR (ATR): 3017 (vw), 2975 (w), 2924 (w), 2865 (vw), 1602 (w), 1578 (m), 1506 (s), 1485 (w), 1459 (m), 1440 (m), 1419 (m), 1364 (w), 1343 (w), 1290 (w), 1243 (vs), 1218 (s), 1173 (m-s), 1144 (m), 1103 (w), 1064 (s), 1035 (s), 1006 (w), 972 (w), 931 (w), 891 (w), 861 (w), 832 (vs), 789 (w), 753 (vs), 740 (s), 728 (m), 683 (w), 621 (w), 594 (w), 519 (m) cm^{-1}.

Compound (-)-(*M,R,R*)-**14e**

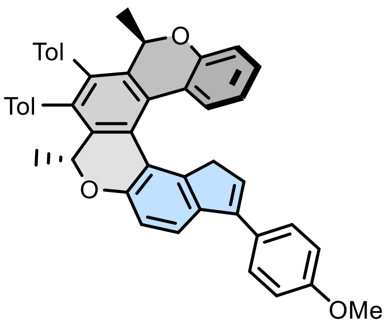

Prepared according to **GP1** starting from (*M,R,R*)-**13** (100 mg, 0.18 mmol, 1.0 equiv.), *p*-methoxyphenylmagnesium bromide (0.27 mL, 1M in THF, 0.27 mmol, 1.5 equiv.), and *p*-TsOH·H_2O (7 mg, 0.04 mmol, 20 mol%) providing (-)-(*M,R,R*)-**14e** (95.6 mg, 83%) as a light yellow amorphous solid.

NMR spectra in accordance with (+)-(*P,S,S*)-**14e**.

$[\alpha]^{20}_D$ -566.8 (*c* 0.119, THF).

UV/VIS (THF): λ_{max} (log ε) = 262 (4.68), 335 (4.06) nm.

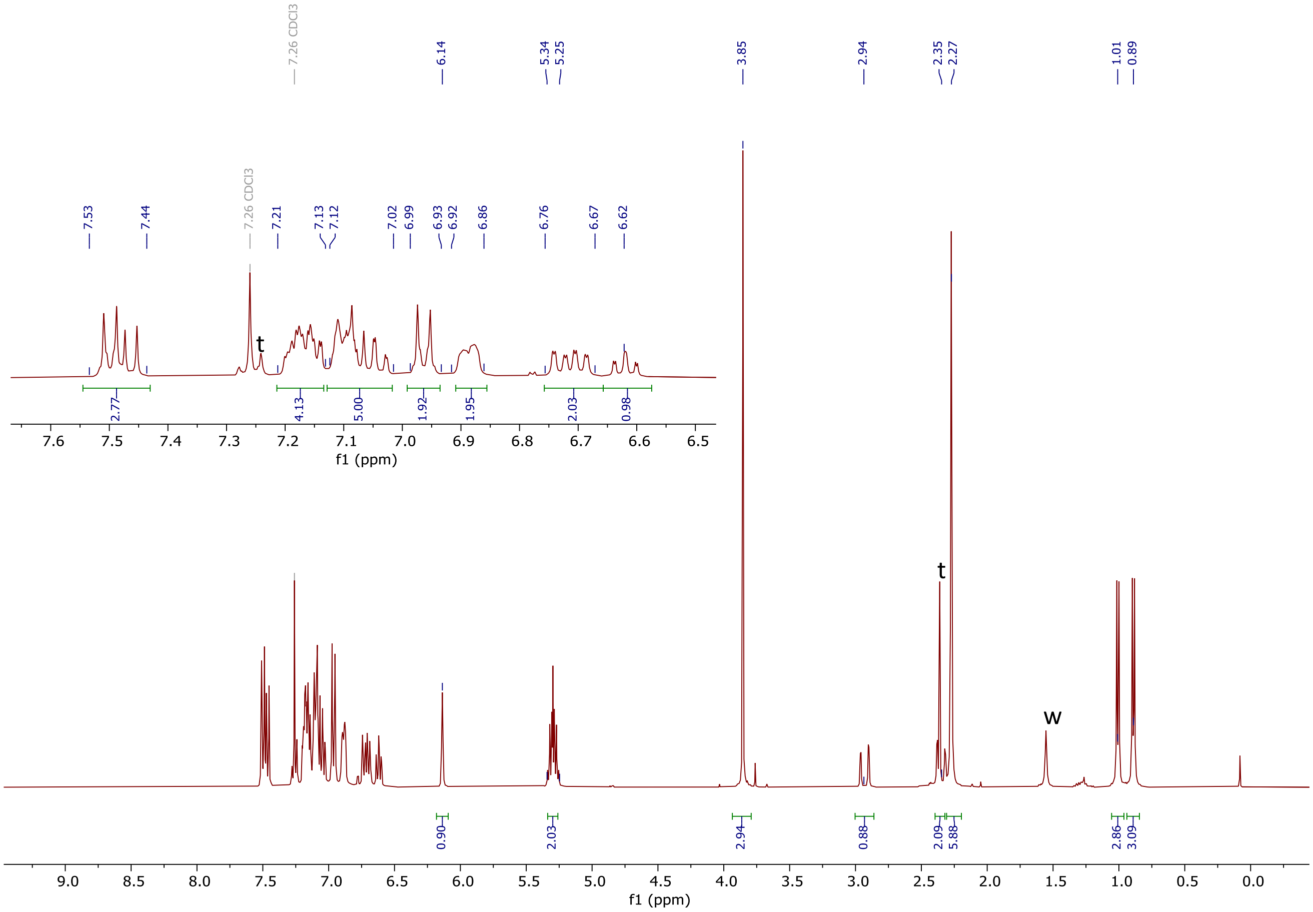

Figure S53: ^{1}H NMR of (+)-(*P,S,S*)-**14e** (400 MHz, 298 K, $CDCl_3$) (t = toluene, w = water).

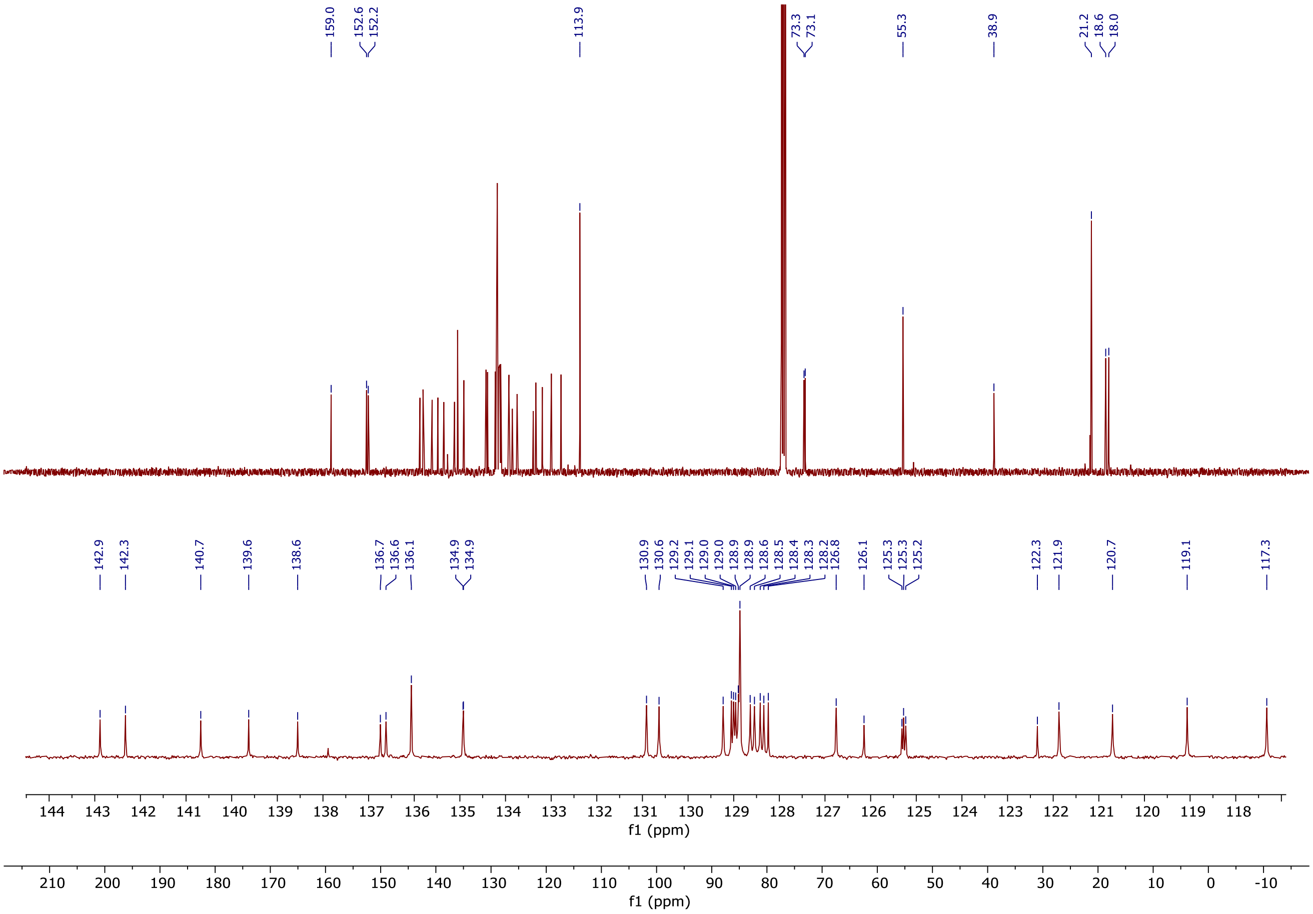

Figure S54: ^{13}C NMR of (+)-(*P,S,S*)-**14e** (101 MHz, 298 K, $CDCl_3$).

Synthesis and characterization of oxa[7]helicene proligands (-)-(*M*,*R*,*R*)-**20a**,**b**.

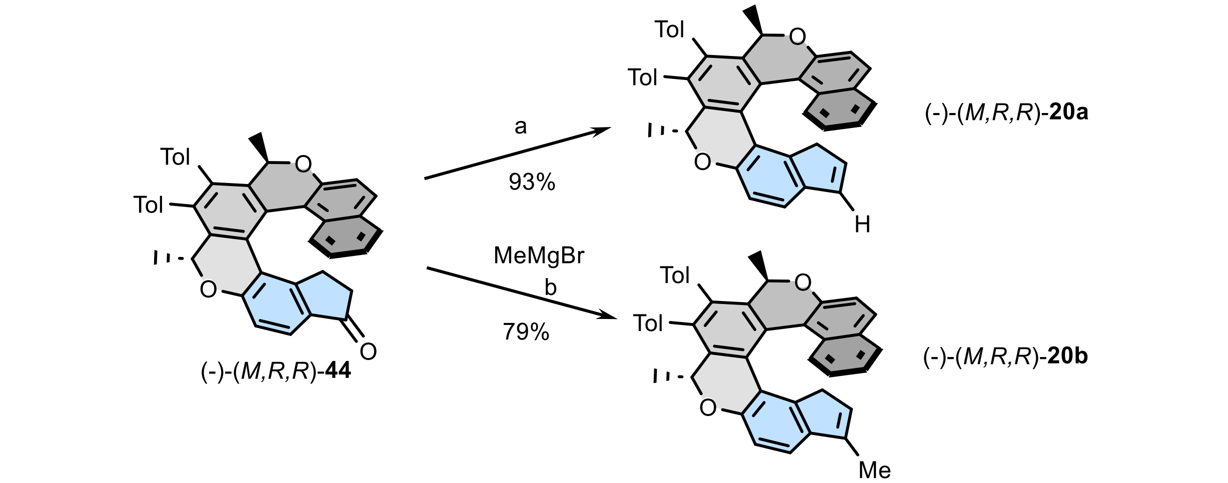

(a) $NaBH_4$ (6.0 equiv.), MeOH, 30 min, then *p*-TsOH (60 mol%), toluene, 110 °C, 1 min, 93%; (b) MeMgBr (1.5 equiv.), THF, -80 °C to rt, 1 h, then *p*-TsOH (20 mol%), toluene, 110 °C, 1 min, 79%.

Compound (-)-(*M,R,R*)-**20a**

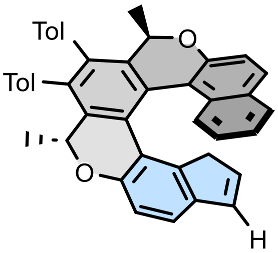

A small round bottom flask was charged with (-)-(*M,R,R*)-**44** (80 mg, 0.13 mmol, 1.0 equiv.) and suspended in MeOH (5 mL) under air. Sodium borohydride (30.4 mg, 0.8 mmol, 6.0 equiv.) was added in one portion and the reaction mixture was allowed to stir at ambient temperature for 30 min. The reaction mixture was diluted with water (20mL) and extracted with EtOAc (3x30 mL). The organic fractions were combined, washed with brine (20 mL), and dried over anhydrous $MgSO_4$. The volatiles were removed under reduced pressure. The residue was purified by fast filtration through a short column of silica gel (heptane:EtOAc 5:1). TLC of the individual fractions was visualized with a vanillin stain, and fractions containing products giving blue/green color were collected. The intermediate alcohol was isolated as a mixture of diastereoisomers, which were directly used in the next dehydration step assuming 100 % yield. The diastereomeric mixture of alcohols was dissolved in toluene (5 mL) and transferred into a small Schlenk vessel; *p*-toluenesulfonic acid monohydrate (15 mg, 0.08 mmol, 60 mol%) was added and the reaction mixture was inserted into a preheated oil bath set to 110 °C. The reaction was stirred for 60 s and then immediately cooled to ambient temperature, diluted with EtOAc (30 mL), washed with water (3x20 mL) and brine (20 mL). The organic phase was dried over anhydrous $MgSO_4$ and the volatiles were removed under reduced pressure to give (-)-(*M,R,R*)-**20a** (74 mg, 93%)as a light yellow amorphous solid. The compound shows limited stability in $CDCl_3$.

R_f = 0.47 (*n*-hexane:EtOAc 9:1).

$[\alpha]^{20}_D$ -555.8 (*c* 0.349, THF).

^{1}H NMR (400 MHz, 298 K, CD_2Cl_2): δ = 7.70 (m, 1H), 7.55 (m, 1H), 7.36 (dq, *J* = 8.6, 0.9 Hz, 1H), 7.31 (d, *J* = 8.7 Hz, 1H), 7.24 – 7.12 (m, 4H), 7.05 – 7.00 (m, 2H), 7.00 – 6.94 (m, 3H), 6.91 – 6.79 (m, 3H), 6.33 (dt, *J* = 5.5, 2.0 Hz, 1H), 5.83 (dt, *J* = 5.5, 2.0 Hz, 1H), 5.34 – 5.27 (m, 2H, overlapping with solvent signal), 5.23 (q, *J* = 6.7 Hz, 1H), 2.46 (dt, *J* = 23.6, 1.9 Hz, 1H), 2.30 (s, 6H), 1.22 (dt, *J* = 23.6, 2.2 Hz, 1H), 1.04 (d, *J* = 6.7 Hz, 3H), 0.92 (d, *J* = 6.7 Hz, 3H).

^{13}C{^{1}H} NMR(101 MHz, 298 K, CD_2Cl_2): δ = 152.5, 152.0, 141.2, 141.0, 140.2, 140.0, 137.1, 136.9, 136.8, 136.6, 135.81, 135.79, 132.4, 131.5, 131.4, 130.50, 130.48, 130.4, 129.9, 129.7, 129.3, 129.01 (2C), 129.00, 128.96, 127.9, 127.1, 125.2, 125.1, 125.0, 124.0, 123.9, 121.3, 120.5, 120.3, 117.5, 73.9, 73.6, 39.5, 21.5 (2C), 18.6, 18.3.

HRMS (ESI) *m/z*: ($[M+Na]^+$) calcd for $C_{43}H_{34}O_2Na$ 605.2451, found 605.2450 (Δ = -0.12 ppm).

IR ($CHCl_3$): 3120 (w), 3059 (w-m), 2986 (m), 2928 (m), 2867 (w), 2812 (vw), 1619 (w), 1591 (m-s), 1558 (w), 1516 (s), 1483 (vw), 1463 (m), 1444 (w), 1428 (s), 1404 (vw), 1381 (s), 1367 (m-s), 1354 (w), 1324 (w), 1308 (vw), 1300 (w), 1252 (m), 1234 (m), 1200 (w), 1183 (w), 1150 (s), 1142 (w), 1117 (w), 1103 (w), 1088 (w), 1077 (m), 1061 (s), 1039 (m), 1022 (m), 1000 (m), 979 (w), 954 (m-w), 916 (w), 909 (w), 867 (w), 855 (w), 840 (s), 826 (s), 818 (s), 557 (w), 533 (m-w) cm^{-1}.

UV/VIS (THF): λ_{max} (log ε) = 254 (5.19), 358 (4.62) nm.

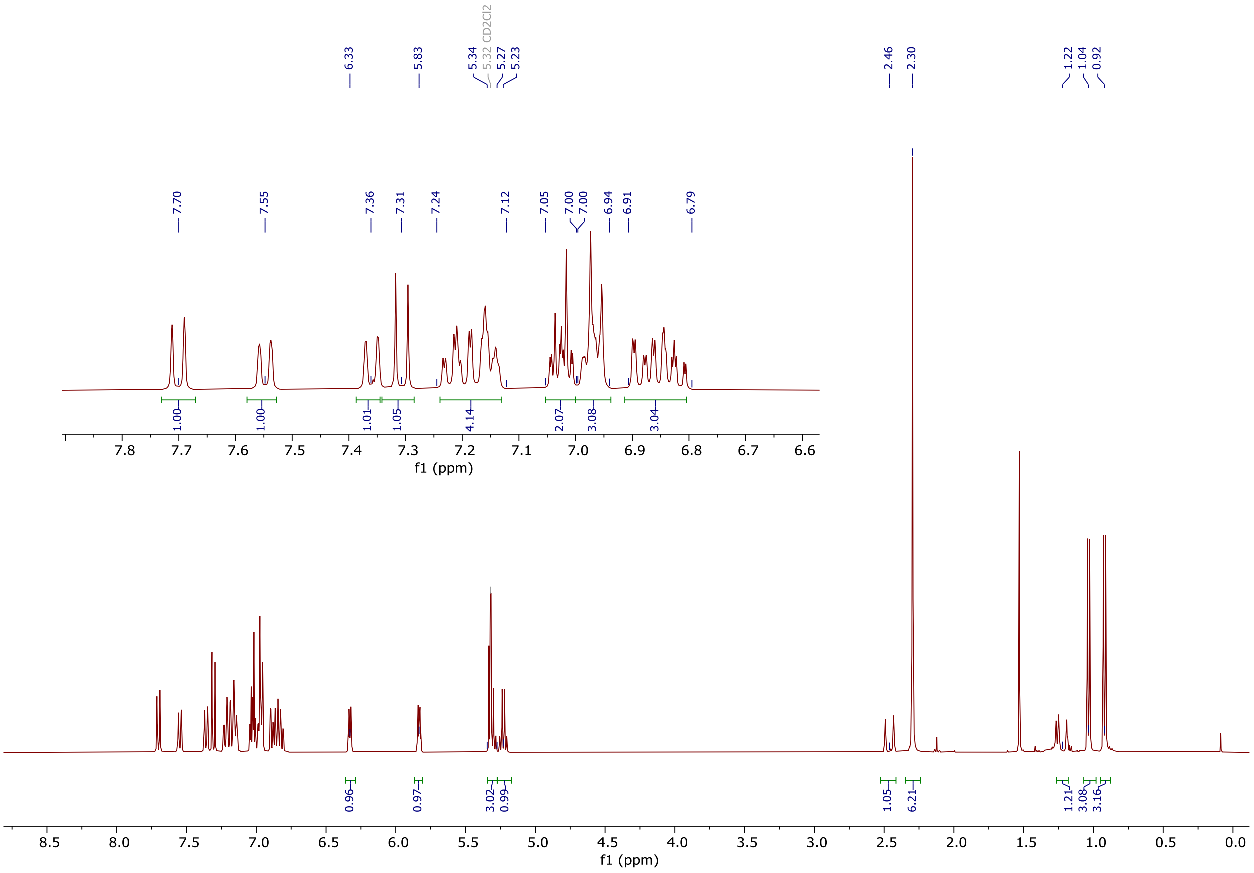

Figure S55: ^{1}H NMR of (-)-(*M,R,R*)-**20a** (400 MHz, 298 K, CD_2Cl_2).

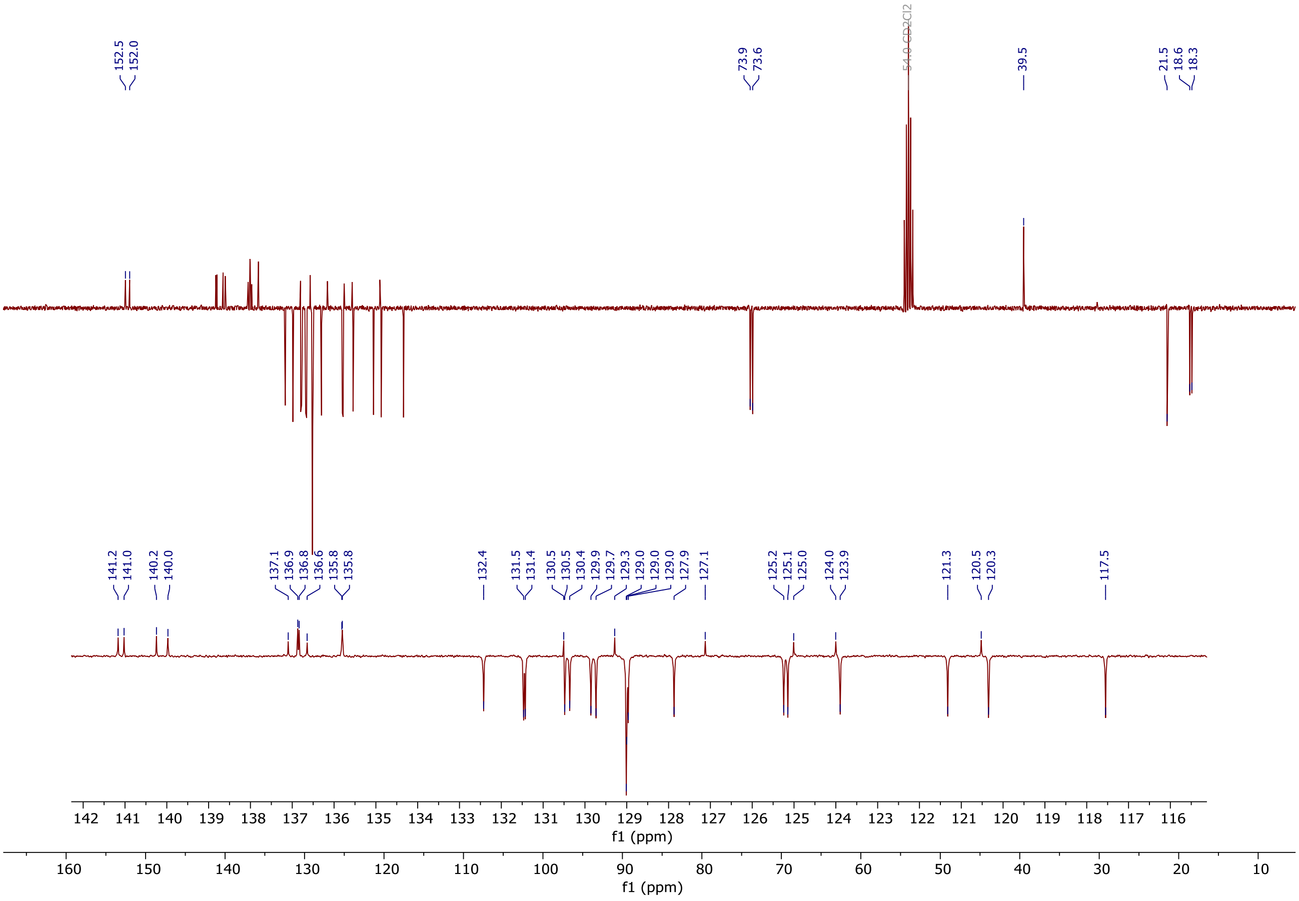

Figure S56: ^{13}C APT NMR (-)-(*M,R,R*)-**20a** (101 MHz, 298 K, CD_2Cl_2).

Compound (-)-(*M,R,R*)-**20b**

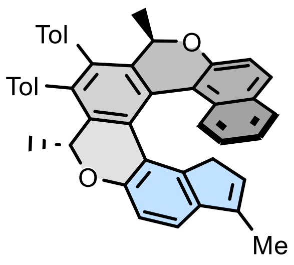

Prepared according to **GP1** starting from (-)-(*M,R,R*)-**44** (83 mg, 0.14 mmol, 1.0 equiv.), methylmagnesium bromide (0.07 mL, 3M in diethyl ether, 0.21 mmol, 1.5 equiv.), and *p*TsOH·H_2O (10 mg, 0.05 mmol, 38 mol%). The compound was purified after dehydration by column chromatography on silica gel (*n*-hexane:EtOAc 1:0 to 5:1) to give (-)-(*M,R,R*)-**20b** (65 mg, 79%) as a light yellow amorphous solid.

R$_f$ = 0.45 (*n*-hexane:EtOAc 9:1).

[α]^{20}$_D$ -542.7 (*c* 0.313, THF).

^{1}H NMR (600 MHz, 298 K, $CDCl_3$): δ = 7.67 (d, *J* = 8.7 Hz, 1H), 7.52 (m, 1H), 7.41 (dd, *J* = 8.5, 1.0 Hz, 1H), 7.29 (d, *J* = 8.7 Hz, 1H), 7.22 (dd, *J* = 7.7, 1.9 Hz, 1H), 7.19 (dd, *J* = 7.7, 1.9 Hz, 1H), 7.12 (dd, *J* = 4.6, 1.8 Hz, 1H), 7.09 (dd, *J* = 4.6, 1.8 Hz, 1H), 7.02 – 6.99 (m, 2H), 6.95 – 6.89 (m, 3H), 6.84 – 6.78 (m, 3H), 5.47 (q, *J* = 1.8 Hz, 1H), 5.37 (q, *J* = 6.7 Hz, 1H), 5.30 (q, *J* = 6.7 Hz, 1H), 2.41 (dt, *J* = 23.2, 2.2 Hz, 1H), 2.28 (s, 6H), 1.83 (q, *J* = 2.0 Hz, 3H), 1.13 (dt, *J* = 23.1, 2.3 Hz, 1H), 1.05 (d, *J* = 6.7 Hz, 3H), 0.94 (d, *J* = 6.7 Hz, 3H).

^{13}C{^{1}H} NMR (151 MHz, 298 K, $CDCl_3$): δ = 151.9, 151.4, 141.6, 141.2, 140.5, 139.4, 137.6, 136.6, 136.18, 136.15 (2C), 135.31, 135.29, 131.0, 130.9, 130.03, 130.00, 129.4, 129.3, 128.9, 128.67, 128.66, 128.5 (2C), 127.5, 126.82, 126.75, 125.0, 124.72, 124.71, 123.5, 123.4, 120.2, 119.8, 118.8, 116.7, 73.5, 73.3, 37.5, 21.3 (2C), 18.5, 18.2, 13.0.

HRMS (ESI) *m/z*: ([M+H]$^+$) calcd for $C_{44}H_{37}O_2$ 597.2788, found 597.2787 (Δ = -0.16 ppm).

IR ($CHCl_3$): 3132 (vw), 3090 (w), 3055 (w), 2984 (m-s), 2929 (m-s), 2867 (w-m), 2812 (vw), 1602 (m), 1591 (m), 1515 (s), 1496 (vw), 1463 (m), 1445 (m), 1429 (m), 1379 (s), 1367 (m-s), 1349 (m), 1335 (w), 1324 (w), 1254 (s), 1246 (m), 1235 (m-s), 1183 (w), 1151 (m), 1110 (w), 1097 (w), 1076 (m), 1062 (s), 1035 (w), 1021 (m), 1009 (w), 999 (w), 963 (w), 956 (w), 935 (vw), 902 (w), 865 (w-m), 839 (s), 817 (s), 558 (w), 535 (w) cm^{-1}.

UV/VIS (THF): λ_{max} (log ε) = 254 (4.76), 321 (4.15), 358 (3.97) nm.

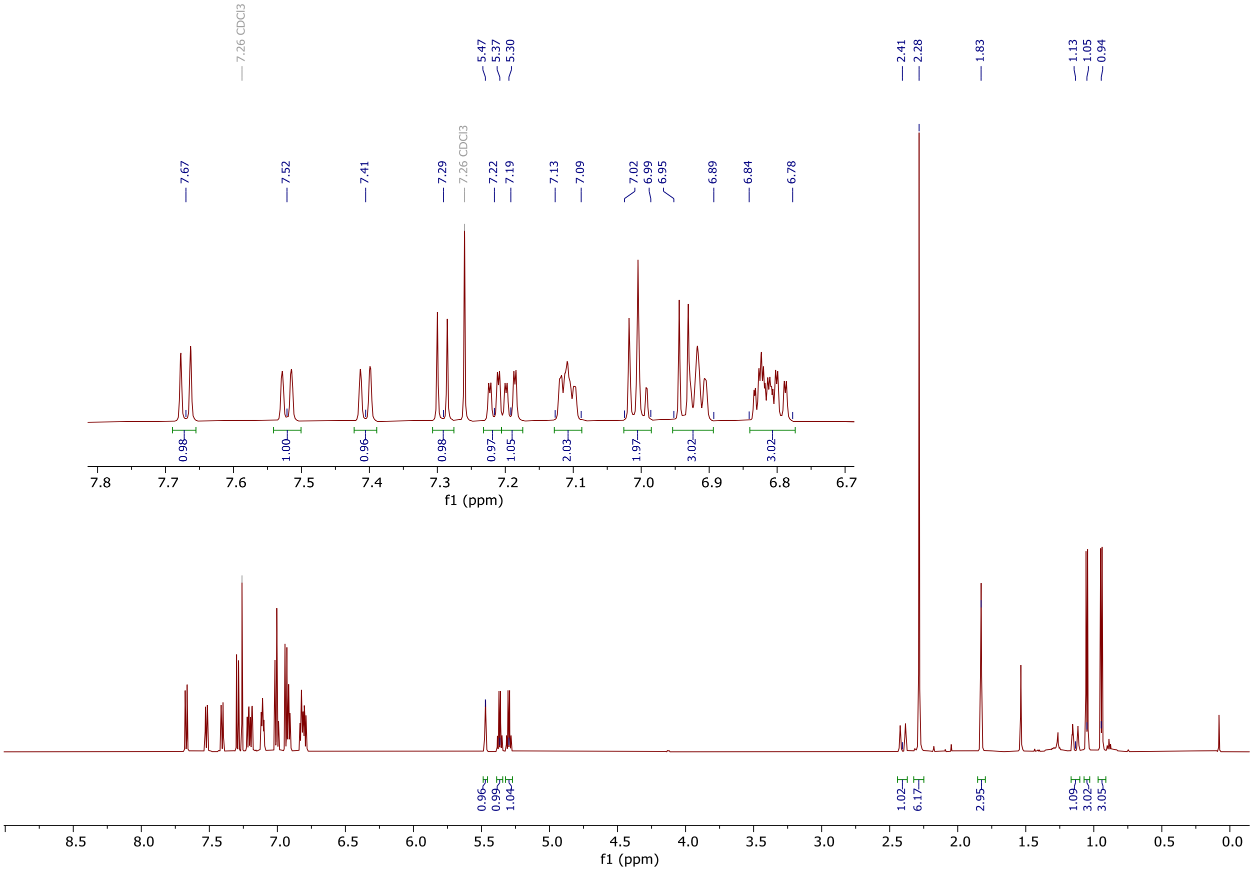

Figure S57: ¹H NMR of (-)-(*M,R,R*)-**20b** (600 MHz, 298 K, $CDCl_3$).

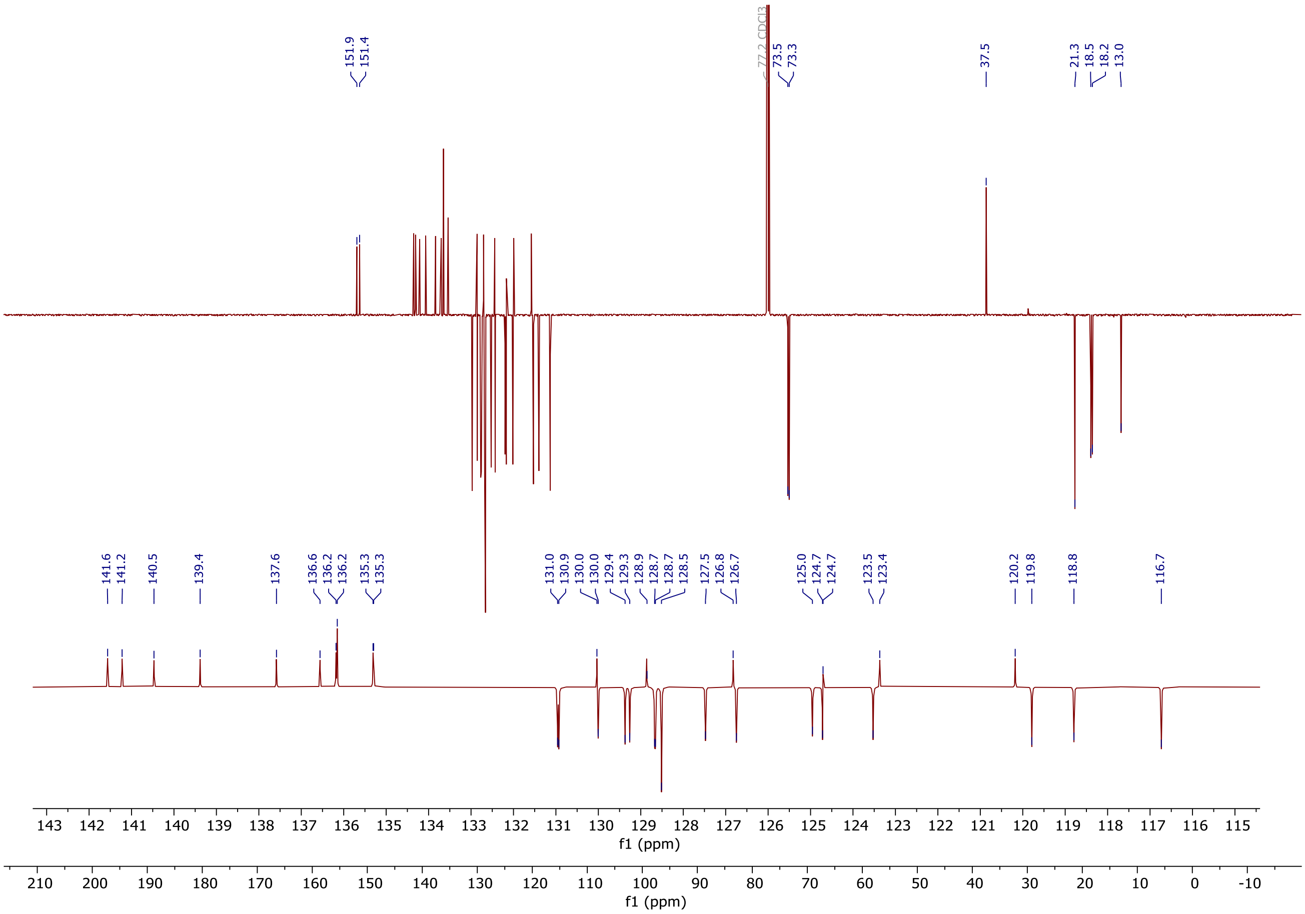

Figure S58: ¹³C APT NMR of (-)-(*M,R,R*)-**20b** (151 MHz, 298 K, $CDCl_3$).

Synthesis and characterization of oxa[6]helicene proligand (-)-(*M*,*R*)-**21** with only one chiral center.

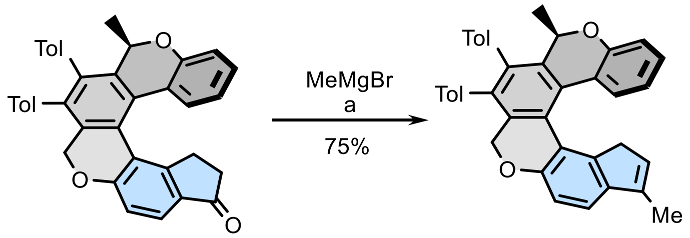

(-)-(*M*,*R*)-**49** (-)-(*M*,*R*)-**21**

(a) MeMgBr (2.0 equiv.), THF, -80 °C to rt, 1 h, then *p*-TsOH (20 mol%), toluene, 110 °C, 1 min, 75%.

Compound (-)-(*M,R*)-**21**

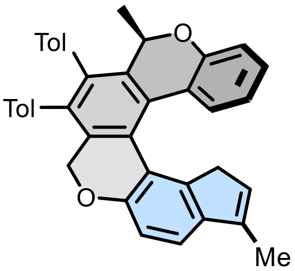

Prepared according to **GP1** starting from (-)-(*M,R,*)-**49** (80 mg, 0.15 mmol, 1.0 equiv.), methylmagnesium bromide (0.1 mL, 3M in diethyl ether, 0.3 mmol, 2.0 equiv.), and *p*TsOH·H_2O (5.7 mg, 0.03 mmol, 20 mol%) providing (-)-(*M,R*)-**21** (59.8 mg, 75%) as a light yellow amorphous solid.

$\mathbf{R_f}$ = 0.50 (*n*-hexane:EtOAc 9:1).

$[\alpha]^{20}_D$ -571.9 (*c* 0.293, THF).

^{1}H NMR (400 MHz, 298 K, $CDCl_3$): δ = 7.22 (d, *J* = 8.0 Hz, 1H), 7.15 – 7.06 (m, 4H), 7.05 – 6.98 (m, 5H), 6.96 – 6.90 (m, 2H), 6.73 (dd, *J* = 7.7, 1.9 Hz, 1H), 6.61 (m, 1H), 5.83 (q, *J* = 2.5 Hz, 1H), 5.18 (q, *J* = 6.6 Hz, 1H), 4.81 (d, *J* = 13.9 Hz, 1H), 4.39 (d, *J* = 13.9 Hz, 1H), 2.78 – 2.69 (m, 1H), 2.28 (s, 3H), 2.26 (s, 3H), 2.17 – 2.07 (t, *J* = 2.2 Hz, 1H overlapping signal), 2.09 (s, 3H, overlapping signal), 1.13 (d, *J* = 6.7 Hz, 3H).

^{13}C{^{1}H} NMR (101 MHz, 298 K, $CDCl_3$): δ = 155.6, 152.9, 142.14, 142.10, 138.9, 138.2, 137.8, 137.2, 136.5, 136.43, 136.38, 135.2, 135.1, 131.2, 130.1, 129.6, 129.3, 129.1, 128.8, 128.7, 128.6, 128.3, 128.1, 126.9, 126.8, 126.1, 125.4, 122.9, 121.9, 119.3, 119.2, 115.6, 73.2, 69.0, 38.6, 21.3, 19.3, 13.2.

HRMS (ESI) *m/z*: ($[M+H]^+$) calcd for $C_{39}H_{33}O_2$ 533.2475, found 533.2471 (Δ = -0.70 ppm).

IR (ATR): 3060 (vw), 3025 (vw), 2974(w), 2917 (w), 2852 (w), 1604 (w), 1583 (w), 1516 (m), 1486 (m) 1462 (m), 1447 (m), 1422 (s), 1377 (m), 1348 (m), 1328 (w), 1295 (w), 1274 (w-m), 1258 (m), 1248 (s), 1219 (s), 1183 (m), 1144 (m), 1110 (m), 1063 (m), 1010 (vs), 945 (m), 907 (w), 894 (w), 842 (m), 832 (s), 819 (vs), 688 (m), 641 (w), 575 (w), 533 (m), 506 (m) cm^{-1}.

UV/VIS (THF): λ_{max} (log ε) = 266 (4.47), 316 (3.92) nm.

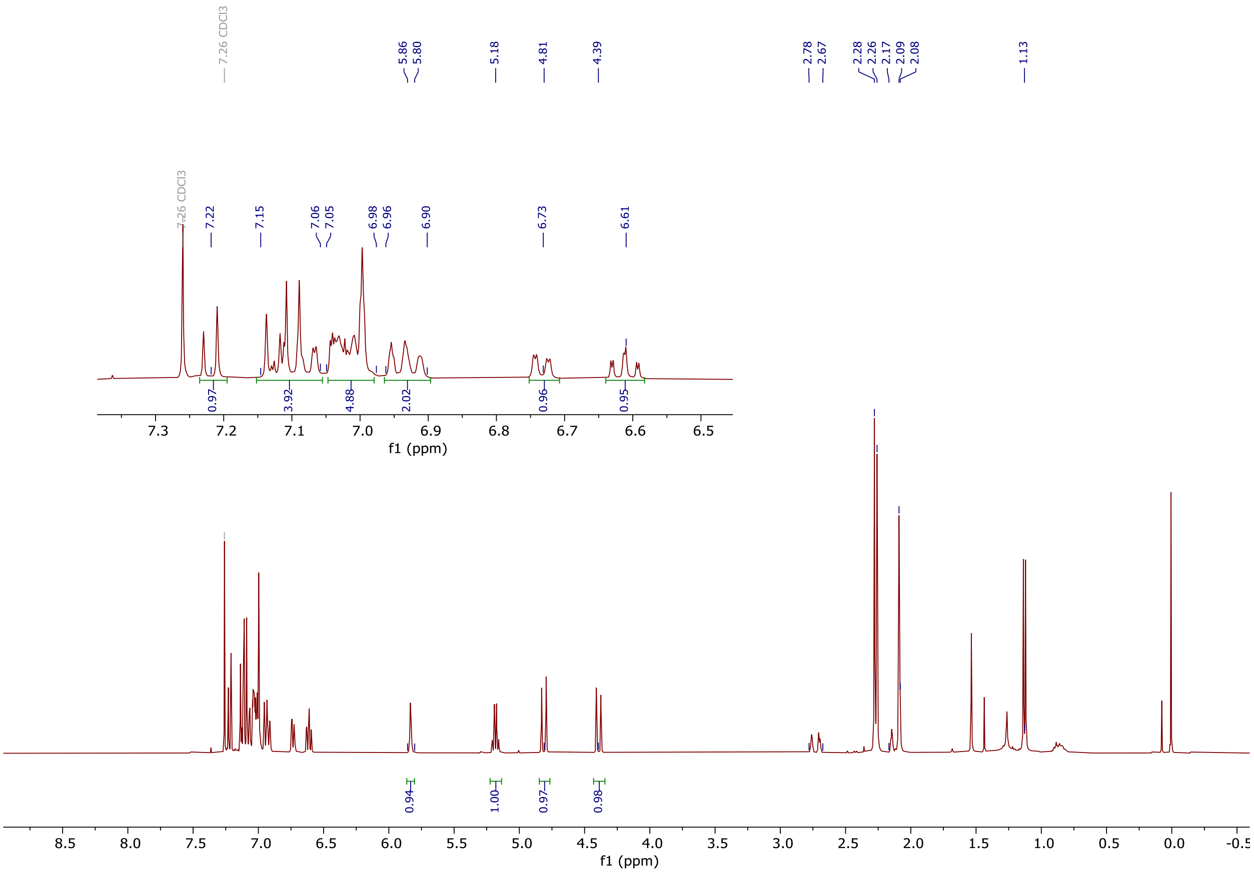

Figure S59: ^{1}H NMR of (-)-(*M,R*)-**21** (600 MHz, 298 K, $CDCl_3$).

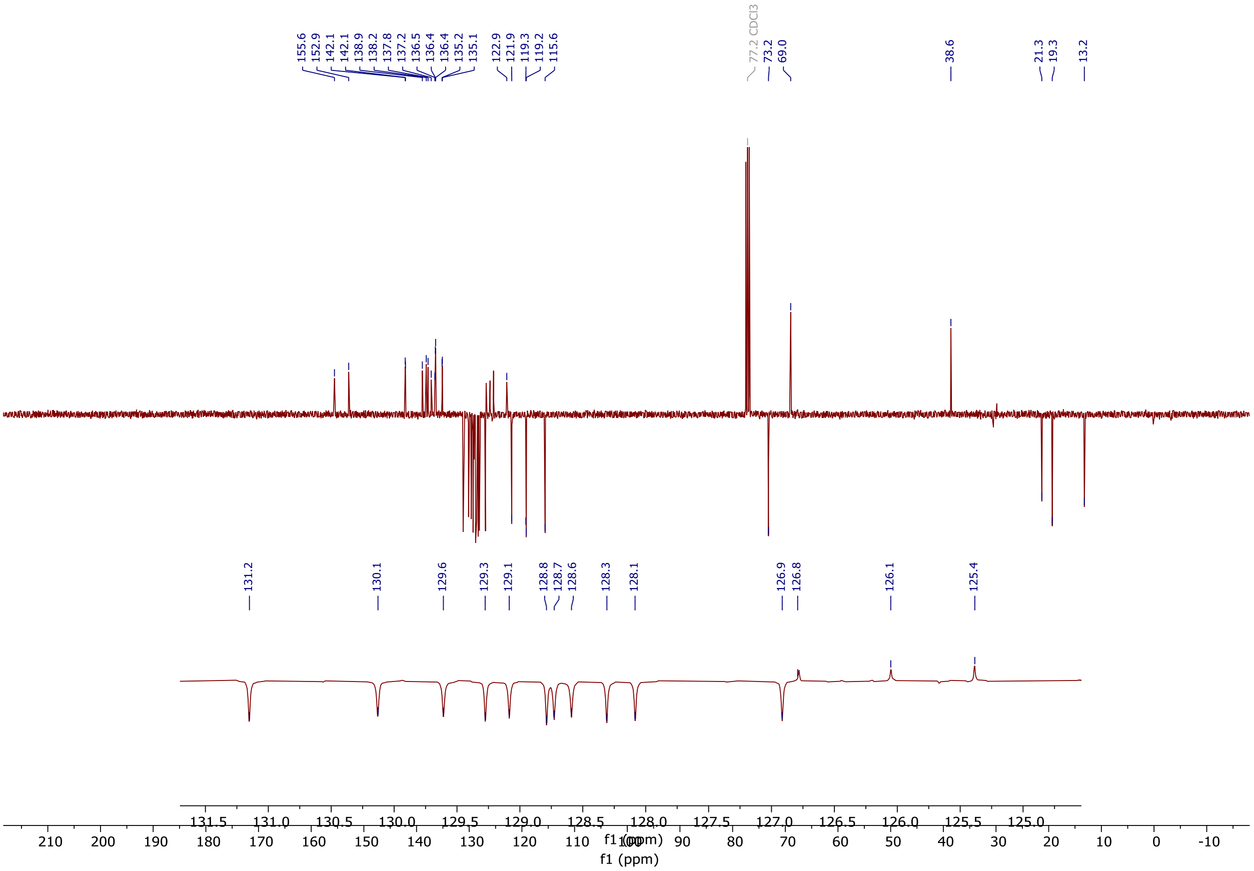

Figure S60: ^{13}C APT NMR of (-)-(*M,R*)-**21** (151 MHz, 298 K, $CDCl_3$).

Synthesis and characterization of symmetric oxa[7]helicene proligand (-)-(*M,R,R*)-**18** and potassium salt (*M,R,R*)-**19**

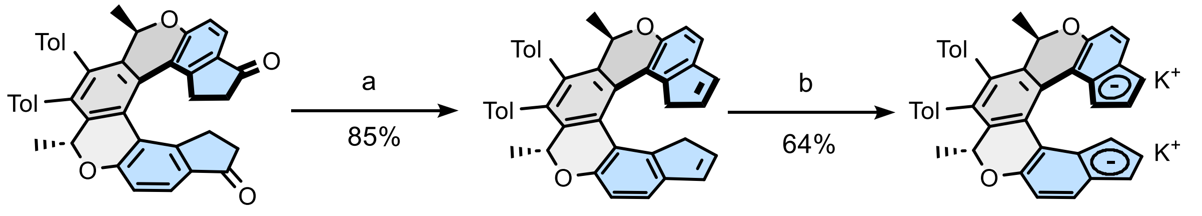

(-)-(*M*,*R*,*R*)-**17** (-)-(*M*,*R*,*R*)-**18** (*M*,*R*,*R*)-**19**

(a) $NaBH_4$ (8.0 equiv.), MeOH, rt, 30 min, then *p*-TsOH (20 mol%), toluene, 110 °C, 1 min, 85%; (b) KHMDS (2.0 equiv.), diethyl ether, rt, 5 min, 64%.

Compound (-)-(*M,R,R*)-**18**

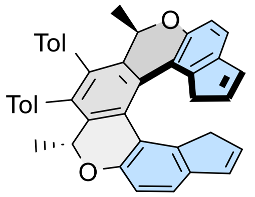

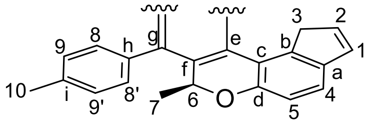

A small round bottom flask was charged with (-)-(*M,R,R*)-**17** (600 mg, 1.0 mmol, 1.0 equiv.) and suspended in MeOH (30 mL) under air. Sodium borohydride (300 mg, 7.9 mmol, 8 equiv.). was added in one portion and the reaction mixture was allowed to stir at ambient temperature for 30 min. The reaction mixture was diluted with water (20mL) and extracted with EtOAc (3x50 mL). The organic fractions were combined, washed with brine (20 mL), and dried over anhydrous $MgSO_4$. The volatiles were removed under reduced pressure. The residue was purified by fast filtration through a short column of silica gel (heptane:EtOAc 4:1). TLC of the individual fractions was visualized with a vanillin stain, and fractions containing products giving blue/green color were collected. The intermediate alcohol was isolated as a mixture of diastereoisomers, which were directly used in the next dehydration step assuming 100 % yield. The diastereomeric mixture of alcohols was dissolved in toluene (15 mL) and transferred into a small Schlenk vessel; *p*-toluenesulfonic acid monohydrate (35 mg, 0.18 mmol, 20 mol%) was added and the reaction mixture was inserted into a preheated oil bath set to 110 °C. The reaction was stirred for 60 s and then immediately cooled to ambient temperature, diluted with EtOAc (50 mL), washed with water (3x20 mL) and brine (20 mL). The organic phase was dried over anhydrous $MgSO_4$ and the volatiles were removed under reduced pressure to give (-)-(*M,R,R*)-**18** (478 mg, 85 %) as a light yellow amorphous solid.

OR $[\alpha]^{20}_{D}$ -577.7 (*c* 0.242, THF)

^{1}H NMR (500 MHz, 298 K, $CDCl_3$) δ = 7.23 (d, *J* = 8.0 Hz, 2H, CH^4), 7.19 (dd, *J* = 7.8, 1.9 Hz, 2H, C$H^{8/8'}$), 7.10 (dd, *J* = 7.9, 1.9 Hz, 2H, C$H^{9/9'}$), 7.06 (d, *J* = 7.9 Hz, 2H, CH^5), 6.89 (dd, *J* = 7.8, 1.9 Hz, 2H, C$H^{9/9'}$), 6.74 (dd, *J* = 7.6, 1.9 Hz, 2H, C$H^{8/8'}$), 6.56 (dt, *J* = 5.5, 1.9 Hz, 2H, CH^1), 6.08 (dt, *J* = 5.6, 2.0 Hz, 2H, CH^2), 5.29 (q, *J* = 6.6 Hz, 2H, CH^6), 2.78 (dt, *J* = 23.8, 1.9 Hz, 2H, CH_2^3), 2.27 (s, 6H, CH_3^{10}), 2.05 (dt, *J* = 23.6, 2.2 Hz, 2H, CH_2^3), 0.93 (d, *J* = 6.7 Hz, 6H, CH_3^7).

^{13}C{^{1}H} NMR (126 MHz, 298 K, $CDCl_3$) δ = 151.3 (C^d), 140.9, 140.7 ($C^{b,c}$), 140.3 (C^f), 136.3 (C^g), 136.1 (C^i), 135.2 (C^h), 133.2 (CH^2), 130.9 ($CH^{8/8'}$), 130.3 (CH^1), 129.3 ($CH^{8/8'}$), 128.7 ($CH^{9/9'}$), 128.5 ($CH^{9/9'}$), 126.3 (C^e), 124.2 (C^a), 121.4 (CH_3^4), 117.7 (CH^5), 73.2 (CH^6), 40.0 (CH_2^3), 21.3 (CH_3^{10}), 18.3 (CH_3^7).

HRMS (ESI) *m/z*: $[M + H]^+$ calcd for $C_{42}H_{35}O_2$ 571.2632, found 571.2626 (Δ = -0.95 ppm).

IR (ATR) 3056 (w), 3028 (w), 2983 (w), 2937 (w), 2917 (m), 2857 (w), 1739 (w), 1586 (m), 1556 (w), 1515 (m), 1459 (m-s), 1424 (m), 1420 (s), 1375 (m-s), 1363 (m-s), 1349 (m), 1301 (m), 1248 (s), 1211 (s), 1181 (m), 1163 (w), 1145 (s), 1108 (m), 1083 (m-s), 1064 (s), 1031 (s)1020 (s), 984 (m), 972 (w), 947 (m), 933 (w), 924 (w), 911 (m), 856 (m), 947 (m), 928 (s), 811 (vs), 806 (s), 798 (s), 764 (m), 756 (w-m), 740 (w), 723 (m), 712 (w), 701 (s), 681 (vs), 675 (s), 633 (s), 624 (m), 606 (m), 588 (s), 526 (s), 474 (vs).

UV/VIS (THF): λ_{max} (log ε) = 261 (4.71), 343 (3.97) nm.

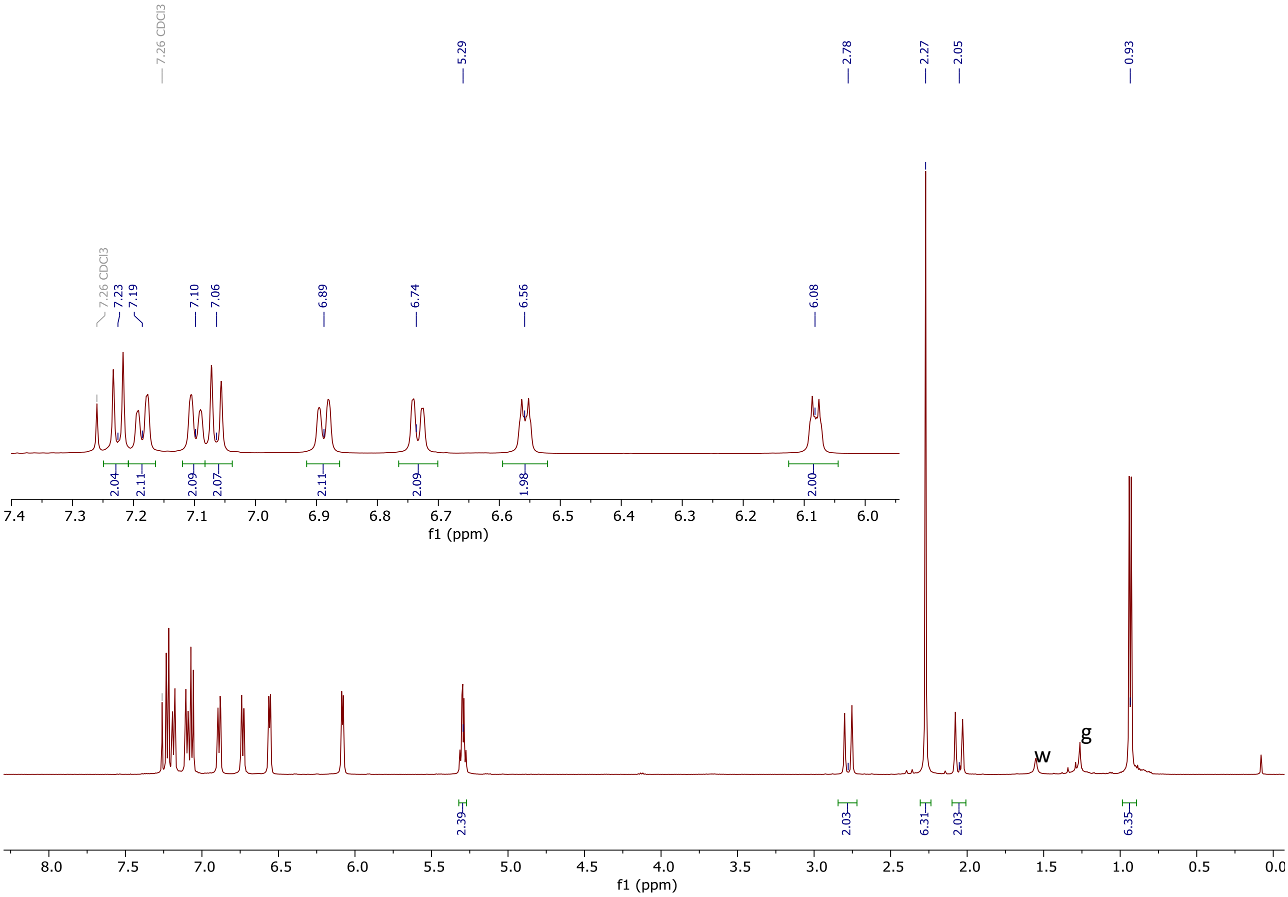

Figure S61: ^{1}H NMR of (-)-(*M,R,R*)-**18** (500 MHz, 298 K, $CDCl_3$) (w = water, g = grease)

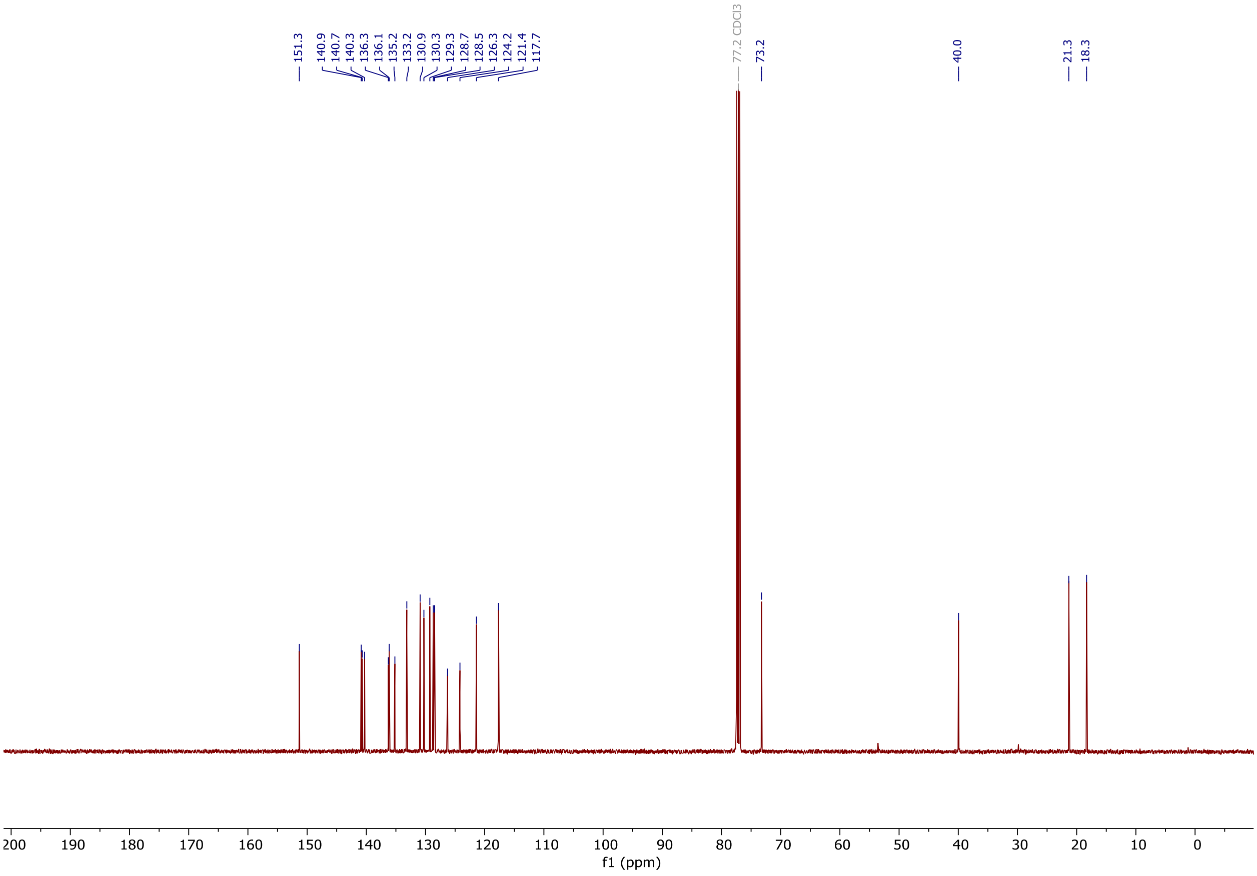

Figure S62: ^{13}C NMR of (-)-(*M,R,R*)-**18** (126 MHz, 298 K, $CDCl_3$)

Compound (*M,R,R*)-**19**

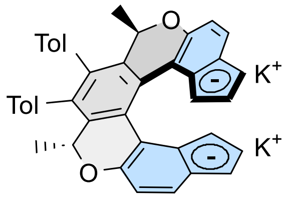

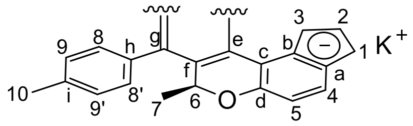

In an Ar glovebox, the bis indenyl helicene (-)-(*M,R,R*)-**18** (300 mg, 0.53 mmol, 1.0 equiv.) was dissolved in 8 mL of diethyl ether in a 20 mL vial containing a stir bar. In a separate vial KHMDS (209 mg, 1.05 mmol, 2.0 equiv.) was dissolved in 4 mL of diethyl ether. Then the solution of KHMDS was added dropwise to the stirred solution of the helicene. Immediate color change and precipitation occurred. The mixture was allowed to stir for 5 more minutes and then the precipitate was filtered, washed with diethyl ether and dried on the frit. The solid was scraped to give the product as a brick red powder containing 1.2 equivalents. of diethylether (248 mg, 64%, M_{corr}= 735 g/mol).

^{1}H NMR (500 MHz, 298 K, THF-d_8) δ =7.21 (dd, *J* = 7.8, 1.9 Hz, 2H, C$H^{8/8'}$), 7.10 – 7.03 (m, 4H, C$H^{9/9'}$ overlapping with CH^4), 6.79 (dd, *J* = 7.8, 1.9 Hz, 2H, C$H^{9/9'}$), 6.64 (dd, *J* = 7.7, 1.9 Hz, 2H, C$H^{8/8'}$), 6.32 (d, *J* = 8.2 Hz, 2H, CH^5), 5.89 (t, *J* = 3.5 Hz, 2H, CH^1), 5.47 (m, 2H, CH^2), 5.42 (t, *J* = 2.6 Hz, 2H, CH^3), 5.14 (q, *J* = 6.6 Hz, 2H, CH^6), 2.22 (s, 6H, CH_3^{10}), 0.72 (d, *J* = 6.5 Hz, 6H, CH_3^7).

^{13}C{^{1}H} NMR (126 MHz, 298 K, THF) δ = 144.3, 140.7, 138.5, 135.7, 133.1, 132.7, 131.0, 130.7, 129.1, 128.9, 127.3, 124.8, 119.7, 118.3, 116.9, 108.4, 96.2, 93.0, 72.6, 21.4, 18.8.

IR (ATR) 3035 (vw), 2969 (w), 2921 (w), 2862 (w), 1578 (w), 1526 (w), 1515 (w), 1441 (w), 1408 (m), 1359 (w), 1318 (vw), 1305 (s), 1296 (s), 1243 (w), 1198 (s), 1173 (s), 1146 (m), 1078 (m), 1068 (s), 1045 (s), 1017 (s), 976 (m), 856 (m), 820 (m), 788 (s), 718 (vs), 678 (s), 592 (m-s), 526 (m), 472 (s).

UV/VIS (THF): λ_{max} (log ε) = 352 (3.77), 460 (3.97) nm.

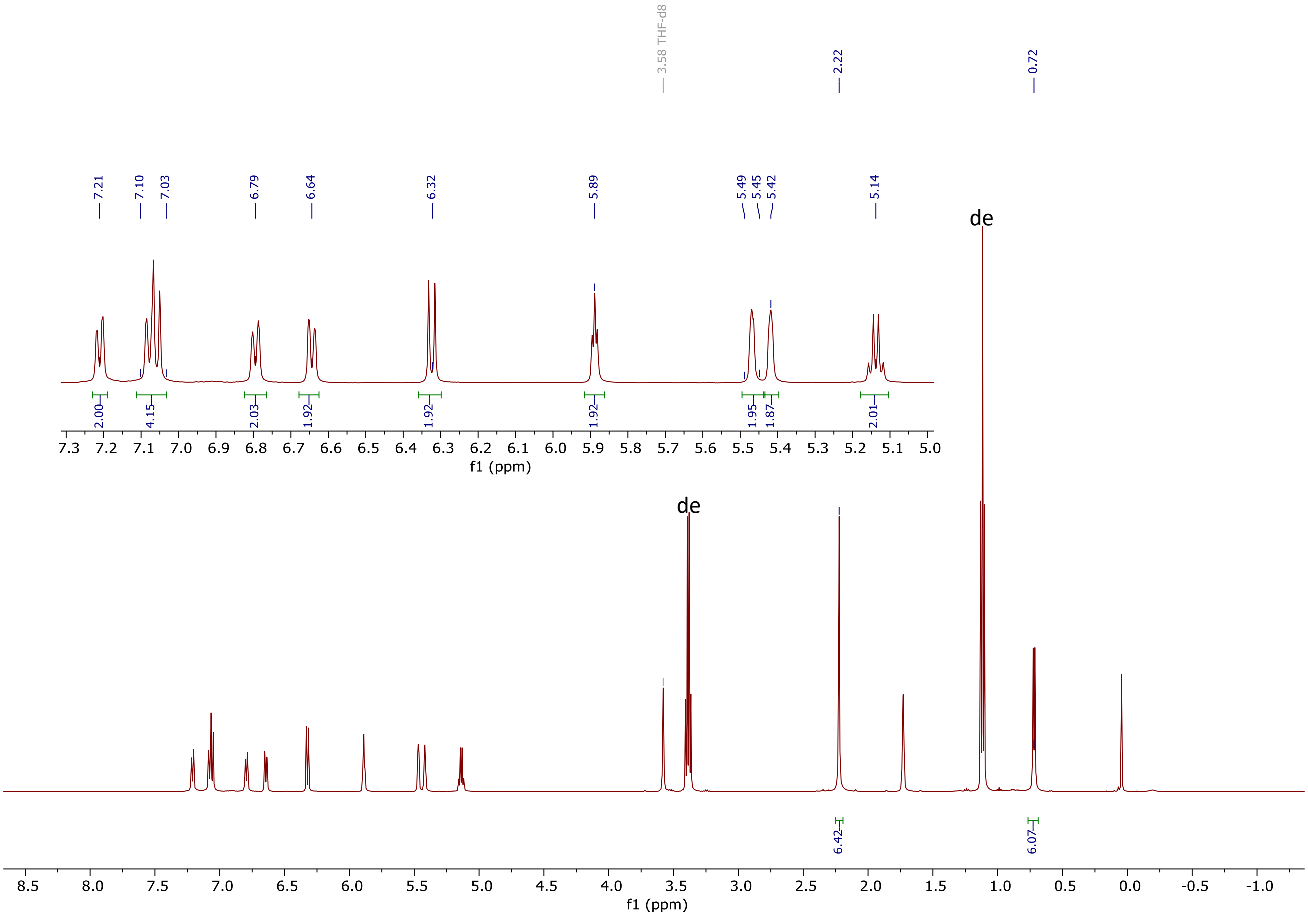

Figure S63: ^{1}H NMR of (-)-(*M,R,R*)-**19** (500 MHz, 298 K, THF-d_8) (de = diethyl ether)

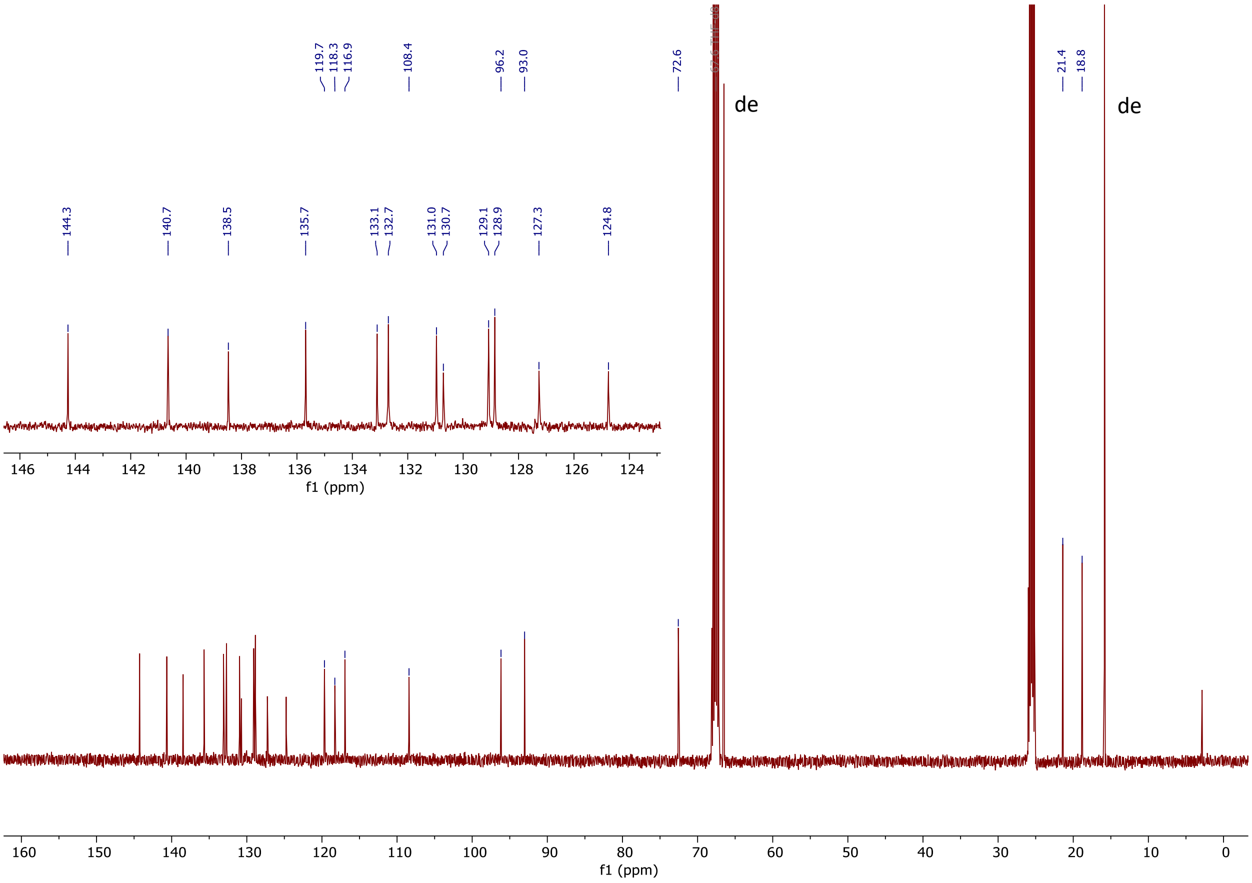

Figure S64: ^{13}C NMR of (-)-(*M,R,R*)-**19** (126 MHz, 298 K, THF-d_8) (de = diethyl ether)

Synthesis of *ansa*-ferrocene (*M,R,R,S_p,S_p*)-**7**

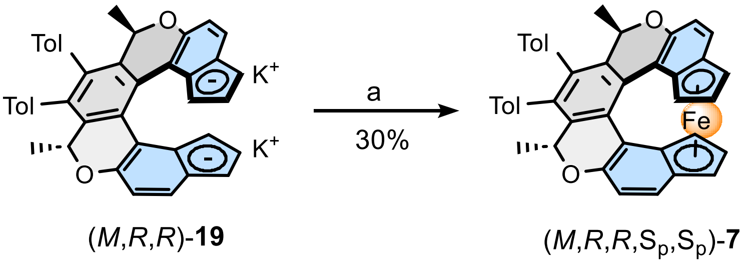

(a) FeCl2 (1.0 equiv.), THF, -20 °C to rt, 18 h, 30%.

Compound (*M,R,R,S_p,S_p*)-**7**

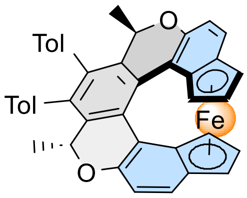

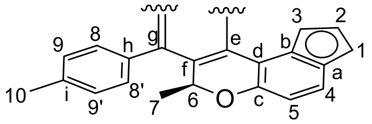

In an Ar glovebox the helicene dipotassium salt (-)-(*M,R,R*)-**19** (135 mg, 0.156 mmol, 1.0 equiv) was weighed into a 10 mL vial and dissolved in 3 mL of THF. The solution was cooled to -20 °C in the glovebox freezer. Similarly, $FeCl_2$ (19.8 mg, 0.156 mmol, 1.0 equiv) was suspended in 3 mL of THF and cooled. Then the solution of the ligand was added dropwise to the stirred solution of $FeCl_2$. The reaction vial was sealed and the mixture was allowed to stir overnight (18h). The mixture was transferred into a small Schlenk and the volatiles were removed under reduced pressure. The residue was dissolved in pentane (8 mL) and the solids were removed by filtration over diatomaceous earth. The pentane solution was cooled to -20 °C and left overnight to crystallize. The crystals were collected by decantation and dried to give (*M,R,R,S_p,S_p*)-**7** as 29.3 mg (30 %) of air sensitive green crystalline solid containing approx. 0.4 equivalent of pentane. Crystals suitable for X-ray diffraction were grown by cooling of a saturated pentane solution of the complex.

^{1}H NMR (500 MHz, 298 K, TDF-*d_8*) δ = 7.35 (d, *J* = 9.0 Hz, 2H, C*H^4*), 7.19 (dd, *J* = 7.7, 1.9 Hz, 2H, C$H^{8/8'}$), 7.12 (dd, *J* = 7.8, 1.9 Hz, 2H, C*$H^{9/9'}$*), 6.96 (dd, *J* = 7.9, 2.0 Hz, 2H, C*$H^{9/9'}$*), 6.91 (dd, *J* = 7.6, 1.9 Hz, 2H, C$H^{8/8'}$), 6.85 (d, *J* = 9.0 Hz, 2H, C*H^5*), 5.28 (q, *J* = 6.6 Hz, 2H, C*H^6*), 4.54 (dd, *J* = 2.5, 1.1 Hz, 2H, C*H^3*), 3.76 (t, *J* = 2.5 Hz, 2H, C*H^2*), 2.82 (dt, *J* = 2.2, 0.9 Hz, 2H, C*H^1*), 2.26 (s, 6H, C*$H_3{}^{10}$*), 1.24 (d, *J* = 6.6 Hz, 6H, C*$H_3{}^7$*).

^{13}C{^{1}H} NMR (126 MHz, 298 K, TDF-*d_8*) δ = 150.3 (*C^c*), 138.2 (*C^f*), 137.1 (*C^g*), 136.9 (*C^i*), 136.5 (*C^h*), 133.5 (*C*H^4), 131.8 (*C*$H^{8/8'}$), 130.2 (*C*$H^{8/8'}$), 129.3 (*C*$H^{9/9'}$), 129.2 (*C*$H^{9/9'}$), 123.6 (*C^e*), 120.8 (*C*H^5), 119.1 (*C^d*), 83.8 (*C^a*), 83.1 (*C^b*), 74.7 (*C*H^2), 73.6 (*C*H^6), 69.5 (*C*H^1), 63.2 (*C*H^3), 21.2 (*CH_3*^{10}), 18.0 (*CH_3*^{7}).

IR (ATR) 2948 (w), 2914 (w), 2847 (w), 1596 (m), 1516 (w), 1511 (w-m), 1454 (w), 1437 (w), 1421 (m), 1385 (w), 1365 (m), 1326 (w), 1299 (w), 1256 (m), 1240 (m), 1197 (m), 1167 (w), 1149 (w), 1133 (w), 1099 (w), 1070 (w-m), 1052 (m), 1027 (m), 1013 (m), 994 (w), 975 (w), 902 (w), 974 (w), 857 (w), 841 (w), 836 (w), 820 (m), 808 (w), 788 (s), 766 (w), 751 (m), 720 (w-m), 697 (w), 673 /m), 636 (w), 587 (w), 551 (m), 522 (s).

UV/VIS (THF): λ_{max} (log ε) = 340 (3.92), 408 (3.66), 670 (2.90) nm.

EA % Calcd for $C_{42}H_{32}O_2Fe$: C, 80.77; H, 5.16. Found: C, 80.56; H, 5.11

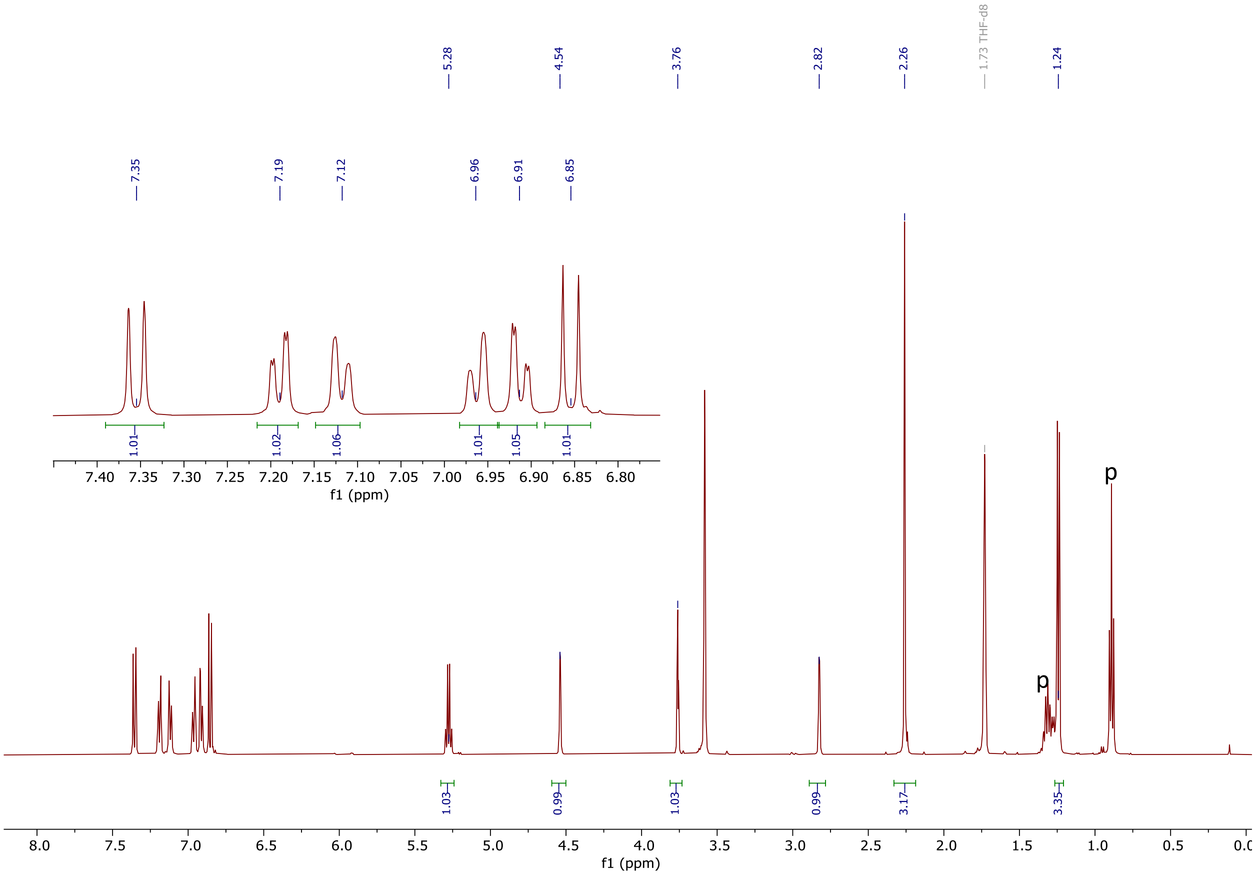

Figure S65: ^{1}H NMR of (M,R,R,S_p,S_p)**-7** (500 MHz, 298 K, TDF-d_8) (p = pentane)

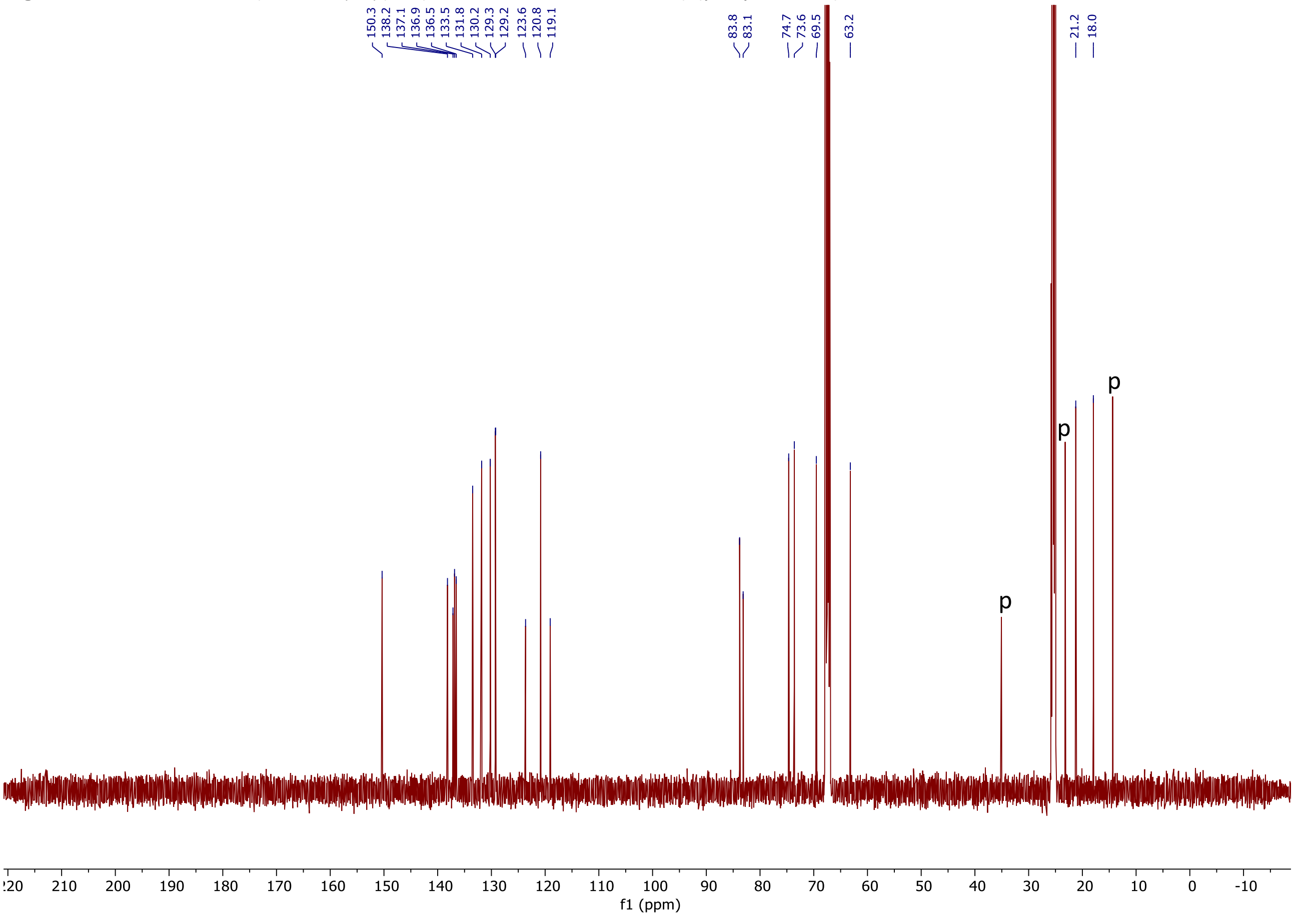

Figure S66: ^{13}C NMR of (M,R,R,S_p,S_p)**-7** (126 MHz, 298 K, TDF-d_8) (p = pentane)

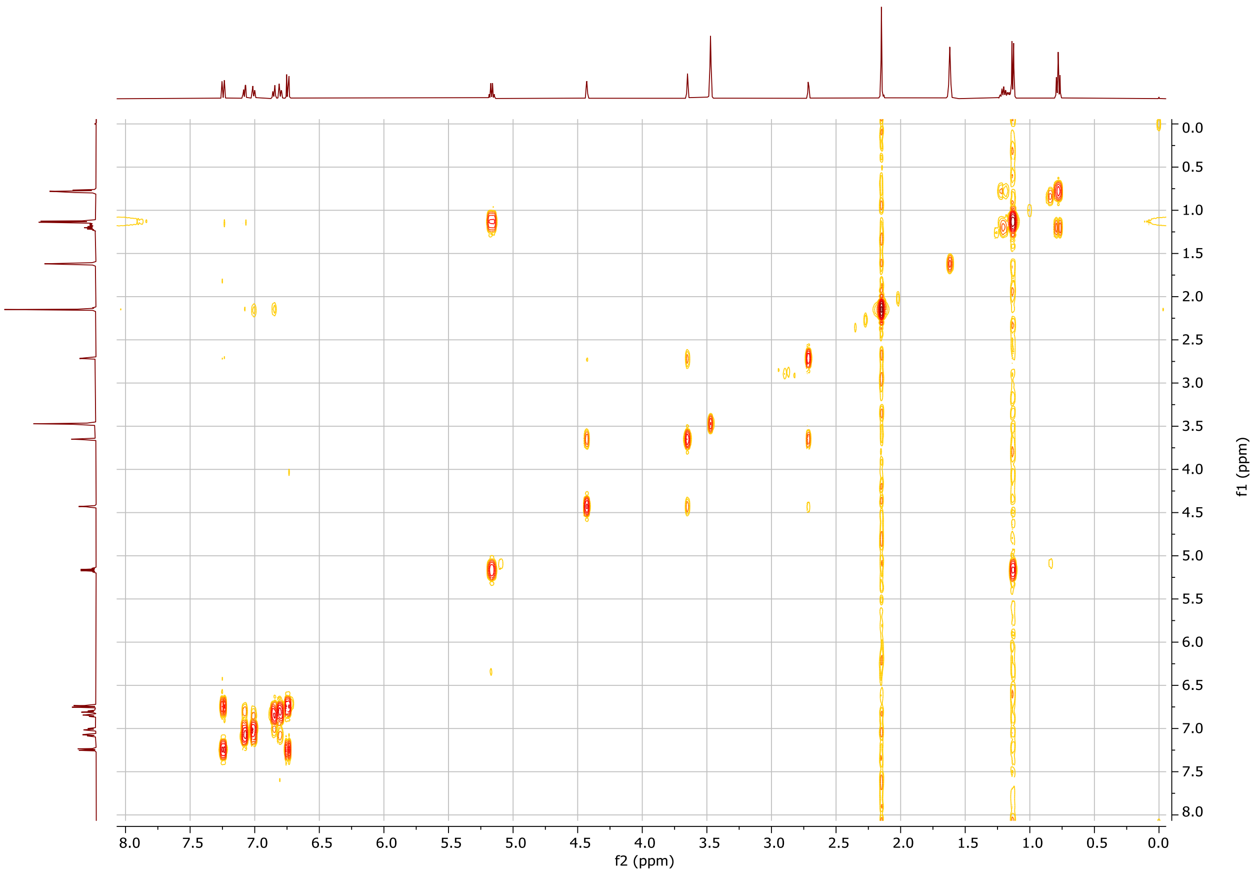

Figure S67: ^{1}H-^{1}H COSY of (*M*,*R*,*R*,*S*$_p$,*S*$_p$)-**7** (500 MHz / 500 MHz, 298 K, TDF-*d*$_8$)

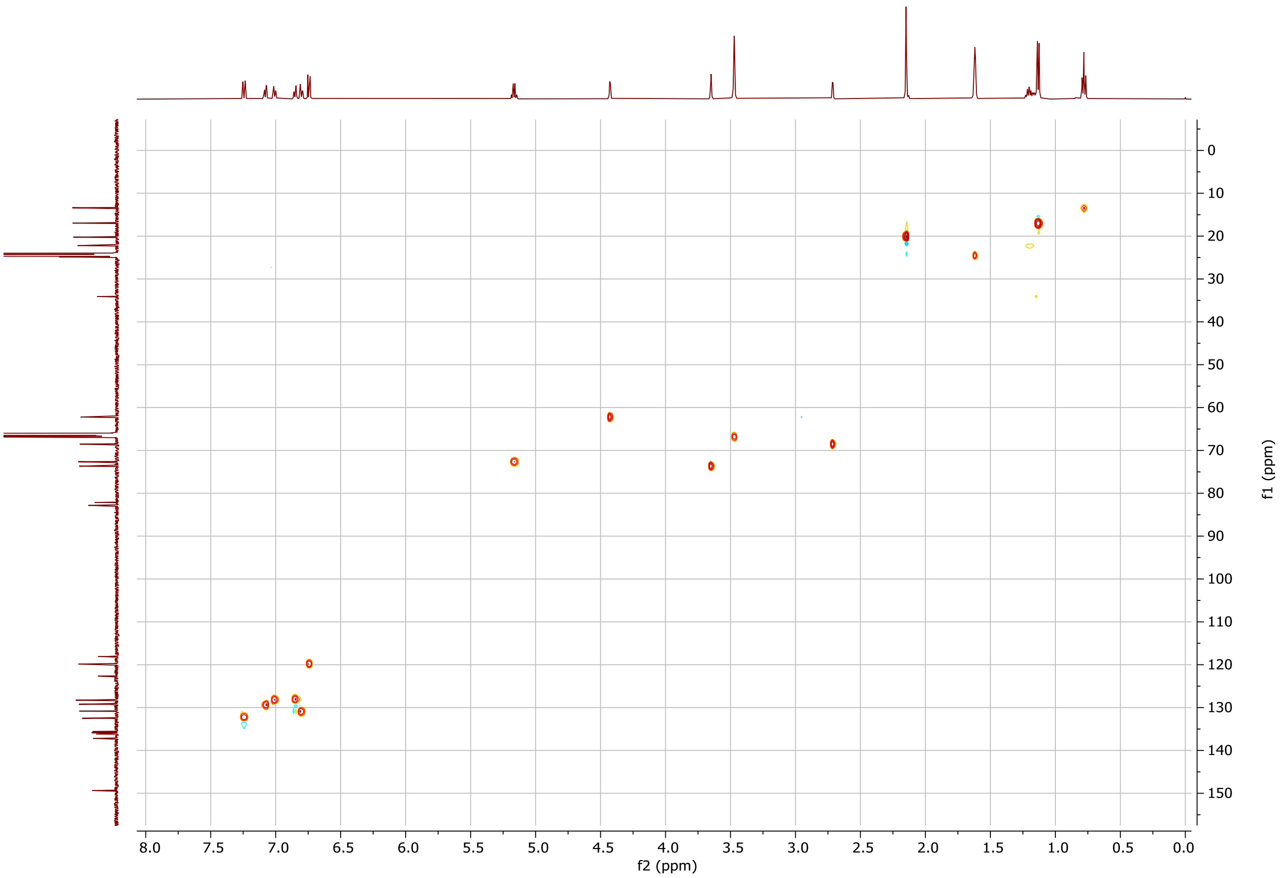

Figure S68: ^{1}H-^{13}C HSQC of (*M*,*R*,*R*,*S*$_p$,*S*$_p$)-**7** (500 MHz / 126 MHz, 298 K, TDF-*d*$_8$))

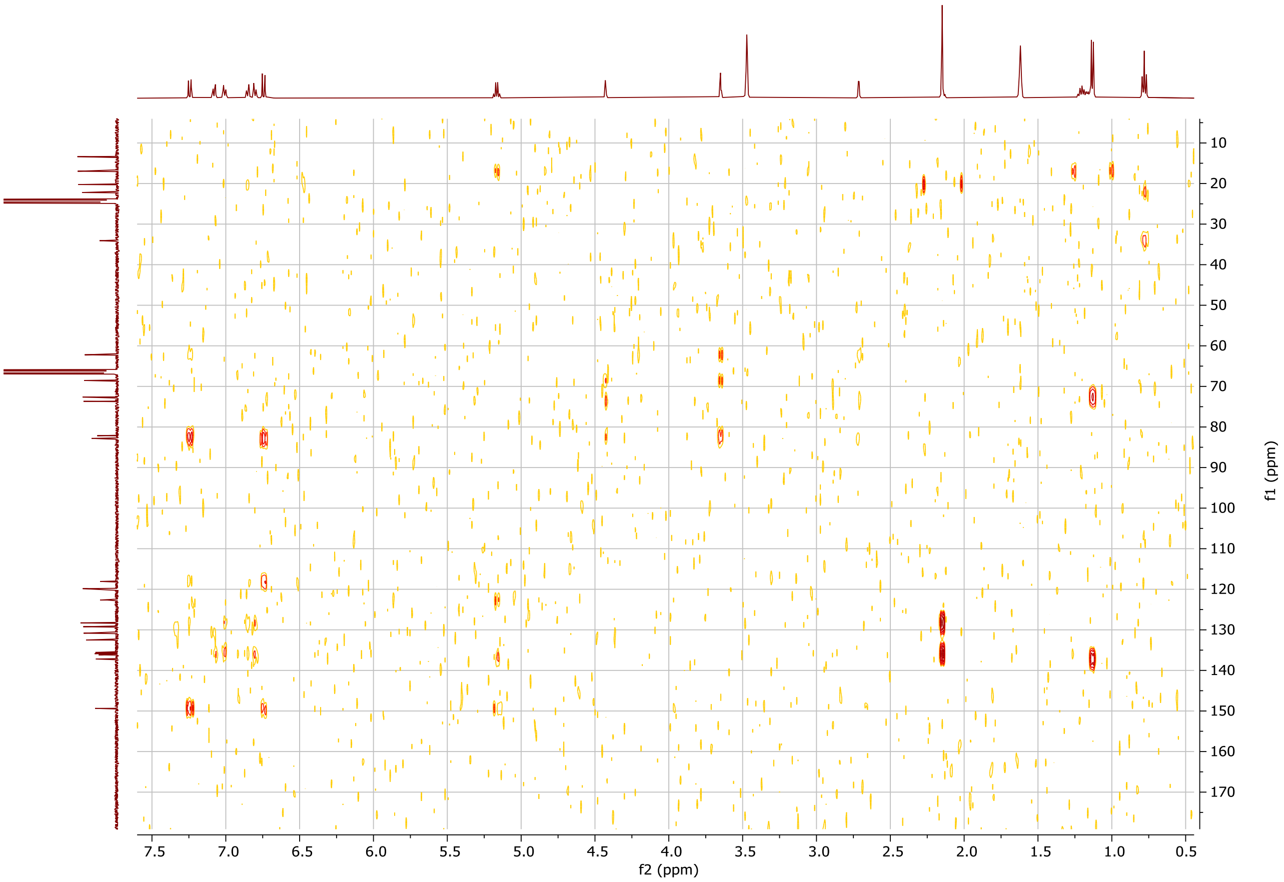

Figure S69: ^{1}H-^{13}C HMBC of (*M,R,R,S$_p$,S$_p$*)**-7** (500 MHz / 126 MHz, 298 K, TDF-d_8)

Synthesis of Rh(I) and Rh(III) complexes

General procedures

General procedure for the synthesis of Cp^{x}Rh(I)(cod) complexes (GP2)

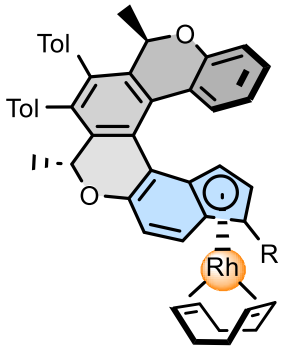

In a glovebox under argon atmosphere, the substituted helicene proligand (1.0 equiv.), $[Rh(cod)Cl]_2$ (0.55 equiv.), and potassium *tert*-butoxide (1.3 equiv.) were weighed into a 10 mL crimp top vial containing a small stir bar. Dry THF was added (10 mL/mmol) and the reaction was allowed to stir at room temperature for 2-4 h. The solvent was evaporated and the residue was purified by fast filtration through a short column on silica gel (pentane:toluene 1:1). The yellow colored fractions were collected and evaporated under reduced pressure to give Cp^{x}Rhodium(I) complexes.

General procedure for the synthesis of Cp^{x}Rh(III)I_2 complexes (GP3)

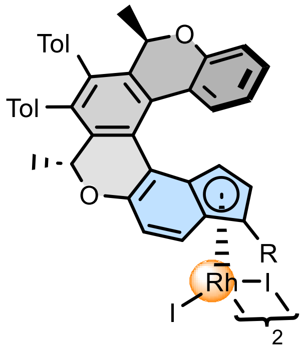

Cp^{x}Rhodium(I) complex was weighed into a small round bottom flask and dissolved in diethyl ether (50 mL/mmol). The solution was cooled to 0 °C and I_2 (2.5 equiv.) was added. The reaction mixture was allowed to stir overnight (14-18 h). The precipitate formed was filtered through a grade 4 frit and washed with ether and pentane (20 mL total). The solid was collected by dissolving in DCM (20 mL). The volatiles were removed under reduced pressure to give the product as dark solid. If necessary, the product was further purified by reprecipitation from DCM/pentane.

General procedure for the synthesis of Cp^{x}Rh(III)Br_2 complexes (GP4)

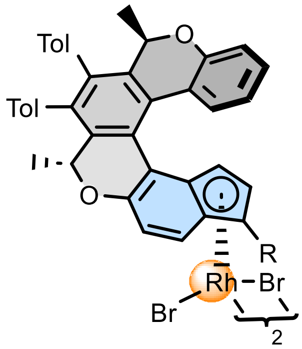

Cp^{x}Rhodium(I) complex was weighed into a small round bottom flask and dissolved in pentane (50 mL/mmol). The solution was cooled to 0 °C and Br_2 (2.5 equiv.) was added by a microsyringe. The reaction mixture was allowed to stir overnight (14-18 h). The precipitate formed was filtered through a grade 4 frit and washed with pentane (20 mL total). The solid was collected by dissolving in DCM (20 mL). The volatiles were removed under reduced pressure to give the product as dark red solid. If necessary, the product was further purified by reprecipitation from DCM/pentane.

Oxa[6]helicene complexes of Rh(I) and Rh(III) (-)-(M,R,R,R_p)-**22a**-**e**, (+)-(P,S,S,S_p)-**22a**-**e**, and (-)-(M,R,R,R_p)-**25a**-**e**.

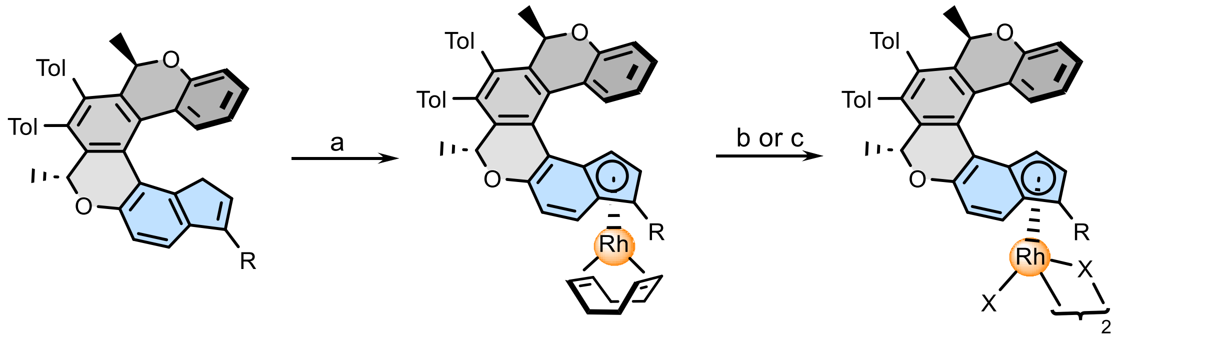

(-)-(M,R,R)-**14a-e**

(-)-(M,R,R,R_p)-**22a**–e
a : R = Me, 69%
b: R = Et, 70%
c : R = Ph, 58%
d: R = o-MeC$_6$H$_4$, 81%
e : R = p-MeOC$_6$H$_4$, 73%

(+)-(P,S,S,S_p)-**22a**–e
a: R = Me, 70%
d: R = o-MeC$_6$H$_4$, 78%
e: R = p-MeOC$_6$H$_4$, 74%

(-)-(M,R,R,R_p)-**25a-e**
a : R = Me, X = I, 97%
a : R = Me, X = Br, 85%
b : R = Et, X = Br, 97%
c : R = Ph, X = Br, 78%
d: R = o-MeC$_6$H$_4$, X = Br, 74%
e : R = p-MeOC$_6$H$_4$, X = I, 79%

(a) [Rh(cod)Cl]$_2$ (0.55 equiv.), t-BuOK (1.3 equiv.), THF, rt, 2-4 h; (b) I$_2$ (2.5 equiv.), Et$_2$O, 0 °C, 14–18 h; (c) Br$_2$ (2.5 equiv.), pentane, 0 °C, 14–18 h.

Compound (+)-(P,S,S,S_p)-**22a**

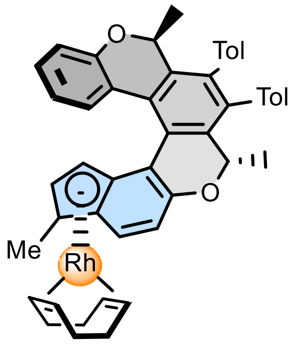

Prepared according to **GP2** from substituted oxa[6]helicene (+)-(P,S,S)-**14a** (50 mg, 0.09 mmol, 1.0 equiv.), $[Rh(cod)Cl]_2$ (24.8 mg, 0.05 mmol, 0.55 equiv.), and potassium *tert*-butoxide (13.3 mg, 0.12 mmol, 1.3 equiv.) providing (+)-(P,S,S,S_p)-**22a** (48.3 mg, 70%) as an orange amorphous solid.

R$_f$ = 0.68 (pentane:toluene 1:1).

$[\alpha]^{20}_D$ +1812.6 (*c* 0.198, THF).

^{1}H NMR (500 MHz, 298 K, $CDCl_3$): δ = 7.19 – 7.15 (m, 2H, *o*-tolyl C*H*), 7.12 – 7.04 (m, 4H, *m*-protons of *p*-tolyl overlapping 2 CH^{Ar}), 7.02 – 6.96 (m, 3H, 3 C*H* Ar), 6.90 – 6.85 (m, 2H, *m*-protons of *p*-tolyl), 6.71 (dd, *J* = 7.8, 1.9 Hz, 1H, *o*-protons of *p*-tolyl), 6.68 (dd, *J* = 7.7, 1.9 Hz, 1H, *o*-protons of *p*-tolyl), 6.54 (td, *J* = 7.5, 1.3 Hz, 1H, CH^{Ar}), 5.52 (t, *J* = 2.3 Hz, 1H, Cp*H*), 5.31 – 5.21 (m, 2H, C*H* chiral center), 4.17 (d, *J* = 2.7 Hz, 1H, Cp*H*), 3.73 – 3.67 (m, 2H, C*H* COD), 3.31 (tdd, *J* = 8.1, 4.6, 2.7 Hz, 2H, C*H* COD), 2.27 (s, 3H, CH_3 tolyl), 2.26 (s, 3H, CH_3 tolyl), 1.99 – 1.86 (m, 4H, CH_2 COD), 1.85 (s, 3H, CpCH_3), 1.69 – 1.60 (m, 2H, CH_2 COD), 1.43 – 1.32 (m, 2H, CH_2 COD), 1.06 (d, *J* = 6.6 Hz, 3H, CH_3 chiral center), 0.97 (d, *J* = 6.6 Hz, 3H, CH_3 chiral center).

^{13}C{^{1}H} NMR (126 MHz, 298 K, $CDCl_3$): δ = 152.5, 150.9, 139.6, 138.6, 136.8, 136.4, 136.04, 136.03, 135.34, 135.27, 131.1, 130.9, 129.4, 129.3, 128.7, 128.6, 128.4 (2C), 128.0, 125.6, 125.2, 125.0, 121.8, 118.9, 118.5, 116.6, 114.2, 111.4 (d, *J* = 2.3 Hz), 109.4 (d, *J* = 2.2 Hz), 92.7 (d, *J* = 5.1 Hz), 88.1 (d, *J* = 4.0 Hz), 74.5 (d, *J* = 4.4 Hz), 73.4, 73.2, 68.9 (d, *J* = 13.4 Hz, 4C), 33.9 (2C), 29.3 (2C), 21.3 (2C), 18.7, 18.6, 10.6.

IR (ATR): 3016 (vw), 2972 (w), 2947 (w), 2915 (m), 2866 (m), 2819 (m), 1603 (w), 1584 (w-m), 1543 (w), 1515 (w-m), 1482 (w), 1442 (m), 1423 (m), 1361 (m), 1317 (m), 1271 (w), 1257 (w), 1227 (w), 1211 (s), 1180 (w), 1148 (m-s), 1119 (w), 1108 (w), 1099 (w), 1084 (w), 1064 (s), 1028 (m), 1003 (m), 984 (m), 953 (w), 939 (w), 908 (w), 856 (m), 831 (m), 790 (m-s), 765 (m-s), 754 (vs), 741 (vs), 680 (m), 634 (m), 626 (w), 524 (m) cm^{-1}.

HRMS (ESI) *m/z*: ($[M]^+$) calcd for $C_{48}H_{45}O_2{}^{103}Rh$ 756.2469, found 756.2463 (Δ = -0.83 ppm).

Compound (-)-(M,R,R,R_p)-**22a**

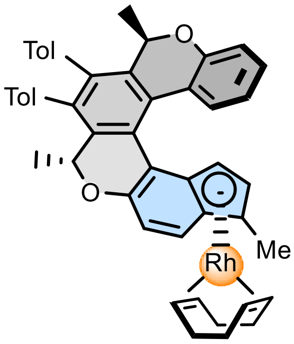

Prepared according to **GP2** from substituted oxa[6]helicene (-)-(M,R,R)-**14a** (100 mg, 0.18 mmol, 1.0 equiv.), $[Rh(cod)Cl]_2$ (49.6 mg, 0.10 mmol, 0.55 equiv.), and potassium *tert*-butoxide (26.6 mg, 0.24 mmol, 1.3 equiv.) providing (-)-(M,R,R,R_p)-**22a** (97 mg, 69%) as an orange amorphous solid containing about 0.3 equivalent of toluene (^{1}H NMR).

$[\alpha]^{20}_D$ -1723.6 (*c* 0.137, THF).

EA: Calcd for $(C_{48}H_{45}O_2Rh)(C_7H_8)_{0.3}$: C, 76.76%; H, 6.10%. Found: C, 77.13%; H, 6.39%.

UV/VIS (THF): λ_{max} (log ε) = 268 (4.47), 372 (4.14) nm.

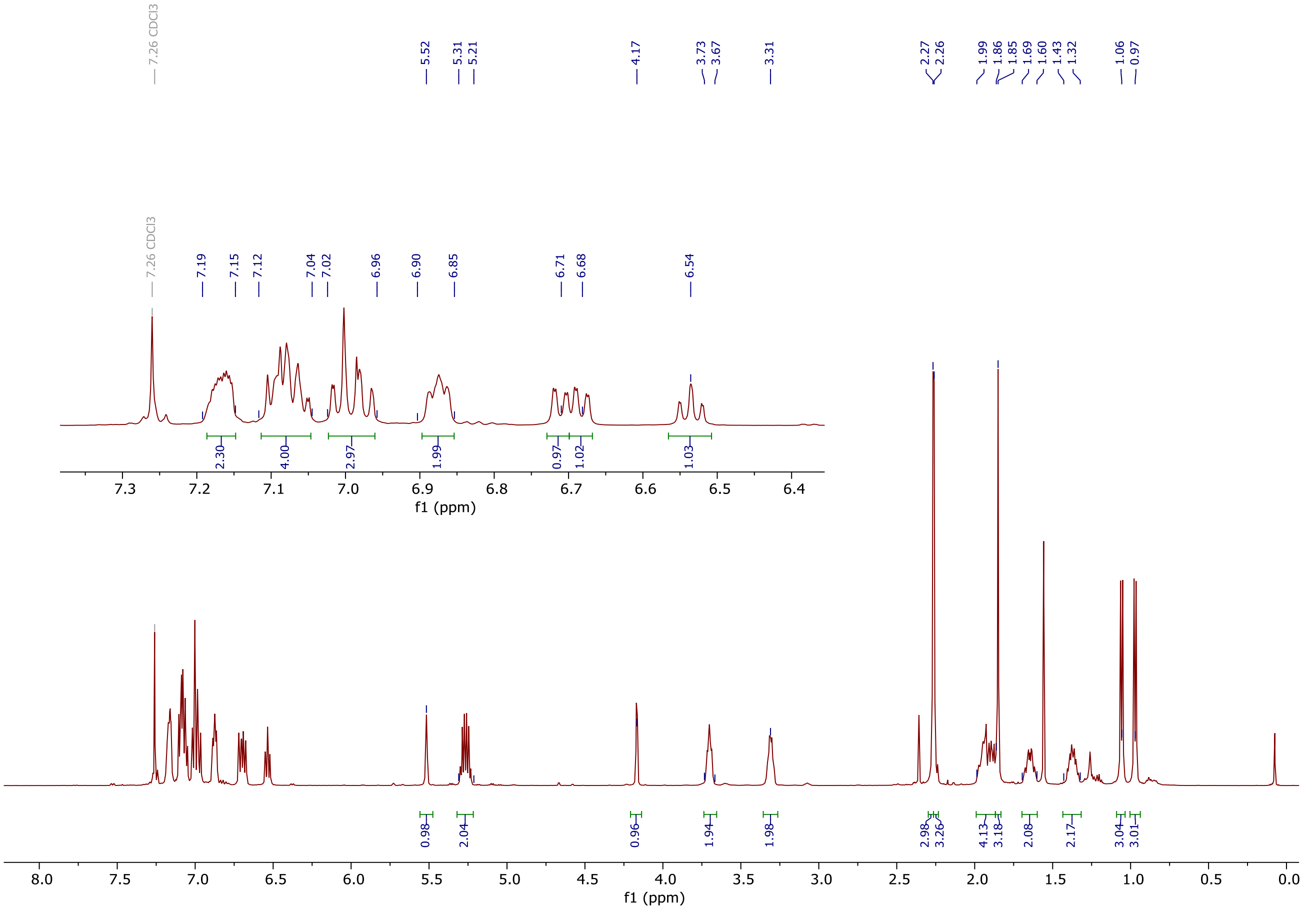

Figure S70: ^{1}H NMR of (+)-(*P,S,S,S$_p$*)-**22a** (500 MHz, 298 K, $CDCl_3$).

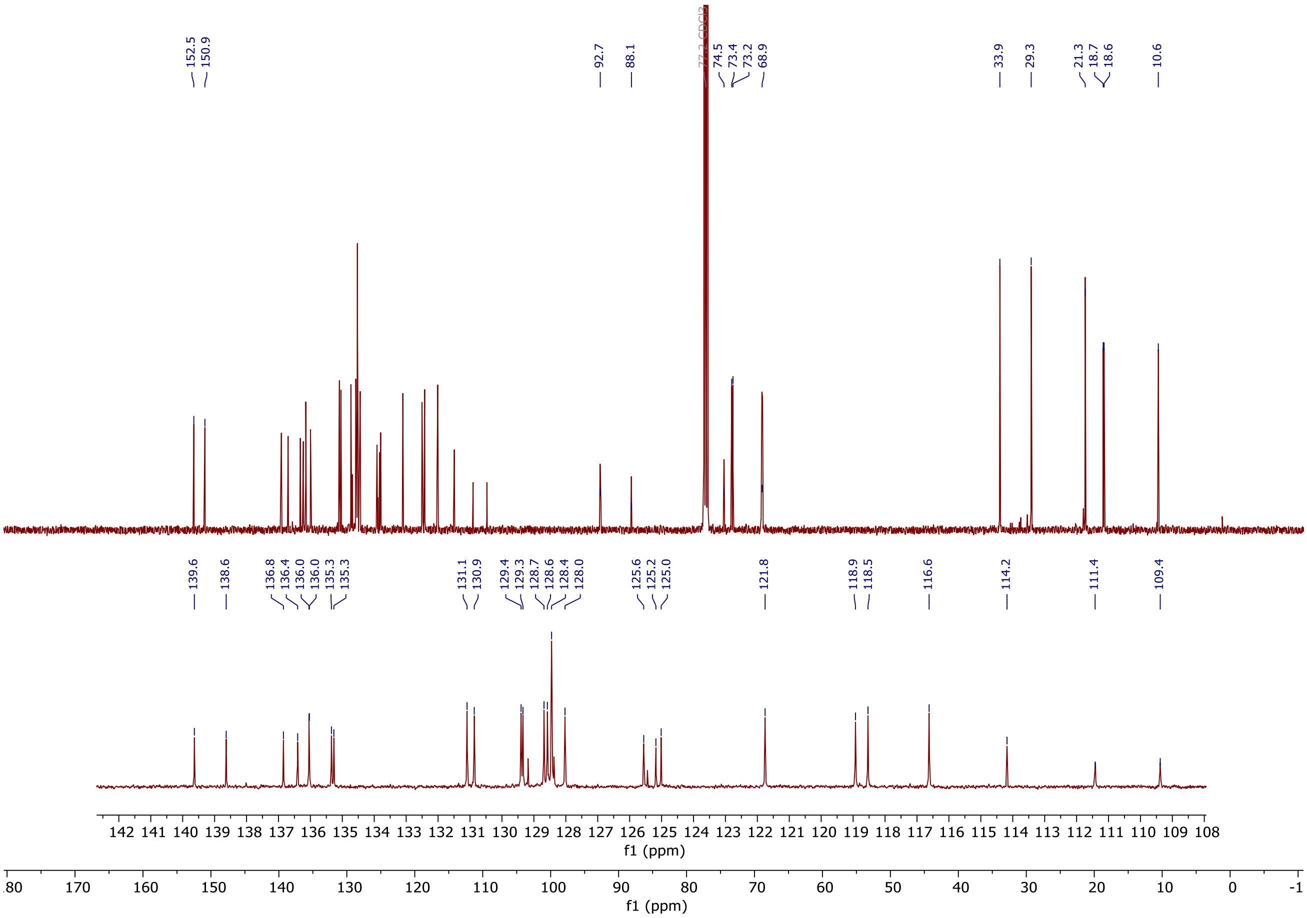

Figure S71: ^{13}C NMR of (+)-(*P,S,S,S$_p$*)-**22a** (126 MHz, 298 K, $CDCl_3$).

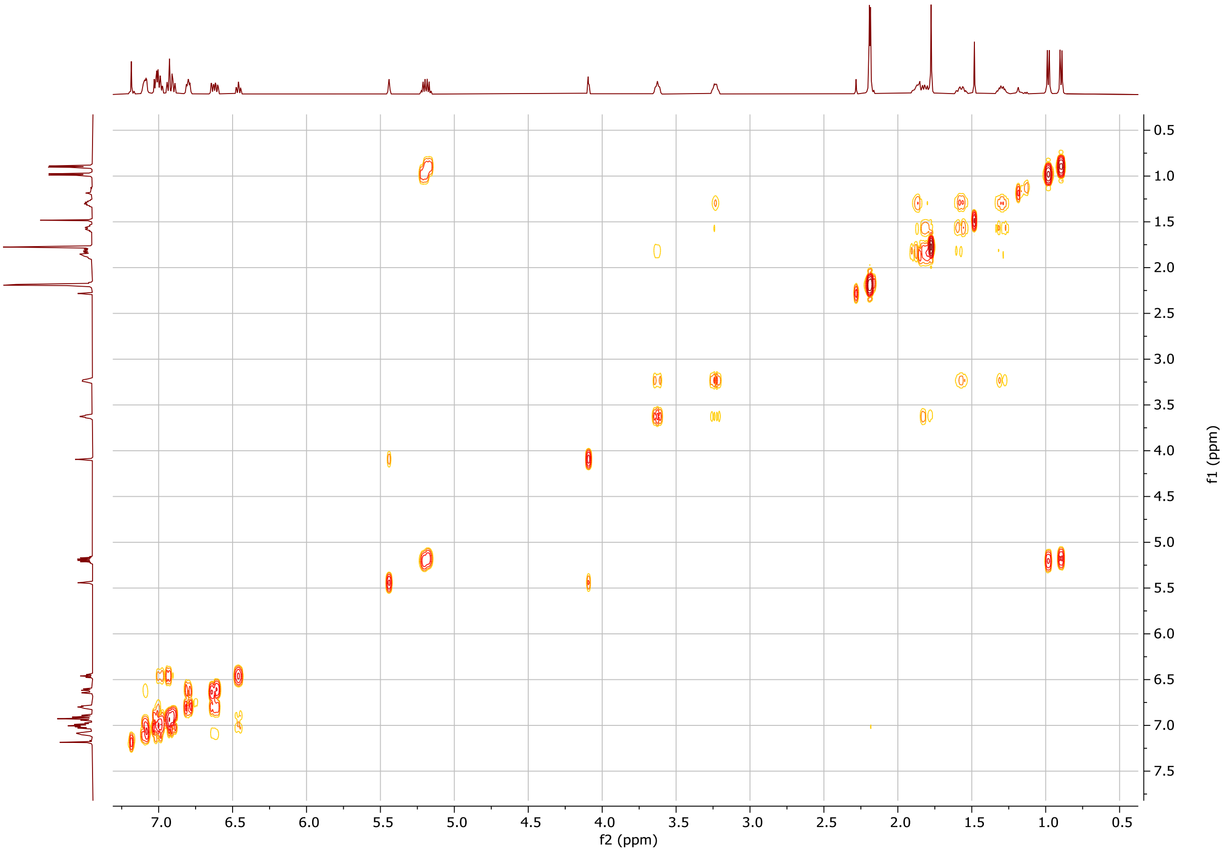

Figure S72: ^{1}H-^{1}H COSY of (+)-(*P,S,S,S$_p$*)-**22a** (500 MHz/500 MHz, 298 K, $CDCl_3$).

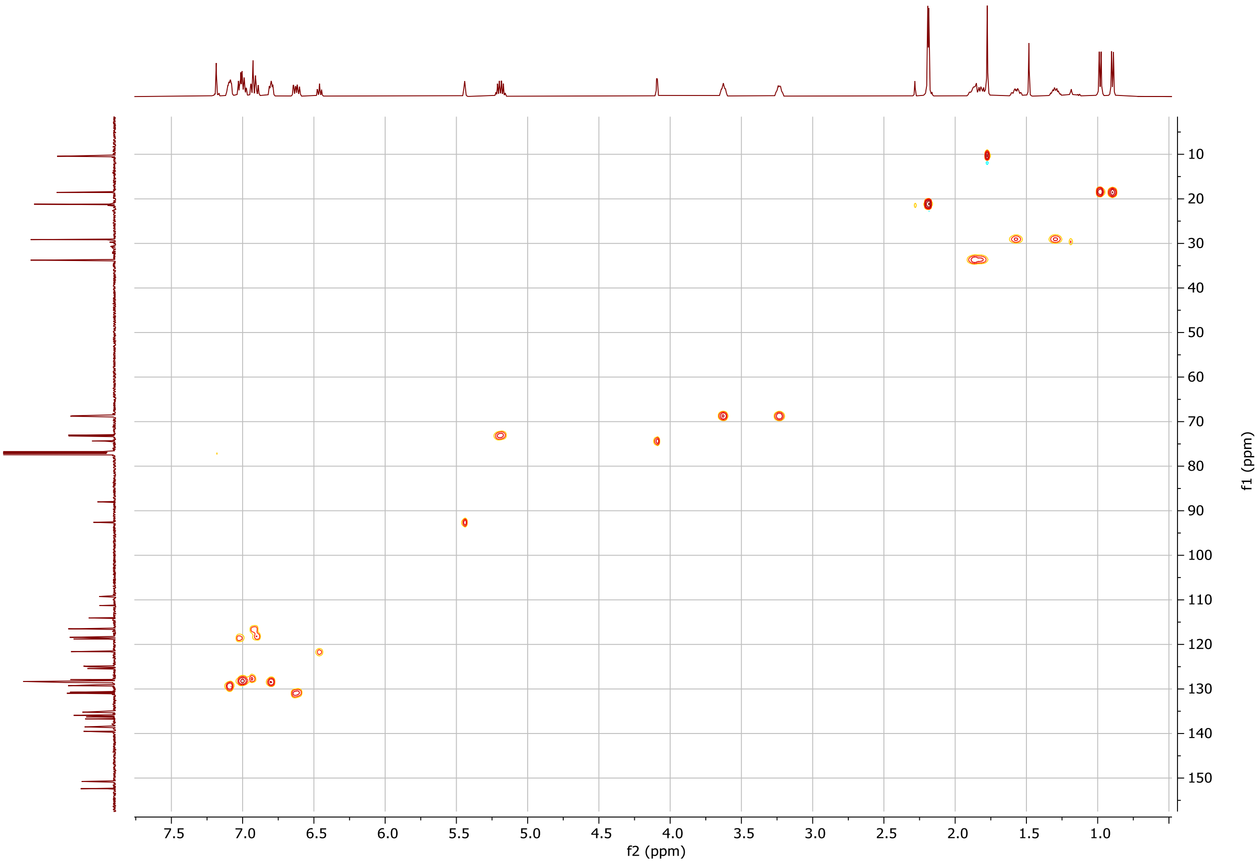

Figure S73: ^{1}H-^{13}C HSQC of (+)-(*P,S,S,S$_p$*)-**22a** (500 MHz/126 MHz, 298 K, $CDCl_3$).

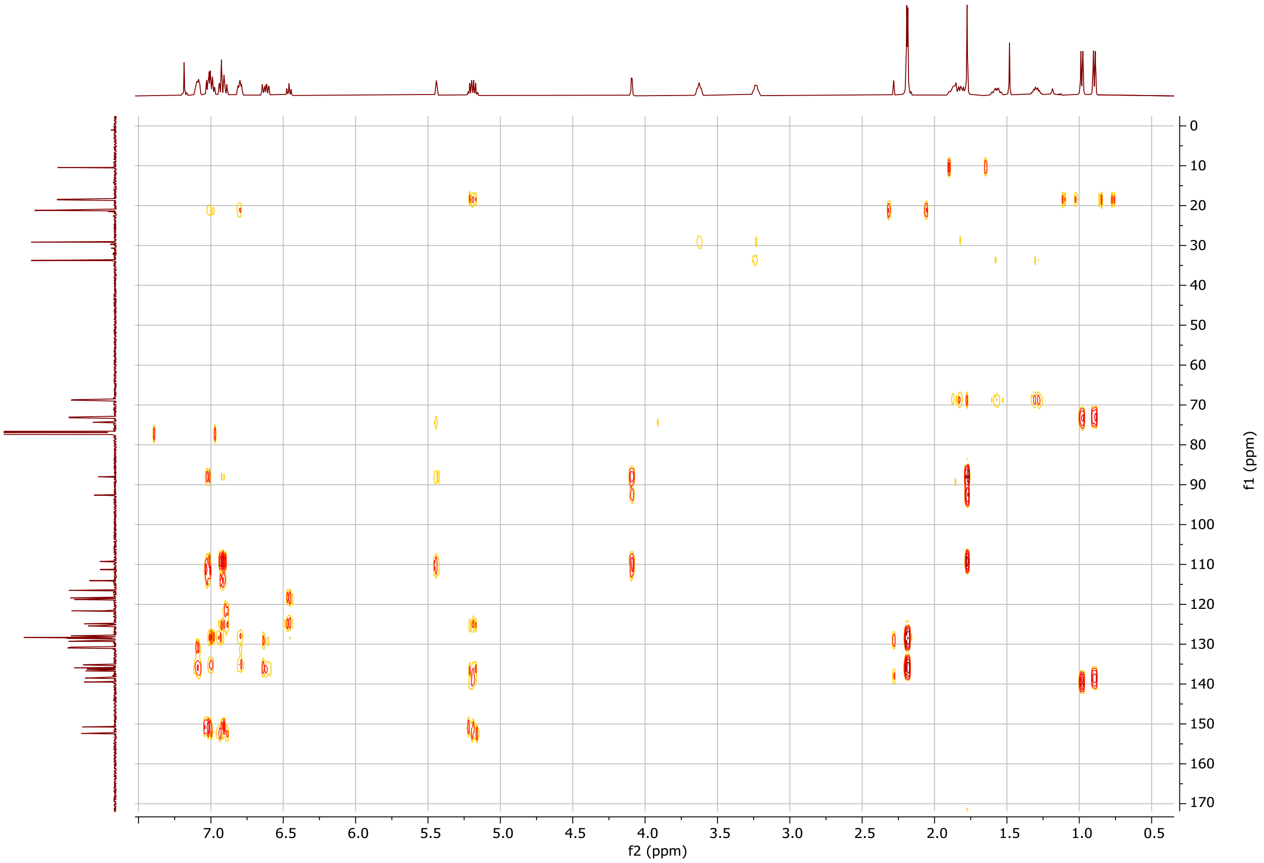

Figure S74: ^{1}H-^{13}C HMBC of (+)-(*P*,*S*,*S*,*S*$_p$)-**22a** (500 MHz/126 MHz, 298 K, $CDCl_3$).

Compound (-)-*(M,R,R,R$_p$)*-**22b**

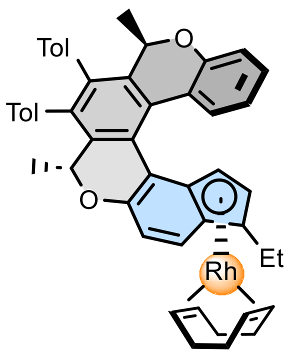

Prepared according to **GP2** from substituted oxa[6]helicene (-)-(*M,R,R*)-**14b** (40 mg, 0.07 mmol, 1.0 equiv.), $[Rh(cod)Cl]_2$ (19.4 mg, 0.04 mmol, 0.55 equiv.), and potassium *tert*-butoxide (10.4 mg, 0.09 mmol, 1.3 equiv.) providing (-)-*(M,R,R,R$_p$)*-**22b** (41 mg, 70%) as an orange amorphous solid containing 0.8 equiv. of toluene.

R$_f$ = 0.74 (pentane:toluene 1:1).

$[\alpha]^{20}_D$ -1502.5 (*c* 0.105, THF).

^{1}H NMR (600 MHz, 298 K, $CDCl_3$): δ 7.20 – 7.15 (m, 2H, *o*-protons of *p*-tolyl overlapping residual toluene signal), 7.11 – 7.04 (m, 4H, 2 *m*-protons of *p*-tolyl overlapping with 2 CH^{Ar}), 7.01 – 6.95 (m, 3H, CH^{Ar}), 6.88 (ddd, *J* = 7.7, 5.6, 1.9 Hz, 2H, *m*-protons of *p*-tolyl), 6.73 – 6.67 (m, 2H, *o*-protons of *p*-tolyl), 6.51 (td, *J* = 7.5, 1.3 Hz, 1H, CH^{Ar}), 5.53 (m, 1H, Cp*H*), 5.30 – 5.23 (m, 2H, C*H* chiral center), 4.15 (d, *J* = 2.8 Hz, 1H, Cp*H*), 3.71 – 3.65 (m, 2H, C*H* COD), 3.35 (m, 2H, C*H* COD), 2.39 – 2.30 (m, 1H, CH_2 ethyl overlapping residual toluene signal), 2.27 (s, 3H, CH_3 tolyl), 2.26 (s, 3H, CH_3 tolyl), 2.10 (dq, *J* = 15.1, 7.5 Hz, 1H, CH_2 ethyl), 1.96 – 1.83 (m, 4H, CH_2 COD), 1.65 (dddd, *J* = 13.7, 10.0, 8.2, 3.6 Hz, 2H, CH_2 COD), 1.42 – 1.35 (m, 2H, CH_2 COD), 1.22 (t, *J* = 7.5 Hz, 3H, CH_3 ethyl), 1.07 (d, *J* = 6.6 Hz, 3H, CH_3 chiral center), 0.98 (d, *J* = 6.7 Hz, 3H, CH_3 chiral center).

^{13}C{^{1}H} NMR (151 MHz, $CDCl_3$): δ = 152.6, 150.9, 139.6, 138.7, 138.0, 136.7, 136.4, 136.1, 136.0, 135.4, 135.3, 131.1, 130.9, 129.43, 129.38, 129.2, 128.7, 128.6, 128.47, 128.46, 128.44, 128.38, 128.1, 125.6, 125.5, 125.2, 125.0, 121.6, 119.0, 118.5, 116.5, 114.3, 111.8 (d, *J* = 2.3 Hz), 108.4 (d, *J* = 2.1 Hz), 95.3 (d, *J* = 3.9 Hz), 90.9 (d, *J* = 5.1 Hz), 74.5 (d, *J* = 4.4 Hz), 73.4, 73.2, 69.1 (d, *J* = 13.1 Hz), 68.4 (d, *J* = 14.0 Hz), 33.8, 29.5, 21.3, 18.8, 18.7, 18.6, 14.0.

HRMS (ESI) *m/z*: ($[M+H]^+$) calcd for $C_{49}H_{48}O_2{}^{103}Rh$ 771.2704, found 771.2706 (Δ = 0.24 ppm).

IR (ATR): 3020 (vw), 2966 (m), 2923 (m), 2867 (m), 2822 (m), 1604 (w), 1585 (w-m), 1546 (w), 1515 (w-m), 1485 (w), 1446 (m), 1425 (m), 1410 (m), 1364 (m), 1324 (w), 1297 (w), 1272 (w), 1228 (m), 1211 (s), 1182 (w), 1182 (s), 1100 (m), 1084 (w), 1066 (s), 1030 (m), 1003 (m-s), 983 (w), 954 (w), 938 (w), 905 (m), 858 (m), 834 (m), 805 (m-s), 792 (m-s), 756 (vs), 742 (vs), 728 (s), 693 (w), 682 (m), 637 (w), 595 (w), 582 (w), 566 (w), 528 (m), 515 (m), 483 (s), 459 (s) cm^{-1}.

EA: Calcd for $(C_{54}H_{49}O_3Rh)(C_7H_8)_{0.8}$: C, 77.47%; H, 6.60%. Found: C, 77.67%; H, 6.52%.

UV/VIS (THF): λ_{max} (log ε) = 270 (4.50), 367 (4.15) nm.

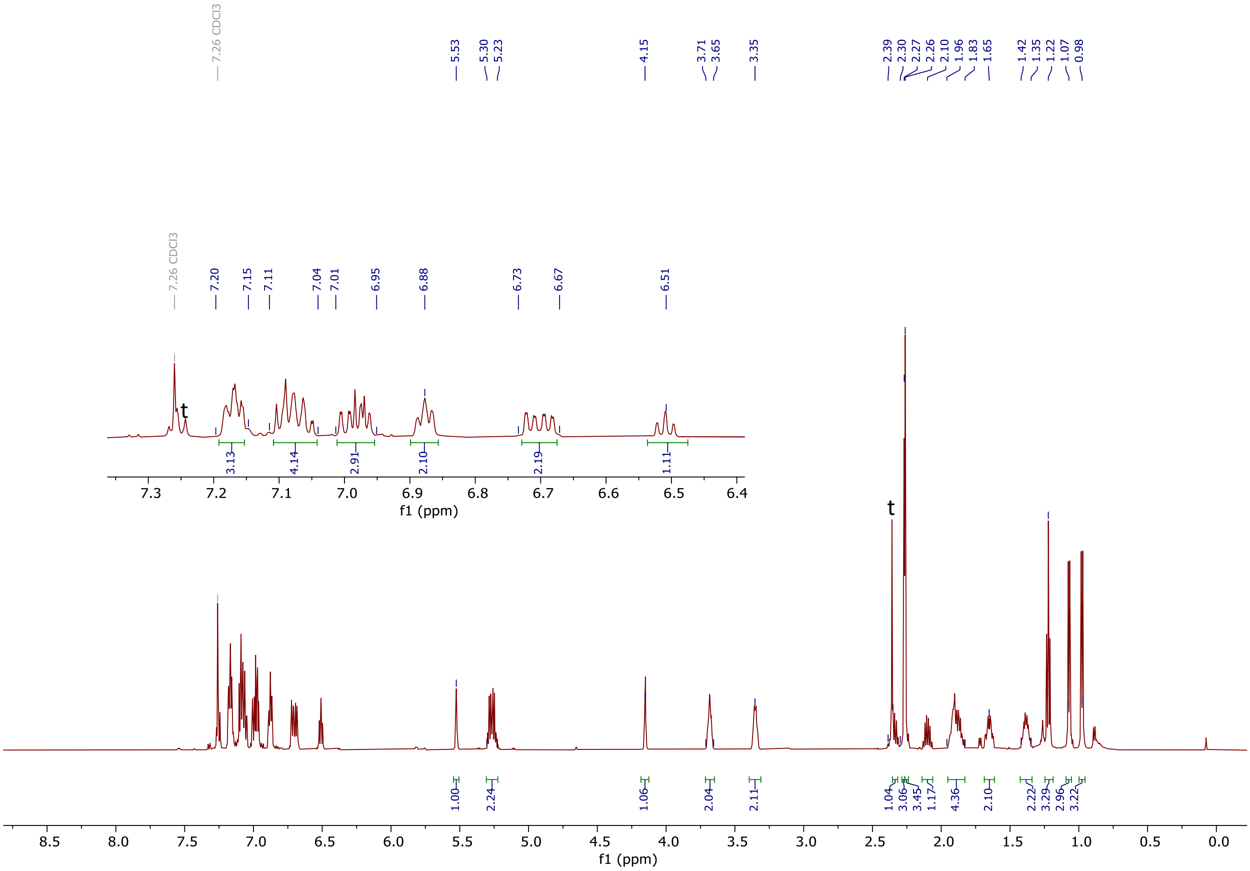

Figure S75: ¹H NMR of (-)-*(M,R,R,R$_p$)*-**22b** (600 MHz, 298 K, $CDCl_3$) (t = toluene).

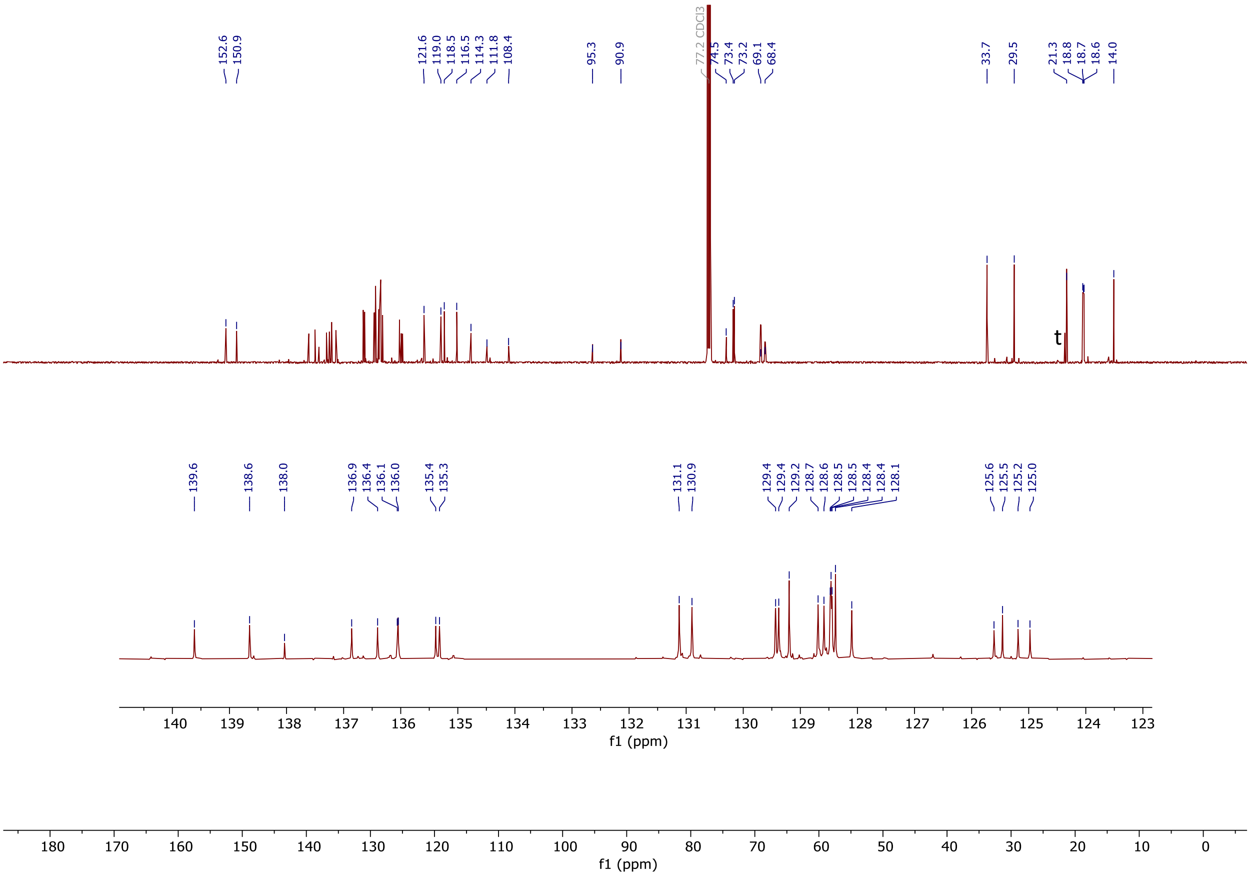

Figure S76: ¹³C NMR of (-)-*(M,R,R,R$_p$)*-**22b** (151 MHz, 298 K, $CDCl_3$) (t = toluene).

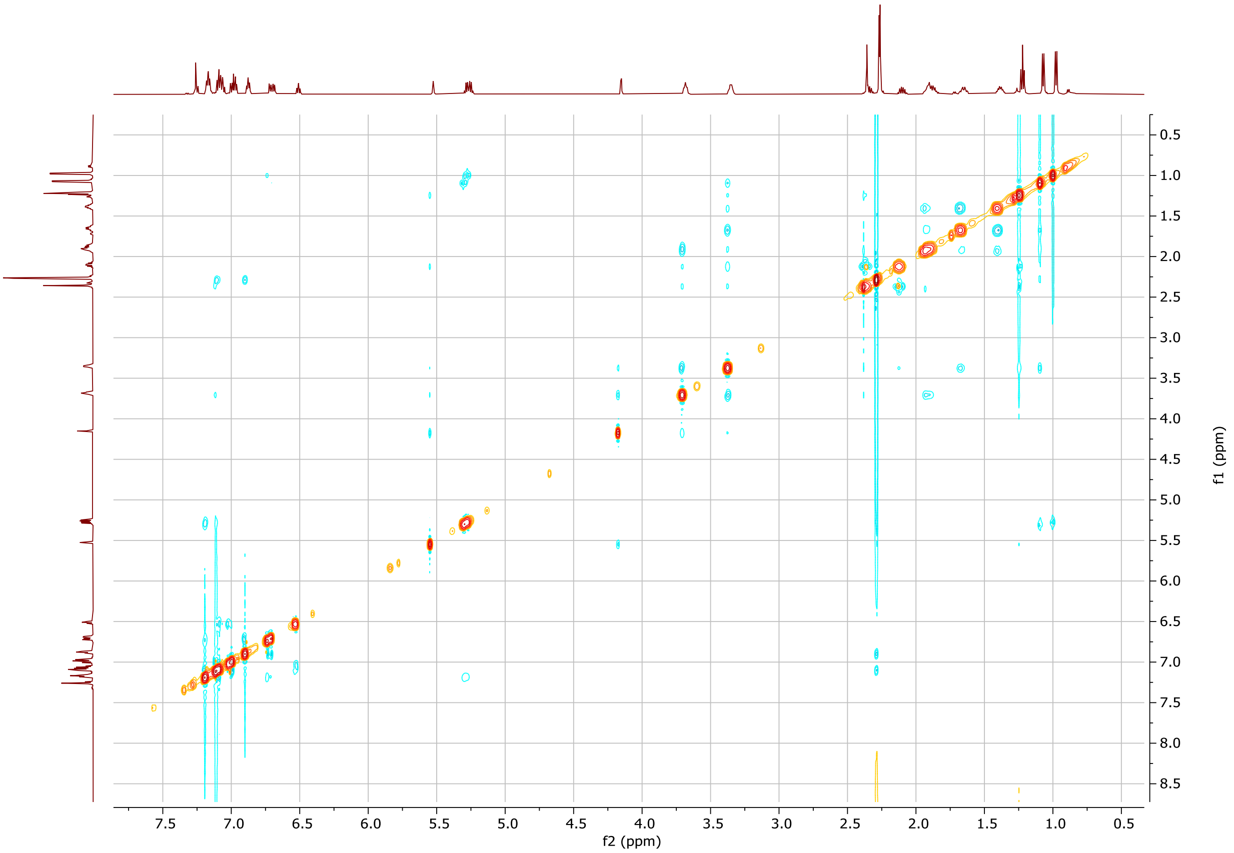

Figure S77: ^{1}H-^{1}H NOESY of (-)-*(M,R,R,R$_p$)*-**22b** (600 MHz/600 MHz, 298 K, $CDCl_3$).

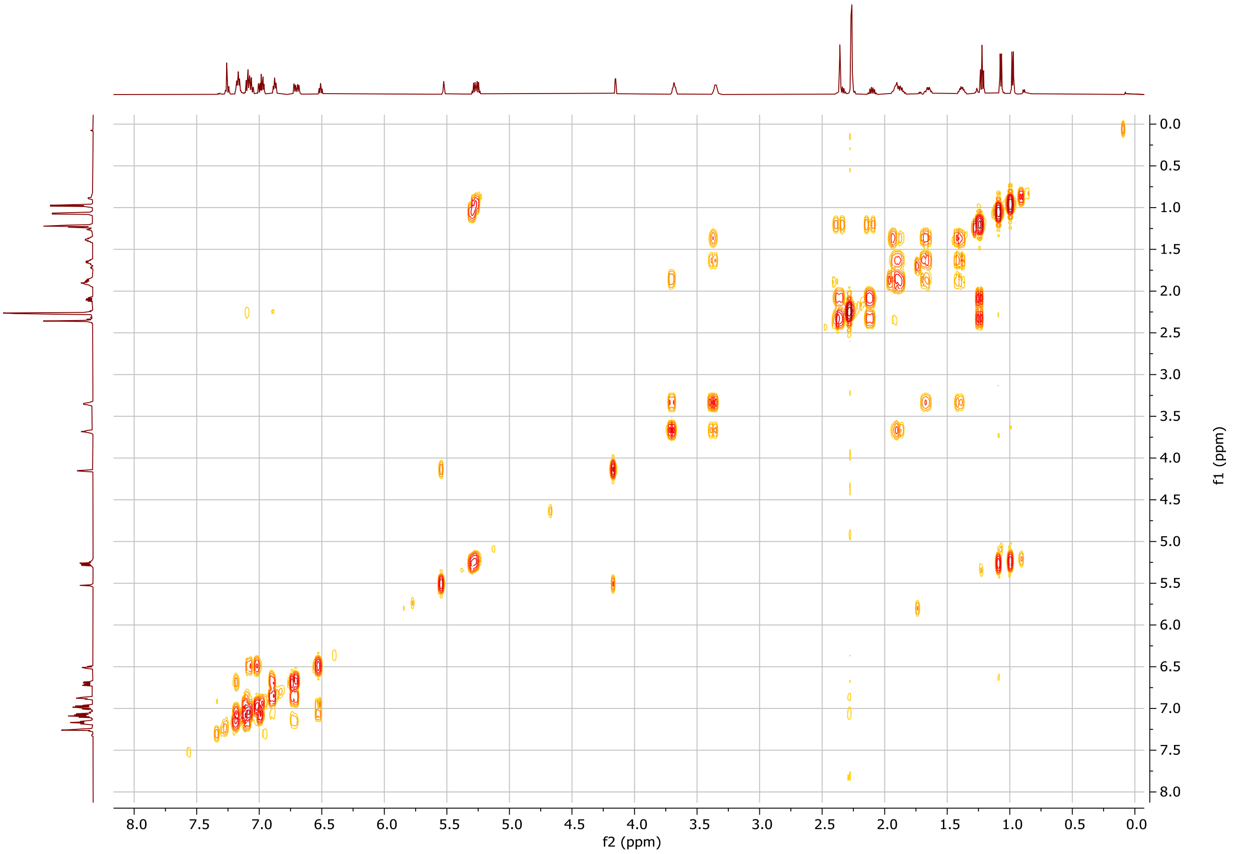

Figure S78: ^{1}H-^{1}H COSY of (-)-*(M,R,R,R$_p$)*-**22b** (600 MHz/600 MHz, 298 K, $CDCl_3$).

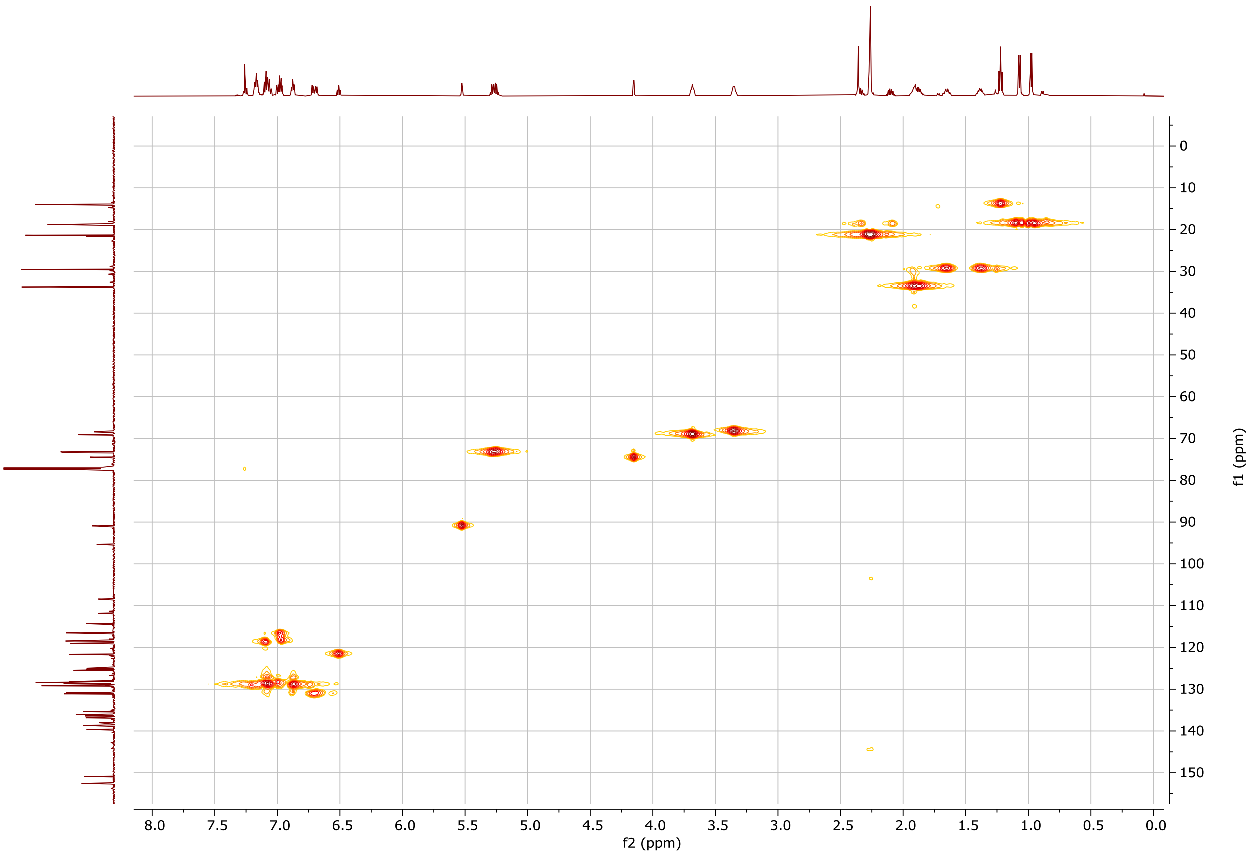

Figure S79: ^{1}H-^{13}C HSQC of (-)-*(M,R,R,R$_p$)*-**22b** (600 MHz/151 MHz, 298 K, CDCl₃).

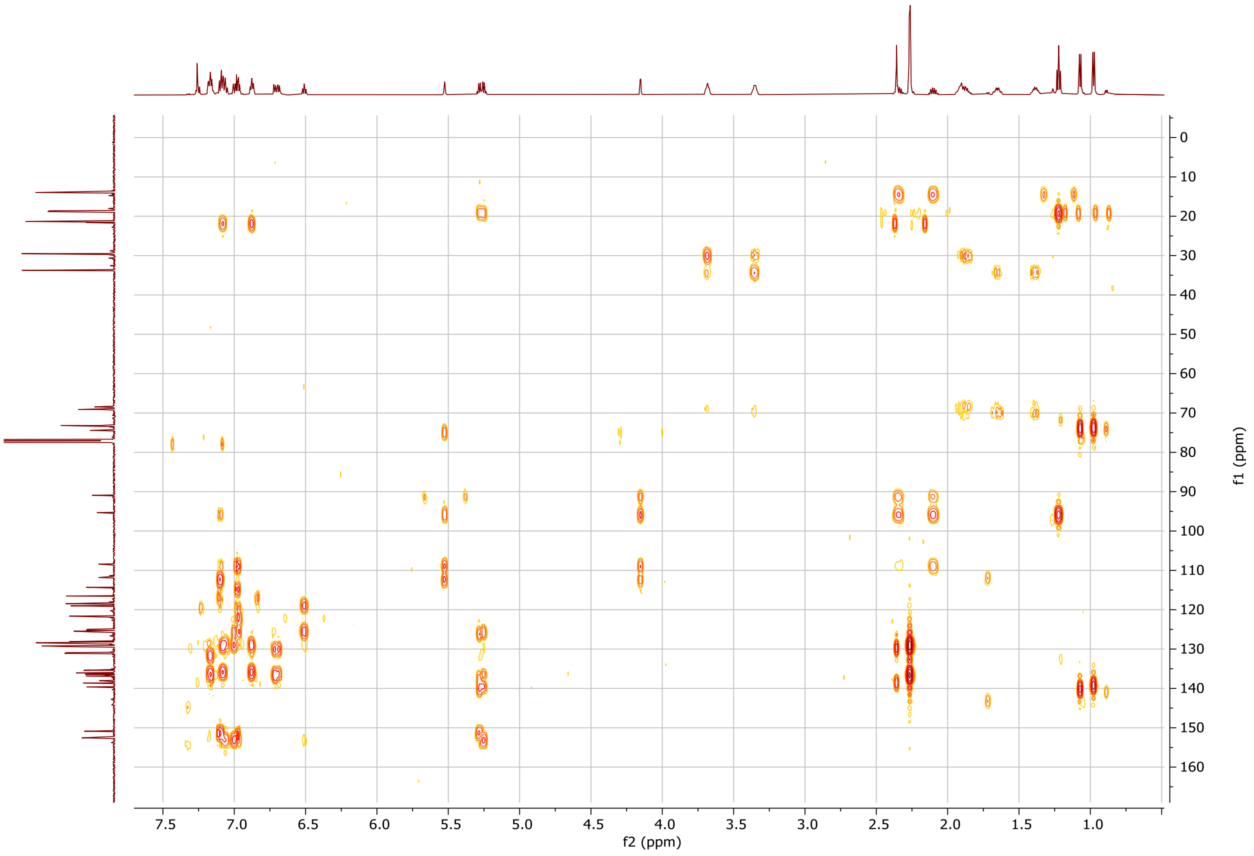

Figure S80: ^{1}H-^{13}C HMBC of (-)-*(M,R,R,R$_p$)*-**22b** (600 MHz/151 MHz, 298 K, CDCl₃).

Compound (-)-*(M,R,R,R_p)*-**22c**

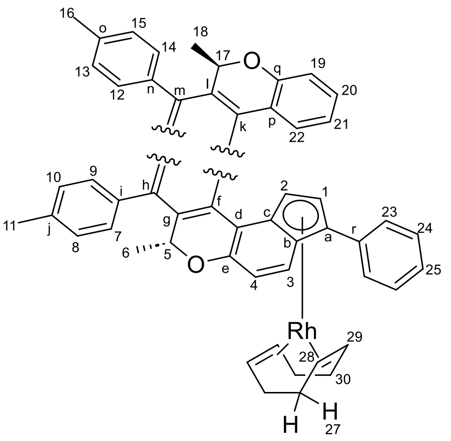

Prepared according to **GP2** from substituted oxa[6]helicene (-)-(*M,R,R*)-**14c** (50 mg, 0.08 mmol, 1.0 equiv.), $[Rh(cod)Cl]_2$ (24.3 mg, 0.05 mmol, 0.55 equiv.), and potassium *tert*-butoxide (12 mg, 0.1 mmol, 1.3 equiv.) providing (-)-*(M,R,R,R_p)*-**22c** (38.7 mg, 58%) as a yellow amorphous solid.

R_f = 0.68 (pentane:toluene 1:1).

$[\alpha]^{20}_D$ -1475.0 (*c* 0.109, THF).

^{1}H NMR (600 MHz, 298 K, CD_2Cl_2): δ = 7.52 (d, *J* = 8.7 Hz, 1H, CH^{3}), 7.48 (m, 2H, CH^{23}), 7.38 (t, *J* = 7.8 Hz, 2H, CH^{24}), 7.24 (m, 1H, CH^{25}), 7.20 (dd, *J* = 7.7, 1.9 Hz, 1H, CH^{7}), 7.18 (dd, *J* = 7.7, 1.9 Hz, 1H, CH^{12}), 7.16 – 7.11 (m, 2H, CH^{8} and CH^{13} overlapping), 7.09 (td, *J* = 7.6, 1.6 Hz, 1H, CH^{22}), 7.03 (d, *J* = 8.6 Hz, 1H, CH^{4}), 6.97 (m, 2H, CH^{19} and CH^{20} overlapping), 6.94 (m, 2H, CH^{15} and CH^{10} overlapping), 6.80 (dd, *J* = 7.7, 1.9 Hz, 1H, CH^{14}), 6.77 (dd, *J* = 7.7, 1.9 Hz, 1H, CH^{9}), 6.52 (td, *J* = 7.6, 1.3 Hz, 1H, CH^{21}), 5.95 (dd, *J* = 2.9, 1.7 Hz, 1H, CH^{1}), 5.24 (q, *J* = 6.7 Hz, 1H, CH^{5}), 5.20 (q, *J* = 6.7 Hz, 1H, CH^{17}), 4.31 (d, *J* = 2.9 Hz, 1H, CH^{2}), 3.77 (tt, *J* = 7.9, 3.4 Hz, 2H, CH^{29}), 3.21 (tdd, *J* = 7.9, 4.6, 2.7 Hz, 2H, CH^{28}), 2.29 (s, 3H, CH_3^{11} and CH_3^{16} partially overlapping), 2.28 (s, 3H, CH_3^{11} and CH_3^{16} partially overlapping), 1.94 – 1.88 (m, 4H, CH_2^{30}), 1.70 – 1.62 (m, 2H, CH^{26}), 1.43 – 1.35 (m, 2H, CH^{27}), 1.13 (d, *J* = 6.7 Hz, 3H, CH_3^{6}), 0.97 (d, *J* = 6.7 Hz, 3H, CH_3^{18}).

^{13}C{^{1}H} NMR (151 MHz, 298 K, CD_2Cl_2): δ = 153.2 (C^e), 151.4 (C^q), 140.3 (C^g), 139.3 (C^l), 137.4 (C^h), 137.0 (C^m), 136.79, 136.76 ($C^{j,o}$), 136.4 (C^r), 135.94, 135.87 ($C^{n,i}$), 131.7, 131.4 ($CH^{9,14}$), 130.0, 129.8 ($CH^{7,12}$), 129.2 (CH^{24}), 129.1, 129.00, 128.99, 128.98, 128.9 ($CH^{8,10,13,15,22}$), 128.3 (CH^{20}), 128.0 (CH^{23}), 126.8 (CH^{25}), 125.8 (C^f), 125.6 (C^k), 125.4 (C^p), 122.1 (CH^{21}), 120.2 (CH^{3}), 119.1 (CH^{19}), 117.6 (CH^{4}), 115.0 (C^d), 113.2 (d, *J* = 2.2 Hz, C^c), 107.4 (d, *J* = 1.4 Hz, C^b), 94.9 (d, *J* = 3.8 Hz, C^a), 92.3 (d, *J* = 4.9 Hz, CH^1), 76.1 (d, *J* = 4.4 Hz, CH^2), 73.9 (CH^5), 73.5 (CH^{17}), 71.5 (d, *J* = 14.0 Hz, 2C, CH^{28}), 70.3 (d, *J* = 13.3 Hz, 2C, CH^{29}), 34.0 (2C, CH_2^{30}), 29.9 (2C, $CH_2^{26/27}$), 21.47, 21.45 ($CH_3^{11,16}$), 19.2 (CH_3^{6}), 18.9 (CH_3^{18}).

HRMS (ESI) *m/z*: ($[M+H]^+$) calcd for $C_{53}H_{48}O_2{}^{103}Rh$ 819.2704, found 819.2692 (Δ = -1.48 ppm).

IR (ATR): 3054 (vw), 3046 (w), 3019 (w), 2976 (w), 2923 (m), 2868 (m), 2823 (w-m), 1600 (w), 1586 (w), 1545 (w), 1515 (w), 1484 (w), 1446 (m), 1426 (m), 1409 (m), 1364 (m), 1324 (m-w), 1317 (w), 1271 (w), 1220 (m), 1177 (w), 1150 (m), 1099 (m), 1069 (s), 1029 (m), 1007 (w-m), 986 (m), 960 (w), 899 (w), 861 (m), 834 (m), 809 (m), 792 (m), 778 (w), 755 (vs), 742 (s), 728 (s), 651 (w), 639 (w), 518 (m), 484 (m), 458 (m) cm^{-1}.

EA: Calcd for $C_{53}H_{47}O_2Rh$: C, 77.74%; H, 5.79%. Found: C, 77.82%; H, 6.10%.

UV/VIS (THF): λ_{max} (log ε) = 276 (4.57), 367 (4.29) nm.

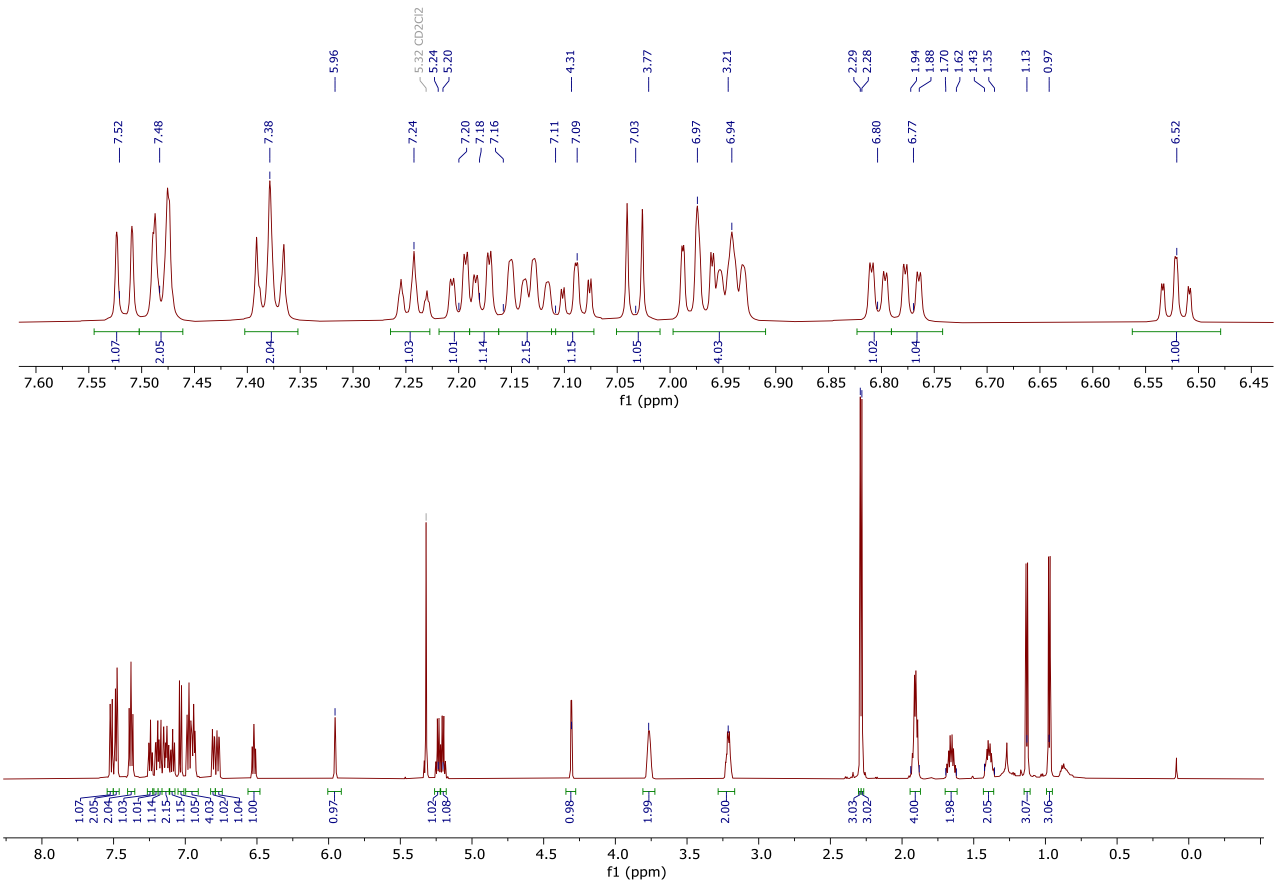

Figure S81: ¹H NMR of (-)-*(M,R,R,R$_p$)*-**22c** (600 MHz, 298 K, CD_2Cl_2).

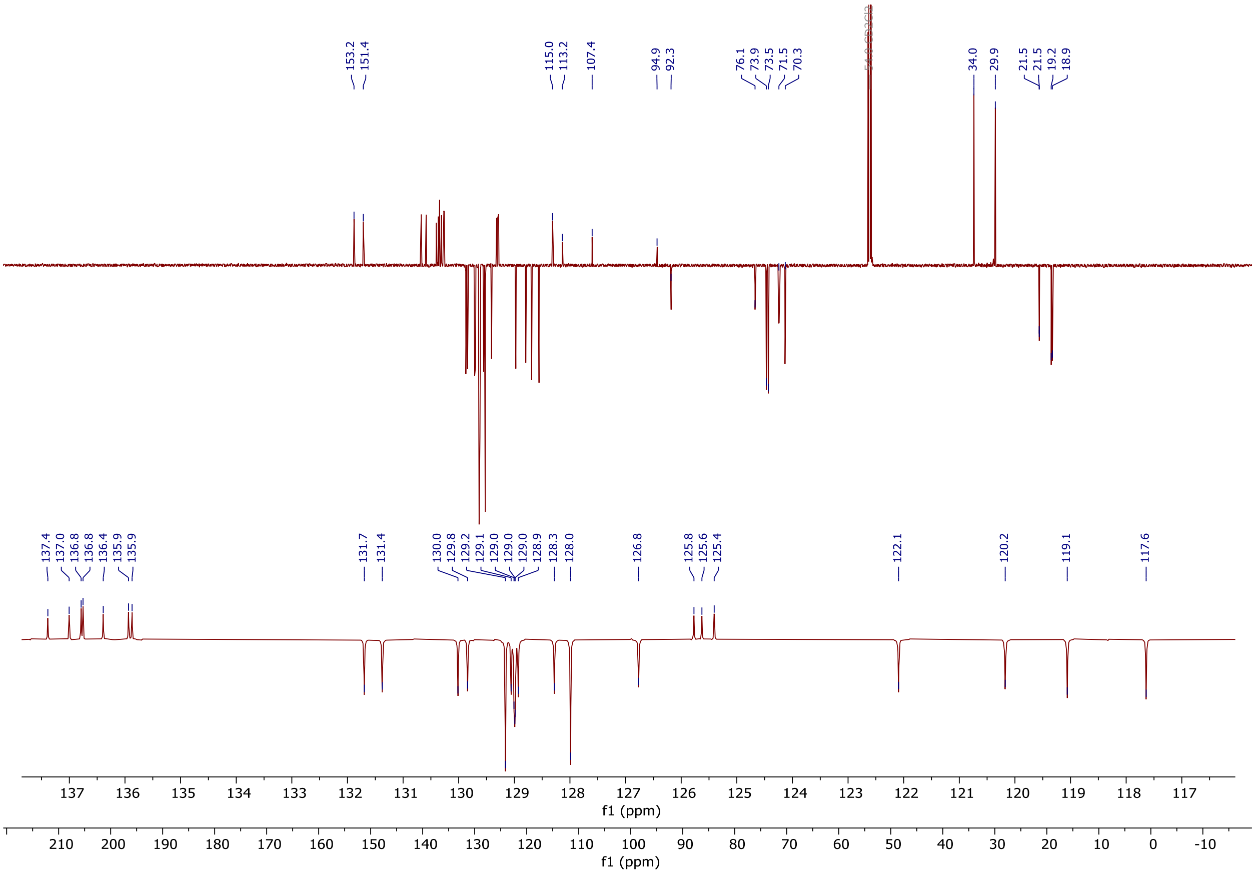

Figure S82: ¹³C APT NMR of (-)-*(M,R,R,R$_p$)*-**22c** (151 MHz, 298 K, CD_2Cl_2).

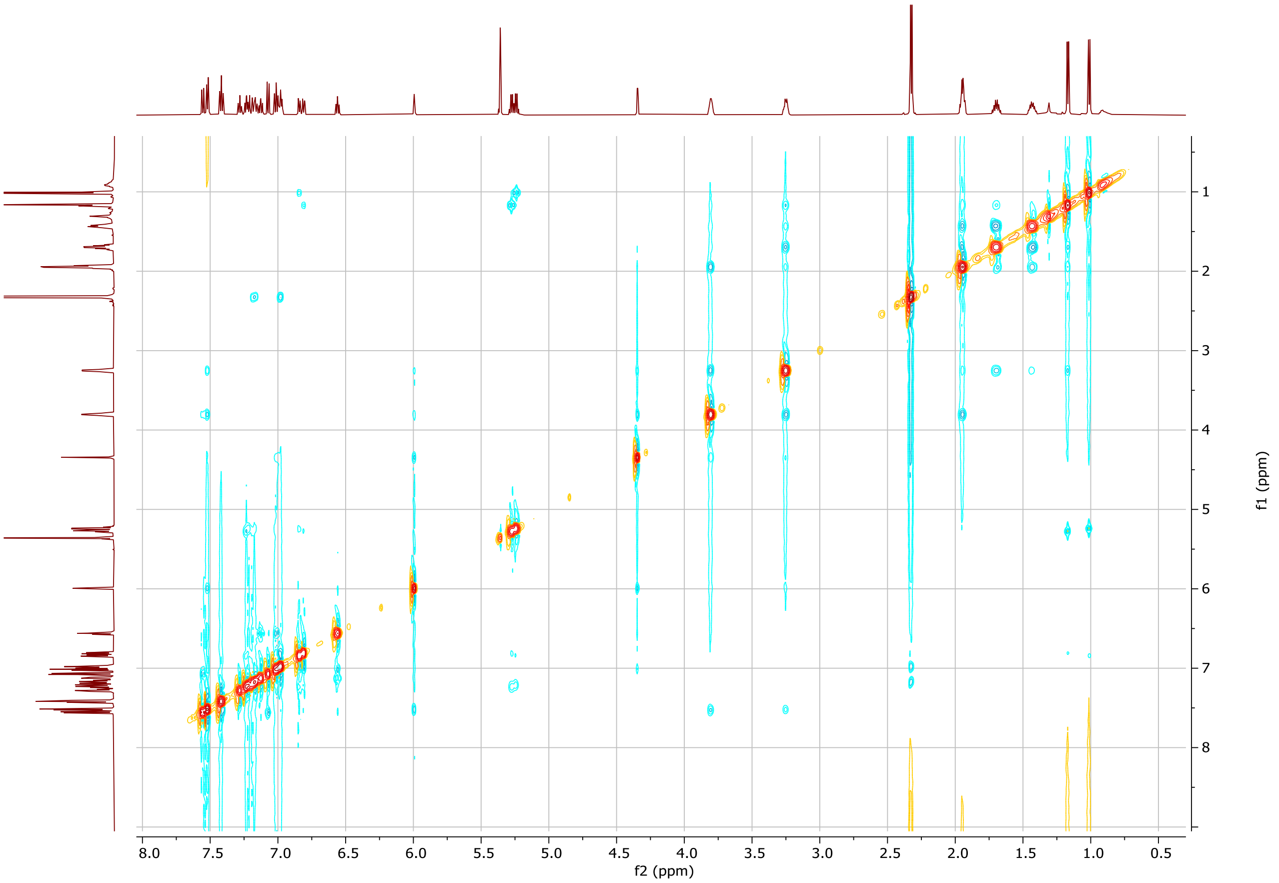

Figure S83: ^{1}H-^{1}H NOESY of (-)-*(M,R,R,R$_p$)*-**22c** (600 MHz/600 MHz, 298 K, CD_2Cl_2).

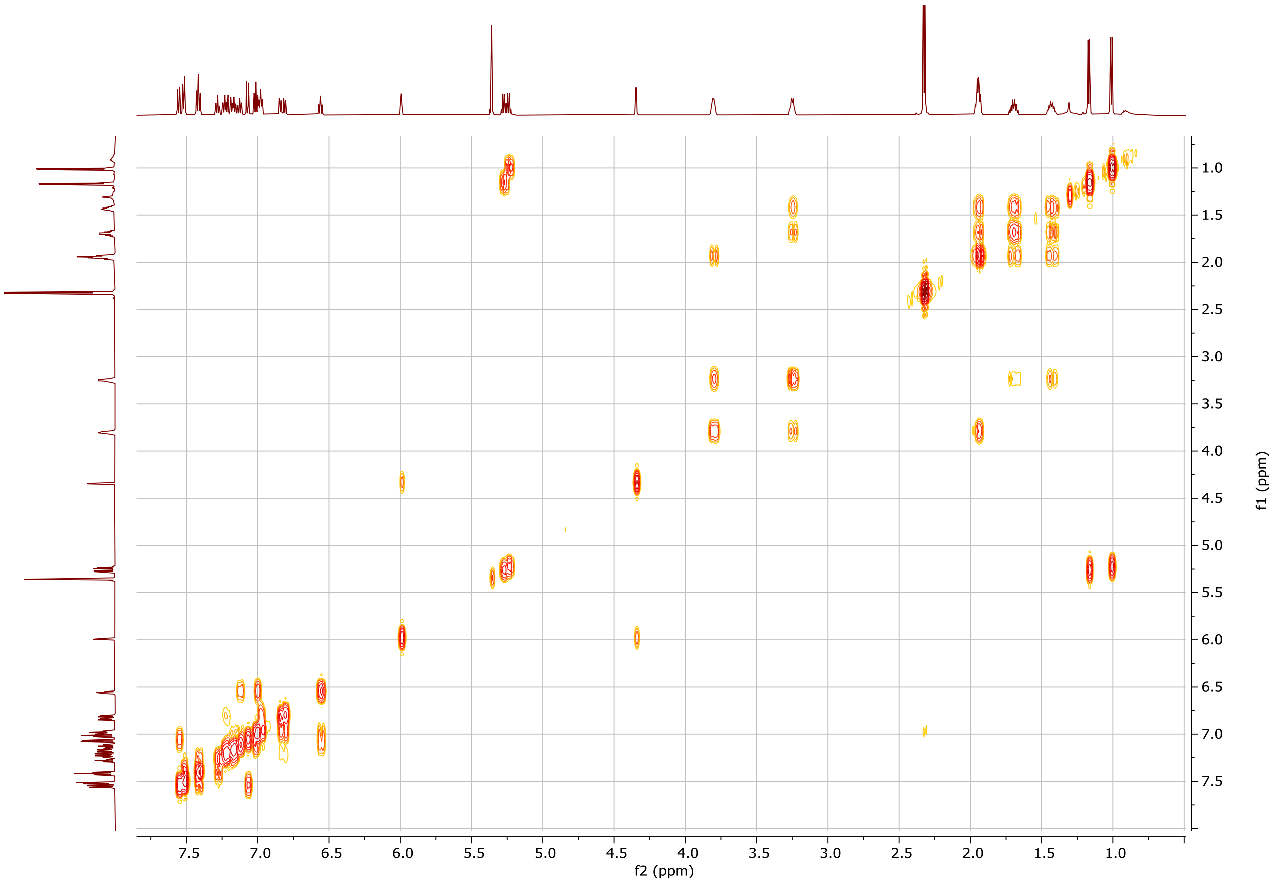

Figure S84: ^{1}H-^{1}H COSY of (-)-*(M,R,R,R$_p$)*-**22c** (600 MHz/600 MHz, 298 K, CD_2Cl_2).

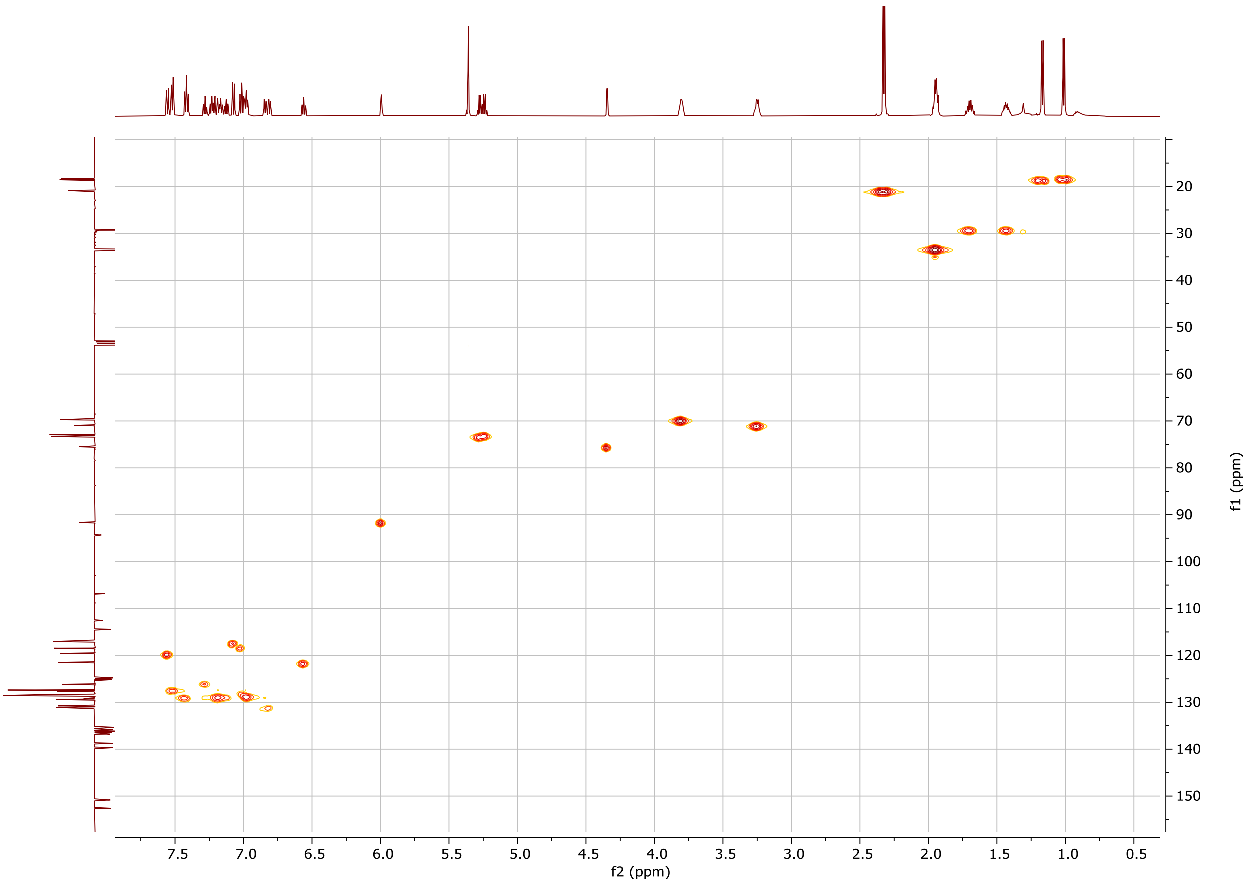

Figure S85: ^{1}H-^{13}C HSQC of (-)-*(M,R,R,R$_p$)*-**22c** (600 MHz/151 MHz, 298 K, CD_2Cl_2).

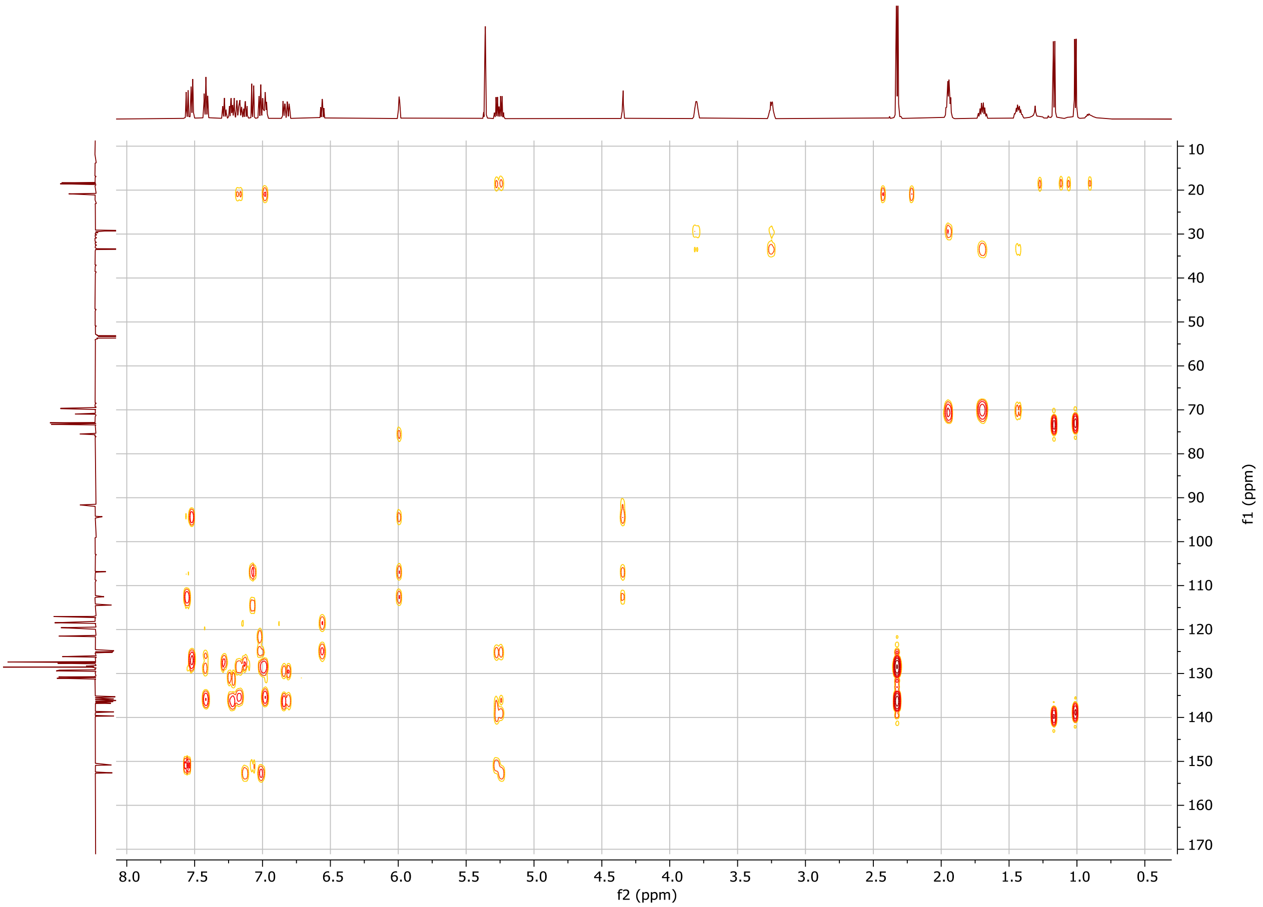

Figure S86: ^{1}H-^{13}C HMBC of (-)-*(M,R,R,R$_p$)*-**22c** (600 MHz/151 MHz, 298 K, CD_2Cl_2).

Compound (+)-(P,S,S,S_p)-**22d**

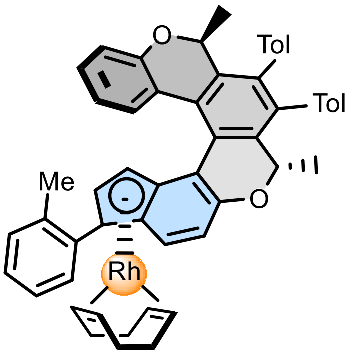

Prepared according to **GP2** from substituted oxa[6]helicene (+)-(*P,S,S*)-**14d** (30 mg, 0.04 mmol, 1.0 equiv.), $[Rh(cod)Cl]_2$ (11.9 mg, 0.02 mmol, 0.55 equiv.), and potassium *tert*-butoxide (7.0 mg, 0.06 mmol, 1.4 equiv.) providing (+)-(P,S,S,S_p)-**22d** (31 mg, 78%) as an orange amorphous solid.

$\mathbf{R_f}$ = 0.71 (pentane:toluene 1:1).

$[\alpha]^{20}_D$ +1480 (*c* 0.178, THF).

^{1}H NMR (400 MHz, 298 K, $CDCl_3$): δ = 7.32 – 7.27 (m, 1H, CH^{Ar}), 7.21 – 7.16 (m, 4H, 2 *o*-protons of *p*-tolyl overlapping with 2 CH^{Ar}), 7.12 – 7.05 (m, 4H, 2 *m*-protons of *p*-tolyl overlapping with 2 CH^{Ar}), 7.03 – 6.94 (m, 4H, 4 CH^{Ar}), 6.93 – 6.87 (m, 2H, *m*-protons of *p*-tolyl), 6.76 – 6.70 (m, 2H, *o*-protons of *p*-tolyl), 6.51 (td, *J* = 7.5, 1.4 Hz, 1H, CH^{Ar}), 5.78 (dd, *J* = 2.9, 1.7 Hz, 1H, Cp*H*), 5.32 (q, *J* = 6.6 Hz, 1H, C*H* chiral center, partially overlapping), 5.28 (q, *J* = 6.6 Hz, 1H, C*H* chiral center, partially overlapping), 4.22 (d, *J* = 2.8 Hz, 1H, Cp*H*), 3.91 – 3.84 (m, 2H, C*H* COD), 3.57 – 3.49 (m, 2H, C*H* COD), 2.32 (s, 3H, CH_3 *o*-tolyl), 2.28 (s, 3H, CH_3 *p*-tolyl), 2.27 (s, 3H, CH_3 *p*-tolyl), 1.96 – 1.88 (m, 4H, CH_2 COD), 1.75 – 1.63 (m, 2H, CH_2 COD), 1.47– 1.35 (m, 2H, CH_2 COD), 1.16 (d, *J* = 6.6 Hz, 3H, CH_3 chiral center), 0.99 (d, *J* = 6.6 Hz, 3H, CH_3 chiral center).

^{13}C{^{1}H} NMR (101 MHz, 298 K, $CDCl_3$): δ = 152.7, 150.9, 139.5, 138.8, 137.0, 136.9, 136.5, 136.12, 136.10, 135.3, 135.2, 134.7, 131.12, 131.07 (2C), 130.8, 129.9, 129.4, 129.3, 128.7, 128.6, 128.5 (2C), 128.2, 126.6, 125.7, 125.3, 125.1, 124.9, 121.5, 119.8, 118.6, 116.9, 114.0, 111.5 (d, *J* = 2.3 Hz), 108.3 (d, *J* = 1.7 Hz), 96.1 (d, *J* = 3.9 Hz), 94.0 (d, *J* = 4.9 Hz), 74.3 (d, *J* = 4.8 Hz), 73.4, 73.2, 69.6 (d, *J* = 13.4 Hz, 4C), 33.7 (2C), 29.6 (2C), 21.4 (3C), 18.8, 18.7.

HRMS (ESI) *m/z*: ($[M]^+$) calcd for $C_{54}H_{49}O_2{}^{103}Rh$ 832.2782, found 832.2776 (Δ = -0.77 ppm).

IR (ATR): 3017 (vw), 2973 (m), 2919 (m), 2865 (m), 2821 (m), 1602 (w), 1585 (w-m), 1544 (w), 1514 (w-m), 1495 (w), 1456 (m), 1444 (m), 1422 (m), 1409 (m), 1378 (w), 1364 (w-m), 1316 (w), 1296 (w), 1270 (w), 1220 (m-s), 1202 (m), 1172 (w), 1148 (m), 1108 (w), 1099 (m), 1090 (w), 1066 (m-s), 1028 (m), 1007 (w), 985 (m), 961 (w), 900 (m), 859 (m), 833 (m), 806 (m-s) 797 (m-s), 787 (w), 778 (w), 753 (vs), 740 (s), 725 (s), 682 (m), 649 (w), 595 (w), 516 (m), 482 (m), 456 (m) cm^{-1}.

Compound *(-)*-(M,R,R,R_p)-**22d**

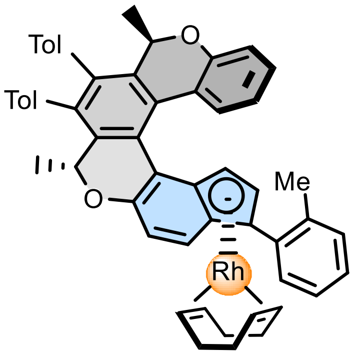

Prepared according to **GP2** from substituted oxa[6]helicene (-)-(*M,R,R*)-**14d** (40 mg, 0.06 mmol, 1.0 equiv.), $[Rh(cod)Cl]_2$ (19 mg, 0.03 mmol, 0.55 equiv.), and potassium *tert*-butoxide (9.4 mg, 0.09 mmol, 1.3 equiv.) providing *(-)*-(M,R,R,R_p)-**22d** (44 mg, 81%) as an orange amorphous solid.

NMR in accordance with (+)-*(P,S,S,S_p)*-**22d**.

$[\alpha]^{20}_D$ -1481.6 (*c* 0.104, THF).

EA: Calcd for $C_{54}H_{51}O_2Rh$: C, 77.68%; H, 6.16%. Found: C, 77.50%; H, 6.44%.

UV/VIS (THF): λ_{max} (log ε) = 272 (4.54), 366 (4.23) nm.

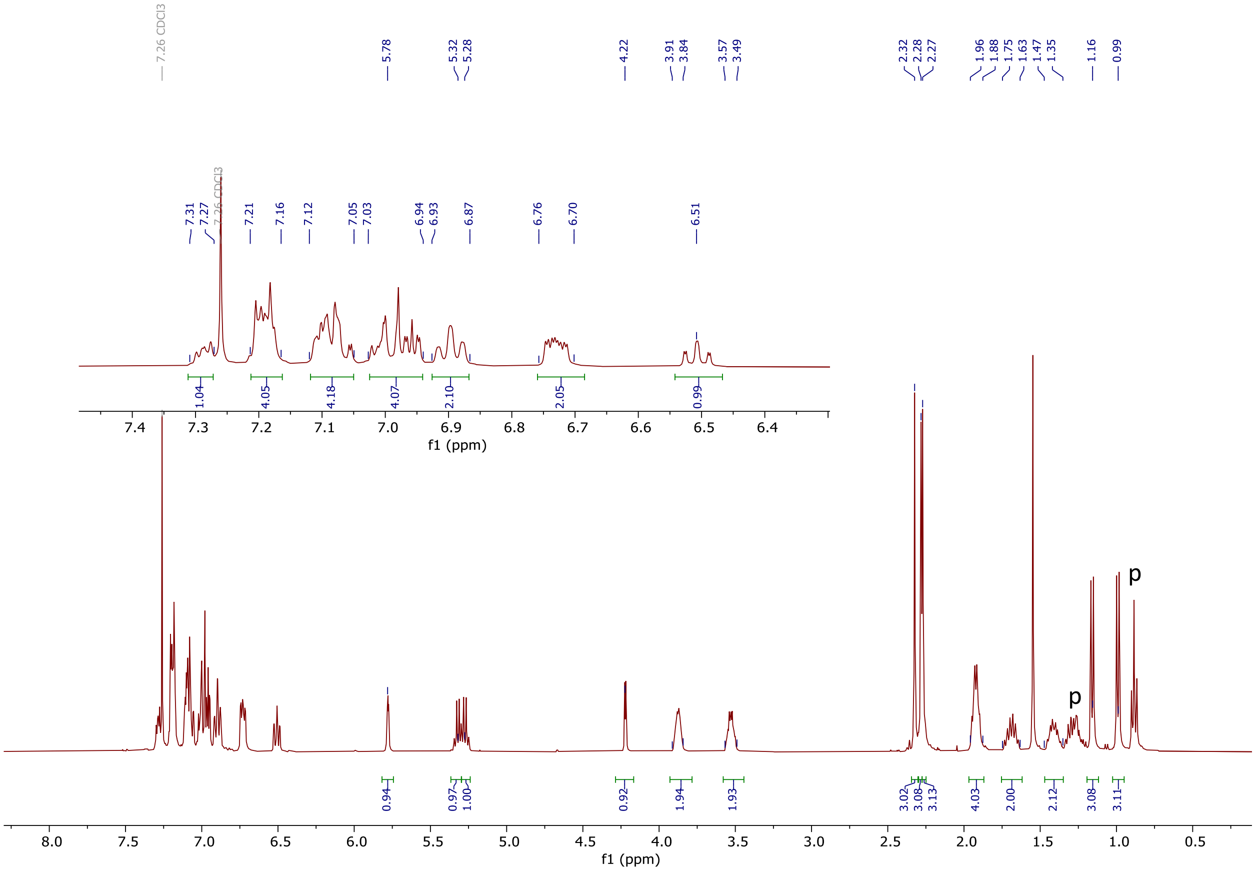

Figure S87: ^{1}H NMR of (+)-(P,S,S,S_p)-**22d** (400 MHz, 298 K, $CDCl_3$) (p = pentane).

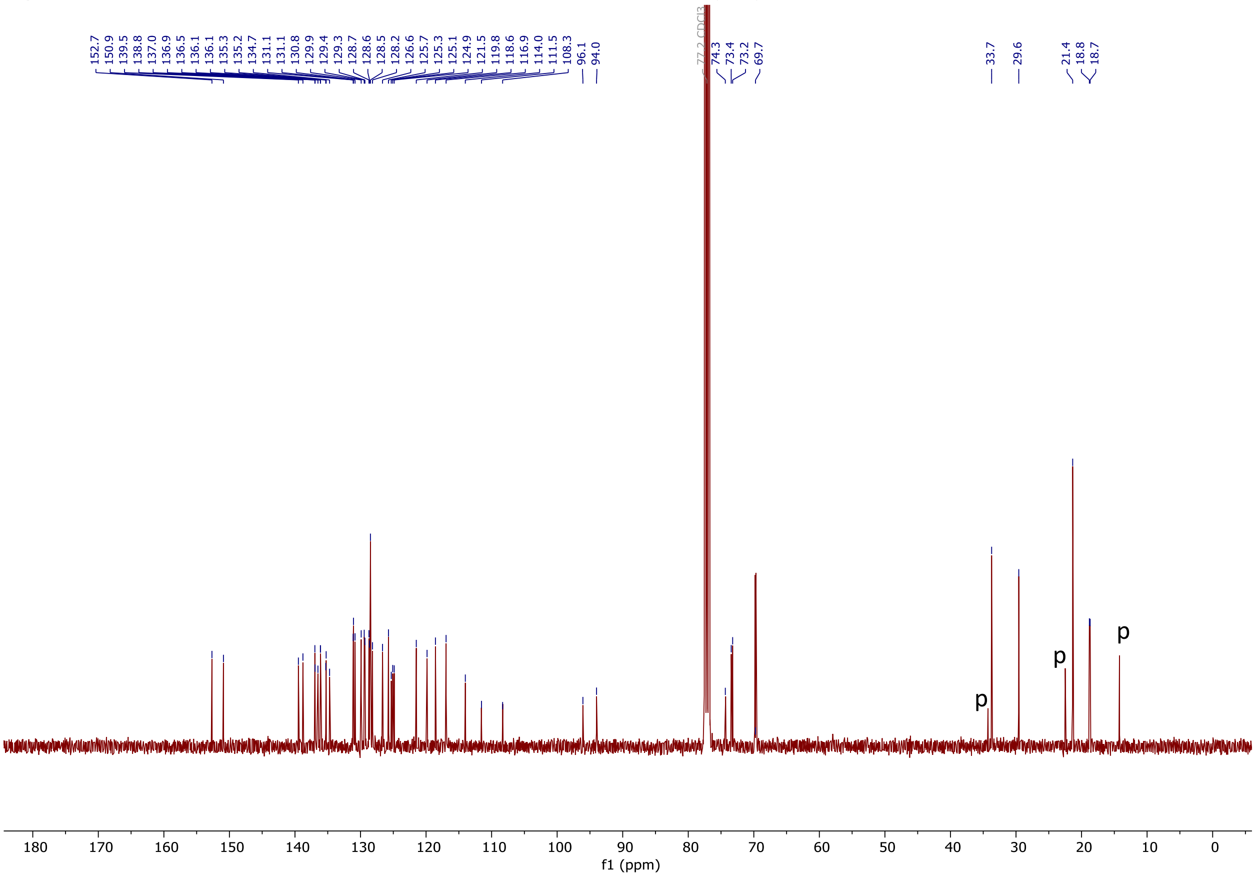

Figure S88: ^{13}C NMR of (+)-(P,S,S,S_p)-**22d** (101 MHz, 298 K, $CDCl_3$) (p = pentane).

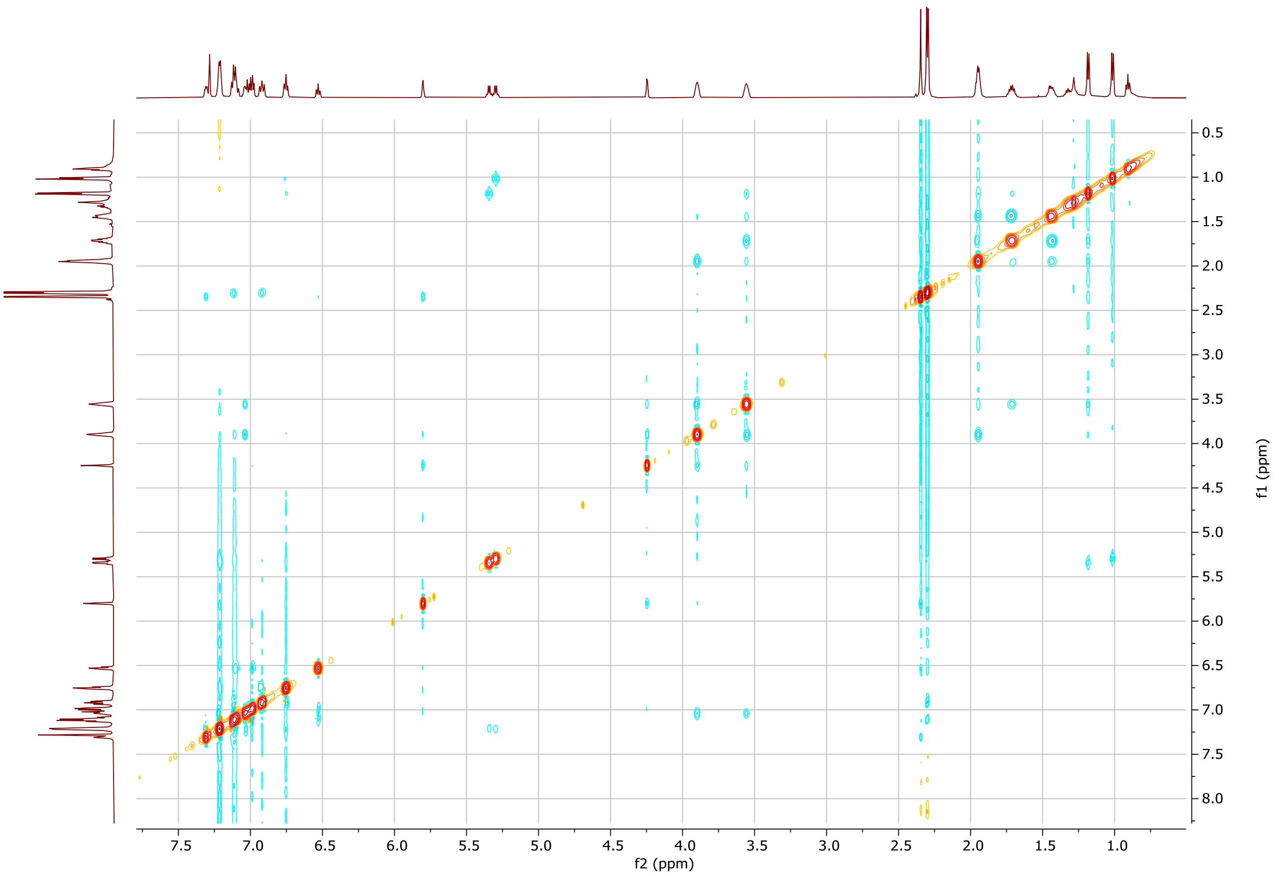

Figure S89: ^{1}H-^{1}H NOESY of (+)-(*P,S,S,S$_p$*)-**22d** (600 MHz/600 MHz, 298 K, $CDCl_3$).

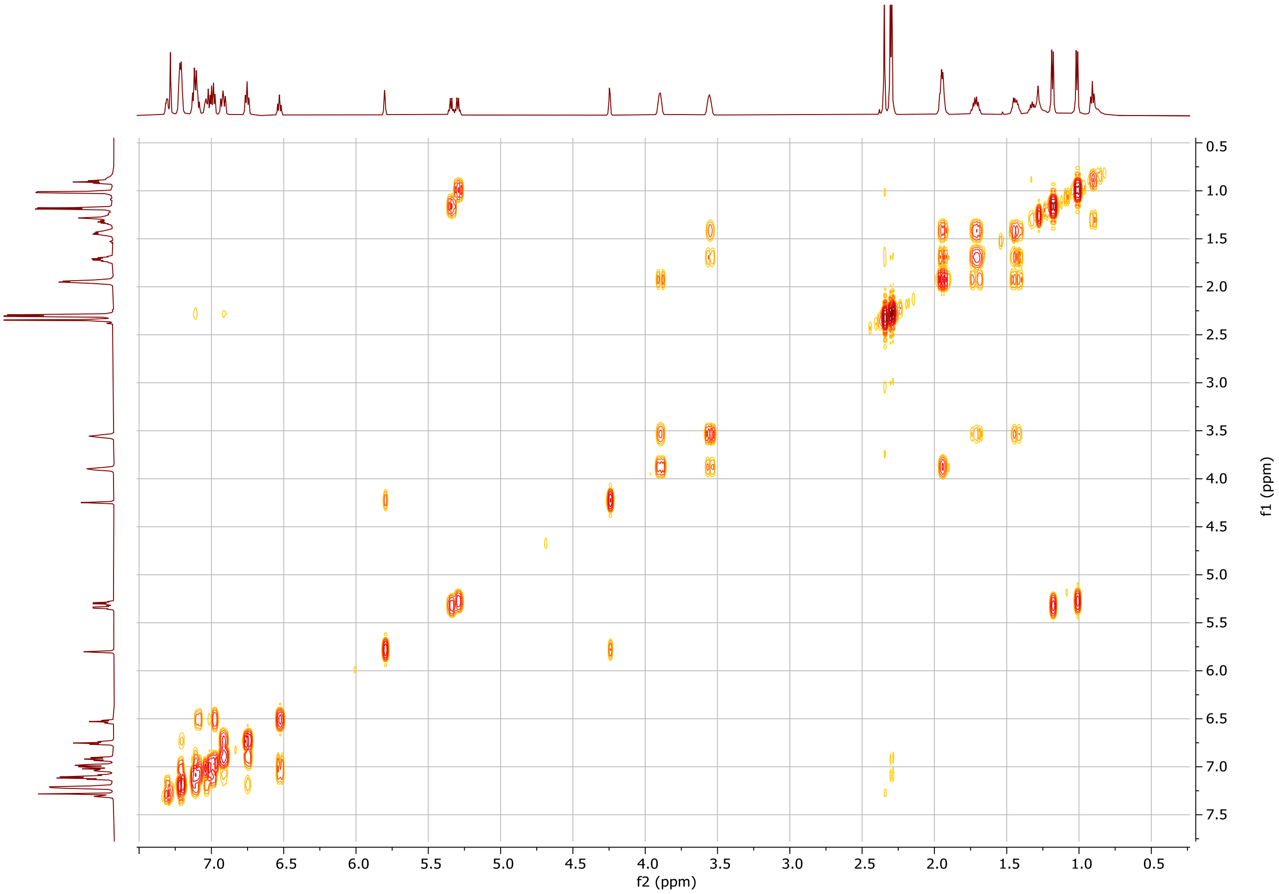

Figure S90: ^{1}H-^{1}H COSY of (+)-(*P,S,S,S$_p$*)-**22d** (600 MHz/600 MHz, 298 K, $CDCl_3$).

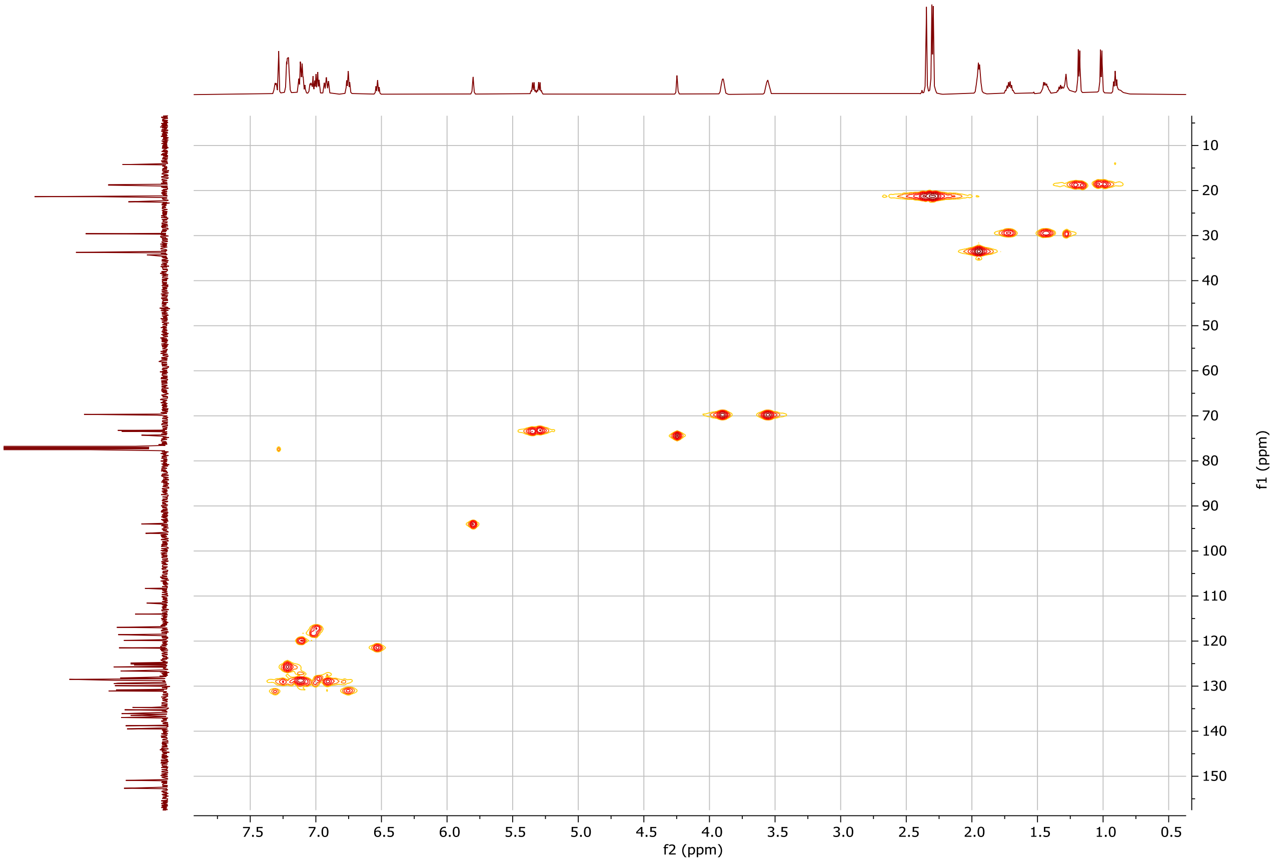

Figure S91: ^{1}H-^{13}C HSQC of (+)-(*P,S,S,S$_p$*)-**22d** (600 MHz/151 MHz, 298 K, $CDCl_3$).

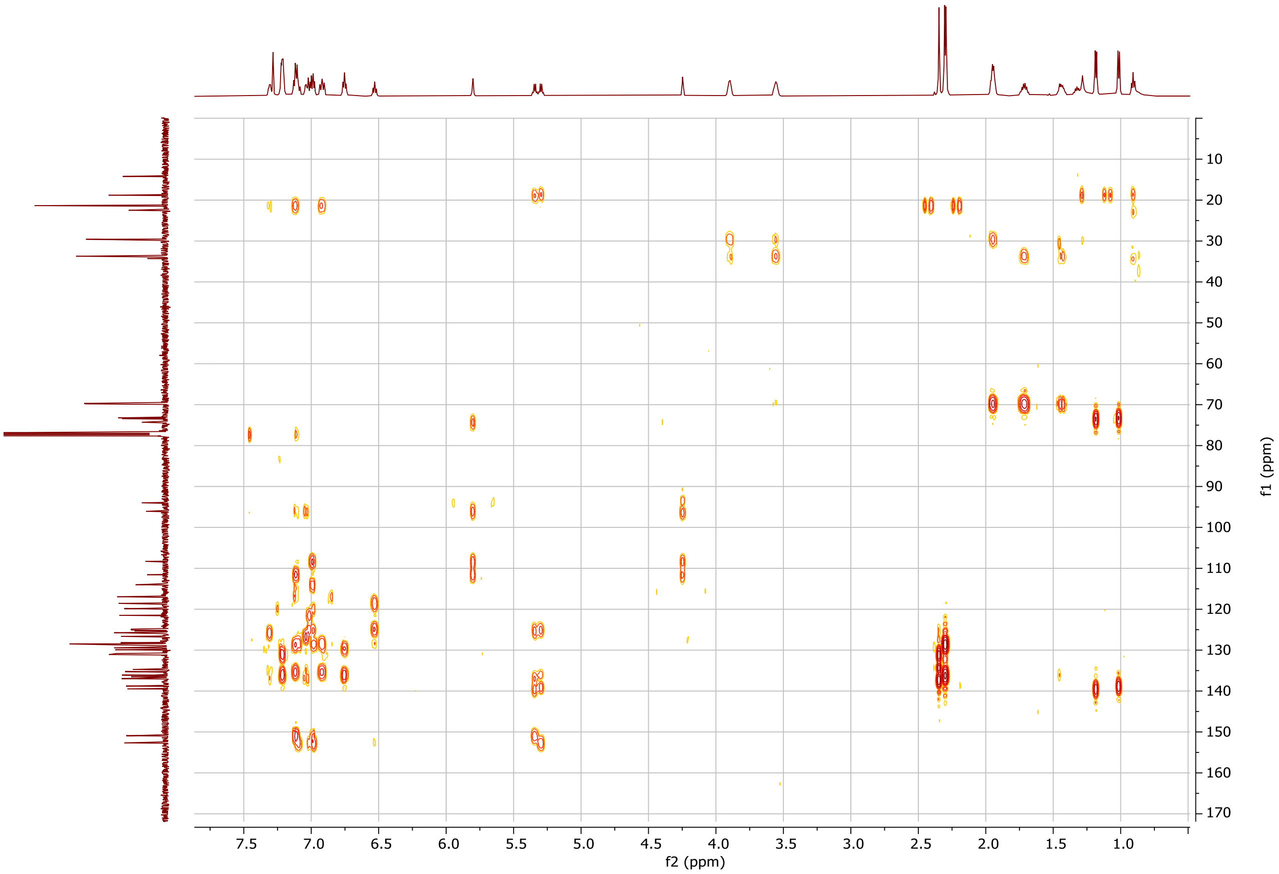

Figure S92: ^{1}H-^{13}C HMBC of (+)-(*P,S,S,S$_p$*)-**22d** (600 MHz/151 MHz, 298 K, $CDCl_3$).

Compound (+)-(*P,S,S,S$_p$*)-**22e**

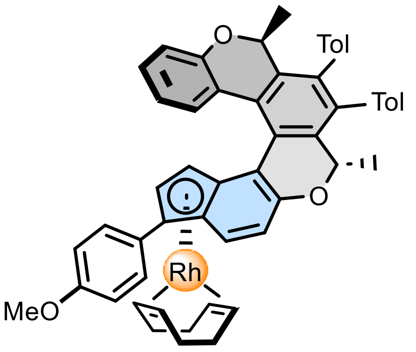

Prepared according to **GP2** from substituted oxa[6]helicene (+)-(*P,S,S*)-**14e** (140 mg, 0.24 mmol, 1.0 equiv.), $[Rh(cod)Cl]_2$ (59.3 mg, 0.12 mmol, 0.55 equiv.), and potassium *tert*-butoxide (34.5 mg, 0.32 mmol, 1.3 equiv.) providing (+)-(*P,S,S,S$_p$*)-**22e** (137 mg, 74%) as an orange amorphous solid.

R$_f$ = 0.47 (pentane:EtOAc 1:1).

$[\alpha]^{20}_D$ +1385.8 (*c* 0.117, THF).

^{1}H NMR (500 MHz, 298 K, CD_2Cl_2): δ = 7.48 (d, *J* = 8.7 Hz, 1H, C*H*^{Ar}), 7.41 (m, 2H, *o*-protons of *p*-anisyl), 7.21 – 7.06 (m, 5H, *m*-protons of *p*-tolyl overlapping with 3 C*H*^{Ar}), 7.02 (d, *J* = 8.7 Hz, 1H, C*H*^{Ar}), 7.00 – 6.90 (m, 6H, *m*-protons of *p*-anisyl overlapping with *m*-protons of *p*-tolyl and 2 C*H*^{Ar}), 6.80 (dd, *J* = 7.8, 1.9 Hz, 1H, *o*-protons of *p*-tolyl), 6.77 (dd, *J* = 7.7, 1.9 Hz, 1H, *o*-protons of *p*-tolyl), 6.52 (td, *J* = 7.5, 1.3 Hz, 1H, C*H*^{Ar}), 5.89 (t, *J* = 2.3 Hz, 1H, Cp*H*), 5.26 – 5.17 (m, 2H, C*H* chiral center), 4.27 (d, *J* = 2.9 Hz, 1H, Cp*H*), 3.82 (s, 3H, OCH_3), 3.75 (m, 2H, CH_2 COD), 3.20 (tdd, *J* = 8.0, 4.6, 2.8 Hz, 2H, C*H* COD), 2.29 (s, 3H, CH_3 tolyl), 2.28 (s, 3H, CH_3 tolyl), 1.94 – 1.87 (m, 4H, CH_2 COD), 1.65 (dq, *J* = 14.4, 7.5 Hz, 2H, CH_2 COD), 1.43 – 1.34 (m, 2H, CH_2 COD), 1.12 (d, *J* = 6.6 Hz, 3H, CH_3 chiral center), 0.97 (d, *J* = 6.7 Hz, 3H, CH_3 chiral center).

^{13}C{^{1}H} NMR (126 MHz, 298 K, CD_2Cl_2): δ = 158.8, 153.2, 151.3, 140.3, 139.3, 137.3, 136.9, 136.8, 136.7, 135.92, 135.86, 131.7, 131.4, 130.0, 129.8, 129.5, 129.03, 128.99, 128.97, 128.96, 128.9, 128.7, 128.4, 128.2, 125.8, 125.6, 125.4, 122.1, 120.2, 119.0, 117.5, 114.9, 114.6 (2C), 112.8 (d, *J* = 2.2 Hz), 107.3 (d, *J* = 2.0 Hz), 95.0 (d, *J* = 3.8 Hz), 91.8 (d, *J* = 5.0 Hz), 75.7 (d, *J* = 4.5 Hz), 73.9, 73.5, 71.3 (d, *J* = 14.0 Hz, 2C), 70.1 (d, *J* = 13.3 Hz, 2C), 55.8, 34.0 (2C), 29.9 (2C), 21.5, 21.4, 19.1, 18.9.

HRMS (ESI) *m/z*: ([M]$^+$) calcd for $C_{54}H_{49}O_3{}^{103}Rh$ 848.2731, found 848.2727 (Δ = -0.54 ppm).

IR (ATR): 2974 (w), 2925 (m), 2866 (m), 2823 (m), 1606 (w), 1583 (w), 1544 (w), 1517 (s), 1483 (w), 1458 (m), 1425 (w), 1408 (m), 1379 (w), 1363 (w), 1315 (w), 1298 (w), 1285 (w), 1270 (w), 1243 (vs), 1219 (m), 1202 (m), 1174 (m), 1149 (m), 1120 (w), 1099 (m), 1092 (w), 1061 (m), 1029 (s), 1007 (w), 986 (m), 961 (w), 899 (m), 860 (m), 830 (s), 796 (s), 787 (m), 778 (w), 763 (m), 753 (s), 740 (s), 725 (m), 681 (m), 636 (w), 610 (w), 601 (w), 593 (w), 515 (m), 486 (m), 456 (m) cm^{-1}.

EA: Calcd for $C_{54}H_{49}O_3Rh$: C, 76.41%; H, 5.82%. Found: C, 76.23%; H, 5.30%.

Compound (-)-(*M,R,R,R$_p$*)-**22e**

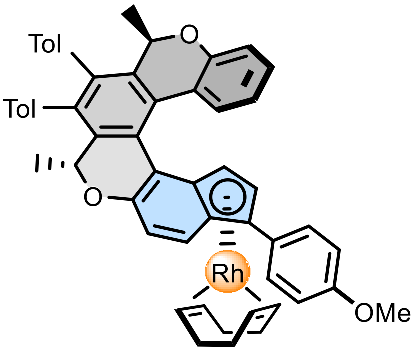

Prepared according to **GP2** from substituted oxa[6]helicene (-)-(*M,R,R*)-**14e** (50 mg, 0.08 mmol, 1.0 equiv.), $[Rh(cod)Cl]_2$ (21.3 mg, 0.04 mmol, 0.55 equiv.), and potassium *tert*-butoxide (11.4 mg, 0.1 mmol, 1.3 equiv.) providing (-)-(*M,R,R,R$_p$*)-**22e** (48 mg, 73%) as an orange amorphous solid.

NMR in accordance with (+)-(*P,S,S,S$_p$*)-**22e**.

$[\alpha]^{20}_D$ -1417.9 (*c* 0.098, THF).

UV/VIS (THF): λ_{max} (log ε) = 276 (4.58), 368 (4.24) nm.

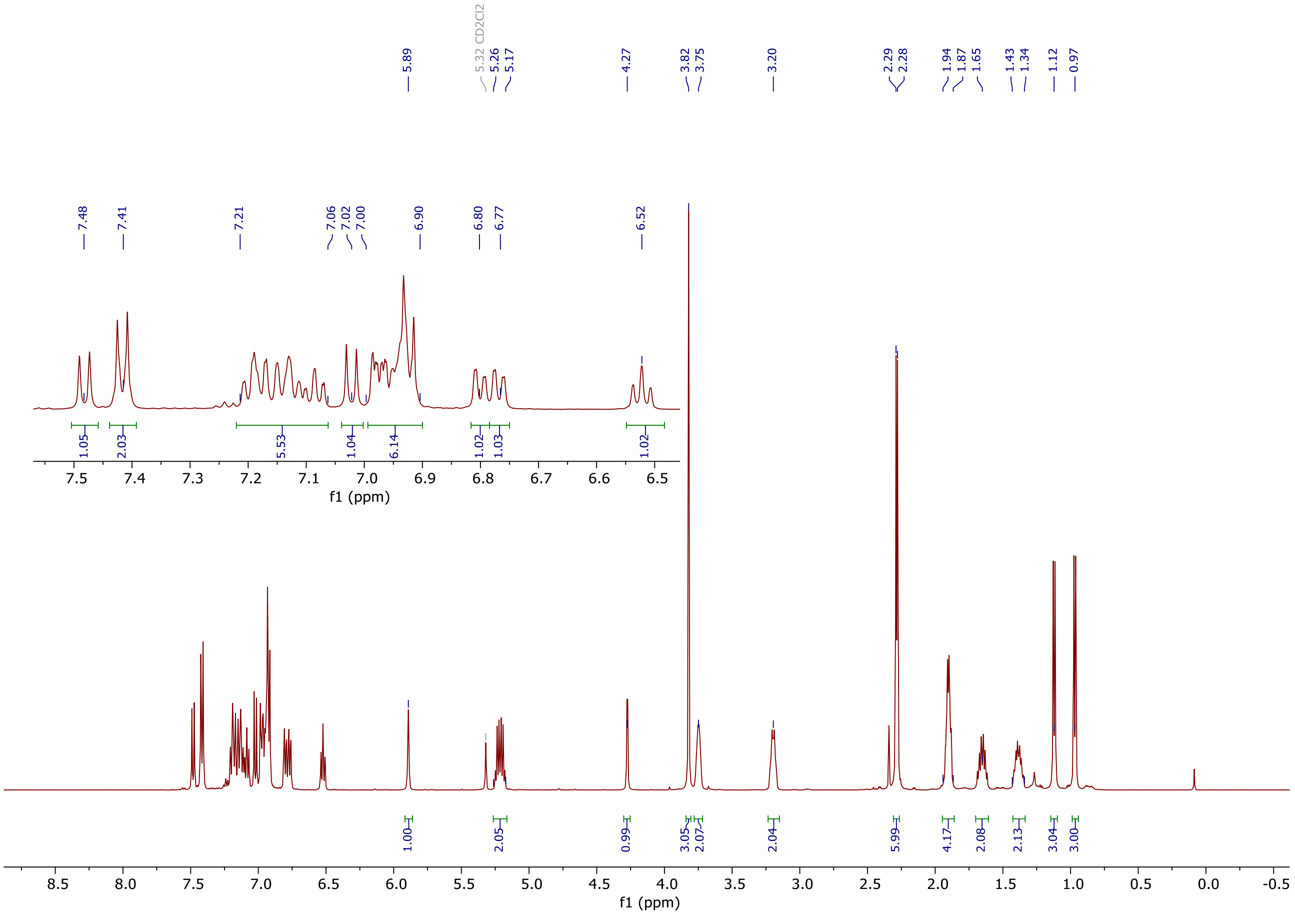

Figure S93: ^{1}H NMR of (+)-(*P,S,S,S$_p$*)-**22e** (500 MHz, 298 K, CD_2Cl_2).

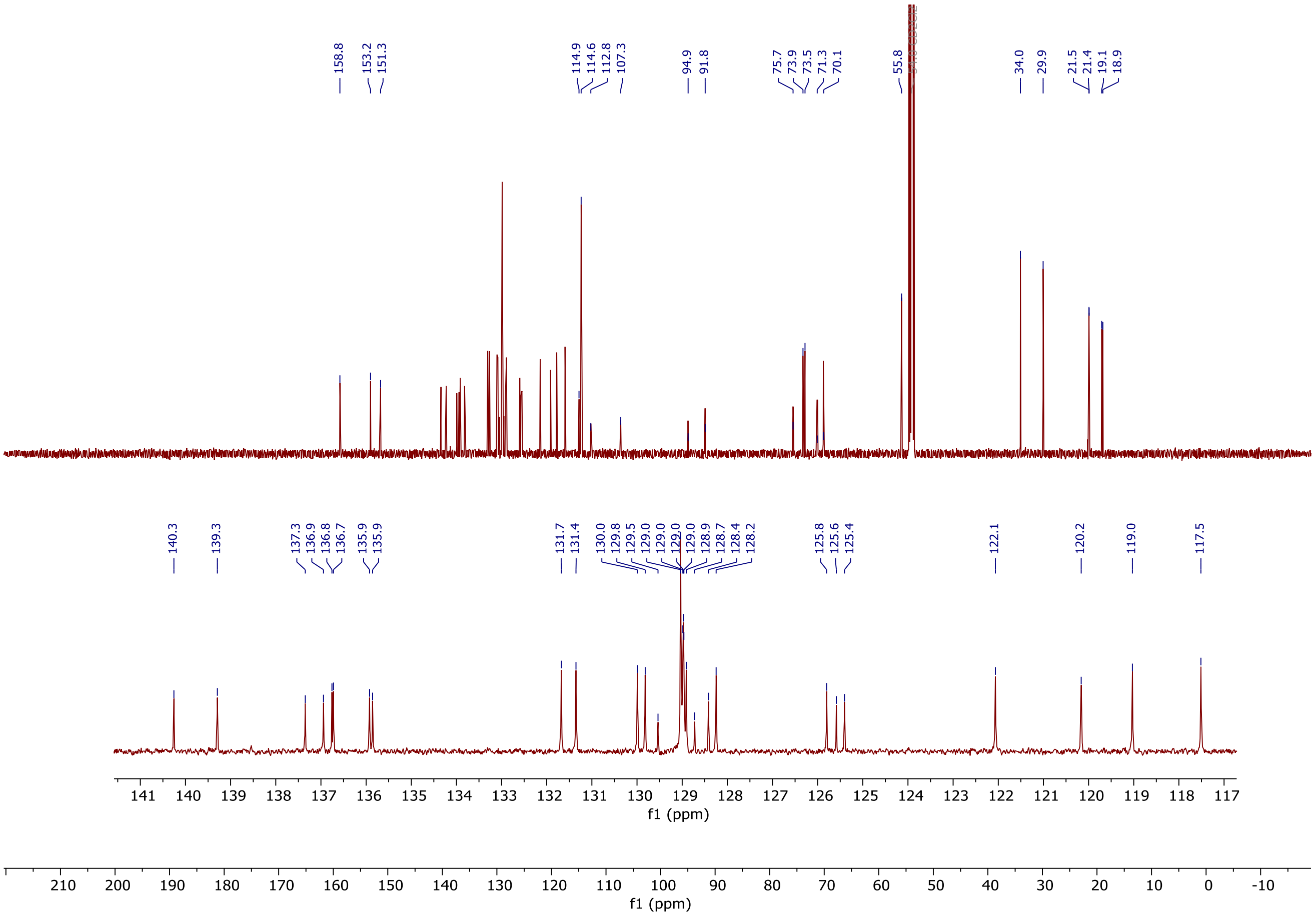

Figure S94: ^{13}C NMR of (+)-(*P,S,S,S$_p$*)-**22e** (126 MHz, 298 K, CD_2Cl_2)

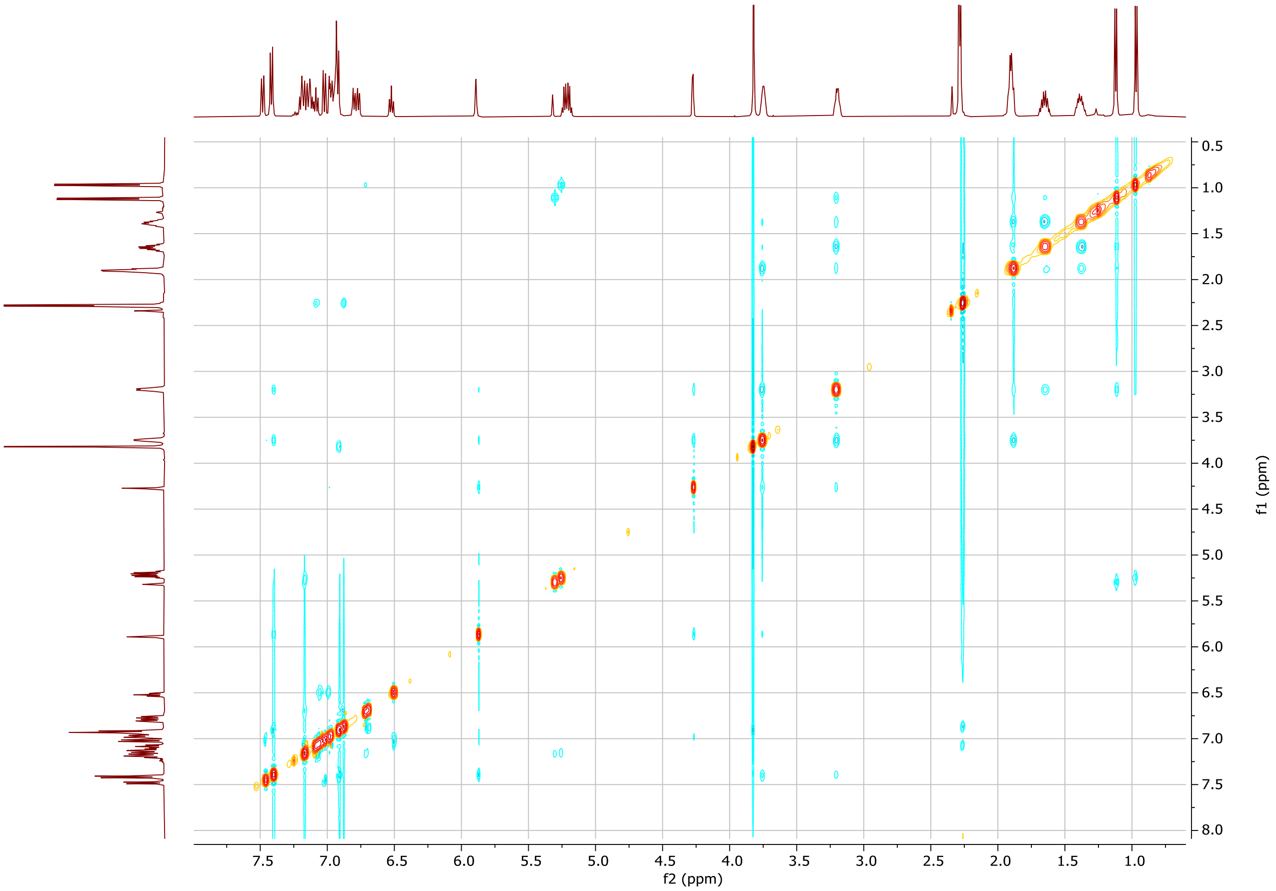

Figure S95: ^{1}H-^{1}H NOESY of (+)-(*P,S,S,S$_p$*)-**22e** (600 MHz/600 MHz, 298 K, $CDCl_3$).

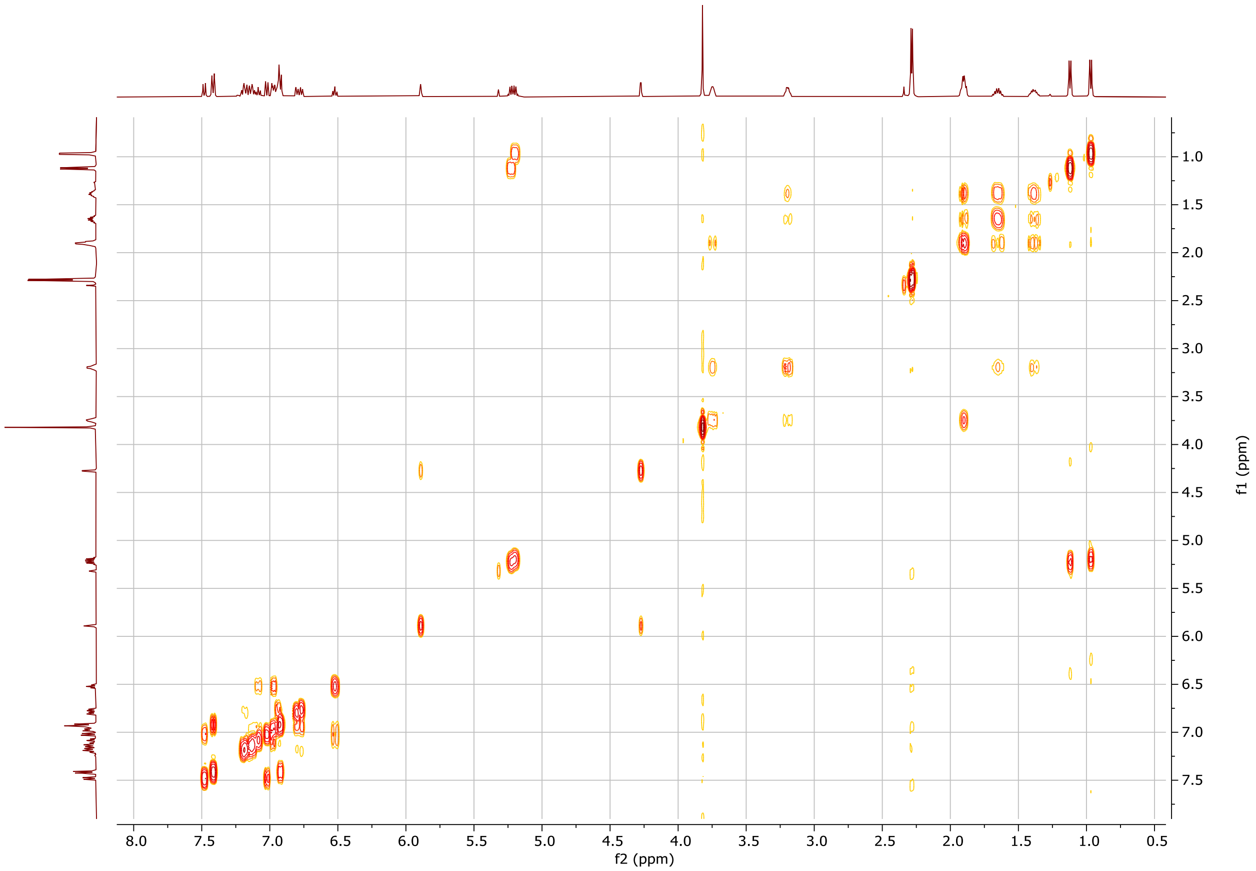

Figure S96: ^{1}H-^{1}H COSY of (+)-(*P,S,S,S$_p$*)-**22e** (500 MHz/500 MHz, 298 K, CD_2Cl_2).

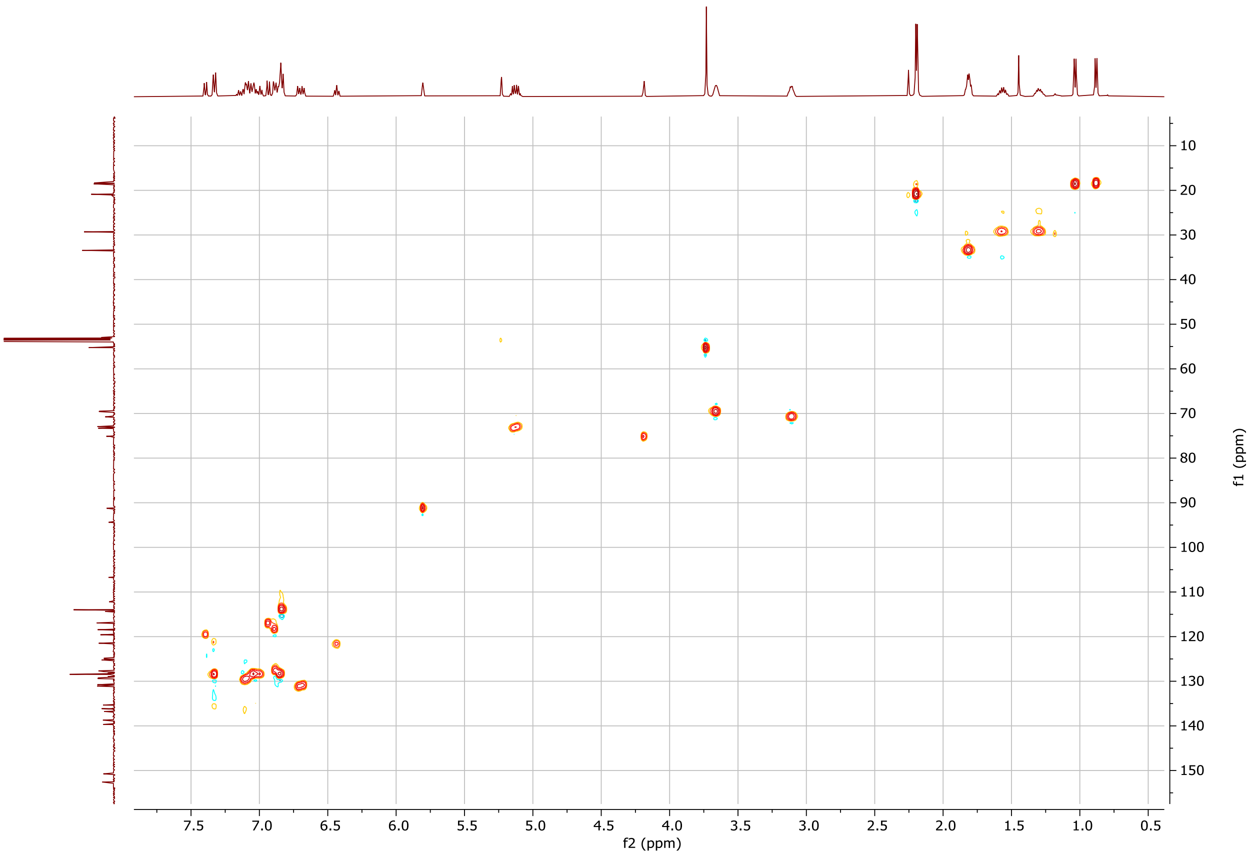

Figure S97: ^{1}H-^{13}C HSQC of (+)-(*P,S,S,S$_p$*)-**22e** (500 MHz/126 MHz, 298 K, CD_2Cl_2).

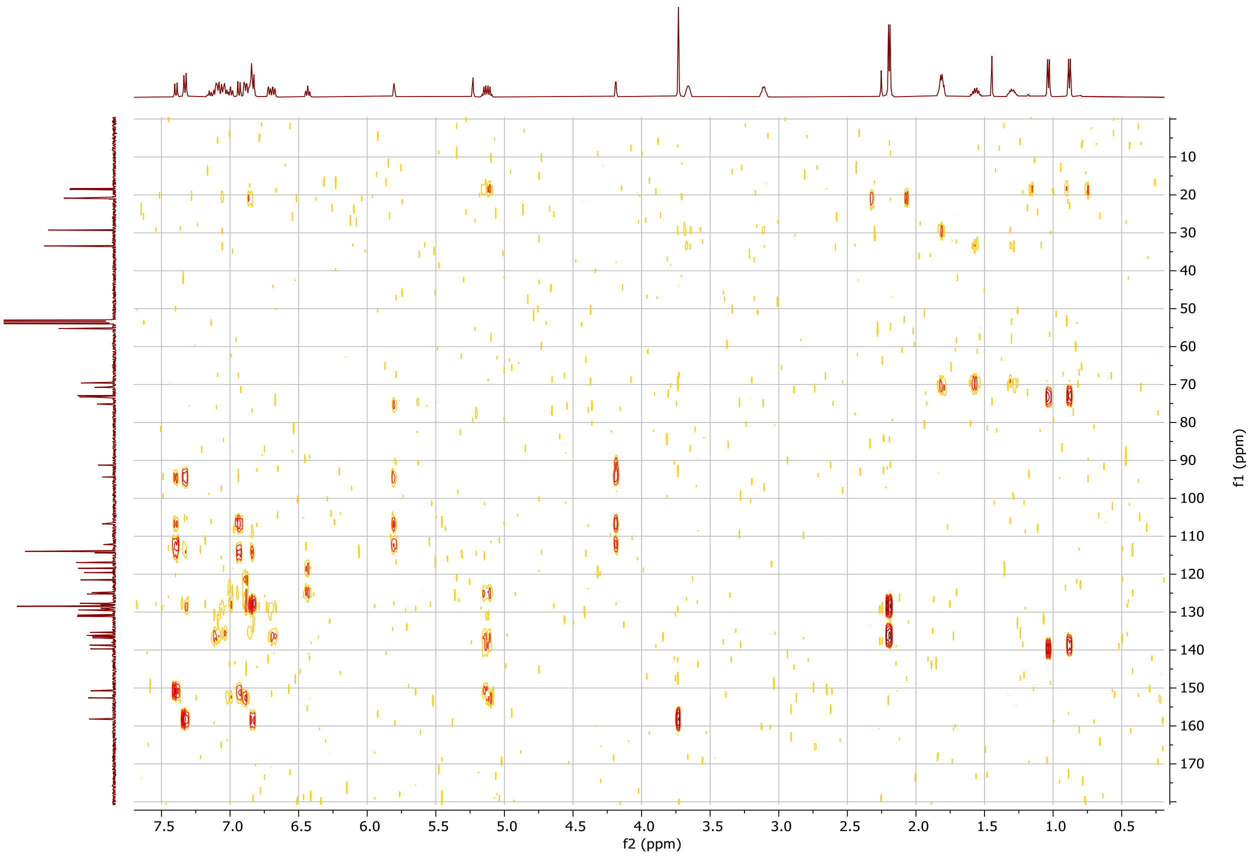

Figure S98: ^{1}H-^{13}C HMBC of (+)-(*P,S,S,S$_p$*)-**22e** (500 MHz/126 MHz, 298 K, CD_2Cl_2).

Compound (-)-(*M,R,R,R$_p$*)-**25a(I)**

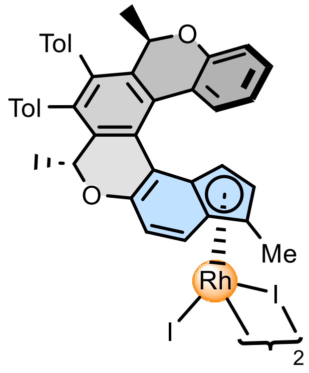

Prepared according to **GP3** starting from (-)-(*M,R,R,R$_p$*)-**22a** (50 mg, 0.07 mmol, 1.0 equiv.) and iodine (41.9 mg, 0.16 mmol, 2.5 equiv.) to give (-)-(*M,R,R,R$_p$*)-**25a(I)** as a dark amorphous solid (58 mg, 97%).

[α]$^{20}_{D}$ -2980 (*c* 0.004, THF).

^{1}H NMR (600 MHz, 298 K, DMSO-*d$_6$*): δ = 7.65 (d, *J* = 8.9 Hz, 1H, C*H*^{Ar}), 7.47 (d, *J* = 8.9 Hz, 1H, C*H*^{Ar}), 7.26 – 7.21 (m, 2H, C*H*^{Ar} overlapping with *o*-proton of *p*-tolyl), 7.20 – 7.16 (m, 1H, *m*-proton of *p*-tolyl), 7.15 – 7.09 (m, 3H, C*H*^{Ar} overlapping with *o*- and *m*-proton of *p*-tolyl), 6.96 (m, 2H, *m*-protons of *p*-tolyl), 6.82 (dd, *J* = 7.8, 1.9 Hz, 1H, *o*-proton of *p*-tolyl), 6.76 (dd, *J* = 7.8, 1.7 Hz, 1H, C*H*^{Ar}), 6.70 (td, *J* = 7.5, 1.2 Hz, 1H, C*H*^{Ar}), 6.66 (dd, *J* = 7.8, 1.9 Hz, 1H, *o*-proton of *p*-tolyl), 5.80 (d, *J* = 2.7 Hz, 1H, Cp*H*), 5.24 (q, *J* = 6.8 Hz, 1H, C*H* chiral center), 5.17 (q, *J* = 6.6 Hz, 1H, C*H* chiral center), 5.03 (d, *J* = 2.7 Hz, 1H, Cp*H*), 2.24 (s, 3H, C*H$_3$* tolyl), 2.22 (s, 3H, C*H$_3$* tolyl), 2.18 (s, 3H, CpC*H$_3$*), 1.15 (d, *J* = 6.8 Hz, 3H, C*H$_3$* chiral center), 0.93 (d, *J* = 6.7 Hz, 3H, C*H$_3$* chiral center).

^{13}C{^{1}H} NMR (151 MHz, 298 K, DMSO-*d$_6$*): δ = 157.4, 152.3, 138.7, 138.3, 138.1, 136.8, 135.9, 135.8, 134.3, 134.1, 130.6, 130.4, 129.8, 129.0, 128.62 (2C), 128.59, 128.5 (3C), 127.5, 126.9, 124.2, 123.6, 123.3, 121.9, 119.2, 118.2, 109.5, 104.9, 92.6 (d, *J* = 6.1 Hz), 92.5 (d, *J* = 6.1 Hz), 74.5 (d, *J* = 6.1 Hz), 74.1, 72.2, 20.7 (2C), 19.6, 18.3, 11.1.

HRMS (ESI) *m/z*: ([M-^{127}I]$^+$) calcd for $C_{80}H_{66}O_4{}^{103}Rh_2{}^{127}I_3$ 1677.0199, found 1677.0193 (Δ = -0.38 ppm).

IR (ATR): 3017 (vw), 2970 (w), 2918 (m), 2861 (w), 1583 (m), 1519 (w-m), 1482 (w), 1460 (w), 1440 (m), 1414 (s), 1362 (m), 1335 (m), 1295 (w), 1270 (w), 1257 (w-m), 1229 (m), 1215 (s), 1181 (w), 1142 (m), 1124 (w), 1102 (m), 1185 (w), 1063 (vs), 1029 (m), 1003 (s), 987 (w), 941 (w), 906 (w), 861 (w), 847 (w), 832 (m), 845 (m), 794 (m), 756 (vs), 741 (vs), 678 (w), 660 (vw), 634 (w), 598 (w), 588 (w), 523 (m), 490 (m), 473 (w), 449 (w), 427 (m) cm^{-1}.

Satisfactory **EA** was not obtained.

UV/VIS (THF): λ_{max} (log ε) = 272 (4.83), 323 (4.68), 528 (4.11) nm.

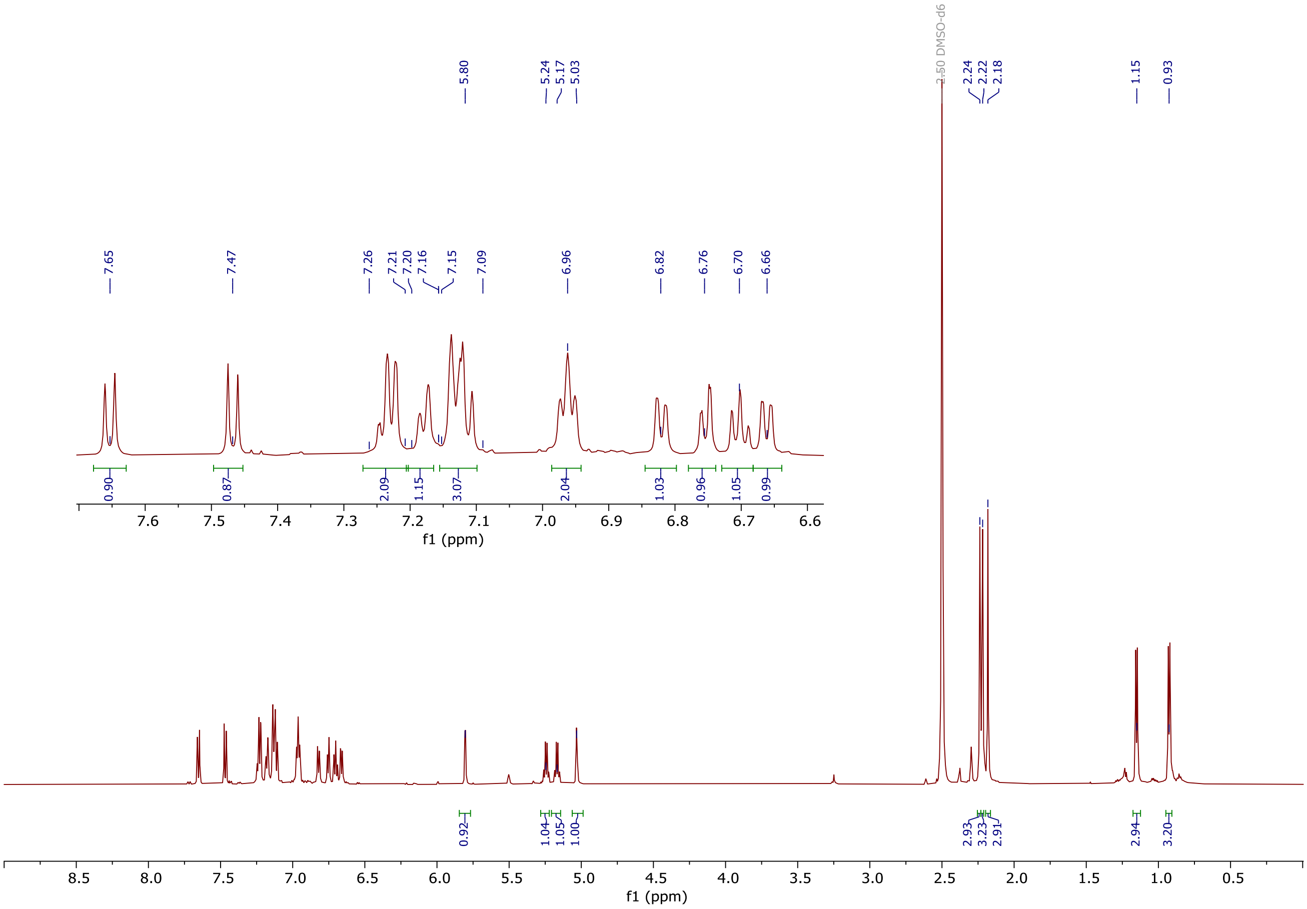

Figure S99: ^{1}H NMR of (-)-(*M,R,R,R$_p$*)-**25a**(I) (600 MHz, 298 K, DMSO-*d$_6$*).

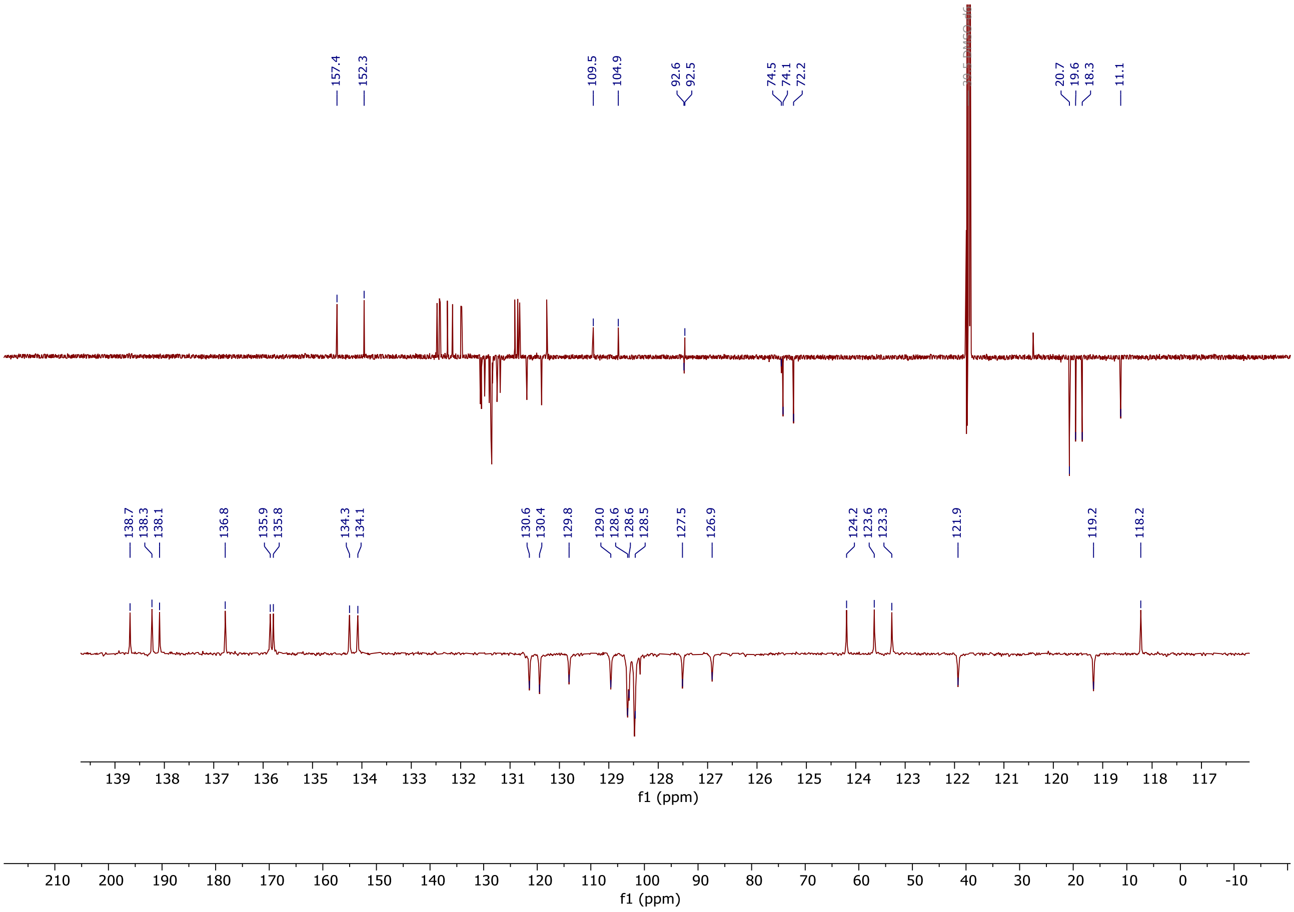

Figure S100: ^{13}C NMR of (-)-(*M,R,R,R$_p$*)-**25a**(I) (151 MHz, 298 K, DMSO-*d$_6$*).

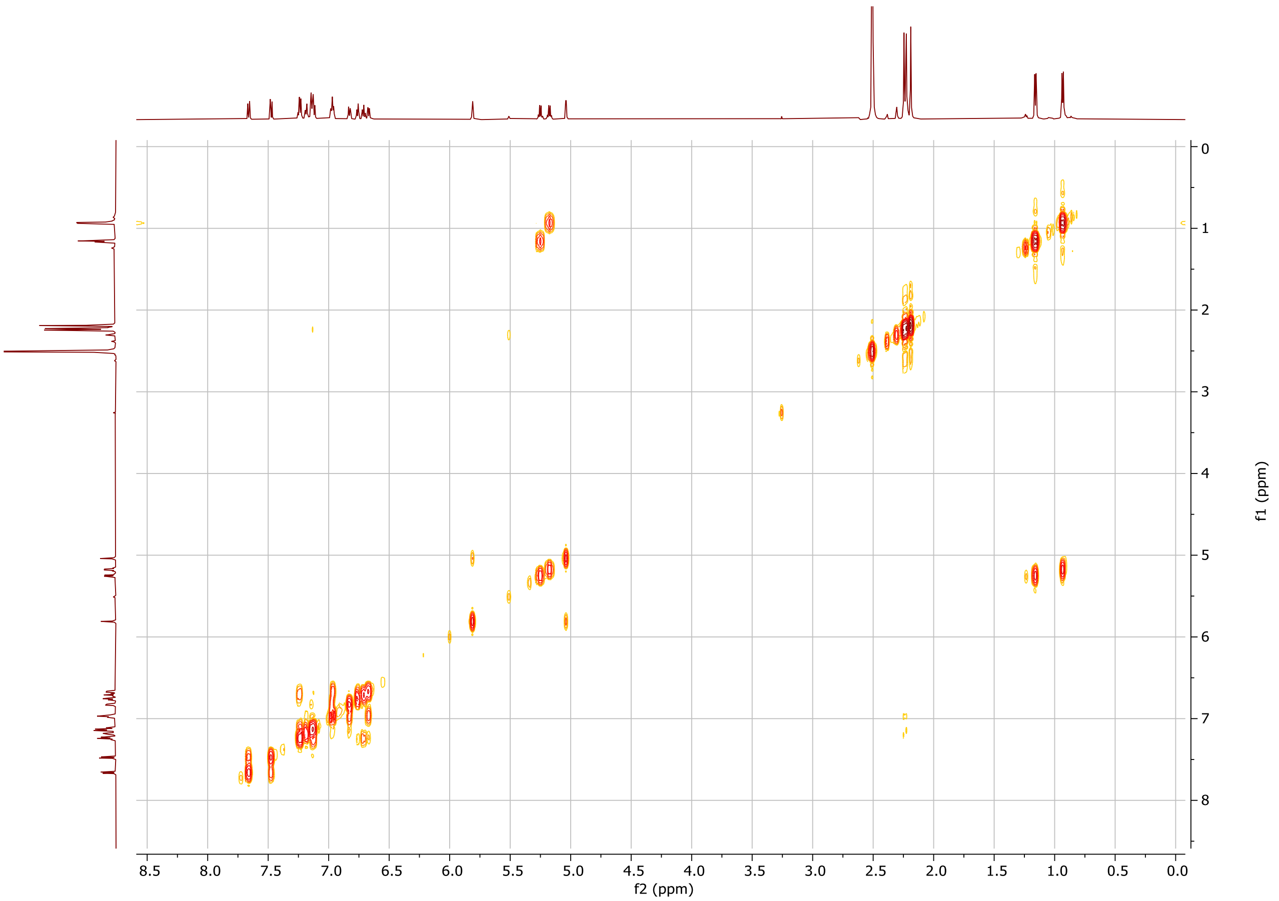

Figure S101: ^{1}H-^{1}H COSY of (-)-(M,R,R,R_p)-**25a**(I) (600 MHz/600 MHz, 298 K, DMSO-d_6).

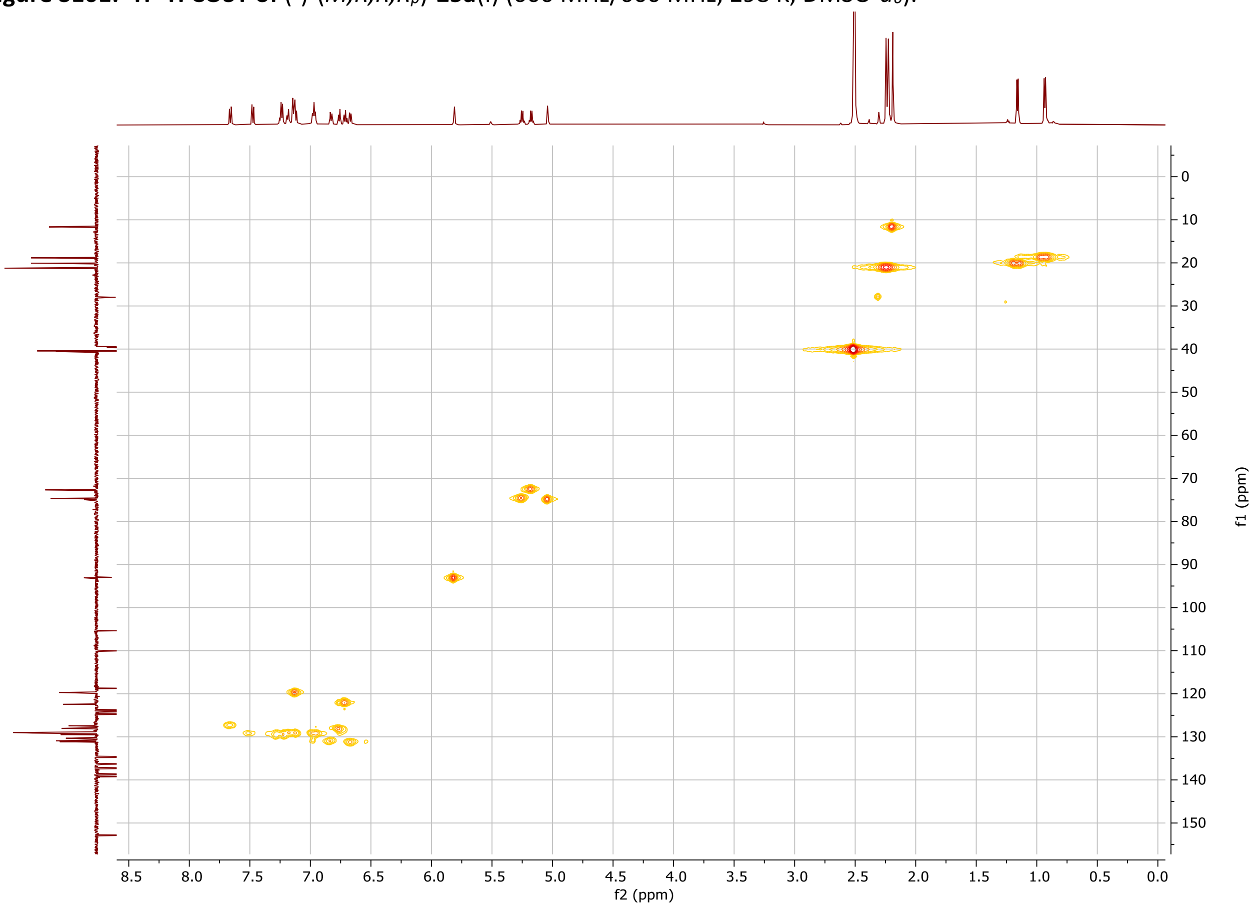

Figure S102: ^{1}H-^{13}C HSQC of (-)-(M,R,R,R_p)-**25a**(I) (600 MHz/151 MHz, 298 K, DMSO-d_6).

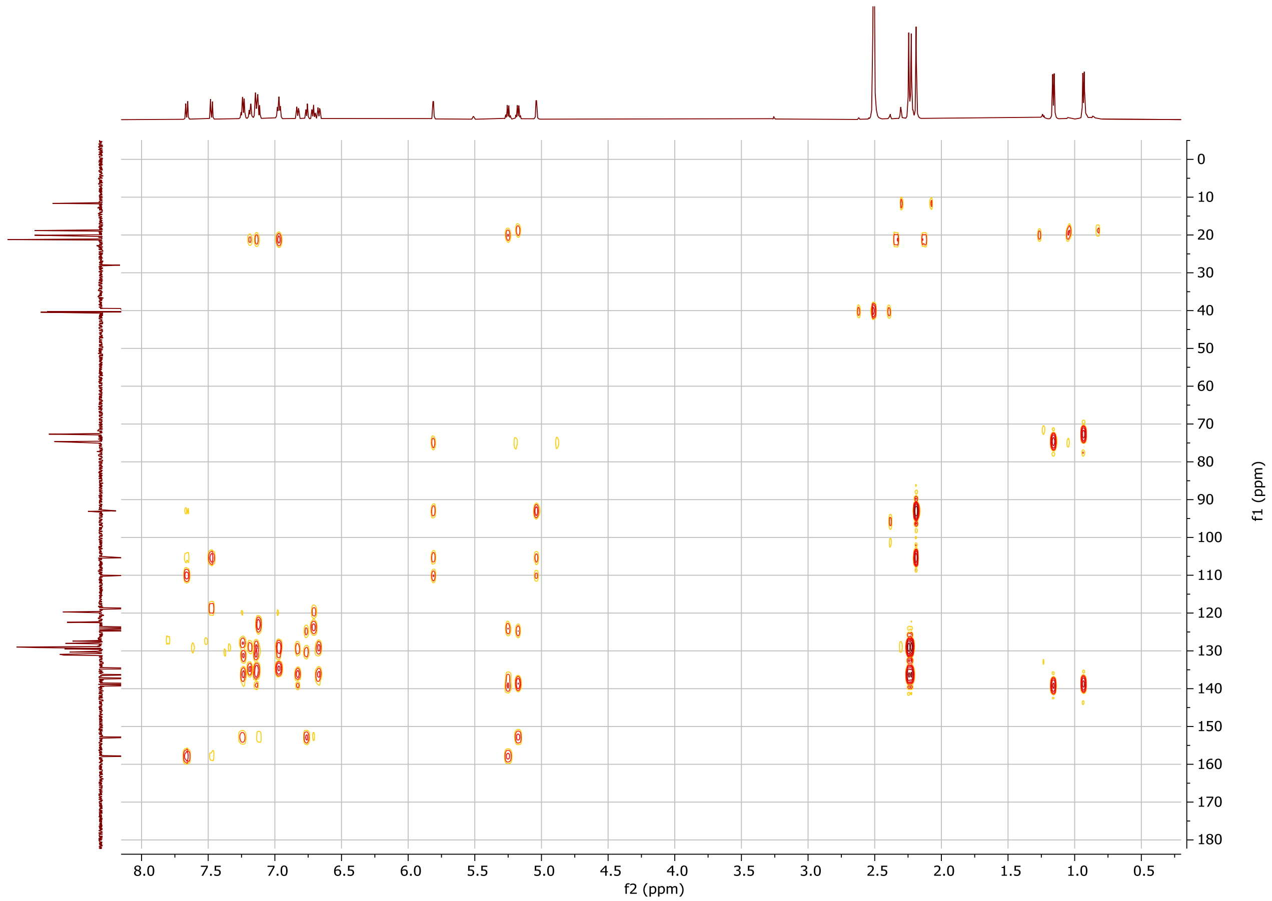

Figure S103: **^{1}H-^{13}C HMBC of** (-)-(*M,R,R,R$_p$*)-**25a**(I) (600 MHz/151 MHz, 298 K, DMSO-*d*$_6$).

Compound (-)-(M,R,R,R_p)-**25e**

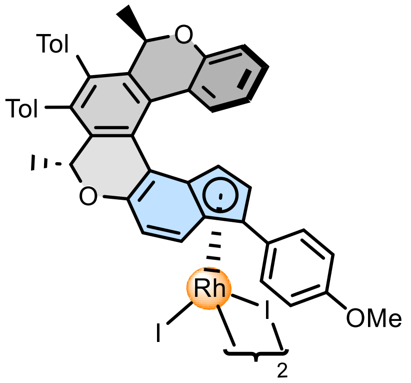

Prepared according to **GP3** starting from (-)-(M,R,R,R_p)-**22e** (50 mg, 0.06 mmol, 1.0 equiv.) and iodine (37.4 mg, 0.15 mmol, 2.5 equiv.). The product was once reprecipitated from toluene/pentane to give (-)-(M,R,R,R_p)-**25e** (46.1 mg, 79%) as a dark shiny solid.

$[\alpha]^{20}_D$ -2700 (*c* 0.004, THF).

^{1}H NMR (600 MHz, 298 K, DMSO-d_6): δ = 8.06 (d, *J* = 8.6 Hz, 2H, *o*-protons of *p*-anisyl), 7.79 (d, *J* = 9.0 Hz, 1H, CH^{Ar}), 7.55 (d, *J* = 9.0 Hz, 1H, CH^{Ar}), 7.33 – 7.22 (m, 2H, CH^{Ar} overlapping with *o*-proton of *p*-tolyl), 7.22 – 7.18 (m, 1H, *m*-proton of *p*-tolyl), 7.16 – 7.12 (m, 3H, *o*- and *m*-proton of *p*-tolyl overlapping with CH^{Ar}), 7.10 (d, *J* = 8.8 Hz, 2H, *m*-protons of *p*-anisyl), 6.97 (m, 2H, *m*-protons of *p*-tolyl), 6.84 (dd, *J* = 7.7, 1.9 Hz, 1H, *o*-proton of *p*-tolyl), 6.81 (dd, *J* = 7.8, 1.6 Hz, 1H, CH^{Ar}), 6.71 (t, *J* = 7.3 Hz, 1H, CH^{Ar}), 6.67 (dd, *J* = 7.7, 1.9 Hz, 1H, *o*-proton of *p*-tolyl), 6.15 (d, *J* = 3.0 Hz, 1H, Cp*H*), 5.31 (d, *J* = 2.9 Hz, 1H, Cp*H*), 5.26 (q, *J* = 6.8 Hz, 1H, C*H* chiral center), 5.18 (q, *J* = 6.7 Hz, 1H, C*H* chiral center), 3.84 (s, 3H, OCH_3), 2.25 (s, 3H, CH_3 tolyl), 2.23 (s, 3H, CH_3 tolyl), 1.13 (d, *J* = 6.8 Hz, 3H, CH_3 chiral center), 0.96 (d, *J* = 6.6 Hz, 3H, CH_3 chiral center).

^{13}C{^{1}H} NMR (151 MHz, 298 K, DMSO-d_6): δ = 160.4, 156.5, 152.4, 138.9, 138.4, 138.2, 136.7, 135.9, 135.8, 134.3, 134.1, 131.0 (2C), 130.6, 130.3, 130.0, 129.8, 129.0, 128.6 (2C), 128.50, 128.49, 128.46, 127.6, 125.7, 124.2, 123.9, 123.2, 121.9, 121.6, 119.4, 119.1, 114.7 (2C), 106.4, 104.4, 93.4 (d, *J* = 4.9 Hz), 90.2 (d, *J* = 6.1 Hz), 76.5 (d, *J* = 5.5 Hz), 74.3, 72.2, 55.4, 20.7 (2C), 19.5, 18.4.

HRMS (ESI) *m/z*: ([M-^{127}I]$^+$) calcd for $C_{92}H_{74}O_6{}^{103}Rh_2{}^{127}I_3$ 1861.0724, found 1861.0714 (Δ = -0.54 ppm).

IR (ATR): 2952 (w), 2917 (w), 2862 (w), 2862 (w), 2832 (w), 2797 (w), 1601 (m), 1581 (m-w), 1515 (m), 1482 (w), 1459 (w), 1435 (m), 1412 (m-s), 1385 (w), 1365 (w), 1335 (w), 1305 (w), 1250 (vs), 1175 (s), 1141 (w-m), 1101 (w), 1056 (m), 1022 (m), 1006 (m), 987 (w), 961 (w), 942 (w), 860 (w), 829 (s), 754 (s), 739 (m), 726 (w), 679 (w), 646 (w), 634 (w), 604 (w), 590 (w-m), 514 (m), 488 (m), 454 (m-w), 429 (m-w) cm^{-1}.

Satisfactory **EA** was not obtained.

UV/VIS (THF): λ_{max} (log ε) = 267 (4.90), 337 (4.71), 528 (4.15) nm.

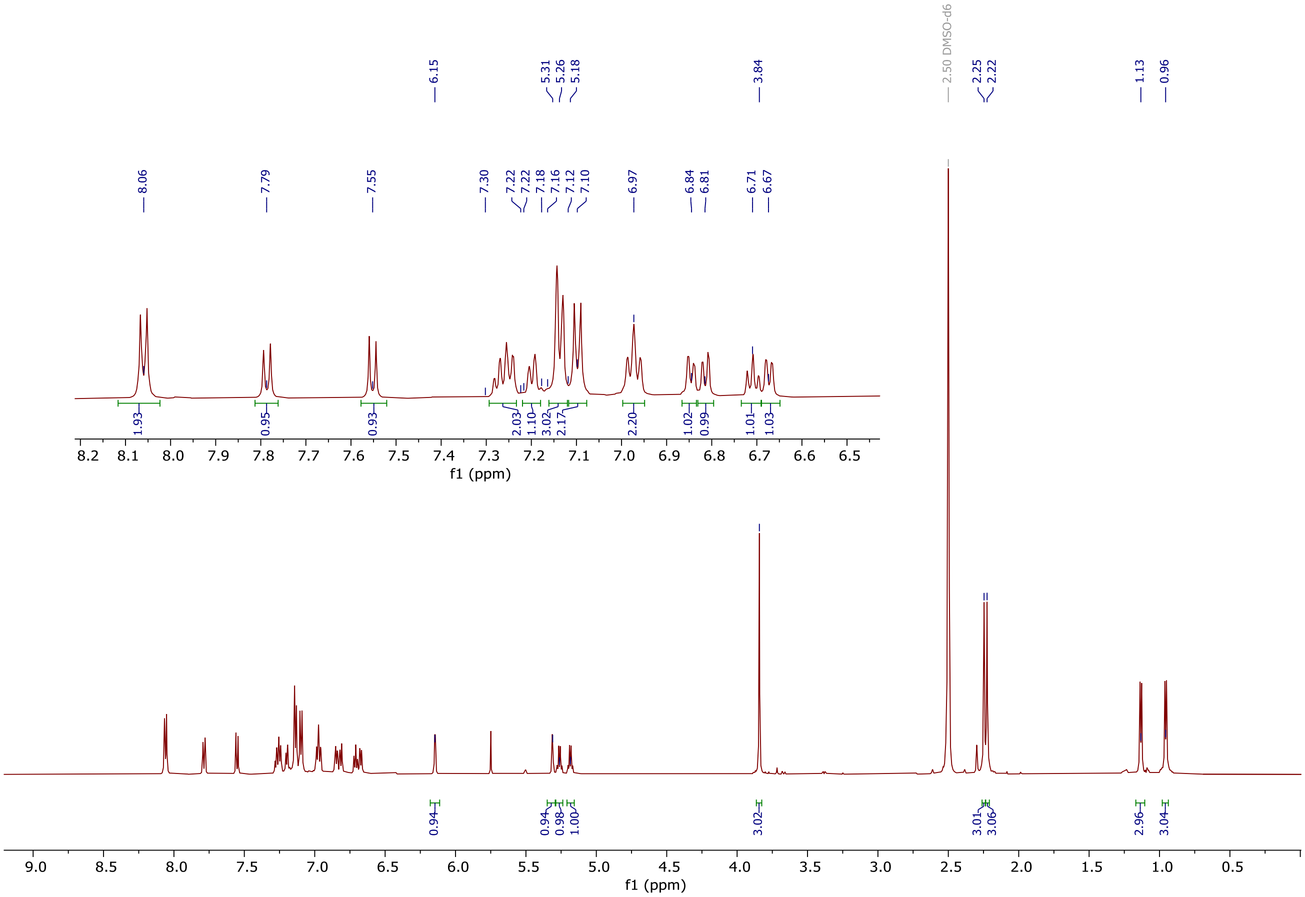

Figure S104: ^{1}**H NMR of** (-)-(M,R,R,R_p)-**25e** (600 MHz, 298 K, DMSO-d_6).

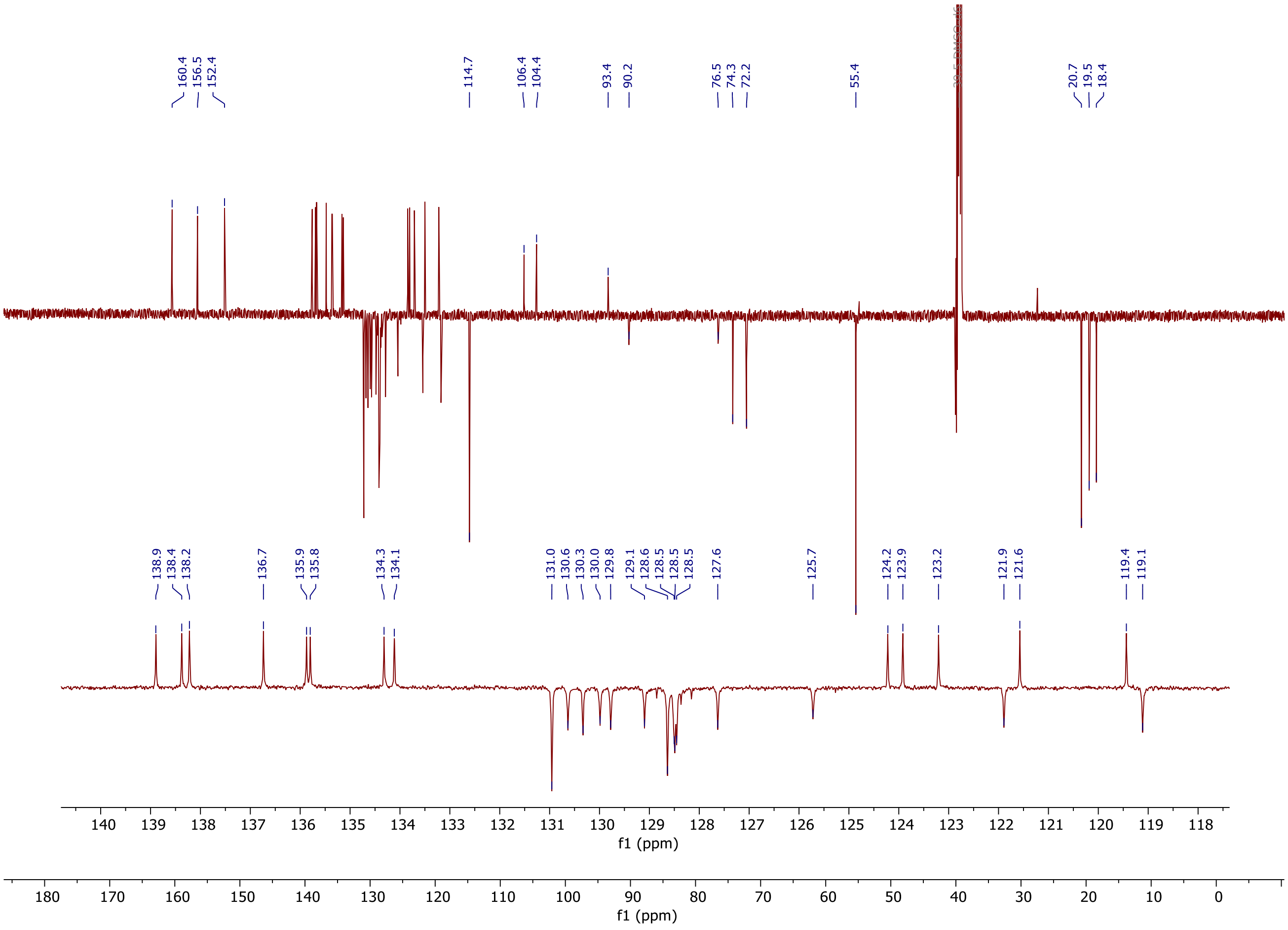

Figure S105: ^{13}**C NMR of** (-)-(M,R,R,R_p)-**25e** (151 MHz, 298 K, DMSO-d_6).

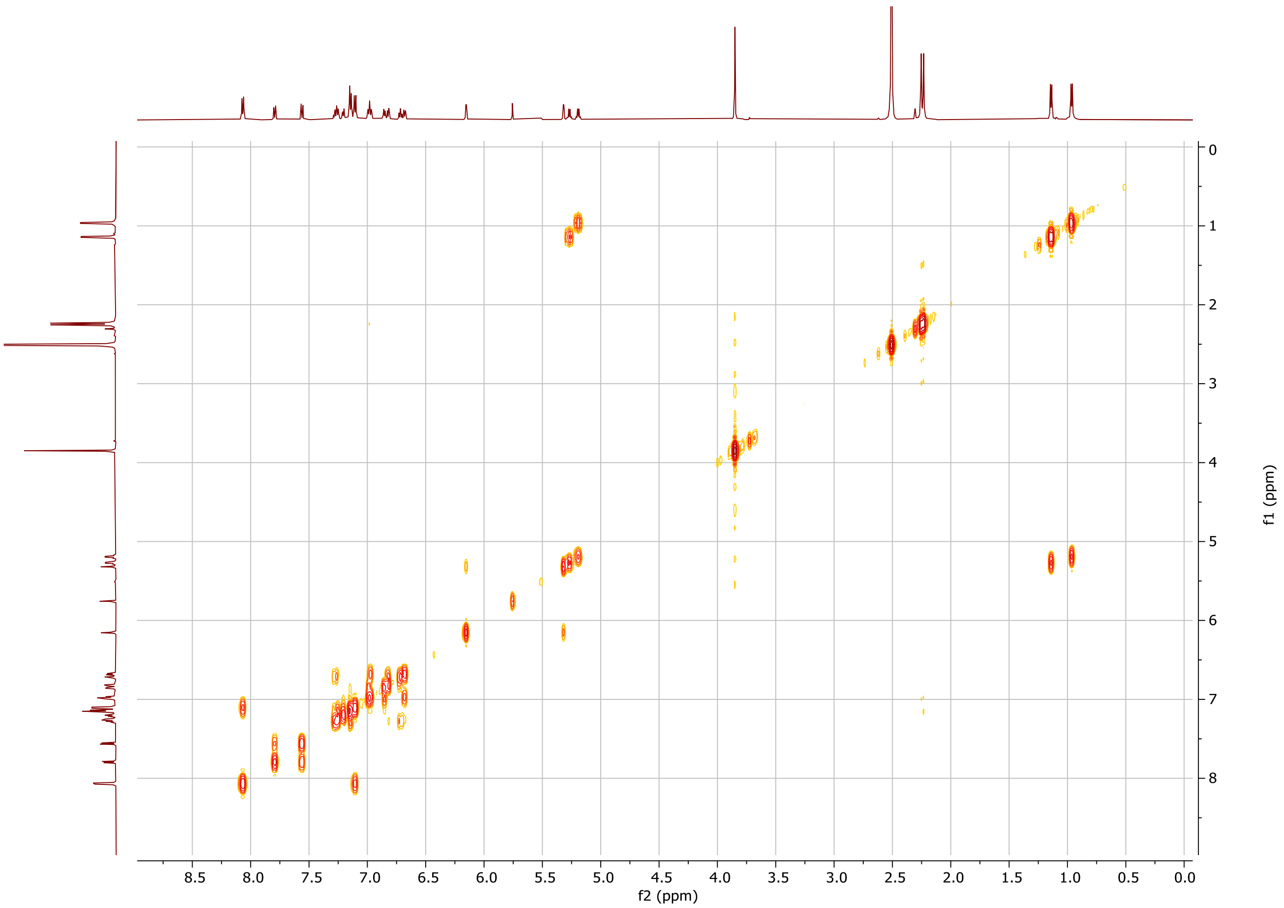

Figure S106: ^{1}H-^{1}H COSY of (-)-(*M,R,R,R$_p$*)-**25e** (600 MHz/600 MHz, 298 K, DMSO-d_6).

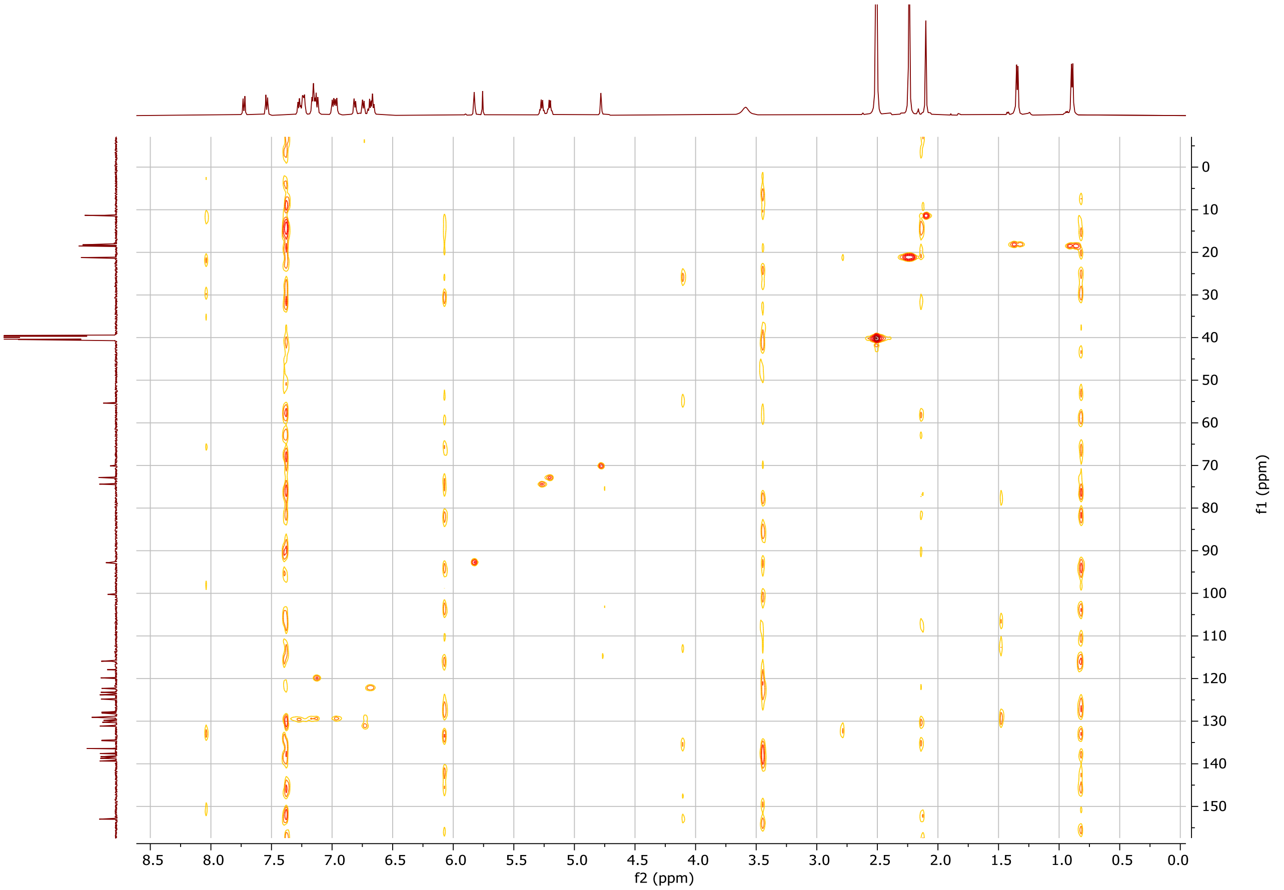

Figure S107: ^{1}H-^{13}C HSQC of (-)-(*M,R,R,R$_p$*)-**25e** (600 MHz/151 MHz, 298 K, DMSO-d_6).

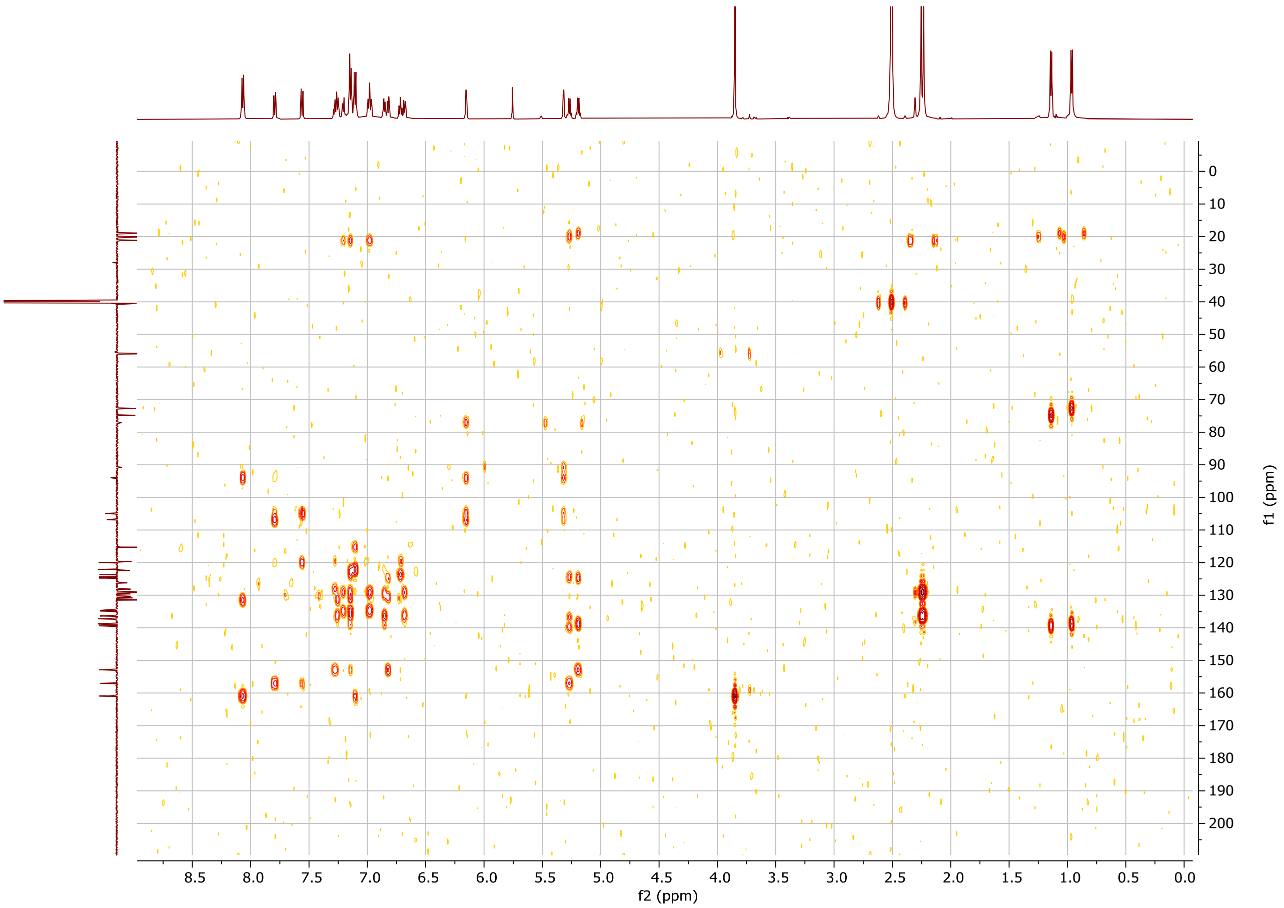

Figure S108: ^{1}H-^{13}C HMBC of (-)-(*M,R,R,R$_p$*)-**25e** (600 MHz/151 MHz, 298 K, DMSO-*d$_6$*).

Compound (-)-(M,R,R,R_p)-**25a(Br)**

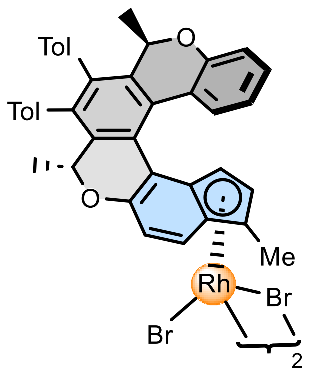

Prepared according to **GP4** starting from (-)-(M,R,R,R_p)-**22a** (70 mg, 0.09 mmol, 1.0 equiv.) and bromine (11.8 µL, 36.9 mg, 0.23 mmol, 2.5 equiv.) to give (-)-(M,R,R,R_p)-**25a(Br)** (63 mg, 85%) as a dark red amorphous solid.

$[\alpha]^{20}_D$ -2870 (*c* 0.010, THF).

^{1}H NMR (400 MHz, 298 K, DMSO-d_6): δ = 7.72 (d, *J* = 8.9 Hz, 1H, CH^{Ar}), 7.53 (d, *J* = 8.9 Hz, 1H, CH^{Ar}), 7.30 – 7.20 (m, 3H, *o*- and *m*-proton of *p*-tolyl overlapping with CH^{Ar}), 7.17 – 7.09 (m, 3H, *o*- and *m*-proton of *p*-tolyl overlapping with CH^{Ar}), 6.97 (t, *J* = 9.0 Hz, 2H, *m*-protons of *p*-tolyl), 6.80 (dd, *J* = 7.8, 1.9 Hz, 1H, *o*-proton of *p*-tolyl), 6.76 – 6.62 (m, 3H, *o*-proton of *p*-tolyl overlapping with 2 CH^{Ar}), 5.83 (d, *J* = 2.7 Hz, 1H, Cp*H*), 5.26 (q, *J* = 6.7 Hz, 1H, C*H* chiral center), 5.19 (q, *J* = 6.5 Hz, 1H, C*H* chiral center), 4.77 (d, *J* = 2.7 Hz, 1H, Cp*H*), 2.23 (s, 6H, CH_3 tolyl), 2.09 (s, 3H, CH_3Cp), 1.34 (d, *J* = 6.8 Hz, 3H, CH_3 chiral center), 0.88 (d, *J* = 6.6 Hz, 3H, CH_3 chiral center).

^{13}C{^{1}H} NMR (151 MHz, 298 K, DMSO-d_6): δ = 158.9, 152.5, 138.8, 138.2, 137.8, 137.1, 135.9 (2C), 134.1, 134.0, 130.6, 130.5, 129.8, 129.3, 128.74, 128.65, 128.59, 128.58, 128.51, 128.45, 127.6, 127.4, 124.3, 123.3, 122.7, 121.8, 119.3, 117.4, 115.4, 99.7, 92.3, 73.9, 72.3, 69.6 (d, *J* = 7.0 Hz), 54.9, 20.71, 20.69, 18.0, 17.7, 10.8.

HRMS (ESI) *m/z*: ([M-^{79}Br]$^+$) calcd for $C_{80}H_{66}O_4{}^{103}Rh_2{}^{79}Br_3$ 1533.0616, found 1533.0612 (Δ = -0.28 ppm).

IR (ATR): 3019 (w), 2973 (w), 2922 (w-m), 2866 (w), 1586 (m-s), 1538 (w), 1515 (m), 1482 (m), 1463 (w), 1443 (w), 1417 (vs), 1364 (m), 1336 (w), 1295 (w), 1261 (m), 1219 (s), 1183 (w), 1143 (m-s), 1104 (m-s), 1088 (w), 1063 (vs), 1031 (m), 1005 (m), 990 (w), 944 (w), 907 (w), 862 (m), 834 (s), 817 (s), 797 (s), 766 (s), 742 (s), 725 (w), 680 (m), 634 (m), 602 (w-m), 589 (w-m), 567 (w), 525 (m), 493 (m), 478 (m), 449 (w-m), 426 (m) cm^{-1}.

Satisfactory **EA** was not obtained.

UV/VIS (THF): λ_{max} (log ε) = 272 (4.86), 528 (3.72) nm.

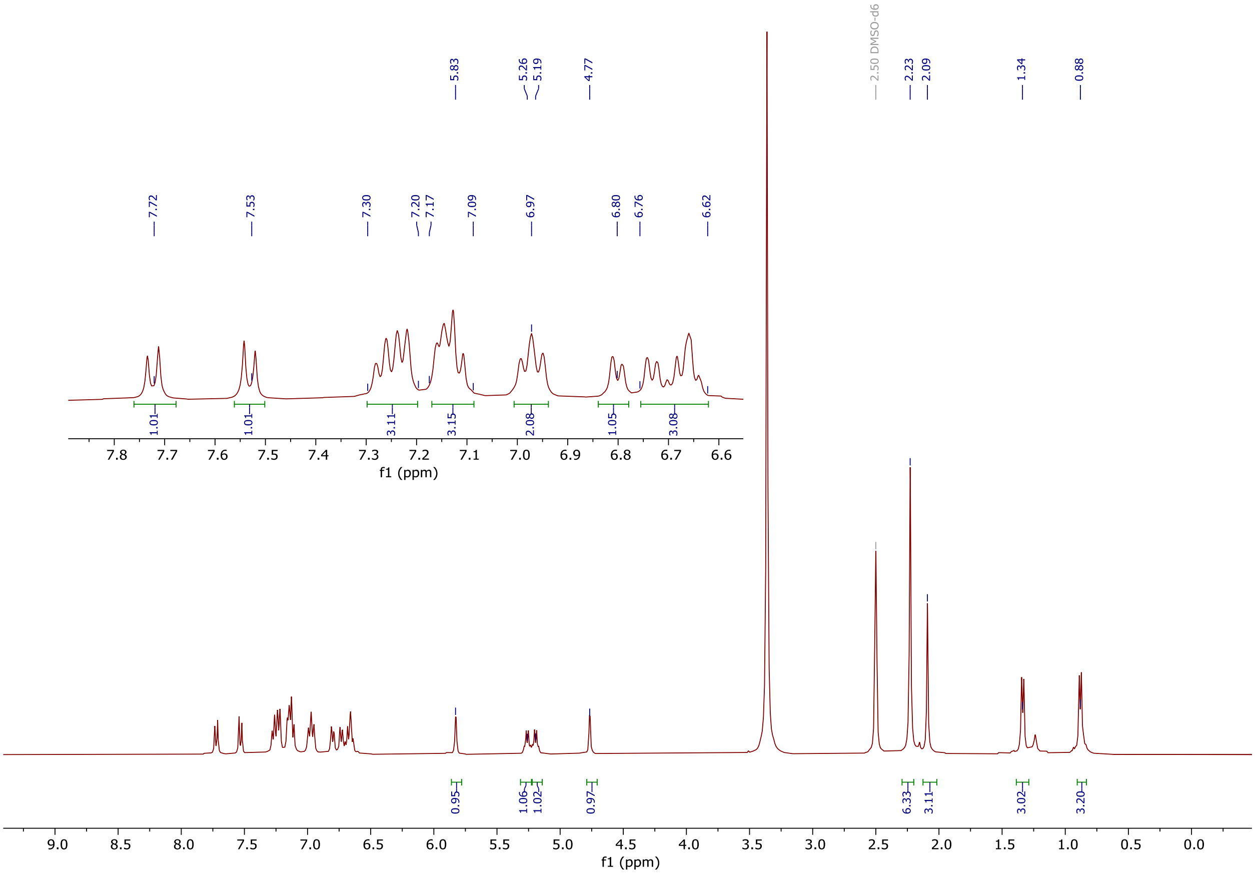

**Figure S109: **

Figure S109: [1]**H NMR of** (-)-(*M,R,R,R$_p$*)-**25a**(Br) (400 MHz, 298 K, DMSO-d_6).

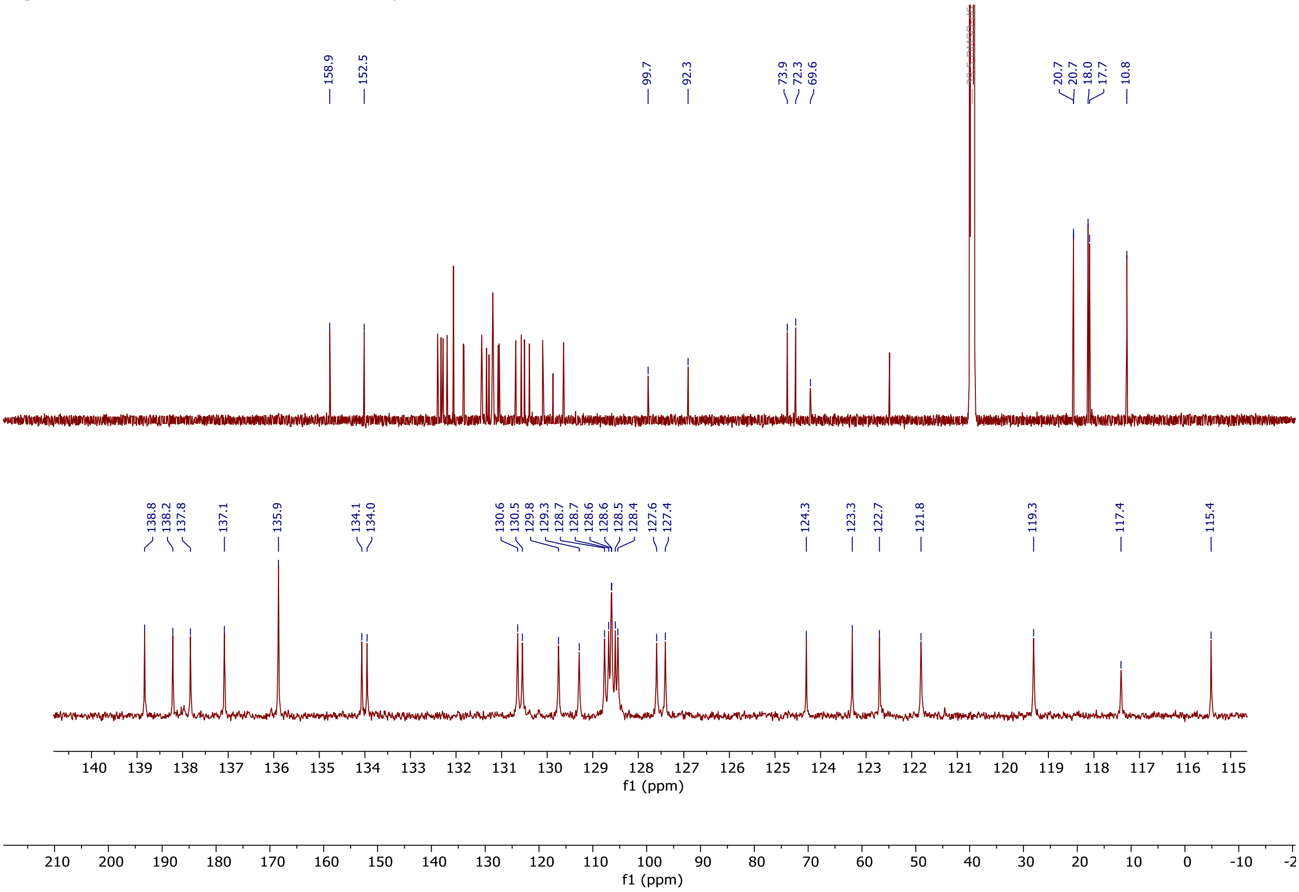

Figure S110: [13]**C NMR of** (-)-(*M,R,R,R$_p$*)-**25a**(Br) (151 MHz, 298 K, DMSO-d_6).

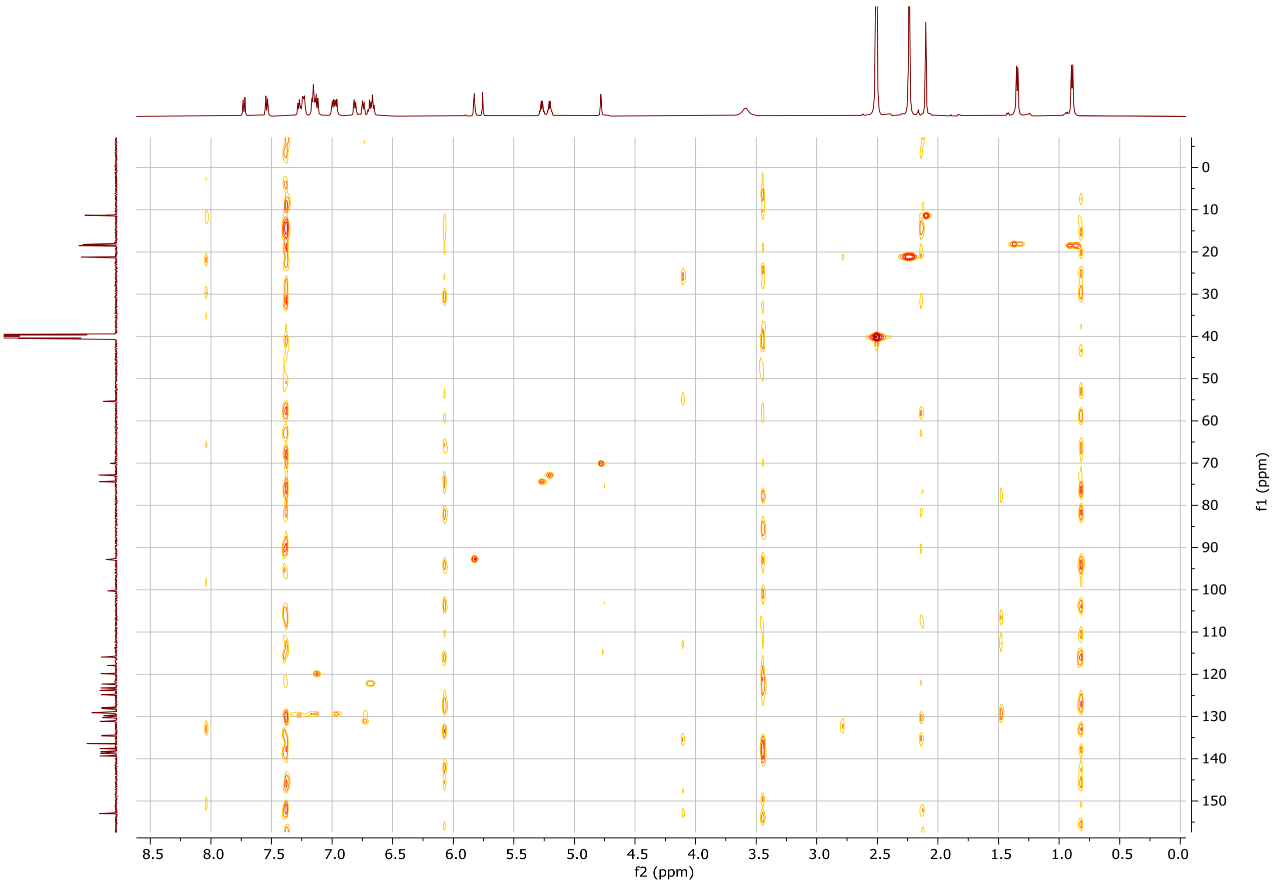

Figure S111: ^{1}H-^{13}C HSQC of (-)-(*M,R,R,R$_p$*)-**25a**(Br) (600 MHz/151 MHz, 298 K, DMSO-*d$_6$*).

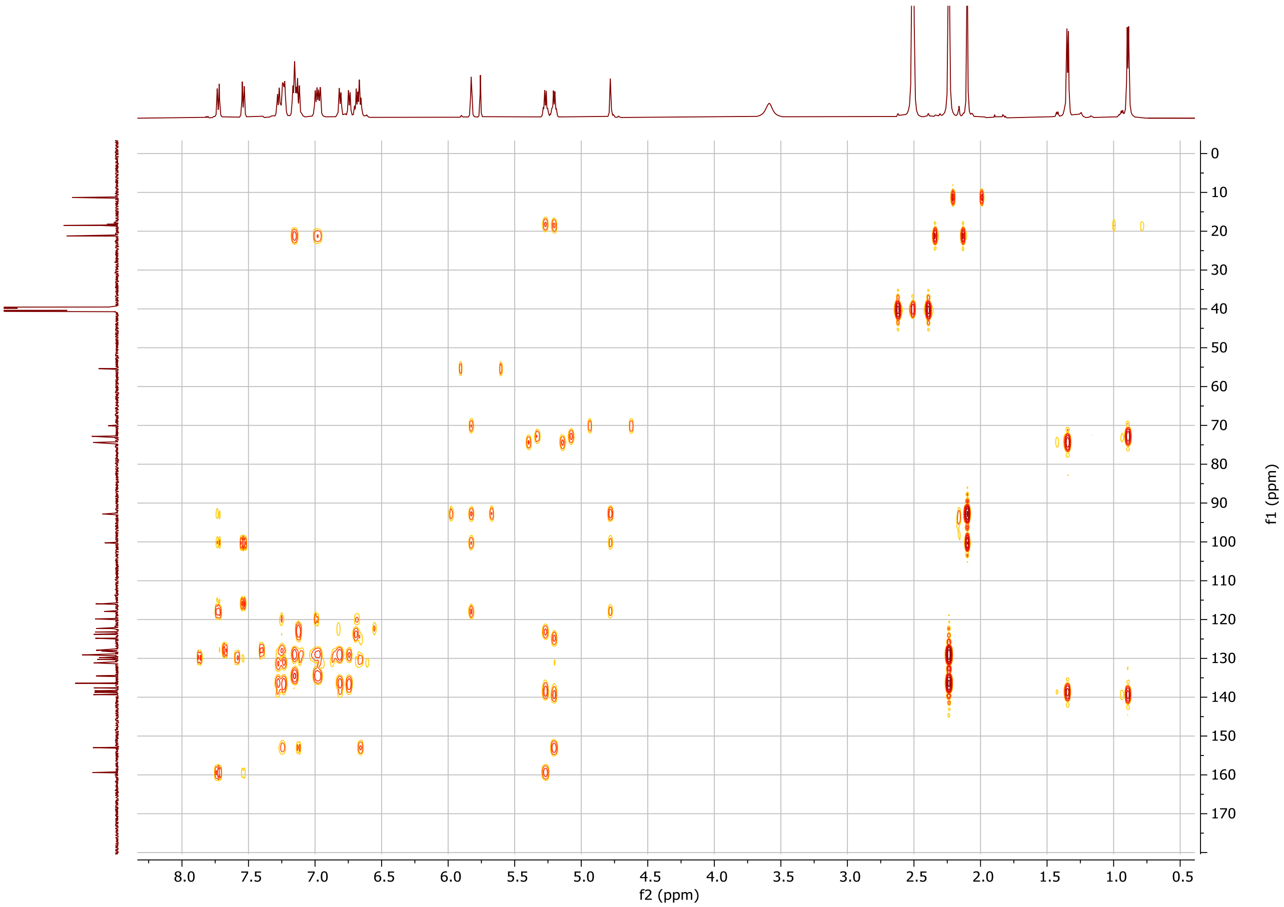

Figure S112: ^{1}H-^{13}C HMBC of (-)-(*M,R,R,R$_p$*)-**25a**(Br) (600 MHz/151 MHz, 298 K, DMSO-*d$_6$*).

Compound (-)-(*M,R,R,Rp*)-**25b**

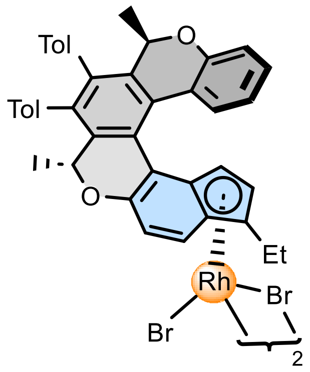

Prepared according to **GP4** starting from (-)-(*M,R,R,R*$_p$)-**22b** (30 mg, 0.04 mmol, 1.0 equiv.) and bromine (5.0 µL, 15.5 mg, 0.1 mmol, 2.5 equiv.) to give (-)-(*M,R,R,Rp*)-**25b** (31.0 mg, 97%) as a dark red amorphous solid.

[α]$^{20}_{D}$ -2250 (*c* 0.010, THF).

^{1}H NMR (600 MHz, 298 K, DMSO-*d*$_6$): δ = 7.77 (d, *J* = 8.9 Hz, 1H, C*H*^{Ar}), 7.52 (d, *J* = 8.9 Hz, 1H, C*H*^{Ar}), 7.31 (dd, *J* = 7.6, 2.0 Hz, 1H, *o*-proton of *p*-tolyl), 7.27 – 7.22 (m, 2H, *o*-proton of *p*-tolyl overlapping with C*H*^{Ar}), 7.18 – 7.10 (m, 3H, *m*-protons of *p*-tolyl overlapping with C*H*^{Ar}), 7.00 – 6.97 (m, 2H, *m*-protons of *p*-tolyl), 6.81 (dd, *J* = 7.8, 1.9 Hz, 1H, *o*-proton of *p*-tolyl), 6.76 (dd, *J* = 7.8, 1.9 Hz, 1H, *o*-proton of *p*-tolyl), 6.66 – 6.50 (m, 2H, C*H*^{Ar}), 5.81 (d, *J* = 2.7 Hz, 1H, Cp*H*), 5.29 (q, *J* = 6.8 Hz, 1H, C*H* chiral center), 5.20 (q, *J* = 6.7 Hz, 1H, C*H* chiral center), 4.80 (d, *J* = 2.7 Hz, 1H, Cp*H*), 2.68 (dq, *J* = 15.1, 7.4 Hz, 1H, C*H*$_2$ ethyl), 2.49 – 2.43 (m, 1H, C*H*$_2$ ethyl, overlapping DMSO signal), 2.24 (s, 6H, C*H*$_3$ tolyl), 1.40 (d, *J* = 6.8 Hz, 3H, C*H*$_3$ chiral center), 1.24 (t, *J* = 7.5 Hz, 3H, C*H*$_3$ ethyl), 0.89 (d, *J* = 6.7 Hz, 3H, C*H*$_3$ chiral center).

^{13}C{^{1}H} NMR (151 MHz, 298 K, DMSO-*d*$_6$): δ = 159.4, 152.6, 139.0, 137.9, 137.8, 137.2, 135.9 (2C), 134.0, 133.9, 130.7, 130.5, 130.2, 129.8, 128.8, 128.62, 128.59 (2C), 128.5, 128.4, 127.6, 127.0, 124.3, 123.3, 122.5, 121.4, 120.4, 119.3, 114.6, 99.0 (d, *J* = 5.9 Hz), 96.7, 90.3 (d, *J* = 7.6 Hz), 73.9, 72.3, 69.3 (d, *J* = 7.1 Hz), 20.71, 20.70, 18.6, 17.9, 17.4, 13.0.

HRMS (ESI) *m/z*: ([M-^{79}Br]$^{+}$) calcd for $C_{82}H_{70}O_4{}^{103}Rh_2{}^{79}Br_3$ 1561.0929, found 1561.0928 (Δ = -0.05 ppm).

IR (ATR): 3020 (w), 2968 (m), 2922 (w-m), 2866 (w), 1586 (m), 1538 (w), 1515 (w-m), 1481 (w), 1445 (m), 1417 (s), 1366 (m-s), 1336 (w), 1289 (w), 1271 (m), 1248 (m), 1228 (m), 1213 (s), 1182 (w), 1143 (m), 1101 (m-s), 1064 (s), 1033 (m), 1021 (w), 1006 (m), 985 (w), 903 (w), 860 (m), 834 (m), 817 (m-s), 781 (s), 758 (s), 743 (s), 681 (m), 936 (w), 627 (w), 601 (w), 830 (m), 514 (m), 491 (m), 481 (wm), 458 (w-m), 427 (m) cm^{-1}.

Satisfactory **EA** was not obtained.

UV/VIS (THF): λ_{max} (log ε) = 272 (4.80), 528 (3.66) nm.

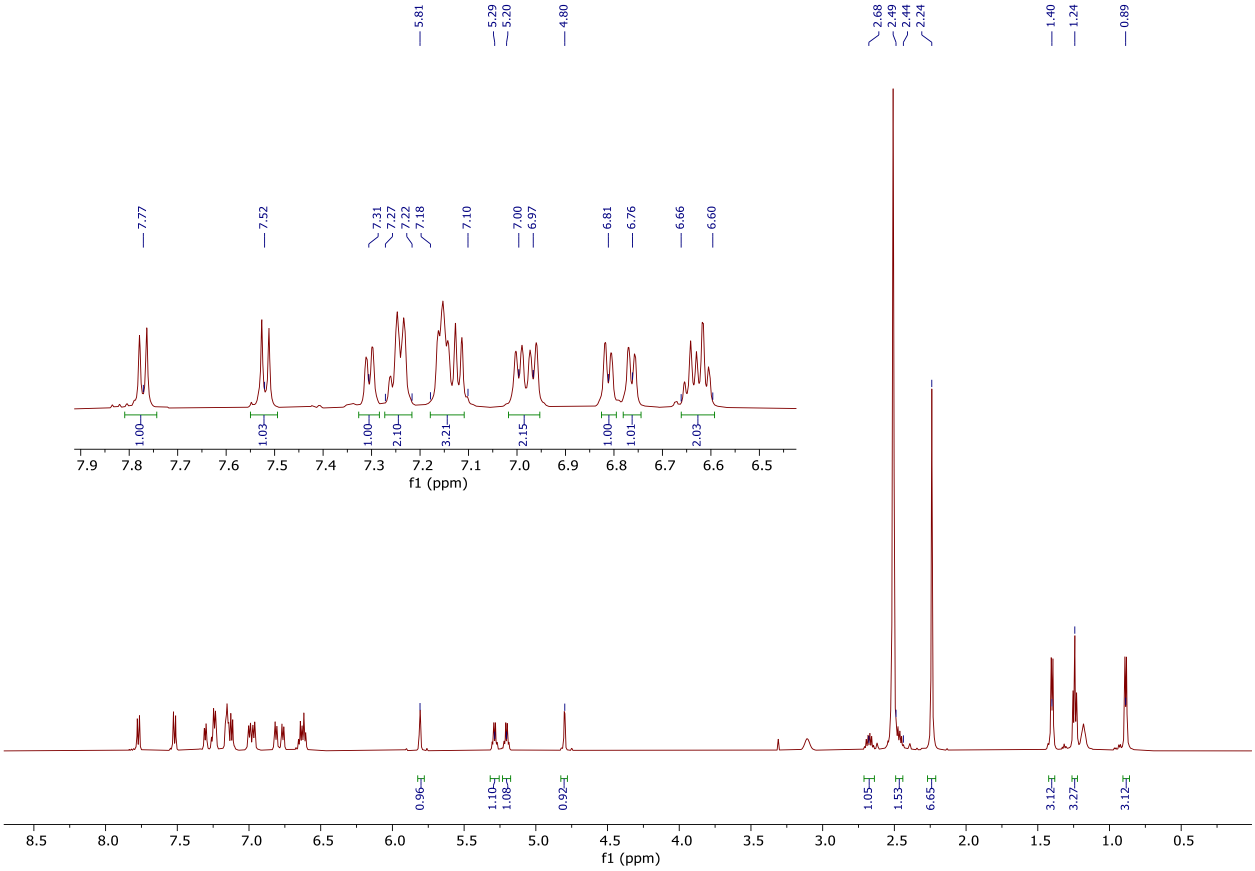

Figure S113: ^{1}H NMR of (-)-(*M,R,R,Rp*)-**25b** (600 MHz, 298 K, DMSO-d_6).

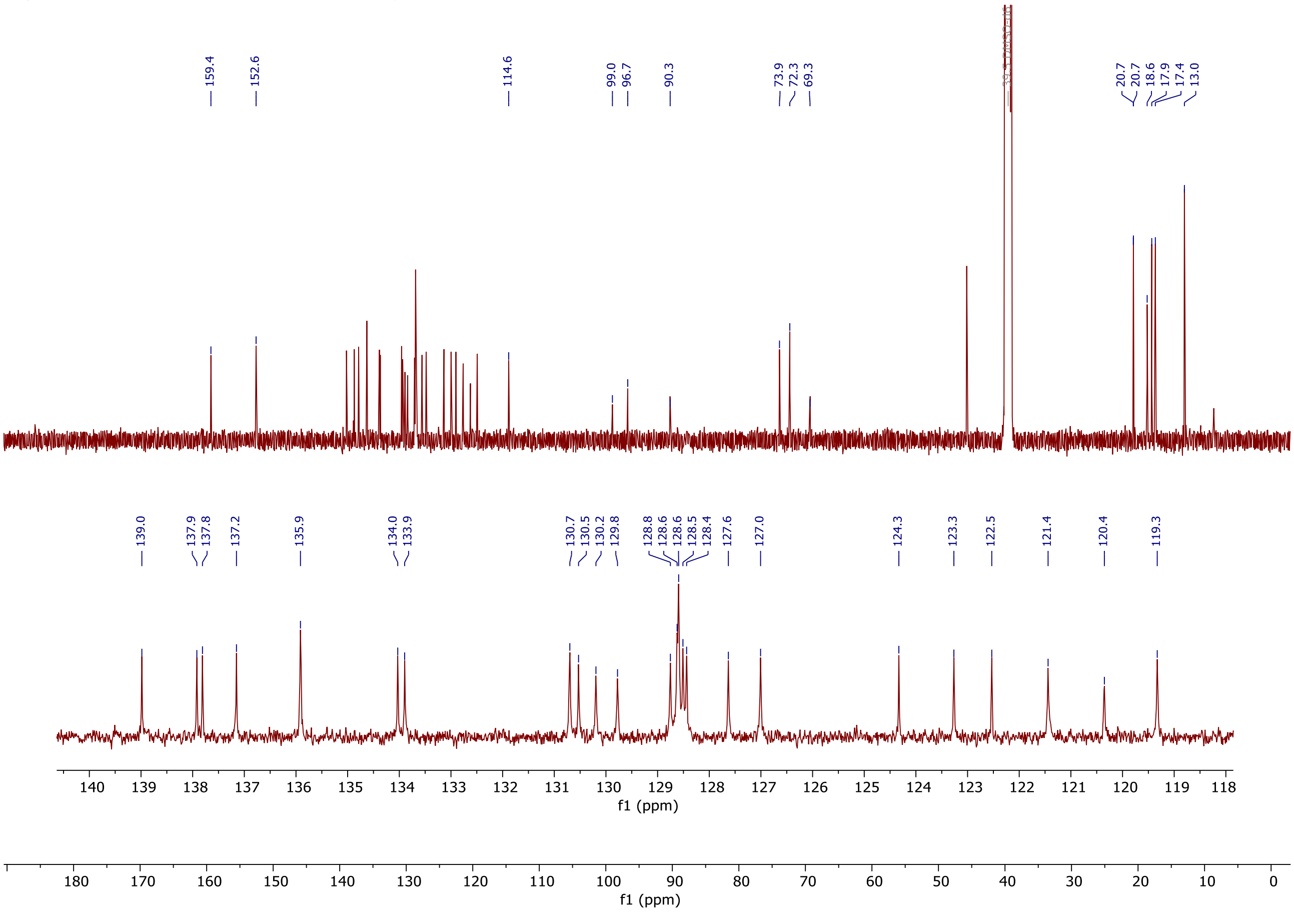

Figure S114: ^{13}C NMR of (-)-(*M,R,R,Rp*)-**25b** (151 MHz, 298 K, DMSO-d_6).

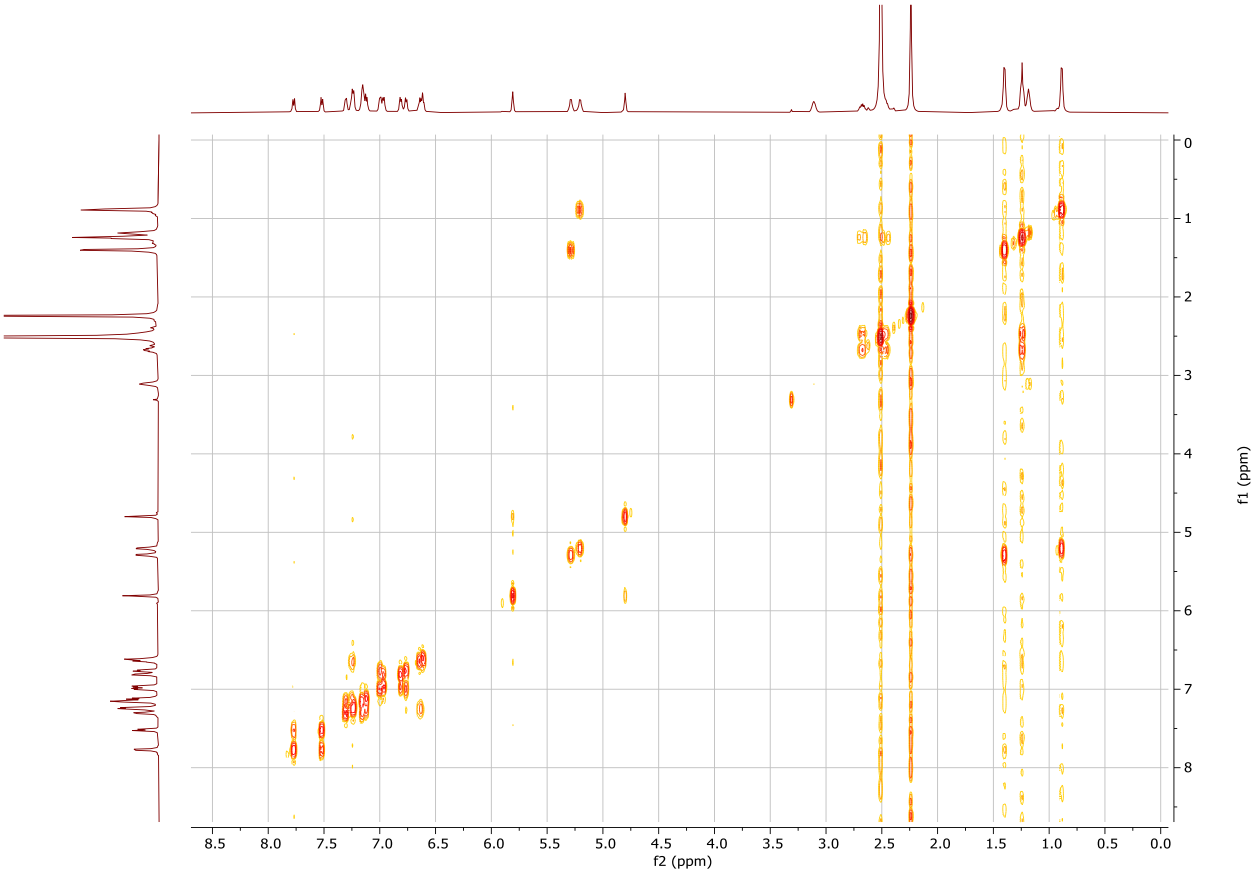

Figure S115: ^{1}H-^{1}H COSY of (-)-(*M,R,R,Rp*)-**25b** (600 MHz/600 MHz, 298 K, DMSO-d_6).

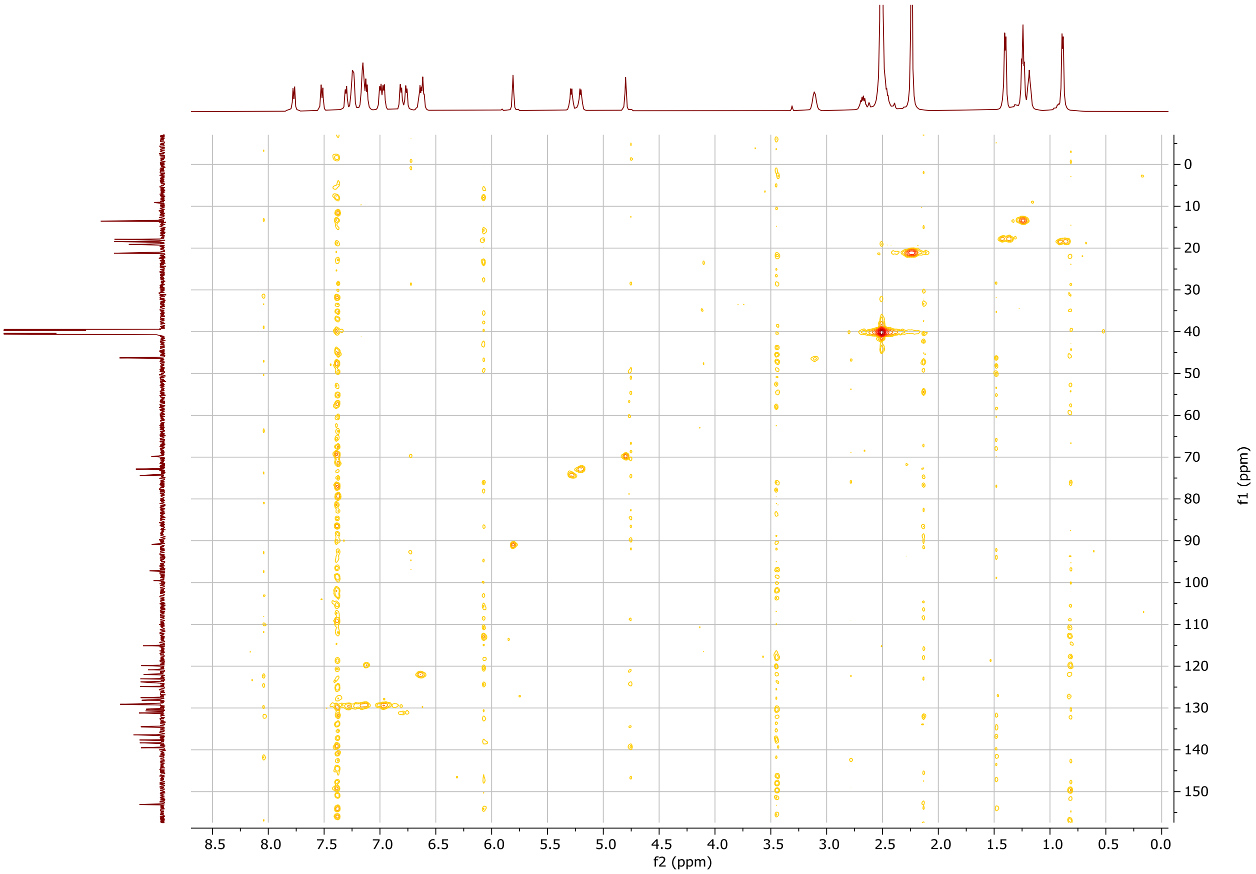

Figure S116: ^{1}H-^{13}C HSQC of (-)-(*M,R,R,Rp*)-**25b** (600 MHz/151 MHz, 298 K, DMSO-d_6).

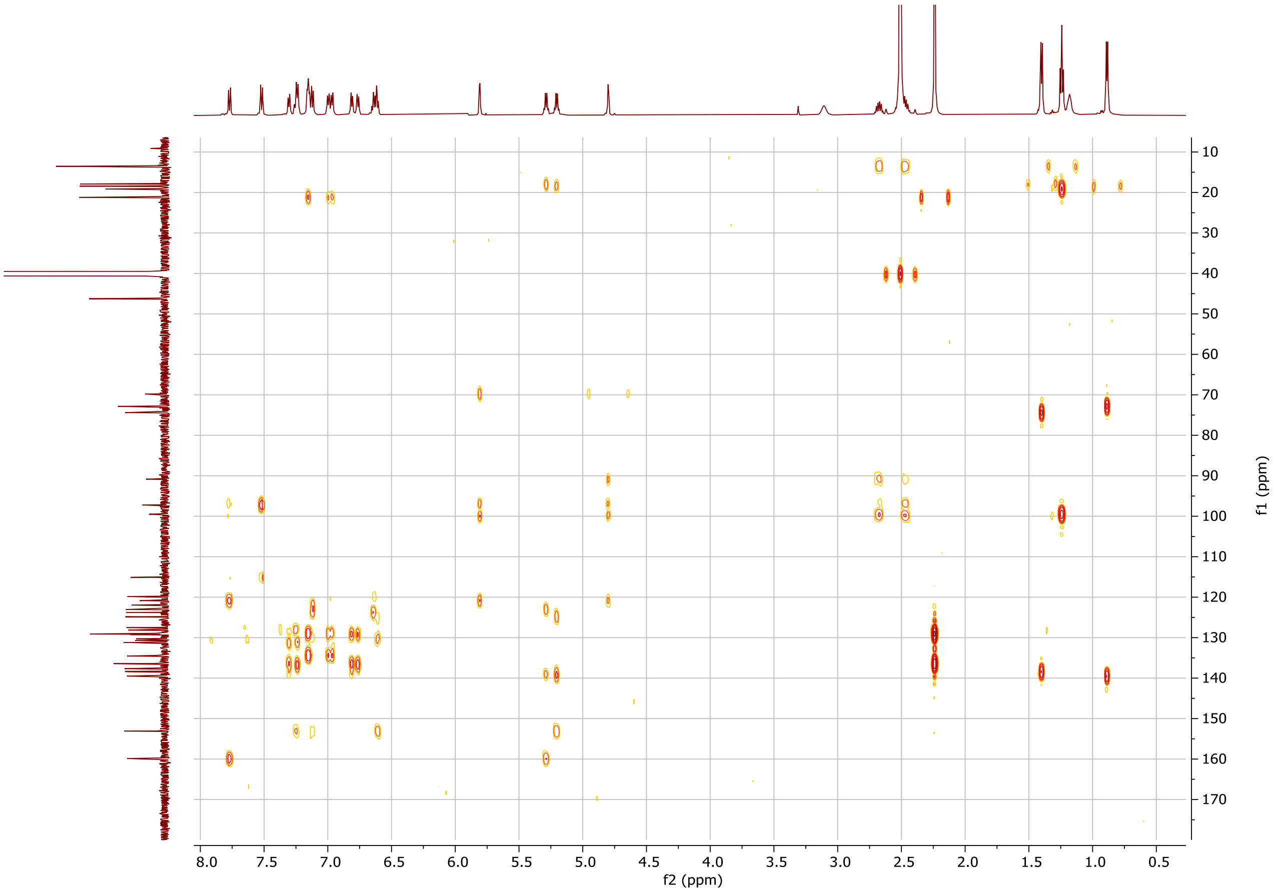

Figure S117: ^{1}H-^{13}C HMBC of (-)-(*M,R,R,Rp*)-**25b** (600 MHz/151 MHz, 298 K, DMSO-d_6).

Compound (-)-(M,R,R,R_p)-**25c**

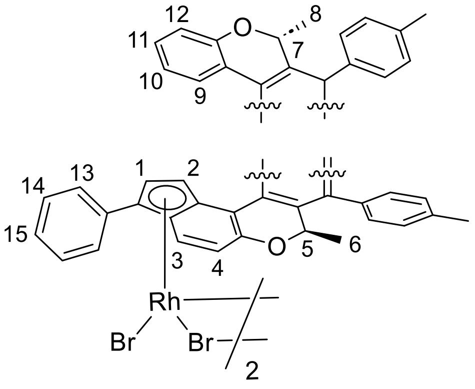

Prepared according to **GP4** starting from **(-)-(M,R,R,R_p)-22c** (50 mg, 0.06 mmol, 1.0 equiv.) and bromine (8.1 µL, 25.3 mg, 0.16 mmol, 2.5 equiv.) to give (-)-(M,R,R,R_p)-**25c** (41.2 mg, 78%) as a dark red amorphous solid.

$[\alpha]^{20}_{D}$ -3380 (*c* 0.010, THF).

^{1}H NMR (600 MHz, 298 K, THF-d_8): δ = 7.96 (d, *J* = 7.6 Hz, 2H, CH^{13}), 7.66 (d, *J* = 9.0 Hz, 1H, CH^3), 7.55 (d, *J* = 9.0 Hz, 1H, CH^4), 7.49 (t, *J* = 7.5 Hz, 1H, CH^{15}), 7.39 (t, *J* = 7.6 Hz, 2H, CH^{14}), 7.25 (dd, *J* = 7.7, 1.9 Hz, 1H, *o*-proton of *p*-tolyl), 7.16 (dd, *J* = 8.2, 1.6 Hz, 1H, *o*-proton of *p*-tolyl), 7.12 (m, 2H, *m*-protons of *p*-tolyl), 7.08 (td, *J* = 7.6, 1.6 Hz, 1H, CH^{11}), 7.01 (dd, *J* = 8.0, 1.3 Hz, 1H, CH^{12}), 6.94 (dd, *J* = 7.8, 1.9 Hz, 1H, *m*-proton of *p*-tolyl), 6.90 (dd, *J* = 7.8, 1.9 Hz, 1H, *m*-proton of *p*-tolyl), 6.79 (dd, *J* = 7.8, 1.7 Hz, 1H, *o*-proton of *p*-tolyl), 6.74 (dd, *J* = 7.7, 1.9 Hz, 1H, *o*-proton of *p*-tolyl), 6.62 (d, *J* = 7.7 Hz, 1H, CH^9), 6.48 (t, *J* = 7.5 Hz, 1H, CH^{10}), 5.46 (d, *J* = 2.8 Hz, 1H, CH^1), 5.38 – 5.32 (m, 2H, CH^2 overlapping CH^5), 5.29 (q, *J* = 6.7 Hz, 1H, CH^7), 2.26 (d, *J* = 2.0 Hz, 6H, CH_3 tolyl), 1.35 (d, *J* = 6.9 Hz, 3H, C$H_3{}^6$), 0.92 (d, *J* = 6.7 Hz, 3H, C$H_3{}^8$).

^{13}C{^{1}H} NMR (151 MHz, 298 K, THF-d_8): δ = 160.8, 153.7, 139.6, 138.6, 138.3, 137.4, 136.2, 136.1, 134.8, 134.6, 131.0, 130.8, 130.3, 129.8 (2C), 129.7, 129.3, 129.2, 129.1, 128.8 (2C), 128.7 (2C), 128.5, 128.4, 128.3, 128.2, 127.9, 124.9, 123.7, 122.5, 120.9, 119.3, 114.6, 91.7, 87.3, 84.7, 75.0, 72.8, 69.6, 20.2 (2C), 19.5, 17.7.

HRMS (ESI) *m/z*: ([M/2-^{79}Br]$^+$) calcd for $C_{45}H_{35}O_2{}^{103}Rh^{79}Br$ 789.0870, found 789.0863 (Δ = -0.83 ppm).

IR (ATR): 3021 (w), 2977 (w), 2923 (w), 2864 (w), 1586 (m), 1515 (m), 1483 (w), 1461 (w), 1445 (m), 1417 (s), 1387 (w), 1365 (w), 1338 (w), 1271 (w), 1255 (m), 1235 (m), 1224 (m), 1204 (w), 1182 (w), 1144 (m), 1102 (m), 1060 (s), 1033 (m), 1008 (m), 989 (w), 963 (w), 944 (w), 896 (w), 862 (m), 834 (m), 817 (m), 765 (vs), 757 (vs), 742 (s), 724 (w), 696 (s), 646 (w-m), 623 (w), 607 (w), 592 (w), 553 (w), 531 (s), 499 (m), 485 (m), 456 (m) cm^{-1}.

Satisfactory **EA** was not obtained.

UV/VIS (THF): λ_{max} (log ε) = 272 (4.88), 314 (4.71), 345 (4.57) nm.

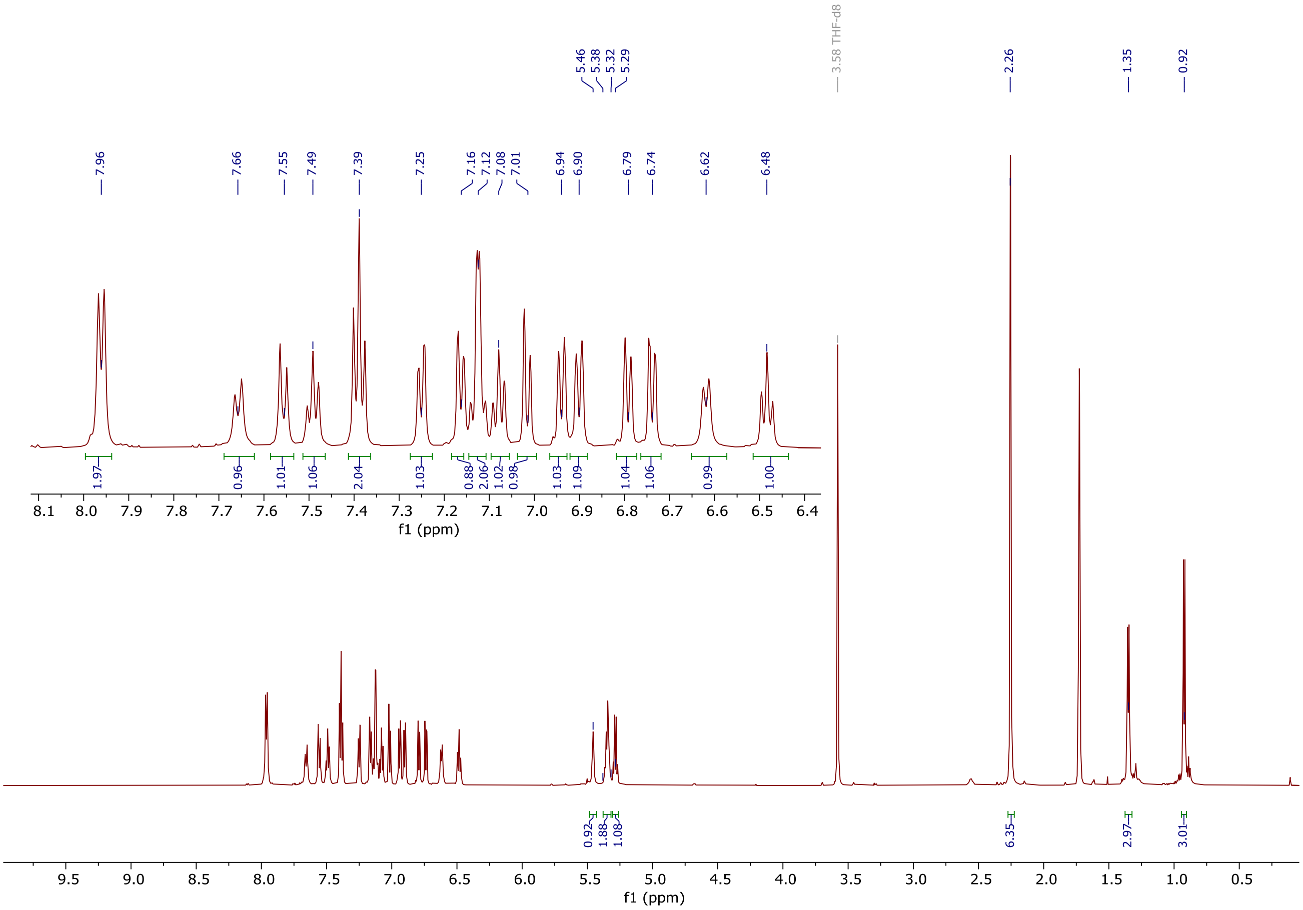

Figure S118: ^{1}H NMR of (-)-(*M,R,R,R$_p$*)-**25c** (600 MHz, 298 K, THF-d_8).

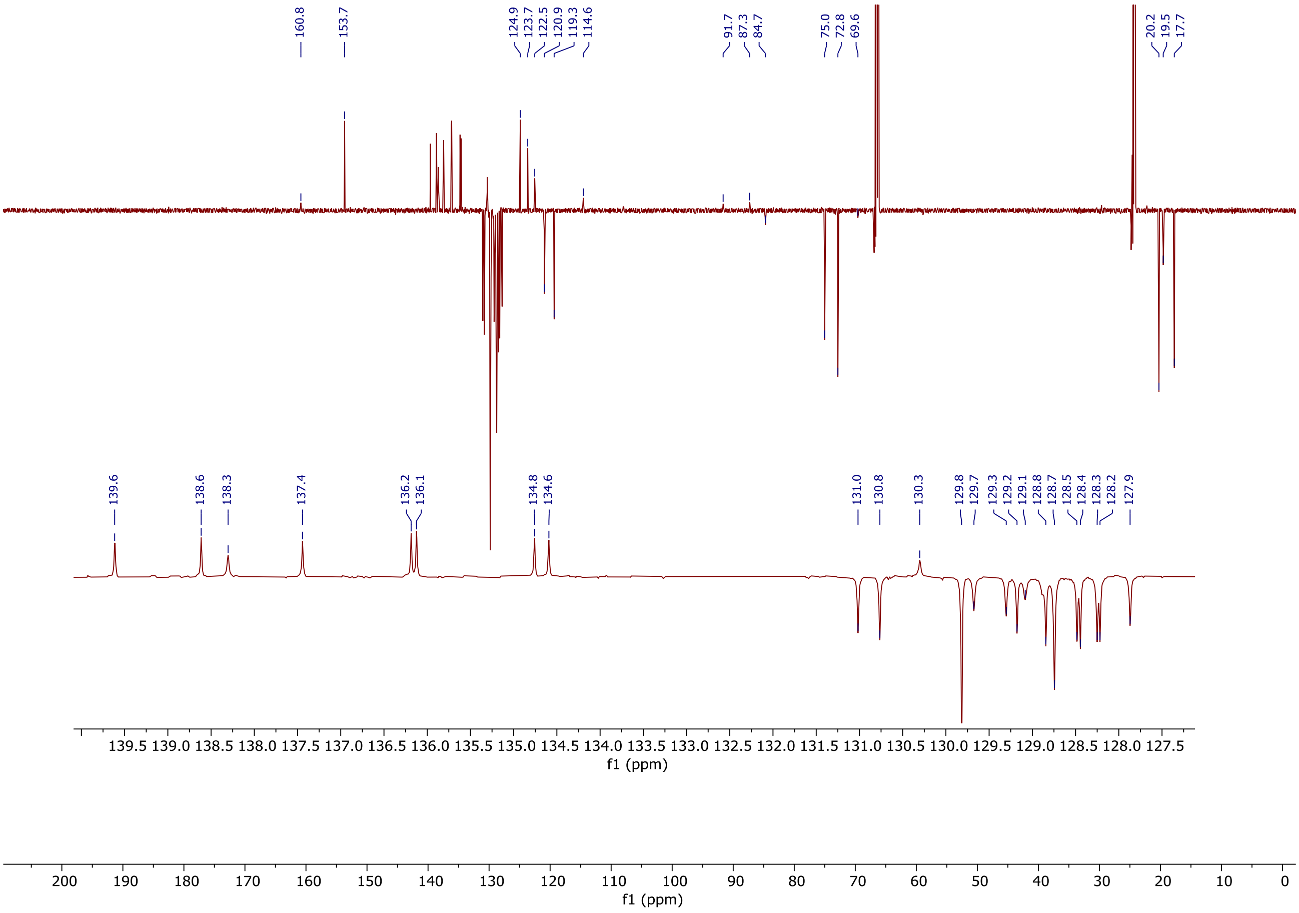

Figure S119: ^{13}C APT NMR of (-)-(*M,R,R,R$_p$*)-**25c** (151 MHz, 298 K, THF-d_8).

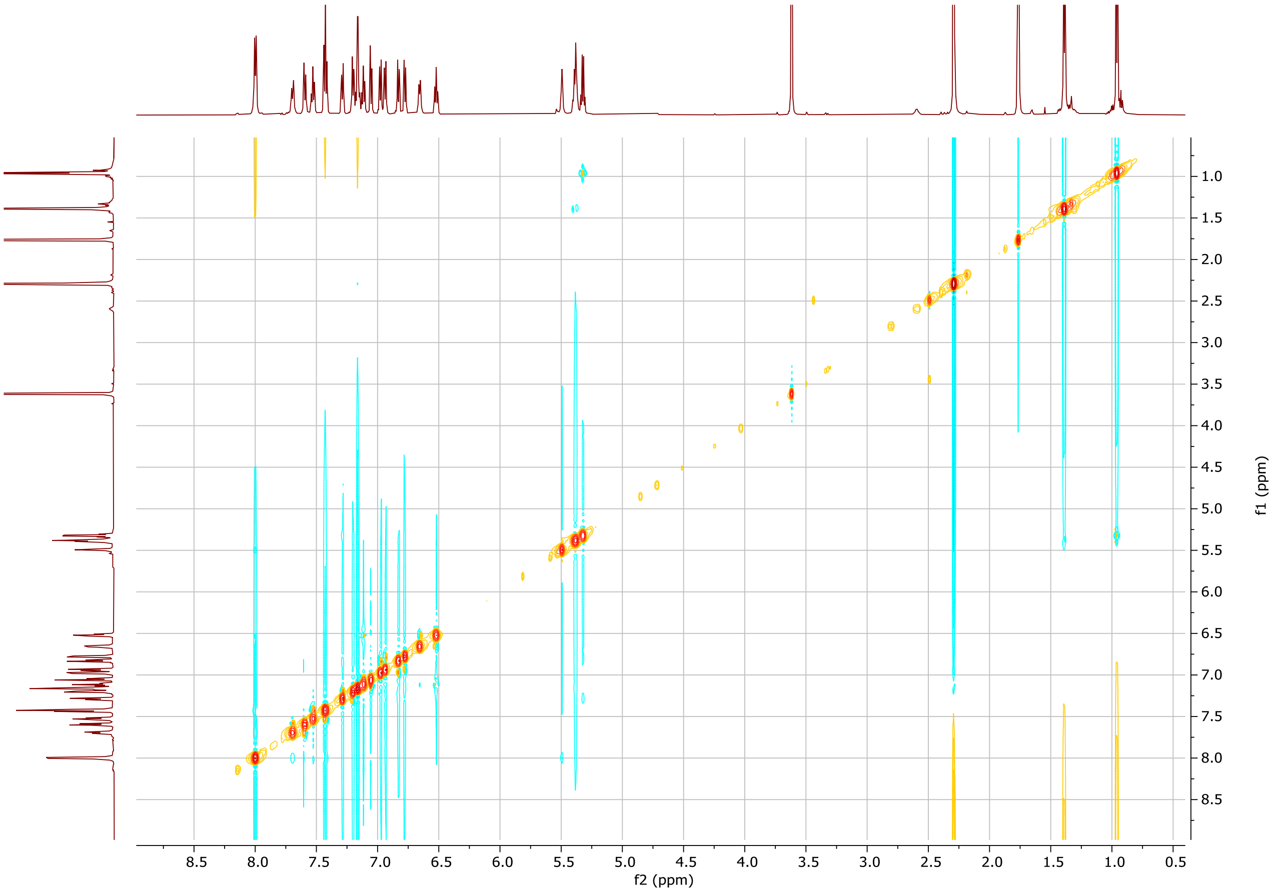

Figure S120: ^{1}H-^{1}H NOESY of (-)-(*M,R,R,R$_p$*)-**25c** (600 MHz/600 MHz, 298 K, DMSO-*d$_6$*).

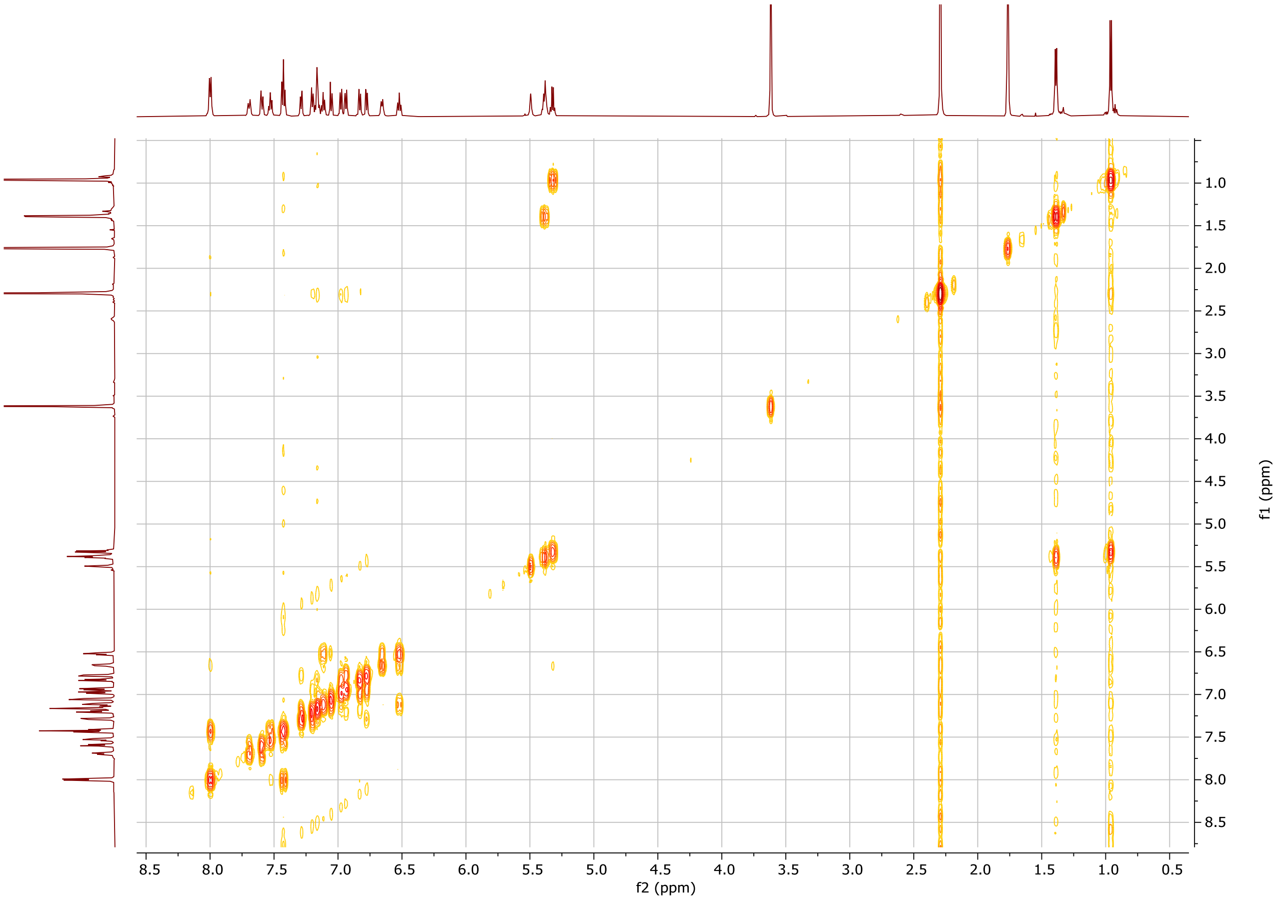

Figure S121: ^{1}H-^{1}H COSY of (-)-(*M,R,R,R$_p$*)-**25c** (600 MHz/600 MHz, 298 K, DMSO-*d$_6$*).

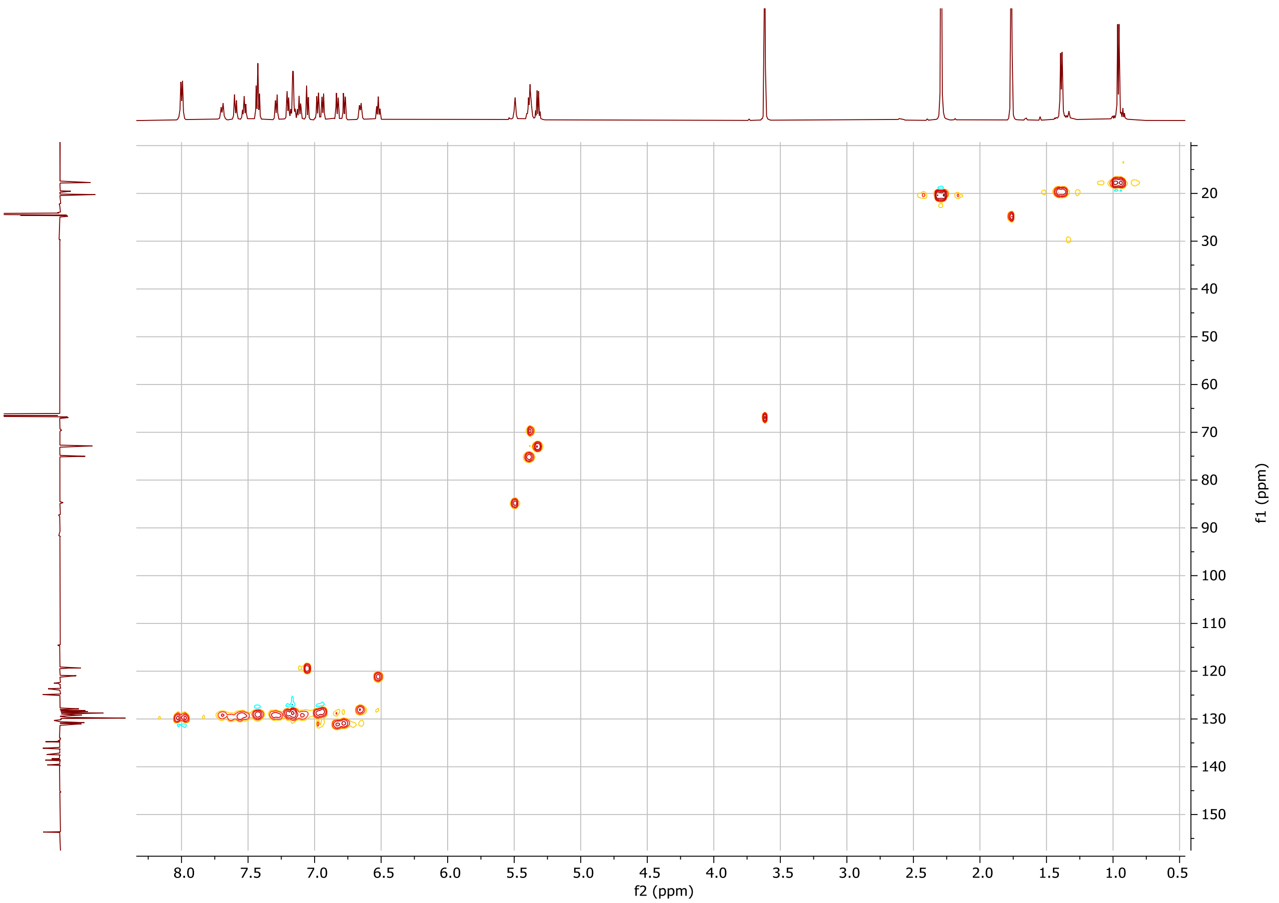

Figure S122: ^{1}H-^{13}C HSQC of (-)-(*M,R,R,R$_p$*)-**25c** (600 MHz/151 MHz, 298 K, DMSO-*d*$_6$).

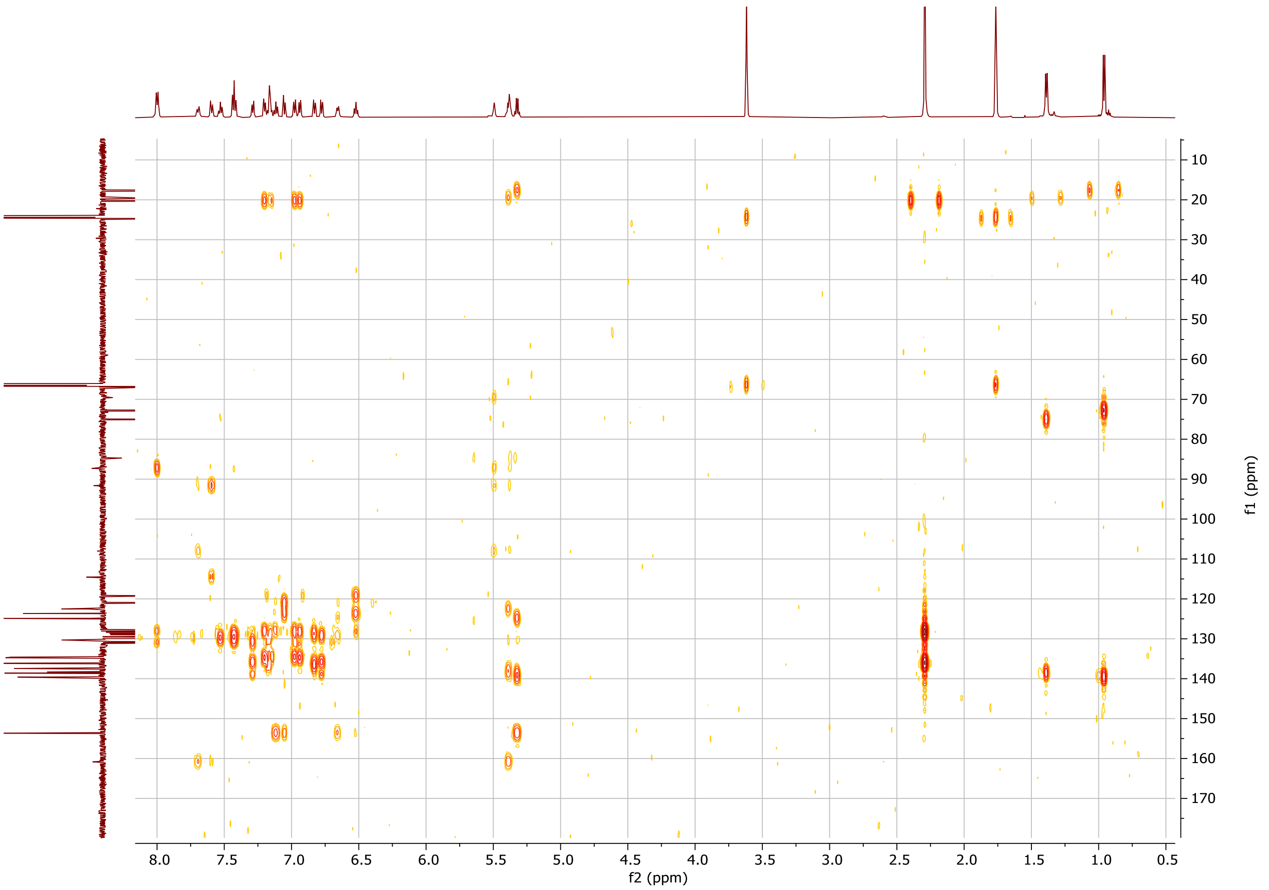

Figure S123: ^{1}H-^{13}C HMBC of (-)-(*M,R,R,R$_p$*)-**25c** (600 MHz/151 MHz, 298 K, DMSO-*d*$_6$).

Compound (-)-(M,R,R,R_p)-**25d**

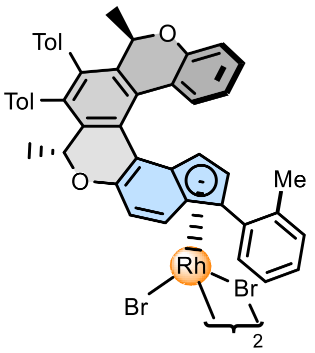

Prepared according to **GP5** starting from (-)-(*M,R,R,Rp*)-**22d** (24 mg, 0.03 mmol, 1.0 equiv.) and bromine (3.7 µL, 11.5 mg, 0.07 mmol, 2.5 equiv.) to give (-)-(M,R,R,R_p)-**25d** (18.9 mg, 74%) as a dark red amorphous solid.

[α]^{20}$_D$ -3160 (*c* 0.010, THF).

^{1}H NMR (600 MHz, 298 K, DMSO-d_6): δ = 8.47 (d, *J* = 7.6 Hz, 1H, *o*-proton of *o*-tolyl), 7.57 (d, *J* = 8.9 Hz, 1H, CH^{Ar}), 7.40 (m, 1H, CH^{Ar}), 7.37 – 7.30 (m, 2H, CH^{Ar} overlapping *o*-proton of *p*-tolyl), 7.30 – 7.22 (m, 2H, CH^{Ar} overlapping *o*-proton of *p*-tolyl), 7.22 – 7.13 (m, 5H, 2 *m*-protons of *p*-tolyl overlapping with 3 CH^{Ar}), 6.99 (m, *m*-protons of *p*-tolyl), 6.85 (m, 1H, *o*-proton of *p*-tolyl), 6.79 (m, 1H, CH^{Ar}), 6.73 (m, 1H, *o*-proton of *p*-tolyl), 6.70 (m, 1H, CH^{Ar}), 6.03 (d, *J* = 2.7 Hz, 1H, Cp*H*), 5.27 – 5.17 (m, 3H, Cp*H* overlapping with 2 C*H* of the chiral centers), 2.24 (s, 3H, CH_3 *p*-tolyl), 2.23 (s, 3H, CH_3 *p*-tolyl), 2.17 (s, 3H, CH_3 *o*-tolyl), 1.26 (d, *J* = 6.8 Hz, 3H, CH_3 chiral center), 0.93 (d, *J* = 6.7 Hz, 3H, CH_3 chiral center).

^{13}C{^{1}H} NMR (151 MHz, 298 K, DMSO-d_6): δ = 157.9, 152.6, 138.6, 138.5, 138.0, 137.0, 136.7, 135.92, 135.90, 134.1, 134.0, 132.0, 130.6, 130.5, 130.1, 129.8, 129.4, 129.1, 128.8, 128.7, 128.6, 128.54, 128.50 (3C), 127.8, 126.5, 125.8, 124.2, 123.2, 123.1, 121.7, 119.4, 117.5, 110.2, 103.5, 93.2 (d, *J* = 7.2 Hz), 91.4 (d, *J* = 7.2 Hz), 74.1, 74.0 (d, *J* = 6.0 Hz), 72.3, 20.7 (2C), 20.1, 18.4, 18.2.

HRMS (ESI) *m/z*: ([M-^{79}Br]$^+$) calcd for $C_{92}H_{74}O_4{}^{103}Rh_2{}^{79}Br_3$ 1685.1242, found 1685.1239 (Δ = -0.20 ppm).

IR (ATR): 2971 (w), 2957 (w), 2922 (w), 2861 (w), 1587 (w-m), 1516 (w), 1484 (w), 1459 (m), 1446 (m), 1415 (s), 1386 (m-w), 1371 (m-w), 1337 (w), 1318 (w), 1271 (w), 1254 (m-w), 1235 (m), 1226 (m), 1205 (w), 1183 (w), 1143 (m), 1102 (m), 1059 (s), 1033 (m), 1008 (m), 988 (w), 964 (w), 945 (w), 897 (w), 862 (m), 835 (w), 817 (m), 804 (w), 758 (vs), 742 (s), 726 (m), 683 (w-m), 657 (w), 623 (w), 592 (w), 563 (w), 531 (w), 521 (m), 791 (m), 456 (m), 433 (m) cm^{-1}.

Satisfactory **EA** was not obtained.

UV/VIS (THF): λ_{max} (log ε) = 272 (4.88), 528 (3.76) nm.

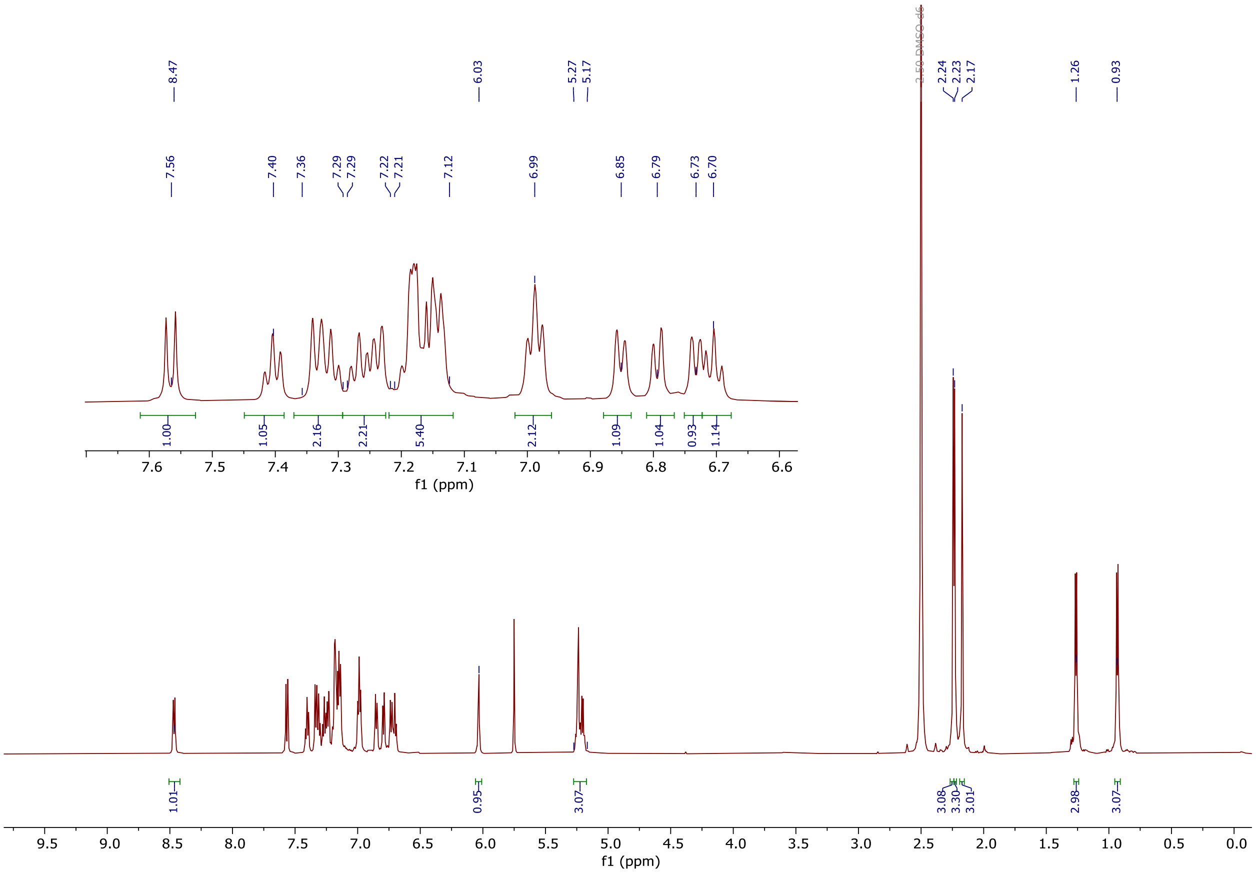

Figure S124: ^{1}H NMR of (-)-(*M,R,R,R$_p$*)-**25d** (600 MHz, 298 K, DMSO-d_6).

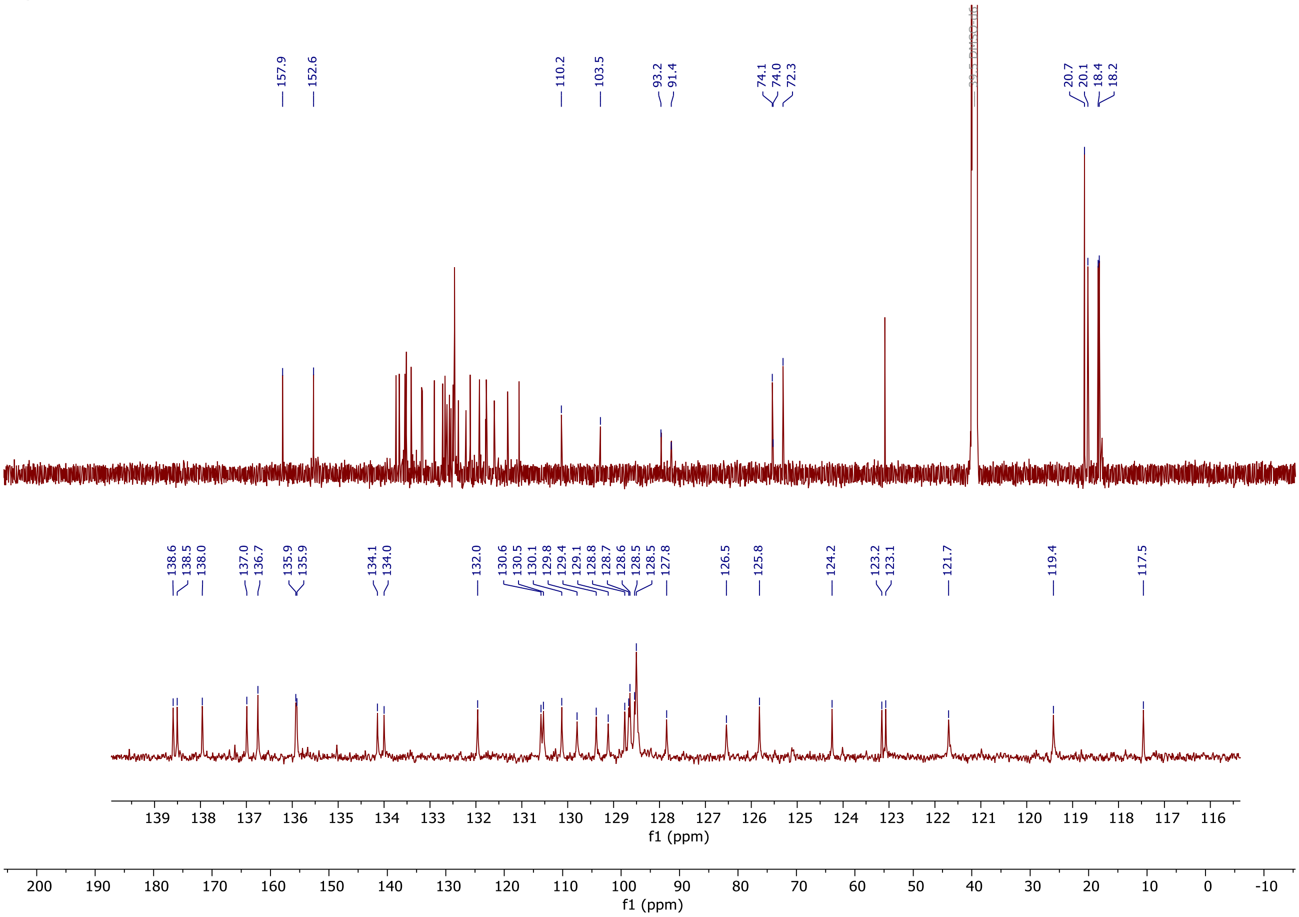

Figure S125: ^{13}C NMR of (-)-(*M,R,R,R$_p$*)-**25d** (151 MHz, 298 K, DMSO-d_6).

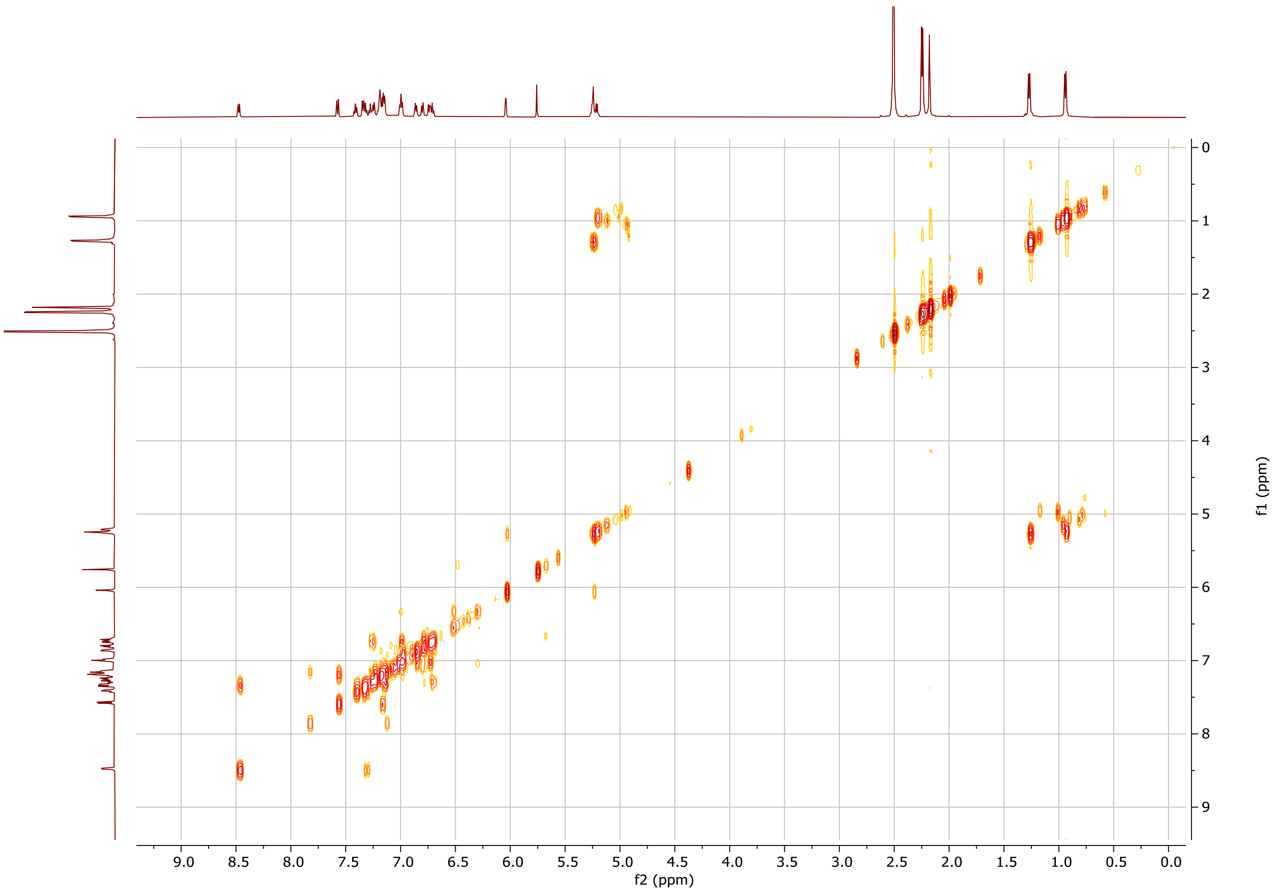

Figure S126: ^{1}H-^{1}H COSY of (-)-(*M*,*R*,*R*,*R*$_p$)-**25d** (600 MHz/600 MHz, 298 K, DMSO-*d*$_6$).

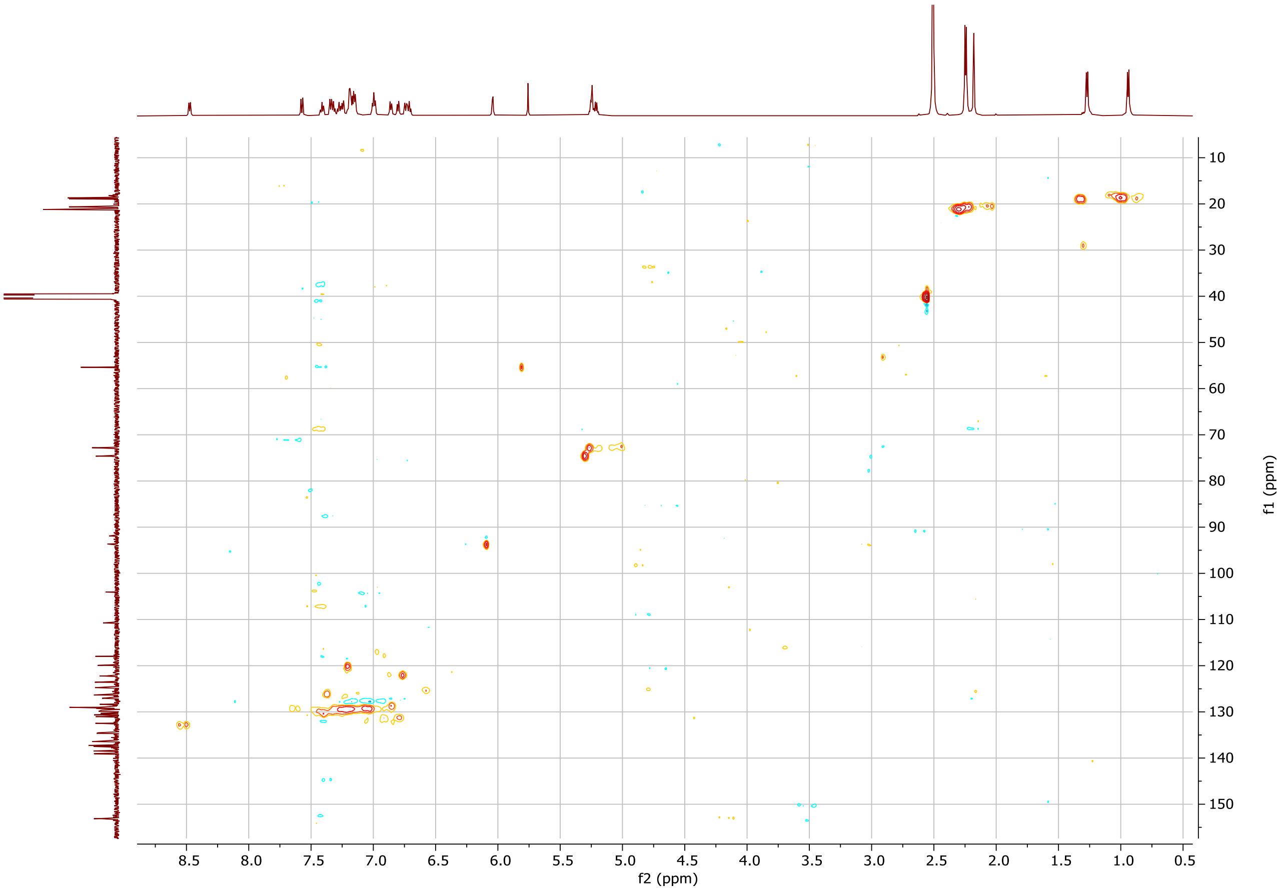

Figure S127: ^{1}H-^{13}C HSQC of (-)-(*M*,*R*,*R*,*R*$_p$)-**25d** (600 MHz/151 MHz, 298 K, DMSO-*d*$_6$).

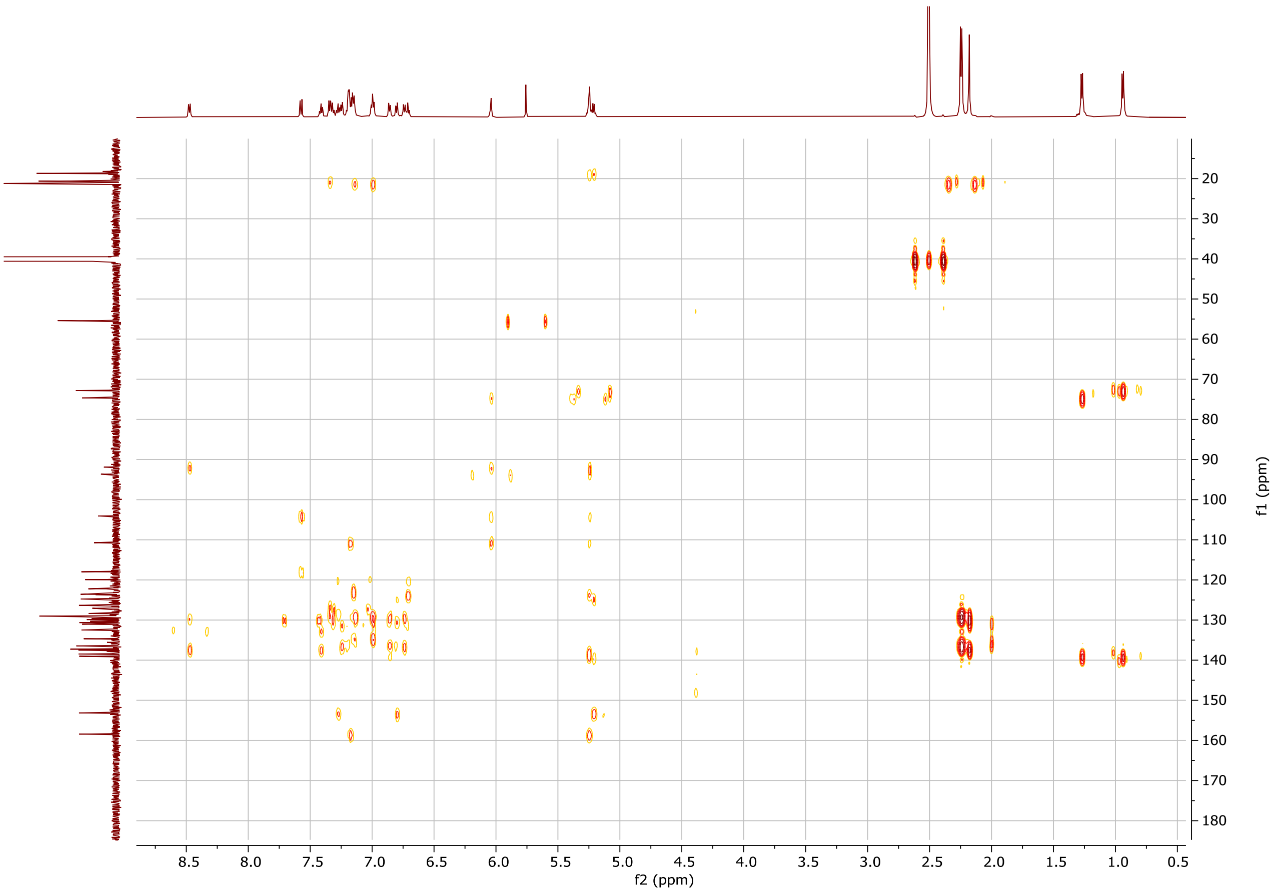

Figure S128: ^{1}H-^{13}C HMBC of (-)-(*M,R,R,R$_p$*)-**25d** (600 MHz/151 MHz, 298 K, DMSO-*d$_6$*).

Oxa[7]helicene complexes of Rh(I) and Rh(III) (-)-(M,R,R,R_p)-**23a,b** and (-)-(M,R,R,R_p)-**26a,b**

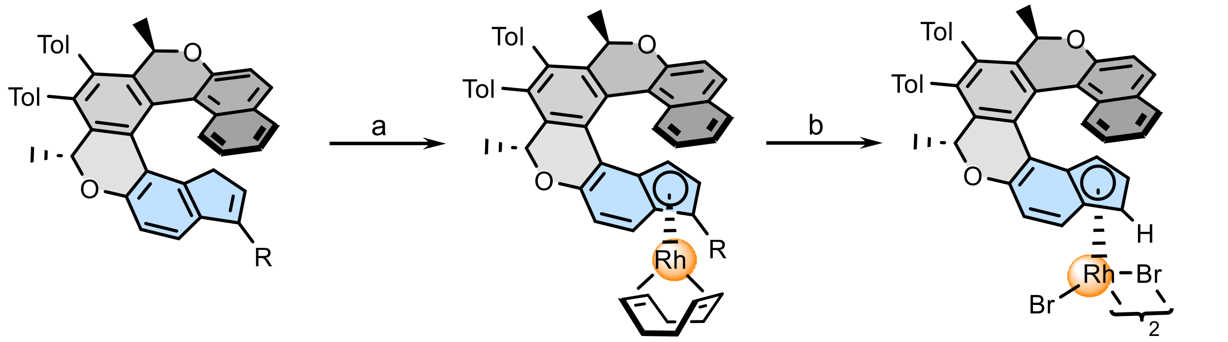

(-)-(M,R,R)-**20a-b**

(-)-(M,R,R,R_p)-**23a** R = H, 79%
(-)-(M,R,R,R_p)-**23b** R = Me, 74%

(-)-(M,R,R,R_p)-**26a**R = H, 76%
(-)-(M,R,R,R_p)-**26b**R = Me, 73%

(a) $[Rh(cod)Cl]_2$ (0.55 equiv.), *t*-BuOK (1.3 equiv.), THF, rt, 2-4 h; (b) Br_2 (2.5 equiv.), pentane, 0 °C, 14–18 h.

Compound (-)-(M,R,R,R_p)-**23a**

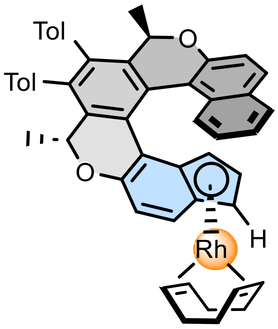

Prepared according to **GP2** from substituted oxa[7]helicene (-)-(M,R,R)-**20a** (40 mg, 0.07 mmol, 1.0 equiv.), $[Rh(cod)Cl]_2$ (20.1 mg, 0.04 mmol, 0.6 equiv.), and potassium *tert*-butoxide (9.9 mg, 0.09 mmol, 1.3 equiv.) providing (-)-(M,R,R,R_p)-**23a** (42.3 mg, 79%) as a yellow amorphous solid.

R_f = 0.68 (pentane:toluene 1:1).

$[\alpha]^{20}_D$ -1628.9 (*c* 0.148, THF).

^{1}H NMR (500 MHz, 298 K, CD_2Cl_2): δ = 7.68 (d, *J* = 8.7 Hz, 1H, CH^{Ar}), 7.50 (d, *J* = 8.1 Hz, 1H, CH^{Ar}), 7.26 (m, 2H, CH^{Ar}), 7.22 (m, 2H, *o*-protons of *p*-tolyl), 7.14(m, 2H, *m*-protons of *p*-tolyl), 7.01 – 6.93 (m, 3H, *m*-protons of *p*-tolyl overlapping with CH^{Ar}), 6.91 – 6.83 (m, 3H, *o*-protons of *p*-tolyl overlapping with CH^{Ar}), 6.70 (dd, *J* = 8.4, 6.7, 1H, CH^{Ar}), 5.29 – 5.21 (m, 2H, C*H* chiral center), 5.17 (dd, *J* = 2.5 Hz, 2.4 Hz, 1H, Cp*H*), 4.55 (m, 1H, Cp*H*), 3.87 (m, 1H, Cp*H*), 3.74 – 3.64 (m, 4H. C*H* COD), 2.30 (s, 3H, CH_3 tolyl), 2.29 (s, 3H, CH_3 tolyl), 1.86 – 1.72 (m, 4H, CH_2 COD), 1.61 (ddq, *J* = 13.7, 8.7, 4.3 Hz, 2H, CH_2 COD), 1.37 (ddt, *J* = 14.0, 8.3, 4.3 Hz, 2H, CH_2 COD), 1.16 (d, *J* = 6.6 Hz, 3H, CH_3 chiral center), 1.02 (d, *J* = 6.6 Hz, 3H, CH_3 chiral center).

^{13}C{^{1}H} NMR (126 MHz, 298 K, CD_2Cl_2): δ = 152.5, 150.9, 140.1, 139.7, 136.74, 136.72 (2C), 136.5, 136.14, 136.05, 131.54, 131.53, 130.8, 130.3, 130.0, 129.94, 129.93, 128.9, 129.0, 128.92, 128.90, 127.8, 127.0, 124.7, 124.3, 124.2, 123.5, 121.0, 120.5, 119.9, 116.9, 115.7, 113.2 (d, *J* = 1.8 Hz), 108.5 (d, *J* = 2.0 Hz), 91.4 (d, *J* = 5.1 Hz), 75.9 (d, *J* = 4.1 Hz), 75.2 (d, *J* = 4.1 Hz), 74.0, 73.6, 68.8 (d, *J* = 13.3 Hz, 2C), 65.9 (d, *J* = 13.8 Hz, 2C), 33.5 (2C), 30.3 (2C), 21.5 (2C), 19.1, 18.3.

HRMS (ESI) *m/z*: ($[M]^+$) calcd for $C_{51}H_{45}O_2{}^{103}Rh$ 792.2469, found 792.2462 (Δ = -0.96 ppm).

IR (ATR): 3049 (vw), 3021 (w), 2973 (m), 2924 (m), 2867 (m), 2822 (m), 1618 (w), 1589 (m), 1549 (w), 1514 (m), 1495 (w), 1462 (w), 1421 (m), 1377 (m), 1363 (m), 1315 (m), 1251 (m), 1229 (s), 1202 (s), 1182 (w), 1150 (s), 1102 (w), 1070 (s), 1055 (vs), 1020 (m), 1001 (m), 981 (m), 955 (m), 915 (w), 883 (w), 857 (m), 838 (m), 804 (vs), 769 (w), 747 (s), 728 (vs), 714 (w), 694 (m), 680 (w), 660 (w), 636 (w), 627 (w), 604 (w), 523 (m), 481 (m), 459 (s), 428 (m) cm^{-1}.

EA (performed on a batch containing 0.5 equiv. of toluene): Calcd for $(C_{51}H_{45}O_2Rh)(C_7H_8)_{0.5}$: C, 78.03%; H, 5.89%. Found: C, 78.02%; H, 6.13%.

UV/VIS (THF): λ_{max} (log ε) = 240 (4.70), 364 (4.32) nm.

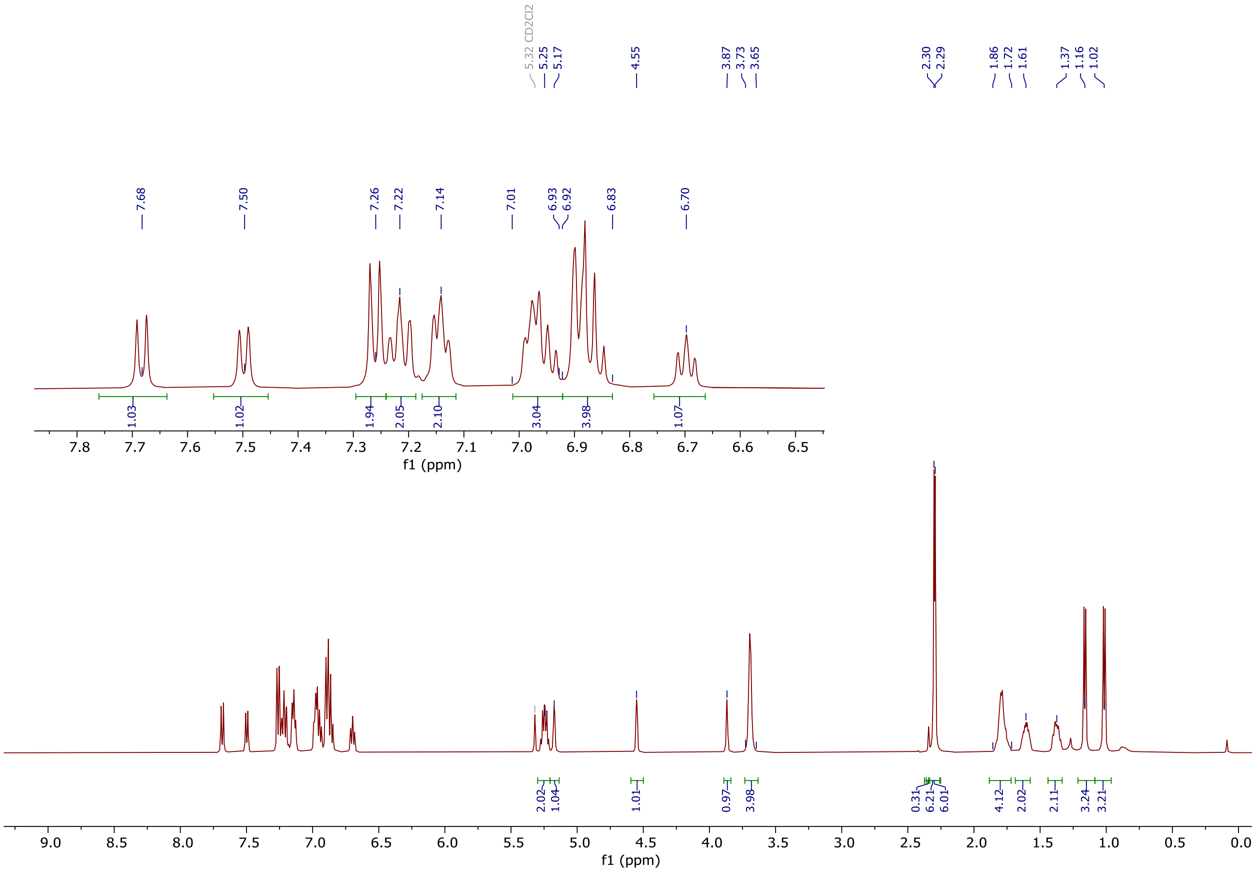

Figure S129: ^{1}H NMR of (-)-(*M,R,R,R$_p$*)-**23a** (500 MHz, 298 K, CD_2Cl_2).

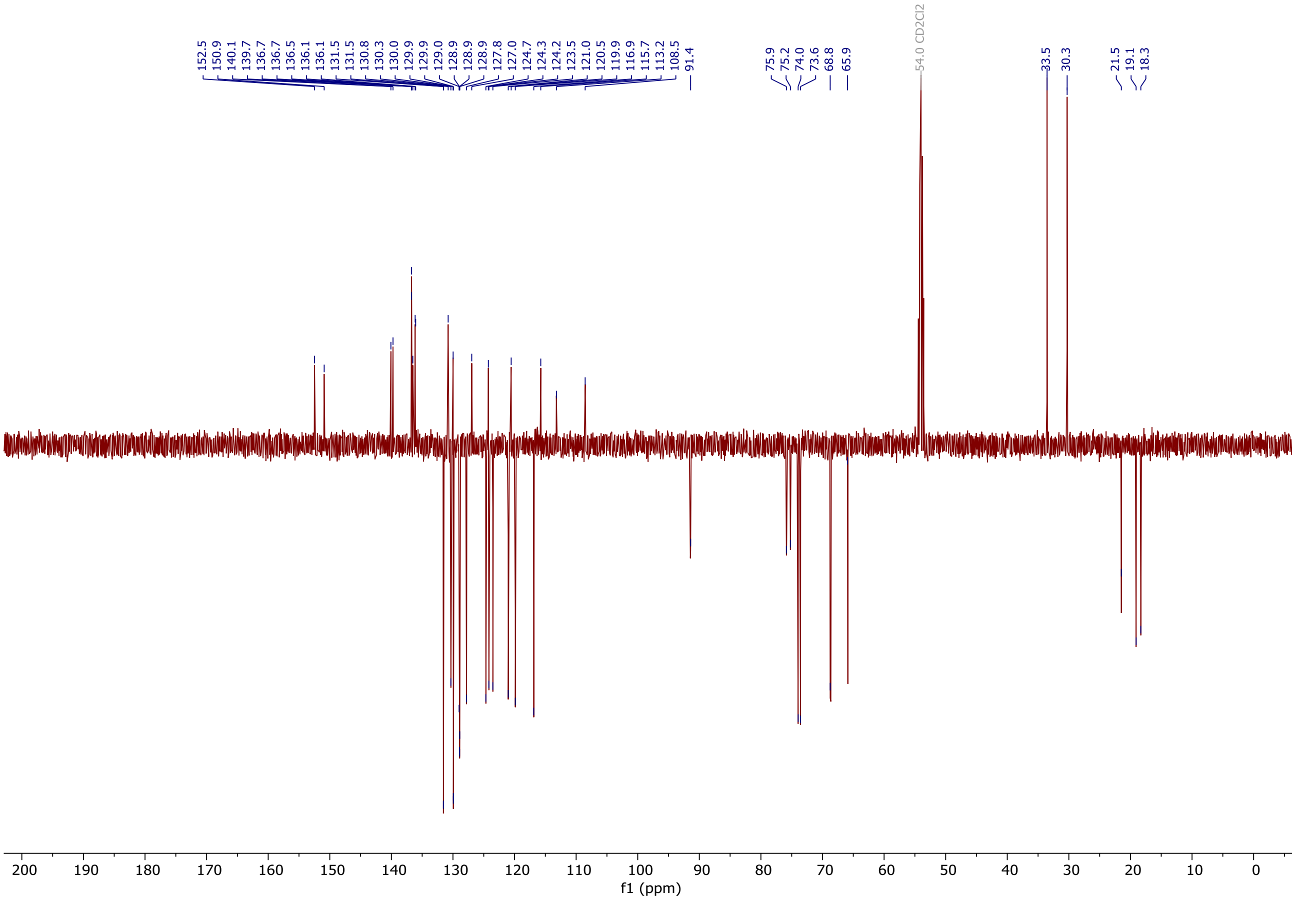

Figure S130: ^{13}C APT NMR of (-)-(*M,R,R,R$_p$*)-**23a** (126 MHz, 298 K, CD_2Cl_2).

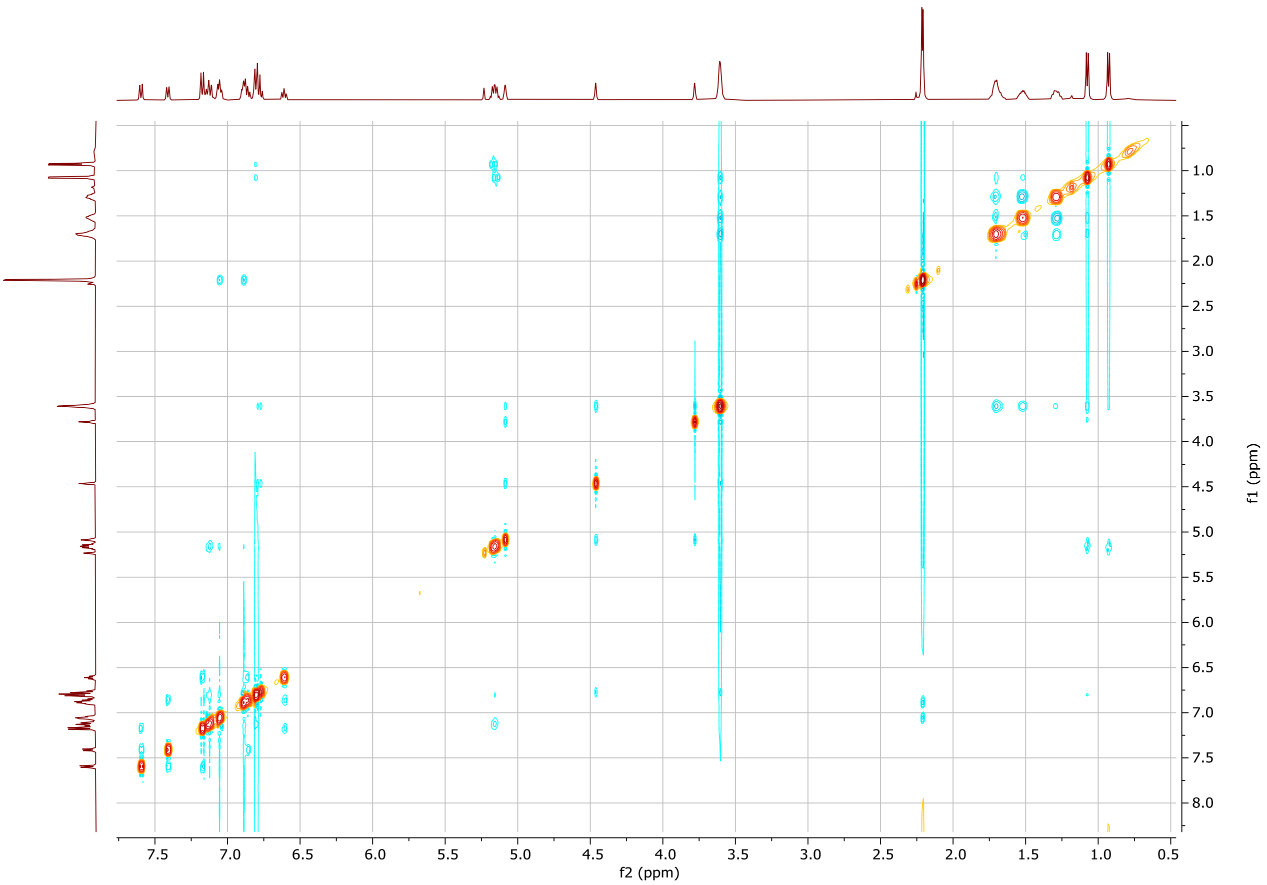

Figure S131: ^{1}H-^{1}H NOESY of (-)-(*M*,*R*,*R*,*R*$_p$)-**23a** (600 MHz/600 MHz, 298 K, CD_2Cl_2).

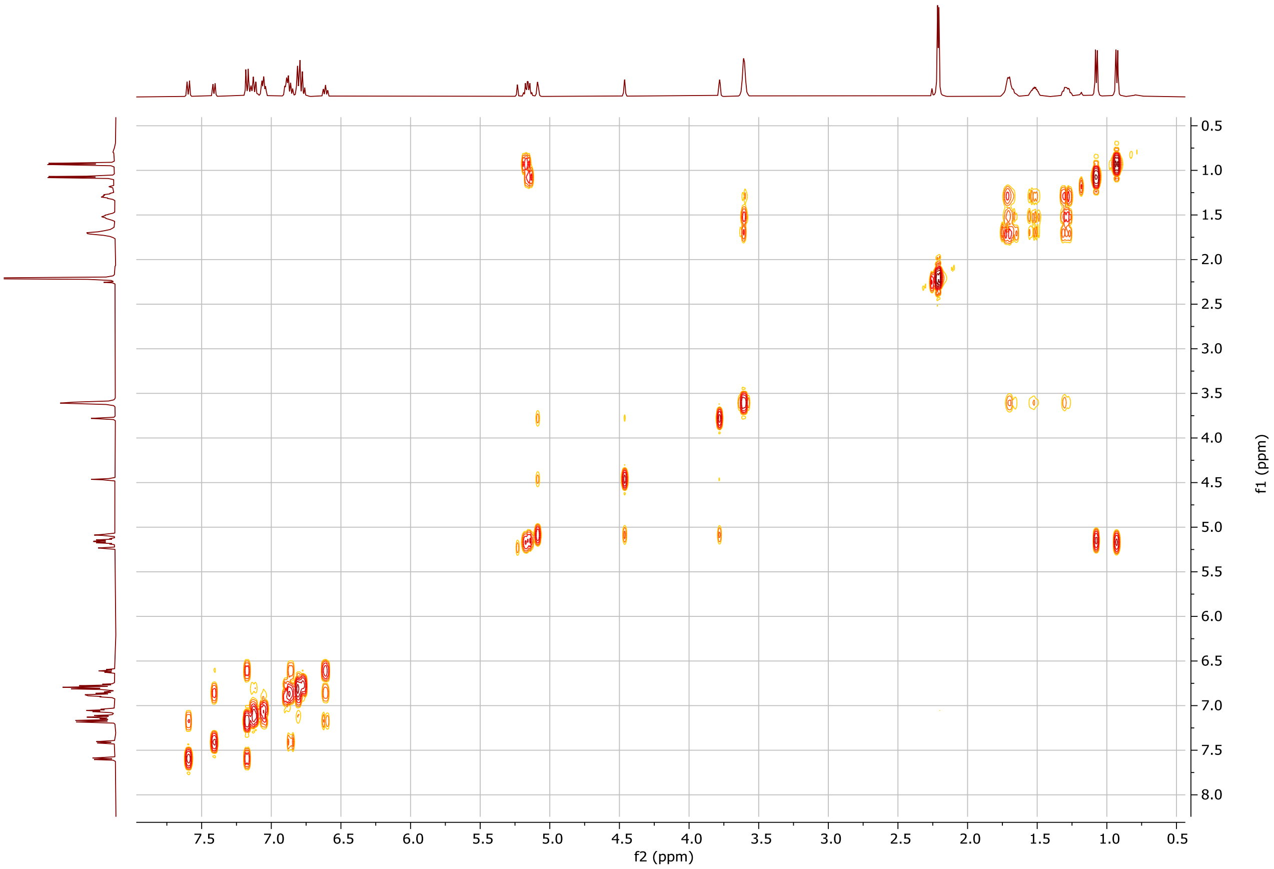

Figure S132: ^{1}H-^{1}H COSY of (-)-(*M*,*R*,*R*,*R*$_p$)-**23a** (600 MHz/600 MHz, 298 K, CD_2Cl_2).

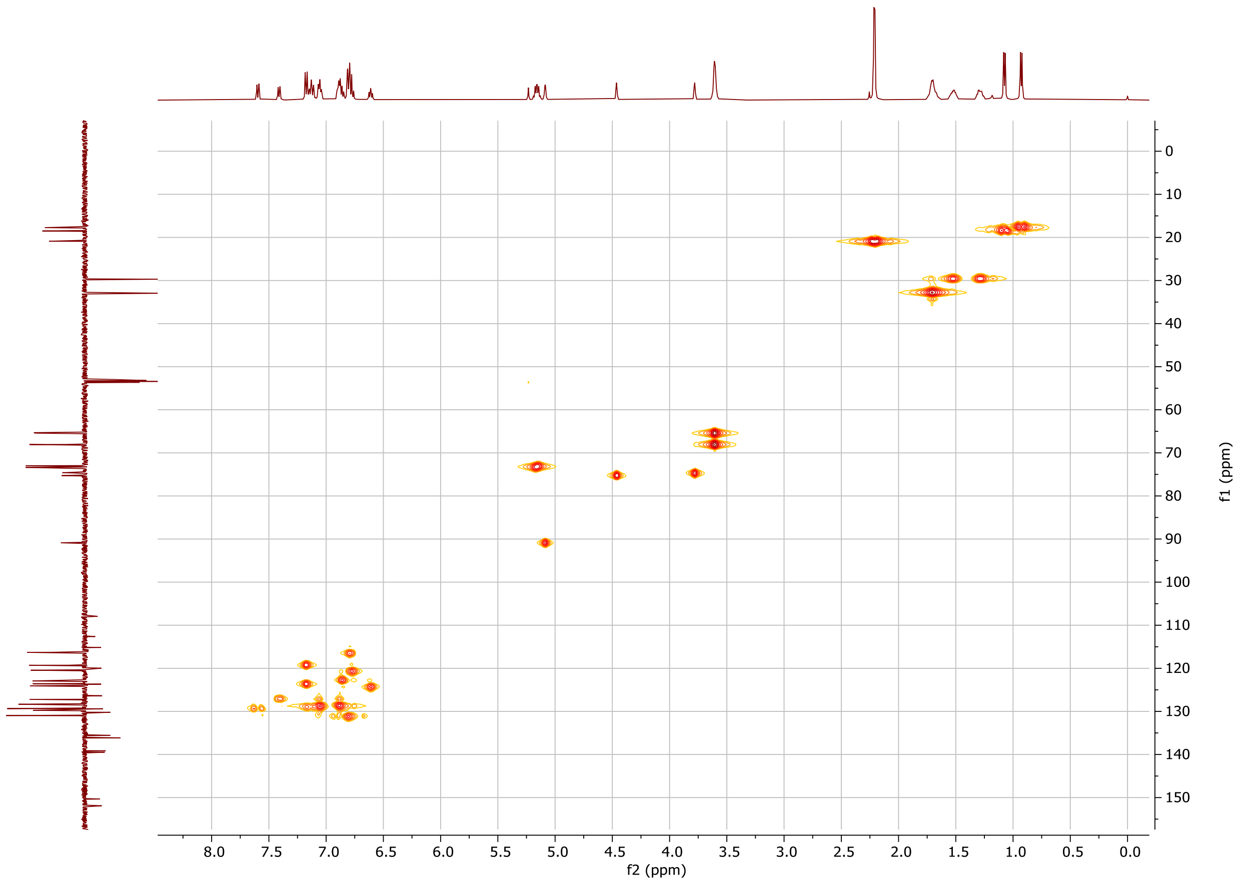

Figure S133: ^{1}H-^{13}C HSQC of (-)-(*M,R,R,R$_p$*)-**23a** (600 MHz/151 MHz, 298 K, CD_2Cl_2).

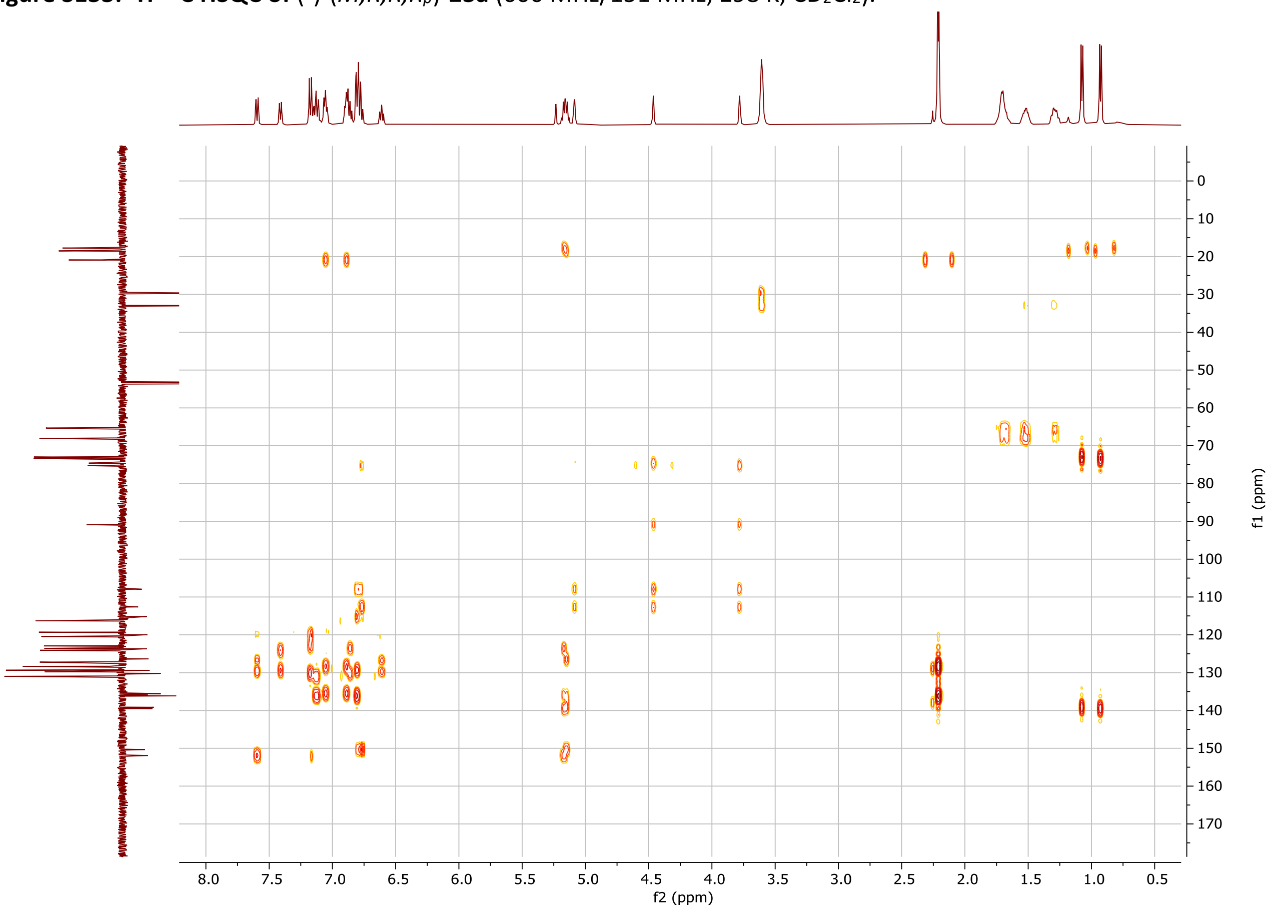

Figure S134: ^{1}H-^{13}C HMBC of (-)-(*M,R,R,R$_p$*)-**23a** (600 MHz/151 MHz, 298 K, CD_2Cl_2).

Compound (-)-(*M,R,R,R$_p$*)-**23b**

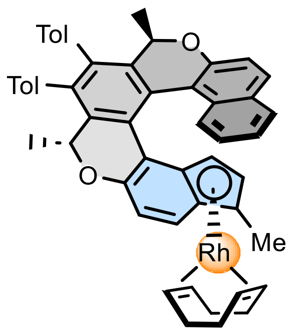

Prepared according to **GP2** from substituted oxa[7]helicene (-)-(*M,R,R*)-**20b** (40 mg, 0.07 mmol, 1.0 equiv.), $[Rh(cod)Cl]_2$ (18 mg, 0.04 mmol, 0.55 equiv.), and potassium *tert*-butoxide (9.6 mg, 0.09 mmol, 1.3 equiv.) providing (-)-(*M,R,R,R$_p$*)-**23b** (39.5 mg, 74%) as a yellow amorphous solid.

R$_f$ = 0.66 (pentane:toluene 1:1).

$[\alpha]^{20}_D$ -1676.6 (*c* 0.108, THF).

^{1}H NMR (600 MHz, 298 K, CD_2Cl_2): δ = 7.68 (d, *J* = 8.7 Hz, 1H, CH^{Ar}), 7.49 (d, *J* = 8.1 Hz, 1H, CH^{Ar}), 7.28 – 7.18 (m, 4H, 2 *o*-protons of *p*-tolyl overlapping 2 CH^{Ar}), 7.14 (m, 2H, *m*-protons of *p*-tolyl), 7.01 – 6.87 (m, 6H, 2 *m*-protons and 2 *o*-protons of *p*-tolyl overlapping 2 CH^{Ar}), 6.83 (d, *J* = 8.5 Hz, 1H, CH^{Ar}), 6.68 (dd, *J* = 8.6, 6.7 Hz, 1H, CH^{Ar}), 5.28 – 5.21 (m, 2H, C*H* chiral center), 4.99 (t, *J* = 2.2 Hz, 1H, Cp*H*), 3.71 (d, *J* = 2.8 Hz, 1H, Cp*H*), 3.54 (m, 2H, C*H* COD), 3.24 (tt, *J* = 7.8, 3.4 Hz, 2H, C*H* COD), 2.30 (s, 3H, CH_3 tolyl), 2.29 (s, 3H, CH_3 tolyl), 1.89 – 1.75 (m, 4H, CH_2 COD), 1.62 (dtd, *J* = 18.0, 8.8, 3.8 Hz, 2H, CH_2 COD), 1.53 (s, 3H, CpCH_3), 1.38 (tt, *J* = 13.0, 6.1 Hz, 2H, CH_2 COD), 1.16 (d, *J* = 6.7 Hz, 3H, CH_3 chiral center), 1.03 (d, *J* = 6.7 Hz, 3H, CH_3 chiral center).

^{13}C{^{1}H} NMR (151 MHz, 298 K, CD_2Cl_2): δ = 152.5, 151.0, 140.0, 139.7, 136.74, 136.73, 136.71, 136.5, 136.2, 136.1, 131.58, 131.57, 130.8, 130.3, 129.98, 129.96, 129.01, 128.97, 128.93, 128.91, 127.8, 127.1, 124.6, 124.3, 124.1, 123.4, 120.6, 119.8, 119.2, 116.4, 115.6, 112.9, 108.5 (d, *J* = 2.1 Hz), 92.3 (d, *J* = 5.2 Hz), 87.7 (d, *J* = 4.0 Hz), 73.9, 73.6, 72.5 (d, *J* = 4.4 Hz), 69.0 (d, *J* = 13.8 Hz, 2C), 68.8 (d, *J* = 13.3 Hz, 2C), 33.8 (2C), 30.0 (2C), 21.5 (2C), 19.0, 18.4, 10.2.

HRMS (ESI) *m/z*: ($[M]^+$) calcd for $C_{52}H_{47}O_2{}^{103}Rh$ 806.2626, found 806.2624 (Δ = -0.27 ppm).

IR (ATR): 2973 (w), 2956 (w), 2920 (m), 2865 (m), 2822 (w-m), 1618 (w), 1589 (m), 1549 (w), 1415 (m), 1442 (w-m), 1429 (w-m), 1412 (w-m), 1377 (m), 1362 (m), 1317 (m), 1259 (w), 1230 (m), 1213 (s), 1182 (w), 1150 (m), 1121 (w), 1109 (w), 1096 (w), 1075 (m), 1060 (s), 1020 (w), 1002 (m), 982 (w), 955 (w), 904 (w), 857 (m), 837 (m), 804 (vs), 771 (w), 745 (vs), 729 (w), 718 (w), 695 (w), 680 (w), 660 (w), 635 (w), 605 (w), 527 (m), 480 (s), 459 (m), 451 (m), 429 (m) cm^{-1}.

EA: Calcd for $C_{52}H_{47}O_2Rh$: C, 77.41%; H, 5.87%. Found: C, 77.47%; H, 6.06%.

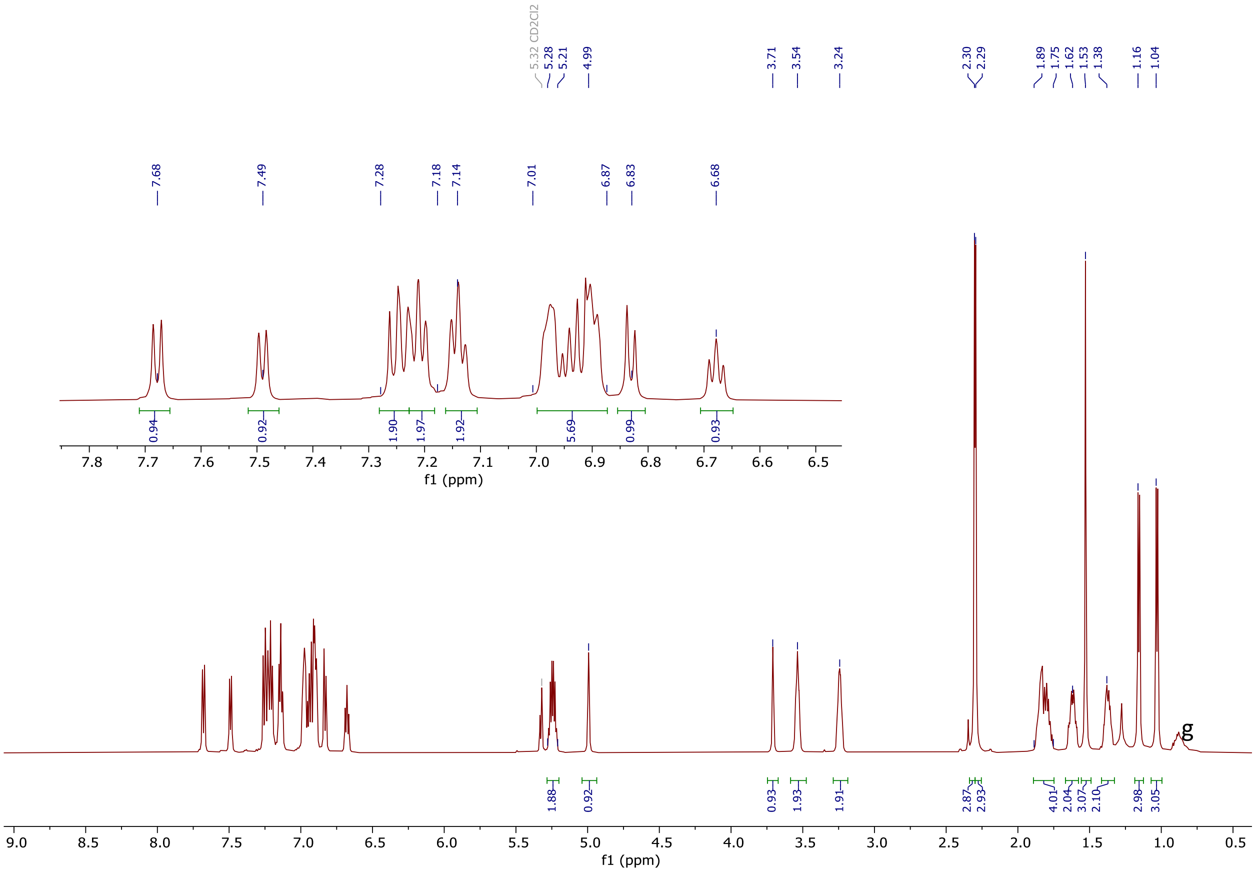

Figure S135: ^{1}H NMR of (-)-(*M,R,R,R$_p$*)-**23b** (600 MHz, 298 K, CD_2Cl_2) (g = grease).

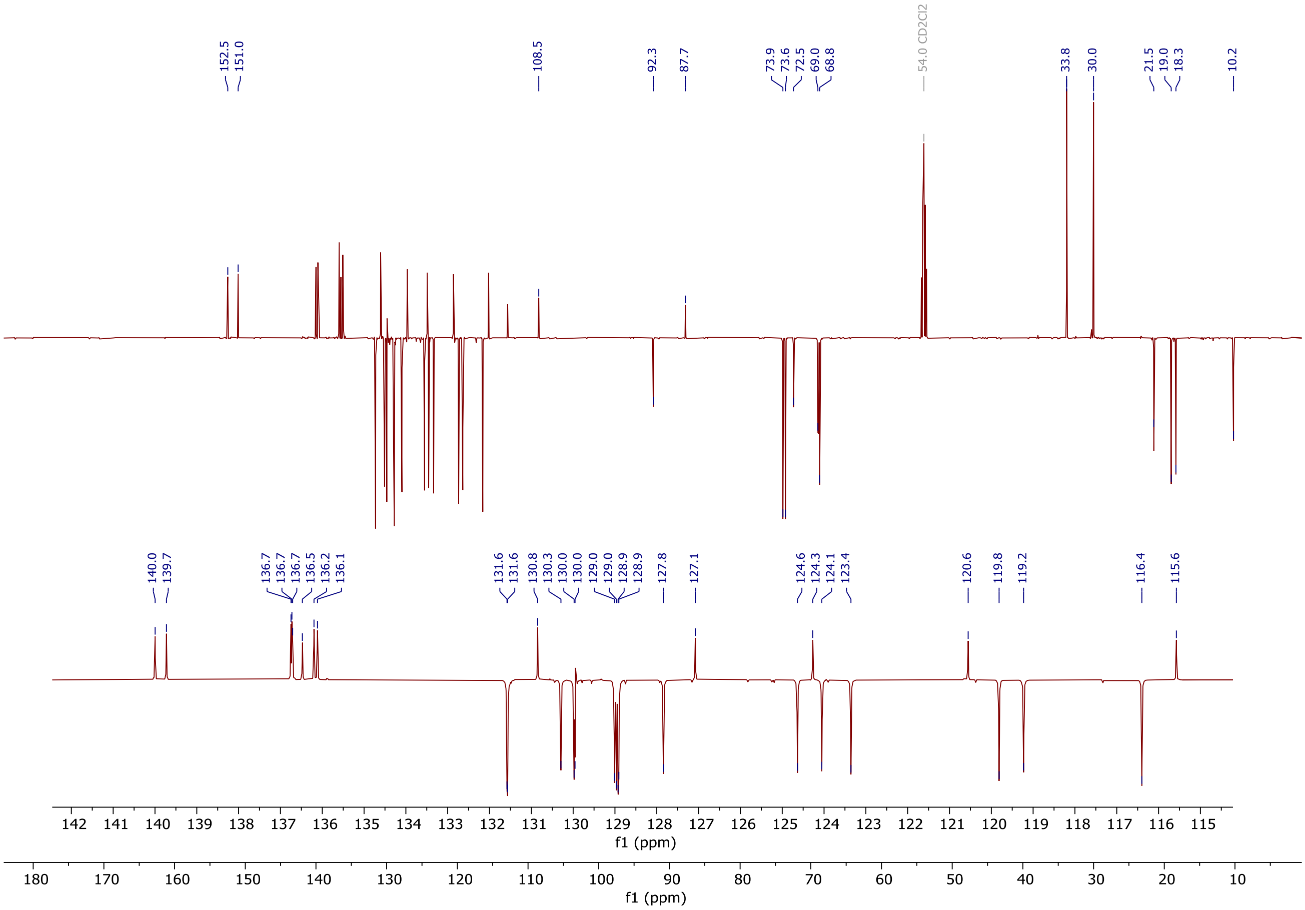

Figure S136: ^{13}C APT NMR of (-)-(*M,R,R,R$_p$*)-**23b** (151 MHz, 298 K, CD_2Cl_2).

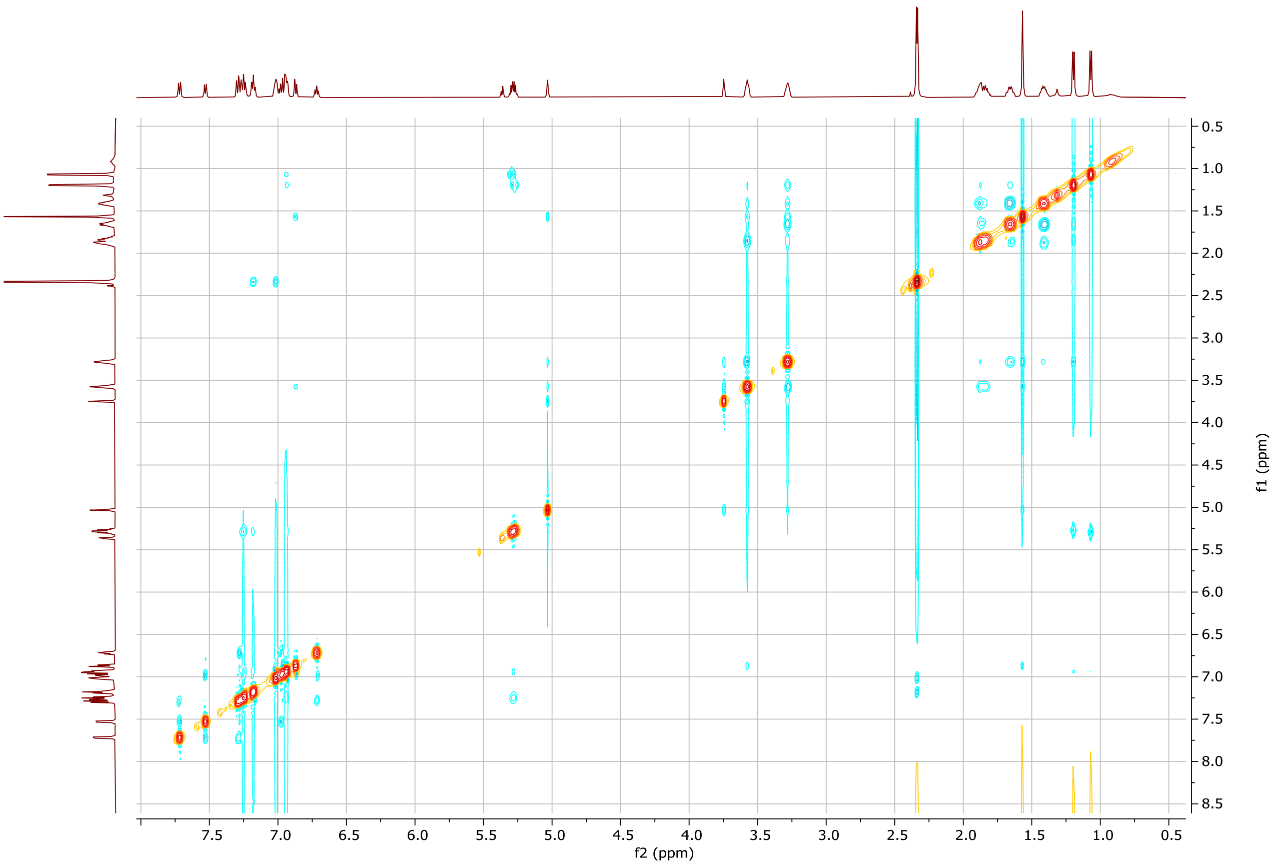

Figure S137: ^{1}H-^{1}H NOESY of (-)-(*M,R,R,R$_p$*)-**23b** (600 MHz/600 MHz, 298 K, CD_2Cl_2).

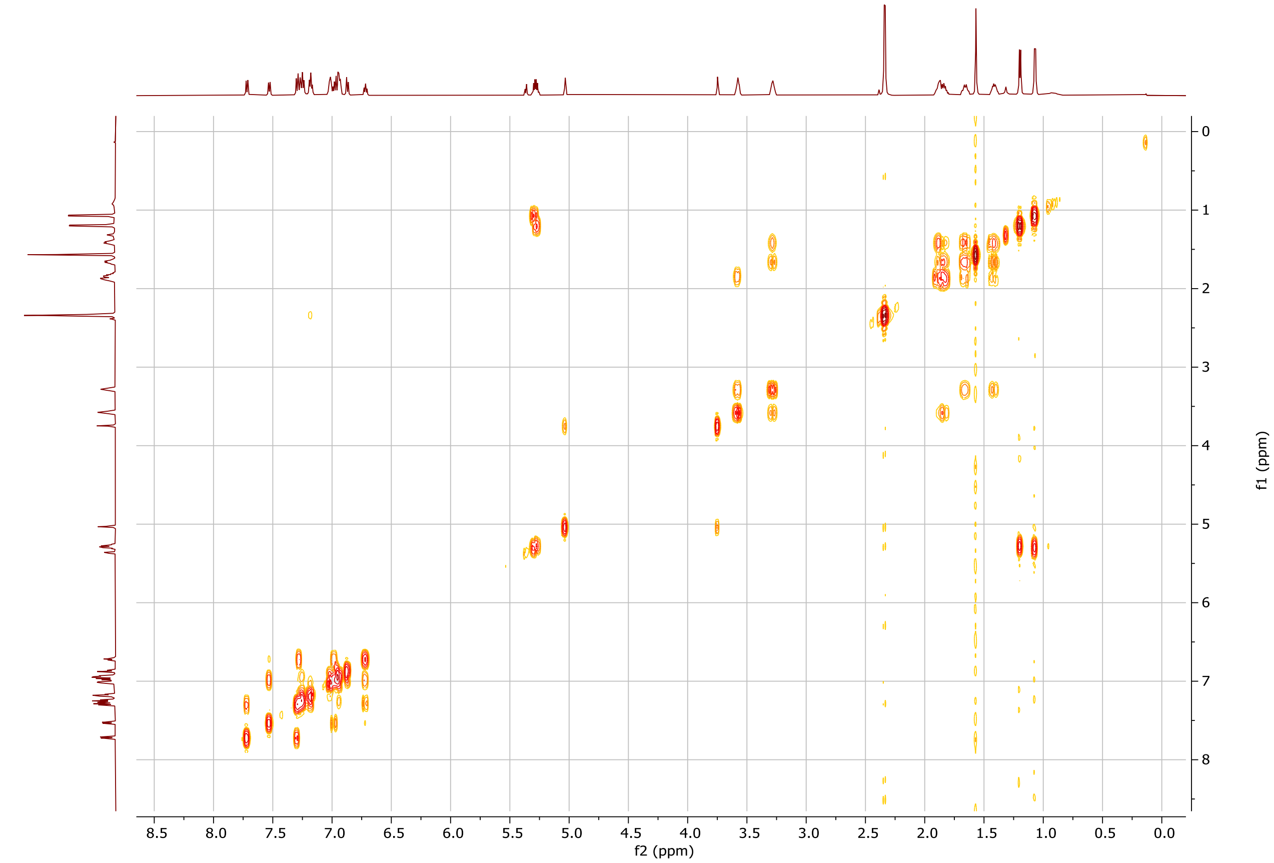

Figure S138: ^{1}H-^{1}H COSY of (-)-(*M,R,R,R$_p$*)-**23b** (600 MHz/600 MHz, 298 K, CD_2Cl_2).

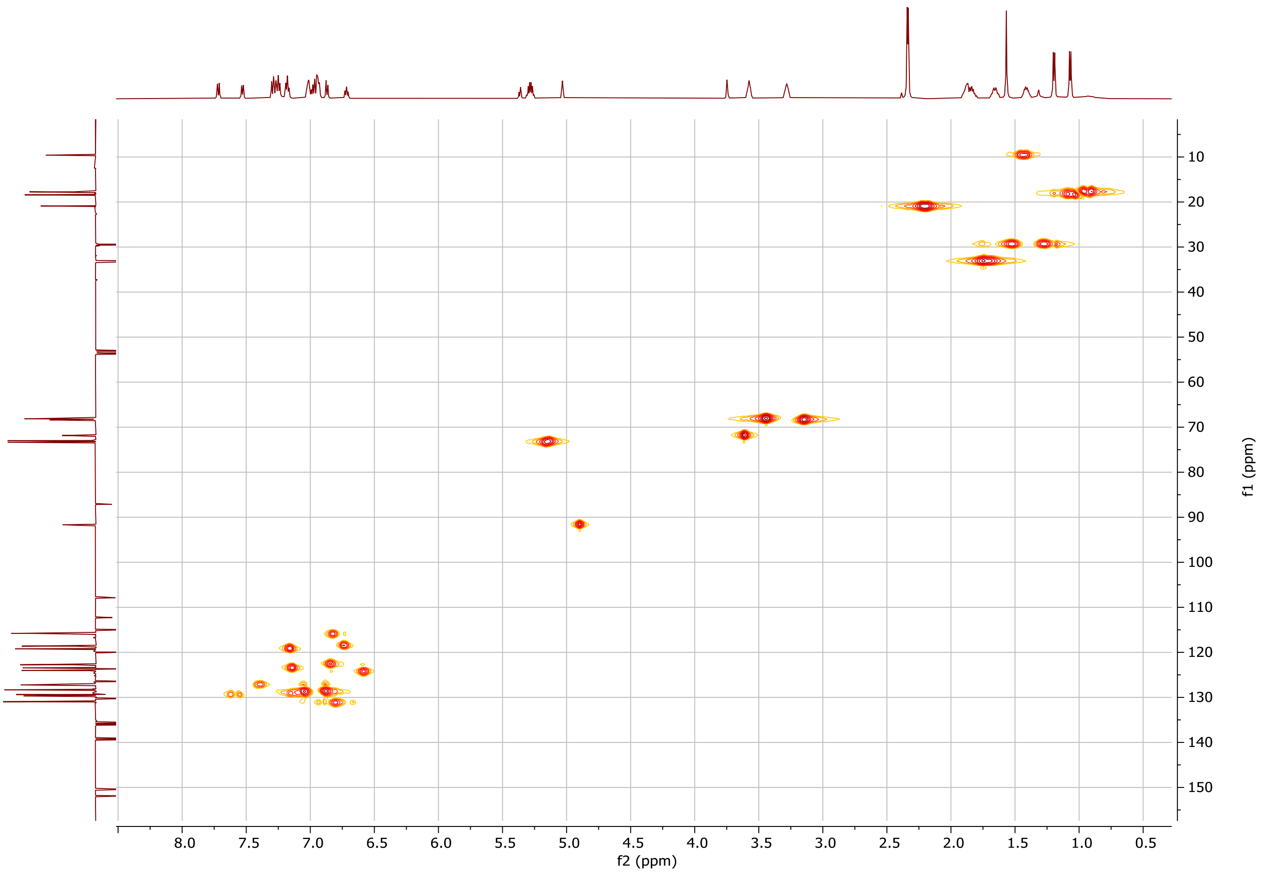

Figure S139: ^{1}H-^{13}C HSQC of (-)-(*M,R,R,R$_p$*)-**23b** (600 MHz/151 MHz, 298 K, CD_2Cl_2).

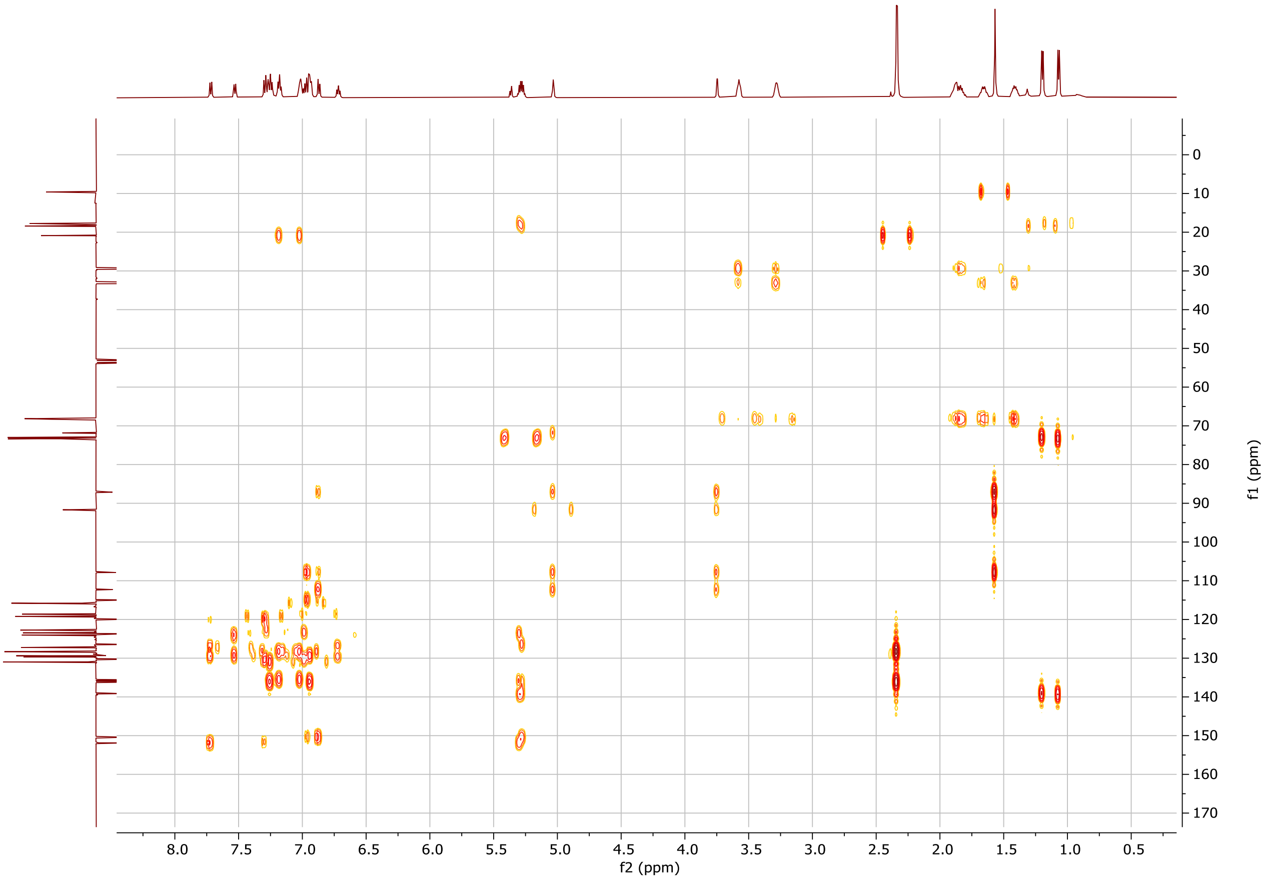

Figure S140: ^{1}H-^{13}C HMBC of (-)-(*M,R,R,R$_p$*)-**23b** (600 MHz/151 MHz, 298 K, CD_2Cl_2).

Compound (-)-(*M,R,R,R_p*)-**26a**

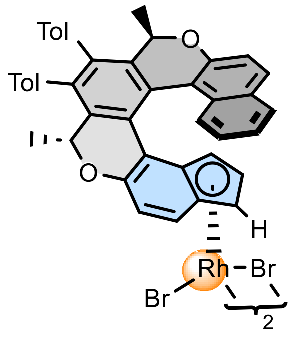

Prepared according to **GP4** starting from (-)-(*M,R,R,R_p*)-**23a** (33 mg, 0.04 mmol, 1.0 equiv.) and bromine (5.3 µL, 16.6 mg, 0.1 mmol, 2.5 equiv.) to give (-)-(*M,R,R,R_p*)-**26a** (26.8 mg, 76 %) as a dark red amorphous solid.

$[\alpha]^{20}_D$ -2690 (*c* 0.010, THF).

^{1}H NMR (600 MHz, 298 K, DMSO-*d_6*): δ = 7.91 (d, *J* = 8.7 Hz, 1H, CH^{Ar}), 7.71 (d, *J* = 8.2 Hz, 1H, CH^{Ar}), 7.47 (m, 1H, CH^{Ar}), 7.43 (m, 2H, CH^{Ar}), 7.32 (m, 1H, *o*-proton of *p*-tolyl), 7.25 (m, 1H, *o*-proton of *p*-tolyl), 7.16 (m, 2H, *m*-protons of *p*-tolyl), 7.09 (t, *J* = 7.5 Hz, 1H, CH^{Ar}), 7.03 (m, 1H, *m*-proton of *p*-tolyl), 7.00 (m, 2H, *m*-proton of *p*-tolyl overlapping with CH^{Ar}), 6.92 (m, 1H, *o*-proton of *p*-tolyl), 6.88 (m, 1H, *o*-proton of *p*-tolyl), 6.80 (t, *J* = 7.7 Hz, 1H, CH^{Ar}), 5.81 (s, 1H, Cp*H*), 5.34 – 5.25 (m, 2H, C*H* chiral center), 5.24 (d, *J* = 2.8 Hz, 1H, Cp*H*), 4.41 (s, 1H, Cp*H*), 2.25 (s, 6H, CH_3 tolyl), 1.42 (d, *J* = 6.8 Hz, 3H, CH_3 chiral center), 0.98 (d, *J* = 6.7 Hz, 3H, CH_3 chiral center).

^{13}C{^{1}H} NMR (151 MHz, 298 K, DMSO-*d_6*): δ = 158.2, 152.5, 139.6, 137.8, 137.7, 136.8, 136.11, 136.09, 134.3, 134.1, 131.1, 130.9, 130.6, 129.9, 128.91, 128.86, 128.74, 128.71, 128.68, 128.66, 128.4, 128.2, 127.6, 125.5, 123.7, 123.4, 123.1, 122.5, 119.8, 117.8, 117.7, 116.7, 98.4, 92.2 (d, *J* = 7.1 Hz), 75.2 (d, *J* = 6.3 Hz), 73.9, 72.9, 70.6 (d, *J* = 5.9 Hz), 20.9 (2C), 17.8, 17.6.

HRMS (ESI) *m/z*: ([M-^{79}Br]$^+$) calcd for $C_{86}H_{66}O_4{}^{103}Rh_2{}^{79}Br_3$ 1605.0616, found 1605.0604 (Δ = -0.72 ppm).

IR (ATR): 3048 (w), 3018 (w), 2971 (w), 2922 (w), 2863 (w), 1614 (w), 1587 (s), 1540 (w), 1513 (m), 1424 (s), 1377 (s), 1258 (s), 1243 (m), 1229 (s), 1208 (s), 1183 (w), 1140 (m), 1100 (w), 1073 (m), 1053 (s), 1034 (m), 1020 (m), 1004 (m), 984 (w), 957 (w), 914 (w), 866 (w), 837 (m), 808 (vs), 767 (w), 750 (s), 739 (m), 678 (w), 660 (w), 602 (w), 522 (m), 488 (m), 460 (m), 426 (m) cm^{-1}.

Satisfactory **EA** was not obtained.

UV/VIS (THF): λ_{max} (log ε) = 299 (4.71), 348 (4.55) nm.

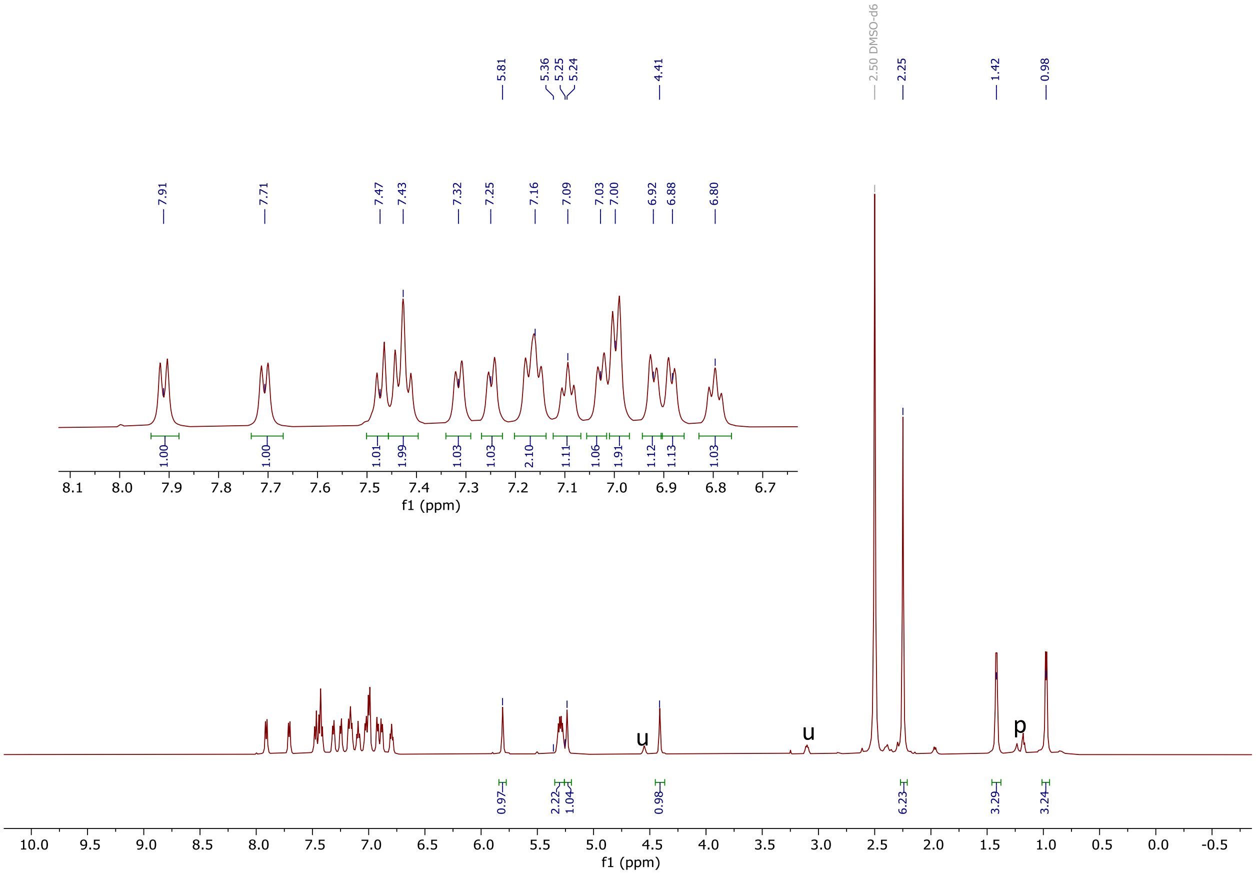

Figure S141: ^{1}H NMR of (-)-(*M,R,R,R$_p$*)-**26a** (600 MHz, 298 K, DMSO-*d$_6$*) (u = unidentified signals, p = pentane).

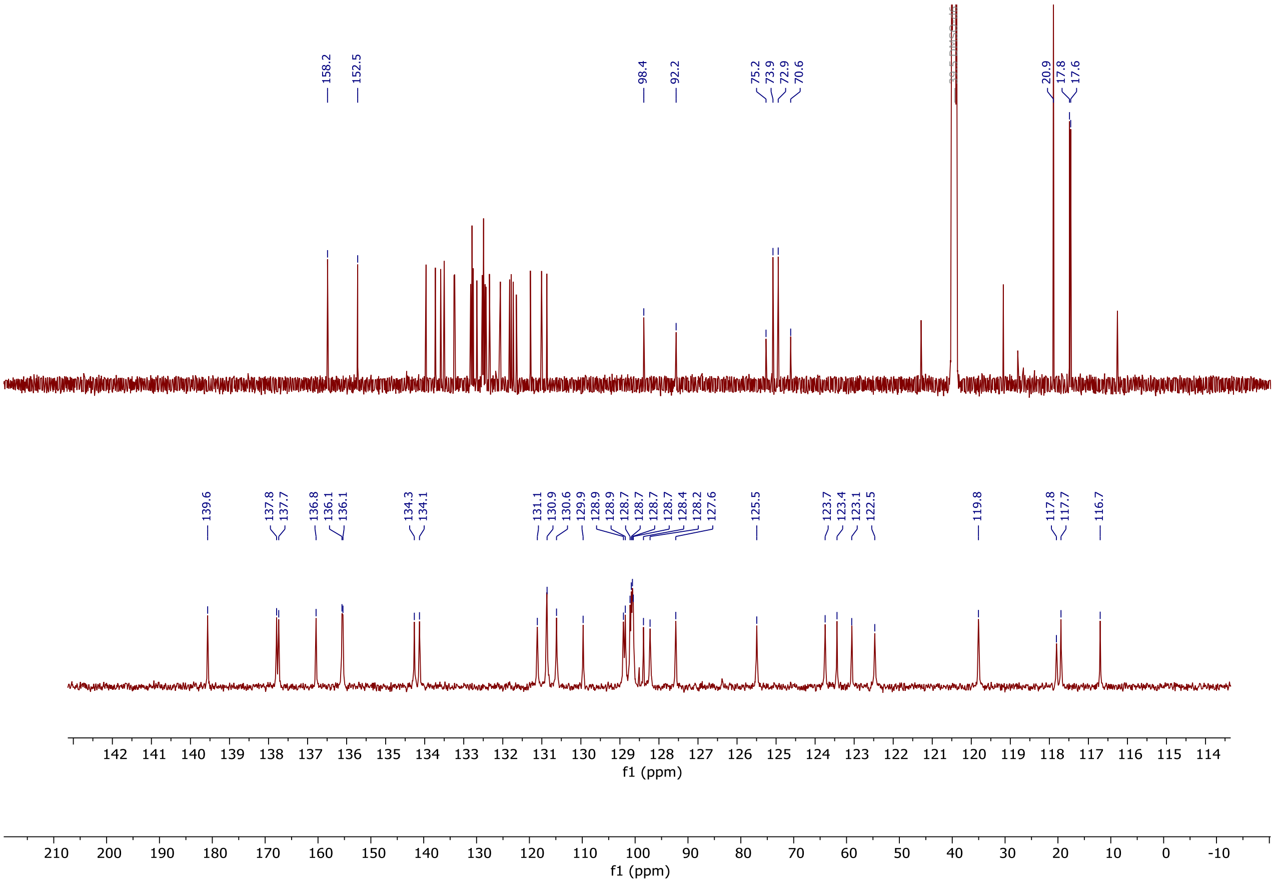

Figure S142: ^{13}C NMR of (-)-(*M,R,R,R$_p$*)-**26a** (151 MHz, 298 K, DMSO-*d$_6$*).

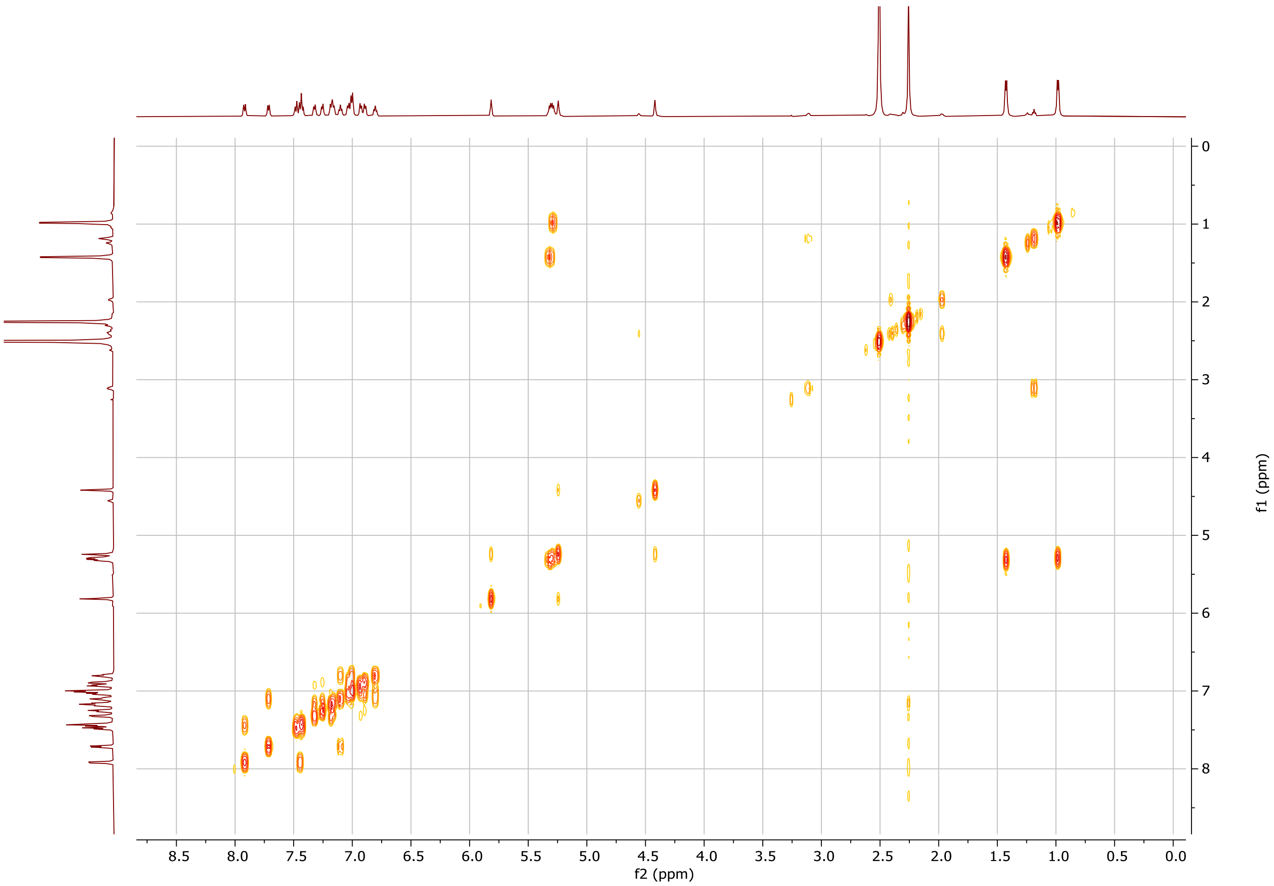

Figure S143: ^{1}H-^{1}H COSY of (-)-(*M*,*R*,*R*,*R*$_p$)-**26a** (600 MHz/600 MHz, 298 K, DMSO-*d*$_6$).

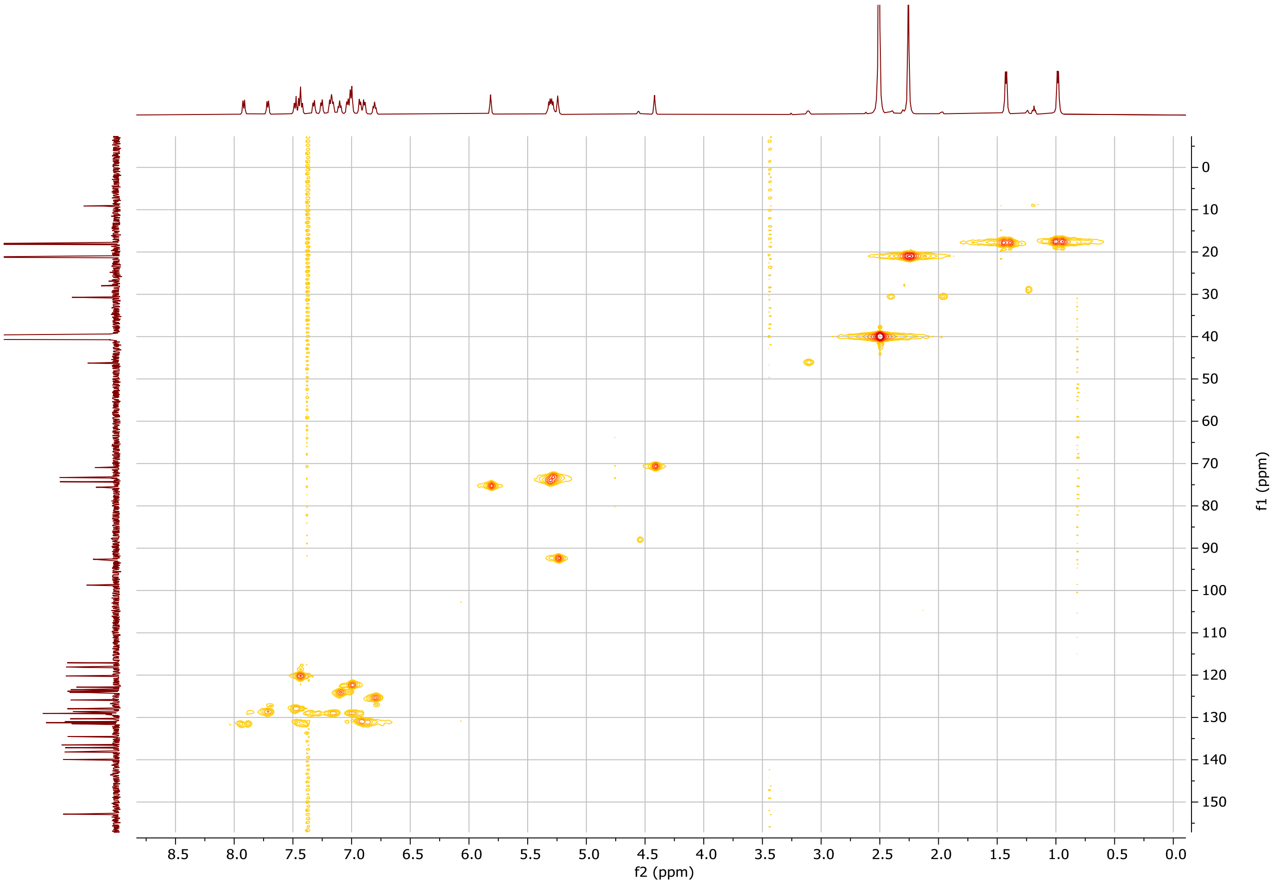

Figure S144: ^{1}H-^{13}C HSQC of (-)-(*M*,*R*,*R*,*R*$_p$)-**26a** (600 MHz/151 MHz, 298 K, DMSO-*d*$_6$).

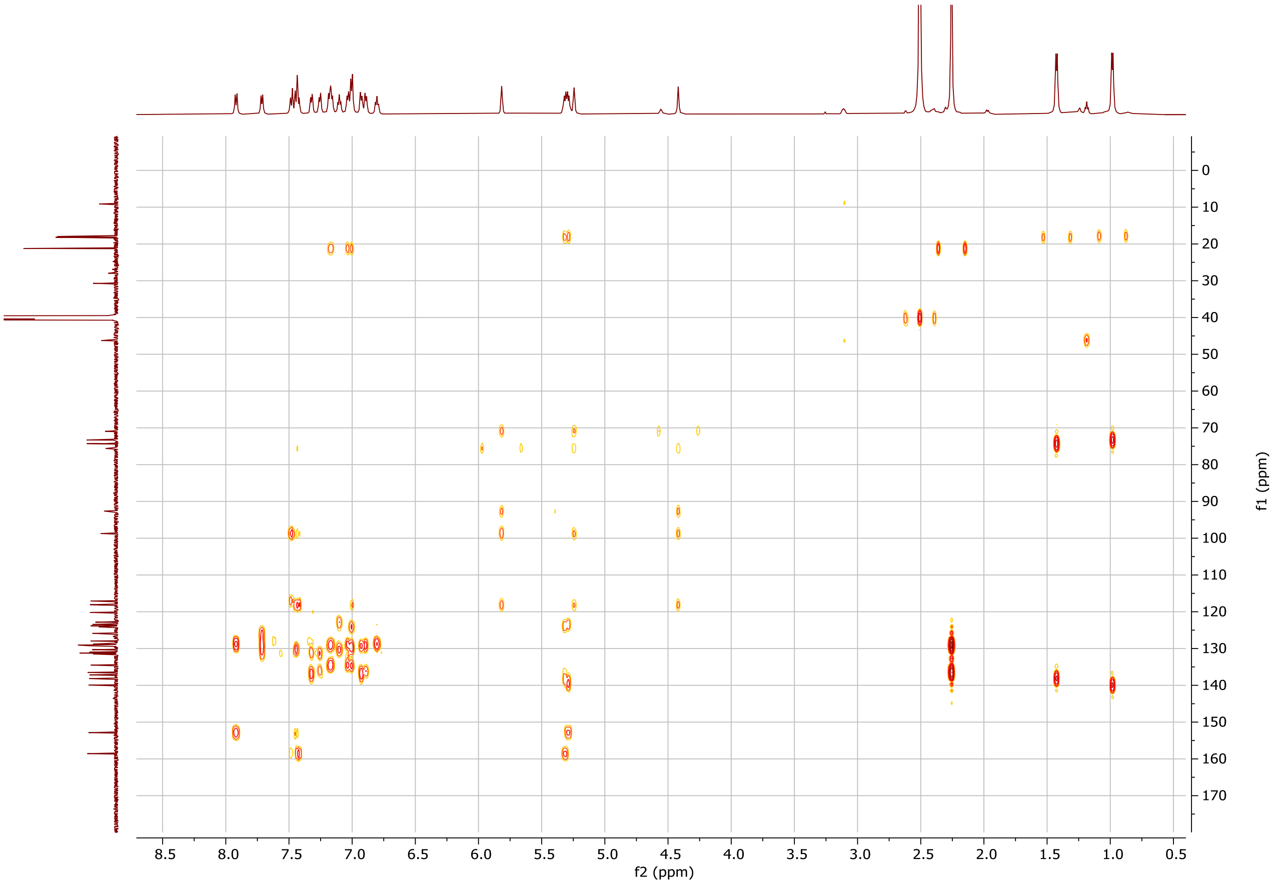

Figure S145: ^{1}H-^{13}C HMBC of (-)-(*M,R,R,R$_p$*)-**26a** (600 MHz/151 MHz, 298 K, DMSO-d_6).

Compound (-)-(*M,R,R,R_p*)-**26b**

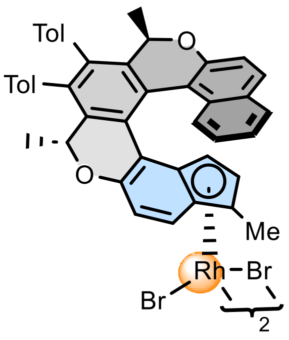

Prepared according to **GP4** starting from (-)-(*M,R,R,R_p*)-**23b** (32 mg, 0.04 mmol, 1.0 equiv.) and bromine (5.0 µL, 15.8 mg, 0.1 mmol, 2.5 equiv.) to give (-)-(*M,R,R,R_p*)-**26b** (24.8 mg, 73%) as a dark red amorphous solid.

[α]$^{20}_{D}$ -2860 (*c* 0.010, THF).

^{1}H NMR (600 MHz, 298 K, DMSO-d_6): δ = 7.91 (d, *J* = 8.7 Hz, 1H, CH^{Ar}), 7.72 (d, *J* = 8.1 Hz, 1H, CH^{Ar}), 7.49 – 7.40 (m, 3H, CH^{Ar}), 7.35 (m, 1H, *o*-proton of *p*-tolyl), 7.24 (m, 1H, *o*-proton of *p*-tolyl), 7.16 (m, 2H, *m*-protons of *p*-tolyl), 7.11 (t, *J* = 7.5 Hz, 1H, CH^{Ar}), 7.03 (m, 1H, *m*-proton of *p*-tolyl), 6.99 (m, 1H, *m*-proton of *p*-tolyl), 6.94 – 6.87 (m, 3H, *o*-protons of *p*-tolyl overlapping with CH^{Ar}), 6.75 (t, *J* = 7.7 Hz, 1H, CH^{Ar}), 5.35 – 5.25 (m, 2H, C*H* chiral center), 5.20 (d, *J* = 2.6 Hz, 1H, Cp*H*), 4.18 (d, *J* = 2.6 Hz, 1H, Cp*H*), 2.25 (s, 6H, CH_3 tolyl), 1.74 (s, 3H, CpCH_3), 1.48 (d, *J* = 6.8 Hz, 3H, CH_3 chiral center), 0.98 (d, *J* = 6.7 Hz, 3H, CH_3 chiral center).

^{13}C{^{1}H} NMR (151 MHz, 298 K, DMSO-d_6): δ = 159.2, 153.0, 140.0, 138.2, 137.9, 137.2, 136.5 (2C), 134.6, 134.5, 131.4, 131.2, 131.0, 130.7, 130.4, 129.3, 129.2, 129.12 (2C), 129.07, 129.0, 128.7, 128.6, 127.0, 125.8, 123.7, 123.5, 123.4, 122.7, 121.6, 120.2, 118.0, 115.5, 95.7, 93.2 (d, *J* = 6.7 Hz), 91.6 (d, *J* = 6.7 Hz), 74.3, 73.3, 66.3 (d, *J* = 7.3 Hz), 21.2 (2C), 17.9, 17.8, 10.9.

HRMS (ESI) *m/z*: ([M-^{79}Br]$^+$) calcd for $C_{88}H_{70}O_4{}^{103}Rh_2{}^{79}Br_3$ 1633.0929, found 1633.0916 (Δ = -0.79 ppm).

IR (ATR): 3050 (vw), 3021 (vw), 2923 (m), 2865 (w), 1615 (w), 1598 (m), 1542 (w), 1514 (m), 1481 (w), 1463 (m), 1422 (m), 1378 (m), 1362 (m), 1293 (w), 1263 (m), 1246 (w), 1222 (m), 1182 (w), 1164 (w), 1152 (w), 1143 (w), 1107 (w), 1077 (w), 1060 (s), 1032 (m), 1020 (m), 1007 (m), 987 (w), 958 (w), 904 (w), 865 (w), 837 (m), 810 (vs), 771 (w), 751 (s), 718 (w), 699 (w), 679 (w), 661 (w), 633 (w), 618 (w), 604 (w), 591 (w), 567 (w), 554 (w), 526 (m), 485 (m), 459 (m), 427 (m) cm^{-1}.

Satisfactory **EA** was not obtained.

UV/VIS (THF): λ_{max} (log ε) = 304 (4.69), 350 (4.51) nm.

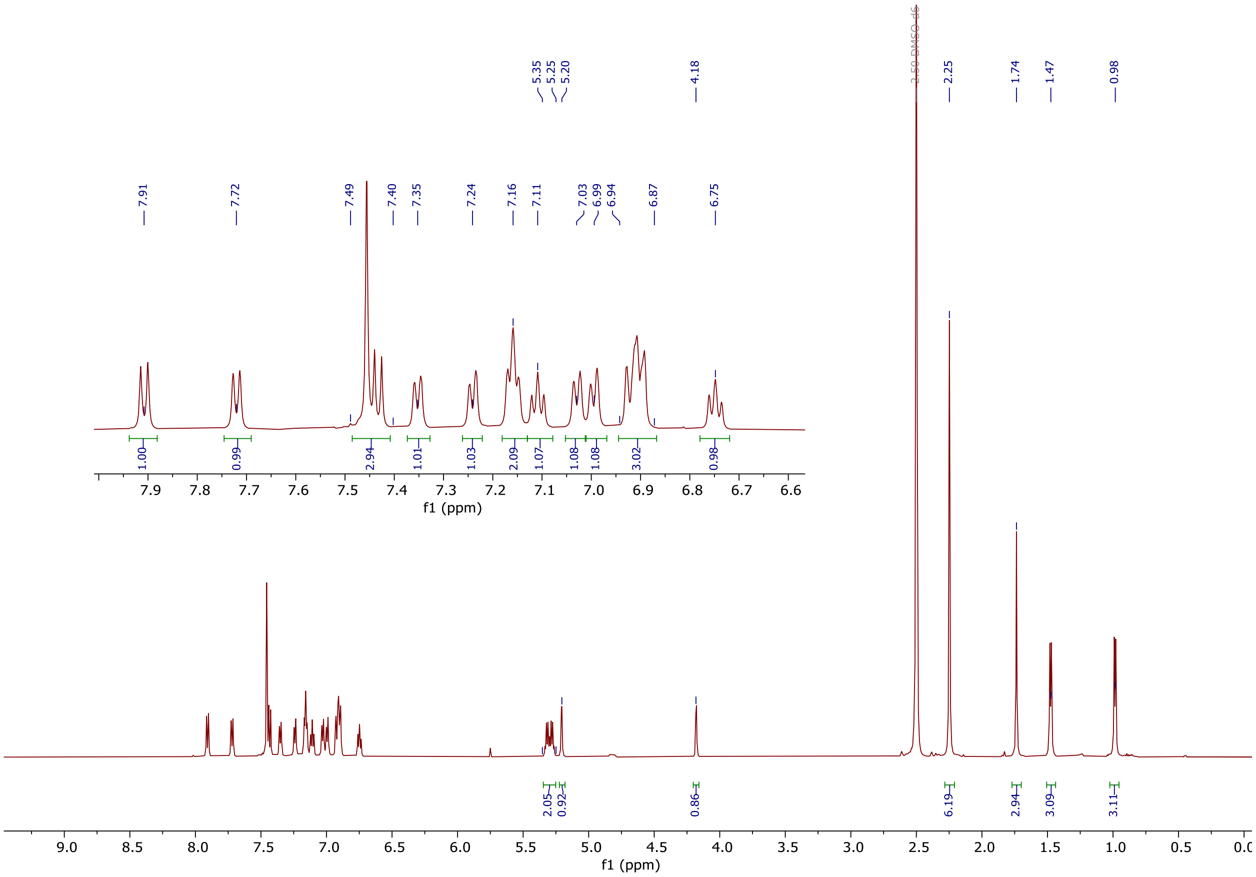

Figure S146: ^{1}H NMR of (-)-(*M,R,R,R$_p$*)-**26b** (600 MHz, 298 K, DMSO-*d$_6$*).

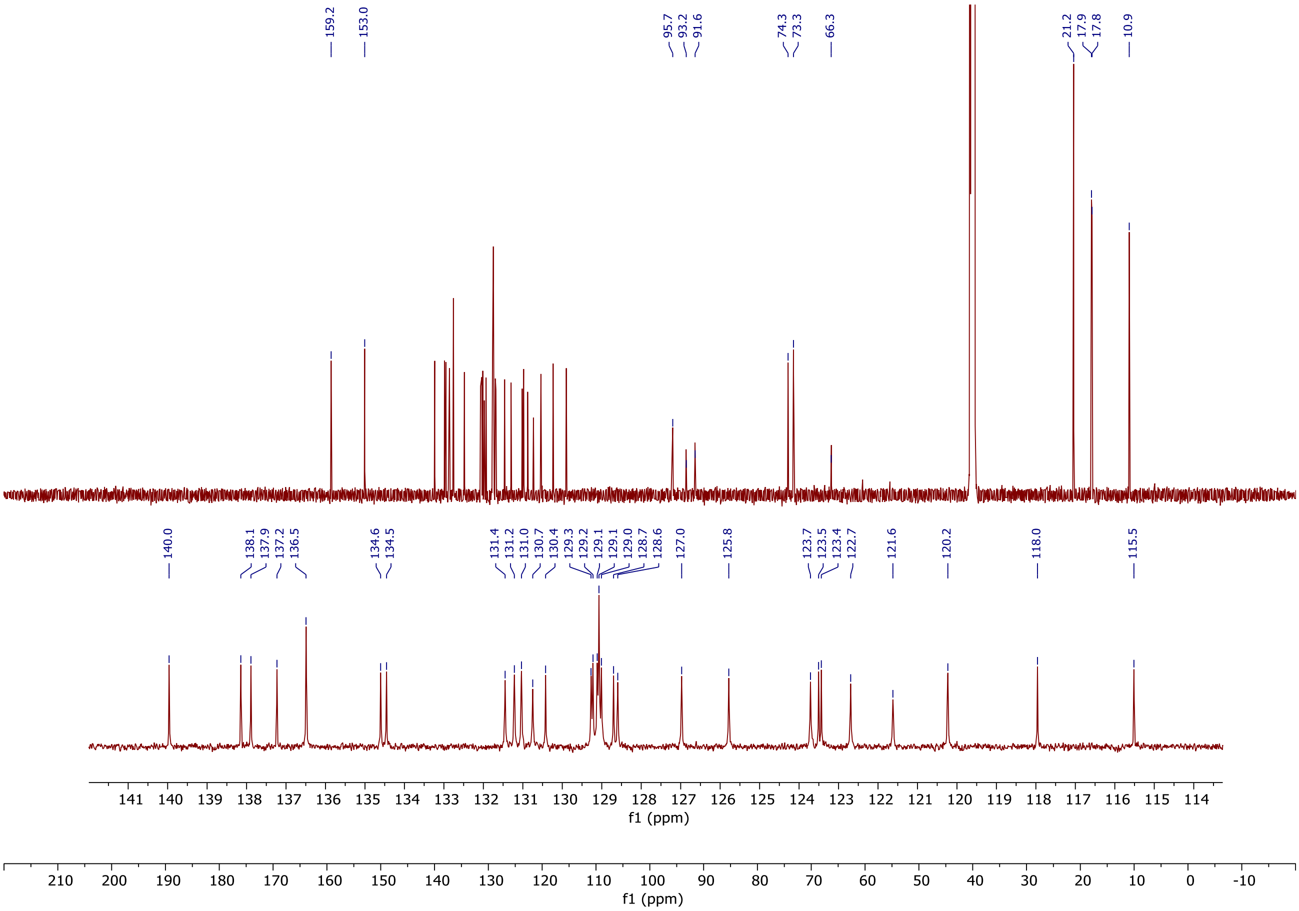

Figure S147: ^{13}C NMR of (-)-(*M,R,R,R$_p$*)-**26b** (151 MHz, 298 K, DMSO-*d$_6$*).

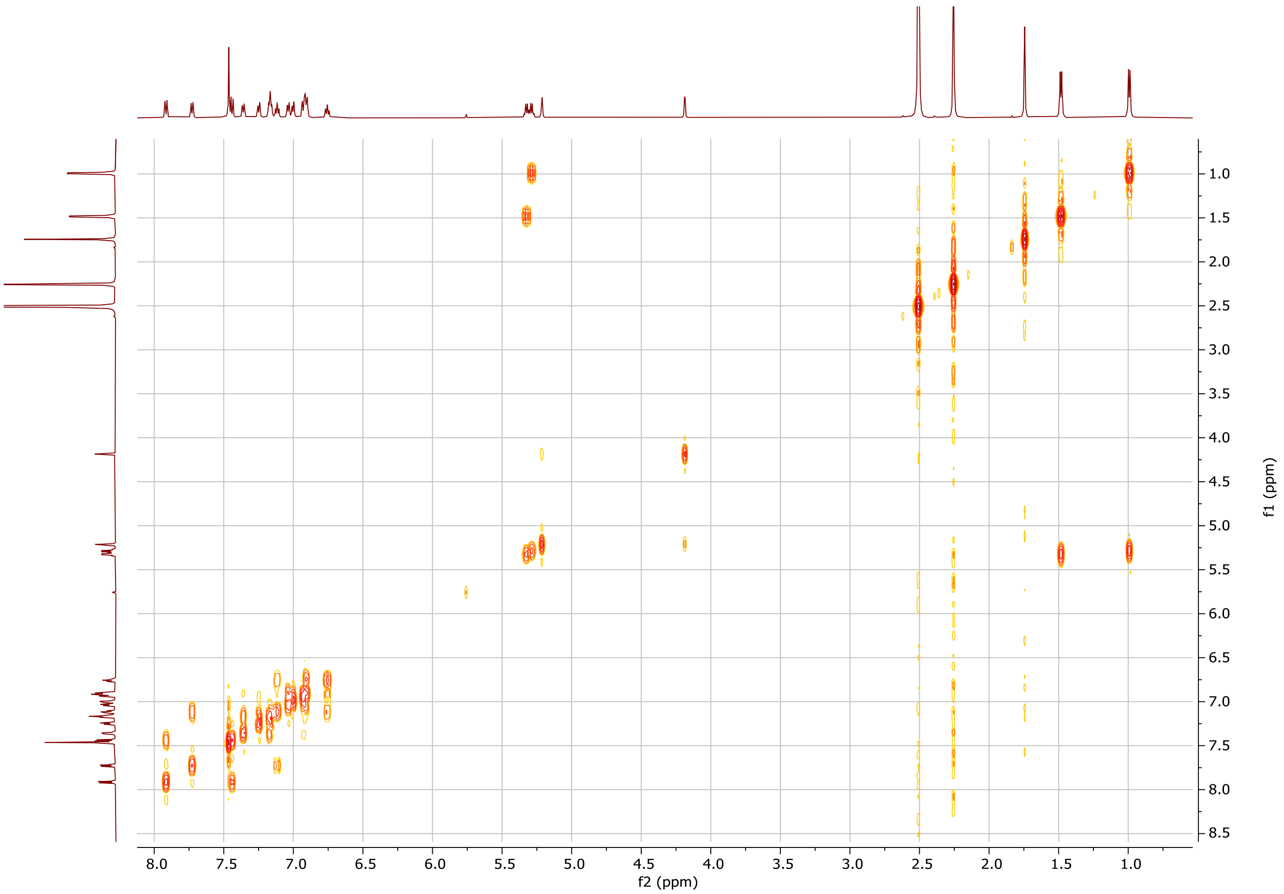

Figure S148: ^{1}H-^{1}H COSY of (-)-(*M,R,R,R$_p$*)-**26b** (600 MHz/600 MHz, 298 K, DMSO-*d$_6$*).

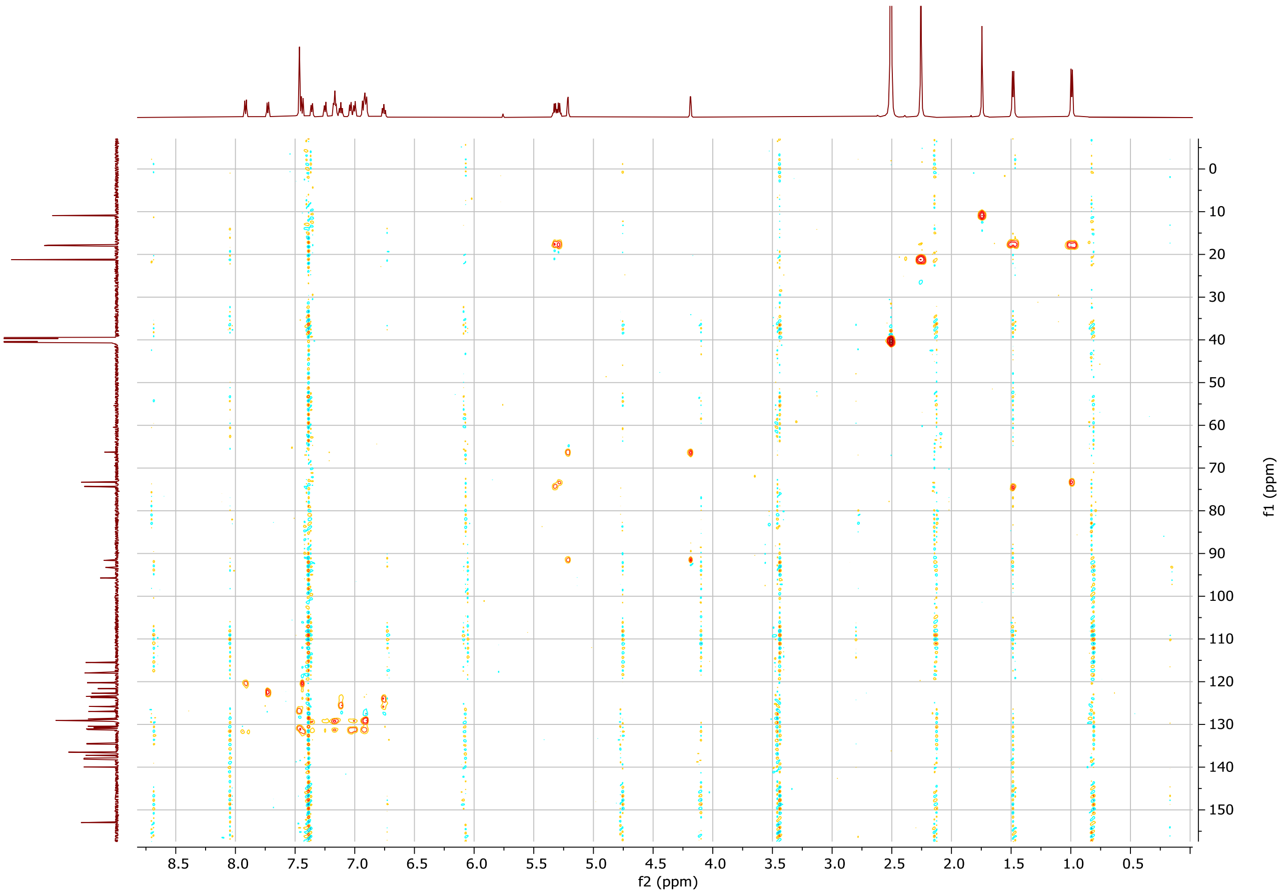

Figure S149: ^{1}H-^{13}C HSQC of (-)-(*M,R,R,R$_p$*)-**26b** (600 MHz/151 MHz, 298 K, DMSO-*d$_6$*).

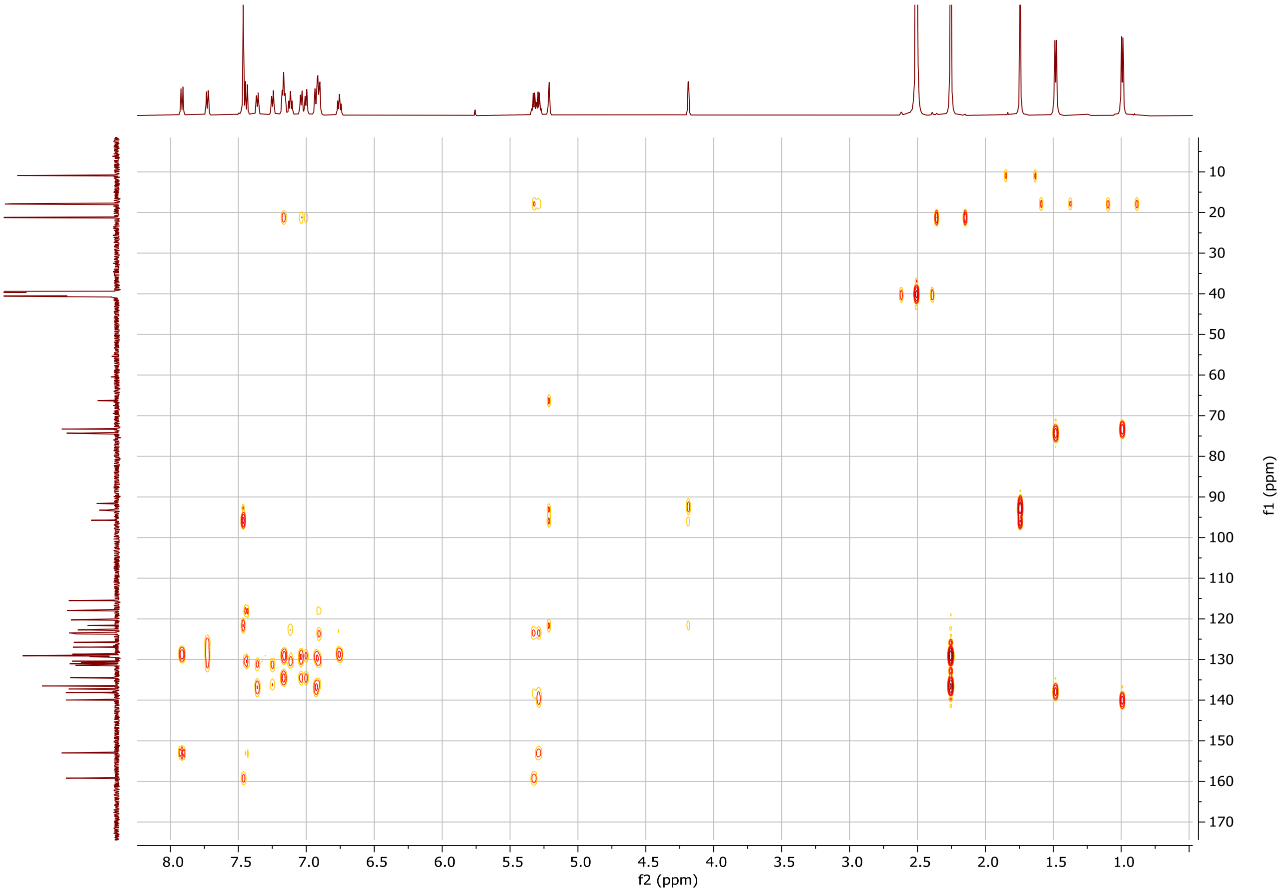

Figure S150: ^{1}H-^{13}C HMBC of (-)-(*M,R,R,R$_p$*)-**26b** (600 MHz/151 MHz, 298 K, DMSO-*d$_6$*).

Oxa[6]helicene with only one chiral center - complexes of Rh(I) and Rh(III) (-)-(M,R,R_p)-**24** and (-)-(M,R,R_p)-**27**

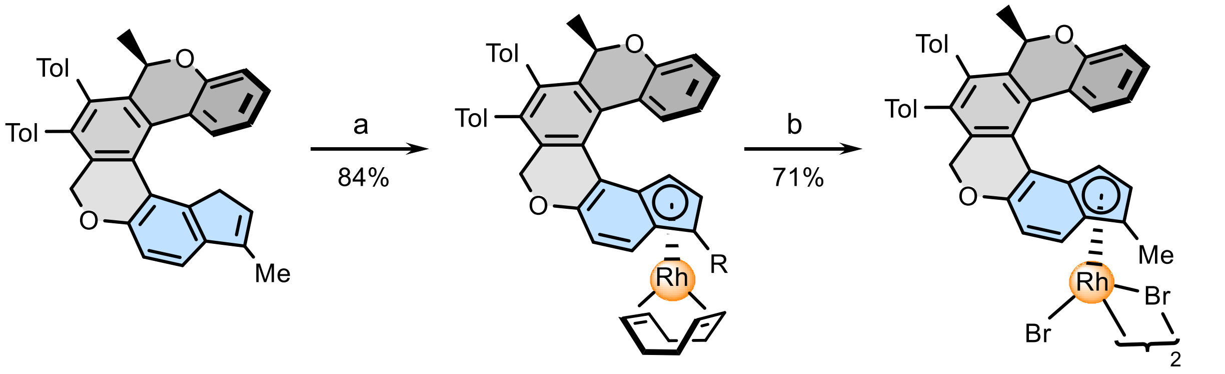

(-)-(*M*,*R*)-**21** (-)-(M,R,R_p)-**24** (-)-(M,R,R_p)-**27**

(a) $[Rh(cod)Cl]_2$ (0.55 equiv.), *t*-BuOK (1.3 equiv.), THF, rt, 2-4 h, 84%; (b) Br_2 (2.5 equiv.), pentane, 0 °C, 14–18 h, 71%.

Compound (-)-(M,R,R_p)-**24**

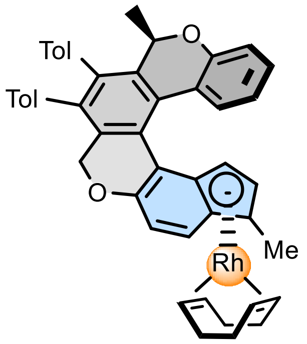

Prepared according to **GP2** from substituted oxa[6]helicene (-)-(*M,R*)-**21** (35 mg, 0.07 mmol, 1.0 equiv.), $[Rh(cod)Cl]_2$ (17.8 mg, 0.04 mmol, 0.55 equiv.), and potassium *tert*-butoxide (9.6 mg, 0.09 mmol, 1.3 equiv.) providing (-)-(M,R,R_p)-**24** (40.8 mg, 84%) as an orange amorphous solid.

$\mathbf{R_f}$ = 0.65 (pentane:toluene 1:1).

$[\alpha]^{20}_D$ -1673 (*c* 0.089, THF).

^{1}H NMR (500 MHz, 298 K, CD_2Cl_2): δ = 7.18 (d, *J* = 8.6 Hz, 1H, CH^{Ar}), 7.13 – 7.03 (m, 6H, 6 CH^{Ar}), 7.03 – 6.94 (m, 5H, 5 CH^{Ar}), 6.76 (dd, *J* = 7.7, 1.9 Hz, 1H, CH^{Ar}), 6.54 (m, 1H, CH^{Ar}), 5.50 (m, 1H, *C*H Cp), 5.08 (q, *J* = 6.6 Hz, 1H, *C*H chiral center), 4.77 (d, *J* = 13.6 Hz, 1H, CH_2^a), 4.42 (d, *J* = 13.6 Hz, 1H, CH_2^b), 4.15 (d, *J* = 2.8 Hz, 1H, *C*H Cp), 3.66 – 3.57 (m, 2H, *C*H COD), 3.11 – 3.03 (m, 2H, *C*H COD), 2.29 (s, 3H, CH_3 tolyl), 2.28 (s, 3H, CH_3 tolyl), 1.87 (s, 3H, CH_3 Cp), 1.84 – 1.66 (m, 6H, CH_2 COD), 1.64 – 1.57 (m, 2H, CH_2 COD), 1.09 (d, *J* = 6.6 Hz, 3H, CH_3 chiral center).

^{13}C{^{1}H} NMR (126 MHz, 298 K, CD_2Cl_2): δ = 154.4, 153.2, 139.3, 137.6, 137.3, 137.0, 136.9, 136.5, 136.0, 135.8, 131.8, 130.6, 130.1, 129.9, 129.12, 129.07, 129.04, 129.03, 128.7, 128.1, 127.5, 122.0, 119.7, 119.0, 115.0, 114.5, 110.4 (d, *J* = 2.6 Hz), 109.2 (d, *J* = 2.2 Hz), 93.3 (d, *J* = 5.1 Hz), 88.9 (d, *J* = 4.1 Hz), 75.6 (d, *J* = 4.3 Hz), 73.4, 72.1 (d, *J* = 13.7 Hz, 2C), 69.3, 68.7 (d, *J* = 13.4 Hz, 2C), 32.0 (2C), 31.5 (2C), 21.4 (2C), 19.5, 10.7.

HRMS (ESI) *m/z*: ($[M]^+$) calcd for $C_{47}H_{43}O_2{}^{103}Rh$ 742.2313 found 742.2310 (Δ = -0.37 ppm).

IR (ATR): 3020 (vw), 2921 (s), 2867 (m), 2823 (m), 1771 (w), 1724 (w), 1604 (w), 1585 (w), 1545 (w), 1516 (w), 1484 (m), 1456 (m), 1410 (w), 1363 (m), 1321 (w-m), 1296 (vw), 1259 (w), 1244 (w), 1213 (s), 1167 (w), 1143 (w), 1110 (w), 1080 (m), 1061 (m), 1031 (s), 1007 (vs), 975 (w), 956 (w), 842 (m), 831 (m), 817 (m-s), 799 (vs), 753 (vs), 741 (s), 686 (w), 526 (m), 483 (m) cm^{-1}.

EA: Calcd for $C_{47}H_{43}O_2Rh$: C, 76.00%; H, 5.84%. Found: C, 76.45%; H, 6.05%.

UV/VIS (THF): λ_{max} (log ε) = 268 (4.37), 370 (4.00) nm.

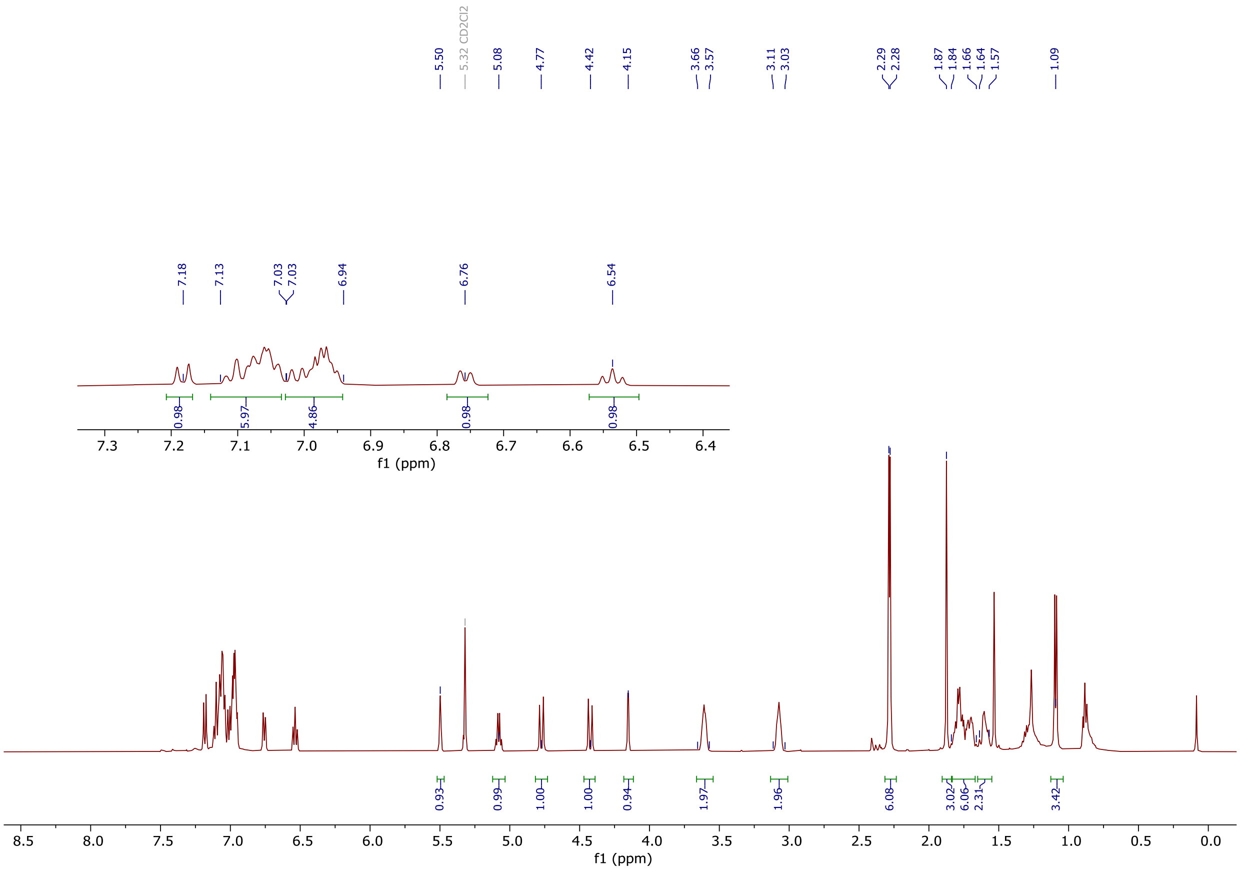

Figure S151: ^{1}H NMR of (-)-(*M,R,R$_p$*)-**24** (500 MHz, 298 K, CD_2Cl_2).

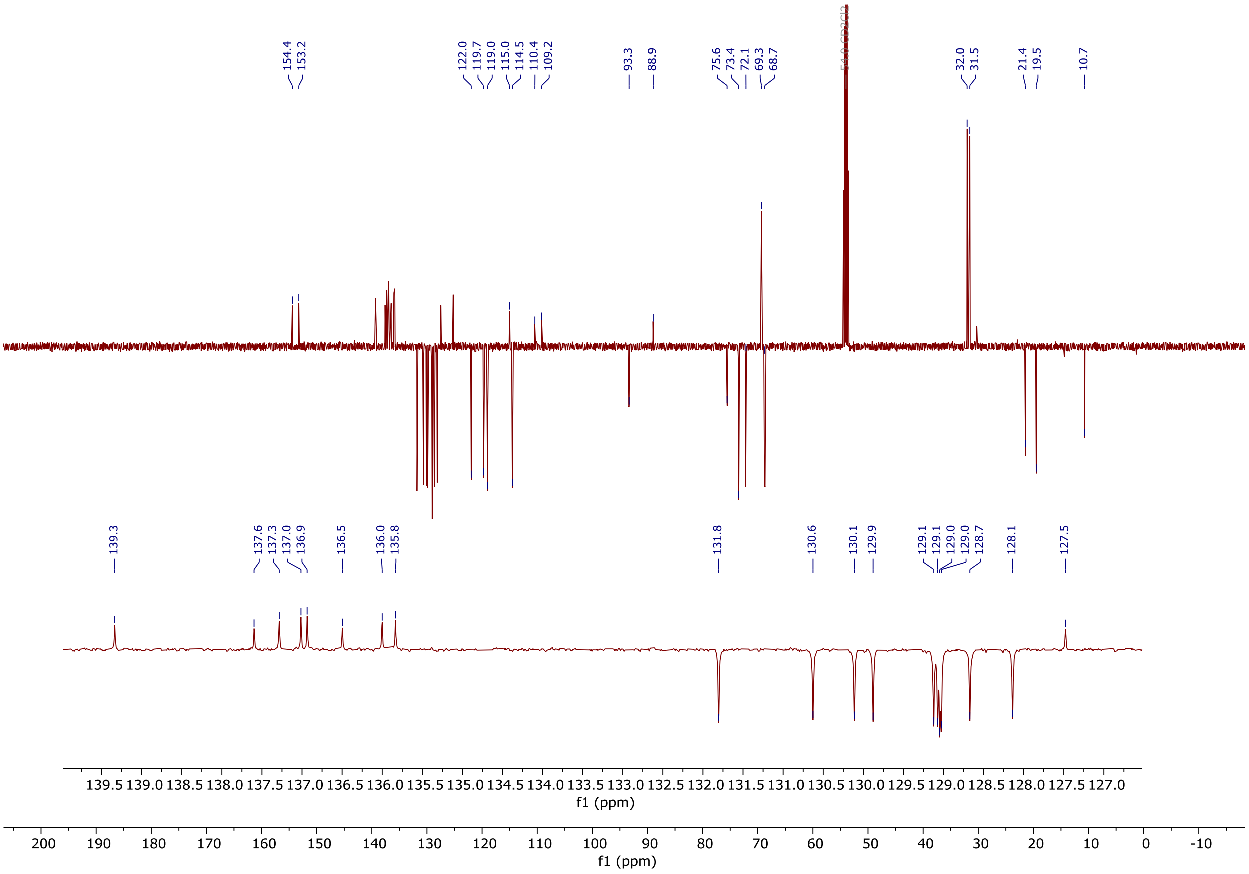

Figure S152: ^{13}C NMR of (-)-(*M,R,R$_p$*)-**24** (126 MHz, 298 K, CD_2Cl_2).

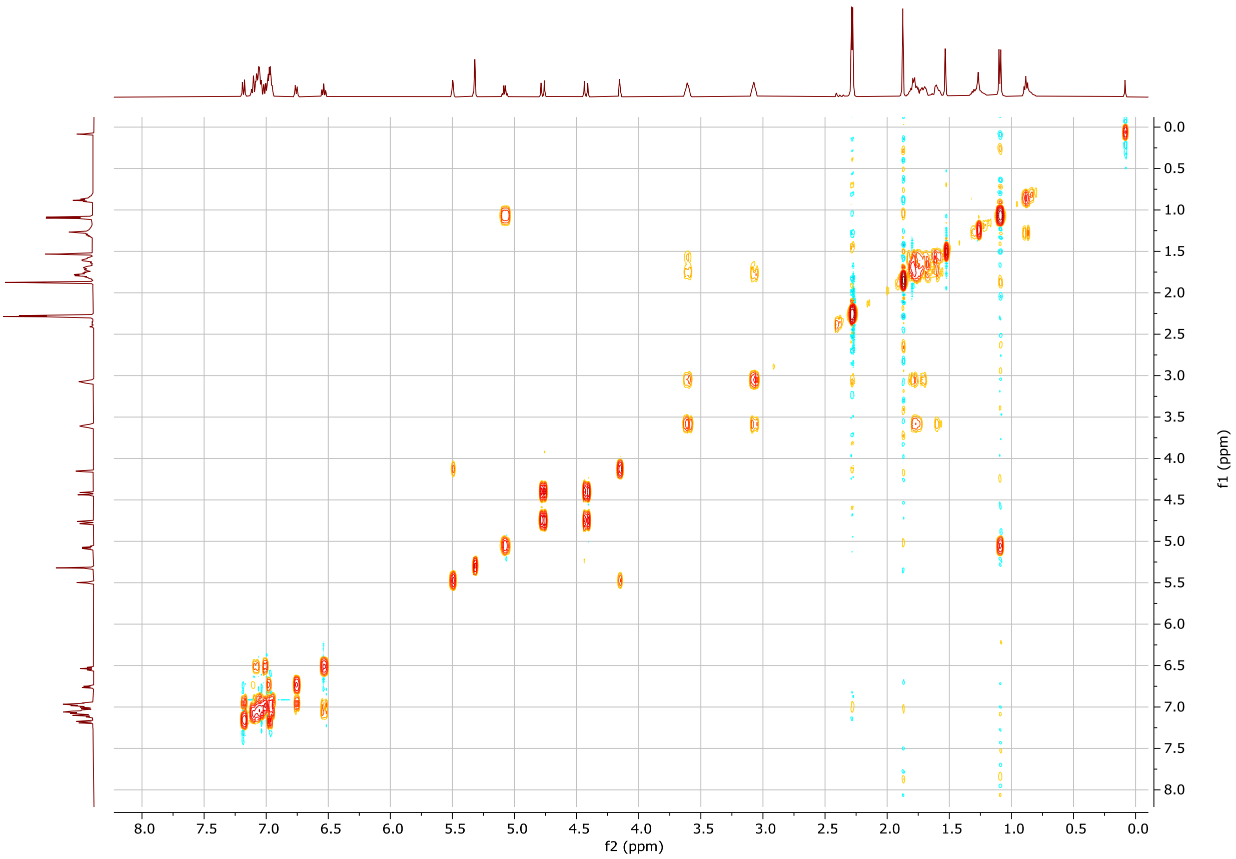

Figure S153: ^{1}H-^{1}H COSY of (-)-(*M,R,R$_p$*)-**24** (500 MHz/500 MHz, 298 K, CD_2Cl_2).

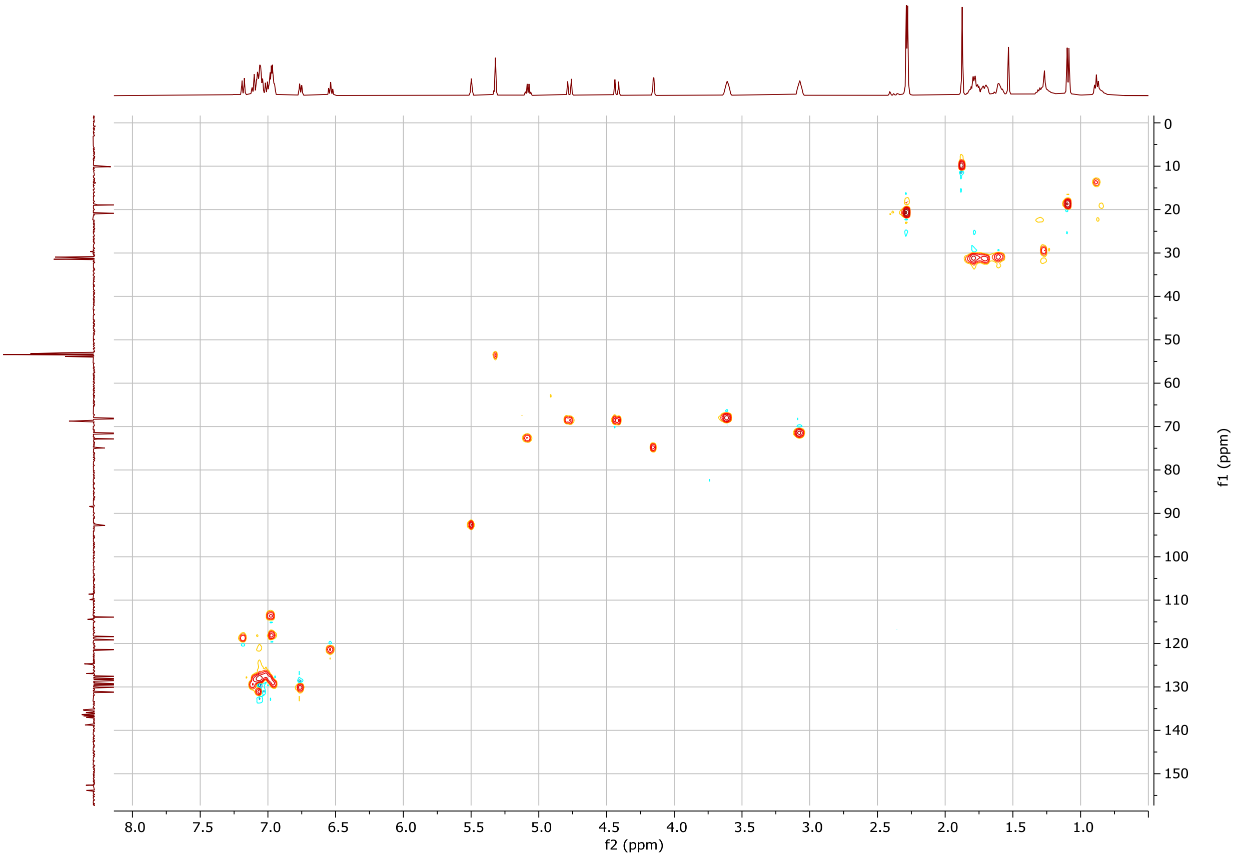

Figure S154: ^{1}H-^{13}C HSQC of (-)-(*M,R,R$_p$*)-**24** (500 MHz/126 MHz, 298 K, CD_2Cl_2).

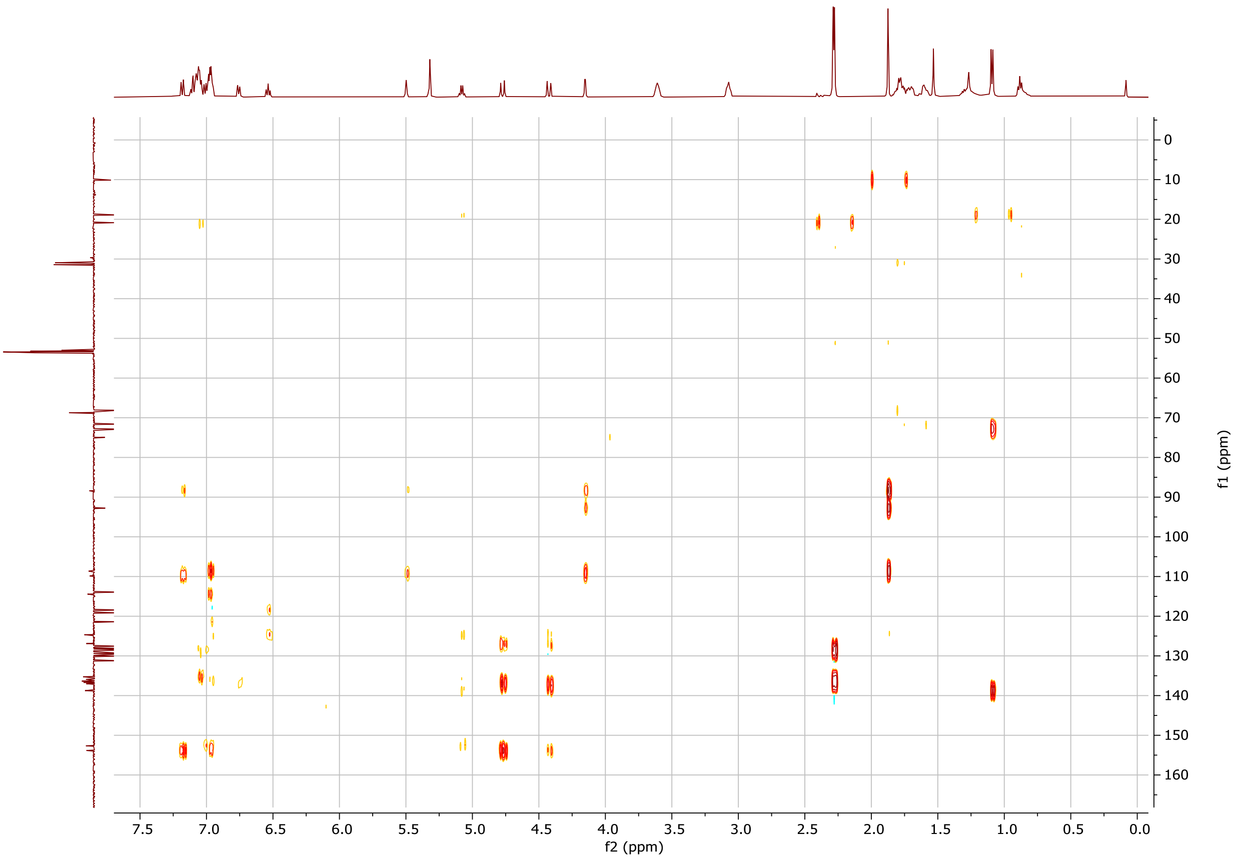

Figure S155: ^{1}H-^{13}C HMBC of (-)-(*M,R,R$_p$*)-**24** (500 MHz/126 MHz, 298 K, DMSO-*d$_6$*).

Compound (-)-(M,R,R_p)-**27**

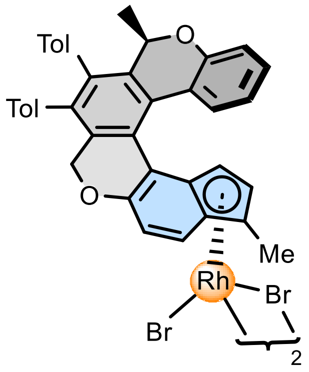

Prepared according to **GP4** starting from (-)-(M,R,R_p)-**24** (35 mg, 0.05 mmol, 1.0 equiv.) and bromine (6.0 µL, 18.8 mg, 0.12 mmol, 2.5 equiv.) to give (-)-(M,R,R_p)-**27** (26.5 mg, 71%) as a dark red amorphous solid.

[α]$^{20}_{D}$ -2950 (*c* 0.010, THF).

^{1}H NMR (600 MHz, 298 K, DMSO-d_6): δ = 7.72 (d, J = 8.9 Hz, 1H, CH^{Ar}), 7.51 (d, J = 8.9 Hz, 1H, CH^{Ar}), 7.24 (m, 1H, CH^{Ar}), 7.19 – 7.16 (m, 1H, CH^{Ar}), 7.13 – 7.01 (m, 7H, 7 CH^{Ar}), 6.81 (m, 1H, CH^{Ar}), 6.77 (m, 1H, CH^{Ar}), 6.69 (m, 1H, CH^{Ar}), 5.92 (d, J = 2.6 Hz, 1H, C*H* Cp), 5.02 (q, J = 6.6 Hz, 1H, C*H* chiral center), 4.82 (d, J = 13.2 Hz, 1H, CH_2^a), 4.72 (d, J = 13.2 Hz, 1H, CH_2^b), 4.35 (d, J = 2.6 Hz, 1H, C*H* Cp), 2.25 (s, 3H, CH_3 tolyl), 2.24 (s, 3H, CH_3 tolyl), 2.19 (s, 3H CH_3 Cp), 1.04 (d, J = 6.6 Hz, 3H, CH_3 chiral center).

^{13}C{^{1}H} NMR (151 MHz, 298 K, DMSO-d_6): δ = 163.5, 152.6, 138.6, 137.5, 137.3, 136.3, 136.13, 136.07, 134.3, 134.1, 130.9, 129.9, 129.7, 129.6, 129.0, 128.74, 128.71, 128.66, 128.6, 128.2, 127.4, 125.6, 123.9, 123.8, 122.7, 121.7, 119.2, 117.7, 115.4, 97.0 (d, J = 6.1 Hz), 95.7, 91.7 (d, J = 6.3 Hz), 72.2, 70.2, 67.4 (d, J = 5.9 Hz), 20.7 (2C), 18.6, 11.4.

HRMS (ESI) *m/z*: ([M-^{79}Br]$^+$ calcd for $C_{78}H_{62}O_4{}^{79}Br_3{}^{103}Rh$ 1505.0303, found 1505.0289 (Δ = -0.93 ppm).

IR (ATR): 3020 (vw), 2977 (w), 2955 (w), 2921 (w), 2865 (w), 1588 (m-s), 1537 (w), 1516 (m), 1483 (m), 1441 (m-s), 1419 (s), 1367 (m), 1331 (w-m), 1297 (w), 1260 (m-s), 1220 (s), 1183 (w), 1142 (m), 1104 (m), 1083 (w-m), 1061 (m-s), 1022 (w-m), 1001 (s), 949 (w), 895 (w), 846 (m), 819 (s), 755 (vs), 741 (s), 726 (w), 682 (m), 636 (m), 598 (m), 575 (w), 526 (s), 501 (s), 465 (m) cm^{-1}.

UV/VIS (THF): λ_{max} (log ε) = 274 (4.65), 320 (4.47) nm.

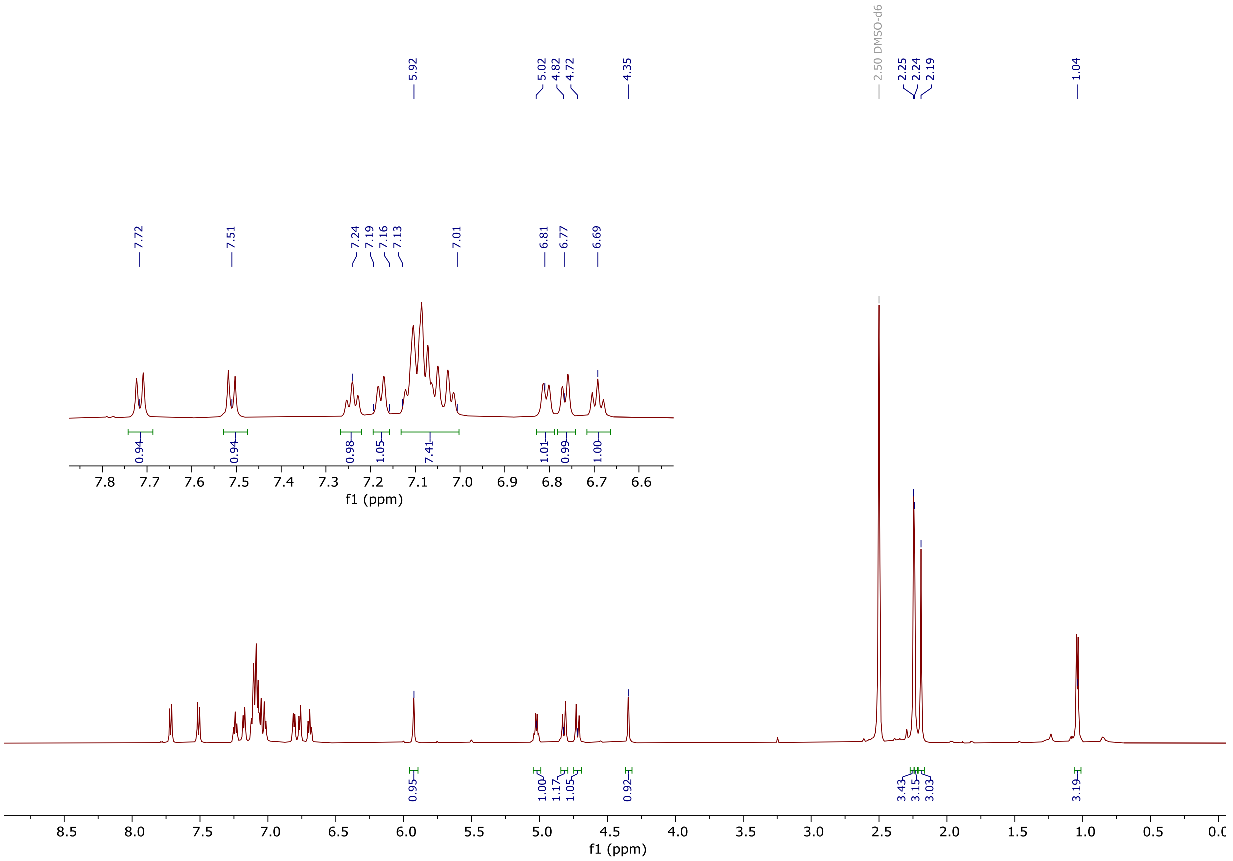

Figure S156: ^{1}H NMR of (-)-(M,R,R_p)-**27** (600 MHz, 298 K, DMSO-d_6).

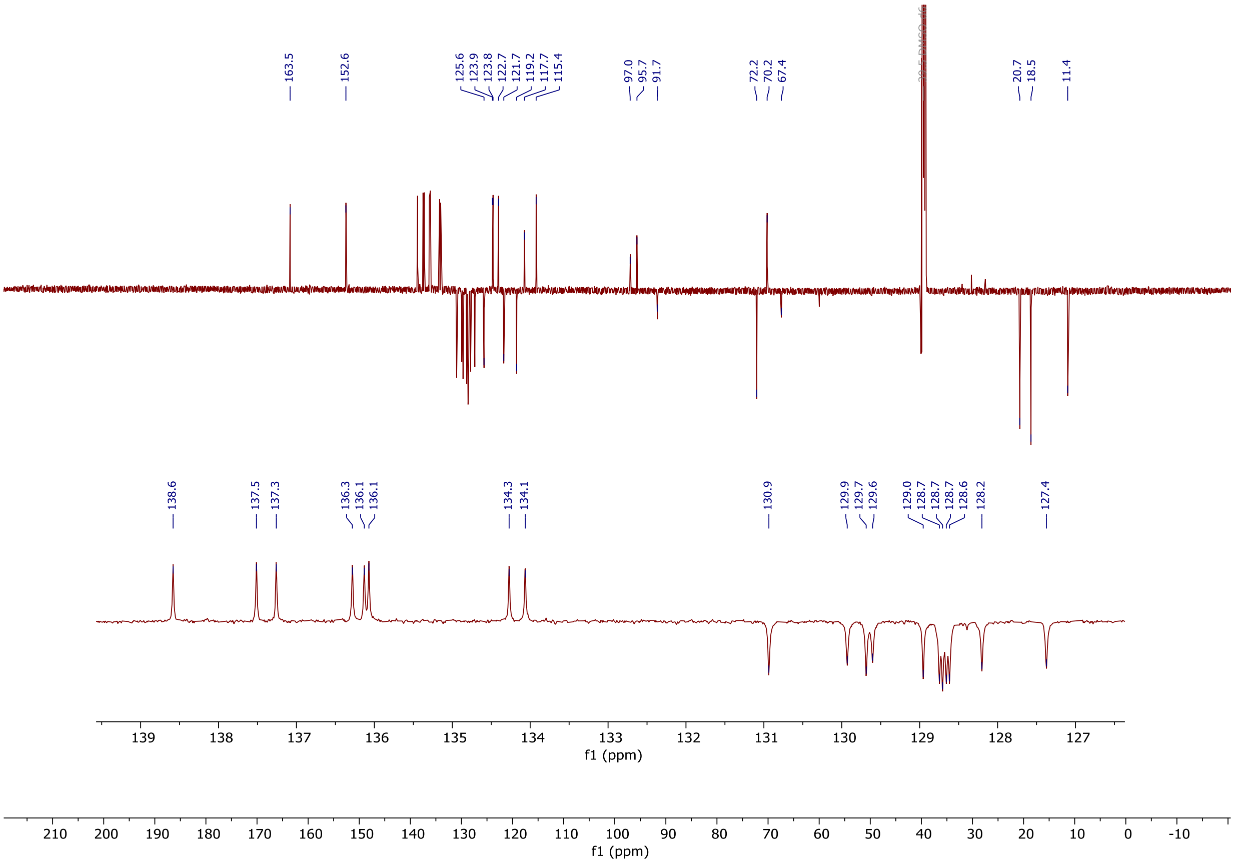

Figure S157: ^{13}C NMR of (-)-(M,R,R_p)-**27** (151 MHz, 298 K, DMSO-d_6).

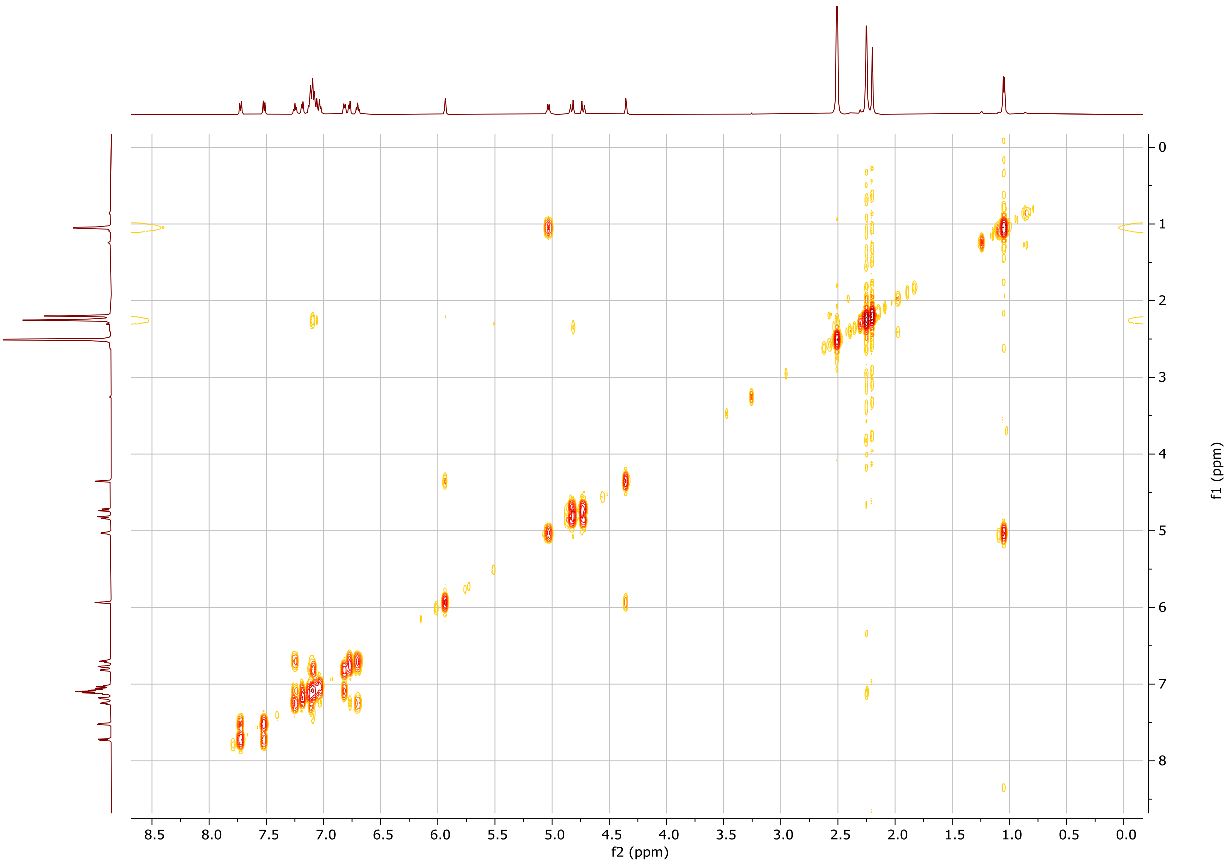

Figure S158: ^{1}H-^{1}H COSY of (-)-(*M,R,R$_p$*)-**27** (600 MHz/600 MHz, 298 K, DMSO-*d$_6$*).

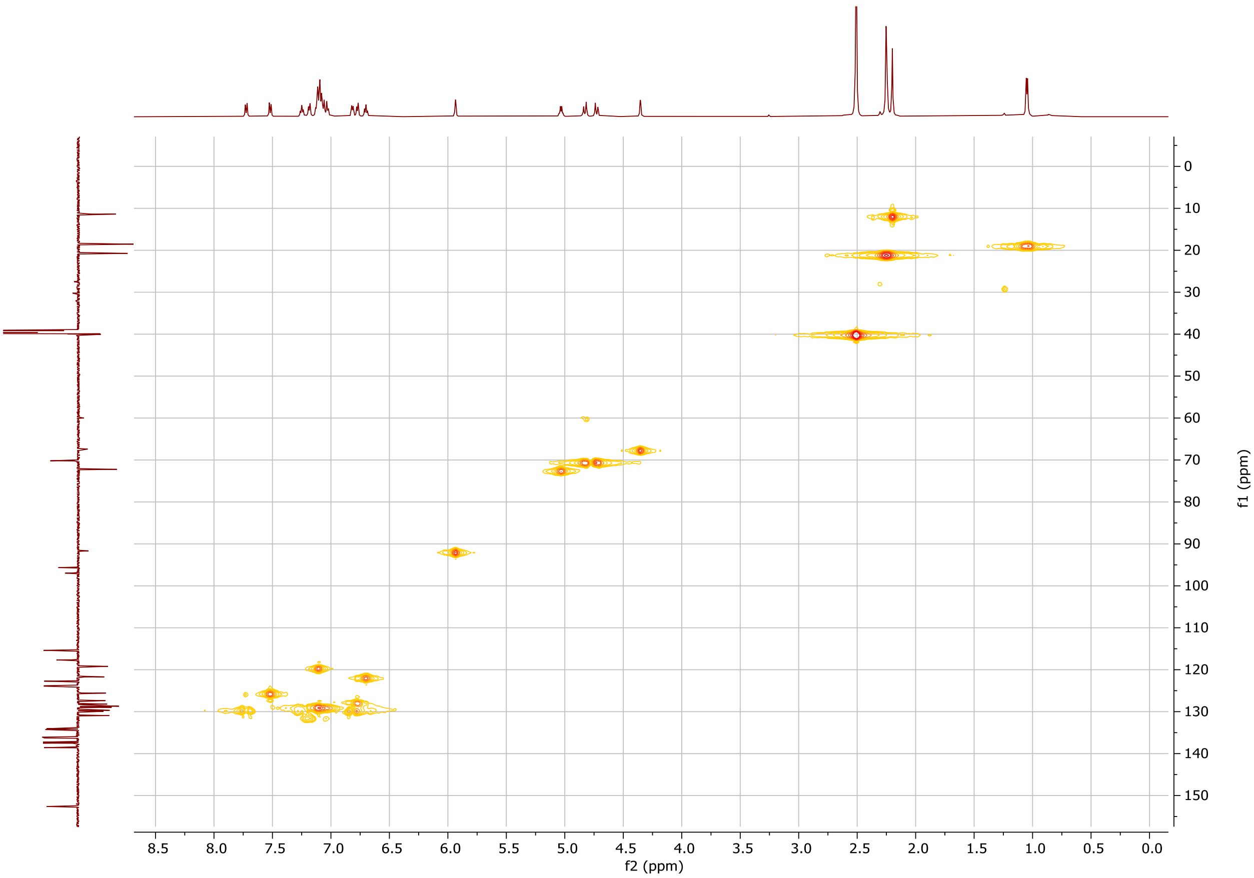

Figure S159: ^{1}H-^{13}C HSQC of (-)-(*M,R,R$_p$*)-**27** (600 MHz/151 MHz, 298 K, DMSO-*d$_6$*).

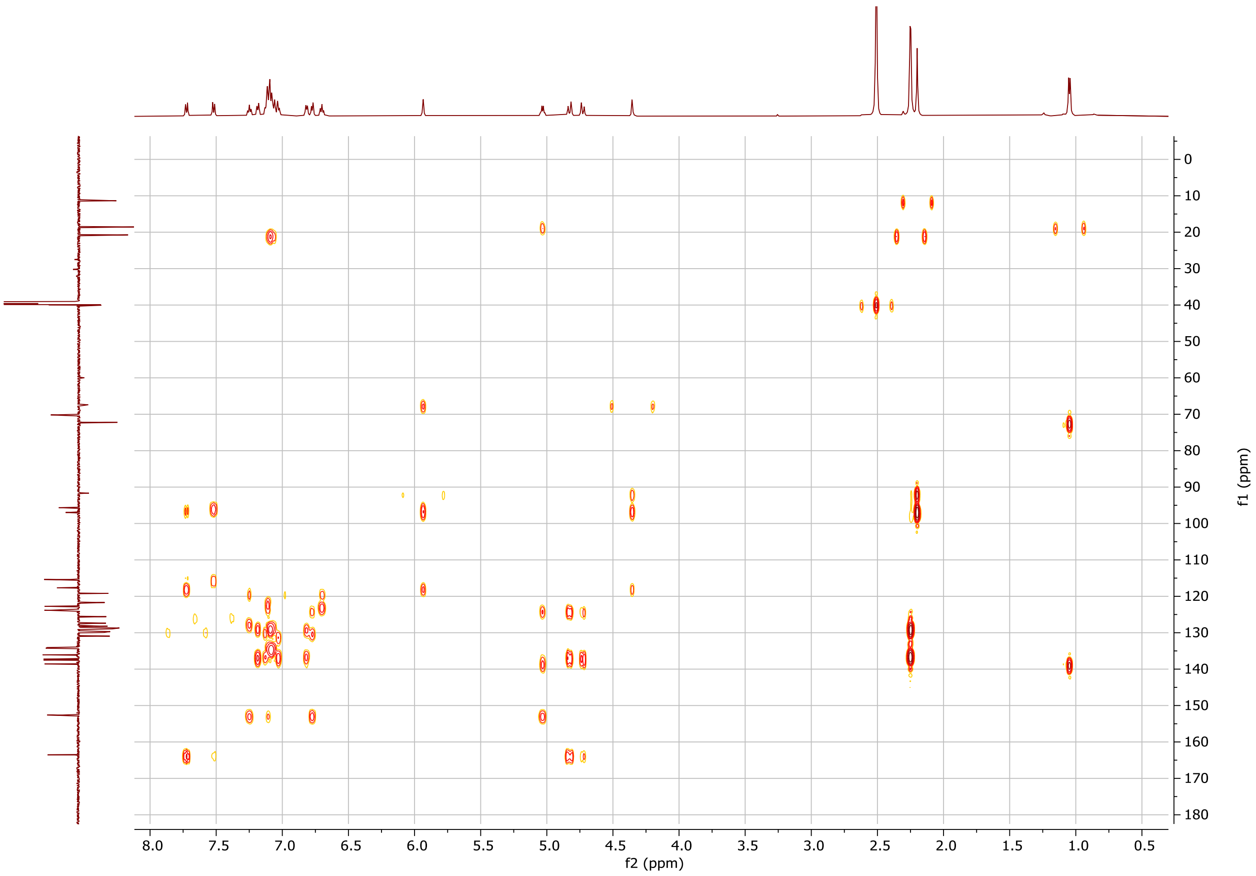

Figure S160: ^{1}H-^{13}C HMBC of (-)-(*M,R,R$_p$*)-**27** (600 MHz/151 MHz, 298 K, DMSO-*d$_6$*).

UV/VIS spectra of the proligands (-)-(*M*,*R*,*R*)-**14a**-**e**, **18**, **20a**,**b**, (-)-(*M*,*R*)-**21**, potassium salt (*M*,*R*,*R*)-**19** and complexes (*M*,*R*,*R*,S_p,S_p)-**7**, (-)-(*M*,*R*,*R*,R_p)-**22a**-**e**, (-)-(*M*,*R*,*R*,R_p)-**23a**,**b**, (-)-(*M*,*R*,R_p)-**24**, (-)-(*M*,*R*,*R*,R_p)-**25a**-**e**, (-)-(*M*,*R*,*R*,R_p)-**26a**,**b** and (-)-(*M*,*R*,R_p)-**27**

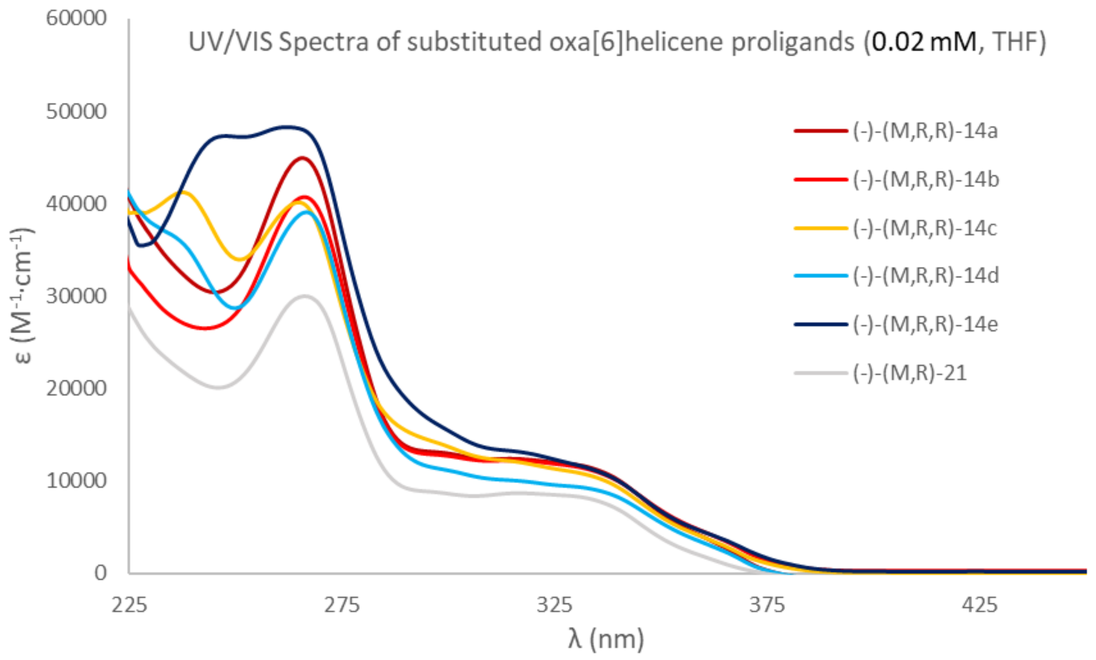

Figure S161: UV/VIS spectra of oxa[6]helicene proligands (-)-(*M*,*R*,*R*)-**14a-e** and (-)-(*M*,*R*)-**21** (0.02 mM, THF).

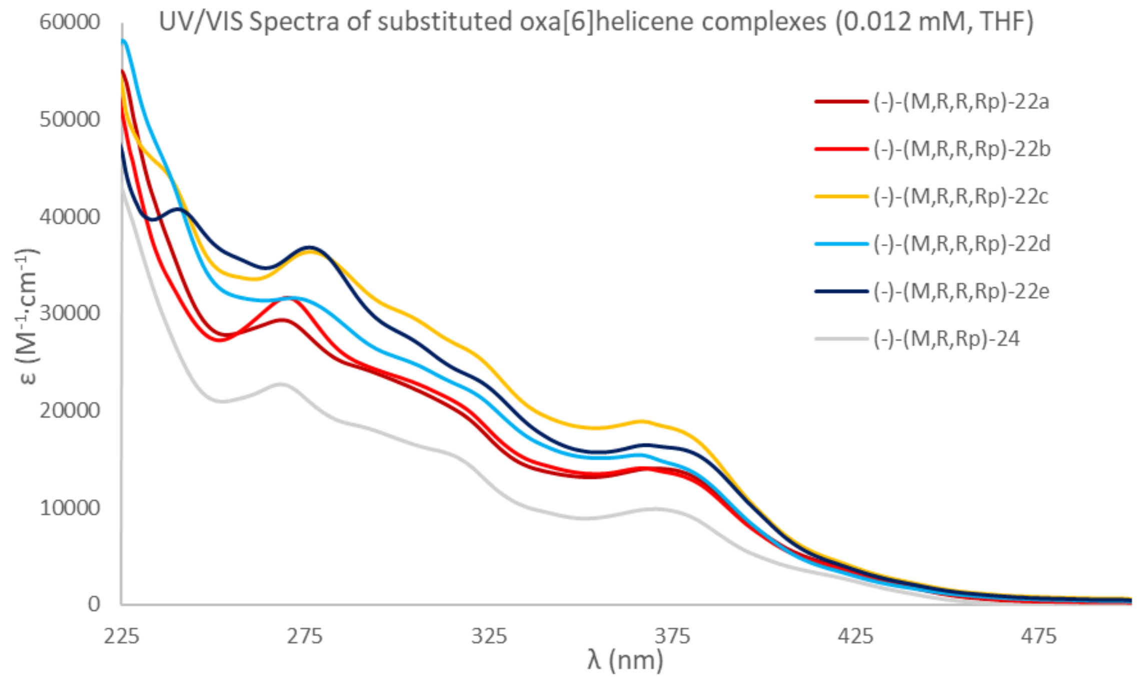

Figure S162: UV/VIS spectra of oxa[6]helicene complexes (-)-(*M*,*R*,*R*,R_p)-**22a-e** and (-)-(*M*,*R*,R_p)-**24** (0.012 mM, THF).

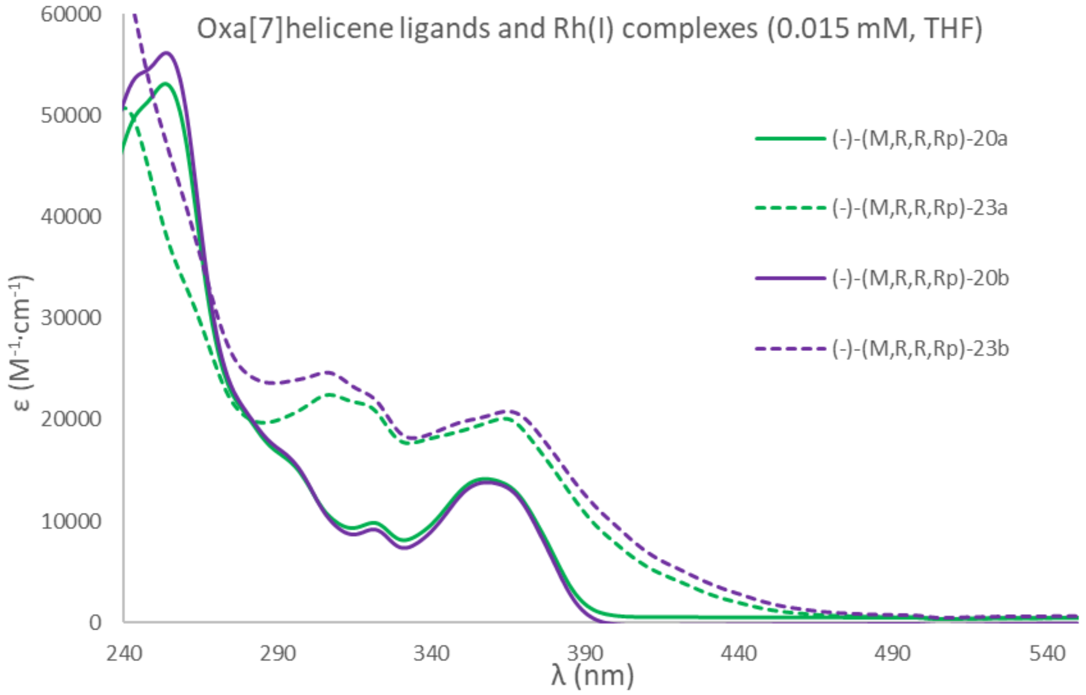

Figure S163: UV/VIS spectra of oxa[7]helicene proligands (-)-(*M*,*R*,*R*,*R*$_p$)-**20a**,**b** and Rh(COD) complexes (-)-(*M*,*R*,*R*,*R*$_p$)-**23a**,**b** (0.015 mM, THF).

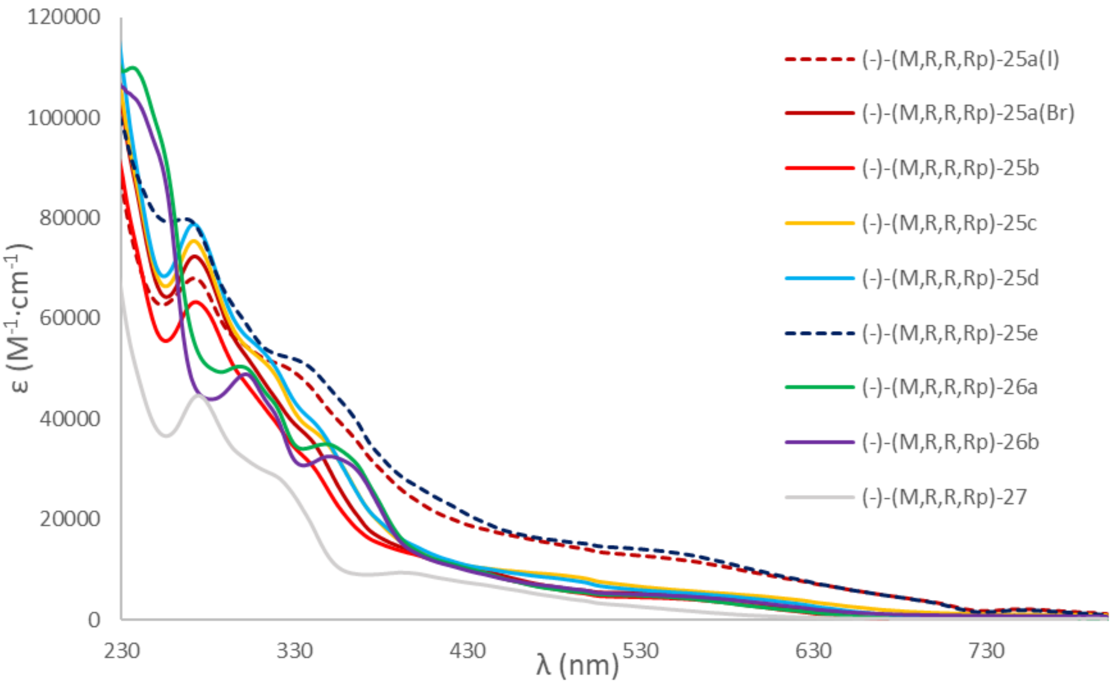

Figure S164: UV/VIS spectra of the oxidized rhodium complexes (-)-(*M*,*R*,*R*,*R*$_p$)-**25a**-**e**, (-)-(*M*,*R*,*R*,*R*$_p$)-**26a**,**b**, and (-)-(*M*,*R*,*R*,*R*$_p$)-**27** (0.01 mM, THF).

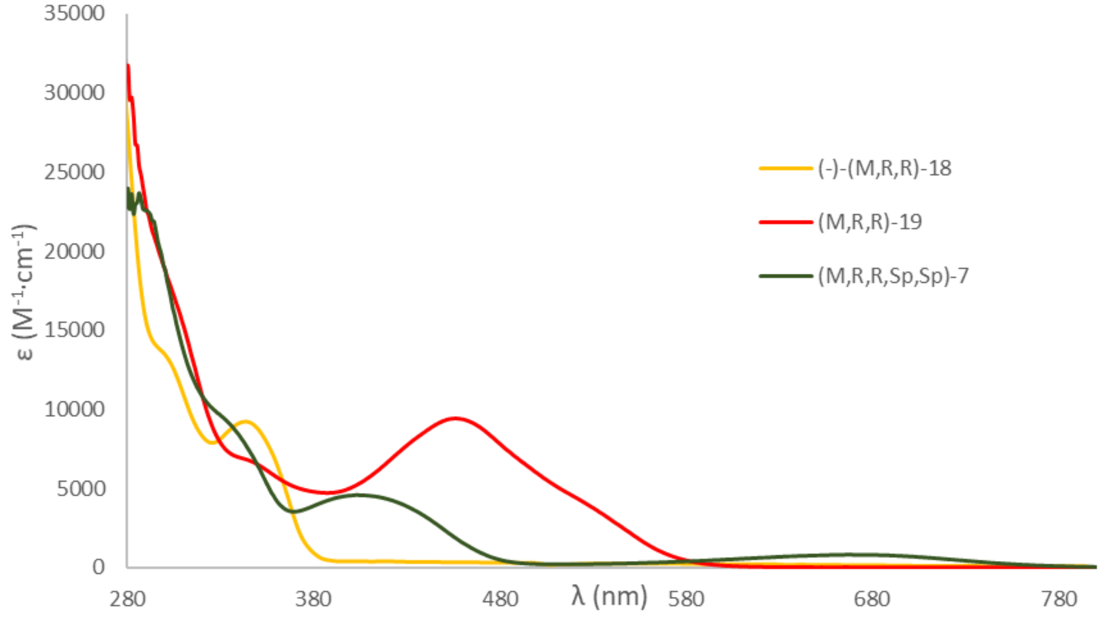

Figure S165: UVVIS spectra of oxa[7]helicene proligand (-)-(*M,R,R*)-**18**, potassium salt (*M,R,R*)-**19**, and iron complex (*M,R,R,S$_p$,S$_p$*)-**7** (THF)

ECD spectra of selected compounds (-)-(*M*,*R*,*R*)- and (+)-(*P*,*S*,*S*)-**14c** and (-)-(*M*,*R*,*R*,*R$_p$*)- and (+)-(*P*,*S*,*S*,*S$_p$*)-**22e**

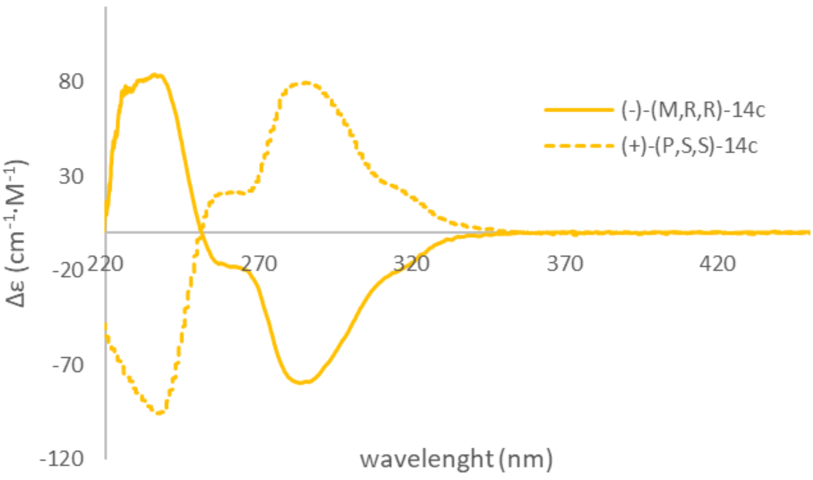

Figure S166: ECD spectra of oxa[6]helicene proligands (-)-(*M,R,R*)-**14c** and (+)-(*P,S,S*)-**14c** (0.1 mM THF).

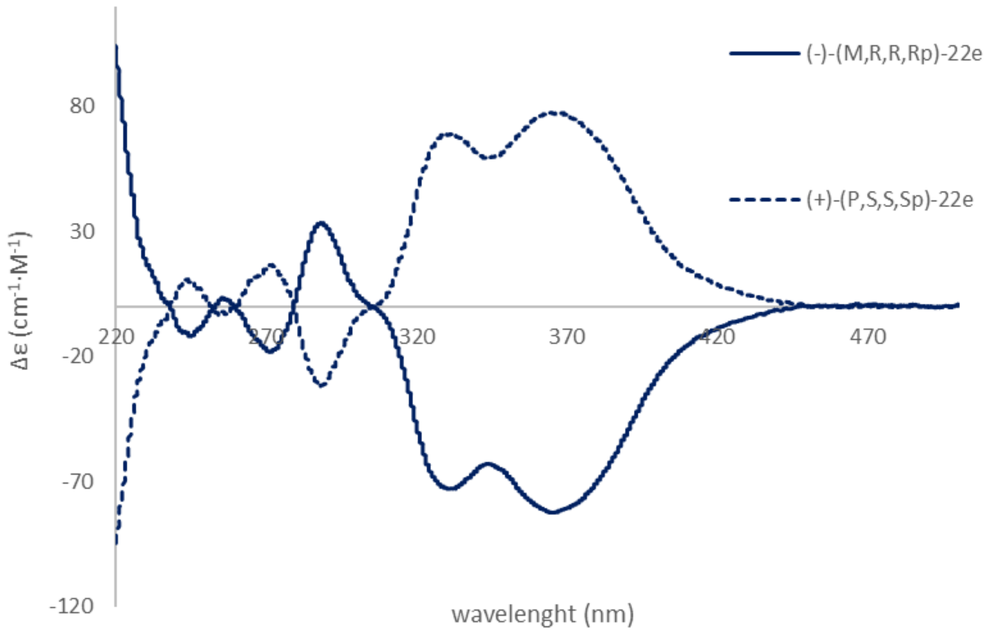

Figure S167: ECD spectra of oxa[6]helicene Rh(I) complexes (-)-(*M,R,R,R*$_p$)-**22e** and (+)-(*P,S,S,S*$_p$)-**22e** (0.1 mM, THF).

Specific rotation $[\alpha]_D$ of the helicene-indene proligands (-)-(*M*,*R*,*R*)-**14a–e**, (-)-(*M*,*R*,*R*)-**20a**,**b**, (-)-(*M*,*R*)-**21**, helicene-indenido Rh^{I} complexes (-)-(*M*,*R*,*R*,R_p)-**22a–e**, (-)-(*M*,*R*,*R*,R_p)-**23a**,**b**, (-)-(*M*,*R*,R_p)-**24**, and helicene-indenido Rh^{III} complexes (-)-(*M*,*R*,*R*,R_p)-**25a**(I)/(Br),**b–e**, (-)-(*M*,*R*,*R*,R_p)-**26a**,**b**, and (-)-(*M*,*R*,R_p)-**27**

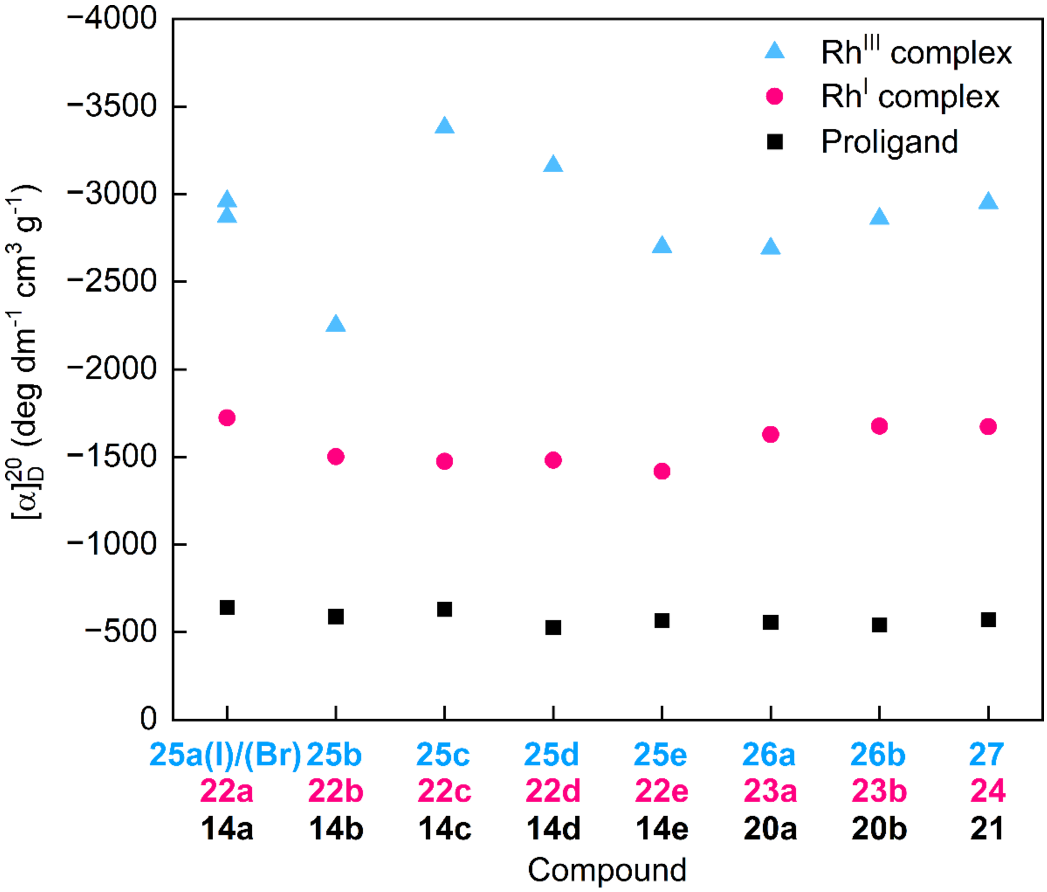

Figure S168: Specific rotation $[\alpha]_D$ of the helicene-indene proligands (-)-(*M*,*R*,*R*)-**14a–e**, (-)-(*M*,*R*,*R*)-**20a**,**b**, (-)-(*M*,*R*)-**21**, helicene-indenido Rh^{I} complexes (-)-(*M*,*R*,*R*,R_p)-**22a–e**, (-)-(*M*,*R*,*R*,R_p)-**23a**,**b**, (-)-(*M*,*R*,R_p)-**24**, and helicene-indenido Rh^{III} complexes (-)-(*M*,*R*,*R*,R_p)-**25a**(I)/(Br),**b–e**, (-)-(*M*,*R*,*R*,R_p)-**26a**,**b**, and (-)-(*M*,*R*,R_p)-**27** (in THF, c 0.004–0.35).

Commented assignment of the NMR spectra of compound (-)-(M,R,R,R_p)-**22c**

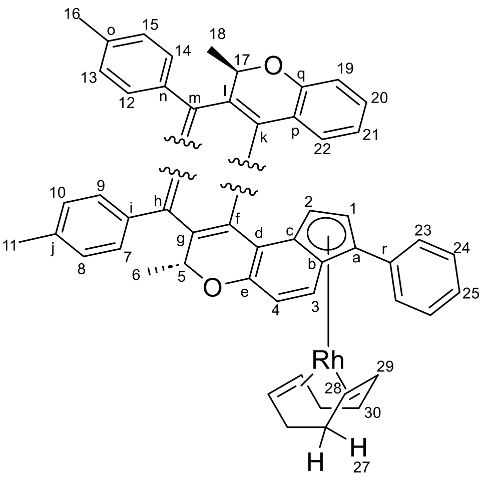

The protons of the phenyl substituted complex (-)-(M,R,R,R_p)-**22c** were assigned based on ^{1}H, ^{13}C APT, HMBC, HSQC, and NOESY measurements as follows:

^{1}H NMR (600 MHz, 298 K, CD_2Cl_2): δ = 7.52 (d, *J* = 8.7 Hz, 1H, CH^{3}), 7.50 – 7.45 (m, 2H, CH^{23}), 7.38 (m, CH^{24}), 7.27 – 7.23 (m, 1H, CH^{25}), 7.20 (dd, *J* = 7.7, 1.9 Hz, 1H, CH^{7}), 7.18 (dd, *J* = 7.7, 1.9 Hz, 1H, CH^{12}), 7.16 – 7.10 (m, 2H, CH^{8} and CH^{13} overlapping), 7.09 (td, *J* = 7.6, 1.6 Hz, 1H, CH^{22}), 7.03 (d, *J* = 8.6 Hz, 1H, CH^{4}), 6.99 – 6.95 (m, 2H, CH^{19} and CH^{20} overlapping), 6.95 – 6.92 (m, 2H, CH^{15} and CH^{10} overlapping), 6.80 (dd, *J* = 7.7, 1.9 Hz, 1H, CH^{14}), 6.77 (dd, *J* = 7.7, 1.9 Hz, 1H, CH^{9}), 6.52 (td, *J* = 7.6, 1.3 Hz, 1H, CH^{21}), 5.96 (dd, *J* = 2.9, 1.7 Hz, 1H, CH^{1}), 5.24 (q, *J* = 6.7 Hz, 1H, CH^{5}), 5.20 (q, *J* = 6.7 Hz, 1H, CH^{17}), 4.31 (d, *J* = 2.9 Hz, 1H, CH^{2}), 3.77 (tt, *J* = 7.9, 3.4 Hz, 2H, CH^{29}), 3.21 (tdd, *J* = 7.9, 4.6, 2.7 Hz, 2H, CH^{28}), 2.29 (s, 3H, CH_3^{11} and CH_3^{16} partially overlapping), 2.28 (s, 3H, CH_3^{11} and CH_3^{16} partially overlapping), 1.91 (td, *J* = 8.2, 5.2 Hz, 4H, CH_2^{30}), 1.70 – 1.62 (m, 2H, CH^{26}), 1.43 – 1.36(m, 2H, CH^{27}), 1.13 (d, *J* = 6.7 Hz, 3H, CH_3^{6}), 0.97 (d, *J* = 6.7 Hz, 3H, CH_3^{18}).

Correspondingly, the carbons in ^{13}C spectrum were also assigned:

^{13}C (151 MHz, 298 K, CD_2Cl_2): δ = 153.21 (C^{e}), 151.43 (C^{q}), 140.29 (C^{g}), 139.34 (C^{l}), 137.39 (C^{h}), 137.00 (C^{m}), 136.79, 136.75 ($C^{j,o}$), 136.39 (C^{r}), 135.94, 135.87 ($C^{n,i}$), 131.70, 131.37 ($CH^{9,14}$), 130.01, 129.84 ($CH^{7,12}$), 129.16 (CH^{24}), 129.06, 129.01, 128.99, 128.99, 128.93 ($CH^{8,10,13,15,22}$), 128.28 (CH^{20}), 127.99 (CH^{23}), 126.77 (CH^{25}), 125.77 (C^{f}), 125.63 (C^{k}), 125.41 (C^{p}), 122.09 (CH^{21}), 120.17 (CH^{3}), 119.06 (CH^{19}), 117.64 (CH^{4}), 115.03 (C^{d}), 113.15 (d, *J* = 2.2 Hz, C^{c}), 107.42 (d, *J* = 1.4 Hz, C^{b}), 94.93 (d, *J* = 3.8 Hz, C^{a}), 92.26 (d, *J* = 4.9 Hz, CH^{1}), 76.09 (d, *J* = 4.4 Hz, CH^{2}), 73.93 (CH^{5}), 73.55 (CH^{17}), 71.51 (d, *J* = 14.0 Hz, 2C, CH^{28}), 70.29 (d, *J* = 13.3 Hz, 2C, CH^{29}), 34.01 (2C, CH_2^{30}), 29.90 (2C, $CH_2^{26/27}$), 21.47, 21.45 ($CH_3^{11,16}$), 19.16 (CH_3^{6}), 18.94 (CH_3^{18}).

From the simple ^{1}H spectra measured at 298 K, we can judge on the free rotation of the three pendent aryl groups attached to the helical backbone. The two tolyl groups have locked conformation and all eight protons of the *ortho* and *meta* positions can be distinguished. On the other hand, the phenyl group attached to the carbon **a** of the indenyl moiety shows free rotation at room temperature with both *ortho* and *meta* protons showing as two multiplets integrating for two protons each.

The protons of the Cp unit are easily identified with proton **2** significantly more shielded which is in accordance with its position pointing more toward the helical cavity. The carbon atoms of the Cp are located upfield from the rest of the aromatic carbons and appear as doublets thanks to the coupling to rhodium.

Considerable amount of structural information can be extracted from the NOESY spectra (**Figure S169**). Most importantly, the NOESY spectra allows for confirming the site of the rhodium coordination to be from the outside of the helicene, rather than from the inside of the helical cavity, as was expected giving a complex with the configuration (M,R,R,R_p). The close up **A** of the NOESY shows a cross-peak between the proton **28** belonging to the C-H group of the COD ligand and protons **6** of the methyl group pointing down toward the metal center. This, together with the absence of NOE between the COD ligand and the upper ring of the helicene, indicates the rhodium atom is coordinated from the less hindered site of the helicene. Similar cross-peaks for cross-relaxation of COD-CH_3 attached to the chiral center can be observed in the NOESY spectra of all Rh(I) complexes. We can also observe the NOE between the alkenyl protons of the COD ligand (**28**, **29**) and the *ortho* protons of the phenyl substituent (**23**) proving that the indenyl substituent is located in close proximity to the catalytic center.

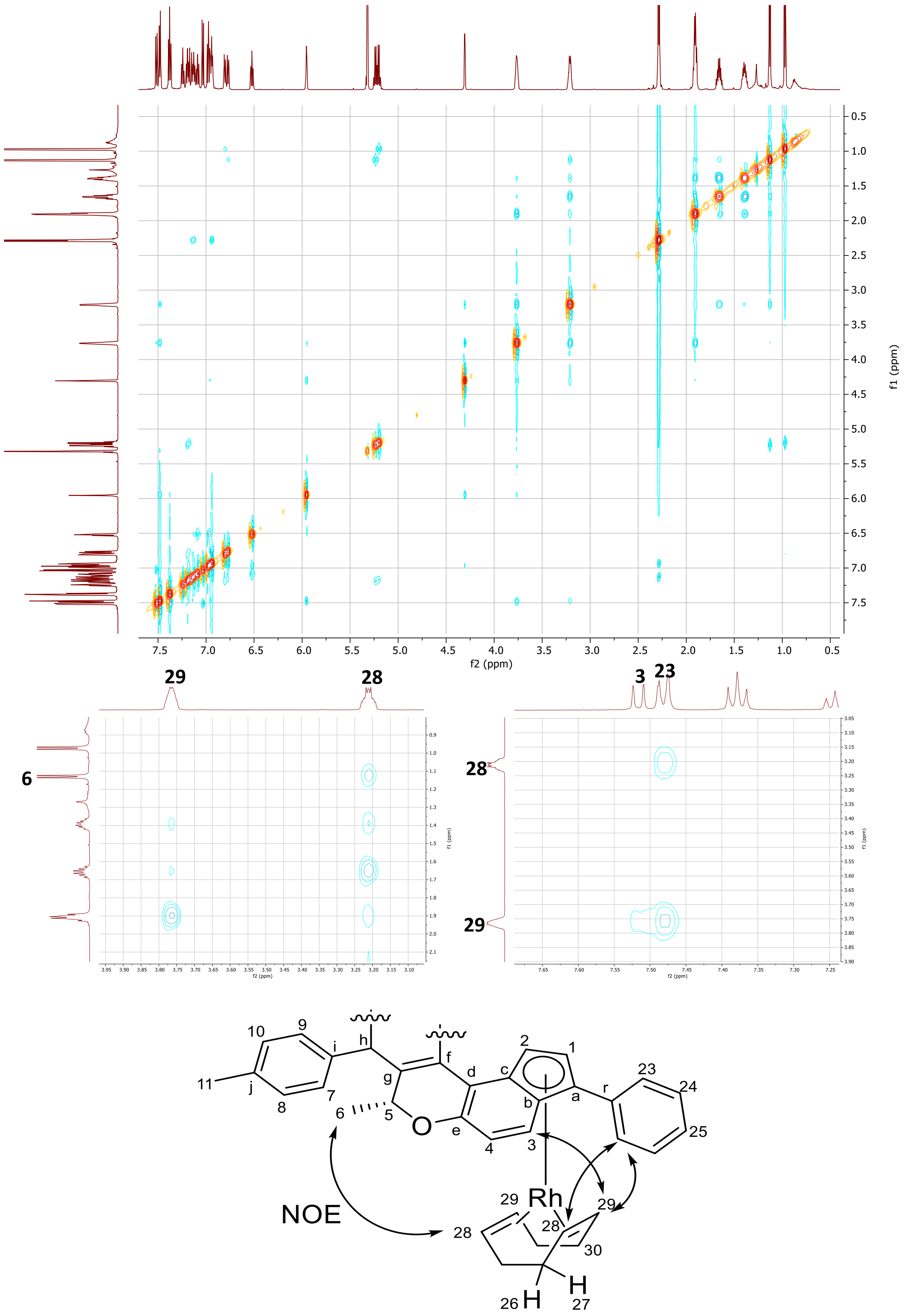

Figure S169: NOESY spectra of complex (-)-(M,R,R,R_p)-**22c**.

Catalysis

The starting substituted benzo[h]quinolines and diazoketones were obtained according to literature procedures as follows: **32b**,[6] **32c**,[6] **32d**,[6] **32e**,[7] **33a**,[8] **33b**,[8] **33c**,[8] and **33d**.[8]

The racemic standards for the HPLC separations were obtained similarly as described below for the asymmetric runs but using $[Cp^*RhCl_2]_2$ as the catalyst.

Atroposelective [4+2] annulative coupling of biphenyl boronic acid **29**

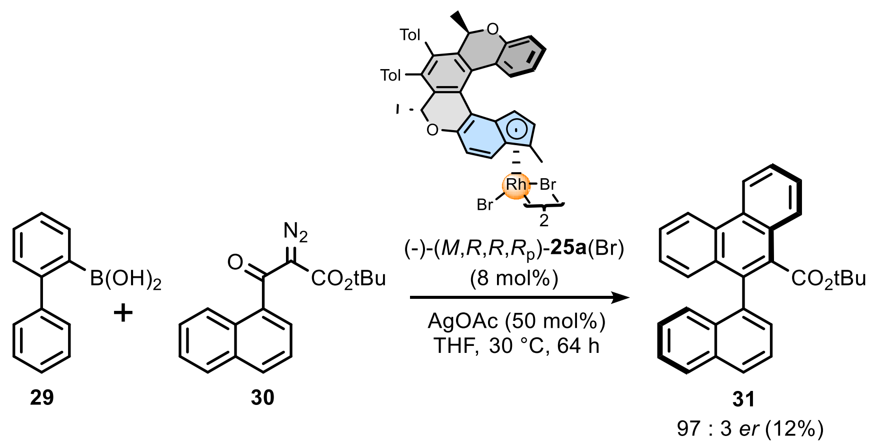

Performed according to the literature procedure,[9] racemic standard was prepared by the same procedure using $[Cp^*RhCl_2]_2$ as the catalyst.

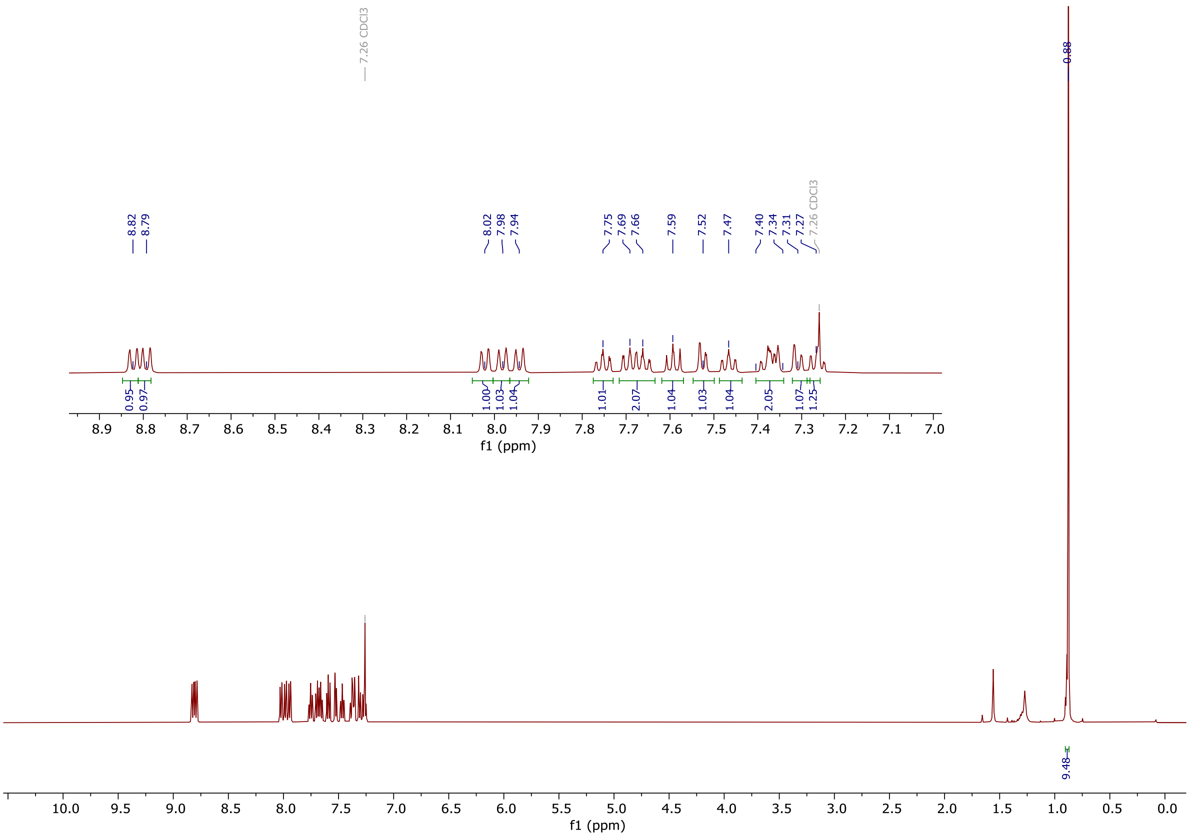

Figure S170: **^{1}H NMR** spectrum of the racemic standard of **31**.

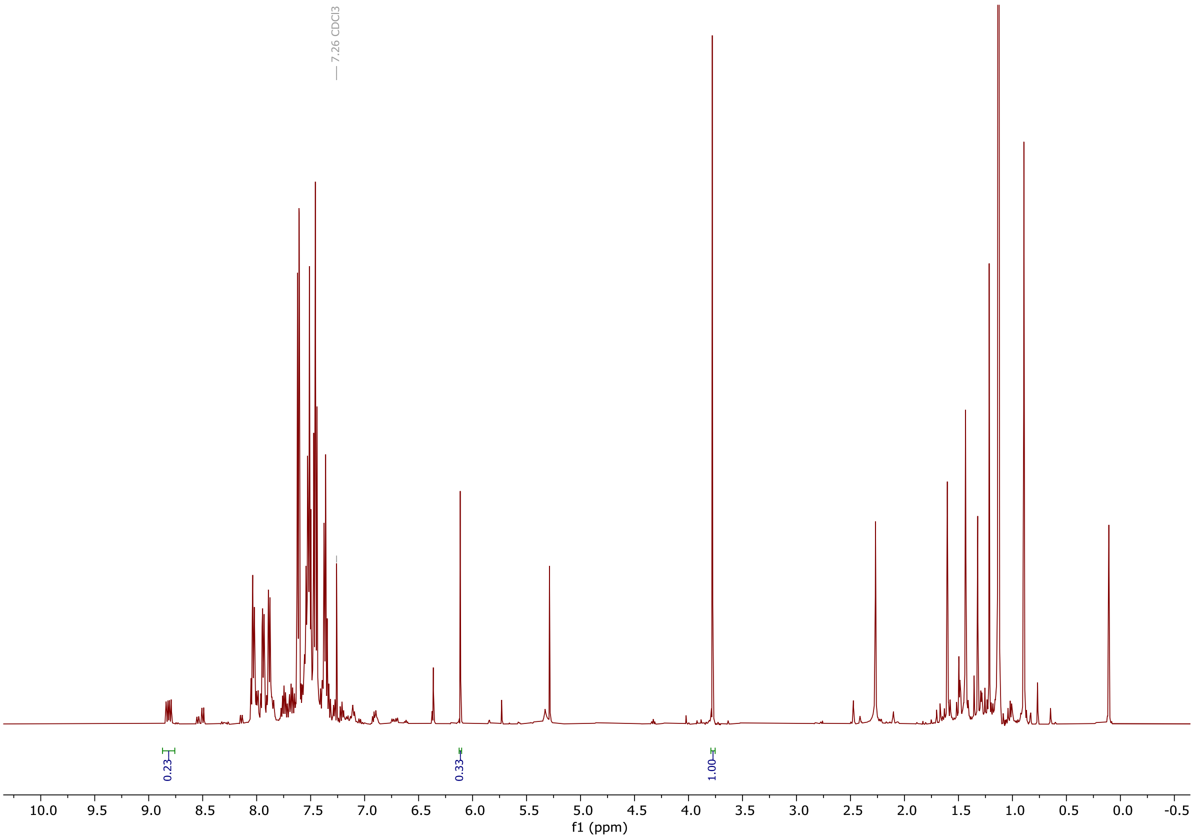

Figure S171: **^{1}H NMR** spectrum of the reaction mixture catalyzed by (-)-(*M,R,R,R$_p$*)-**25a**(Br)**,** internal standard 1,3,5-trimethoxybenzene (0.11 equiv.).

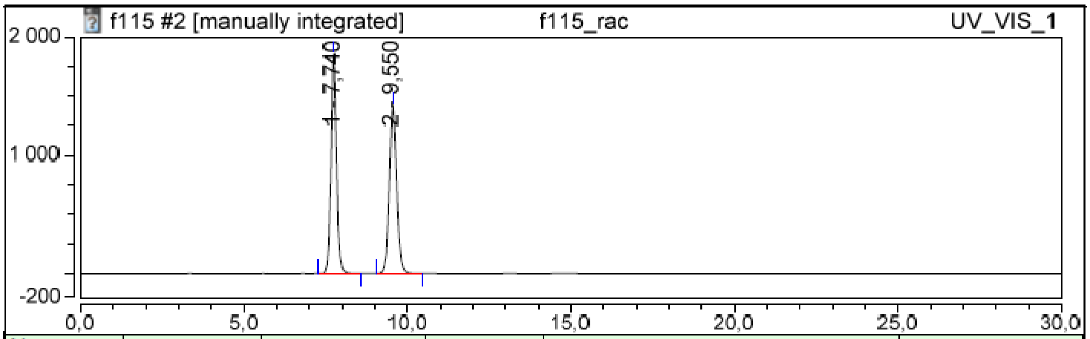

No.	Peak Name	Retention Time<br>min	Area<br>mAU*min	Height<br>mAU	elative Area<br>%	elative Heig<br>%	Amount<br>n.a.
1		7,740	356,746	1849,282	49,93	56,67	n.a.
2		9,550	357,746	1414,085	50,07	43,33	n.a.
**Total:**			**714,492**	**3263,367**	**100,00**	**100,00**	

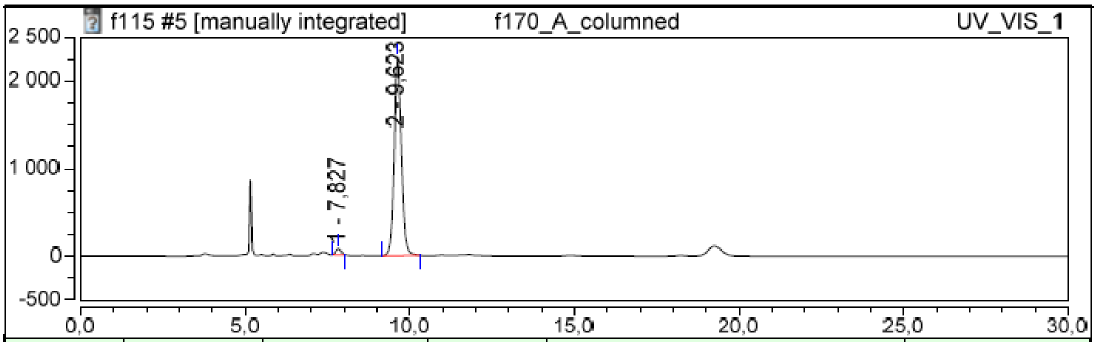

No.	Peak Name	Retention Time<br>min	Area<br>mAU*min	Height<br>mAU	elative Area<br>%	elative Heig<br>%	Amount<br>n.a.
1		7,827	12,647	73,239	2,22	3,12	n.a.
2		9,623	557,348	2271,025	97,78	96,88	n.a.
**Total:**			**569,994**	**2344,264**	**100,00**	**100,00**	

Figure S172: Separation of racemic **31** (top) and the product of catalysis using (-)-(*M,R,R,R$_p$*)-**25a**(Br) as the catalyst.

C-H activation of benzo[*h*]quinolines **32** - Catalyst optimization

General procedure for catalyst optimization **GP5**

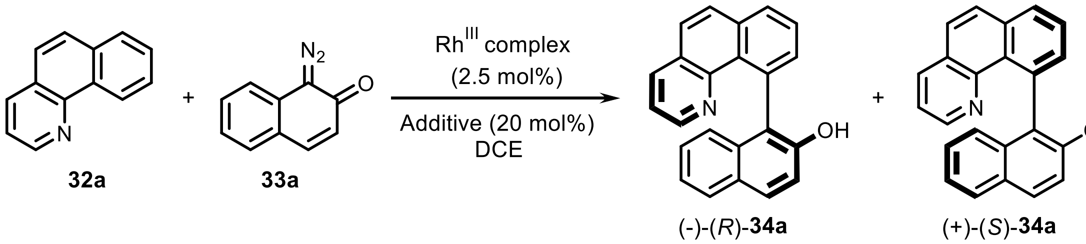

All reactions were performed as two parallel runs under the same conditions. Benzo[*h*]quinoline **32a** (17.9 mg, 0.1 mmol, 1.0 equiv.) and $AgSbF_6$ (6.9 mg, 0.02 mmol, 20 mol%) were weighed under air into a 10 mL crimp top vial containing a small oval stir bar. Then the catalyst (0.0025 mmol, 2.5 mol%) was added as a solution in dry dichloroethane (1 mL) and the mixture was inserted into a preheated block heater set to 30 °C for 15 min. Then diazonaphthalaquinone **33a** (25.5 mg, 0.15 mmol, 1.5 equiv.) was added as a solid, the vial was crimped, and allowed to stir for 24 h. The experiments at lower temperature (**Table S1**, **entry 5-8**) were performed correspondingly using a precooled isopropanol bath.

Fifteen minutes before the end of the reaction period, a solution of 1,3,5-trimethoxybenzene and tetra-*n*-butylammonium iodide in dichloromethane (1 mL, 1,3,5-trimethoxybenzene 1.87 mg/mL, 0.011 mmol, 0.11 equiv.; *n*-Bu_4NI 18.5 mg/mL, 0.05 mmol, 0.5 equiv.) was added. After 24 h, the reaction vial was opened and its content was filtered through a layer of diatomaceous earth with EtOAc (30 mL). The volatiles were removed under reduced pressure and an NMR sample was prepared by dissolving the residue in $CDCl_3$. A ^1H NMR spectrum was measured on a 500 MHz spectrometer using a standard proton sequence by adjusting the d1 to 60 s and the number of scans to 1.

The residue was then purified by column chromatography on silica gel (heptane:EtOAc 5:1). The products were collected and subjected to HPLC separations to establish the enantiomeric excess. HPLC conditions: Lux Cellulose-1 column, hexane/*i*-PrOH, 85:15 v/v, flow rate 1 mL/min, λ = 254 nm, 25 °C. t_R = 11.14 min, t_R = 14.03 min.

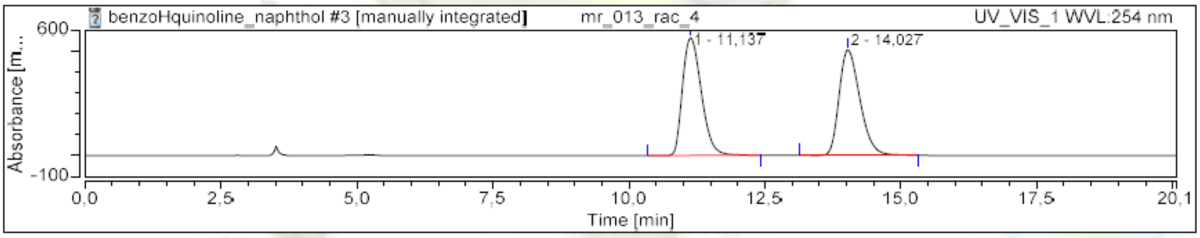

Integration Results							
No.	Peak Name	Retention Time min	Area mAU*min	Height mAU	Relative Area %	Relative Height %	Amount n.a.
1		11,137	225,126	563,458	50,06	52,66	n.a.
2		14,027	224,580	506,541	49,94	47,34	n.a.
Total:			449,706	1069,999	100,00	100,00	

Figure S173: Separation of racemic **34a** into enantiomers.

Table S1: Catalytic table comparing different catalysts

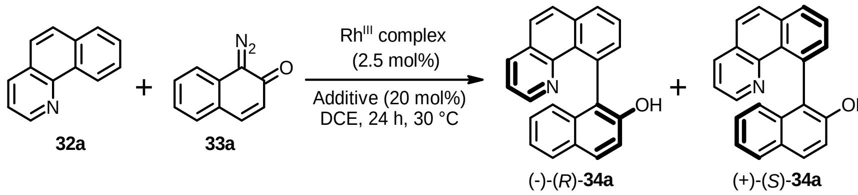

Rh^{III} complexes:

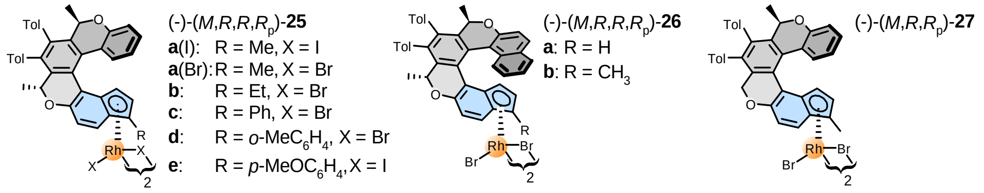

Entry	Catalyst	Conversion (%)	NMR yield (%)	*er*
**1**	(-)-(*M*,*R*,*R*,*R$_p$*)-**25a**(Br)	90	76	86 : 14
**2**	(-)-(*M*,*R*,*R*,*R$_p$*)-**25a**(Br)	91	74	87 : 13
**3**	(-)-(*M*,*R*,*R*,*R$_p$*)-**25a**(I)	49	49	86 : 14
**4**	(-)-(*M*,*R*,*R*,*R$_p$*)-**25a**(I)	51	51	86 : 14
**5**[a]	(-)-(*M*,*R*,*R*,*R$_p$*)-**25a**(Br)	100	93	90 : 10
**6**[a]	(-)-(*M*,*R*,*R*,*R$_p$*)-**25a**(Br)	100	95	90 : 10
**7**[b]	(-)-(*M*,*R*,*R*,*R$_p$*)-**25a**(Br)	98	98	91 : 9
**8**[b]	(-)-(*M*,*R*,*R*,*R$_p$*)-**25a**(Br)	99	99	90 : 10
**9**	(-)-(*M*,*R*,*R*,*R$_p$*)-**25b**	84	79	79 : 21
**10**	(-)-(*M*,*R*,*R*,*R$_p$*)-**25b**	86	79	78 : 22
**11**	(-)-(*M*,*R*,*R*,*R$_p$*)-**25c**	33	32	58 : 42
**12**	(-)-(*M*,*R*,*R*,*R$_p$*)-**25c**	31	31	59 : 41
**13**	(-)-(*M*,*R*,*R*,*R$_p$*)-**25d**	24	25	25 : 75
**14**	(-)-(*M*,*R*,*R*,*R$_p$*)-**25d**	25	25	24 : 76
**15**	(-)-(*M*,*R*,*R*,*R$_p$*)-**25e**	10	8	71 : 29
**16**	(-)-(*M*,*R*,*R*,*R$_p$*)-**25e**	8	7	71 : 29
**17**	(-)-(*M*,*R*,*R*,*R$_p$*)-**26a**	100	96	76 : 24
**18**	(-)-(*M*,*R*,*R*,*R$_p$*)-**26a**	100	95	76 : 24
**19**	(-)-(*M*,*R*,*R*,*R$_p$*)-**26b**	77	77	77 : 23
**20**	(-)-(*M*,*R*,*R*,*R$_p$*)-**26b**	80	75	77 : 23
**21**	(-)-(*M*,*R*,*R$_p$*)-**27**	75	59	72 : 28
**22**	(-)-(*M*,*R*,*R$_p$*)-**27**	75	58	73 : 27

Performed according to **GP5**; [a] At 0 °C; [b] at -20 °C for 48 h.

Table S1, Entry 1 & 2

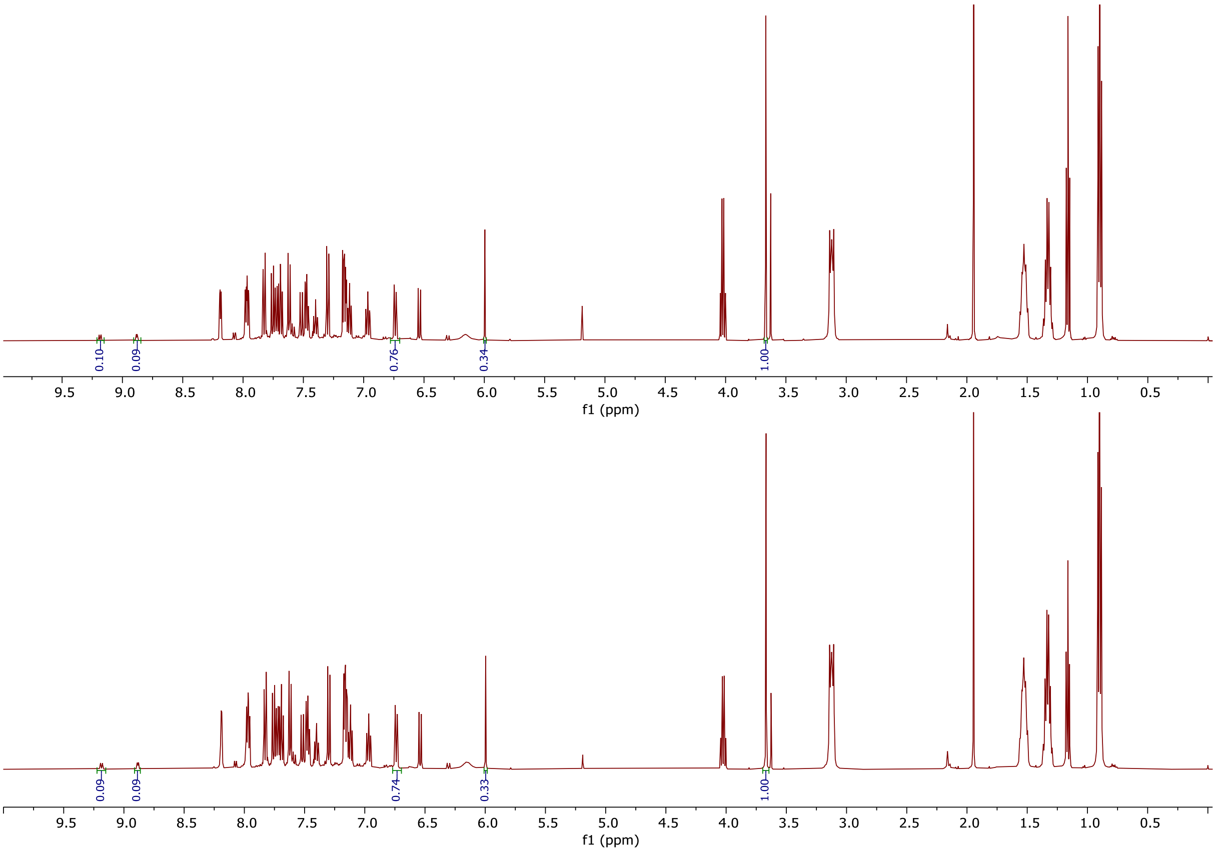

Figure S174: ^{1}H NMR of the crude reaction mixtures **Table S1**, **Entry 1** & **2** (500 MHz, 298 K, $CDCl_3$).

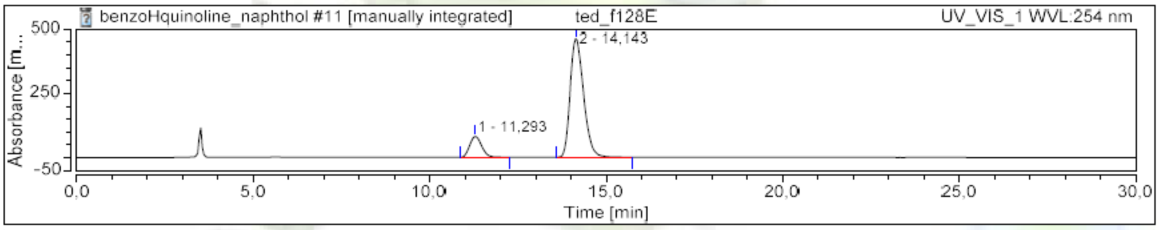

Integration Results							
No.	Peak Name	Retention Time min	Area mAU*min	Height mAU	Relative Area %	Relative Height %	Amount n.a.
1		11,293	31,921	80,669	13,51	14,83	n.a.
2		14,143	204,345	463,350	86,49	85,17	n.a.
Total:			236,265	544,019	100,00	100,00	

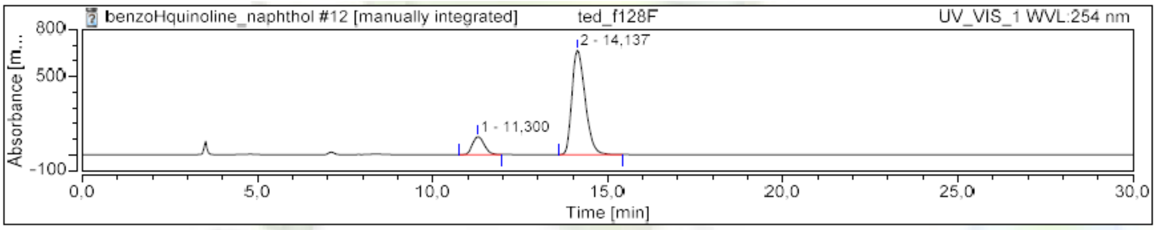

Integration Results							
No.	Peak Name	Retention Time min	Area mAU*min	Height mAU	Relative Area %	Relative Height %	Amount n.a.
1		11,300	44,939	113,893	13,30	14,59	n.a.
2		14,137	293,009	666,489	86,70	85,41	n.a.
Total:			337,948	780,382	100,00	100,00	

Figure S175: HPLC separation of the isolated product **Table S1**, **Entry 1** & **2**.

Table S1, Entry 3 & 4

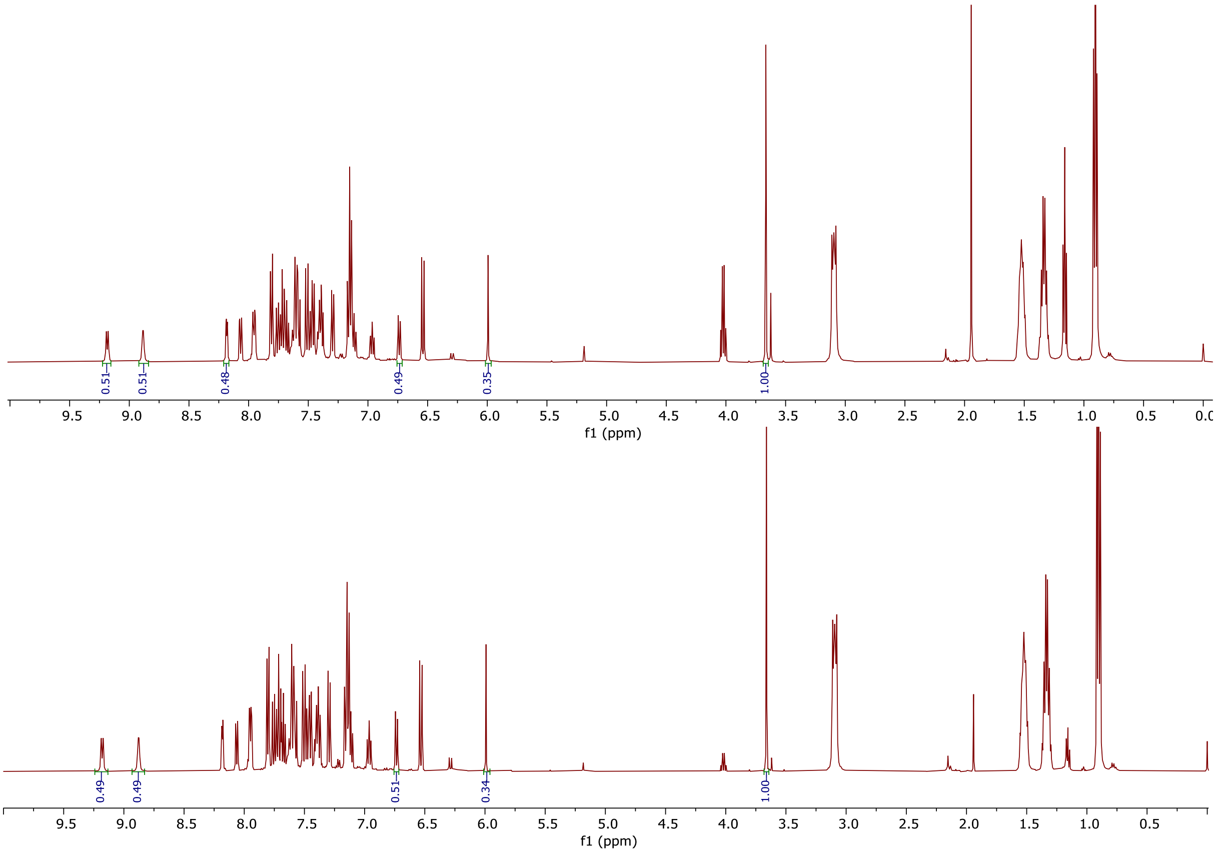

Figure S176: ^{1}H NMR of the crude reaction mixtures **Table S1**, **Entry 3** & **4** (500 MHz, 298 K, $CDCl_3$).

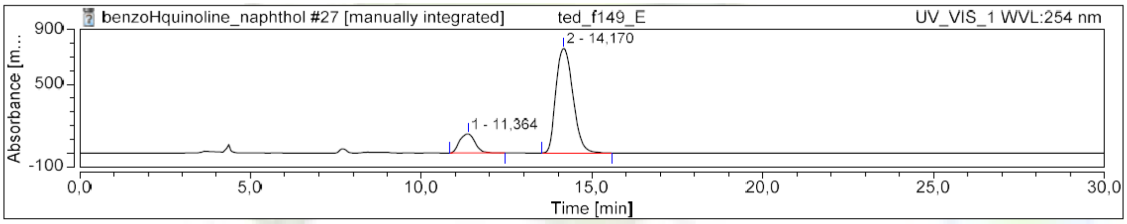

Integration Results							
No.	Peak Name	Retention Time min	Area mAU*min	Height mAU	Relative Area %	Relative Height %	Amount n.a.
1		11,364	71,977	138,853	14,07	15,40	n.a.
2		14,170	439,577	762,731	85,93	84,60	n.a.
**Total:**			**511,554**	**901,583**	**100,00**	**100,00**	

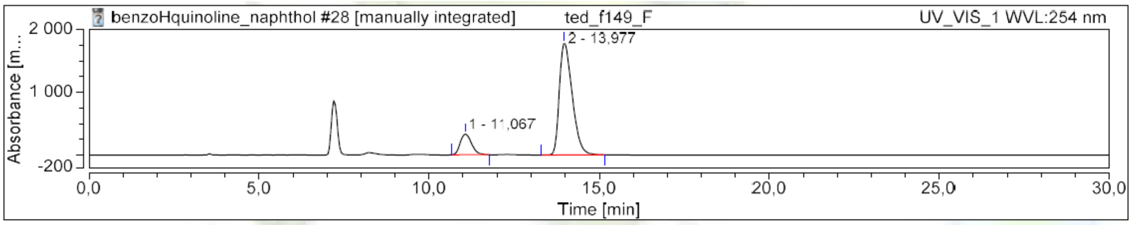

Integration Results							
No.	Peak Name	Retention Time min	Area mAU*min	Height mAU	Relative Area %	Relative Height %	Amount n.a.
1		11,067	126,801	328,959	13,99	15,55	n.a.
2		13,977	779,577	1785,906	86,01	84,45	n.a.
**Total:**			**906,378**	**2114,865**	**100,00**	**100,00**	

Figure S177: HPLC separation of the isolated product **Table S1**, **Entry 3** & **4**.

Table S1, Entry 5 & 6

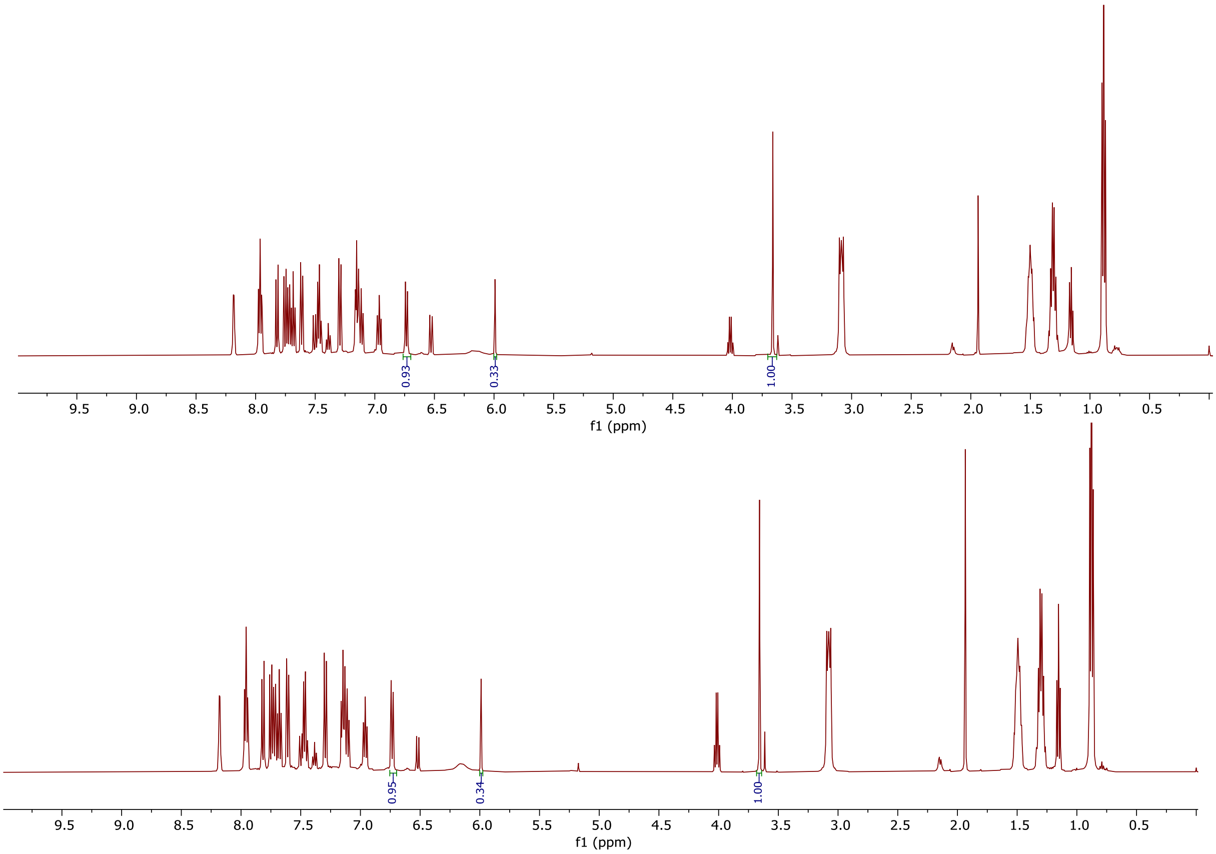

Figure S178: ^{1}H NMR of the crude reaction mixtures **Table S1**, **Entry 5** & **6** (500 MHz, 298 K, $CDCl_3$).

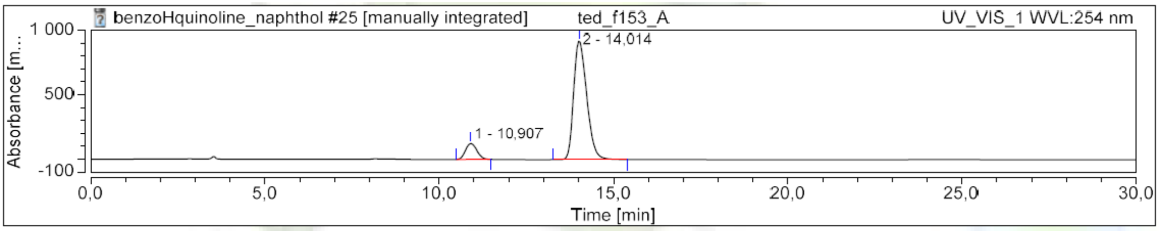

Integration Results							
No.	Peak Name	Retention Time<br>min	Area<br>mAU*min	Height<br>mAU	Relative Area<br>%	Relative Height<br>%	Amount<br>n.a.
1		10,907	46,291	122,986	10,36	11,77	n.a.
2		14,014	400,496	921,517	89,64	88,23	n.a.
**Total:**			**446,786**	**1044,503**	**100,00**	**100,00**	

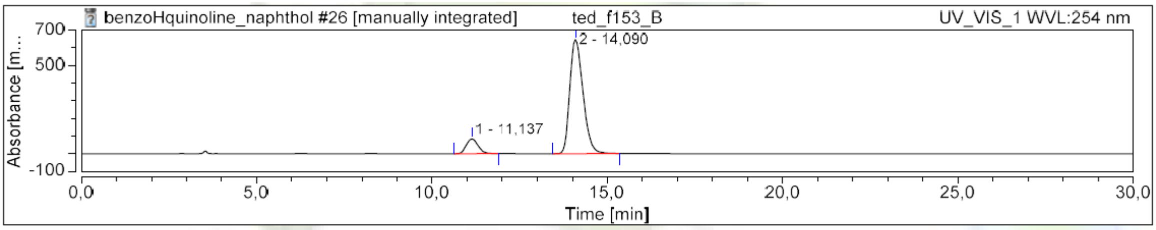

Integration Results							
No.	Peak Name	Retention Time<br>min	Area<br>mAU*min	Height<br>mAU	Relative Area<br>%	Relative Height<br>%	Amount<br>n.a.
1		11,137	32,526	82,962	10,34	11,37	n.a.
2		14,090	282,137	646,663	89,66	88,63	n.a.
**Total:**			**314,663**	**729,625**	**100,00**	**100,00**	

Figure S179: HPLC separation of the isolated product **Table S1**, **Entry 5** & **6**.

Table S1, Entry 7 & 8

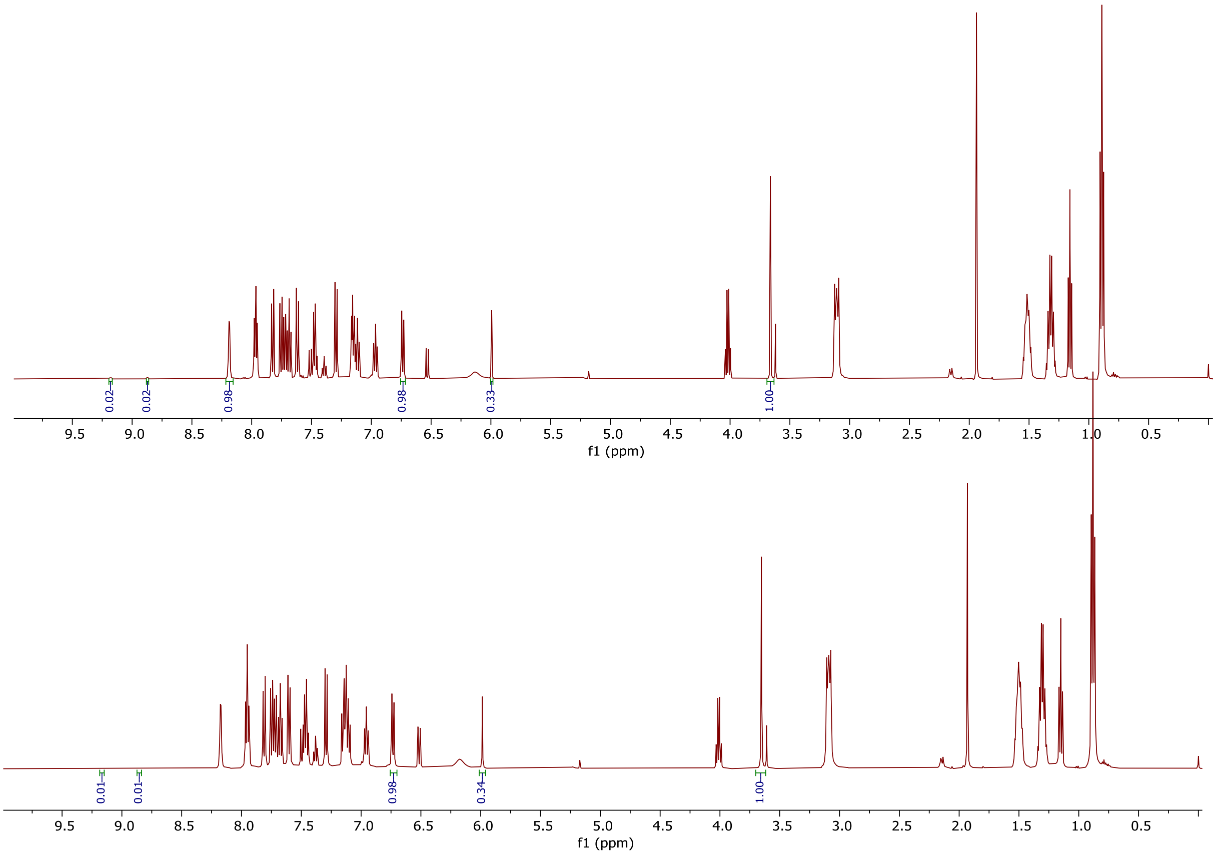

Figure S180: ^{1}H NMR of the crude reaction mixtures **Table S1**, **Entry 7** & **8** (500 MHz, 298 K, $CDCl_3$).

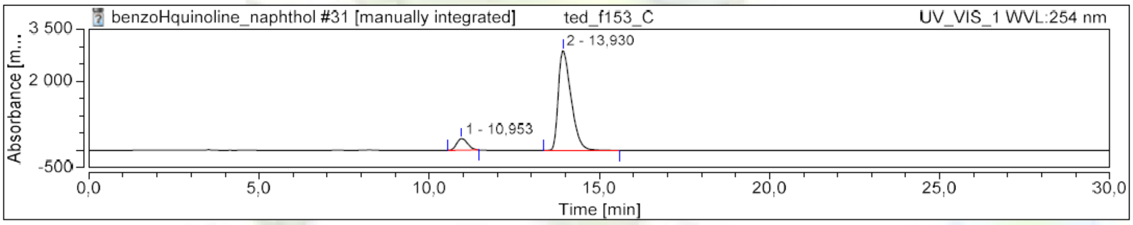

Integration Results							
No.	Peak Name	Retention Time min	Area mAU*min	Height mAU	Relative Area %	Relative Height %	Amount n.a.
1		10,953	124,018	333,965	8,98	10,33	n.a.
2		13,930	1257,352	2897,793	91,02	89,67	n.a.
**Total:**			**1381,370**	**3231,758**	**100,00**	**100,00**	

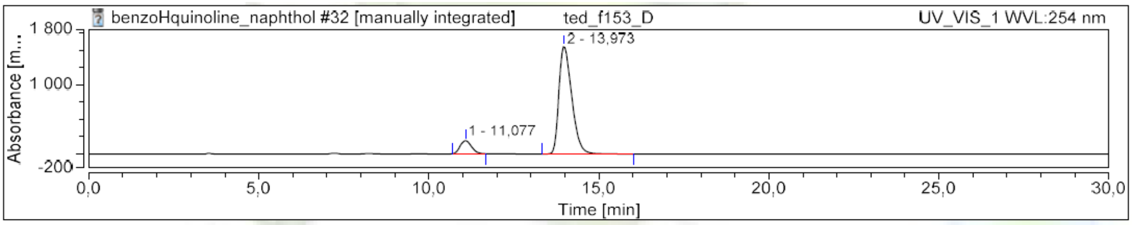

Integration Results							
No.	Peak Name	Retention Time min	Area mAU*min	Height mAU	Relative Area %	Relative Height %	Amount n.a.
1		11,077	73,139	190,134	9,66	10,85	n.a.
2		13,973	683,802	1562,178	90,34	89,15	n.a.
**Total:**			**756,940**	**1752,312**	**100,00**	**100,00**	

Figure S181: HPLC separation of the isolated product **Table S1**, **Entry 7** & **8**.

Table S1, Entry 9 & 10

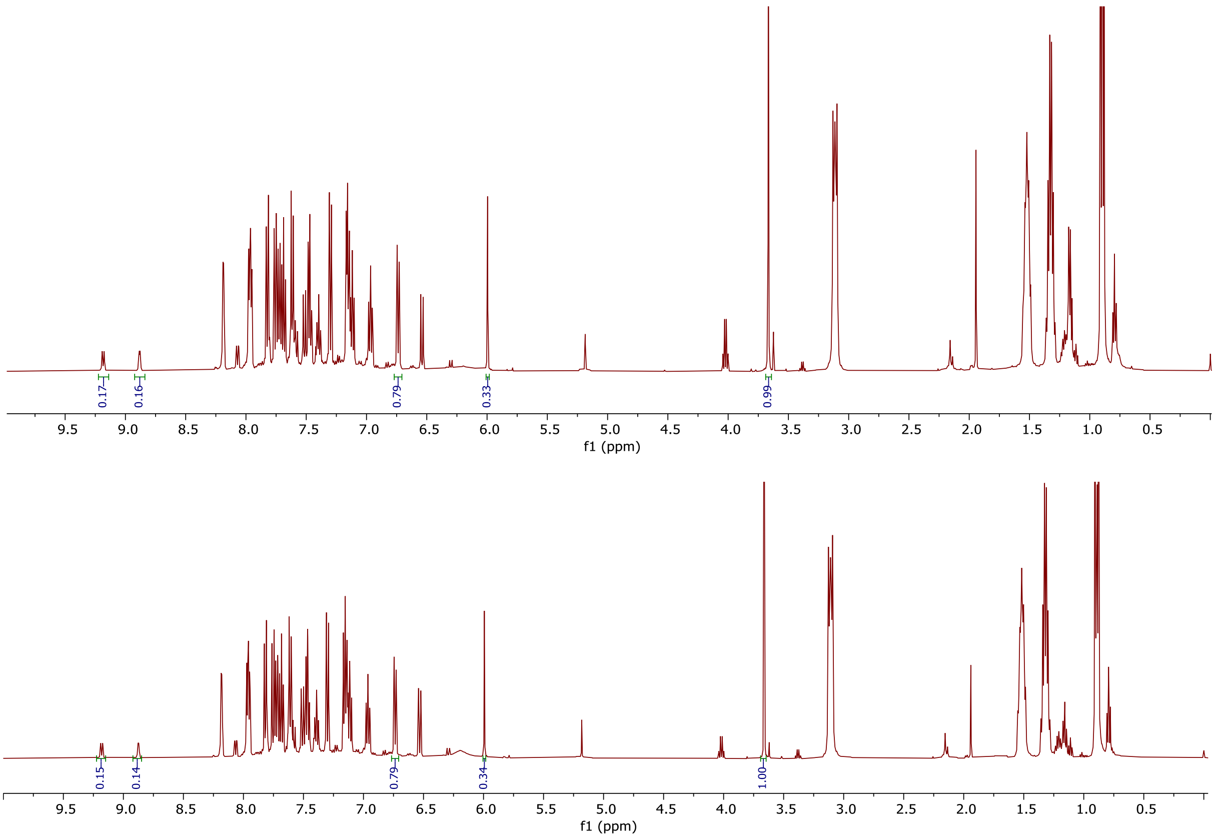

Figure S182: ^{1}H NMR of the crude reaction mixtures **Table S1**, **Entry 9** & **10** (500 MHz, 298 K, $CDCl_3$).

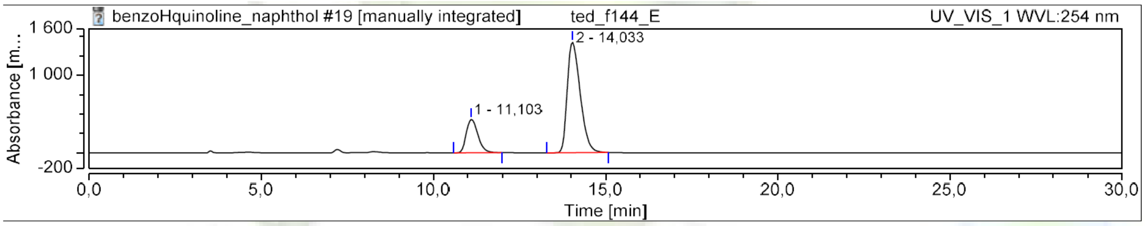

Integration Results							
No.	Peak Name	Retention Time min	Area mAU*min	Height mAU	Relative Area %	Relative Height %	Amount n.a.
1		11,103	169,094	430,083	21,38	23,17	n.a.
2		14,033	621,836	1425,776	78,62	76,83	n.a.
Total:			**790,930**	**1855,858**	**100,00**	**100,00**	

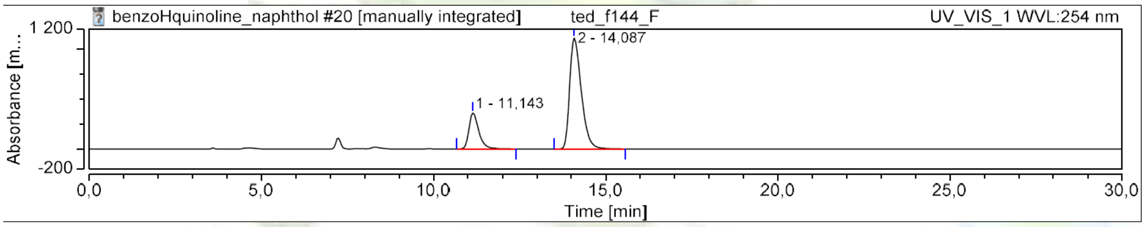

Integration Results							
No.	Peak Name	Retention Time min	Area mAU*min	Height mAU	Relative Area %	Relative Height %	Amount n.a.
1		11,143	120,058	360,887	21,64	24,53	n.a.
2		14,087	434,765	1110,419	78,36	75,47	n.a.
Total:			**554,823**	**1471,307**	**100,00**	**100,00**	

Figure S183: HPLC separation of the isolated product **Table S1**, **Entry 9** & **10**.

Table S1, Entry 11 & 12

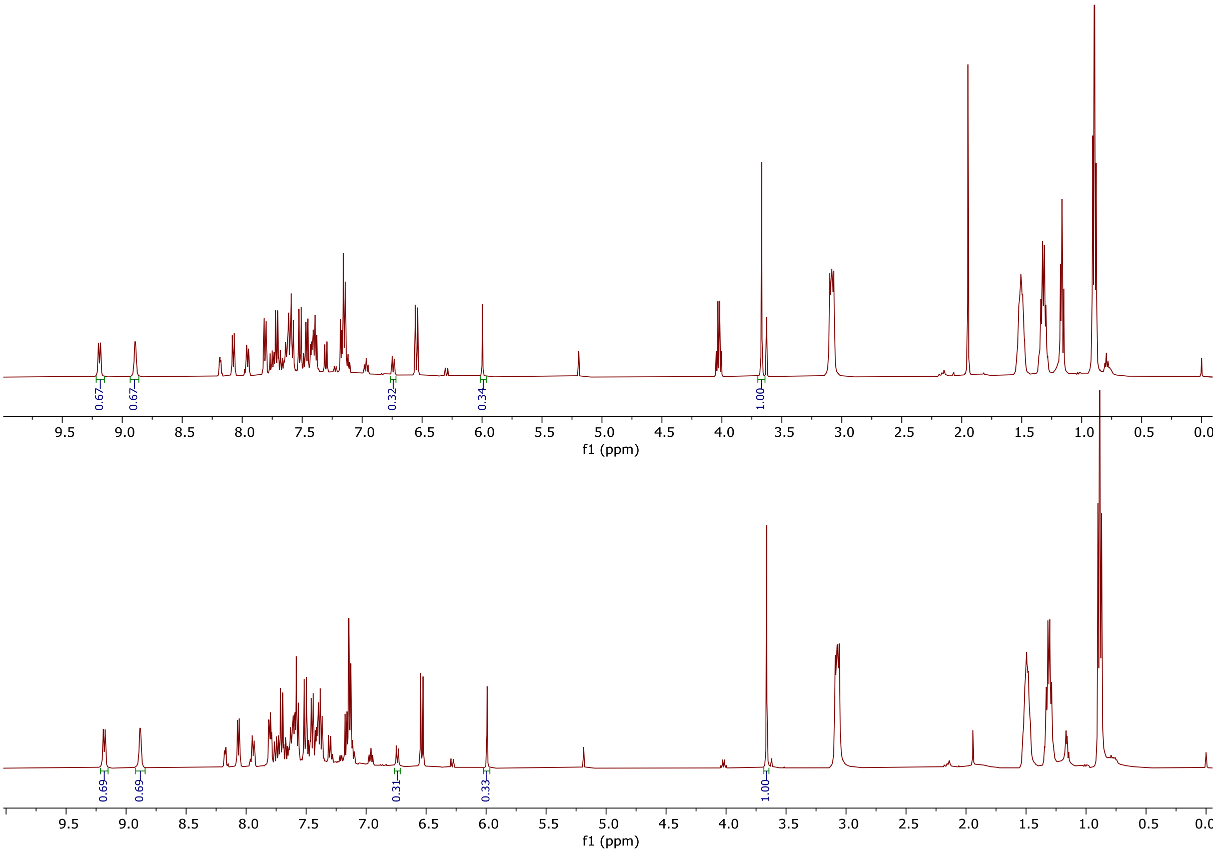

Figure S184: ^{1}H NMR of the crude reaction mixtures **Table S1**, **Entry 11** & **12** (500 MHz, 298 K, $CDCl_3$).

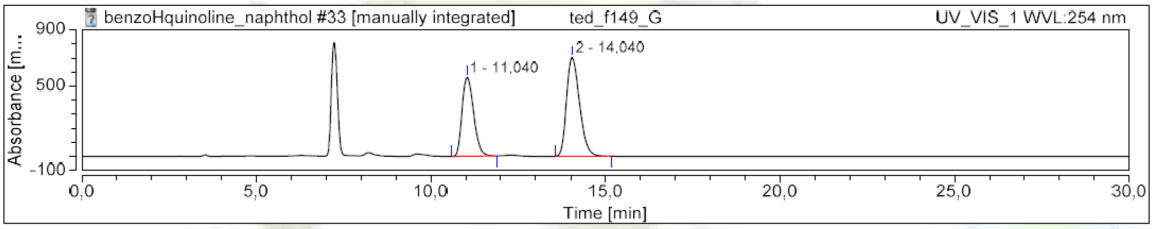

Integration Results

No.	Peak Name	Retention Time min	Area mAU*min	Height mAU	Relative Area %	Relative Height %	Amount n.a.
1		11,040	215,987	563,283	41,58	44,40	n.a.
2		14,040	303,419	705,367	58,42	55,60	n.a.
Total:			519,405	1268,650	100,00	100,00	

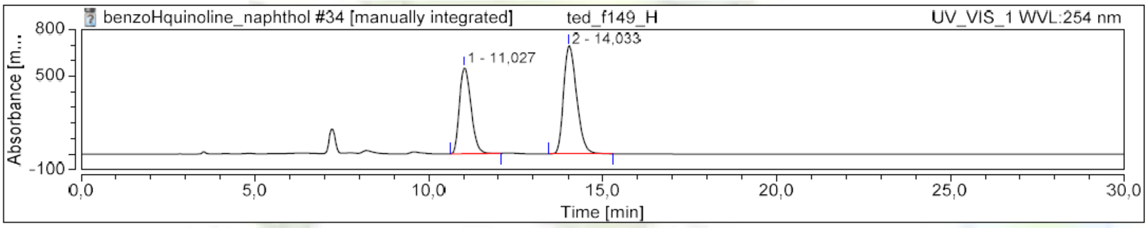

Integration Results

No.	Peak Name	Retention Time min	Area mAU*min	Height mAU	Relative Area %	Relative Height %	Amount n.a.
1		11,027	212,711	552,440	41,27	44,23	n.a.
2		14,033	302,695	696,612	58,73	55,77	n.a.
Total:			515,405	1249,052	100,00	100,00	

Figure S185: HPLC separation of the isolated product **Table S1**, **Entry 11** & **12**.

Table S1, Entry 13 & 14

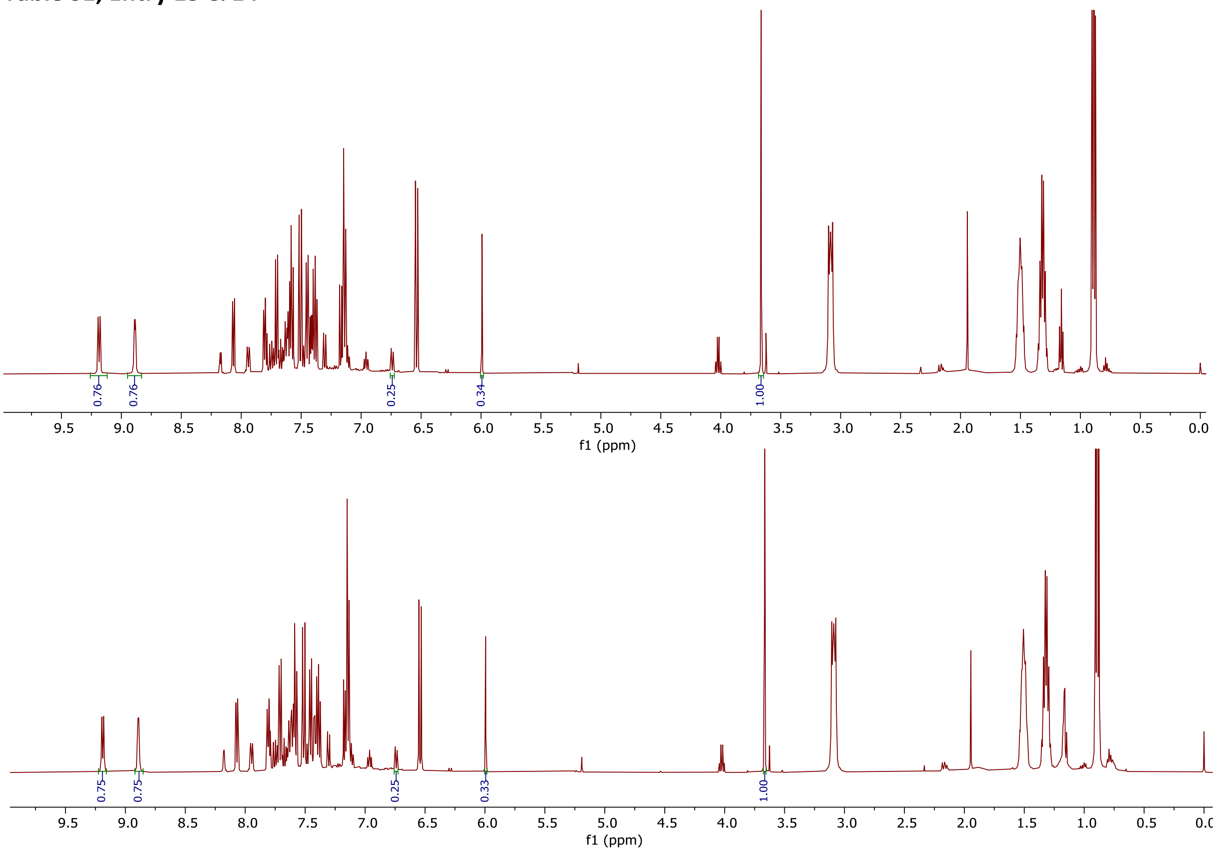

Figure S186: ^{1}H NMR of the crude reaction mixtures **Table S1**, **Entry 13** & **14** (500 MHz, 298 K, $CDCl_3$).

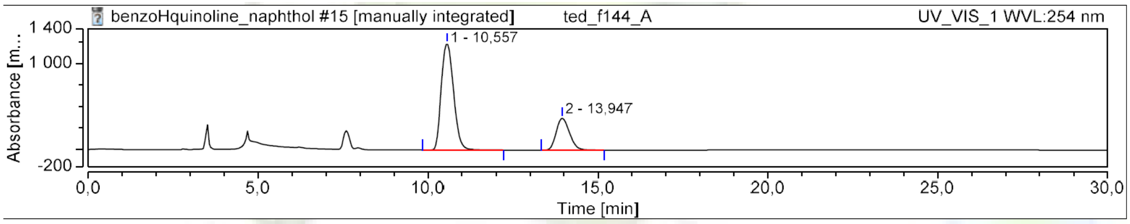

Integration Results							
No.	Peak Name	Retention Time min	Area mAU*min	Height mAU	Relative Area %	Relative Height %	Amount n.a.
1		10,557	517,942	1234,312	75,48	76,94	n.a.
2		13,947	168,270	369,957	24,52	23,06	n.a.
Total:			686,212	1604,270	100,00	100,00	

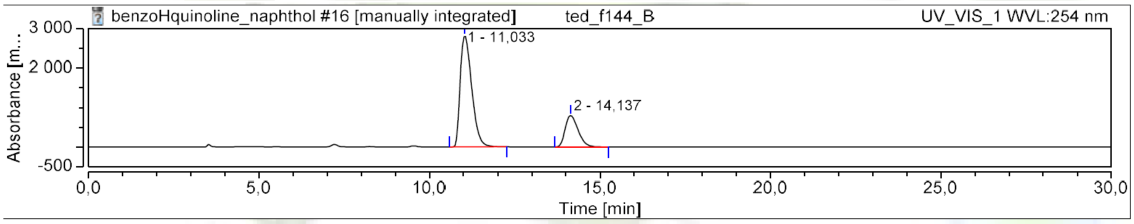

Integration Results							
No.	Peak Name	Retention Time min	Area mAU*min	Height mAU	Relative Area %	Relative Height %	Amount n.a.
1		11,033	1102,246	2820,396	75,82	77,96	n.a.
2		14,137	351,510	797,192	24,18	22,04	n.a.
Total:			1453,756	3617,588	100,00	100,00	

Figure S187: HPLC separation of the isolated product **Table S1**, **Entry 13** & **14**.

Table S1, Entry 15 & 16

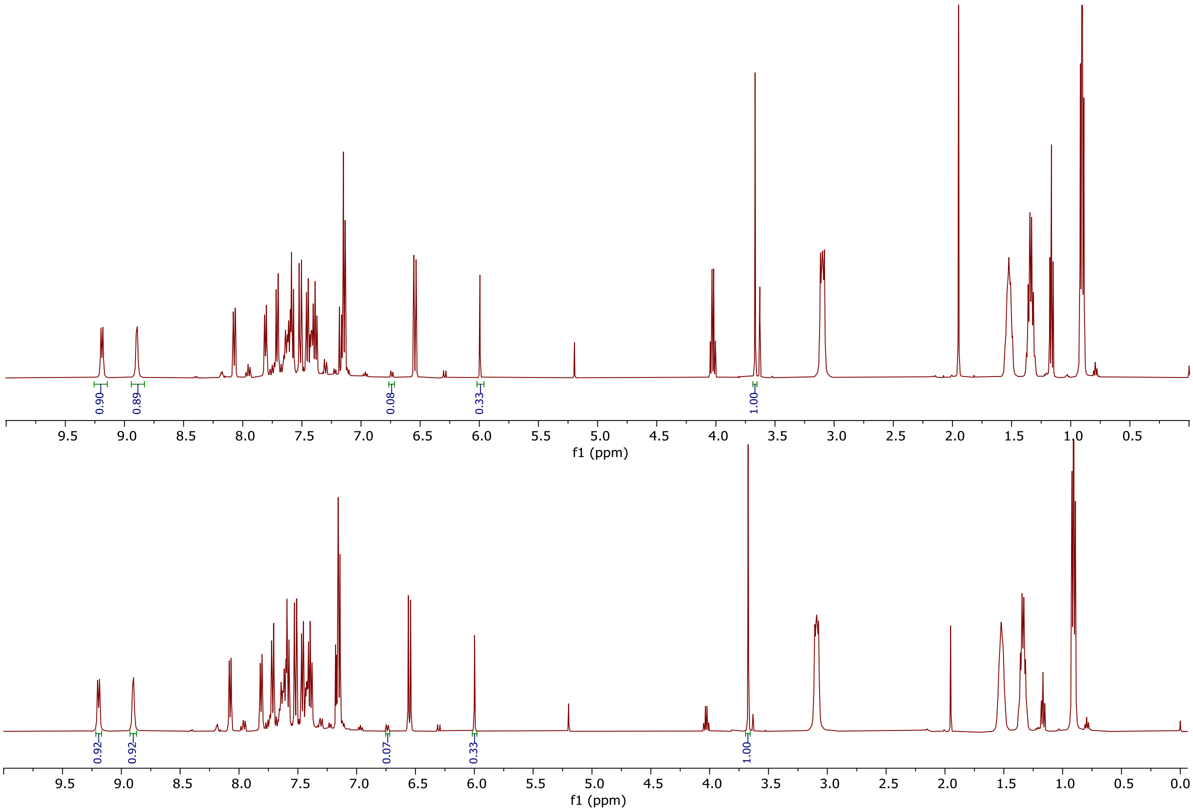

Figure S188: ^{1}H NMR of the crude reaction mixtures **Table S1**, **Entry 15** & **16** (500 MHz, 298 K, $CDCl_3$).

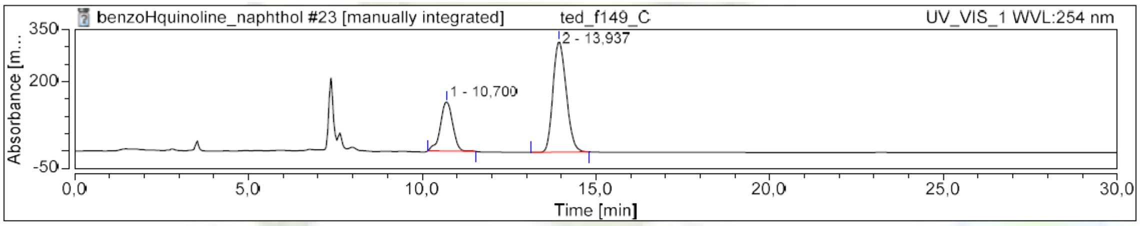

Integration Results

No.	Peak Name	Retention Time min	Area mAU*min	Height mAU	Relative Area %	Relative Height %	Amount n.a.
1		10,700	57,922	142,021	29,14	30,79	n.a.
2		13,937	140,830	319,223	70,86	69,21	n.a.
Total:			198,752	461,244	100,00	100,00	

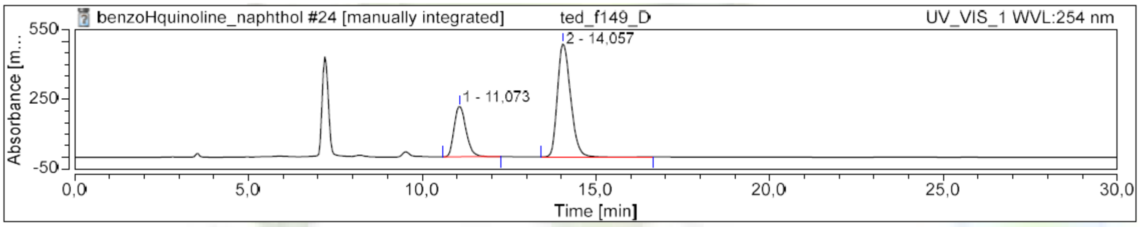

Integration Results

No.	Peak Name	Retention Time min	Area mAU*min	Height mAU	Relative Area %	Relative Height %	Amount n.a.
1		11,073	86,277	217,667	28,91	30,85	n.a.
2		14,057	212,147	487,791	71,09	69,15	n.a.
Total:			298,425	705,458	100,00	100,00	

Figure S189: HPLC separation of the isolated product **Table S1**, **Entry 15** & **16**.

Table S1, Entry 17 & 18

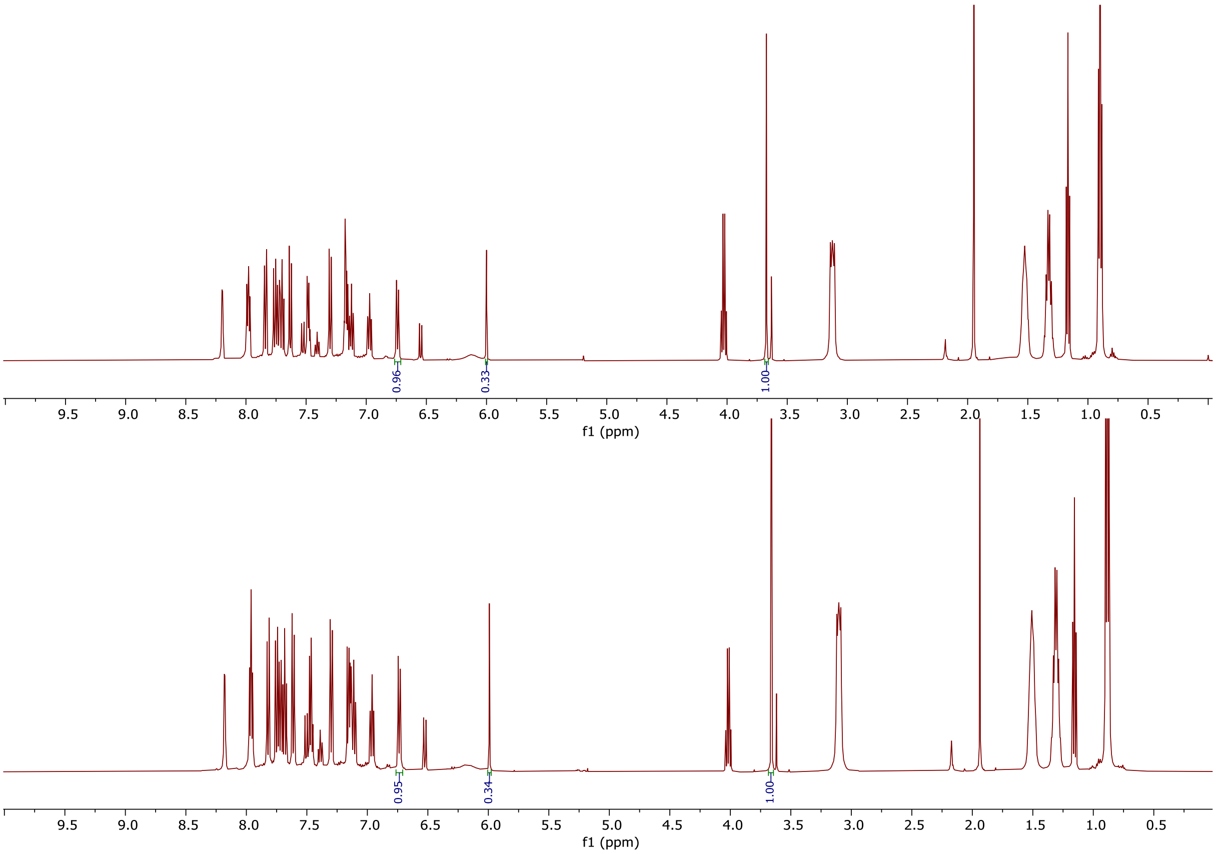

Figure S190: ^{1}H NMR of the crude reaction mixtures **Table S1, Entry 17** & **18** (500 MHz, 298 K, $CDCl_3$).

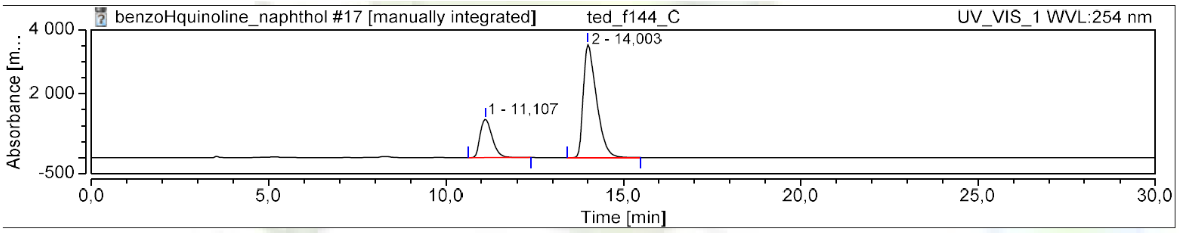

**Integration Results**							
No.	Peak Name	Retention Time min	Area mAU*min	Height mAU	Relative Area %	Relative Height %	Amount n.a.
1		11,107	473,291	1197,839	23,66	25,24	n.a.
2		14,003	1527,213	3548,843	76,34	74,76	n.a.
Total:			**2000,504**	**4746,682**	**100,00**	**100,00**	

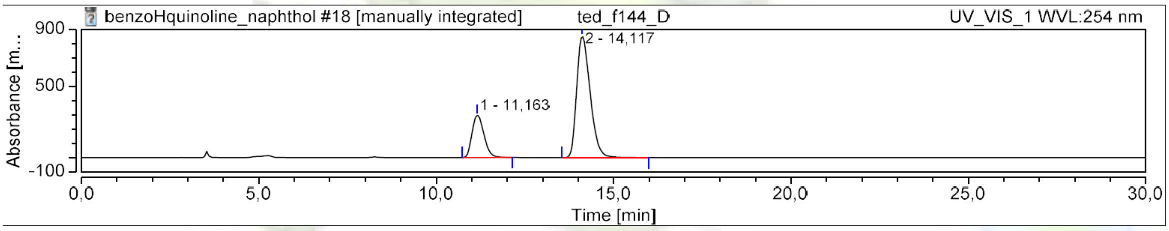

**Integration Results**							
No.	Peak Name	Retention Time min	Area mAU*min	Height mAU	Relative Area %	Relative Height %	Amount n.a.
1		11,163	116,388	296,298	23,66	25,80	n.a.
2		14,117	375,473	852,015	76,34	74,20	n.a.
Total:			**491,861**	**1148,313**	**100,00**	**100,00**	

Figure S191: HPLC separation of the isolated product **Table S1, Entry 17** & **18**.

Table S1, Entry 19 & 20

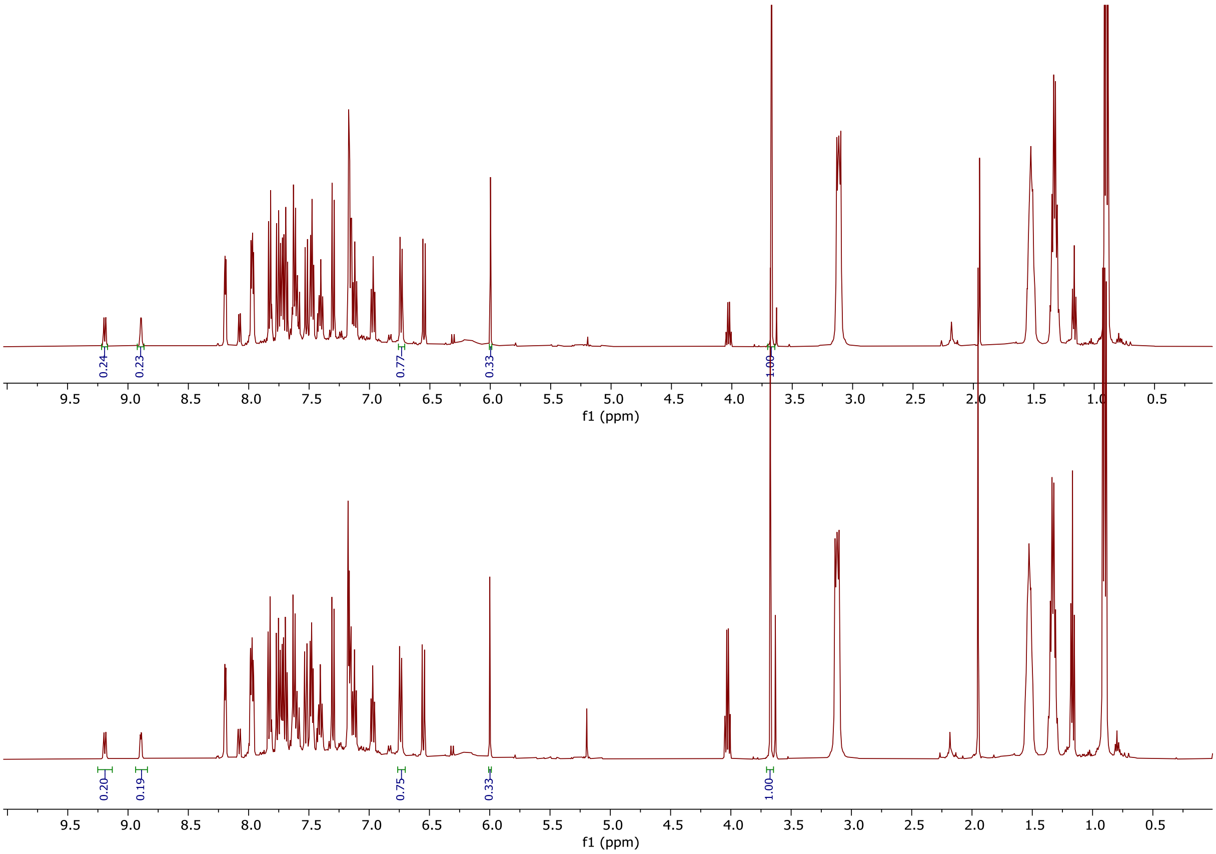

Figure S192: ^{1}H NMR of the crude reaction mixtures **Table S1**, **Entry 19** & **20** (500 MHz, 298 K, $CDCl_3$).

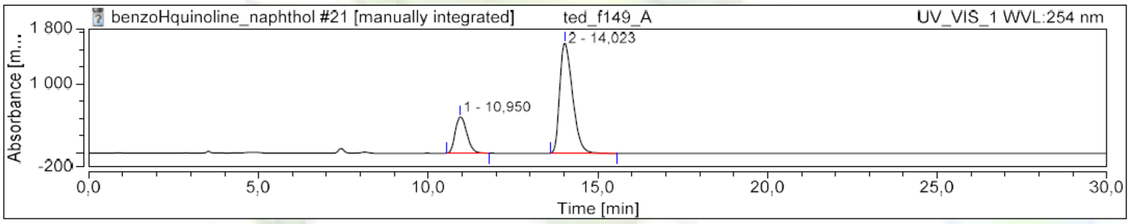

Integration Results

No.	Peak Name	Retention Time min	Area mAU*min	Height mAU	Relative Area %	Relative Height %	Amount n.a.
1		10,950	209,693	528,389	23,01	24,77	n.a.
2		14,023	701,453	1604,864	76,99	75,23	n.a.
**Total:**			**911,146**	**2133,254**	**100,00**	**100,00**	

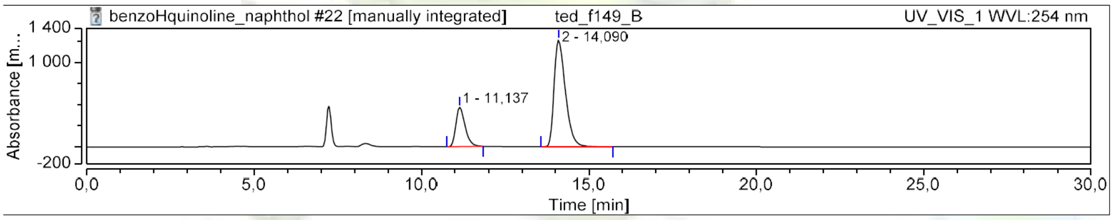

Integration Results

No.	Peak Name	Retention Time min	Area mAU*min	Height mAU	Relative Area %	Relative Height %	Amount n.a.
1		11,137	149,640	463,526	23,19	26,91	n.a.
2		14,090	495,763	1258,958	76,81	73,09	n.a.
**Total:**			**645,402**	**1722,484**	**100,00**	**100,00**	

Figure S193: HPLC separation of the isolated product **Table S1**, **Entry 19** & **20**.

Table S1, Entry 21 & 22

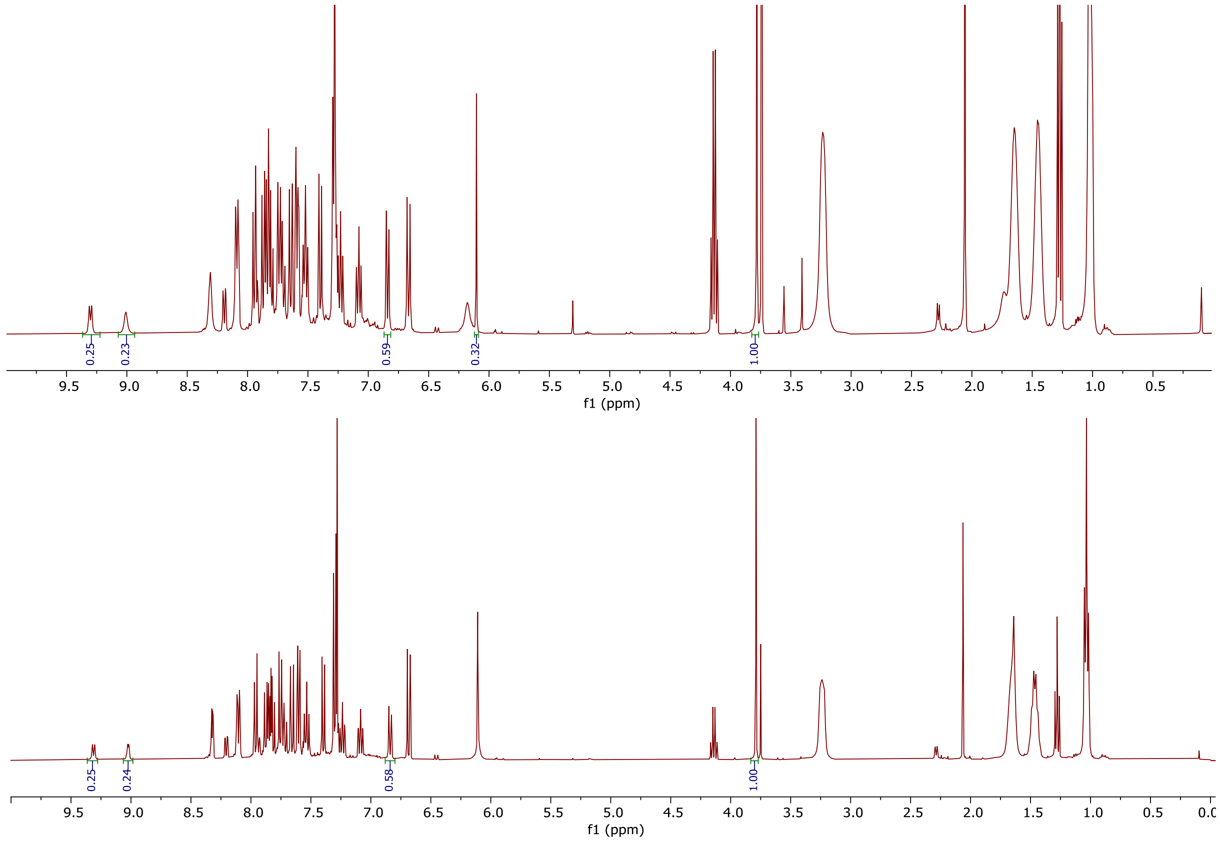

Figure S194: ^{1}H NMR of the crude reaction mixtures **Table S1**, **Entry 21** & **22** (400 MHz, 298 K, $CDCl_3$).

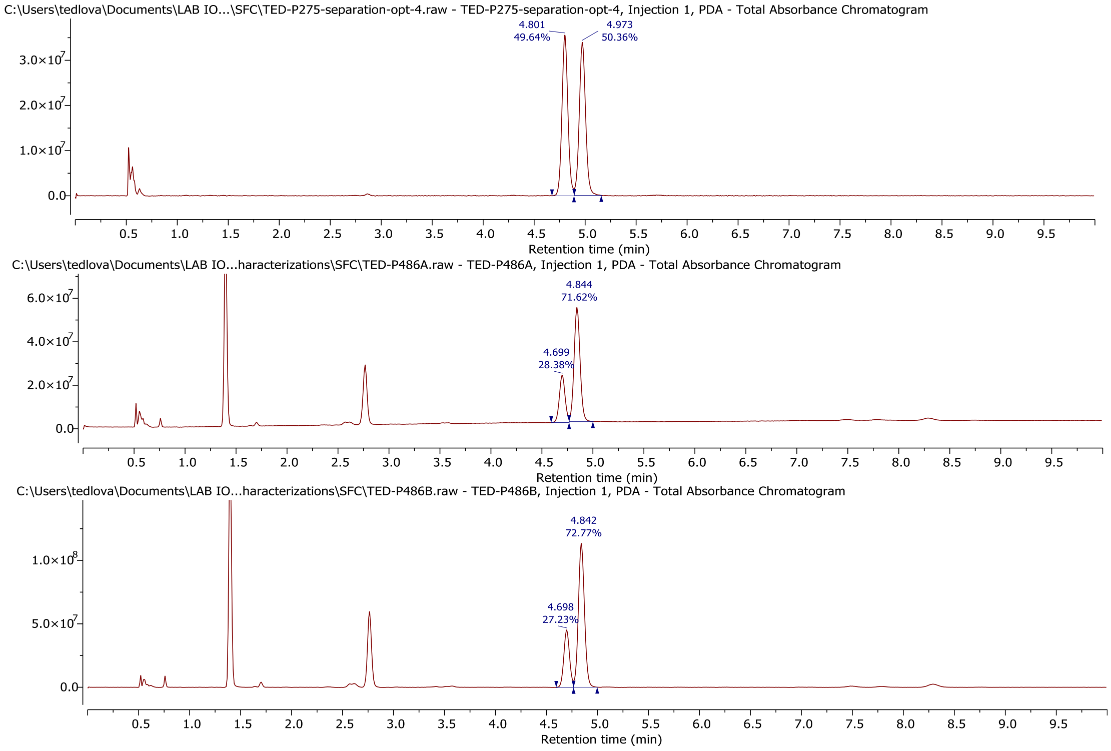

Figure S195: SFC separation of racemic **34a** (top) and the reaction mixtures **Table S1**, **Entry 21** & **22** (Alcyon Cellulose-SJ (YMC), 3 mm ID x 150 mm L, 3 µm, flow rate 1.5 mL/min, 15-40% *i*-PrOH/CO_2, backpressure 2000 psi, temperature 35 °C, 10 min, detection PDA 210 – 400 nm)

C-H activation of benzo[*h*]quinolines **32a**-**e** - Scope of the catalysis

General procedure **GP6**

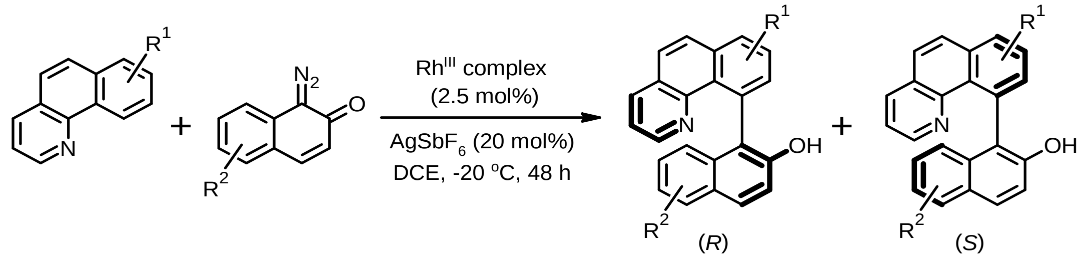

32a: R^1 = H
b: R^1 = 8-CH_3
c: R^1 = 8-Cl
d: R^1 = 6-Ph
e: R^1 = 3-Ph

33a: R^2 = H
b: R^2 = 6-Br
c: R^2 = 7-Br
d: R^1 = 7-OMe

34a: R^1 = H, R^2 = H
b: R^1 = H, R^2 = 6-Br
c: R^1 = H, R^2 = 7-Br
d: R^1 = H, R^2 = 7-OMe
e: R^1 = 8-Me, R^2 = H
f: R^1 = 8-Cl, R^2 = H
g: R^1 = 6-Ph, R^2 = H
h: R^1 = 3-Ph, R^2 = H

All reactions were performed as two parallel runs under the same conditions. The substituted benzo[*h*]quinolines **32a**-**e** (0.1 mmol, 1.0 equiv.) and $AgSbF_6$ (6.9 mg, 0.02 mmol, 20 mol%) were weighed under air into a microwave vial containing a small oval stir bar. Then catalyst (*M,R,R,R$_p$*)-**25a**(Br) (4.1 mg, 0.0025 mmol, 2.5 mol%) was added as a solution in dry dichloroethane (0.5 mL), the vial was sealed, and the mixture was inserted into a preheated block heater set to 30 °C for 15 min. The solution was then allowed to cool to -20 °C for 5 min and diazonaphthalaquinones **33a**-**d** (0.15 mmol, 1.5 equiv.) were added as solutions in dichloroethane (0.5 mL). The reactions were then allowed to stir at the same temperature for 48 h.

Fifteen minutes before the end of the reaction period, a solution of 1,3,5-trimethoxybenzene and tetra-*n*-butylammonium iodide in dichloromethane (1 mL, 1,3,5-trimethoxybenzene 1.87 mg/mL, 0.011 mmol, 0.111 equiv.; *n*-Bu_4NI 18.5 mg/mL, 0.05 mmol, 0.5 equiv.) was added. After 48 h, the reactions were opened and their content filtered through a layer of diatomaceous earth with EtOAc (30 mL). The volatiles were removed under reduced pressure and an NMR sample was prepared by dissolving the residue in $CDCl_3$. A ^{1}H NMR spectrum was measured on a 500 MHz spectrometer using a standard proton sequence by adjusting the d1 to 60 seconds and the number of scans to 1.

The residue was then purified by column chromatography on silica gel (heptane:EtOAc). The products **34a**-**e** were collected to give the isolated yields and subjected to HPLC separations to establish the enantiomeric excess. For column and HPLC conditions see the description of the corresponing compounds **34a**-**e**.

Results of the catalysis scope, characterization of products **34a-h**, NMR spectra & HPLC chromatograms of **34a-h**.

Table S2: Scope of the C-H activation

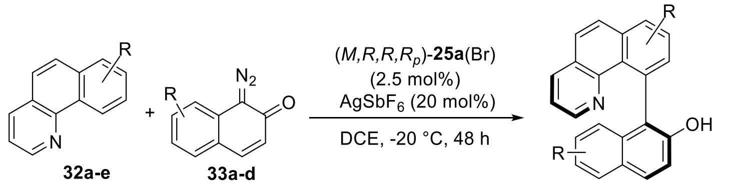

Benzo[h]quinolines

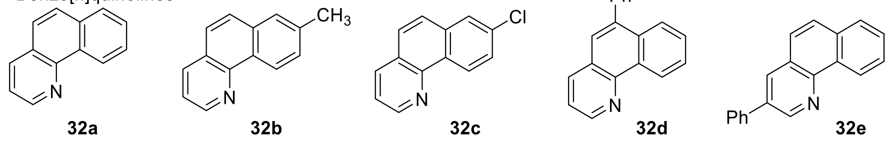

Diazonaphthalaketones

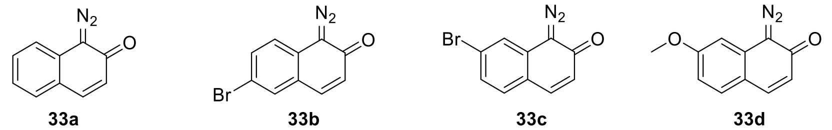

Entry	Benzo[h]quinoline	Diazoketone	Product	NMR yield (%)	NMR conversion (%)	Isolated yield (%)	*er*
1	a	a	**34a**	98	98	99	91 : 9
2	a	a	**34a**	99	99	96	91 : 9
3	a	b	**34b**	100	100	97	93 : 7
4	a	b	**34b**	100	100	95	93 : 7
5	a	c	**34c**	99	100	97	94 : 6
6	a	c	**34c**	99	100	95	94 : 6
7	a	d	**34d**	99	100	99	96 : 4
8	a	d	**34d**	99	100	99	96 : 4
9	b	a	**34e**	73	74	68	93 : 7
10	b	a	**34e**	75	78	76	93 : 7
11	c	a	**34f**	20	23	18	83 : 17
12	c	a	**34f**	19	22	17	83 : 17
13	d	a	**34g**	60	61	54	91:9
14	d	a	**34g**	70	73	60	91:9
15	e	a	**34h**	27	28	23	82 : 18
16	e	a	**34h**	28	29	24	82 : 18

Prepared according to **GP6**. Two runs were run in parallel under the same conditions.

Table S2, Entry 1 & 2

Compound (-)-(*R*)-**34a**

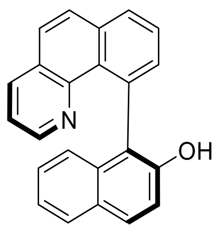

Prepared according to GP6 starting from benzo[*h*]quinoline **32a** (17.9 mg, 0.1 mmol, 1.0 equiv.) and diazonaphtalaquinone **33a** (25.5 mg, 0.15 mmol, 1.5 equiv.). After column chromatography on silica gel (heptane:EtOAc 6:1), the two runs gave products (-)-(*R*)-**34a** (32.0 mg, 99%, and 30.8 mg, 96%, respectively) as grey amorphous solids.

$[\alpha]^{20}_D$ -118 (*c* 0.126, $CHCl_3$).

NMR in accordance with literature.[10] **^{1}H NMR** (500 MHz, 298 K, $CDCl_3$): δ = 8.30 (d, *J* = 4.4 Hz, 1H), 8.13 – 8.03 (m, 2H), 7.94 (d, *J* = 8.7 Hz, 1H), 7.89 – 7.76 (m, 3H), 7.73 (d, *J* = 8.8 Hz, 1H), 7.58 (d, *J* = 7.3 Hz, 1H), 7.37 (d, *J* = 8.8 Hz, 1H), 7.30 – 7.18 (m, 2H, overlapping with solvent signal), 7.06 (t, *J* = 7.6 Hz, 1H), 6.82 (d, *J* = 8.5 Hz, 1H), 6.02 (s, 1H).

^{13}C{^{1}H} NMR (126 MHz, 298 K, $CDCl_3$): δ = 150.5, 148.0, 146.7, 135.5, 135.4, 134.3, 133.6, 133.1, 131.1, 129.4, 129.3, 128.6, 128.3, 128.1, 127.9, 127.3, 126.7, 126.2, 125.6, 125.2, 122.8, 121.6, 118.6.

HPLC conditions: Lux Cellulose-1 column, *n*-hexane/*i*-PrOH, 90:10 v/v, flow rate 1 mL/min, λ = 254 nm, 25 °C. t_R (minor) = 11.1 min, t_R (major) = 14.0 min, 91:9 *er*.

For NMR spectra of the crude reaction, mixture and HPLC chromatograms, see **Figure S180** and **S181** (**Table S1, Entry 7** & **8**).

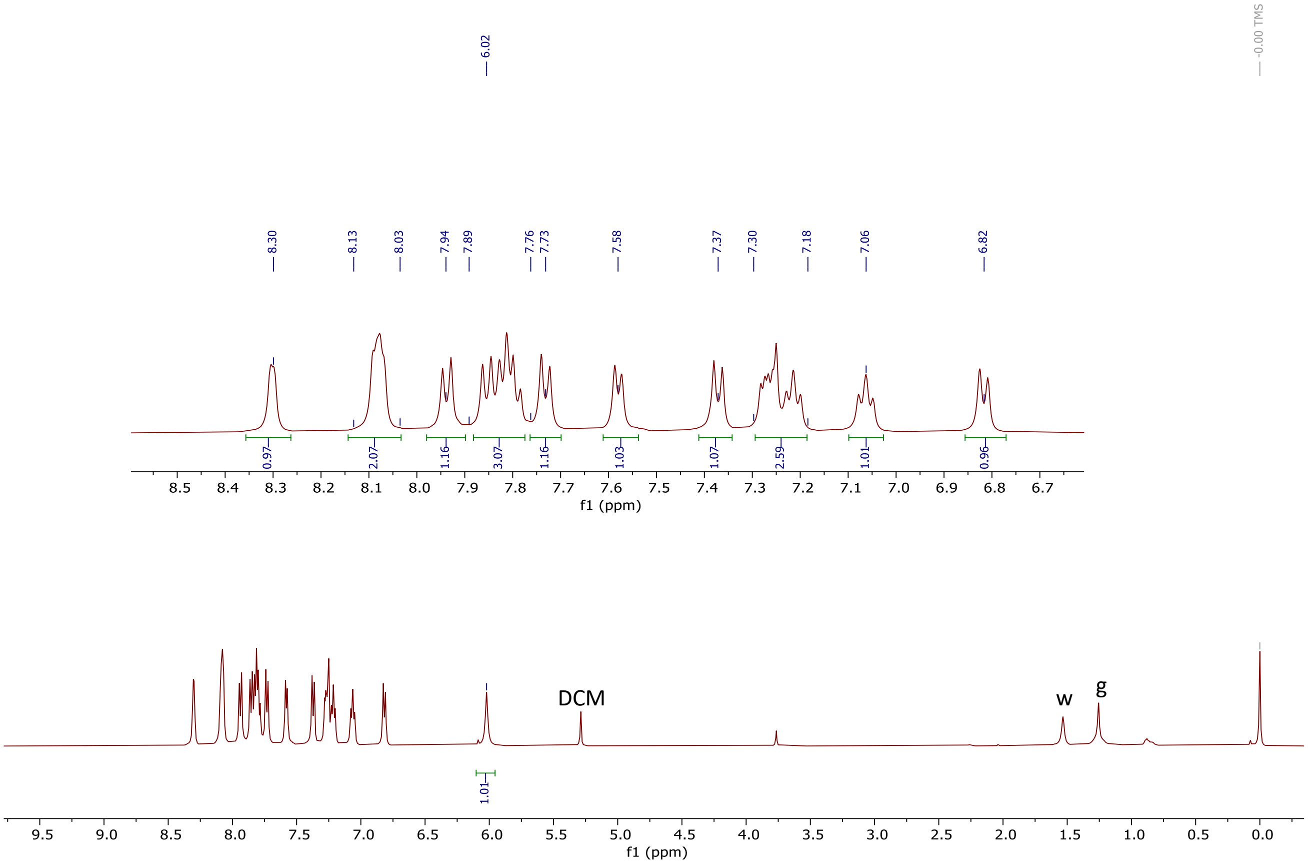

Figure S196: ^{1}H NMR of 34a (500 MHz, 298 K, $CDCl_3$ + 0.03 % TMS) (DCM = dichloromethane, w = water, g = grease).

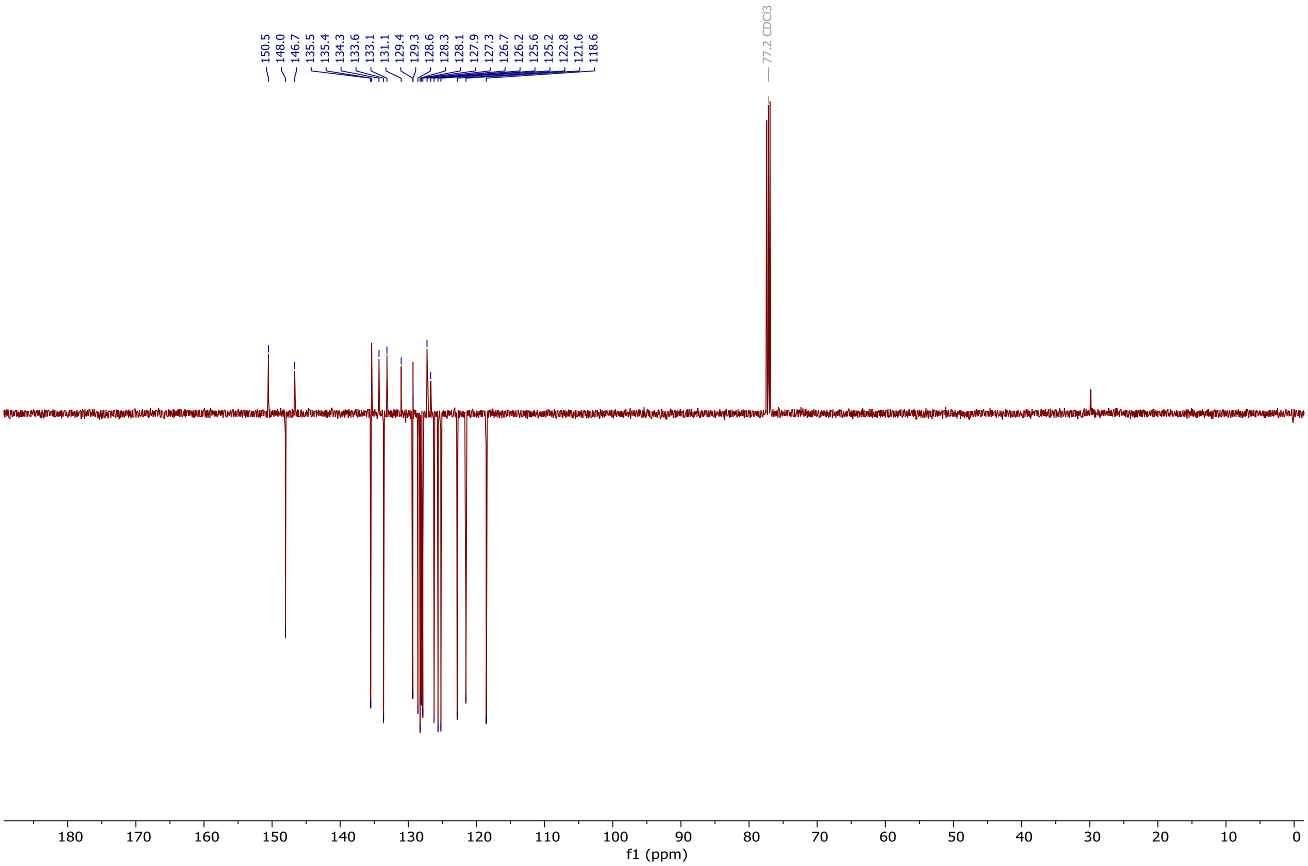

Figure S197: ^{13}C APT NMR of 34a (126 MHz, 298 K, $CDCl_3$ + 0.03 % TMS).

Table S2, Entry 3 & 4

Compound (+)-(*R*)-**34b**

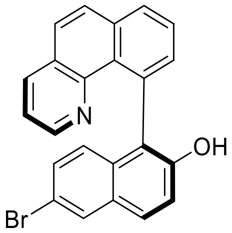

Prepared according to **GP6** starting from benzo[*h*]quinoline **32a** (17.9 mg, 0.1 mmol, 1.0 equiv.) and diazonaphtalaquinone **33b** (37.4 mg, 0.15 mmol, 1.5 equiv.). After column chromatography on silica gel (heptane:EtOAc 6:1), the two runs gave products (-)-(*R*)-**34b** (38.6 mg, 97%, and 37.9 mg, 95%, respectively) as grey amorphous solids.

[α]$^{20}_{D}$ +40 (*c* 0.184, $CHCl_3$).

NMR in accordance with literature.[10] **^{1}H NMR** (500 MHz, 298 K, $CDCl_3$): δ = 8.27 (dd, *J* = 4.3, 1.8 Hz, 1H), 8.09 (m, 2H), 8.00 – 7.91 (m, 2H), 7.81 (t, *J* = 7.6 Hz, 1H), 7.75 (m, 2H), 7.55 (dd, *J* = 7.3, 1.4 Hz, 1H), 7.37 (d, *J* = 8.8 Hz, 1H), 7.28 (dd, *J* = 8.0, 4.3 Hz, 1H), 7.11 (dd, *J* = 9.0, 2.1 Hz, 1H), 6.72 (d, *J* = 9.0 Hz, 1H), 5.79 (s, 1H).

^{13}C{^{1}H} NMR (126 MHz, 298 K, $CDCl_3$): δ = 150.5, 148.0, 146.4, 135.5, 135.3, 133.3, 132.7, 132.2, 130.8, 130.3, 129.6, 129.5, 128.7, 128.4, 128.0, 127.18, 127.16, 127.0, 126.7, 126.3, 121.5, 119.3, 116.5.

HPLC conditions: Lux Cellulose-1 column, *n*-hexane/*i*-PrOH, 90:10 v/v, flow rate 1 mL/min, λ = 254 nm, 25 °C. t_R (minor) = 16.4 min, t_R (major) = 20.0 min, 93:7 *er*.

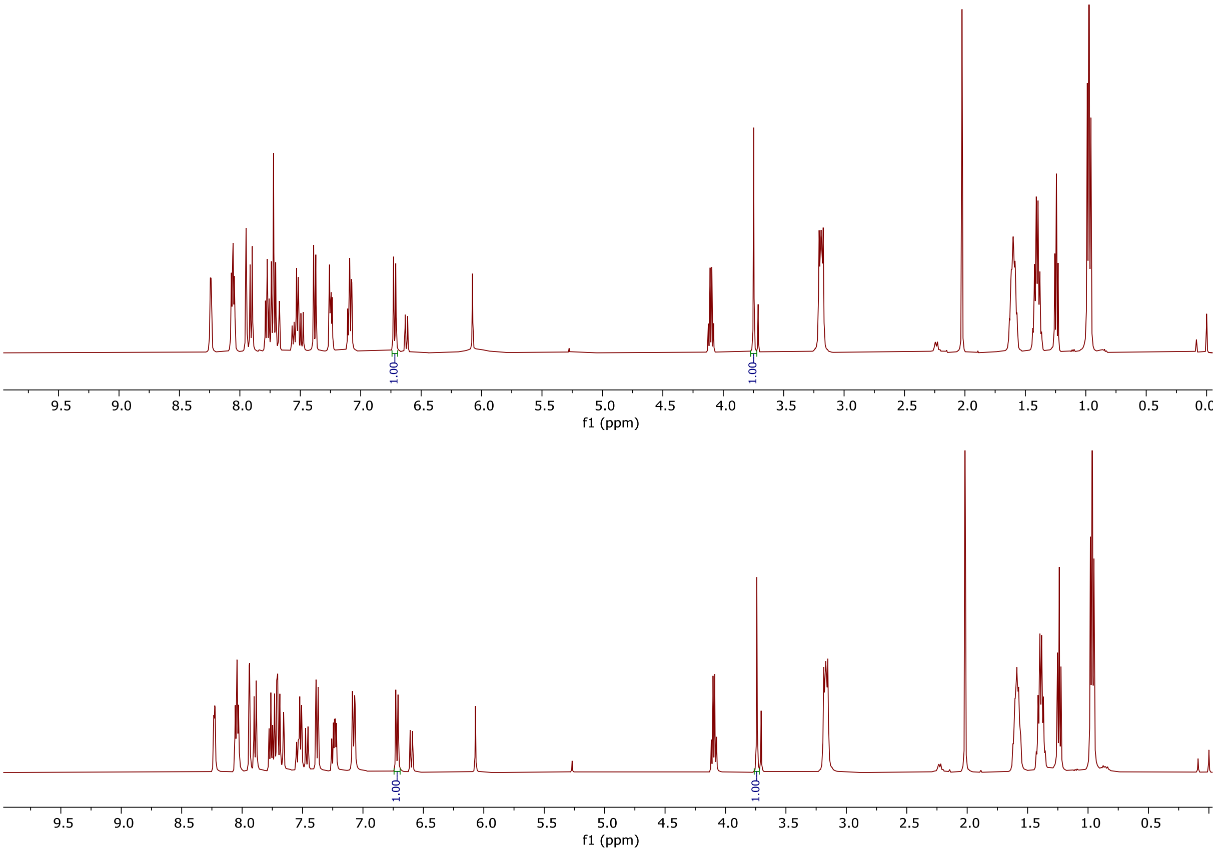

Figure S198: ^{1}H NMR of the crude reaction mixtures of **34b** (500 MHz, 298 K, $CDCl_3$).

Chromatogram

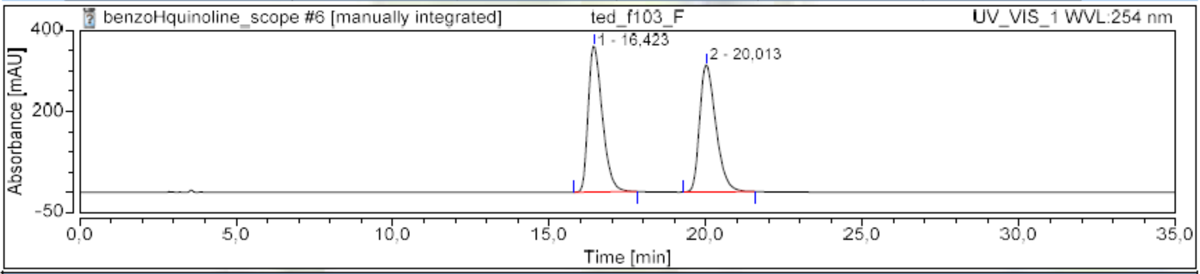

Integration Results

No.	Peak Name	Retention Time min	Area mAU*min	Height mAU	Relative Area %	Relative Height %	Amount n.a.
1		16,423	189,149	362,072	50,00	53,50	n.a.
2		20,013	189,127	314,722	50,00	46,50	n.a.
**Total:**			**378,276**	**676,795**	**100,00**	**100,00**	

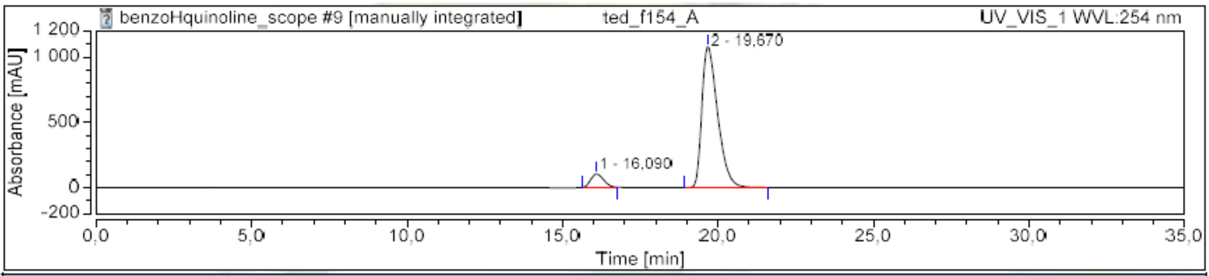

Integration Results

No.	Peak Name	Retention Time min	Area mAU*min	Height mAU	Relative Area %	Relative Height %	Amount n.a.
1		16,090	49,299	103,151	7,19	8,69	n.a.
2		19,670	635,921	1083,319	92,81	91,31	n.a.
**Total:**			**685,220**	**1186,470**	**100,00**	**100,00**	

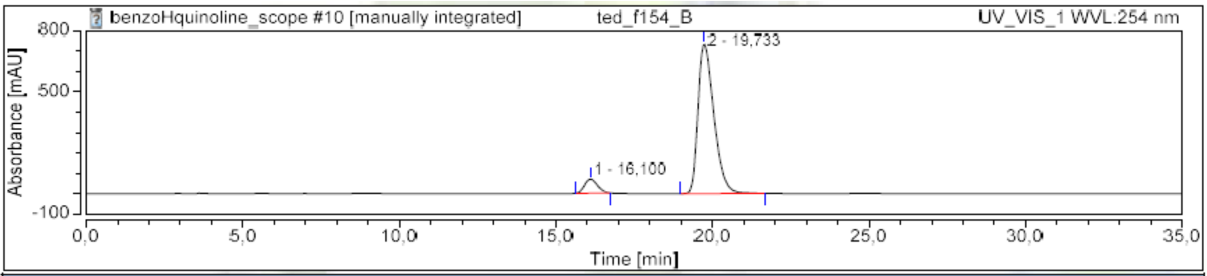

Integration Results

No.	Peak Name	Retention Time min	Area mAU*min	Height mAU	Relative Area %	Relative Height %	Amount n.a.
1		16,100	33,094	69,145	7,13	8,59	n.a.
2		19,733	430,919	736,206	92,87	91,41	n.a.
**Total:**			**464,013**	**805,352**	**100,00**	**100,00**	

Figure S199: HPLC separation of the racemic standard and isolated product of the asymmetric catalysis **34b**.

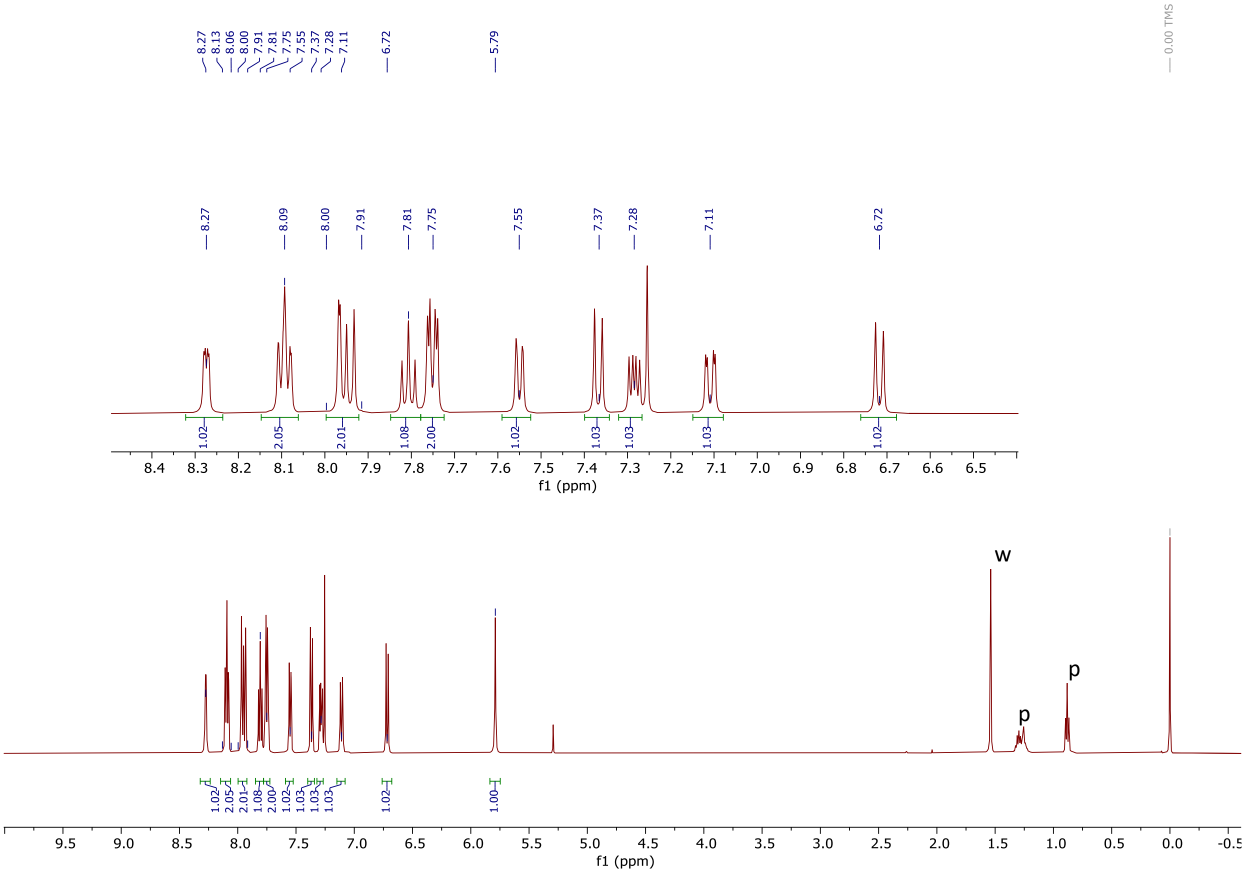

Figure S200: ^{1}H NMR of 34b (500 MHz, 298 K, $CDCl_3$ + 0.03 % TMS) (w = water, p = pentane).

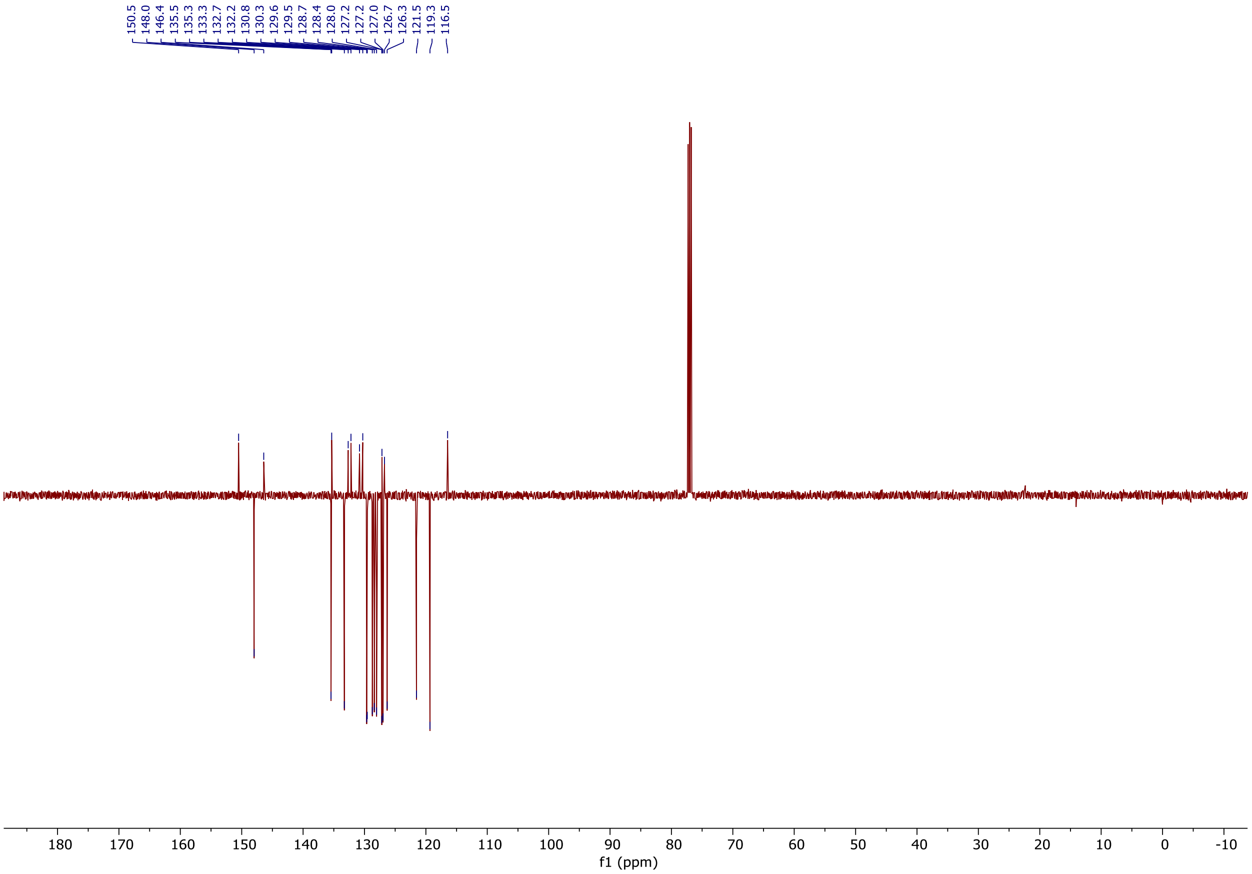

Figure S201: ^{13}C APT NMR of 34b (126 MHz, 298 K, $CDCl_3$ + 0.03 % TMS).

Table S2, Entry 5 & 6

Compound (+)-(*R*)-**34c**

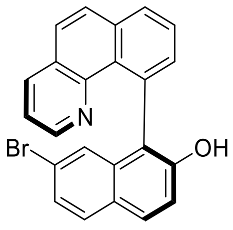

Prepared according to **GP6** starting from benzo[*h*]quinoline **32a** (17.9 mg, 0.1 mmol, 1.0 equiv.) and diazonaphtalaquinone **33c** (37.4 mg, 0.15 mmol, 1.5 equiv.). After column chromatography on silica gel (heptane:EtOAc 6:1), the two runs gave products (+)-(*R*)-**34c** (38.9 mg, 97%, and 38.1 mg, 95%, respectively) as grey amorphous solids.

[α]$^{20}_{D}$ +9 (*c* 0.141, $CHCl_3$).

NMR in accordance with literature.[10] **^{1}H NMR** (500 MHz, 289 K, $CDCl_3$): δ = 8.29 (dd, *J* = 4.4, 1.8 Hz, 1H), 8.11 (m, 2H), 7.95 (d, *J* = 8.8 Hz, 1H), 7.85 – 7.80 (m, 2H), 7.76 (d, *J* = 8.8 Hz, 1H), 7.69 (d, *J* = 8.7 Hz, 1H), 7.55 (dd, *J* = 7.3, 1.4 Hz, 1H), 7.35 (d, *J* = 8.8 Hz, 1H), 7.32 – 7.27 (m, 2H), 7.01 (d, *J* = 2.0 Hz, 1H), 5.82 (s, 1H).

^{13}C{^{1}H} NMR (126 MHz, 298 K, $CDCl_3$): δ = 151.3, 148.1, 146.6, 135.6, 135.5, 133.6, 132.1, 130.8, 129.8, 129.6, 128.6, 128.23, 128.20, 127.7, 127.3, 127.3, 126.4, 126.2, 126.1, 121.7, 120.2, 118.8.

HPLC conditions: Lux Cellulose-1 column, *n*-hexane/*i*-PrOH, 90:10 v/v, flow rate 1 mL/min, λ = 254 nm, 25 °C. t_R (minor) = 16.2 min, t_R (major) = 22.4 min, 94:6 *er*.

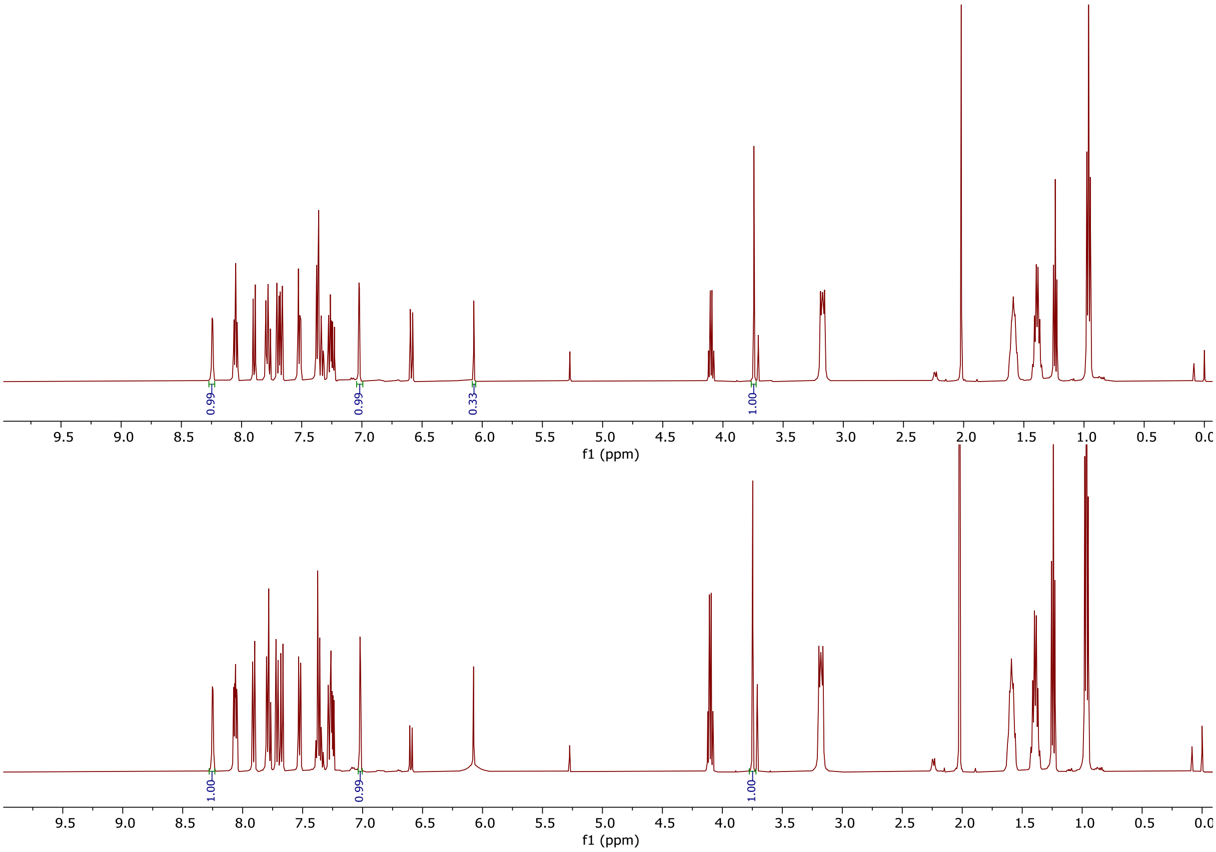

Figure S202: ^{1}H NMR of the crude reaction mixtures **34c** (500 MHz, 298 K, $CDCl_3$).

Chromatogram

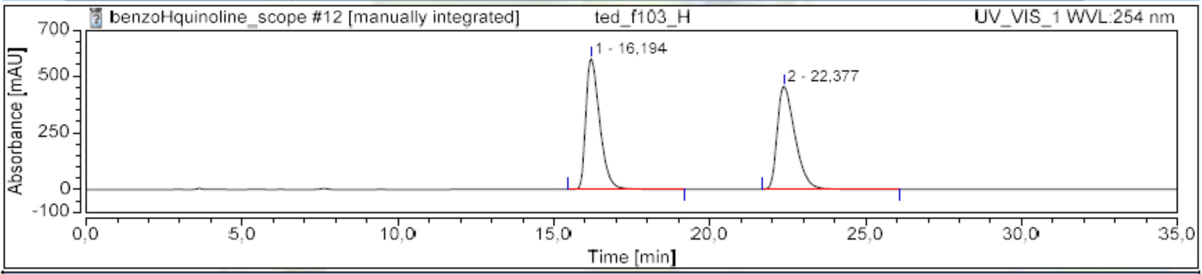

Integration Results

No.	Peak Name	Retention Time min	Area mAU*min	Height mAU	Relative Area %	Relative Height %	Amount n.a.
1		16,194	297,684	576,433	49,96	55,93	n.a.
2		22,377	298,207	454,261	50,04	44,07	n.a.
**Total:**			**595,891**	**1030,695**	**100,00**	**100,00**	

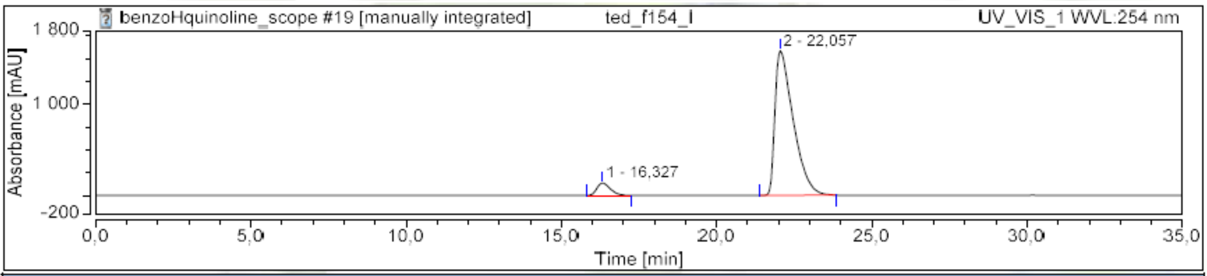

Integration Results

No.	Peak Name	Retention Time min	Area mAU*min	Height mAU	Relative Area %	Relative Height %	Amount n.a.
1		16,327	68,069	135,821	5,94	7,91	n.a.
2		22,057	1078,425	1580,686	94,06	92,09	n.a.
**Total:**			**1146,495**	**1716,507**	**100,00**	**100,00**	

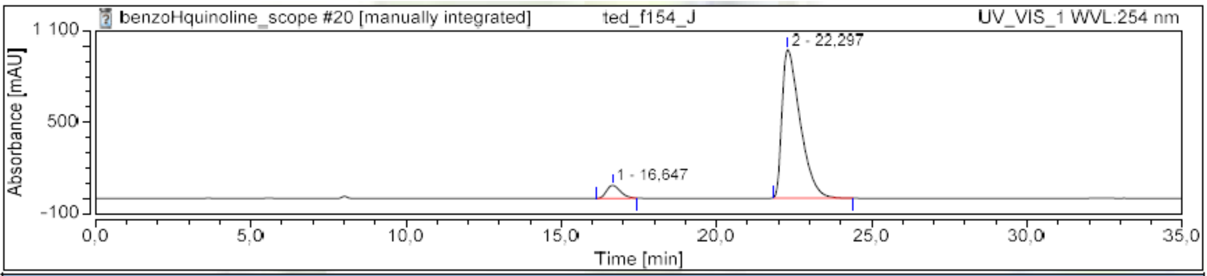

Integration Results

No.	Peak Name	Retention Time min	Area mAU*min	Height mAU	Relative Area %	Relative Height %	Amount n.a.
1		16,647	43,479	84,984	6,18	8,02	n.a.
2		22,297	660,074	975,236	93,82	91,98	n.a.
**Total:**			**703,553**	**1060,220**	**100,00**	**100,00**	

Figure S203: HPLC separation of the racemic standard and isolated product of the asymmetric catalysis **34c**.

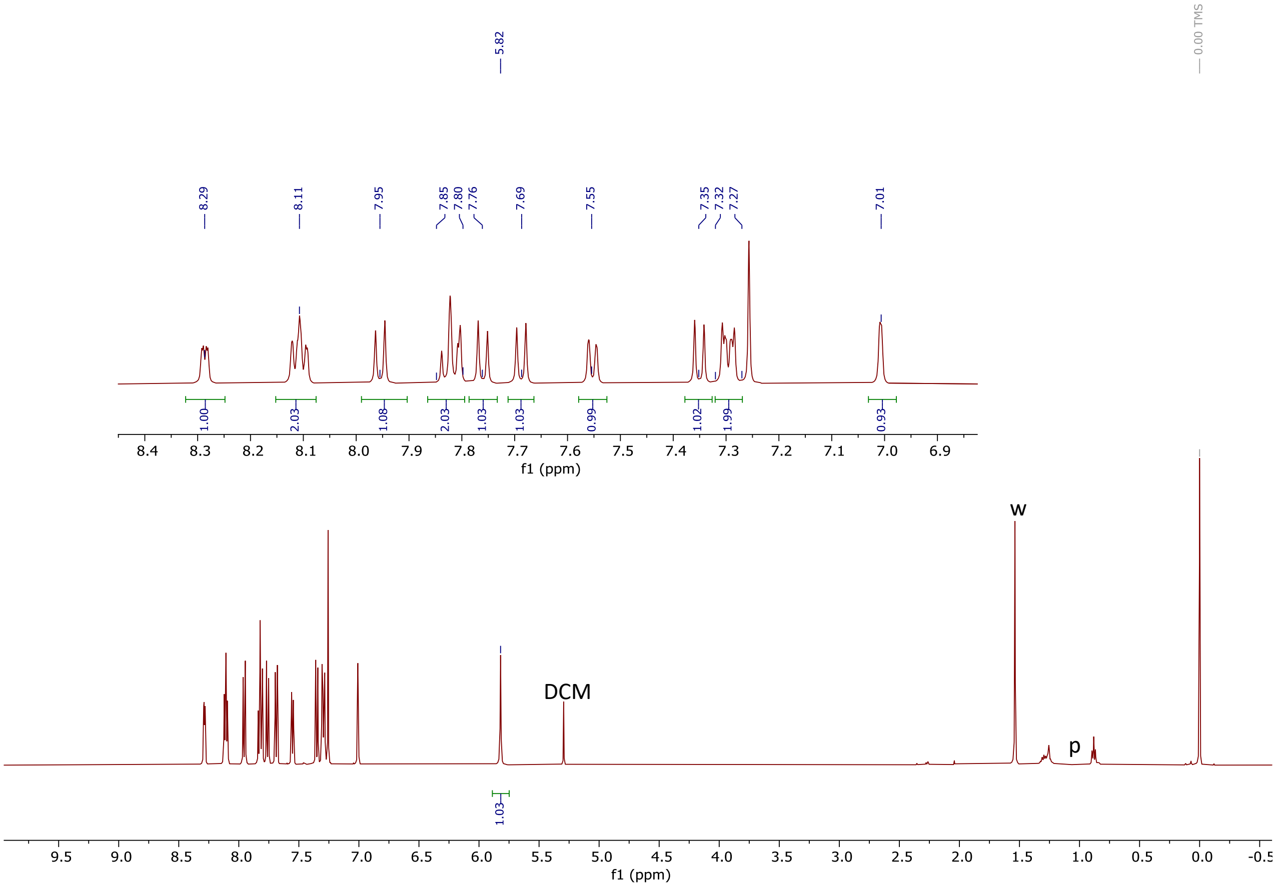

Figure S204: ^{1}H NMR of 34c (500 MHz, 298 K, $CDCl_3$ + 0.03 % TMS) (DCM= dichloromethane, w = water, p = pentane).

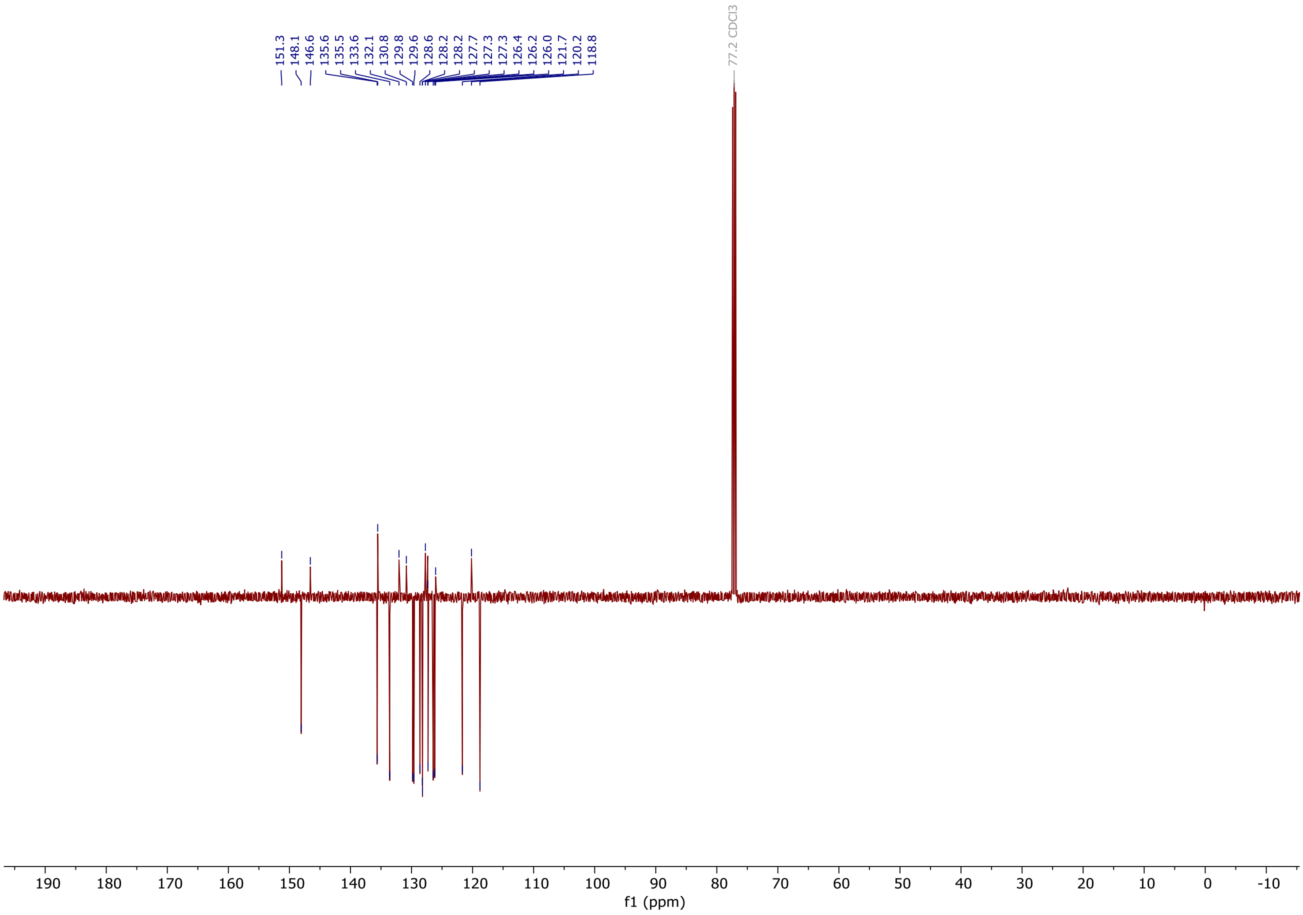

Figure S205: ^{13}C APT NMR of 34c (126 MHz, 298 K, $CDCl_3$ + 0.03 % TMS).

Table S2, Entry 7 & 8

Compound (-)-(*R*)-**34d**

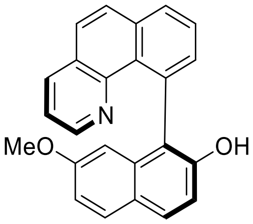

Prepared according to **GP6** starting from benzo[*h*]quinoline **32a** (17.9 mg, 0.1 mmol, 1.0 equiv.) and diazonaphtalaquinone **33d** (30.0 mg, 0.15 mmol, 1.5 equiv.). After column chromatography on silica gel (heptane:EtOAc, 6:1), the two runs gave products (-)-(*R*)-**34d** (35.1 mg, 99%, and 34.8 mg, 99%, respectively) as grey amorphous solids.

[α]$^{20}_{D}$ -67 (*c* 0.143, $CHCl_3$).

NMR in accordance with literature.[10] **^{1}H NMR** (500 MHz, $CDCl_3$): δ = 8.34 (dd, *J* = 4.4, 1.9 Hz, 1H), 8.09 (m, 2H), 7.94 (d, *J* = 8.8 Hz, 1H), 7.83 – 7.70 (m, 4H), 7.60 (dd, *J* = 7.2, 1.4 Hz, 1H), 7.30 (dd, *J* = 8.0, 4.3 Hz, 1H), 7.22 (d, *J* = 8.8 Hz, 1H), 6.90 (dd, *J* = 8.9, 2.5 Hz, 1H), 6.13 (d, *J* = 2.6 Hz, 1H), 6.01 (s, 1H), 3.40 (s, 3H).

^{13}C{^{1}H} NMR (126 MHz, $CDCl_3$): δ = 157.6, 151.2, 148.1, 146.8, 135.53, 135.49, 135.4, 133.7, 133.3, 131.0, 129.42, 129.35, 128.6, 128.2, 128.1, 127.2, 126.2, 125.9, 124.9, 121.6, 116.2, 114.6, 104.7, 55.0.

HPLC conditions: Lux Cellulose-1 column, *n*-hexane/*i*-PrOH, 90:10 v/v, flow rate 1 mL/min, λ = 254 nm, 25 °C. t_R (minor) = 18.4 min, t_R (major) = 25.8 min, 96:4 *er*.

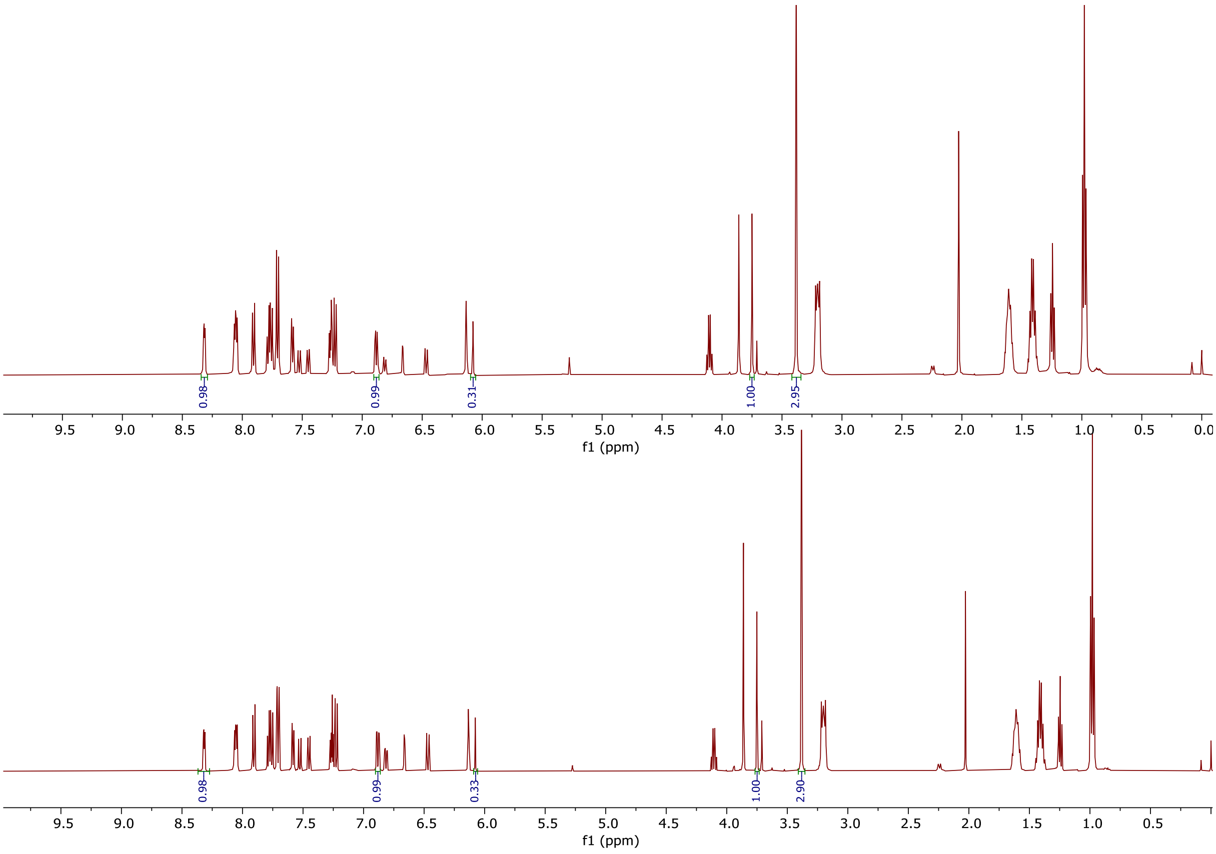

Figure S206: ^{1}H NMR of the crude reaction mixtures **34d** (500 MHz, 298 K, $CDCl_3$).

Chromatogram

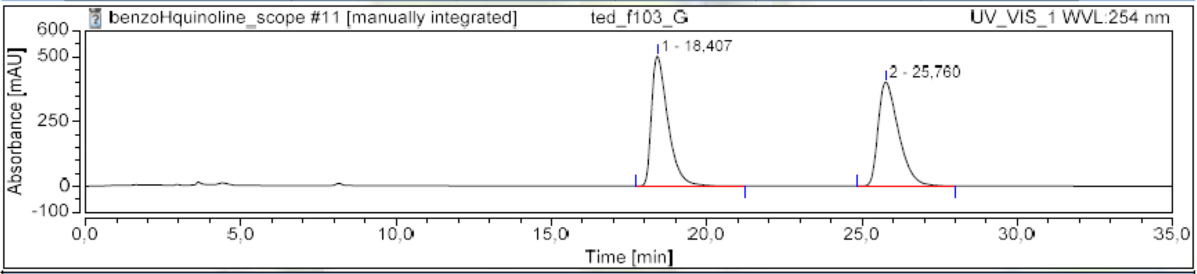

Integration Results

No.	Peak Name	Retention Time min	Area mAU*min	Height mAU	Relative Area %	Relative Height %	Amount n.a.
1		18,407	308,293	501,651	50,05	55,51	n.a.
2		25,760	307,719	402,137	49,95	44,49	n.a.
**Total:**			**616,012**	**903,788**	**100,00**	**100,00**	

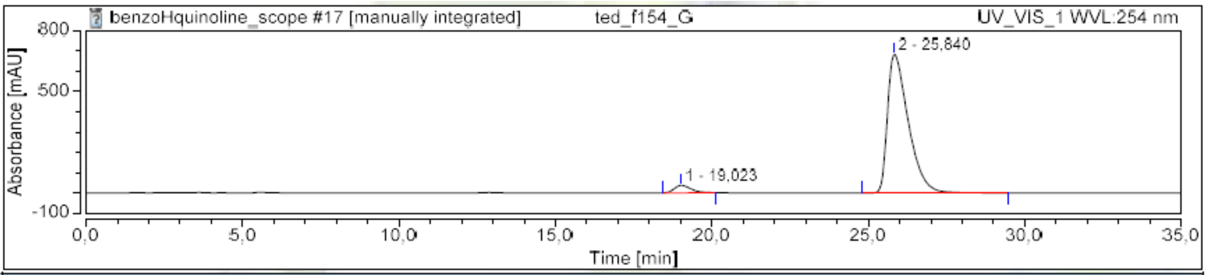

Integration Results

No.	Peak Name	Retention Time min	Area mAU*min	Height mAU	Relative Area %	Relative Height %	Amount n.a.
1		19,023	22,195	37,021	4,05	5,13	n.a.
2		25,840	526,445	684,647	95,95	94,87	n.a.
**Total:**			**548,640**	**721,668**	**100,00**	**100,00**	

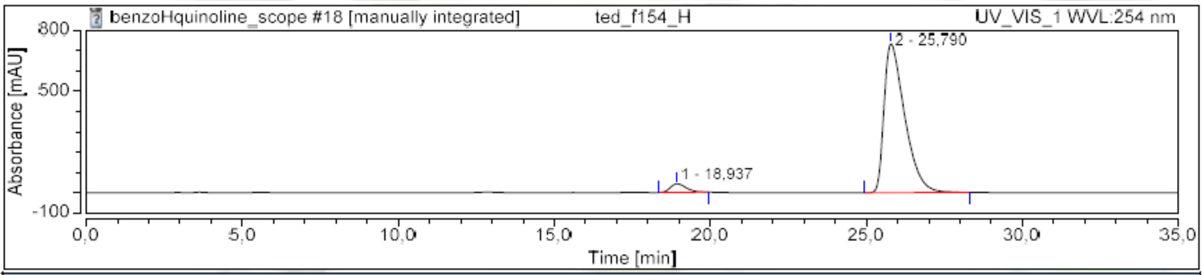

Integration Results

No.	Peak Name	Retention Time min	Area mAU*min	Height mAU	Relative Area %	Relative Height %	Amount n.a.
1		18,937	25,005	42,141	4,24	5,43	n.a.
2		25,790	565,243	734,026	95,76	94,57	n.a.
**Total:**			**590,248**	**776,167**	**100,00**	**100,00**	

Figure S207: HPLC separation of the racemic standard and isolated product of the asymmetric catalysis **34d**.

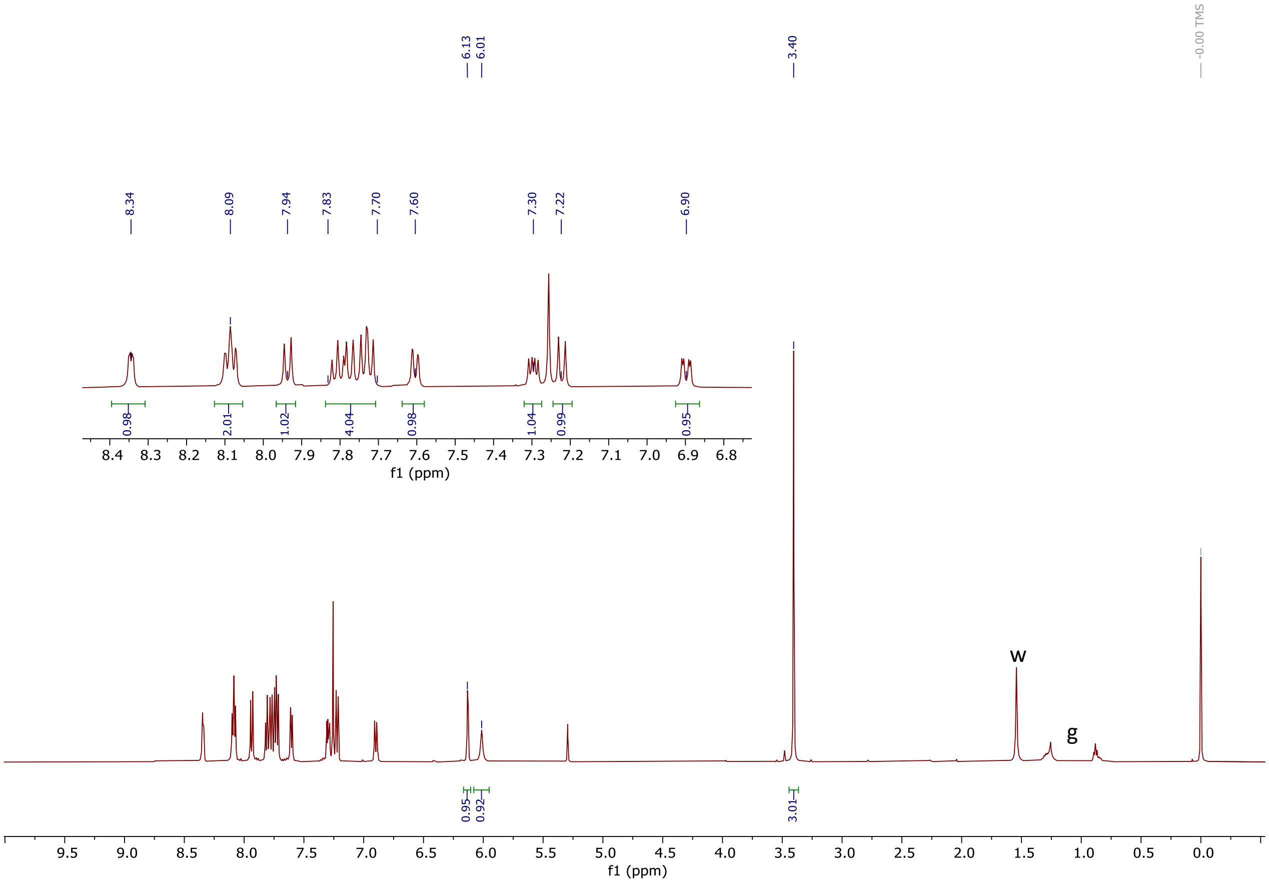

Figure S208: ¹H NMR of 34d (500 MHz, 298 K, $CDCl_3$ + 0.03 % TMS) (w = water, g = grease).

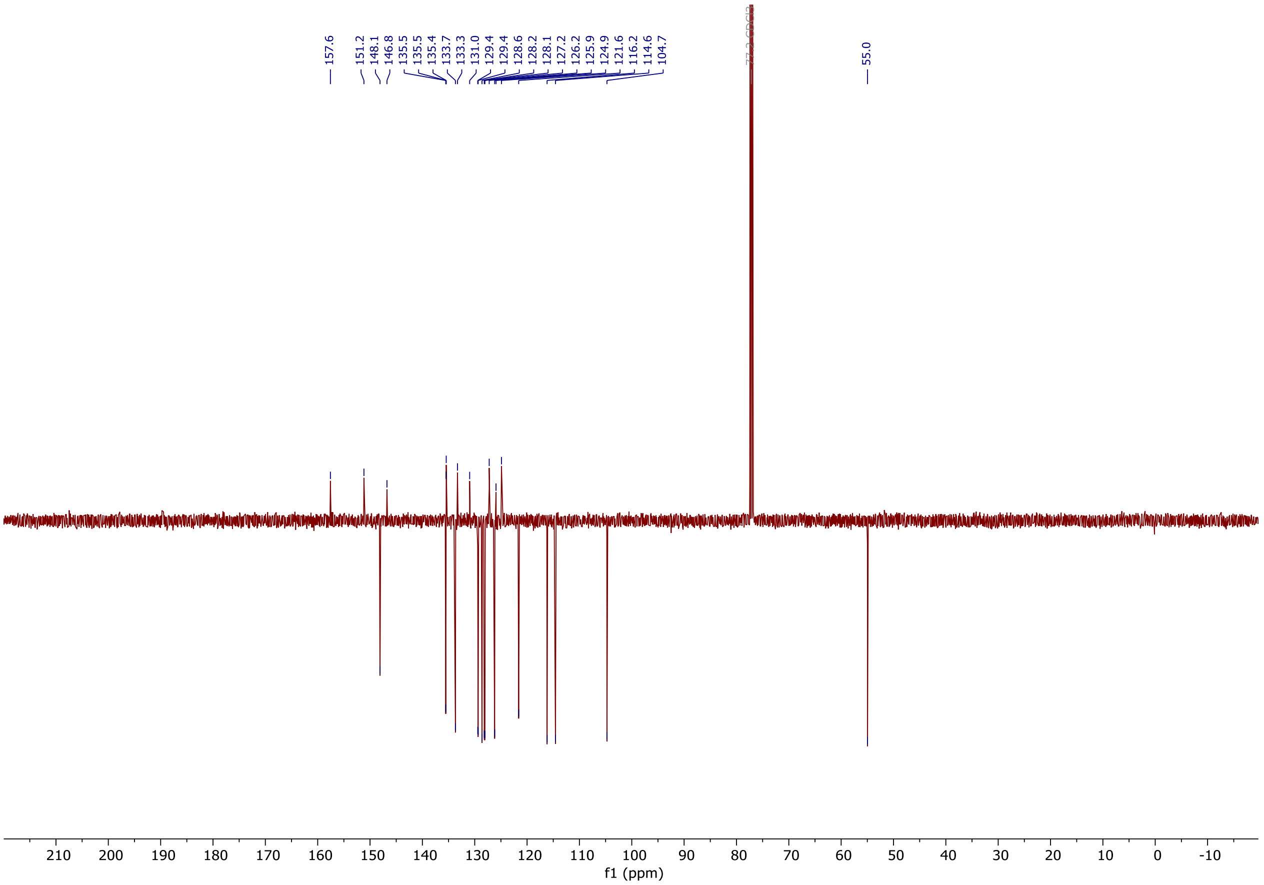

Figure S209: ¹³C APT NMR of 34d (126 MHz, 298 K, $CDCl_3$ + 0.03 % TMS).

Table S2, Entry 9 & 10

Compound (-)-(*R*)-**34e**

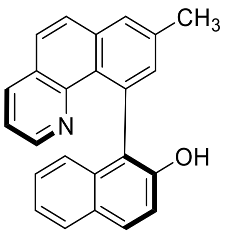

Prepared according to **GP6** starting from 8-methylbenzo[*h*]quinoline **32b** (19.3 mg, 0.1 mmol, 1.0 equiv.) and diazonaphtalaquinone **33a** (25.5 mg, 0.15 mmol, 1.5 equiv.). After column chromatography on silica gel (heptane:EtOAc 6:1), the two runs gave products (-)-(*R*)-**34e** (22.8 mg, 68%, and 25.5 mg, 76%, respectively) as light pink amorphous solids.

$[\alpha]^{20}_{D}$ -176 (*c* 0.110, $CHCl_3$).

^{1}H NMR (500 MHz, 298 K, $CDCl_3$): δ = 8.29 (dd, *J* = 4.3, 1.8 Hz, 1H), 8.06 (dd, *J* = 8.0, 1.9 Hz, 1H), 7.90 – 7.80 (m, 4H), 7.70 (d, *J* = 8.8 Hz, 1H), 7.42 (d, *J* = 1.9 Hz, 1H), 7.38 (d, *J* = 8.8 Hz, 1H), 7.27 – 7.19 (m, 2H, overlapping with the solvent signal), 7.08 (ddd, *J* = 8.2, 6.7, 1.4 Hz, 1H), 6.85 (m, 1H), 6.13 (s, 1H), 2.60 (s, 3H).

^{13}C{^{1}H} NMR (126 MHz, 298 K, $CDCl_3$): δ = 150.6, 148.0, 146.8, 138.1, 135.6, 135.5, 135.2, 134.4, 132.9, 129.3, 129.0, 128.9, 128.4, 128.2, 127.9, 126.9, 126.8, 126.2, 125.6, 125.3, 122.8, 121.2, 118.6, 21.5.

HRMS (ESI) *m/z*: ([M+H]$^{+}$) calcd for $C_{24}H_{18}ON$ 336.1383, found 336.1382 (Δ = -0.38 ppm).

IR (ATR): 3524 (w), 3406 (w), 3044 (w), 2919 (w), 2854 (w), 1619 (m), 1594 (m-s), 1565 (w), 1508 (m), 1435 (w), 1435 (w), 1398 (w), 1380 (m), 1329 (w), 1311 (w), 1270 (w), 1239 (w), 1186 (m), 1134 (m), 1110 (w), 1039 (w), 958 (m), 895 (w), 868 (s), 829 (w), 811 (vs), 780 (w), 746 (s), 730 (vs), 694 (m), 627 (w), 576 (w), 548 (w), 538 (w), 496 (w), 477 (w) cm^{-1}.

HPLC conditions: Lux Cellulose-1 column, *n*-hexane/*i*-PrOH, 90:10 v/v, flow rate 1 mL/min, λ = 254 nm, 25 °C. t_R (minor) = 12.3 min, t_R (major) = 20.1 min, 93:7 *er*.

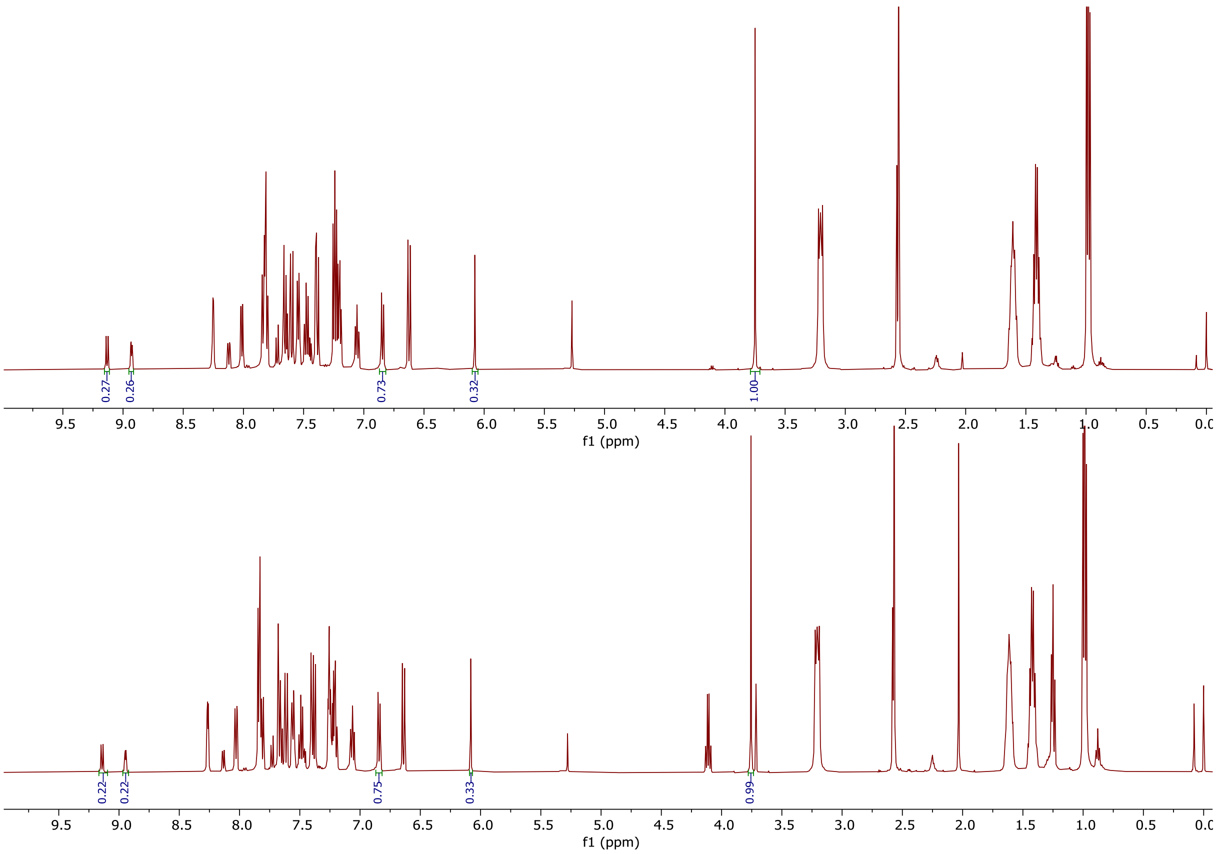

Figure S210: ^{1}H NMR of the crude reaction mixtures **34e** (500 MHz, 298 K, $CDCl_3$).

Chromatogram

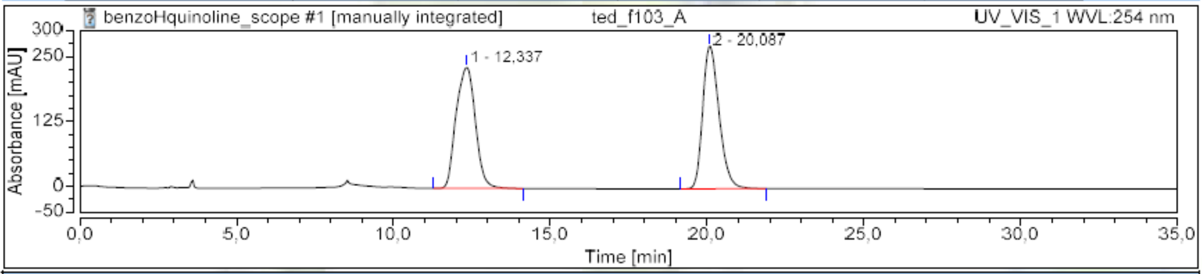

Integration Results

No.	Peak Name	Retention Time min	Area mAU*min	Height mAU	Relative Area %	Relative Height %	Amount n.a.
1		12,337	166,092	233,268	49,95	45,81	n.a.
2		20,087	166,457	275,948	50,05	54,19	n.a.
**Total:**			**332,549**	**509,216**	**100,00**	**100,00**	

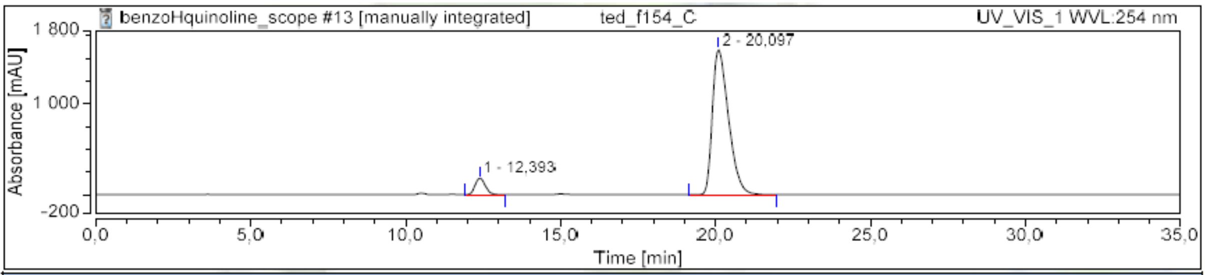

Integration Results

No.	Peak Name	Retention Time min	Area mAU*min	Height mAU	Relative Area %	Relative Height %	Amount n.a.
1		12,393	70,155	182,220	6,79	10,27	n.a.
2		20,097	962,668	1592,934	93,21	89,73	n.a.
**Total:**			**1032,823**	**1775,154**	**100,00**	**100,00**	

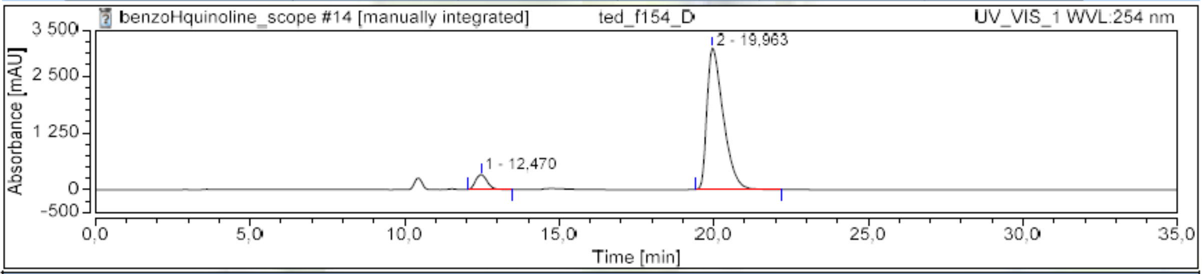

Integration Results

No.	Peak Name	Retention Time min	Area mAU*min	Height mAU	Relative Area %	Relative Height %	Amount n.a.
1		12,470	140,523	326,167	6,89	9,48	n.a.
2		19,963	1898,913	3115,142	93,11	90,52	n.a.
**Total:**			**2039,435**	**3441,309**	**100,00**	**100,00**	

Figure S211: HPLC separation of the racemic standard and isolated product of the asymmetric catalysis **34e**.

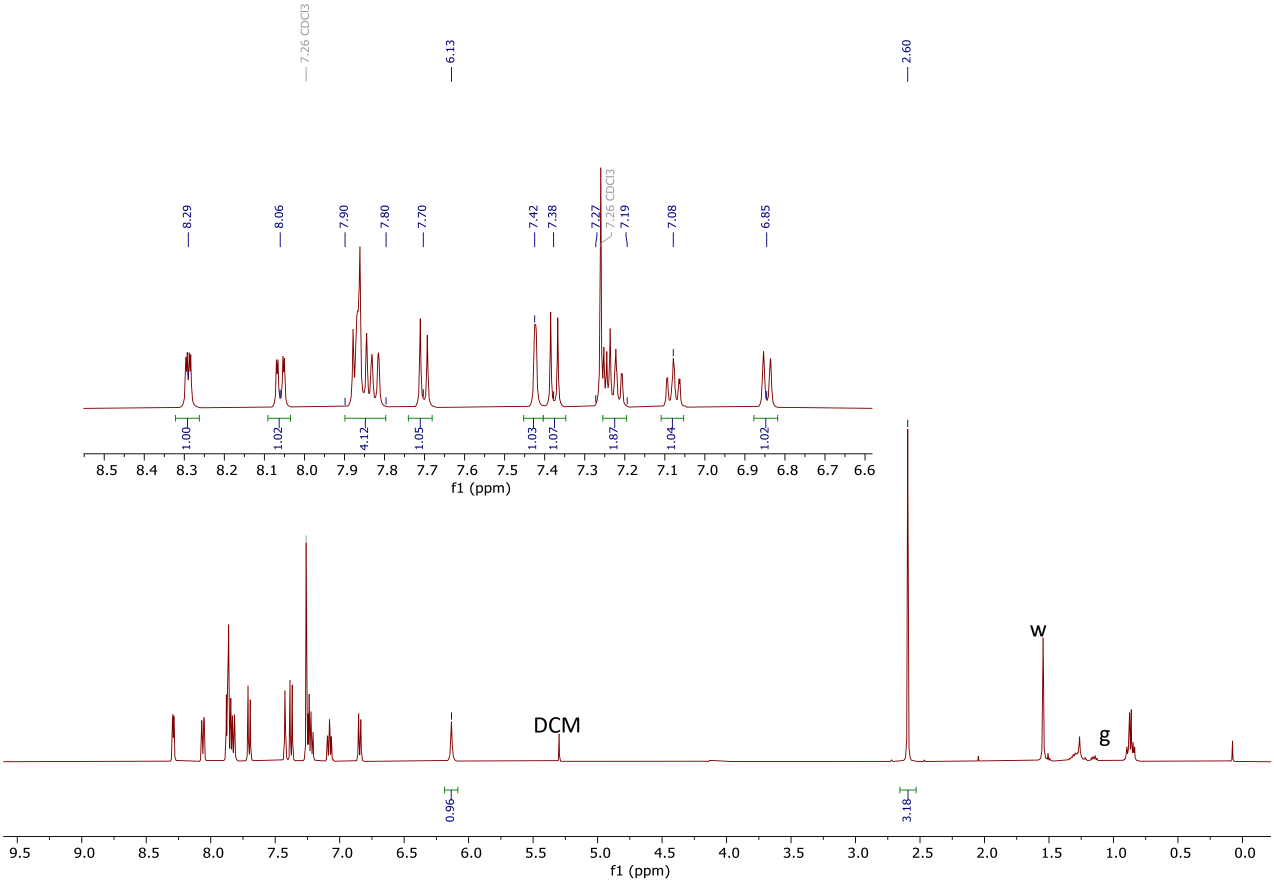

Figure S212: **^{1}H NMR of 34e** (500 MHz, 298 K, $CDCl_3$) (DCM = dichloromethane, w = water, g = grease).

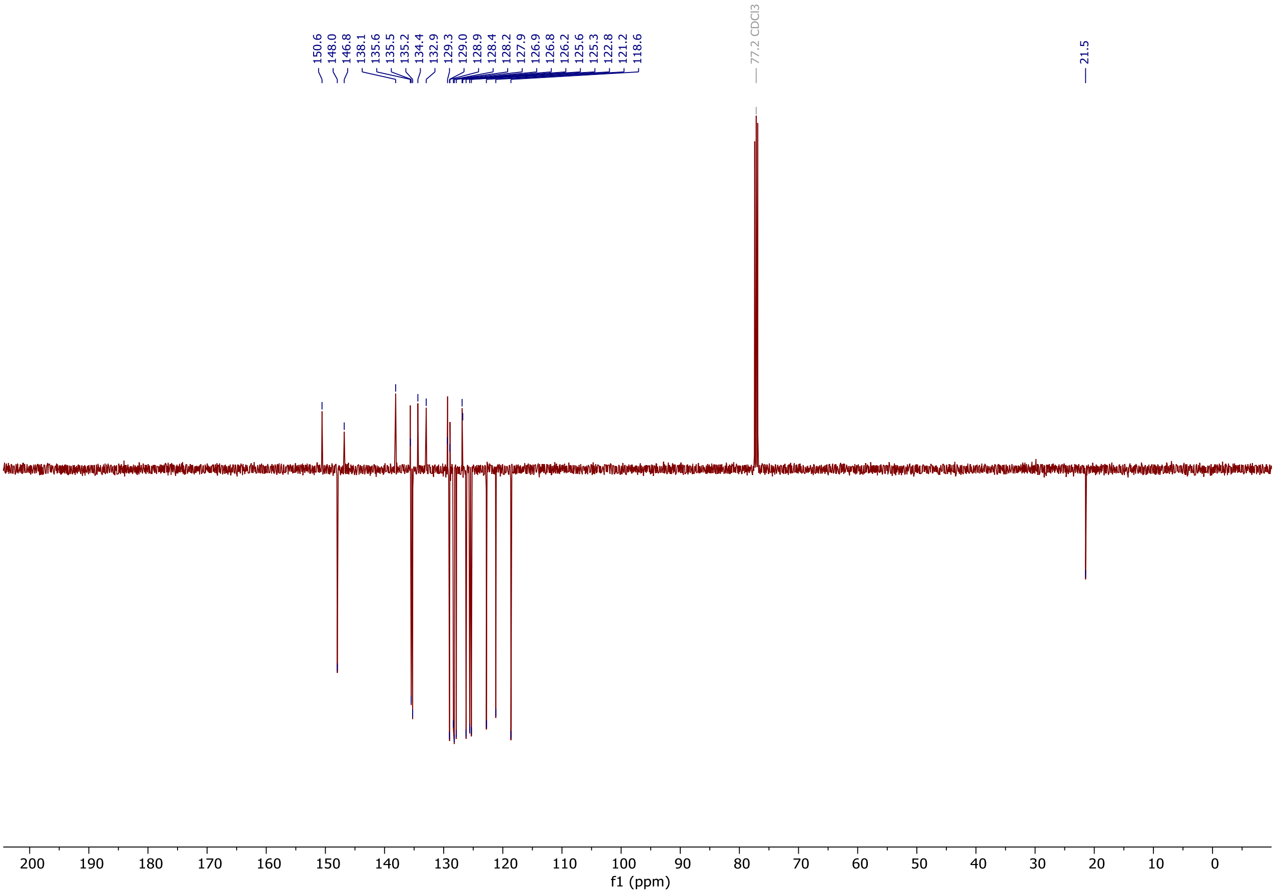

Figure S213: **^{13}C APT NMR of 34e** (126 MHz, 298 K, $CDCl_3$).

Table S2, Entry 11 & 12

Compound (-)-(*R*)-**34f**

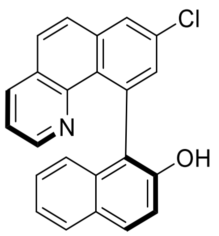

Prepared according to **GP6** starting from 8-chlorobenzo[*h*]quinoline **32c** (21.3 mg, 0.1 mmol, 1.0 equiv.) and diazonaphtalaquinone **33a** (25.5 mg, 0.15 mmol, 1.5 equiv.). After column chromatography on silica gel (heptane:EtOAc 6:1), the two runs gave products (-)-(*R*)-**34f** (6.2 mg, 18%, and 5.9 mg, 17%, respectively) as light pink amorphous solids.

[α]$^{20}_{D}$ -143 (*c* 0.061, $CHCl_3$).

^{1}H NMR (500 MHz, 298 K, $CDCl_3$): δ = 8.32 (dd, *J* = 4.4, 1.8 Hz, 1H), 8.09 (dd, *J* = 8.1, 1.8 Hz, 1H), 8.07 (d, *J* = 2.3 Hz, 1H), 7.89 – 7.81 (m, 3H), 7.77 (d, *J* = 8.8 Hz, 1H), 7.56 (d, *J* = 2.3 Hz, 1H), 7.36 (d, *J* = 8.8 Hz, 1H), 7.30 (dd, *J* = 8.0, 4.3 Hz, 1H), 7.25 – 7.22 (m, 1H), 7.11 (ddd, *J* = 8.3, 6.7, 1.4 Hz, 1H), 6.83 (d, *J* = 8.5 Hz, 1H), 6.01 (s, 1H).

^{13}C{^{1}H} NMR (126 MHz, 298 K, $CDCl_3$): δ = 150.6, 148.4, 146.4, 136.3, 135.7, 135.6, 134.0, 133.8, 133.6, 129.44, 129.35 128.8, 128.1, 128.0, 127.6, 127.5, 127.2, 126.0, 125.5, 125.0, 123.0, 121.8, 118.7.

HRMS (ESI) *m/z*: ([M+H]$^+$) calcd for $C_{23}H_{15}ON^{35}Cl$ 356.0837, found 356.0835 (Δ = -0.58 ppm).

IR (ATR): 3537 (w), 3354 (w br), 3052 (w), 2955 (w), 2922 (w), 2868 (w), 2853 (w), 1620 (m), 1597 (w), 1578 (s), 1563 (w), 1513 (m), 1466 (w), 1419 (w), 1399 (w-m), 1377 (w), 1344 (w), 1322 (w), 1307 (w), 1269 (m), 1230 (m), 1207 (w), 1187 (m), 1136 (m), 1122 (w), 1101 (w), 1060 (w), 1017 (w), 964 (m), 927 (m), 869 (vs), 830 (m), 813 (vs), 767 (m), 747 (s), 728 (vs), 891 (w), 624 (w), 583 (w), 547 (w), 493 (m), 424 (w) cm^{-1}.

HPLC conditions: Lux Cellulose-1 column, *n*-hexane/*i*-PrOH, 90:10 v/v, flow rate 1 mL/min, λ = 254 nm, 25 °C. t_R (minor) = 13.3 min, t_R (major) = 16.1 min, 83:17 *er*.

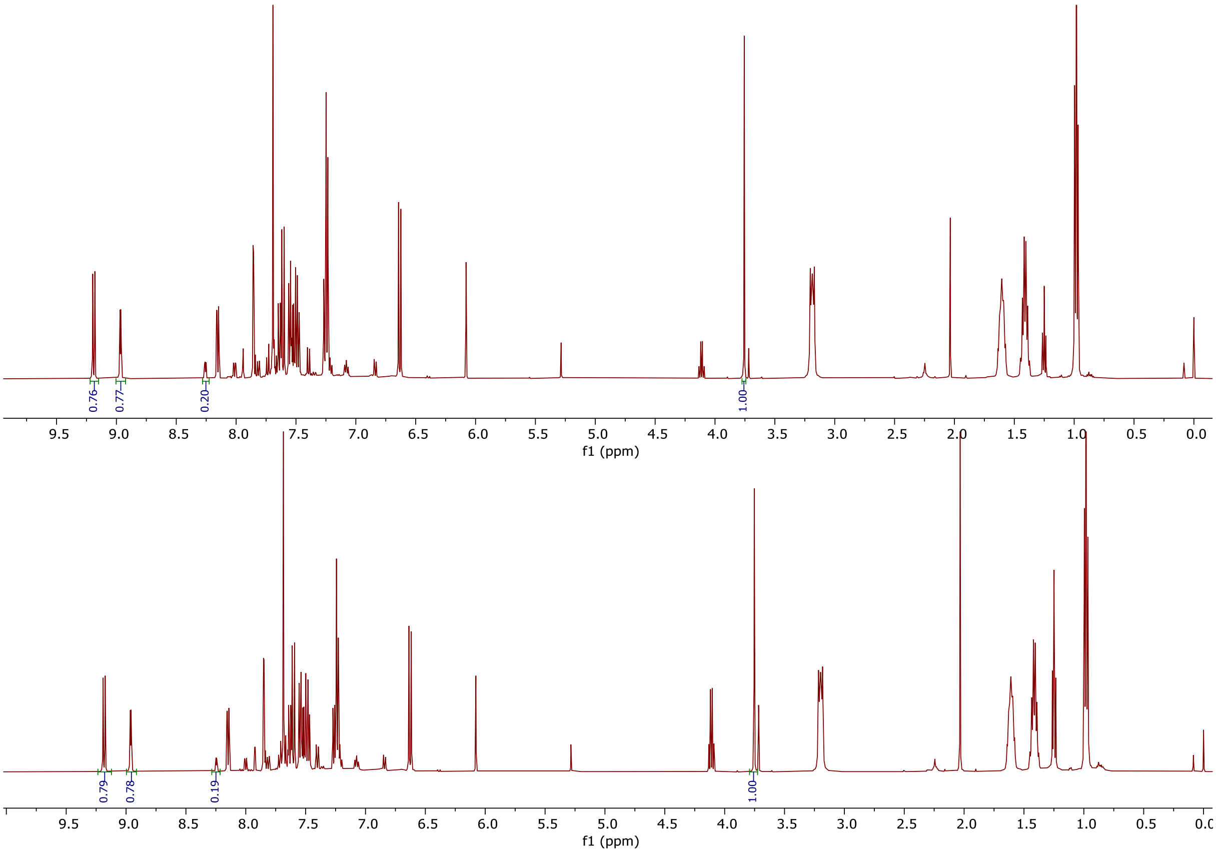

Figure S214: ^{1}H NMR of the crude reaction mixtures **34f** (500 MHz, 298 K, $CDCl_3$).

Chromatogram

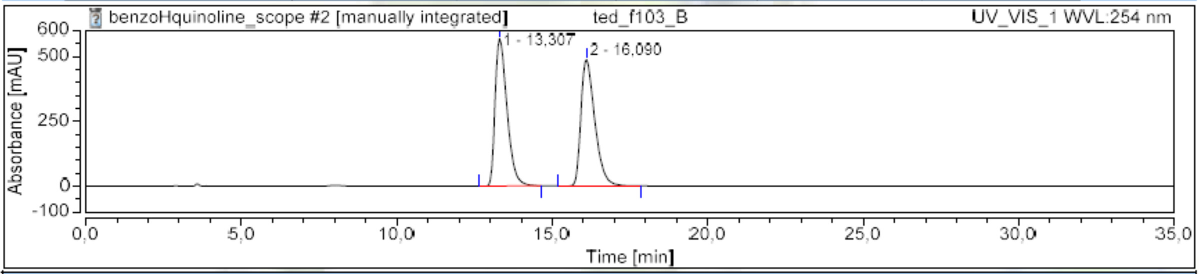

Integration Results

No.	Peak Name	Retention Time min	Area mAU*min	Height mAU	Relative Area %	Relative Height %	Amount n.a.
1		13,307	252,891	568,508	49,88	53,78	n.a.
2		16,090	254,058	488,543	50,12	46,22	n.a.
**Total:**			**506,949**	**1057,051**	**100,00**	**100,00**	

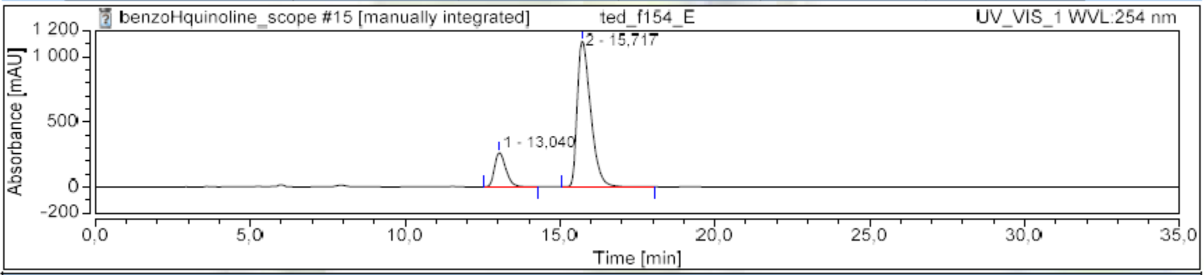

Integration Results

No.	Peak Name	Retention Time min	Area mAU*min	Height mAU	Relative Area %	Relative Height %	Amount n.a.
1		13,040	111,718	262,804	16,57	18,98	n.a.
2		15,717	562,703	1121,715	83,43	81,02	n.a.
**Total:**			**674,421**	**1384,519**	**100,00**	**100,00**	

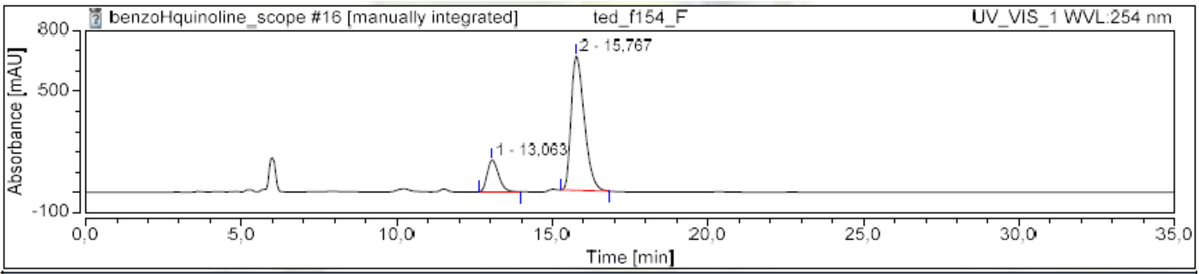

Integration Results

No.	Peak Name	Retention Time min	Area mAU*min	Height mAU	Relative Area %	Relative Height %	Amount n.a.
1		13,063	66,618	157,536	16,91	19,11	n.a.
2		15,767	327,412	666,635	83,09	80,89	n.a.
**Total:**			**394,030**	**824,172**	**100,00**	**100,00**	

Figure S215: HPLC separation of the racemic standard and isolated product of the asymmetric catalysis **34f**.

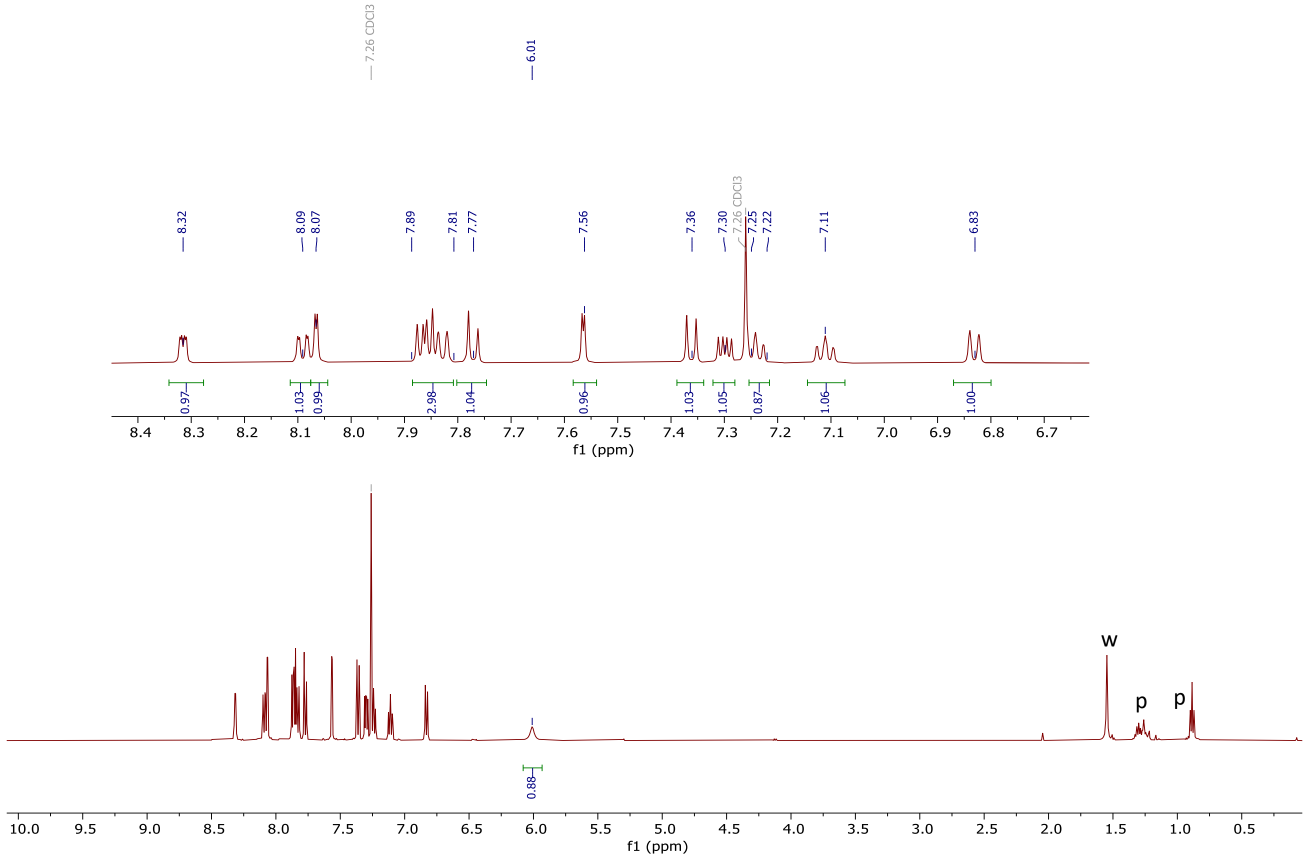

Figure S216: **^{1}H NMR of 34f** (500 MHz, 298 K, $CDCl_3$) (w = water, p = pentane).

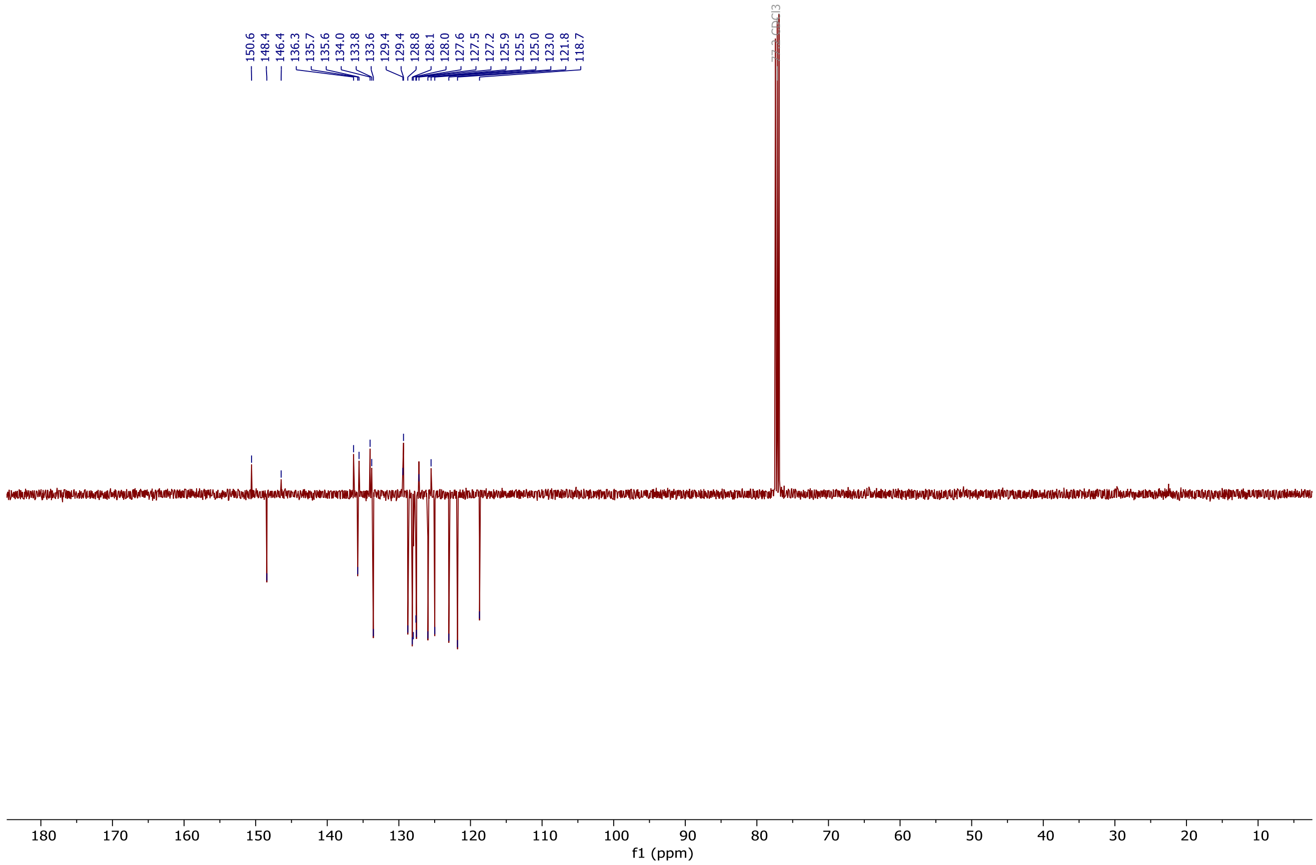

Figure S217: **^{13}C APT NMR of 34f** (126 MHz, 298 K, $CDCl_3$).

Table S2, Entry 13 & 14

Compound (-)-(*R*)-**34g**

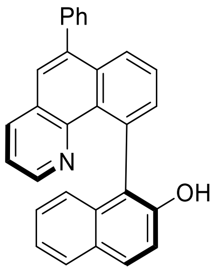

Prepared according **GP6** from 6-phenylbenzo[*h*]quinoline **32d** (25.5 mg, 0.1 mmol, 1.0 equiv.) and diazonaphtalaquinone **33a** (25.5 mg, 0.15 mmol, 1.5 equiv.). After column chromatography on silica gel (heptane:EtOAc 8:1), the two runs gave products (-)-(*R*)-**34g** (22.5 mg, 54%, and 23.8 mg, 60%, respectively) as grey amorphous solids.

[α]$^{20}_{D}$ -181 (*c* 0.042, $CHCl_3$).

NMR in accordance with literature.[10] **^{1}H NMR** (500 MHz, $CDCl_3$): δ = 8.30 (dd, *J* = 4.3, 1.8 Hz, 1H), 8.08 (ddd, *J* = 8.0, 3.3, 1.6 Hz, 2H), 7.86 (d, *J* = 8.8 Hz, 1H), 7.85 – 7.82 (m, 1H), 7.72 (dd, *J* = 8.2, 7.1 Hz, 1H), 7.67 (s, 1H), 7.64 – 7.56 (m, 5H), 7.55 – 7.50 (m, 1H), 7.38 (d, *J* = 8.9 Hz, 1H), 7.29 (dd, *J* = 7.9, 4.3 Hz, 1H), 7.23 (ddd, *J* = 8.0, 6.7, 1.2 Hz, 1H), 7.09 (ddd, *J* = 8.2, 6.7, 1.4 Hz, 1H), 6.85 (dd, *J* = 8.5, 1.0 Hz, 1H), 6.05 (s, 1H).

^{13}C{^{1}H} NMR (126 MHz, $CDCl_3$): δ = 150.3, 147.8, 146.1, 140.5, 140.2, 135.4, 134.4, 134.2, 133.4, 133.2, 131.2, 130.1, 129.2, 128.5, 128.1, 127.79, 127.77, 127.7, 127.0, 126.7, 126.5, 125.5, 125.1, 122.7, 121.7, 118.4.

HPLC conditions: Lux Cellulose-1 column, *n*-hexane/*i*-PrOH, 95:5 v/v, flow rate 1 mL/min, λ = 254 nm, 25 °C. t_R (minor) = 18.7 min, t_R (major) = 20.5 min, 91:9 *er*.

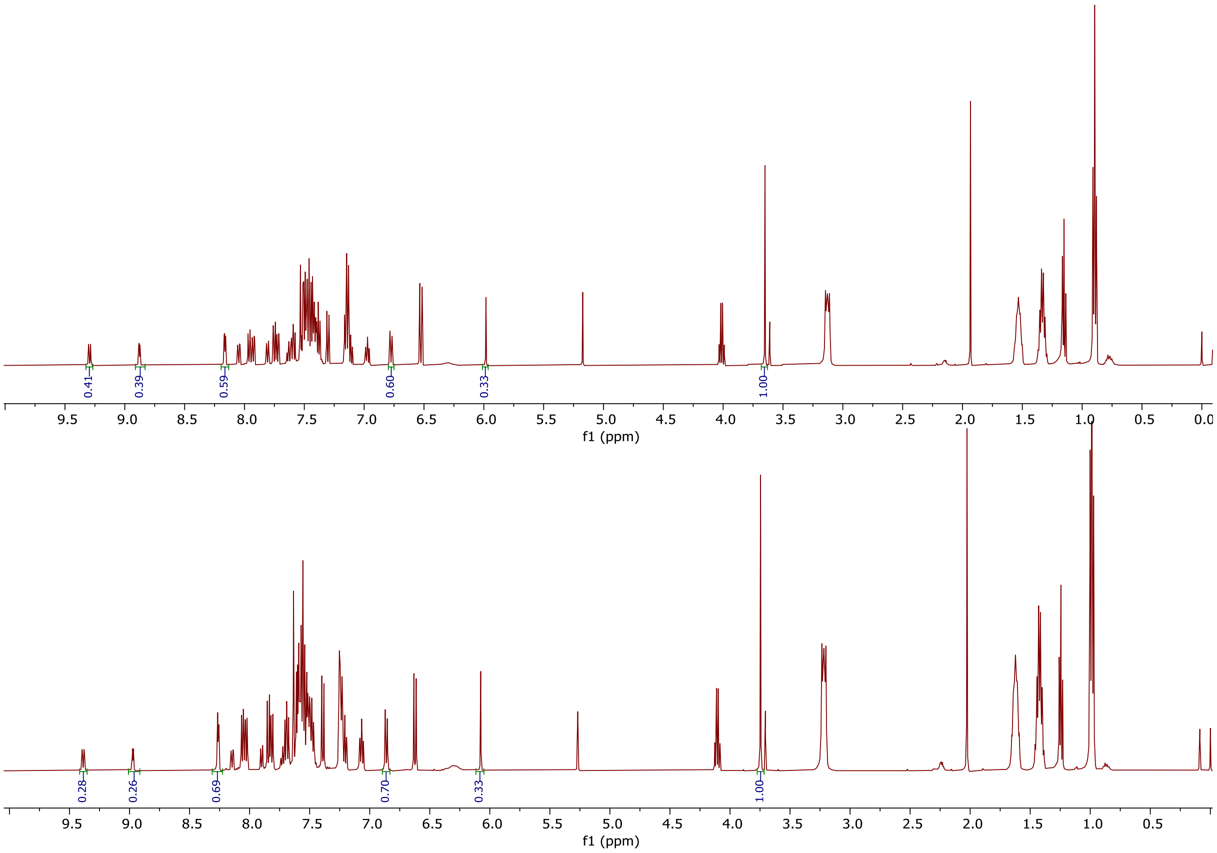

Figure S218: ^{1}H NMR of the crude reaction mixtures **34g** (500 MHz, 298 K, $CDCl_3$).

Chromatogram

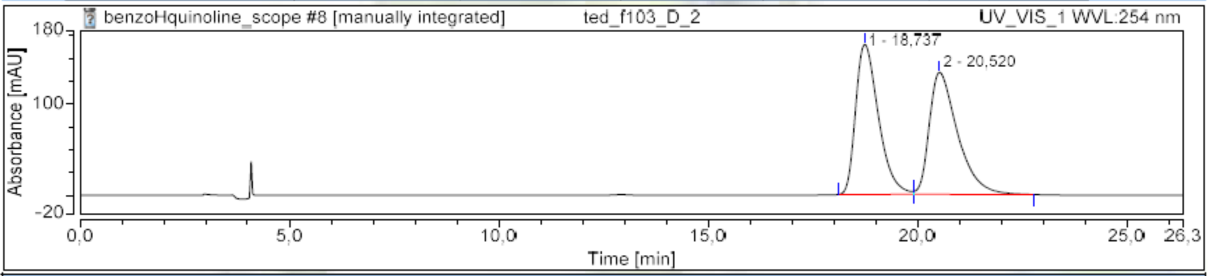

Integration Results

No.	Peak Name	Retention Time min	Area mAU*min	Height mAU	Relative Area %	Relative Height %	Amount n.a.
1		18,737	103,435	164,108	49,77	55,16	n.a.
2		20,520	104,376	133,388	50,23	44,84	n.a.
**Total:**			**207,811**	**297,497**	**100,00**	**100,00**	

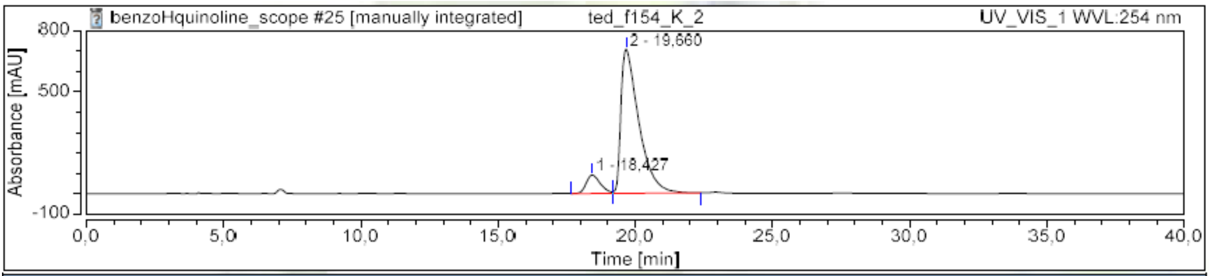

Integration Results

No.	Peak Name	Retention Time min	Area mAU*min	Height mAU	Relative Area %	Relative Height %	Amount n.a.
1		18,427	54,062	90,914	9,18	11,36	n.a.
2		19,660	535,109	709,566	90,82	88,64	n.a.
**Total:**			**589,171**	**800,480**	**100,00**	**100,00**	

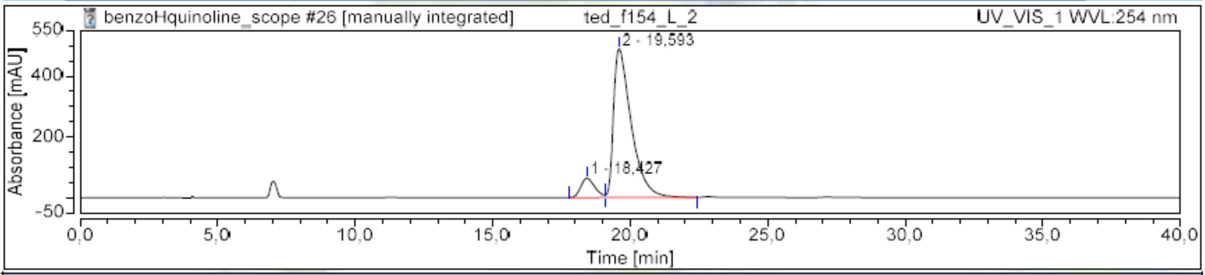

Integration Results

No.	Peak Name	Retention Time min	Area mAU*min	Height mAU	Relative Area %	Relative Height %	Amount n.a.
1		18,427	37,912	63,939	9,42	11,54	n.a.
2		19,593	364,688	489,965	90,58	88,46	n.a.
**Total:**			**402,600**	**553,904**	**100,00**	**100,00**	

Figure S219: HPLC separation of the racemic standard and isolated product of the asymmetric catalysis **34g**.

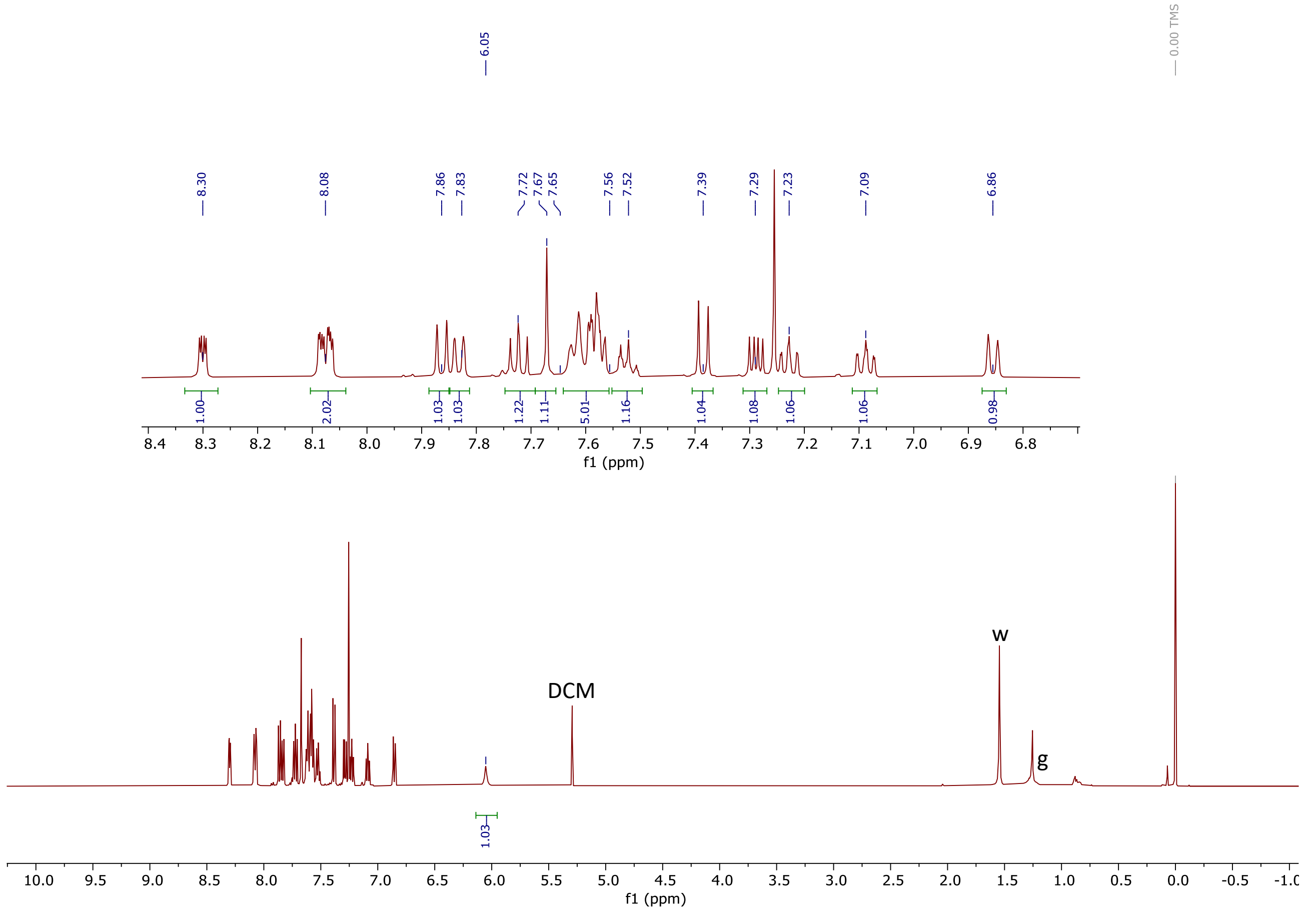

Figure S220: ¹H NMR of 34g (500 MHz, 298 K, $CDCl_3$ + 0.03 % TMS) (DCM = dichloromethane, w = water, g = grease).

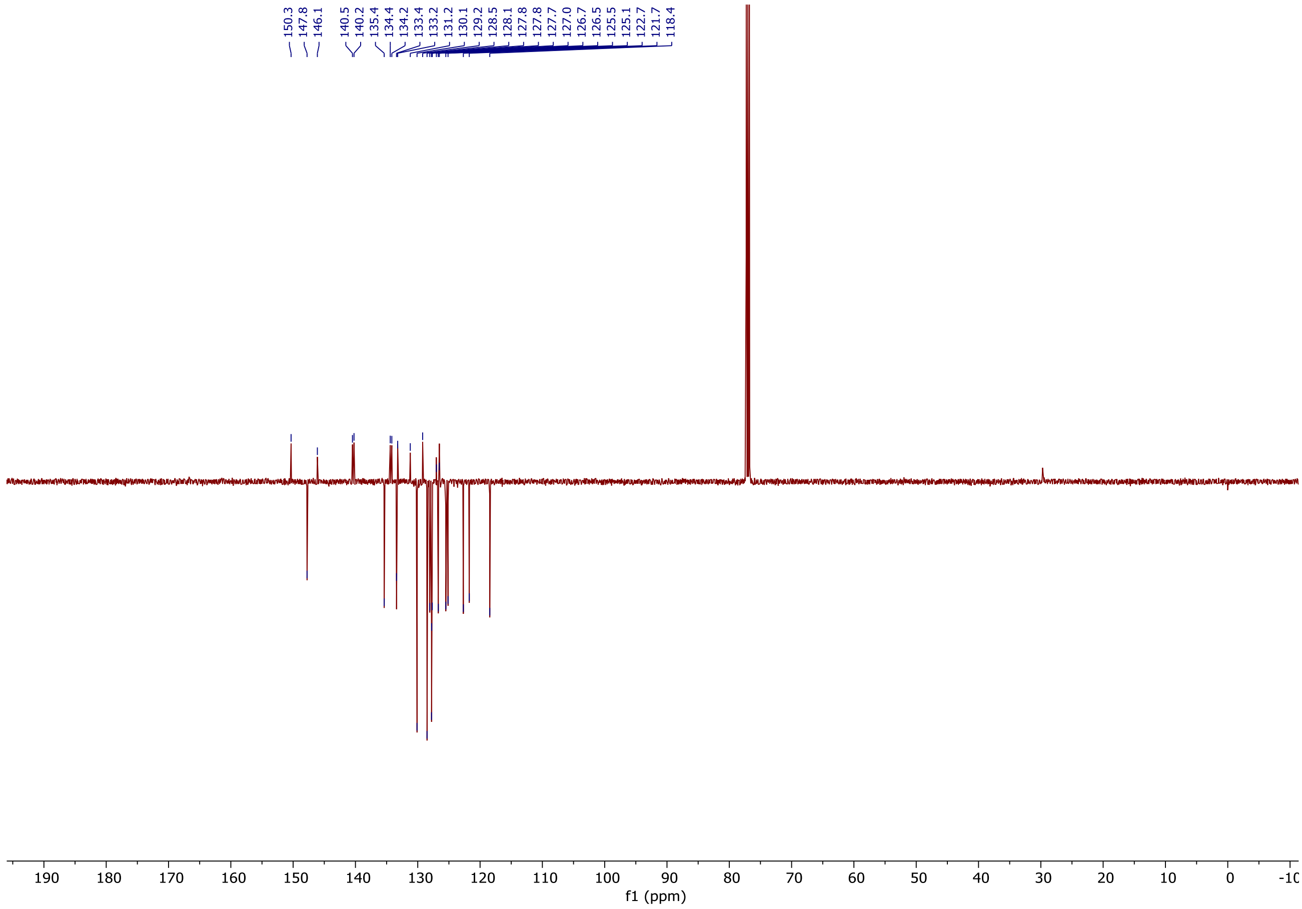

Figure S221: ¹³C APT NMR of 34g (126 MHz, 298 K, $CDCl_3$ + 0.03 % TMS).

Table S2, Entry 15 & 16

Compound (+)-(*R*)-**34h**

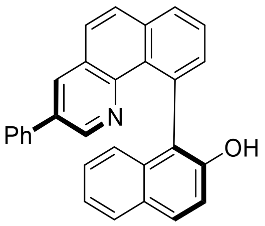

Prepared according to **GP6** starting from 3-phenylbenzo[*h*]quinoline **32d** (25.5 mg, 0.1 mmol, 1.0 equiv.) and diazonaphtalaquinone **33a** (25.5 mg, 0.15 mmol, 1.5 equiv.). After column chromatography on silica gel (heptane:EtOAc 8:1), the two runs gave products (+)-(*R*)-**34h** (9.3 mg, 23%, and 9.5 mg, 24%, respectively) as light pink amorphous solids.

[α]$^{20}_{D}$ +8 (*c* 0.147, $CHCl_3$).

NMR in accordance with literature.[10] **^{1}H NMR** (500 MHz, $CDCl_3$): δ = 8.55 (d, *J* = 2.4 Hz, 1H), 8.23 (d, *J* = 2.4 Hz, 1H), 8.10 (dd, *J* = 8.0, 1.4 Hz, 1H), 7.97 (d, *J* = 8.8 Hz, 1H), 7.90 – 7.77 (m, 4H), 7.61 (dd, *J* = 7.2, 1.4 Hz, 1H), 7.57 (m, 2H), 7.46 – 7.32 (m, 4H), 7.23 (ddd, *J* = 8.1, 6.7, 1.2 Hz, 1H), 7.09 (ddd, *J* = 8.3, 6.7, 1.4 Hz, 1H), 6.89 (d, *J* = 8.5 Hz, 1H), 5.80 (s, 1H).

^{13}C{^{1}H} NMR (126 MHz, $CDCl_3$): δ = 150.4, 147.1, 145.7, 137.6, 135.4, 134.3, 134.1, 133.7, 132.9, 132.9, 130.9, 129.5, 129.3, 129.1, 129.0, 128.3, 128.2, 128.1, 127.9, 127.4, 127.1, 126.5, 126.5, 125.7, 125.2, 122.9, 118.4.

HPLC conditions: Lux Cellulose-1 column, *n*-hexane/*i*-PrOH, 90:10 v/v, flow rate 1 mL/min, λ = 254 nm, 25 °C. t_R (minor) = 18.0 min, t_R (major) = 24.1 min, 82.5:17.5 *er*.

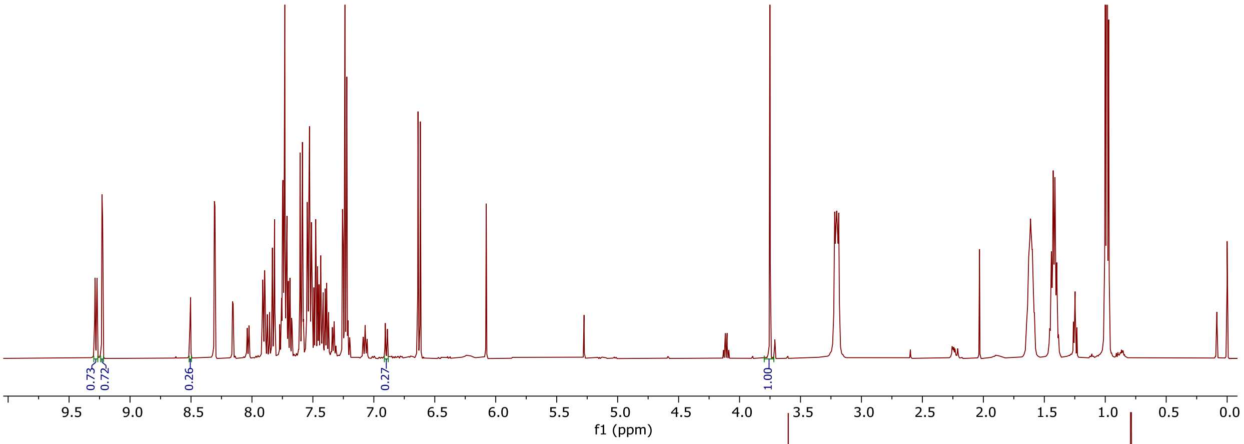

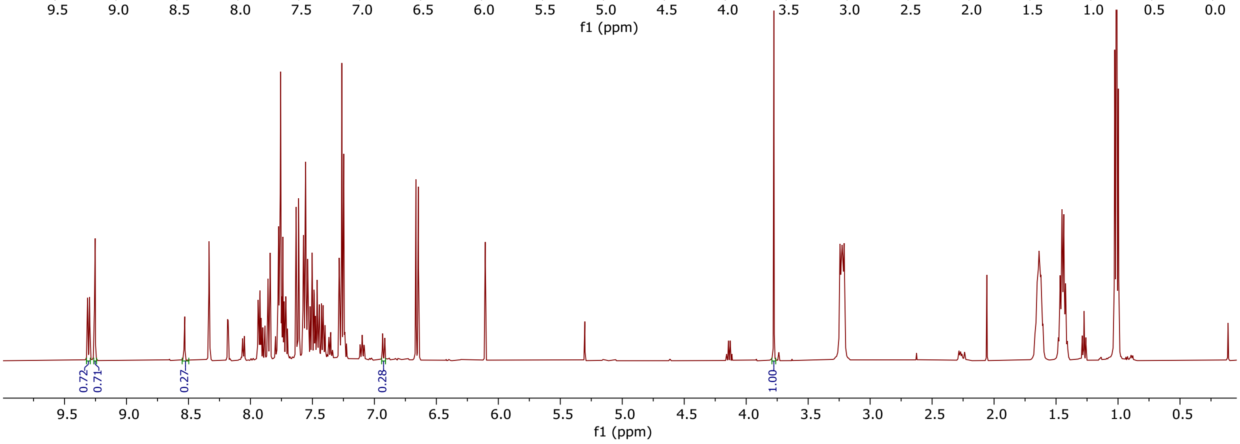

Figure S222: ^{1}H NMR of the crude reaction mixtures **34h** (500 MHz, 298 K, $CDCl_3$).

Chromatogram

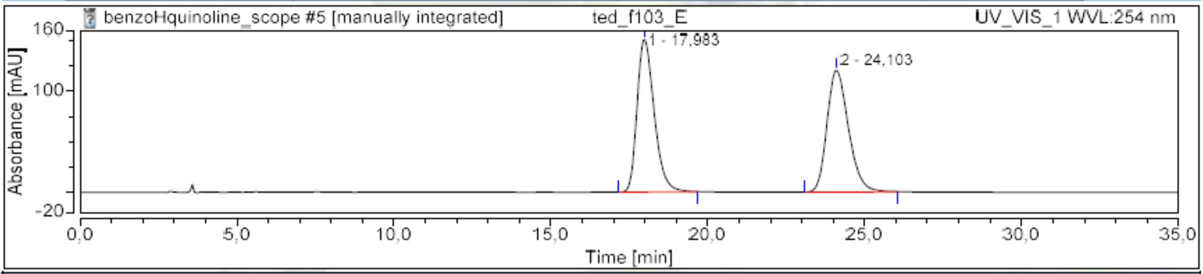

Integration Results

No.	Peak Name	Retention Time min	Area mAU*min	Height mAU	Relative Area %	Relative Height %	Amount n.a.
1		17,983	95,604	151,384	50,01	55,65	n.a.
2		24,103	95,556	120,643	49,99	44,35	n.a.
**Total:**			**191,160**	**272,028**	**100,00**	**100,00**	

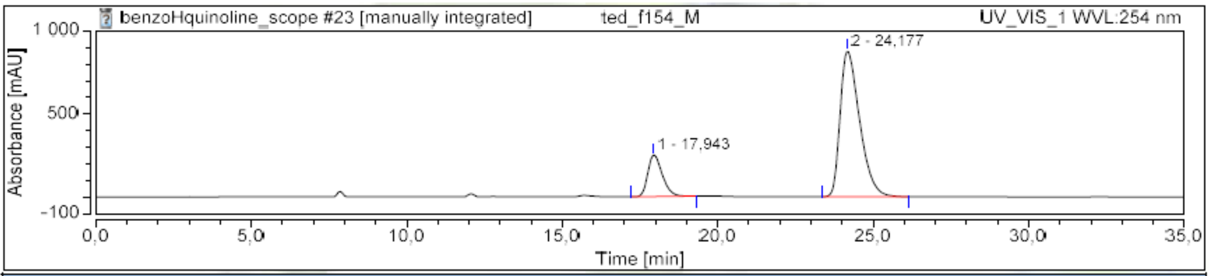

Integration Results

No.	Peak Name	Retention Time min	Area mAU*min	Height mAU	Relative Area %	Relative Height %	Amount n.a.
1		17,943	135,421	250,360	17,56	22,22	n.a.
2		24,177	635,689	876,235	82,44	77,78	n.a.
**Total:**			**771,110**	**1126,595**	**100,00**	**100,00**	

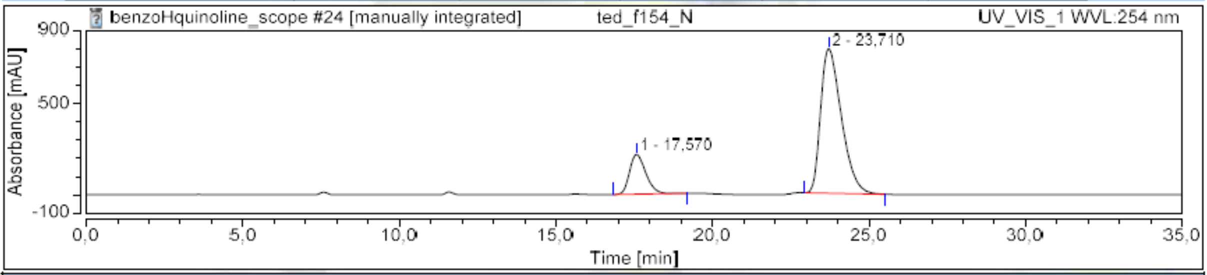

Integration Results

No.	Peak Name	Retention Time min	Area mAU*min	Height mAU	Relative Area %	Relative Height %	Amount n.a.
1		17,570	129,737	217,128	17,58	21,50	n.a.
2		23,710	608,040	792,701	82,42	78,50	n.a.
**Total:**			**737,777**	**1009,829**	**100,00**	**100,00**	

Figure S223: HPLC separation of the racemic standard and isolated product of the asymmetric catalysis **34h**.

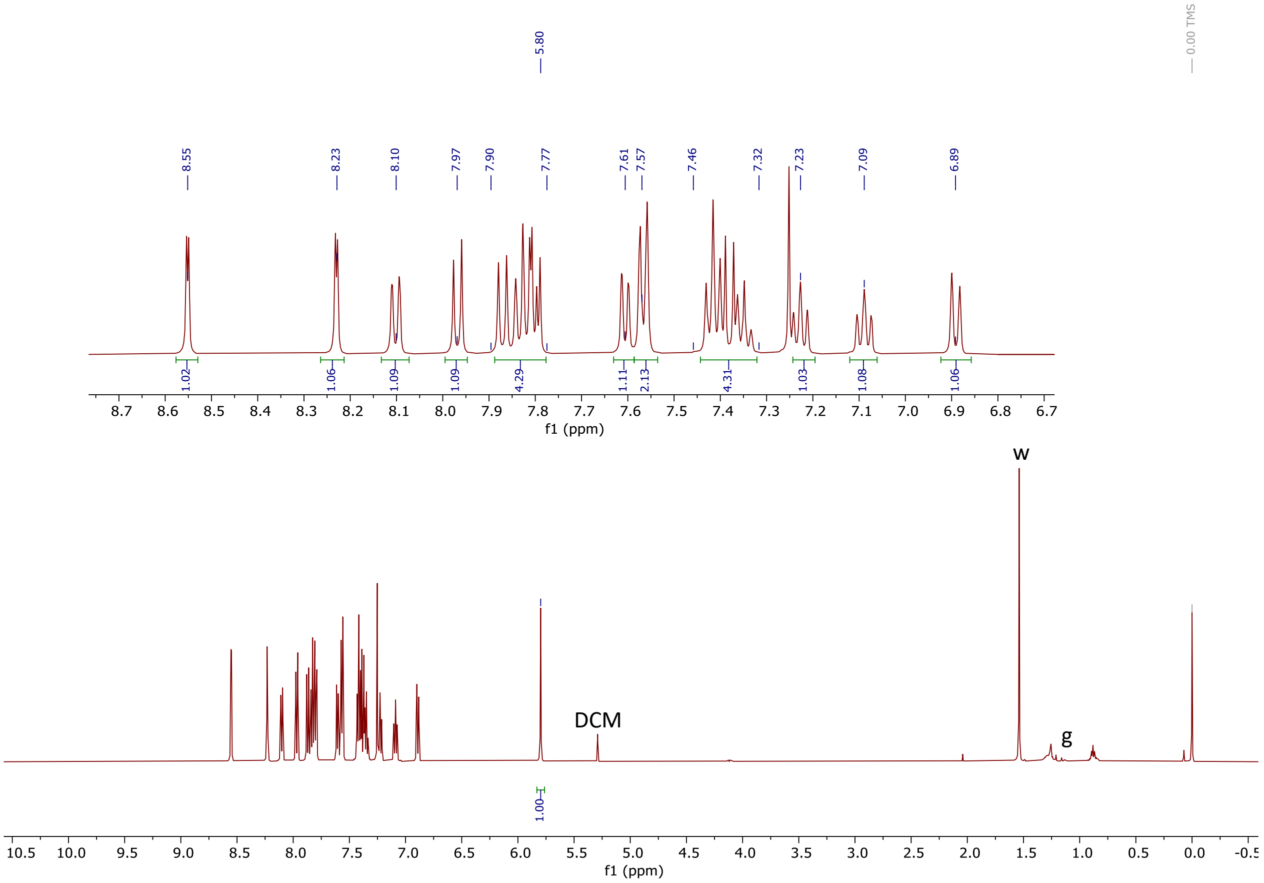

Figure S224: ¹H NMR of 34h (500 MHz, 298 K, $CDCl_3$ + 0.03 % TMS) (DCM = dichloromethane, w = water, g = grease).

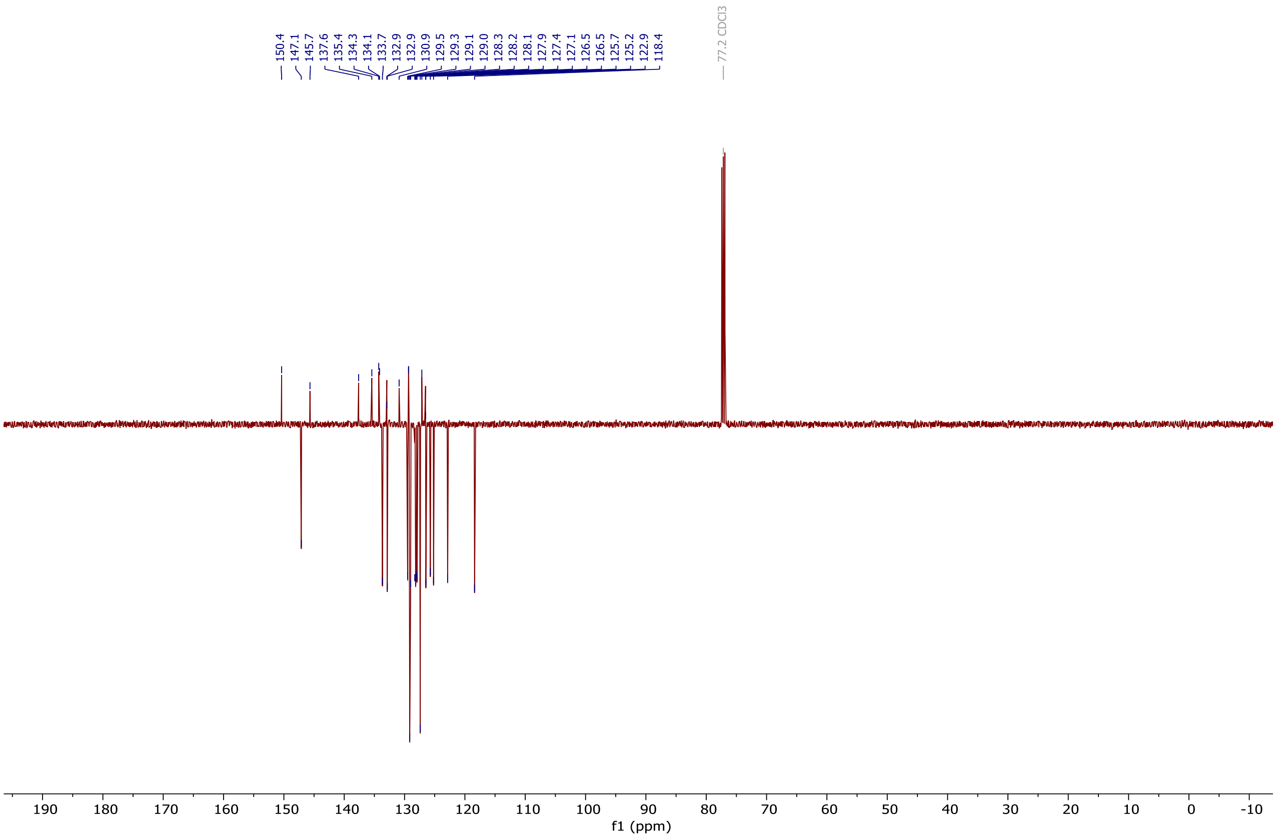

Figure S225: ¹³C APT NMR of 34h (126 MHz, 298 K, $CDCl_3$ + 0.03 % TMS).

DFT calculations and buried volumes

All calculations were performed using the Gaussian 16 software package.[11]

For the comparison of relative energies, geometries of the two possible stereoisomers of the first cyclization product, ketones (*M*,*R*,*R*)-**13** and (*P*,*R*,*R*)-**13**, differing in their helicity, and those of all four possible diastereoisomers of Rh complex **22a**, were optimized by DFT (B3LYP[12]/def2-TZVP[13]/GD3[14]). In all cases, vibrational analysis confirmed absence of negative frequencies. Thermochemical analysis (298 K, 1 atm) provided the free energy differences. Cartesian coordinates of the final geometries of compounds (*M*,*R*,*R*)-**13**, (*P*,*R*,*R*)-**13**, (*M,R,R,R*$_p$)-**22a**, (*M,R,R,S*$_p$)-**22a**, (*P,R,R,R*$_p$)-**22a** and (*P,R,R,S*$_p$)-**22a**, are listed below.

For the simulation of ECD spectra of the Rh complexes and proligand, their geometries were first optimized at the PBE0[15]/def2-SVP[13] (**25a**) or def2-TZVP (**14a** and **22a**)/GD3/PCM[16,17] level of theory using built-in solvent parameters for tetrahydrofuran. In the case of the dimeric complex **25a**, rotation of the heliceno-indenyl ligands around their η5 coordination bonds to rhodium gives rise to conformers differing in the relative position of the two helicene units. The geometry with minimal steric hindrance, where the two units are oriented away from each other, was selected as the starting point for the optimization. In the other cases, conformational flexibility was neglected due to the inherent rigidity of the helical backbone. Absence of negative frequencies was always confirmed by vibrational analysis. The ECD spectral traces were simulated by extracting vertical excitations from TD-DFT calculations of 100 lowest-lying singlet states (200 states were necessary in the case of **25a**) at the same level of theory and applying gaussian broadening (0.5 eV) to the resulting line spectrum (Energy vs Rotational strength). Cartesian coordinates and a full list of TD-DFT transitions are given below.

Percent buried volumes[18,19] for two chiral cyclopentadiene type ligands were calculated from their respective Rh complexes (*M, R, R, R*$_p$)-**22a** (geometry taken from B3LYP DFT optimization as described above) and (*R*)-**28** (geometry taken from crystallographic data reported in literature[20]). Quadrant-resolved percent buried volumes and topographic steric maps were generated using ChimeraX[21,22] software, taking advantage of the algorithm implemented in the SEQCROW[23,24] plugin with the following parameters: Bondi radii were scaled by the factor of 1.17[25], integration sphere with the radius of 3.5 Å or 7.0 Å was centred on the rhodium atom, 99 radial and 2702 angular points were used for Lebedev integration, hydrogen atoms were excluded, topographic maps were plotted for 1000 points on each of the x and y axes using a 20-color scale in the z direction.

(*M,R,R*)-**13** (B3LYP/def2-TZVP/GD3):

SCF: -1730.611120

C	-2.430033	-2.600202	-0.661311
C	-2.389592	-1.299285	-0.095631
O	-1.284242	-3.131928	-1.161261
C	-1.130583	-0.543473	-0.226109
C	-0.099269	-2.803771	-0.396089
C	0.047030	-1.303207	-0.269250
C	-3.593007	-3.354246	-0.720505
C	-4.759947	-2.872162	-0.124371
C	-3.532140	-0.898786	0.588378
C	-4.699891	-1.672758	0.550297
C	-1.031507	0.863262	-0.308949
C	0.219500	1.460169	-0.094418
C	1.303502	-0.700670	-0.164885
C	1.382848	0.691244	0.011028
C	-2.142969	1.767602	-0.658945
C	-2.066546	3.095567	-0.214954
C	0.276579	2.973716	-0.055678
O	-0.957741	3.520135	0.456755
C	-3.240813	1.410380	-1.450004
C	-4.267148	2.308211	-1.698277
C	-3.102583	3.994701	-0.433346
C	-4.212376	3.593253	-1.163356
C	-3.797222	0.274838	1.458179
C	-5.060609	0.246392	1.903607
C	-5.742960	-0.970891	1.358737
H	-3.555317	-4.324200	-1.197181
H	-5.678848	-3.444160	-0.145256
H	-3.295084	0.413165	-1.862031
H	-5.111552	2.004816	-2.302746

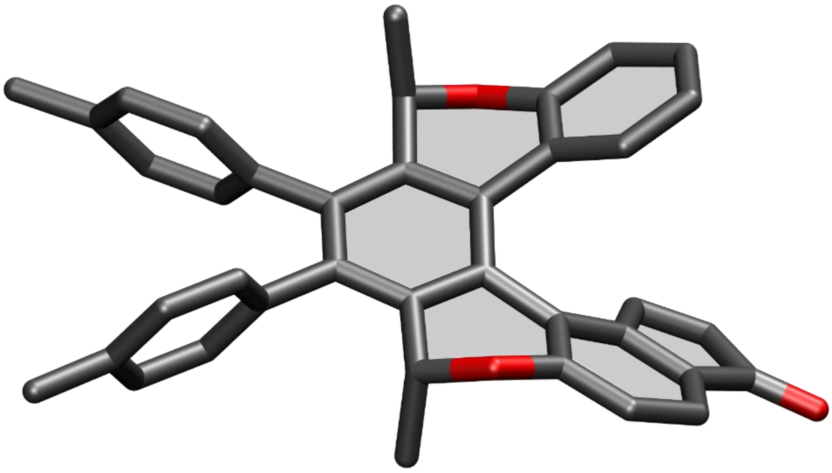

H	-3.007641	5.000811	-0.047110
H	-5.020174	4.291733	-1.340552
H	-3.057460	1.022820	1.695507
H	-5.546298	0.954145	2.556971
O	-6.889798	-1.314210	1.540396
H	1.016263	3.293332	0.673695
C	4.278423	2.414886	1.742307
C	3.039043	1.832558	1.509593
C	5.227428	2.520330	0.725726
C	4.889453	2.026612	-0.534685
C	3.652402	1.442767	-0.769856
C	2.706504	1.335827	0.249406
C	6.584846	3.117321	0.986015
C	4.437185	-2.515085	0.955052
C	3.266244	-1.770105	0.983071
C	4.931129	-3.039886	-0.239605
C	4.211859	-2.788653	-1.407893
C	3.039770	-2.043738	-1.383000
C	2.547824	-1.522924	-0.186341
C	6.185714	-3.871946	-0.261229
H	7.306750	2.339573	1.251710
H	6.555781	3.831754	1.809790
H	6.970124	3.630863	0.103774
H	5.958594	-4.924280	-0.067372
H	6.893006	-3.545709	0.502846
H	6.683329	-3.818478	-1.230483
H	4.511503	2.791691	2.731562
H	2.319993	1.756460	2.316612
H	5.605742	2.098333	-1.345099
H	3.417773	1.057474	-1.753847
H	4.979819	-2.686772	1.877580
H	2.910767	-1.360944	1.920216
H	4.576318	-3.175097	-2.352762
H	2.504395	-1.851757	-2.305317
H	0.708162	-3.197929	-1.007206
C	-0.107688	-3.525478	0.949071
C	0.614481	3.586045	-1.413762
H	-0.898296	-3.142576	1.595326
H	-0.261136	-4.594568	0.797903
H	0.850416	-3.373267	1.446281
H	0.601222	4.674350	-1.343846
H	-0.102667	3.271639	-2.172872
H	1.609460	3.264311	-1.721309

(*P,R,R*)-**13** (B3LYP/def2-TZVP/GD3):

SCF: -1730.597122

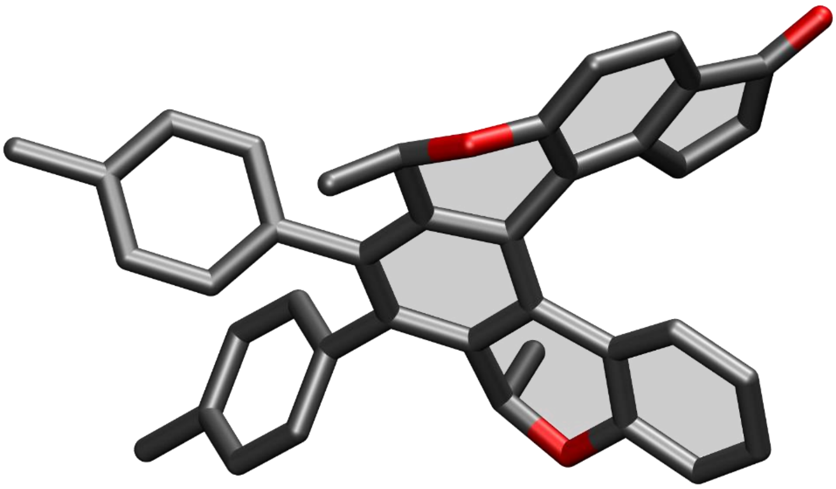

C	2.500788	-2.607490	-0.633836
C	2.396353	-1.306132	-0.093665
O	1.471828	-3.063051	-1.387075
C	1.178804	-0.537106	-0.404684
C	0.149257	-2.778533	-0.866612
C	-0.003451	-1.294185	-0.526057
C	3.610893	-3.416941	-0.443857
C	4.651590	-2.974837	0.376604
C	3.399306	-0.932124	0.794127
C	4.514138	-1.756265	1.003342
C	1.108751	0.874520	-0.466739
C	-0.141656	1.473716	-0.230581
C	-1.249614	-0.681703	-0.343554
C	-1.304678	0.710171	-0.132170
C	2.219180	1.768249	-0.866437
C	1.973351	3.151685	-0.954834
C	-0.163872	2.948296	0.106943
O	0.766311	3.704838	-0.675947
C	3.460941	1.325761	-1.355340
C	4.425885	2.198417	-1.824371
C	2.943389	4.038365	-1.420161
C	4.172656	3.567933	-1.842905
C	3.555805	0.266305	1.653518
C	4.705365	0.200375	2.339155
C	5.413668	-1.071244	1.981379
H	3.632151	-4.387652	-0.919869
H	5.529015	-3.584775	0.549729

H	3.671786	0.270010	-1.386677
H	5.369549	1.809248	-2.182710
H	2.695064	5.090872	-1.451798
H	4.923586	4.261753	-2.198677
H	2.828375	1.060332	1.706404
H	5.094803	0.914504	3.047804
O	6.480972	-1.459864	2.399844
H	-1.127476	3.382210	-0.143092
C	-4.404904	1.762876	1.774354
C	-3.182177	1.183136	1.463702
C	-5.096937	2.533068	0.839701
C	-4.521482	2.700791	-0.420104
C	-3.298168	2.123333	-0.732841
C	-2.608376	1.354394	0.204613
C	-6.409164	3.183838	1.187983
C	-3.841859	-3.135091	0.909632
C	-2.673506	-2.392141	0.794186
C	-4.883860	-2.983390	-0.003915
C	-4.715224	-2.058901	-1.037081
C	-3.551935	-1.313695	-1.151929
C	-2.506389	-1.474595	-0.241994
C	-6.164310	-3.762856	0.133565
H	-6.963462	2.595686	1.920874
H	-6.249440	4.176415	1.619137
H	-7.038697	3.309096	0.305858
H	-6.018628	-4.671247	0.719263
H	-6.930394	-3.165820	0.636757
H	-6.563572	-4.047076	-0.841548
H	-4.831455	1.608318	2.758798
H	-2.672266	0.575316	2.200504
H	-5.040344	3.286569	-1.170198
H	-2.874780	2.260116	-1.720808
H	-3.944748	-3.842144	1.724707
H	-1.880027	-2.523930	1.519835
H	-5.508208	-1.921557	-1.763267
H	-3.445220	-0.604716	-1.962071
H	0.022236	-3.390413	0.035799
C	-0.771516	-3.289835	-1.968224
C	0.110806	3.177562	1.593264
H	-0.743885	-2.615529	-2.824568
H	-1.797039	-3.391045	-1.631994
H	-0.408820	-4.267898	-2.283271
H	-0.650334	2.679630	2.194826
H	1.088484	2.778229	1.867852
H	0.093764	4.245731	1.812760

(*M,R,R,R*$_p$)-**22a** (B3LYP/def2-TZVP/GD3):

SCF: -2118.160124

C	-1.344196	-0.771285	-2.711196
C	-1.211375	0.172846	-1.695478
O	-0.217032	-1.350518	-3.231611
C	0.151860	0.532731	-1.272485
C	0.739001	-1.701330	-2.208524
C	1.123994	-0.471462	-1.411246
C	-2.585398	-1.198077	-3.204551
C	-3.753295	-0.767025	-2.614035
C	-2.406744	0.585038	-1.052090
C	-3.675222	0.099544	-1.512014
C	0.540765	1.771593	-0.716654
C	1.761436	1.848639	-0.028675
C	2.376535	-0.356243	-0.805156
C	2.665533	0.783291	-0.033367
C	-0.222197	3.024136	-0.851622
C	-0.017968	4.023260	0.110954
C	2.102443	3.157584	0.653360
O	0.907945	3.836923	1.095286
C	-1.108951	3.301736	-1.897110
C	-1.829124	4.485654	-1.931087
C	-0.754390	5.201152	0.102000
C	-1.672010	5.425386	-0.914913
C	-2.670759	1.381002	0.129986
C	-4.079761	1.491116	0.289962
C	-4.704797	0.602785	-0.617712

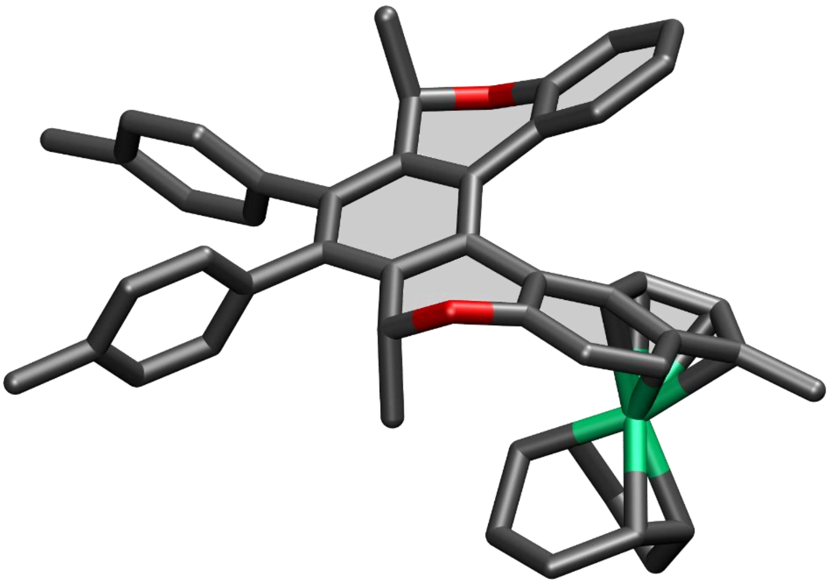

H	-2.590817	-1.905077	-4.023173
H	-4.713629	-1.117139	-2.970341
H	-1.244644	2.570759	-2.681296
H	-2.516868	4.672921	-2.745190
H	-0.577752	5.930263	0.881627
H	-2.243547	6.344801	-0.929402
H	-1.939286	1.940681	0.684945
H	-4.585749	2.102913	1.020252
Rh	-3.389460	-0.606978	0.792127
C	-2.379210	-0.728818	2.666973
C	-1.749850	-1.613536	1.769178
C	-4.791987	-1.901889	1.803855
C	-4.176171	-2.603006	0.752716
C	-3.349759	-1.132401	3.757944
H	-1.881155	0.219263	2.848628
C	-1.917462	-3.127463	1.813534
H	-0.832276	-1.260773	1.308118
H	-5.787956	-1.513990	1.615198
C	-4.440475	-2.106917	3.271968
H	-4.746532	-2.705441	-0.166714
C	-3.055135	-3.612602	0.895468
C	-6.174010	0.401341	-0.806925
H	2.638229	2.961099	1.578444
C	5.155777	0.800190	2.821573
C	3.962436	0.747661	2.112717
C	6.375739	0.969458	2.167193
C	6.358005	1.090127	0.777401
C	5.166954	1.037012	0.066555
C	3.948427	0.863955	0.722677
C	7.673377	0.988219	2.930708
C	4.592771	-3.309913	0.048974
C	3.640015	-2.305534	0.150957
C	5.320689	-3.498101	-1.126247
C	5.063236	-2.640928	-2.195908
C	4.110155	-1.635326	-2.096647
C	3.382330	-1.451656	-0.921255
C	6.332202	-4.607329	-1.243331
H	8.109669	-0.013723	2.980815
H	7.528994	1.333543	3.955383
H	8.407510	1.638947	2.452946
H	5.856881	-5.535400	-1.574200
H	6.812172	-4.811353	-0.284880
H	7.110241	-4.362827	-1.967906
H	5.137680	0.705658	3.901327
H	3.027018	0.608932	2.641769
H	7.290750	1.226348	0.242073
H	5.180092	1.122632	-1.012458
H	4.777674	-3.955482	0.899982
H	3.097483	-2.169849	1.077954
H	5.620351	-2.756536	-3.118617
H	3.935151	-0.974387	-2.937233
H	-6.409851	-0.646201	-1.005592
H	-6.726566	0.707059	0.081726
H	-6.545341	0.985482	-1.654602
H	1.599393	-2.052257	-2.772620
C	0.208044	-2.838827	-1.337485
C	2.946207	4.076552	-0.228718
H	-0.657903	-2.512878	-0.762872
H	-0.082192	-3.683286	-1.964006
H	0.984815	-3.163037	-0.644831
H	3.130579	5.020007	0.286721
H	2.440087	4.282061	-1.172713
H	3.902188	3.599119	-0.443844
H	-2.810341	-1.556577	4.615617
H	-3.828240	-0.219590	4.119216
H	-0.982639	-3.601592	1.508232
H	-2.092227	-3.447504	2.842512
H	-4.125710	-3.140300	3.428389
H	-5.338447	-1.973369	3.878262
H	-3.444520	-4.580663	1.238312
H	-2.650469	-3.784537	-0.104419

(*M,R,R,S*$_p$)-**22a** (B3LYP/def2-TZVP/GD3):

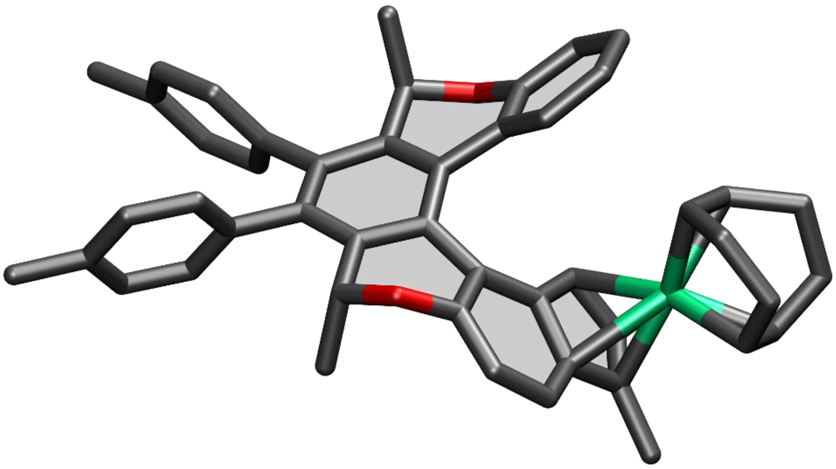

SCF: -2118.1434347			
C	-0.646007	-2.514168	-0.778617
C	-0.635623	-1.207856	-0.287230
O	0.425953	-2.949629	-1.516416
C	0.626130	-0.442131	-0.445869
C	1.665453	-2.692620	-0.828886
C	1.803790	-1.211117	-0.541122
C	-1.630332	-3.462964	-0.467245
C	-2.588601	-3.177196	0.478186
C	-2.589720	-1.906091	1.069844
C	-1.687256	-0.879093	0.619021
C	0.772178	0.962037	-0.373070
C	2.019058	1.505305	-0.021841
C	3.063785	-0.647160	-0.323523
C	3.162497	0.707389	0.041110
C	-0.256038	1.933086	-0.775259
C	-0.172264	3.236359	-0.264383
C	2.106069	3.003965	0.197877
O	0.830723	3.542035	0.610463
C	-1.208733	1.680913	-1.763944
C	-2.068261	2.680878	-2.196410
C	-1.065446	4.226510	-0.646286
C	-2.017752	3.945171	-1.617407
C	-3.393216	-1.369683	2.155360
C	-2.937138	-0.037753	2.383236
C	-2.009638	0.307371	1.379820
H	-1.548100	-4.442950	-0.916930
H	-3.299870	-3.930146	0.792496
H	-1.249754	0.695533	-2.208728
H	-2.790709	2.471608	-2.972860
H	-0.968788	5.211865	-0.210126
H	-2.701830	4.719247	-1.941254
C	-4.242874	-2.188891	3.074838
H	-3.277124	0.612332	3.174798
Rh	-4.152616	-0.213126	0.460733
C	-6.179105	-0.176136	1.078010
C	-6.028574	-1.201064	0.117399
C	-4.829525	1.463408	-0.752282
C	-4.486507	0.383291	-1.577336
C	-6.804104	1.179224	0.821936
H	-6.227159	-0.485609	2.117415
C	-6.539449	-1.095902	-1.313111
H	-5.941697	-2.213395	0.498254
H	-4.092084	2.246855	-0.635456
C	-6.267240	1.860630	-0.450480
H	-3.508405	0.412502	-2.040811
C	-5.457398	-0.555941	-2.263249
H	2.754182	3.209294	1.046222
C	5.976484	2.149123	2.128283
C	4.741428	1.634679	1.753645
C	6.995480	2.329040	1.193677
C	6.732010	1.981067	-0.131802
C	5.499731	1.466297	-0.508244
C	4.482373	1.283330	0.428966
C	8.347352	2.850228	1.603503
C	6.193704	-2.579578	0.604607
C	5.024651	-1.839348	0.710510
C	6.684097	-2.981786	-0.638232
C	5.962702	-2.611821	-1.772889
C	4.791718	-1.871644	-1.669250
C	4.301818	-1.474208	-0.425332
C	7.937048	-3.809651	-0.747167
H	9.038964	2.025753	1.800100
H	8.285713	3.449778	2.512693
H	8.791169	3.466210	0.819604
H	7.708687	-4.876078	-0.661593
H	8.646431	-3.564388	0.044868
H	8.433212	-3.658100	-1.706857
H	6.150706	2.411680	3.165508
H	3.967955	1.494896	2.499536
H	7.504032	2.113232	-0.881268
H	5.323717	1.193446	-1.540756
H	6.738042	-2.844586	1.503834

H	4.672323	-1.526575	1.685019
H	6.324674	-2.900047	-2.753231
H	4.256093	-1.583871	-2.566142
H	2.424202	-2.993617	-1.547304
C	1.784020	-3.564484	0.421878
C	2.627171	3.755168	-1.026942
H	1.055816	-3.265554	1.176459
H	1.609524	-4.608946	0.159956
H	2.782612	-3.469414	0.845834
H	2.622727	4.828369	-0.831865
H	2.005745	3.552847	-1.900158
H	3.646777	3.440811	-1.246383
H	-7.898663	1.095260	0.784956
H	-6.574204	1.809443	1.683725
H	-6.865345	-2.080404	-1.653905
H	-7.427528	-0.461196	-1.339461
H	-6.899438	1.620059	-1.307430
H	-6.323564	2.944240	-0.330996
H	-5.912191	-0.071433	-3.137612
H	-4.875310	-1.396356	-2.646942
H	-4.846541	-1.552189	3.721831
H	-4.918065	-2.848517	2.527091
H	-3.619617	-2.819487	3.715672
H	-1.522687	1.261918	1.291042

(*P*,*R*,*R*,R_p)-**22a** (B3LYP/def2-TZVP/GD3):

SCF: -2118.148186

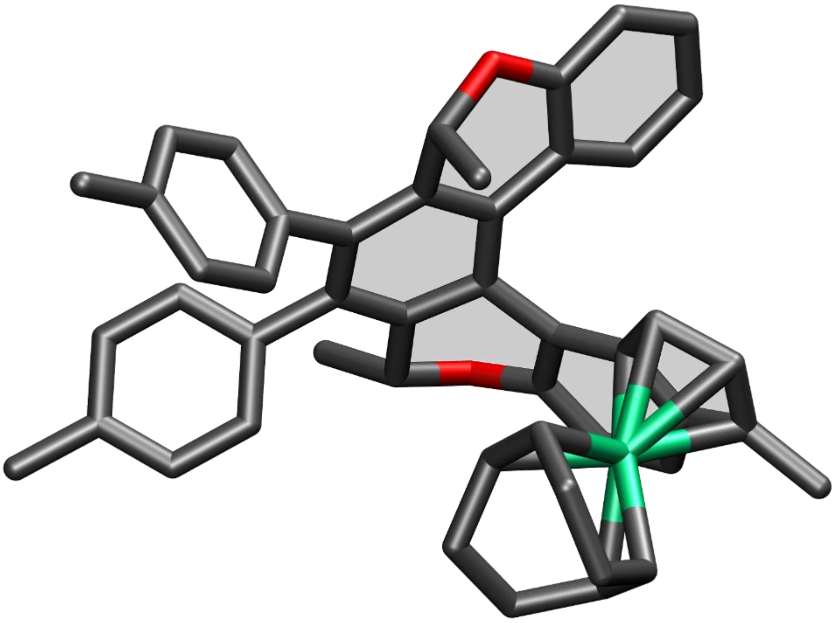

C	1.681573	0.454256	2.879131
C	1.530938	-0.396470	1.793243
O	0.595023	0.667335	3.679072
C	0.177008	-0.886943	1.490449
C	-0.605865	1.000669	2.950040
C	-0.868453	-0.000104	1.823146
C	2.874215	1.129628	3.173781
C	3.947270	1.045447	2.311812
C	2.629586	-0.488424	0.898491
C	3.825190	0.261798	1.153788
C	-0.121639	-2.047074	0.741858
C	-1.320304	-2.061272	0.005577
C	-2.076966	-0.046884	1.120474
C	-2.268221	-1.050448	0.148245
C	0.685825	-3.282708	0.745854
C	0.216082	-4.398296	0.027938
C	-1.455513	-3.098418	-1.086719
O	-0.936378	-4.374853	-0.689977
C	1.811794	-3.488328	1.559998
C	2.468730	-4.704497	1.614979
C	0.874140	-5.625743	0.076170
C	2.003931	-5.778969	0.859743
C	2.809670	-1.140081	-0.383107
C	4.129660	-0.877171	-0.837457
C	4.713313	0.091386	0.015305
H	2.907074	1.744048	4.063368
H	4.858982	1.594342	2.509602
H	2.169987	-2.678874	2.175852
H	3.336042	-4.816334	2.252150
H	0.467974	-6.444202	-0.503269
H	2.513600	-6.733681	0.890463
H	2.130371	-1.843218	-0.828061
H	4.591442	-1.300709	-1.715573
Rh	2.860119	1.004124	-0.949474
C	1.356720	0.988818	-2.471180
C	0.840872	1.656071	-1.344121
C	3.621530	2.638106	-2.132955
C	3.195091	3.119276	-0.881665
C	1.880228	1.663982	-3.722118
H	1.033789	-0.036583	-2.622761
C	0.678497	3.168451	-1.259589
H	0.190401	1.080001	-0.692530
H	4.689089	2.485669	-2.256118
C	2.841289	2.833385	-3.427598
H	3.968904	3.301746	-0.140675
C	1.893987	3.841699	-0.598035

C	6.098157	0.646932	-0.078562
H	-2.502361	-3.298447	-1.296014
C	-4.641609	0.025790	-2.595434
C	-3.530515	-0.048904	-1.766487
C	-5.721919	-0.842125	-2.434418
C	-5.651246	-1.787342	-1.410979
C	-4.540580	-1.864809	-0.580829
C	-3.462171	-0.996072	-0.744778
C	-6.913263	-0.777316	-3.353003
C	-3.790620	3.327334	1.075542
C	-2.829797	2.332390	0.947701
C	-5.073224	3.032986	1.535455
C	-5.359210	1.705667	1.861706
C	-4.402077	0.710893	1.731122
C	-3.116027	1.008977	1.278414
C	-6.129008	4.100565	1.645056
H	-7.094483	0.241571	-3.698845
H	-6.757160	-1.399718	-4.239007
H	-7.817916	-1.134453	-2.858785
H	-5.686017	5.093342	1.733562
H	-6.771385	4.104542	0.759589
H	-6.772198	3.936776	2.511427
H	-4.671828	0.775653	-3.377747
H	-2.708526	0.643290	-1.901161
H	-6.479627	-2.469267	-1.256350
H	-4.511809	-2.601193	0.213464
H	-3.538906	4.347691	0.809796
H	-1.843298	2.583766	0.580174
H	-6.347855	1.447677	2.223944
H	-4.648322	-0.310093	1.990908
H	6.122464	1.703842	0.194353
H	6.488047	0.552481	-1.092229
H	6.779219	0.120017	0.596717
H	-0.469952	2.008540	2.535858
C	-1.682789	1.028377	4.027580
C	-0.771681	-2.639116	-2.374691
H	-1.877484	0.016314	4.383798
H	-2.609927	1.461404	3.667730
H	-1.315601	1.626309	4.861674
H	-1.226219	-1.712481	-2.726613
H	0.290082	-2.459275	-2.200775
H	-0.877872	-3.403034	-3.145957
H	1.048235	2.001546	-4.354889
H	2.414143	0.906036	-4.299142
H	-0.220240	3.406246	-0.687514
H	0.508153	3.577194	-2.257022
H	2.292837	3.776010	-3.381891
H	3.543989	2.937564	-4.256451
H	1.960616	4.898330	-0.890323
H	1.753433	3.831981	0.485573

(*P*,*R*,*R*,S_p)-**22a** (B3LYP/def2-TZVP/GD3):

SCF: -2118.1334429

C	0.665032	-2.599750	-0.646096
C	0.655715	-1.264542	-0.253697
O	-0.326067	-3.036149	-1.478490
C	-0.586316	-0.505948	-0.511081
C	-1.663839	-2.716960	-1.047517
C	-1.775927	-1.255808	-0.614318
C	1.602448	-3.542477	-0.202860
C	2.532682	-3.192189	0.751524
C	2.541069	-1.875865	1.232965
C	1.661248	-0.878679	0.679640
C	-0.692335	0.900398	-0.458796
C	-1.901143	1.494032	-0.054745
C	-3.007275	-0.650532	-0.338726
C	-3.064640	0.719769	0.002059
C	0.330279	1.798905	-1.007757
C	0.312657	3.136704	-0.603615
C	-1.881421	2.978011	0.318148
O	-0.537309	3.507268	0.391197
C	1.180722	1.442333	-2.056112
C	2.010376	2.383955	-2.649230

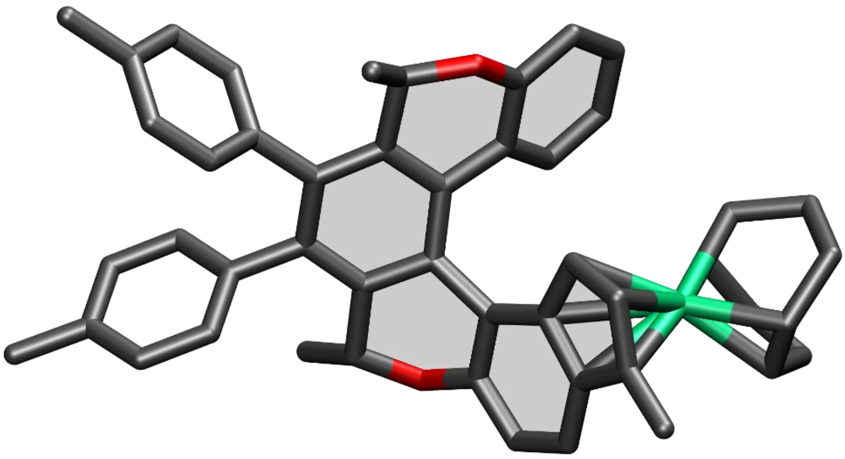

C	1.159730	4.080754	-1.165787
C	2.014779	3.698326	-2.192705
C	3.323850	-1.258306	2.289384
C	2.858666	0.083914	2.416225
C	1.954172	0.356833	1.369909
H	1.529000	-4.551929	-0.583919
H	3.220361	-3.926661	1.150677
H	1.168592	0.422235	-2.416497
H	2.664126	2.090277	-3.459174
H	1.121572	5.099832	-0.804439
H	2.671454	4.429476	-2.646644
C	4.174234	-2.002181	3.270088
H	3.182085	0.788907	3.166681
Rh	4.092054	-0.202859	0.525093
C	6.103877	-0.035385	1.162982
C	6.013289	-1.138238	0.284869
C	4.715491	1.400143	-0.811138
C	4.434105	0.242860	-1.549946
C	6.674442	1.322485	0.809039
H	6.146589	-0.259872	2.224548
C	6.543331	-1.120727	-1.142318
H	5.963084	-2.120425	0.743442
H	3.943371	2.158273	-0.775704
C	6.129273	1.881766	-0.518800
H	3.463428	0.195433	-2.027171
C	5.455479	-0.704128	-2.146081
H	-2.416650	3.561063	-0.441932
C	-6.325370	1.533202	1.761377
C	-5.092890	0.967438	1.475917
C	-6.905792	2.473501	0.906663
C	-6.207062	2.820301	-0.247955
C	-4.969247	2.256071	-0.534301
C	-4.388057	1.326310	0.325392
C	-8.234685	3.098848	1.237978
C	-5.622809	-3.189980	0.690389
C	-4.449812	-2.445346	0.646898
C	-6.654359	-2.960899	-0.218010
C	-6.470810	-1.957525	-1.172373
C	-5.303389	-1.211890	-1.214969
C	-4.267664	-1.448220	-0.309100
C	-7.939323	-3.743296	-0.159349
H	-8.939198	2.355790	1.616817
H	-8.124524	3.864253	2.011461
H	-8.681246	3.575114	0.364466
H	-7.817929	-4.670161	0.402407
H	-8.727418	-3.162300	0.328350
H	-8.296255	-3.996611	-1.159404
H	-6.850530	1.237064	2.662414
H	-4.666960	0.236288	2.150276
H	-6.637575	3.536967	-0.937890
H	-4.452078	2.532586	-1.445061
H	-5.736872	-3.959441	1.445319
H	-3.666351	-2.636433	1.370097
H	-7.255930	-1.757747	-1.892778
H	-5.186200	-0.438342	-1.962332
H	-1.907897	-3.379181	-0.206554
C	-2.520139	-3.087197	-2.253928
C	-2.450137	3.291733	1.697908
H	-2.357746	-2.370147	-3.059418
H	-3.577342	-3.116973	-2.014211
H	-2.210303	-4.073806	-2.598841
H	-3.526684	3.168241	1.736103
H	-1.991095	2.643213	2.444921
H	-2.207145	4.326752	1.938662
H	7.772066	1.284086	0.791741
H	6.404726	2.006438	1.616627
H	6.917992	-2.112753	-1.401558
H	7.403018	-0.450465	-1.205498
H	6.785881	1.605878	-1.346413
H	6.136902	2.972669	-0.480756
H	5.903716	-0.269285	-3.049563
H	4.916299	-1.596359	-2.471032
H	4.770136	-1.315694	3.871820
H	4.857194	-2.693271	2.772898
H	3.553254	-2.590415	3.952175

H	1.468619	1.301988	1.205788

Theoretical ECD spectra:

(M,R,R,R_p)-**25a** (PBE0/def2-SVP/GD3/PCM):

SCF: -13898.6056683

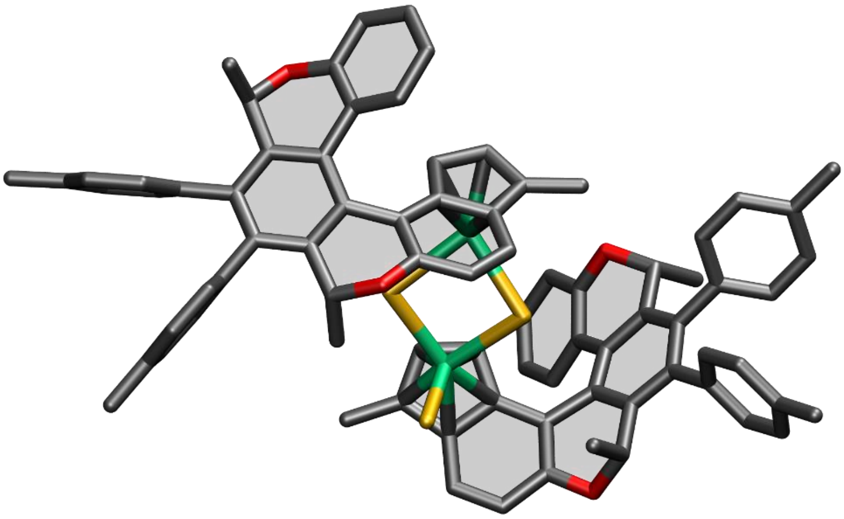

C	-2.481453	-1.299105	-2.825556
C	-2.823922	-1.777760	-1.569927
O	-3.385410	-0.634929	-3.561946
C	-4.207939	-1.601035	-1.124373
C	-4.258839	0.234382	-2.810622
C	-4.896495	-0.510291	-1.665696
C	-1.212727	-1.509255	-3.412707
C	-0.218685	-2.142421	-2.729251
C	-4.879292	-2.432145	-0.206334
C	-6.052258	-1.962513	0.389893
C	-6.124498	-0.103569	-1.144304
C	-6.660960	-0.784387	-0.042364
C	-4.478778	-3.802345	0.131348
C	-4.890602	-4.312996	1.367410
C	-6.683524	-2.821328	1.456227
O	-5.685804	-3.565010	2.170296
C	-3.760558	-4.646596	-0.716794
C	-3.374906	-5.910890	-0.306842
C	-4.482063	-5.565464	1.800834
C	-3.709690	-6.357727	0.966697
H	-1.057516	-1.135179	-4.417075
H	-3.495032	-4.299920	-1.708236
H	-2.811516	-6.547414	-0.978544
H	-4.797252	-5.909944	2.778596
H	-3.396258	-7.340443	1.299694
H	-7.126068	-2.182984	2.219719
C	-8.995752	0.768932	2.485748
C	-7.846942	0.286310	1.878119
C	-10.232054	0.694625	1.849582
C	-10.279552	0.113469	0.584488
C	-9.133357	-0.367399	-0.027518
C	-7.898721	-0.287057	0.610568
C	-11.467845	1.247613	2.491687
C	-7.553748	3.372132	-1.531185
C	-6.871900	2.282226	-1.015552
C	-8.220760	3.294169	-2.751813
C	-8.184640	2.082867	-3.436927
C	-7.502154	0.989812	-2.924745
C	-6.834933	1.074091	-1.706391
C	-8.933157	4.484354	-3.318508
H	-11.632905	2.284819	2.183527
H	-11.389428	1.238483	3.580232
H	-12.353996	0.678399	2.204612
H	-8.241891	5.116465	-3.884670
H	-9.365010	5.102002	-2.528659
H	-9.732881	4.183708	-3.997566
H	-8.929265	1.214023	3.473381
H	-6.892944	0.362527	2.389664
H	-11.231271	0.036279	0.068375
H	-9.194656	-0.809251	-1.015851
H	-7.571003	4.302369	-0.972298
H	-6.365826	2.364486	-0.060038
H	-8.704902	1.989856	-4.384914
H	-7.500559	0.051941	-3.470819
H	-5.031245	0.494230	-3.533340
C	-3.511476	1.483190	-2.395172
C	-7.749619	-3.748077	0.897033
H	-2.705702	1.253912	-1.697932
H	-3.085190	1.973069	-3.271785
H	-4.199849	2.175083	-1.907547
H	-8.158990	-4.372693	1.693073
H	-7.339405	-4.390456	0.114924
H	-8.557035	-3.153738	0.466413
Rh	0.664886	2.302103	0.512201
Br	-1.398431	0.892052	0.803165
Br	-0.121188	3.148884	-1.722419
C	0.719331	2.744919	2.574017
H	0.015871	2.277797	3.247499
C	0.482176	3.952126	1.831180
C	1.687918	4.213174	1.078695
C	2.588085	3.107671	1.261346
C	1.954005	2.186475	2.182448
C	-0.687110	4.856342	1.955349
Rh	-0.146554	-1.345389	0.447631
Br	1.711803	0.126488	-0.548216

Br	0.825033	-1.195946	2.726982
C	-0.479827	-2.559685	-1.406847
C	0.386027	-3.193529	-0.428637
C	-0.407424	-3.408282	0.735849
H	-0.059053	-3.872060	1.646596
C	-1.675501	-2.788447	0.560619
C	-1.771523	-2.353694	-0.818103
C	1.770746	-3.669668	-0.664975
H	-0.480812	5.640337	2.690648
H	-1.567407	4.303786	2.283955
H	-0.912793	5.333326	1.001091
H	1.754525	-4.608037	-1.227545
H	2.280912	-3.845242	0.281913
H	2.342698	-2.939630	-1.239781
H	2.338834	1.238177	2.517001
H	-2.463328	-2.760939	1.294721
C	3.987972	4.021409	-0.428514
C	3.795105	3.032122	0.515558
O	4.996641	3.898326	-1.315413
C	4.724960	1.904165	0.531894
C	5.112689	2.556148	-1.835841
C	5.282701	1.571237	-0.706032
C	3.161402	5.164638	-0.530187
C	2.020439	5.272628	0.205839
C	5.019723	1.105819	1.651245
C	5.579227	-0.155652	1.441016
C	5.907970	0.340950	-0.898891
C	5.976509	-0.566138	0.168523
C	4.801355	1.513800	3.041146
C	4.664086	0.504869	4.002430
C	5.809887	-1.025036	2.649616
O	4.812649	-0.793106	3.651671
C	4.735342	2.840093	3.471784
C	4.444249	3.151228	4.788209
C	4.340312	0.806592	5.317435
C	4.217419	2.130577	5.705326
H	3.439206	5.922857	-1.252252
H	1.364030	6.126732	0.103467
H	4.907310	3.636679	2.757836
H	4.391833	4.187574	5.099590
H	4.207766	-0.005696	6.022102
H	3.972194	2.366832	6.734400
H	5.666791	-2.070180	2.380136
C	6.088339	-4.312313	-0.239137
C	5.628432	-3.025975	-0.006870
C	7.429917	-4.555417	-0.523753
C	8.295963	-3.465065	-0.560505
C	7.838948	-2.176498	-0.332822
C	6.496592	-1.938348	-0.054440
C	7.921839	-5.941798	-0.809011
C	6.201203	-1.364280	-4.253077
C	5.726992	-0.984783	-3.008680
C	7.375009	-0.822265	-4.773396
C	8.059395	0.111114	-4.000419
C	7.587127	0.494806	-2.753736
C	6.414039	-0.048086	-2.240014
C	7.868606	-1.216667	-6.132134
H	7.885878	-6.152180	-1.882572
H	7.308665	-6.692988	-0.308029
H	8.956879	-6.070738	-0.487043
H	7.360145	-0.641008	-6.911863
H	7.677541	-2.272813	-6.332726
H	8.939578	-1.034516	-6.235274
H	5.390922	-5.143249	-0.198915
H	4.579106	-2.854712	0.208165
H	9.348069	-3.627245	-0.772325
H	8.531147	-1.342937	-0.375478
H	5.650498	-2.098800	-4.832248
H	4.813725	-1.422055	-2.619990
H	8.982039	0.541692	-4.376325
H	8.145936	1.214208	-2.163975
H	6.031886	2.589789	-2.419498
C	3.940139	2.247634	-2.748295
C	7.204140	-0.840714	3.224894
H	2.998062	2.228582	-2.196623
H	3.875199	3.000923	-3.535581
H	4.084746	1.269532	-3.208499
H	7.329811	-1.462597	4.113126
H	7.381699	0.203276	3.492207
H	7.945645	-1.136510	2.480571
H	0.752953	-2.307750	-3.176532

TD-DFT-calculated CD transitions:

[Wavelength (nm), Rot. Strength (length)]: 611.482, 40.0877; 606.665, -4.7846; 579.690, 2.3711; 541.297, -38.7971; 528.784, 71.6928; 494.453, -16.648; 477.799, -152.5168; 468.678, -66.5174; 461.748, -27.6956; 456.075, 26.9877; 447.871, -45.9308; 439.348, 6.836; 436.717, 34.7666; 431.204, -3.778; 429.724, -53.0333; 425.419, -8.4179; 421.801, -2.1853; 412.606, 26.3572; 412.496, -45.6772; 407.950, -29.9369; 406.932, -6.5383; 400.931, 17.0112; 391.154, -9.5383; 387.438, -27.6823; 386.689, 0.1181; 383.424, -38.9426; 381.454, -3.9021; 378.196, 6.27; 376.989, -131.3221; 375.755, -18.7638; 372.168, -15.2487; 359.593, -14.6523; 358.366, -8.9826; 355.011, -0.6738; 353.493, 0.7277; 351.688, -10.0245; 348.603, -22.7238; 346.276, -13.4395; 344.094, -3.9521; 342.876, -1.0746; 341.497, -45.2625; 341.413, 33.0427; 339.255, -38.1949; 337.932, -23.0597; 335.654, -58.7433; 331.295, -23.653; 330.862, 43.6096; 329.508, 41.8211; 328.853, -11.417; 325.922, -13.285; 325.520, 76.41; 324.362, -5.0572; 323.684, 13.6819; 323.018, -44.6278; 320.986, 78.8464; 318.823, 51.238; 317.233, -26.2325; 316.593, -26.0281; 315.521, -290.1229; 313.891, -50.9772; 313.590, -5.0344; 311.760, -23.5308; 311.135, -28.7678; 310.410, -59.6344; 309.588, -14.6313; 308.925, 26.5391; 306.823, 14.8483; 306.345, 11.247; 306.269, 7.8373; 305.176, -19.8314; 303.815, 59.9298; 303.681, -1.5083; 301.855, -13.7652; 299.529, -31.6199; 299.471, -10.8615; 298.203, 9.6394; 297.981, 11.358; 296.904, -0.098; 296.095, -11.8991; 294.331, 8.8399; 293.905, 1.0569; 292.443, 32.6293; 292.050, 9.0341; 291.645, -37.3573; 291.350, -23.576; 291.240, -0.048; 290.075, -67.0244; 289.527, 71.5309; 289.162, 110.2506; 288.771, 37.2414; 288.194, -0.4708; 287.853, -230.8776; 287.773, 22.8565; 286.721, 3.7137; 286.000, -25.5867; 285.329, 112.563; 284.772, -47.5826; 284.497, -10.1686; 283.807, 2.281; 282.250, 12.8106; 280.901, -16.6074; 280.348, -9.6387; 279.111, -39.3209; 277.121, -23.816; 276.843, -0.0493; 275.257, 25.3168; 274.083, 85.9948; 273.726, 99.8933; 273.454, 2.467; 272.378, -90.8682; 271.746, -18.1967; 270.879, 117.4161; 270.696, -20.5614; 270.100, -86.8178; 268.381, -1.9061; 267.992, -14.3202; 267.853, -11.2624; 267.005, -19.6711; 265.741, -3.3043; 265.326, -14.8812; 264.556, -22.9794; 263.325, 29.5361; 262.450, 31.8573; 262.056, -30.22; 261.873, 24.221; 261.366, 7.3577; 260.942, 17.2584; 260.580, -24.9753; 259.832, -14.0869; 259.636, -7.3801; 259.435, -2.6655; 258.872, 8.8436; 258.435, 26.1368; 257.913, -25.9493; 257.822, -12.8121; 257.490, 3.0652; 257.346, -5.8423; 257.249, 21.1778; 256.770, -1.027; 256.250, -2.0122; 256.017, -5.3687; 255.864, 9.7133; 255.616, -16.5008; 254.446, -4.3388; 254.248, 65.7972; 253.946, -17.6317; 253.163, -6.7324; 252.359, 31.231; 252.077, 0.0175; 251.862, -4.1662; 251.290, -5.8805; 251.000, -2.1291; 250.549, -0.1716; 250.321, 29.1744; 250.139, 1.456; 249.867, 2.8444; 249.646, 4.3827; 249.490, 6.5295; 248.674, 8.0577; 248.584, -18.7667; 248.142, -14.5225; 247.953, 19.4233; 247.686, 16.1787; 247.394, -35.8494; 245.829, -1.9433; 245.766, -11.7675; 245.751, 11.7653; 245.158, 3.2484; 244.511, -3.2547; 244.385, 41.3447; 243.914, -18.4635; 243.402, -2.3679; 242.716, 18.6254; 242.507, -43.4984; 241.755, 11.8981; 241.388, -2.4568; 241.054, -6.9502; 240.956, -4.5519; 240.783, 10.8203; 240.149, -33.9417; 239.856, 1.1636; 239.661, 38.1327; 239.439, -11.5211; 238.807, 16.5786; 238.614, -11.6956; 238.289, -7.8947; 237.978, 45.0606; 237.809, 5.9649; 237.513, -6.8801; 237.381, 48.0473; 236.814, -6.062; 236.733, -3.1174; 236.471, -7.1783; 236.182, 1.2285; 236.115, -18.7204; 235.958, -10.2616; 235.702, 13.5292; 234.716, -13.1167; 234.312, -17.5499; 234.206, 9.8177.

(*M*,*R*,*R*,*R*$_p$)-**22a** (PBE0/def2-TZVP/GD3/PCM):

SCF: -2115.7179352

C	-1.319495	-0.837880	-2.659057
C	-1.190865	0.121795	-1.664020
O	-0.195269	-1.416742	-3.168631
C	0.166725	0.489487	-1.249600
C	0.746991	-1.752387	-2.140824
C	1.135870	-0.513402	-1.374235
C	-2.558489	-1.271330	-3.149633
C	-3.725761	-0.825774	-2.577819
C	-2.386793	0.552337	-1.036543
C	-3.652430	0.066352	-1.496315
C	0.555225	1.733515	-0.716119
C	1.778614	1.825802	-0.044212
C	2.389671	-0.387315	-0.779272
C	2.682920	0.764285	-0.034764
C	-0.205696	2.978948	-0.865465
C	0.014238	3.994556	0.071261
C	2.124712	3.145299	0.597357
O	0.943225	3.822654	1.045494
C	-1.100577	3.235296	-1.904835
C	-1.813418	4.420264	-1.959220
C	-0.716613	5.172901	0.043959
C	-1.641571	5.378949	-0.967027
C	-2.654070	1.375973	0.119320
C	-4.063238	1.499101	0.265488
C	-4.687015	0.601581	-0.633586
H	-2.564634	-1.995895	-3.955138
H	-4.686090	-1.179551	-2.935211
H	-1.245305	2.485578	-2.672724
H	-2.508469	4.594159	-2.772003
H	-0.531527	5.921444	0.805351
H	-2.208918	6.302225	-0.995635
H	-1.919638	1.930626	0.680130
H	-4.572708	2.130050	0.979566
Rh	-3.402254	-0.567706	0.767882
C	-2.442612	-0.652608	2.639326
C	-1.817571	-1.569796	1.770462
C	-4.824778	-1.788357	1.771819
C	-4.208933	-2.525014	0.744036
C	-3.420503	-1.021848	3.726345
H	-1.927038	0.291094	2.806013
C	-2.007782	-3.071964	1.860513
H	-0.883563	-1.242557	1.318240
H	-5.811647	-1.385092	1.557690

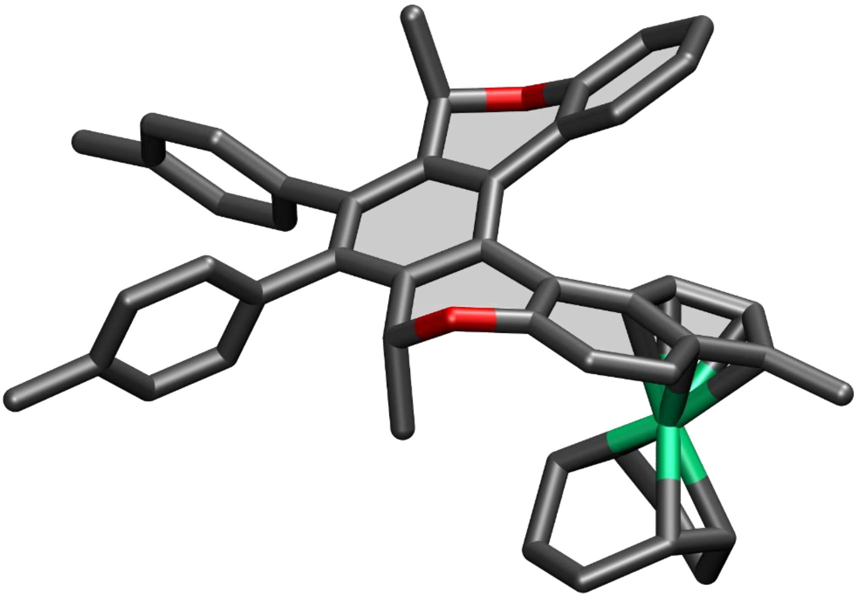

C	-4.506412	-1.985548	3.241367
H	-4.770047	-2.632484	-0.182689
C	-3.124631	-3.556300	0.933632
C	-6.149978	0.407258	-0.832881
H	2.685278	2.967673	1.514299
C	5.199342	0.883903	2.780552
C	3.999273	0.804566	2.090830
C	6.408893	1.028235	2.106114
C	6.374168	1.096840	0.715289
C	5.176762	1.015921	0.022699
C	3.969590	0.867615	0.700306
C	7.709127	1.079371	2.849316
C	4.588928	-3.318586	0.139936
C	3.646430	-2.305995	0.220586
C	5.301632	-3.547934	-1.034708
C	5.039961	-2.723570	-2.125863
C	4.096827	-1.710103	-2.049065
C	3.385528	-1.485960	-0.873817
C	6.300359	-4.661336	-1.126992
H	8.148369	0.080132	2.930787
H	7.573894	1.460833	3.862912
H	8.435686	1.712621	2.336604
H	5.813360	-5.596518	-1.420854
H	6.789312	-4.837223	-0.166944
H	7.069165	-4.445622	-1.871030
H	5.195691	0.830610	3.864661
H	3.069660	0.684816	2.637885
H	7.301515	1.216022	0.163858
H	5.176121	1.066007	-1.060552
H	4.778157	-3.940979	1.008889
H	3.110577	-2.139958	1.148796
H	5.588468	-2.872193	-3.050639
H	3.919464	-1.072573	-2.909137
H	-6.391947	-0.642366	-1.016683
H	-6.709427	0.734312	0.044611
H	-6.507155	0.979630	-1.695131
H	1.612935	-2.120693	-2.689867
C	0.208967	-2.857962	-1.248393
C	2.941246	4.037105	-0.323066
H	-0.662481	-2.514142	-0.688583
H	-0.074946	-3.723435	-1.850304
H	0.980142	-3.160800	-0.538063
H	3.145994	4.992651	0.163525
H	2.411901	4.220698	-1.260458
H	3.889988	3.549494	-0.553053
H	-2.892542	-1.434927	4.597689
H	-3.893270	-0.096695	4.067517
H	-1.072297	-3.571375	1.594659
H	-2.212111	-3.354821	2.896334
H	-4.206928	-3.022705	3.411573
H	-5.414914	-1.836409	3.830123
H	-3.546656	-4.507649	1.287822
H	-2.700230	-3.762261	-0.053523

TD-DFT-calculated CD transitions:

[Wavelength (nm), Rot. Strength (length)]: 400.893, -18.4497; 375.743, -210.2099; 338.384, -191.7746; 328.208, 90.3364; 325.605, 52.6181; 324.073, -82.0788; 312.736, -121.4288; 311.635, 88.2639; 300.618, 26.0851; 290.715, 6.2626; 290.075, -44.0624; 288.295, -17.0738; 282.494, 58.35; 279.388, 27.2265; 274.355, -21.5901; 273.973, -10.7367; 273.026, -38.7963; 271.033, 68.6561; 268.829, -29.8954; 268.654, 4.9145; 266.747, 22.9815; 264.606, -27.2675; 260.427, 5.5295; 258.580, 5.4219; 255.975, -21.154; 255.727, -9.853; 254.964, -33.2171; 253.173, 7.11; 252.169, -11.343; 250.023, -19.9489; 248.375, 53.5347; 247.532, 18.7145; 247.034, -20.9044; 246.102, -78.2161; 244.921, -5.9842; 244.159, 36.8104; 243.373, 44.0537; 242.730, 10.7343; 241.374, -27.725; 240.563, 9.5679; 240.037, 63.037; 239.305, -3.4666; 237.622, -48.0438; 235.818, -28.5462; 235.317, -66.5733; 233.663, 0.3948; 232.315, -0.4221; 231.845, -63.494; 231.171, 0.3823; 230.685, 29.907; 229.256, -8.0832; 229.209, 27.7053; 228.092, -0.0234; 227.485, -30.7645; 227.227, 1.9755; 226.798, -3.263; 226.322, -7.3036; 226.001, -33.7554; 225.008, 11.9433; 224.401, -23.2481; 223.407, -0.7147; 222.804, -15.67; 222.150, -31.0122; 221.975, -41.149; 221.076, 40.5437; 220.208, -4.9387; 218.505, 18.8666; 218.212, -30.7341; 217.623, 17.7404; 217.081, 122.9039; 216.509, 43.1852; 216.166, -61.2541; 216.034, 18.4723; 215.940, 19.6517; 215.310, 86.93; 214.918, 16.5539; 214.246, -3.0997; 214.142, -20.2149; 214.131, -12.1799; 213.578, 34.2923; 213.232, 7.8866; 212.899, 3.2735; 212.735, 16.4783; 212.076, -28.3936; 211.920, 1.7729; 211.609, -13.8565; 211.112, -6.7555; 210.739, -4.337; 210.488, -8.2323; 210.064, 48.0766; 209.659, 17.3671; 208.776, 4.6677; 208.331, -57.3101; 208.142, 64.7145; 207.217, -30.4781; 206.809, -14.2693; 206.616, 9.7652; 205.844, -15.3324; 205.370, 7.0102; 205.152, 39.6709.

(*M*,*R*,*R*)-**14a** (PBE0/def2-TZVP/GD3/PCM):

SCF: -1693.8770063

C	-2.432311	-2.464227	-0.786188
C	-2.377449	-1.195344	-0.183648
O	-1.298488	-2.983841	-1.330551
C	-1.101557	-0.469912	-0.288688

C	-0.137670	-2.740618	-0.522397
C	0.053483	-1.257793	-0.336185
C	-3.595737	-3.217609	-0.828522
C	-4.733277	-2.767302	-0.175436
C	-3.496782	-0.804136	0.550351
C	-4.661445	-1.585624	0.550178
C	-0.956610	0.928853	-0.341859
C	0.303373	1.485879	-0.100244
C	1.321640	-0.696442	-0.193143
C	1.439350	0.685141	0.014942
C	-2.024295	1.865150	-0.707613
C	-1.914072	3.185338	-0.258470
C	0.402858	2.989732	-0.057695
O	-0.813533	3.569231	0.434782
C	-3.114349	1.539683	-1.516551
C	-4.110155	2.464517	-1.776807
C	-2.920653	4.111746	-0.488979
C	-4.027448	3.743752	-1.236578
C	-3.707885	0.342765	1.494363
C	-5.097968	0.115756	1.998519
C	-5.648415	-0.974434	1.441406
H	-3.576886	-4.172619	-1.340201
H	-5.641235	-3.360180	-0.187275
H	-3.188069	0.540555	-1.926989
H	-4.954015	2.187759	-2.397376
H	-2.808579	5.115716	-0.096941
H	-4.813172	4.466676	-1.423687
H	-5.583449	0.770858	2.711130
C	-7.009123	-1.530799	1.662821
H	1.147238	3.283601	0.681213
C	4.326368	2.329263	1.813157
C	3.078440	1.785449	1.549225
C	5.306617	2.393713	0.826695
C	4.991254	1.900100	-0.437306
C	3.746371	1.353654	-0.703892
C	2.770100	1.286575	0.286717
C	6.666865	2.949720	1.120065
C	4.366638	-2.612501	0.962946
C	3.222313	-1.830722	0.975867
C	4.862299	-3.145361	-0.224464
C	4.173245	-2.863954	-1.401506
C	3.027937	-2.083002	-1.392285
C	2.534802	-1.554372	-0.202412
C	6.083748	-4.013427	-0.232180
H	7.363879	2.148547	1.385190
H	6.639230	3.650098	1.956553
H	7.081187	3.465780	0.251754
H	5.812768	-5.065952	-0.102355
H	6.764268	-3.749618	0.579438
H	6.624720	-3.930541	-1.176616
H	4.542769	2.709468	2.806473
H	2.333089	1.739349	2.336638
H	5.734065	1.943947	-1.227590
H	3.526922	0.969860	-1.694014
H	4.888227	-2.808914	1.894333
H	2.861256	-1.418164	1.911556
H	4.542954	-3.256748	-2.343419
H	2.515708	-1.866621	-2.324203
H	-6.956439	-2.553469	2.049609
H	-7.574712	-0.924782	2.371559
H	-7.571130	-1.577538	0.724713
H	0.681758	-3.134911	-1.122167
C	-0.204218	-3.501953	0.790347
C	0.775480	3.582544	-1.406261
H	-0.999261	-3.115692	1.431279
H	-0.381922	-4.562603	0.602622
H	0.745850	-3.392972	1.316469
H	0.806442	4.671884	-1.342581
H	0.056244	3.290592	-2.174398
H	1.761245	3.219645	-1.701736
H	-2.959011	0.335866	2.296574
H	-3.616335	1.319203	1.010849

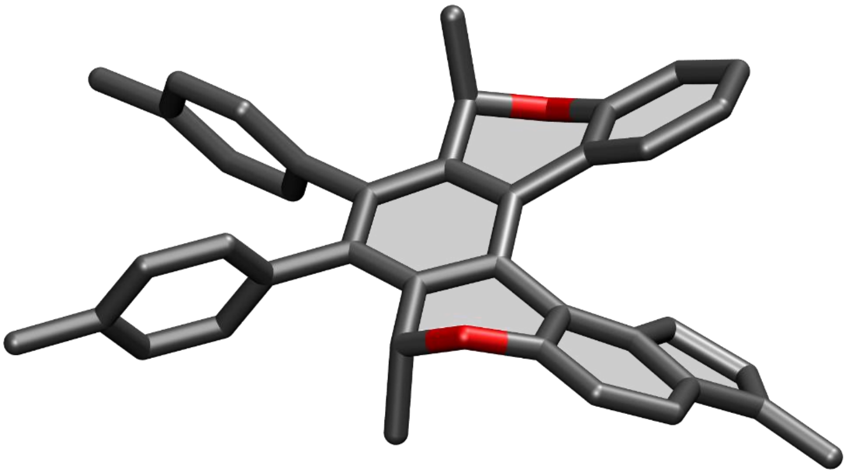

TD-DFT-calculated CD transitions:

[Wavelength (nm), Rot. Strength (length)]: 352.718, 13.6465; 327.290, -68.5231; 304.315, -39.6388; 291.466, -105.0844; 280.875, -51.4218; 272.990, -43.829; 268.451, -219.5907; 266.220, 166.4309; 258.699, 26.9723; 256.250, 17.1877; 255.038, 29.8803; 252.421, 5.4028; 251.259, 88.66; 248.345, -20.1785; 247.814, -8.062; 246.137, 2.6165; 243.986, 43.1318; 242.640, 10.6424; 241.303, -21.3911; 240.783, 4.0588; 239.190, -20.6603; 237.978, -5.8994; 236.394, 38.5372; 236.016, 19.7532; 233.149, -11.809; 232.528, 17.9343; 231.542, 18.7108; 231.072, -0.6061; 229.040, 59.411; 228.176, 9.8139; 226.836, 14.8046; 223.790, 79.2788; 222.904, -

0.5145; 220.985, 21.3461; 218.455, -9.2192; 217.085, -8.1851; 216.842, 16.0304; 216.226, -3.8436; 215.740, -20.6513; 214.683, -26.5106; 214.305, -3.5139; 213.861, -33.3276; 213.269, -29.9534; 212.833, 8.0232; 211.917, 1.4656; 211.008, 4.8857; 210.057, -2.3984; 210.028, 31.0403; 209.372, 7.6847; 207.682, -0.6422; 207.050, 75.9746; 205.936, -7.962; 205.670, 47.404; 204.334, -25.2927; 203.369, 16.1071; 202.648, 38.1726; 201.751, -2.4015; 201.119, -71.2603; 200.346, -78.712; 199.376, 2.9831; 198.829, -2.682; 198.263, -39.3763; 198.086, 0.0355; 197.552, 42.6918; 196.563, -84.6599; 195.799, 0.756; 195.512, -34.1717; 194.925, 14.2674; 194.552, -23.4216; 193.773, 20.1113; 193.613, 3.7223; 193.329, 43.0355; 192.824, 7.5358; 192.626, -8.7853; 192.101, -28.3135; 191.434, 47.7364; 190.832, -17.0651; 190.448, -4.0309; 190.095, -1.1982; 188.793, -9.7775; 188.239, -52.2926; 187.318, -290.9843; 186.624, 138.0462; 186.419, 343.137; 185.738, 39.3279; 185.203, -142.1061; 185.042, -30.3522; 184.294, -10.3861; 184.048, -23.5222; 183.756, 77.5379; 183.208, -71.902; 182.964, 47.3202; 182.377, -48.6965; 182.136, 9.8214; 181.867, 35.9157; 181.510, -87.7651; 181.396, -19.1347; 181.131, 35.8948; 180.671, 28.1874; 180.485, -44.4303.

Single Crystal X-Ray analysis data for compound (M,R,R,S_p,S_p)-7

Report created with ReportPlus

R_1=4.66%

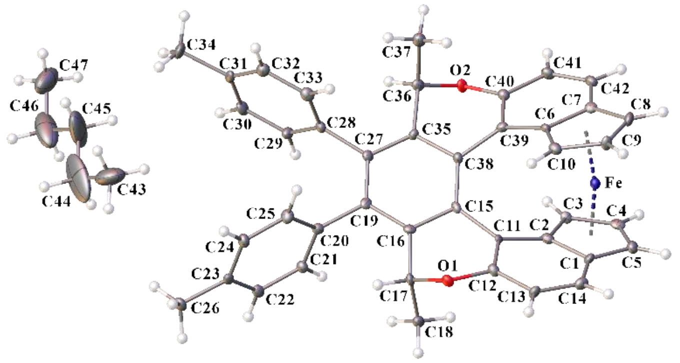

**Compound**	**(M,R,R,S_p,S_p)-7**
**CCDC**	**2373549**
Formula	$C_{47}H_{44}FeO_2$
$D_{calc.}$/ g cm^{-3}	1.330
μ/mm^{-1}	0.474
Formula Weight	696.67
Color	clear light green
Shape	plate-shaped
Size/mm^3	0.15×0.10×0.08
T/K	110.00
Crystal System	monoclinic
Flack Parameter	0.011(8)
Hooft Parameter	0.011(8)
Space Group	$P2_1$
a/Å	11.6884(10)
b/Å	8.2163(7)
c/Å	18.1173(15)
α/°	90
β/°	90.239(4)
γ/°	90
V/Å^3	1739.9(3)
Z	2
Z'	1
Wavelength/Å	0.71073
Radiation type	Mo K_α
Θ_{min}/°	1.742
Θ_{max}/°	24.999
Measured Refl's.	48085
Indep't Refl's	6114
Refl's I≥2 σ(I)	5530
R_{int}	0.0772
Parameters	449
Restraints	1
Largest Peak	0.523
Deepest Hole	-0.626
GooF	1.054
wR_2 (all data)	0.1007
wR_2	0.0955
R_1 (all data)	0.0569
R_1	0.0466

Experimental. Single clear light green plate-shaped crystals of (M,R,R,S_p,S_p)-**7** recrystallised by cooling its saturated pentane solution to -20 °C. A suitable crystal with dimensions 0.15 × 0.10 × 0.08 mm^3 was selected and mounted on a mylar loop oil on a Bruker APEX-II CCD diffractometer. The crystal was kept at a steady T = 110.00 K during data collection. The structure was solved with the **ShelXT** 2018/2[26] solution program using dual methods and by using **Olex2** 1.5[27] as the graphical interface. The model was refined with **ShelXL** 2018/3[26] using full matrix least squares minimisation on $\boldsymbol{F^2}$.

Crystal Data. $C_{47}H_{44}FeO_2$, M_r = 696.67, monoclinic, $P2_1$ (No. 4), a = 11.6884(10) Å, b = 8.2163(7) Å, c = 18.1173(15) Å, β = 90.239(4)°, α = γ = 90°, V = 1739.9(3) Å^3, T = 110.00 K, Z = 2, Z' = 1, μ(Mo K_α) = 0.474, 48085 reflections measured, 6114 unique (R_{int} = 0.0772) which were used in all calculations. The final wR_2 was 0.1007 (all data) and R_1 was 0.0466 (I≥2 σ(I)).

A clear light green plate-shaped-shaped crystal with dimensions 0.15 × 0.10 × 0.08 mm[3] was mounted on a mylar loop oil. Data were collected using a Bruker APEX-II CCD diffractometer operating at T = 110.00 K.

Data were measured using ϕ and ω scans with Mo K_α radiation. The diffraction pattern was indexed and the total number of runs and images was based on the strategy calculation from the program APEX3.[28] The maximum resolution that was achieved was Θ = 24.999° (0.84 Å). The unit cell was refined using SAINT V8.40B[29] on 9802 reflections, 20% of the observed reflections.

Data reduction, scaling and absorption corrections were performed using SAINT V8.40B.[29] The final completeness is 99.90 % out to 24.999° in Θ. SADABS-2016/2[30] was used for absorption correction. wR_2(int) was 0.0813 before and 0.0706 after correction. The Ratio of minimum to maximum transmission is 0.8860. The absorption coefficient μ of this material is 0.474 mm^{-1} at this wavelength (λ = 0.71073Å) and the minimum and maximum transmissions are 0.295 and 0.333.

The structure was solved and the space group $P2_1$ (# 4) determined by the ShelXT 2018/2[26] structure solution program using using dual methods and refined by full matrix least squares minimisation on $\boldsymbol{F^2}$ using version 2018/3 of **ShelXL** 2018/3.[26] All non-hydrogen atoms were refined anisotropically. Hydrogen atom positions were calculated geometrically and refined using the riding model. SADABS-2016/2[30] was used for absorption correction. wR_2(int) was 0.0813 before and 0.0706 after correction. The Ratio of minimum to maximum transmission is 0.8860. Refined as a 2-component twin. TWIN/BASF refinement type was used to determine absolute configuration from anomalous scattering using the Flack method.[31] The structure displays a merohedral twinning and the twin law was found by using TwinRotMat implemented in Platon.[32] The use of twin law (-1 0 0 0 -1 0 0 0 1) with a population of 0.652(2)/0.348(2) reduced the R1 (for I > 2sigma(I)) to 4.66%. The Flack parameter was refined to 0.011(8). Determination of absolute structure using Bayesian statistics on Bijvoet differences using the Olex2 results in 0.011(8).

Note: The Flack parameter is used to determine chirality of the crystal studied, the value should be near 0, a value of 1 means that the stereochemistry is wrong and the model should be inverted. A value of 0.5 means that the crystal consists of a racemic mixture of the two enantiomers.

Table S3: Fractional Atomic Coordinates (×10[4]) and Equivalent Isotropic Displacement Parameters (Å^2×10^3) for (M,R,R,S_p,S_p)-**7**. U_{eq} is defined as 1/3 of the trace of the orthogonalised U_{ij}.

Atom	x	y	z	U_{eq}
Fe	7444.8(9)	3941.7(13)	725.0(5)	16.3(2)
O1	6286(4)	696(6)	3014(3)	19.8(11)
O2	8391(4)	8369(5)	2617(3)	19.2(12)
C1	6074(7)	2370(10)	855(4)	18.6(18)
C2	6185(6)	3529(9)	1450(4)	18.7(18)
C3	6089(7)	5125(9)	1139(4)	16.1(17)
C4	5935(7)	4940(10)	369(5)	20(2)
C5	5973(8)	3284(10)	183(5)	23(2)
C6	8633(7)	4767(9)	1443(4)	14.4(18)
C7	8822(7)	5558(9)	745(5)	17.3(17)
C8	8986(7)	4295(10)	205(4)	24(2)
C9	8975(7)	2785(9)	575(4)	19.2(19)
C10	8763(7)	3057(10)	1338(5)	21.0(19)
C11	6453(6)	2946(8)	2197(4)	13.2(15)
C12	6272(6)	1324(8)	2310(4)	18.6(16)
C13	6128(6)	157(9)	1725(4)	19.8(17)
C14	6105(6)	659(9)	1012(4)	21.4(18)
C15	6940(5)	3914(11)	2812(3)	14.8(12)
C16	6624(6)	3363(8)	3524(4)	16.8(16)
C17	5879(6)	1872(8)	3550(4)	17.5(16)
C18	4619(6)	2207(9)	3427(4)	23.2(17)
C19	7043(5)	4126(10)	4160(4)	16.9(15)

Atom	x	y	z	U_{eq}
C20	6651(6)	3532(7)	4897(4)	16.8(17)
C21	5539(6)	3806(11)	5132(3)	19.3(15)
C22	5162(6)	3233(8)	5805(4)	21.1(16)
C23	5865(6)	2349(9)	6269(4)	20.5(17)
C24	6987(6)	2067(9)	6043(4)	23.1(17)
C25	7364(7)	2647(8)	5376(4)	23.2(17)
C26	5461(7)	1791(9)	7009(4)	26.4(19)
C27	7774(6)	5467(8)	4098(3)	16.7(16)
C28	8311(6)	6152(9)	4790(4)	18.0(16)
C29	7758(6)	7188(9)	5266(4)	23.0(17)
C30	8274(7)	7663(9)	5931(4)	27.5(19)
C31	9352(7)	7102(9)	6127(4)	23.6(18)
C32	9908(7)	6085(8)	5650(4)	24.9(18)
C33	9404(7)	5615(9)	4998(4)	24.3(18)
C34	9911(8)	7533(12)	6870(4)	36(2)
C35	8082(6)	6007(8)	3400(4)	14.2(15)
C36	8780(7)	7546(8)	3294(4)	19.9(17)
C37	10075(7)	7316(10)	3280(4)	28.0(19)
C38	7748(6)	5175(8)	2754(4)	15.5(15)
C39	8305(6)	5739(9)	2075(4)	16.6(15)
C40	8506(6)	7388(8)	2015(4)	17.2(16)
C41	8722(6)	8169(9)	1331(4)	21.6(17)
C42	8826(6)	7302(9)	703(4)	22.1(17)
C43	7733(12)	4755(13)	7986(6)	107(6)
C44	7171(8)	6125(14)	8430(6)	196(14)
C45	7833(7)	7736(13)	8398(4)	172(12)
C46	7278(9)	9117(15)	8841(5)	151(8)
C47	7947(12)	10721(14)	8803(7)	117(6)

Table S4: Anisotropic Displacement Parameters (×10^4) for (*M*,*R*,*R*,S_p,S_p)-**7.** The anisotropic displacement factor exponent takes the form: $-2\pi^2[h^2a^{*2} \times U_{11} + ... + 2hka^* \times b^* \times U_{12}]$

Atom	U_{11}	U_{22}	U_{33}	U_{23}	U_{13}	U_{12}
Fe	18.4(4)	16.9(4)	13.7(4)	0.2(6)	0.2(5)	-0.4(5)
O1	24(3)	16(2)	20(3)	1(2)	5(2)	-4(2)
O2	28(3)	14(2)	16(3)	-4.9(19)	5(2)	-2(2)
C1	18(4)	23(4)	16(4)	-3(3)	-5(3)	-5(3)
C2	14(4)	20(5)	23(4)	2(3)	2(3)	1(3)
C3	18(4)	11(4)	19(4)	1(3)	3(3)	5(3)
C4	9(4)	22(4)	31(5)	3(4)	3(4)	4(3)
C5	22(5)	29(5)	17(4)	-2(4)	3(4)	-2(4)
C6	13(4)	16(4)	13(4)	3(3)	2(3)	-4(3)
C7	11(4)	19(4)	22(4)	1(4)	-1(3)	5(3)
C8	25(4)	35(6)	13(4)	-5(4)	-3(3)	-1(4)
C9	31(5)	10(4)	16(4)	-2(3)	0(4)	1(3)
C10	17(5)	22(4)	23(5)	3(4)	2(4)	1(4)
C11	12(4)	18(4)	10(4)	-4(3)	4(3)	-4(3)
C12	20(4)	18(4)	18(4)	3(3)	4(3)	4(3)
C13	24(4)	17(4)	18(4)	-4(3)	-4(3)	-4(3)
C14	21(4)	18(4)	26(4)	-7(3)	-1(3)	0(3)
C15	14(3)	17(3)	13(3)	5(4)	2(2)	5(4)
C16	10(4)	18(4)	22(4)	-1(3)	3(3)	0(3)
C17	24(4)	19(4)	10(4)	-1(3)	4(3)	-1(3)
C18	25(5)	26(4)	19(4)	-5(4)	4(3)	-8(3)
C19	13(3)	19(4)	19(4)	-5(4)	2(2)	5(3)
C20	17(4)	14(4)	20(4)	-2(3)	2(3)	0(3)
C21	22(3)	18(4)	18(3)	2(4)	1(3)	-1(4)
C22	25(4)	17(3)	21(4)	-5(3)	6(4)	-3(3)
C23	27(4)	19(4)	16(4)	-4(3)	2(3)	-10(3)

Atom	U_{11}	U_{22}	U_{33}	U_{23}	U_{13}	U_{12}
C24	28(4)	23(4)	18(4)	3(3)	2(3)	-1(3)
C25	22(4)	25(4)	22(4)	2(3)	-3(4)	6(4)
C26	34(5)	23(4)	22(4)	5(3)	5(4)	-8(4)
C27	16(4)	17(3)	17(4)	-4(3)	3(3)	2(3)
C28	20(4)	20(4)	14(4)	2(3)	2(3)	-1(3)
C29	19(4)	31(4)	19(4)	-7(3)	3(3)	-4(3)
C30	34(5)	26(4)	23(4)	-9(3)	11(3)	-4(4)
C31	33(5)	26(4)	12(4)	5(3)	-2(3)	-13(4)
C32	28(4)	23(4)	24(4)	0(4)	1(4)	-3(3)
C33	27(5)	19(4)	26(4)	0(3)	5(3)	2(3)
C34	43(6)	47(5)	18(4)	-7(4)	-6(4)	-12(4)
C35	16(4)	13(3)	13(4)	-3(3)	-2(3)	1(3)
C36	23(4)	16(4)	21(4)	-6(3)	4(3)	-1(3)
C37	33(5)	26(4)	25(4)	-1(4)	2(4)	-7(4)
C38	19(4)	15(3)	13(3)	0(3)	1(3)	0(3)
C39	15(4)	15(3)	19(4)	0(3)	-2(3)	-1(3)
C40	16(4)	16(4)	19(4)	1(3)	2(3)	1(3)
C41	24(4)	16(4)	25(4)	1(3)	5(3)	-3(3)
C42	19(4)	25(4)	22(4)	6(4)	2(3)	-4(3)
C43	74(11)	126(12)	121(13)	12(10)	-23(10)	-52(9)
C44	180(20)	330(40)	70(12)	105(18)	10(13)	110(20)
C45	290(30)	172(17)	49(9)	-46(11)	61(14)	-100(20)
C46	66(11)	320(30)	71(9)	25(18)	15(7)	50(20)
C47	81(11)	188(17)	82(11)	-72(12)	2(8)	-20(12)

Table S5: Bond Lengths in Å for (M,R,R,S_p,S_p)-**7**.

Atom	Atom	Length/Å
Fe	C1	2.072(8)
Fe	C2	2.005(7)
Fe	C3	2.007(8)
Fe	C4	2.048(8)
Fe	C5	2.050(9)
Fe	C6	2.017(8)
Fe	C7	2.087(8)
Fe	C8	2.058(8)
Fe	C9	2.044(8)
Fe	C10	2.030(9)
O1	C12	1.376(9)
O1	C17	1.452(8)
O2	C36	1.470(9)
O2	C40	1.364(8)
C1	C2	1.443(11)
C1	C5	1.435(11)
C1	C14	1.435(11)
C2	C3	1.431(10)
C2	C11	1.468(10)
C3	C4	1.413(11)
C4	C5	1.403(11)
C6	C7	1.440(11)
C6	C10	1.426(10)
C6	C39	1.449(11)
C7	C8	1.440(11)
C7	C42	1.435(11)
C8	C9	1.411(12)
C9	C10	1.424(11)
C11	C12	1.365(9)
C11	C15	1.483(10)
C12	C13	1.438(10)
C13	C14	1.357(10)
C15	C16	1.417(9)
C15	C38	1.406(10)
C16	C17	1.504(10)
C16	C19	1.399(10)
C17	C18	1.514(10)
C19	C20	1.495(10)
C19	C27	1.399(10)
C20	C21	1.388(10)
C20	C25	1.404(10)
C21	C22	1.382(10)
C22	C23	1.379(10)
C23	C24	1.394(11)
C23	C26	1.496(10)
C24	C25	1.374(10)
C27	C28	1.509(10)
C27	C35	1.389(9)
C28	C29	1.374(10)
C28	C33	1.402(10)
C29	C30	1.401(10)
C30	C31	1.387(11)
C31	C32	1.368(11)
C31	C34	1.535(10)
C32	C33	1.372(11)
C35	C36	1.518(10)
C35	C38	1.409(9)
C36	C37	1.526(11)
C38	C39	1.468(10)
C39	C40	1.379(10)
C40	C41	1.419(10)
C41	C42	1.349(10)

Atom	Atom	Length/Å
C43	C44	1.5336
C44	C45	1.5349
C45	C46	1.5351
C46	C47	1.5336

Table S6: Bond Angles in ° for (*M*,*R*,*R*,S_p,S_p)-**7**.

Atom	Atom	Atom	Angle/°
C1	Fe	C7	172.4(3)
C2	Fe	C1	41.4(3)
C2	Fe	C3	41.8(3)
C2	Fe	C4	68.9(3)
C2	Fe	C5	69.7(3)
C2	Fe	C6	98.0(3)
C2	Fe	C7	131.6(3)
C2	Fe	C8	166.0(3)
C2	Fe	C9	130.8(3)
C2	Fe	C10	98.0(3)
C3	Fe	C1	69.4(3)
C3	Fe	C4	40.8(3)
C3	Fe	C5	69.2(3)
C3	Fe	C6	98.0(3)
C3	Fe	C7	107.2(3)
C3	Fe	C8	142.8(3)
C3	Fe	C9	165.3(3)
C3	Fe	C10	124.6(3)
C4	Fe	C1	67.6(3)
C4	Fe	C5	40.0(3)
C4	Fe	C7	114.5(3)
C4	Fe	C8	123.7(3)
C5	Fe	C1	40.7(3)
C5	Fe	C7	145.3(3)
C5	Fe	C8	123.5(3)
C6	Fe	C1	131.8(3)
C6	Fe	C4	131.2(3)
C6	Fe	C5	166.5(3)
C6	Fe	C7	41.0(3)
C6	Fe	C8	69.3(3)
C6	Fe	C9	69.0(3)
C6	Fe	C10	41.3(3)
C8	Fe	C1	145.0(3)
C8	Fe	C7	40.7(3)
C9	Fe	C1	113.7(3)
C9	Fe	C4	153.8(3)
C9	Fe	C5	123.1(3)
C9	Fe	C7	67.9(3)
C9	Fe	C8	40.2(3)
C10	Fe	C1	107.5(3)
C10	Fe	C4	165.2(4)
C10	Fe	C5	143.2(4)
C10	Fe	C7	68.6(3)
C10	Fe	C8	68.7(3)
C10	Fe	C9	40.9(3)
C12	O1	C17	111.6(5)
C40	O2	C36	111.4(5)
C2	C1	Fe	66.8(4)
C5	C1	Fe	68.8(5)
C5	C1	C2	107.2(7)
C5	C1	C14	133.0(7)
C14	C1	Fe	127.9(6)
C14	C1	C2	119.8(7)
C1	C2	Fe	71.8(4)
C1	C2	C11	119.4(6)
C3	C2	Fe	69.2(4)
C3	C2	C1	107.7(7)
C3	C2	C11	132.7(7)
C11	C2	Fe	120.3(5)
C2	C3	Fe	69.0(4)
C4	C3	Fe	71.2(5)
C4	C3	C2	107.4(7)
C3	C4	Fe	68.1(5)
C5	C4	Fe	70.1(6)
C5	C4	C3	109.8(8)
C1	C5	Fe	70.4(5)
C4	C5	Fe	69.9(6)
C4	C5	C1	107.8(8)
C7	C6	Fe	72.1(5)
C7	C6	C39	119.2(7)
C10	C6	Fe	69.9(5)
C10	C6	C7	108.1(8)
C10	C6	C39	132.6(8)
C39	C6	Fe	120.8(6)
C6	C7	Fe	66.9(4)
C6	C7	C8	107.1(7)
C8	C7	Fe	68.6(5)
C42	C7	Fe	129.6(5)
C42	C7	C6	119.9(7)
C42	C7	C8	133.0(8)
C7	C8	Fe	70.8(5)
C9	C8	Fe	69.4(5)
C9	C8	C7	108.0(7)
C8	C9	Fe	70.4(5)
C8	C9	C10	109.0(7)
C10	C9	Fe	69.0(5)
C6	C10	Fe	68.9(6)
C9	C10	Fe	70.1(5)
C9	C10	C6	107.7(8)
C2	C11	C15	126.7(6)
C12	C11	C2	115.2(6)
C12	C11	C15	118.0(6)
O1	C12	C13	115.7(6)
C11	C12	O1	120.3(6)
C11	C12	C13	123.9(6)
C14	C13	C12	120.1(7)
C13	C14	C1	119.1(7)
C16	C15	C11	114.3(7)
C38	C15	C11	126.5(6)
C38	C15	C16	118.8(6)
C15	C16	C17	116.3(6)
C19	C16	C15	121.0(7)
C19	C16	C17	122.7(6)
O1	C17	C16	109.2(5)
O1	C17	C18	110.1(6)
C16	C17	C18	114.2(6)

Atom	Atom	Atom	Angle/°
C16	C19	C20	118.8(6)
C16	C19	C27	119.9(6)
C27	C19	C20	121.3(6)
C21	C20	C19	120.8(6)
C21	C20	C25	116.7(7)
C25	C20	C19	122.5(6)
C22	C21	C20	121.2(7)
C23	C22	C21	121.7(7)
C22	C23	C24	117.9(7)
C22	C23	C26	121.2(7)
C24	C23	C26	120.9(7)
C25	C24	C23	120.4(7)
C24	C25	C20	122.1(7)
C19	C27	C28	118.6(6)
C35	C27	C19	119.0(6)
C35	C27	C28	121.9(6)
C29	C28	C27	123.8(7)
C29	C28	C33	117.2(7)
C33	C28	C27	118.8(6)
C28	C29	C30	120.7(7)
C31	C30	C29	121.0(7)
C30	C31	C34	122.0(7)
C32	C31	C30	118.3(7)
C32	C31	C34	119.6(8)
C31	C32	C33	120.8(7)
C32	C33	C28	122.0(7)
C27	C35	C36	121.5(6)
C27	C35	C38	122.0(6)
C38	C35	C36	116.5(6)
O2	C36	C35	108.9(6)
O2	C36	C37	110.2(6)
C35	C36	C37	115.6(6)
C15	C38	C35	118.6(6)
C15	C38	C39	126.6(6)
C35	C38	C39	114.8(6)
C6	C39	C38	127.4(6)
C40	C39	C6	115.6(7)
C40	C39	C38	117.0(6)
O2	C40	C39	119.9(6)
O2	C40	C41	116.7(6)
C39	C40	C41	123.0(7)
C42	C41	C40	121.0(7)
C41	C42	C7	118.8(7)
C43	C44	C45	113.3
C44	C45	C46	113.8
C47	C46	C45	113.3

Table S7: Torsion Angles in ° for (*M*,*R*,*R*,S_p,S_p)-**7**.

Atom	Atom	Atom	Atom	Angle/°
Fe	C1	C2	C3	60.1(5)
Fe	C1	C2	C11	-115.1(7)
Fe	C1	C5	C4	-60.2(7)
Fe	C1	C14	C13	88.9(9)
Fe	C2	C3	C4	61.2(6)
Fe	C2	C11	C12	-102.0(7)
Fe	C2	C11	C15	74.8(8)
Fe	C3	C4	C5	57.6(7)
Fe	C4	C5	C1	60.6(6)
Fe	C6	C7	C8	-56.8(6)
Fe	C6	C7	C42	123.6(7)
Fe	C6	C10	C9	59.6(6)
Fe	C6	C39	C38	74.4(9)
Fe	C6	C39	C40	-103.0(7)
Fe	C7	C8	C9	-59.6(6)
Fe	C7	C42	C41	87.6(9)
Fe	C8	C9	C10	-58.3(6)
Fe	C9	C10	C6	-58.8(7)
O1	C12	C13	C14	-179.9(7)
O2	C40	C41	C42	-178.7(7)
C1	C2	C3	Fe	-61.8(5)
C1	C2	C3	C4	-0.6(9)
C1	C2	C11	C12	-17.0(10)
C1	C2	C11	C15	159.9(7)
C2	C1	C5	Fe	55.8(5)
C2	C1	C5	C4	-4.4(10)
C2	C1	C14	C13	6.3(11)
C2	C3	C4	Fe	-59.8(5)
C2	C3	C4	C5	-2.2(10)
C2	C11	C12	O1	-167.9(6)
C2	C11	C12	C13	15.9(11)
C2	C11	C15	C16	151.5(7)

Atom	Atom	Atom	Atom	Angle/°
C2	C11	C15	C38	-35.3(11)
C3	C2	C11	C12	169.2(8)
C3	C2	C11	C15	-13.9(13)
C3	C4	C5	Fe	-56.4(6)
C3	C4	C5	C1	4.1(11)
C5	C1	C2	Fe	-57.0(6)
C5	C1	C2	C3	3.1(9)
C5	C1	C2	C11	-172.2(7)
C5	C1	C14	C13	-175.4(9)
C6	C7	C8	Fe	55.8(5)
C6	C7	C8	C9	-3.8(9)
C6	C7	C42	C41	4.0(11)
C6	C39	C40	O2	-170.0(6)
C6	C39	C40	C41	16.4(11)
C7	C6	C10	Fe	-62.3(6)
C7	C6	C10	C9	-2.8(11)
C7	C6	C39	C38	160.0(7)
C7	C6	C39	C40	-17.4(11)
C7	C8	C9	Fe	60.5(5)
C7	C8	C9	C10	2.2(9)
C8	C7	C42	C41	-175.3(8)
C8	C9	C10	Fe	59.2(6)
C8	C9	C10	C6	0.4(11)
C10	C6	C7	Fe	60.9(6)
C10	C6	C7	C8	4.1(10)
C10	C6	C7	C42	-175.5(7)
C10	C6	C39	C38	-15.8(15)
C10	C6	C39	C40	166.8(10)
C11	C2	C3	Fe	112.6(9)
C11	C2	C3	C4	173.7(8)
C11	C12	C13	C14	-3.6(12)
C11	C15	C16	C17	1.6(9)
C11	C15	C16	C19	178.3(6)
C11	C15	C38	C35	177.5(7)
C11	C15	C38	C39	-2.7(12)
C12	O1	C17	C16	-59.0(7)
C12	O1	C17	C18	67.1(7)
C12	C11	C15	C16	-31.8(9)
C12	C11	C15	C38	141.5(7)
C12	C13	C14	C1	-8.2(11)
C14	C1	C2	Fe	121.6(7)
C14	C1	C2	C3	-178.3(7)
C14	C1	C2	C11	6.5(11)
C14	C1	C5	Fe	-122.6(9)
C14	C1	C5	C4	177.2(8)
C15	C11	C12	O1	15.0(10)
C15	C11	C12	C13	-161.2(6)
C15	C16	C17	O1	42.0(8)
C15	C16	C17	C18	-81.7(8)
C15	C16	C19	C20	177.9(6)
C15	C16	C19	C27	0.7(10)
C15	C38	C39	C6	-34.1(12)
C15	C38	C39	C40	143.3(7)
C16	C15	C38	C35	-9.6(9)
C16	C15	C38	C39	170.3(7)
C16	C19	C20	C21	-71.0(9)
C16	C19	C20	C25	107.5(7)
C16	C19	C27	C28	-173.5(6)
C16	C19	C27	C35	-0.7(9)
C17	O1	C12	C11	31.6(9)
C17	O1	C12	C13	-151.9(6)
C17	C16	C19	C20	-5.6(10)

Atom	Atom	Atom	Atom	Angle/˚
C17	C16	C19	C27	177.2(6)
C19	C16	C17	O1	-134.6(6)
C19	C16	C17	C18	101.7(8)
C19	C20	C21	C22	178.7(7)
C19	C20	C25	C24	-178.2(7)
C19	C27	C28	C29	-80.7(9)
C19	C27	C28	C33	94.2(8)
C19	C27	C35	C36	174.2(6)
C19	C27	C35	C38	-4.7(10)
C20	C19	C27	C28	9.4(9)
C20	C19	C27	C35	-177.8(6)
C20	C21	C22	C23	-0.8(11)
C21	C20	C25	C24	0.4(10)
C21	C22	C23	C24	1.0(10)
C21	C22	C23	C26	177.6(7)
C22	C23	C24	C25	-0.6(10)
C23	C24	C25	C20	-0.1(11)
C25	C20	C21	C22	0.1(11)
C26	C23	C24	C25	-177.2(7)
C27	C19	C20	C21	106.2(8)
C27	C19	C20	C25	-75.3(9)
C27	C28	C29	C30	174.3(7)
C27	C28	C33	C32	-174.7(7)
C27	C35	C36	O2	-144.5(6)
C27	C35	C36	C37	90.8(8)
C27	C35	C38	C15	9.9(10)
C27	C35	C38	C39	-170.0(6)
C28	C27	C35	C36	-13.2(10)
C28	C27	C35	C38	167.9(6)
C28	C29	C30	C31	0.0(11)
C29	C28	C33	C32	0.5(11)
C29	C30	C31	C32	0.7(11)
C29	C30	C31	C34	-176.8(7)
C30	C31	C32	C33	-0.8(11)
C31	C32	C33	C28	0.3(12)
C33	C28	C29	C30	-0.6(11)
C34	C31	C32	C33	176.8(7)
C35	C27	C28	C29	106.7(8)
C35	C27	C28	C33	-78.4(9)
C35	C38	C39	C6	145.8(7)
C35	C38	C39	C40	-36.9(9)
C36	O2	C40	C39	36.5(9)
C36	O2	C40	C41	-149.5(6)
C36	C35	C38	C15	-169.0(6)
C36	C35	C38	C39	11.1(9)
C38	C15	C16	C17	-172.2(6)
C38	C15	C16	C19	4.5(10)
C38	C35	C36	O2	34.5(8)
C38	C35	C36	C37	-90.3(8)
C38	C39	C40	O2	12.3(10)
C38	C39	C40	C41	-161.3(7)
C39	C6	C7	Fe	-115.8(7)
C39	C6	C7	C8	-172.7(7)
C39	C6	C7	C42	7.8(11)
C39	C6	C10	Fe	113.8(10)
C39	C6	C10	C9	173.4(8)
C39	C40	C41	C42	-5.0(11)
C40	O2	C36	C35	-58.9(7)
C40	O2	C36	C37	69.0(7)
C40	C41	C42	C7	-5.8(11)
C42	C7	C8	Fe	-124.8(9)
C42	C7	C8	C9	175.6(9)

Atom	Atom	Atom	Atom	Angle/°
C43	C44	C45	C46	180.0
C44	C45	C46	C47	-180.0

Table S8: Hydrogen Fractional Atomic Coordinates (×10[4]) and Equivalent Isotropic Displacement Parameters (Å[2]×10[3]) for (*M*,*R*,*R*,*S*p,*S*p)-**7**. U_{eq} is defined as 1/3 of the trace of the orthogonalised U_{ij}.

Atom	x	y	z	U_{eq}
H3	6123.38	6125.06	1401.21	19
H4	5821.91	5806.17	29.6	24
H5	5938.59	2845.36	-302.3	27
H8	9085.07	4452.4	-310.79	29
H9	9089.84	1752.55	350.53	23
H10	8715.88	2245.87	1710.1	25
H13	6048.76	-966	1838.51	24
H14	6110.03	-112.78	621.14	26
H17	5966.52	1375.15	4051.44	21
H18A	4508.53	2696.48	2938.21	35
H18B	4343.65	2958.58	3806.57	35
H18C	4190.54	1184.58	3455.09	35
H21	5027.16	4398.73	4823.74	23
H22	4398.51	3453.66	5953.16	25
H24	7493.1	1470.62	6352.46	28
H25	8131.75	2441.99	5233.83	28
H26A	5610.6	2641.28	7375.94	40
H26B	5869.49	795.55	7149.25	40
H26C	4637.61	1569.27	6986.21	40
H29	7019.3	7587.13	5141.8	28
H30	7878.86	8379.65	6252.91	33
H32	10651.22	5699.07	5770.93	30
H33	9808.71	4904.18	4678.4	29
H34A	9499.59	8445.02	7095.07	54
H34B	10710.9	7841.5	6790.7	54
H34C	9879.01	6587.43	7199.27	54
H36	8603.07	8284.94	3716.94	24
H37A	10276.38	6552.09	2885.79	42
H37B	10333.77	6882.15	3755.7	42
H37C	10445.56	8366.34	3188.54	42
H41	8794.45	9320.17	1315	26
H42	8901.02	7835.58	240.15	27
H43A	7772.74	5070.29	7465.05	161
H43B	7278.1	3758.44	8033.34	161
H43C	8507.52	4559.24	8175.42	161
H44A	7108.24	5778.9	8952.06	235
H44B	6386.53	6304.84	8239.18	235
H45A	7896.44	8079.6	7875.54	206
H45B	8617.81	7553.92	8588.2	206
H46A	6493.59	9301.77	8650.35	181
H46B	7215.98	8776.75	9363.11	181
H47A	7808.58	11243.23	8324.87	175
H47B	8765.85	10499.11	8861.27	175
H47C	7692.41	11444.99	9199.92	175

References

1. Bovonsombat, P.; Leykajarakul, J.; Khan, C.; Pla-on, K.; Krause, M. M.; Khanthapura, P.; Ali, R.; Doowa, N., Regioselective iodination of phenol and analogues using N-iodosuccinimide and p-toluenesulfonic acid. *Tetrahedron Lett.* **2009**, *50*, 2664-2667.
2. Alexandrová, Z.; Stará, I. G.; Sehnal, P.; Teplý, F.; Starý, I.; Šaman, D.; Fiedler, P., Synthetic studies toward chiral aromatic triynes as key substrates for the asymmetric synthesis of helicene-like molecules. *Collect. Czechoslov. Chem. Commun.* **2004**, *69*, 2193-2211.
3. Geny, A.; Agenet, N.; Iannazzo, L.; Malacria, M.; Aubert, C.; Gandon, V., Air-Stable {(C_5H_5)Co} Catalysts for [2+2+2] Cycloadditions. *Angew. Chem. Int. Ed.* **2009,** *48*, 1810-1813.
4. Fulmer, G. R.; Miller, A. J. M.; Sherden, N. H.; Gottlieb, H. E.; Nudelman, A.; Stoltz, B. M.; Bercaw, J. E.; Goldberg, K. I., NMR Chemical Shifts of Trace Impurities: Common Laboratory Solvents, Organics, and Gases in Deuterated Solvents Relevant to the Organometallic Chemist. *Organometallics* **2010,** *29*, 2176-2179.
5. Nejedlý, J.; Šámal, M.; Rybáček, J.; Tobrmanová, M.; Szydlo, F.; Coudret, C.; Neumeier, M. Vacek, J.; Vacek Chocholoušová, J.; Buděšínský, M.; Šaman, D.; Bednárová, L.; Sieger, L.; Stará, I.G.; Starý. I., Synthesis of Long Oxahelicenes by Polycyclization in a Flow Reactor. *Angew. Chem. Int. Ed.* **2017**, *56*, 5839-5843.
6. Shestakov, A. N.; Pankova, A. S.; Kuznetsov, M. A., Cycloisomerization – a straightforward way to benzo[*h*]quinolines and benzo[*c*]acridines. *Chem. Heterocycl. Comdp.* **2017,** *53*, 1103-1113.
7. Zhang, Y.; Wang, M.; Li, P.; Wang, L., Iron-Promoted Tandem Reaction of Anilines with Styrene Oxides via C–C Cleavage for the Synthesis of Quinolines. *Org. Lett.* **2012,** *14*, 2206-2209.
8. Kitamura, M.; Sakata, R.; Tashiro, N.; Ikegami, A.; Okauchi, T., Synthesis of Diazonaphthoquinones from Naphthols by Diazo-Transfer Reaction. *Bull. Chem. Soc. Jap.* **2015,** *88*, 824-833.
9. Hu, P.; Liu, B.; Wang, F.; Mi, R.; Li, X.-X.; Li, X., A Stereodivergent–Convergent Chiral Induction Mode in Atroposelective Access to Biaryls via Rhodium-Catalyzed C–H Bond Activation. *ACS Catalysis* **2022,** *12*, 13884-13896.
10. Pan, C.; Yin, S.-Y.; Wang, S.-B.; Gu, Q.; You, S.-L., Oxygen-Linked Cyclopentadienyl Rhodium(III) Complexes-Catalyzed Asymmetric C−H Arylation of Benzo[*h*]quinolines with 1-Diazonaphthoquinones. *Angew. Chem.Int. Ed.* **2021,** *60*, 15510-15516.
11. Gaussian 16, Revision C.01, Frisch, M. J., Trucks, G. W., Schlegel, H. B., Scuseria, G. E., Robb, M. A., Cheeseman, J. R., Scalmani, G., Barone, V., Petersson, G. A., Nakatsuji, H., Li, X., Caricato, M., Marenich, A. V., Bloino, J., Janesko, B. G., Gomperts, R., Mennucci, B., Hratchian, H. P., Ortiz, J. V., Izmaylov, A. F., Sonnenberg, J. L., Williams-Young, D., Ding, F., Lipparini, F., Egidi, F., Goings, J., Peng, B., Petrone, A., Henderson, T, Ranasinghe, D., Zakrzewski, V. G., Gao, J., Rega, N., Zheng, G., Liang, W., Hada, M., Ehara, M., Toyota, K., Fukuda, R., Hasegawa, J., Ishida, M., Nakajima, T., Honda, Y., Kitao, O., Nakai, H., Vreven, T., Throssell, K., Montgomery, Jr., J. A., Peralta, J. E., Ogliaro, F., Bearpark, M. J., Heyd, J. J., Brothers, E. N., Kudin, K. N., Staroverov, V. N., Keith, T. A., Kobayashi, R., Normand, J., Raghavachari, K., Rendell, A. P., Burant, J. C., Iyengar, S. S., Tomasi, J., Cossi, M., Millam, J. M., Klene, M., Adamo, C., Cammi, R., Ochterski, J. W., Martin, R. L., Morokuma, K., Farkas, O., Foresman, J. B., Fox, D. J., Gaussian, Inc., Wallingford CT, 2016.
12. Becke, A. D., Density-functional thermochemistry. III. The role of exact exchange. *J. Chem. Phys.* **1993**, *98*, 5648-5652.
13. Weigend, F.; Ahlrichs, R., Balanced basis sets of split valence, triple zeta valence and quadruple zeta valence quality for H to Rn: Design and assessment of accuracy. *Phys. Chem. Chem. Phys.* **2005**, *7*, 3297-3305.
14. Grimme, S.; Antony, J.; Ehrlich, S.; Krieg, H., A consistent and accurate ab initio parametrization of density functional dispersion correction (DFT-D) for the 94 elements H-Pu. *J. Chem. Phys.* **2010**, *132*, 154104.
15. Adamo, C.; Barone, V., Toward reliable density functional methods without adjustable parameters: The PBE0 model. *J. Chem. Phys.* **1999**, *110*, 6158-69.
16. Miertuš, S.; Scrocco, E.; Tomasi, J., Electrostatic interaction of a solute with a continuum. A direct utilizaion of AB initio molecular potentials for the prevision of solvent effects. *Chemical Physics* **1981**, *55*, 117-129.
17. Miertuš, S.; Tomasi, J., Approximate evaluations of the electrostatic free energy and internal energy changes in solution processes. *Chemical Physics* **1982**, *65*, 239-245.
18. Hillier, A. C.; Sommer, W. J.; Yong, B. S.; Petersen, J. L.; Cavallo, L.; Nolan, S. P., A Combined Experimental and Theoretical Study Examining the Binding of *N*-Heterocyclic Carbenes (NHC) to the Cp*RuCl (Cp* = η^5-C_5Me_5) Moiety: Insight into Stereoelectronic Differences between Unsaturated and Saturated NHC Ligands. *Organometallics* **2003**, *22*, 4322–4326.

19. Clavier, H.; Nolan, S. P., Percent buried volume for phosphine and *N*-heterocyclic carbeneligands: steric properties in organometallic chemistry. *Chem. Commun.* **2010**, *46*, 841–861.
20. Pan, C.; Yin, S.-Y.; Wang, S.-B.; Gu, Q.; You, S.-L., Oxygen-Linked Cyclopentadienyl Rhodium(III) Complexes-Catalyzed Asymmetric C−H Arylation of Benzo[h]quinolines with 1-Diazonaphthoquinones. *Angew. Chem. Int. Ed.* **2021**, *60*, 15510–15516.
21. Meng, E. C.; Goddard, T. D.; Pettersen, E. F.; Couch, G. S.; Pearson, Z. J.; Morris, J. H.; Ferrin, T.E., UCSF ChimeraX: Structure visualization for researchers, educators, and developers. *Protein Sci.* **2023**, *32*, e4792.
22. ChimeraX, https://www.rbvi.ucsf.edu/chimerax, last accessed 24.4.2024.
23. Schaefer, A. J.; Ingman, V. M.; Wheeler, S. E., SEQCROW: A ChimeraX bundle to facilitate quantum chemical applications to complex molecular systems. *J. Comp. Chem.* **2021**, *42*, 1750-1754.
24. Ingman, V. M.; Schaefer, A. J.; Andreola, L. R.; Wheeler, S. E., QChASM: Quantum chemistry automation and structure manipulation. *WIREs Comp. Mol. Sci.* **2021**, *11*, e1510.
25. Falivene, L.; Credendino, R.; Poater, A.; Petta, A.; Serra, L.; Oliva, R.; Scarano, V.; Cavallo, L., SambVca 2. A Web Tool for Analyzing Catalytic Pockets with Topographic Steric Maps. *Organometallics* **2016**, *35*, 2286-2293.
26. a) Sheldrick, G.M., Crystal structure refinement with ShelXL, *Acta Cryst.* **2015**, *C71*, 3-8. b) Sheldrick, G.M., ShelXT-Integrated space-group and crystal-structure determination, *Acta Cryst.* **2015**, *A71*, 3-8.
27. Dolomanov, O.V.; Bourhis, L.J.; Gildea, R.J.; Howard, J.A.K.; Puschmann, P., Olex2: A complete structure solution, refinement and analysis program. *J. Appl. Cryst.* **2009**, *42*, 339-341.
28. APEX3, Bruker AXS, Medison, Wisconsin, USA.
29. SAINT - Software for the Integration of CCD Detector System Bruker Analytical X-ray Systems, Bruker AXS, Madison, Wisconsin, USA.
30. SADABS 2016/2, Bruker AXS, Madison, Wisconsin, USA.
31. Flack, H.D., On enantiomorph-polarity estimation. *Acta Cryst.* **1983**, *A39*, 876-881.
32. Spek, A.L., Structure validation in chemical crystallography. *Acta Cryst.* **2009**, *D65*, 148–155.
